# Simple and versatile electrochemical synthesis of highly substituted 2,1-benzisoxazoles†

**DOI:** 10.1039/d4ob01875c

**Published:** 2024-12-03

**Authors:** Marola S. Lenhard, Johannes Winter, Alexander Sandvoß, María de Jesús Gálvez-Vázquez, Dieter Schollmeyer, Siegfried R. Waldvogel

**Affiliations:** a Department of Chemistry, Johannes Gutenberg University Duesbergweg 10–14 55128 Mainz Germany siegfried.waldvogel@cec.mpg.de; b Karlsruhe Institute of Technology (KIT), Institute of Biological and Chemical Systems – Functional Molecular Systems (IBCS-FMS) Kaiserstraße 12 76131 Karlsruhe Germany; c Max-Planck-Institute for Chemical Energy Conversion Stiftstraße 34–36 45470 Mülheim an der Ruhr Germany

## Abstract

A sustainable, general and scalable electrochemical protocol for direct access to 3-(acylamidoalkyl)-2,1-benzisoxazoles by cathodic reduction of widely accessible nitro arenes is established. The method is characterised by a simple undivided set-up under constant current conditions, inexpensive and reusable carbon-based electrodes, and environmentally benign reaction conditions. The versatility of the developed protocol is demonstrated on 39 highly diverse examples with up to 81% yield. A 50-fold scale-up electrolysis highlights its relevance for preparative applications.

## Abstract 2

A simple, sustainable and scalable electrochemical method providing direct access to a highly diverse range of 3-(acylamidoalkyl)-2,1-benzisoxazoles by cathodic reduction of widely accessible nitro arenes was established.

## Graphical abstract

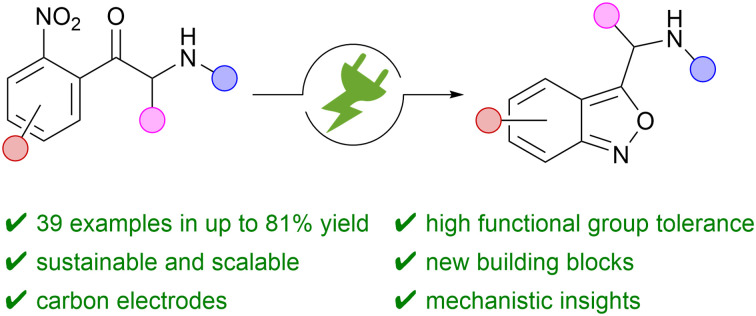

## Introduction

The isoxazole ring represents an important structural motif in medicinal chemistry and organic synthesis.^1,2^ Compounds bearing the isoxazole moiety exhibit a broad spectrum of biological activities including anticancer, antibacterial, antiviral, antifungal, antimicrobial, anti-tuberculosis, and anti-inflammatory properties.^3^ Muscimol (1), for example, is a hallucinogen found in *Amanita muscaria*.^4^ 2,1-Benzisoxazoles, also called anthranils, are derivatives of isoxazole with potential applications in drug discovery.^5–9^ Several 2,1-benzisoxazoles, 2 in particular, have been investigated as MAO inhibitors, serving as novel drug candidates for the treatment of neurological disorders.^5^ Furthermore, the 2,1-benzisoxazole 3 serves as the Pim-1 kinase inhibitor for the therapy of various forms of cancer.^6^ Additionally, antibacterial activity has been observed in different studies investigating other 2,1-benzisoxazoles.^7,8^ Also they are applied in the treatment of central nervous system abnormalities^9^ and serve as key intermediates in the synthesis of various pharmaceuticals,^10,11^ such as antimicrobial,^12^ antitubulin,^13^ and anti-inflammatory drugs.^14^

**Fig. 1:**
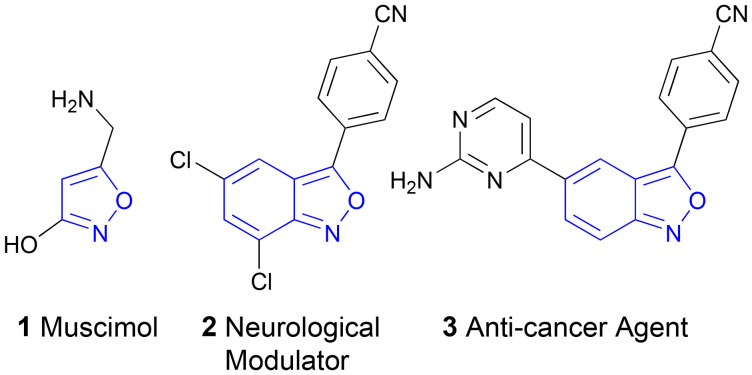
Biologically active isoxazoles and anthranils: potent hallucinogen muscimol (1), neurological modulator for the treatment of mental disorders (2), and the drug candidate showing anti-cancer activity (3).

Moreover, anthranils are used as precursors for organic functional materials and industrial surfactants and as highly versatile synthons in organic synthesis.^2,15,16^ For instance, various transition metal-catalysed cross-coupling reactions and C–H amination and annulation reactions have been developed for the formation of valuable N-containing molecules (Fig. 1).^17^

**Fig. 2:**
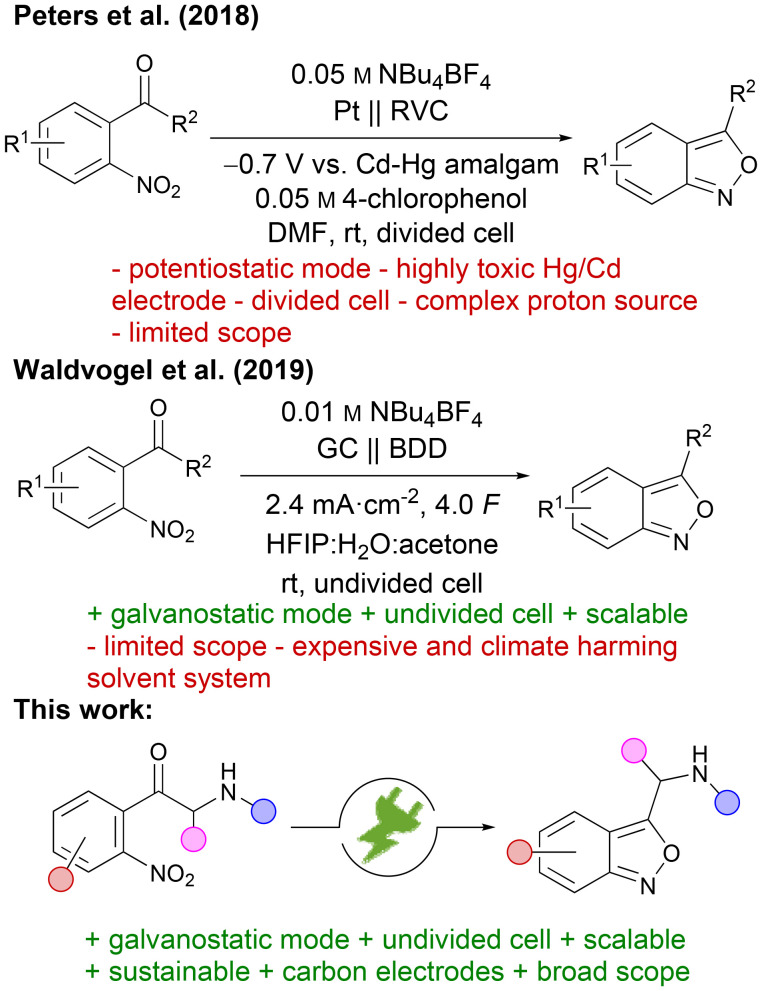
Previous work on the electrochemical synthesis of 2,1-benzisoxazoles.

In addition, 2,1-benzisoxazoles are used as substrates for [4 + 3] and [4 + 2] cycloaddition reactions, yielding benzazepines and benzodiazepines with 1,3-dipolar species,^18^ or benzoxazinone and quinoline scaffolds with dienophiles.^19^ Conventionally, 2,1-benzisoxazoles are constructed by partial reduction and subsequent dehydrative cyclisation of *ortho*-nitroacylbenzenes, using either stoichiometric amounts of reducing agents such as SnCl_2_,^20^ catalytic hydrogenation at Pt-supported nanoparticles,^16,21^ or nitroreductases under photoenzymatic conditions.^22^ Other strategies for the synthesis of anthranils include the oxidative heterocyclisation of *ortho*-substituted anilines,^11,23^ the thermolytic or metal-catalysed cyclisation of 2-azidoacylbenzenes,^24^ the condensation of nitro benzenes with arylacetonitriles,^25^ and the acid- or base-catalysed dehydrative cyclisation of 2-nitrobenzyl compounds.^26^ However, these methods often suffer from drawbacks such as the need for stoichiometric amounts of toxic reducing agents, high hydrogen pressure, expensive catalysts, and elevated temperatures, and low to moderate yield of the desired product. Notwithstanding the necessity of a specialised reaction set-up, organic electrochemistry has emerged in recent years as a versatile and sustainable tool for overcoming the use of stoichiometric amounts of toxic reducing agents and expensive catalysts. Furthermore, electrochemical reactions can be conducted under ambient conditions without the need for high pressures of hydrogen or elevated temperatures.^27^ Since electrons are applied as surrogates for common reagents, hazardous or toxic reducing agents as well as the reagent waste can be avoided. If electricity from sustainable sources is employed, electrosynthesis can even be considered almost waste- and pollutant-free.^28^ Precise control of electrochemical parameters prevents a thermal runaway of the reaction as switching off the electricity stops the reaction, resulting in an inherently safe process.^29^ One important parameter is the electrolysis mode. In the potentiostatic mode, the reaction is run at constant potential to achieve high selectivity. This requires a three-electrode set-up with an additional reference electrode, which increases the cost and complexity of the set-up and inhibits scalability. Additionally, longer reaction times are needed to reach full conversion because the current decreases with the decreasing substrate concentration over time. In the galvanostatic mode, on the other hand, the reaction is run at constant current, so a two-electrode set-up is sufficient. Selectivity can be lower than that in the potentiostatic mode since the potential automatically adjusts to the process with the smallest potential difference. However, reaction times are usually shorter, the set-up is simpler and scale-up is easier in the galvanostatic mode, which is why it is the economically more favourable choice.^30^ As a result, electro-organic chemistry has become a valuable tool in the organic chemist's toolbox for the selective synthesis of compounds which are difficult to access by conventional methods.^31–35^ For instance, various nitrogen heterocycles with an exocyclic N,O moiety could be obtained by electrochemical reduction of nitro groups (Fig. 2).^35–38^

Early studies on the formation of anthranils by electroreduction of 2-nitroacylbenzenes have been conducted with Hg cathodes, identifying anthranils as possible products without exploring any synthetic applications.^39^ The first general synthetic method by Kim *et al.* involved a sacrificial lead cathode,^40^ whereas Peters *et al.* later achieved similar results with stable reticulated vitreous carbon (RVC) electrodes.^41^ However, both approaches were conducted in a divided cell under potentiostatic conditions, limiting the scalability due to the complexity of the set-up. In addition, the method of Peters *et al.* required the addition of a large excess (10 eq.) of chlorophenol as a proton donor and achieved a significantly worse yield and selectivity when it was transferred to galvanostatic conditions (60% yield under galvanostatic conditions as opposed to 90% under potentiostatic conditions). Noteworthily, such cadmium electrodes are prone to cathodic corrosion contaminating the products with highly toxic heavy metals.^42^ Moreover, important experimental information for the method from Peters *et al.* is missing since no ESI was published. A simpler and scalable synthesis of 2,1-benzisoxazoles was developed by this group, using an undivided cell, constant current conditions and protic solvents (HFIP/water) which eliminate the need for additional proton donors.^43^ In contrast to previous methods, the reaction was scaled up successfully and also electron-withdrawing groups were tolerated at the nitro arene core. However, the scope of the synthesised anthranils was still very limited, including only one 3-substituted product whose synthesis required a large excess of applied charge (7 F instead of the theoretical amount of 4 F). Although the work with hexafluoroisopropanol (HFIP) can be conducted and recycled on a larger scale, the environmental footprint is tremendous.^44^

Herein, a simple, sustainable and versatile electrochemical method for the reduction of 2-nitroacylbenzenes is reported, yielding a diverse range of 2,1-benzisoxazoles in moderate to very good yields. The simplest and readily available undivided set-up in combination with constant current mode allows for easy scale-up and the reaction conditions take sustainable aspects into account, avoiding the use of environmentally harmful and expensive fluorinated solvents, supporting electrolytes or additional organic additives. Moreover, the scope of the reaction provides access to a broad and highly diverse variety of 3-substituted 2,1-benzisoxazoles, enabling novel building blocks for organic synthesis and medicinal chemistry.

## Results and discussion

### Optimisation of the electrolysis conditions

4a was chosen as the test substrate and was synthesised in 3 steps starting from 2-bromo-2′-nitroacetophenone. The initial electrolysis conditions for the reductive cyclisation of 4a were chosen based on previous work by Waldvogel *et al.*^36^ A water/methanol mixture was chosen as a green solvent and acetone was added to ensure the complete dissolution of the starting material 4a. Sulphuric acid in a moderate concentration (0.5 M) serves both as a supporting electrolyte and as a proton donor catalysing the cyclo-condensation. Initially, a divided cell was employed to prevent oxidative side reactions. The cell was equipped with a glassy carbon (GC) anode and a boron-doped diamond (BDD) cathode. BDD is an innovative carbon-based electrode material which exhibits unique reactivity towards the electrochemical conversion of various substrates and can be produced sustainably using methane as a renewable carbon source.^45^ A theoretical amount of applied charge necessary for the reduction to the corresponding hydroxylamines (4 F) was used and a current density of 3.7 mA cm^−2^ was used.

**Fig. 3:**
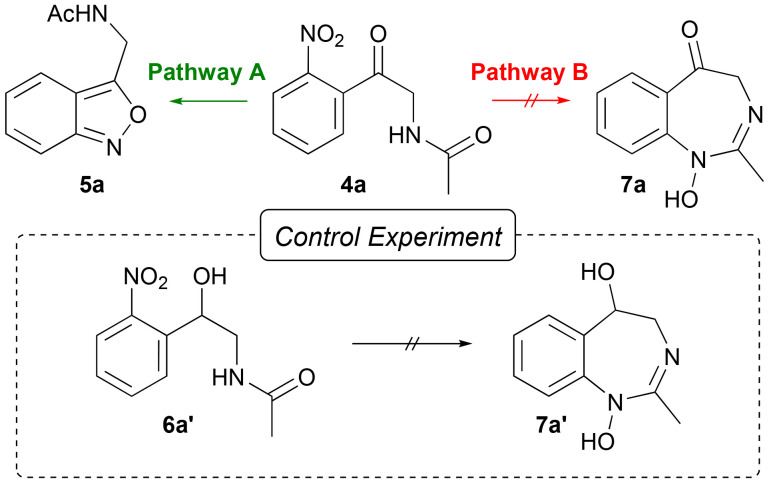
Possible reaction pathways for the electrochemical reduction of *N*-(2-nitrophenacyl)acetamide 4a and a control experiment of the reduction of *N*-(2-hydroxy-2-(2-nitrophenyl)ethyl)acetamide 6a′.

Under these conditions, the desired 2,1-benzisoxazole 5a was detected in 72% yield (Table 1, entry 1). The structure of the products was later confirmed by X-ray analysis of a suitable single crystal of 5ac (CCDC 2389090†). The reduction of 6a′ showed no formation of the corresponding 1,3-benzodiazepine 7a′ (Fig. 3, control experiment) detected by LC-MS analysis. This indicates that the lack of reactivity of the amide inhibits the formation of 7a (Fig. 3, pathway B) and solely results in the formation of 5a (Fig. 3, pathway A). To optimise the yield of 5a, a linear screening of the electrolysis conditions was performed. First, different supporting electrolytes were tested. Acetate buffer as a weaker and biogenic alternative to sulphuric acid significantly decreased the yield to 41% (Table 1, entry 2).

**Table 1:** Screening of electrolysis parameters for the optimisation of the synthesis of 3-(acetamidomethyl)benzo[*c*]isoxazole 5a

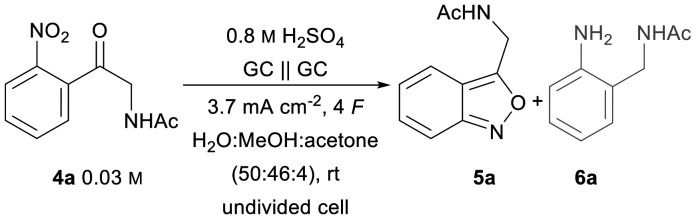		
Entry	Deviation from optimised conditions	Yield^a^ (%)
1	Divided cell, BDD cathode, 0.5 m H_2_SO_4_	72
2	Divided cell, BDD cathode, 5 m AcOH + 0.27 m NaOAc in MeOH	41
3	Divided cell, BDD cathode, 5 m HCOOH + 0.5 m NaHCOO in MeOH	62
4	Divided cell, 1.0 m H_2_SO_4_	79
5	1.0 m H_2_SO_4_	82
6	1.0 m H_2_SO_4_, 4.5 mA cm^−2^	57
7	1.0 m H_2_SO_4_, 5 *F*	41
8	None	87 (81^b^)

a Yield determined by ^1^H NMR spectroscopy using 1,3,5-trimethoxybenzene as an internal standard.

b Isolated yield; GC = glassy carbon, BDD = boron-doped diamond.

In previous studies, formate buffer in methanol gave excellent results as a supporting electrolyte.^35,38^ In this case, however, it led to a slightly lower yield of 62% (Table 1, entry 3). Glassy carbon as the cathode material in combination with 1 M sulphuric acid increased the yield to 79% (Table 1, entry 4). Fortunately, using an undivided cell resulted in an even higher yield of 82% (Table 1, entry 5), which enabled a tremendous simplification of the set-up. Increasing the current density to 4.5 mA cm^−2^ led to both, a worse yield of 57% and a decrease in selectivity of the desired product 5a, resulting in a more complex impurity profile detected by LC-MS analysis (Table 1, entry 6). A higher amount of applied charge of 5 F led to a drop in yield to 41% due to a reductive ring opening reaction of 2,1-benzisoxazole to 6a (Table 1, entry 7). Next, the solvent system was investigated again in the undivided set-up (ESI, Table S5†). Without using acetone as the additive, the yield decreased to 70%. Increasing the methanol/water ratio to 3 : 1 or switching to an ethanol/water mixture instead of adding acetone resulted in yields of 28% and 77%, respectively, confirming the necessity of acetone for the complete dissolution of the substrate and a successful conversion. Furthermore, no pinacol condensation product could be detected in the crude reaction mixture by LC-MS or GC-MS analysis. Finally, the concentration of sulphuric acid was screened (ESI, Table S5†), revealing 0.8 M sulphuric acid to be the most effective concentration with a yield of 87% (Table 1, entry 8).

### Scope of the reductive cyclisation

**Fig. 4:**
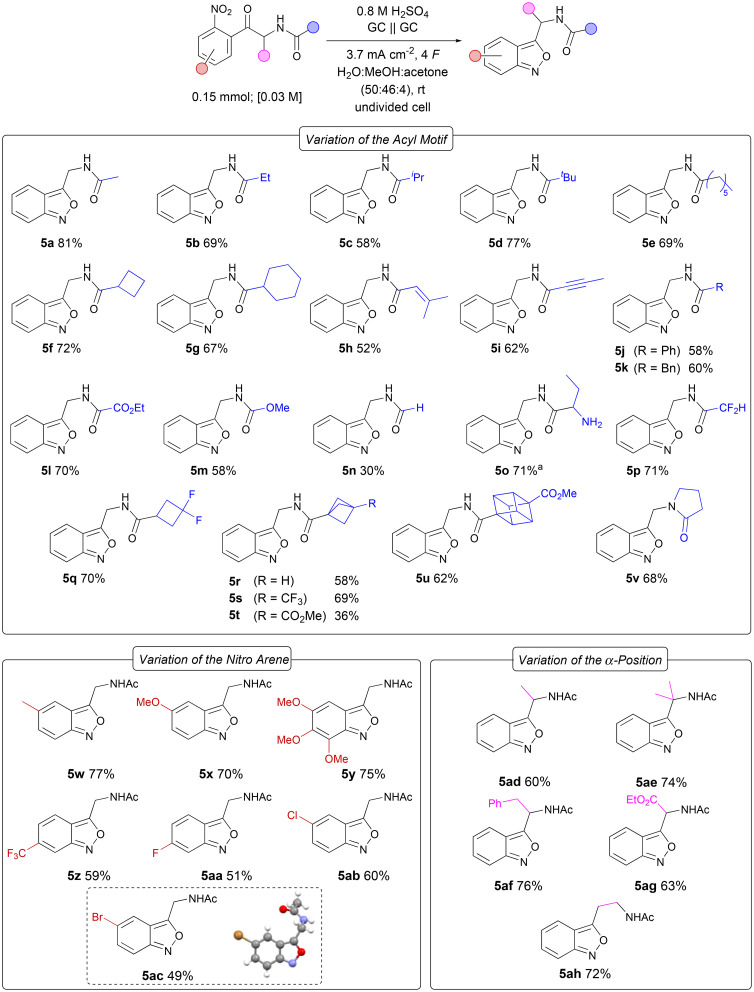
Scope of the electrochemical synthesis of 3-(acetamidomethyl)benzo[*c*]isoxazoles by cathodic reduction of nitro arenes; isolated yields. ^*a*^The corresponding *N*-Boc-protected substrate was used as the starting material.

The optimised reaction conditions were applied to a versatile range of substrates to explore the applicability of the developed protocol (Fig. 4). First, we investigated the influence of *N*-acyl moieties with different electronic and steric effects on the selective formation of the desired 2,1-benzisoxazoles. Primary, secondary and tertiary alkyl substituents were explored in substrates 5a–e, yielding the desired products in up to 81% yield. Interestingly, no decrease in yield was observed even with highly sterically demanding *tert*-butyl 5d or *n*-hexyl 5e substituents. Substrates with cyclobutyl and cyclohexyl moieties yielded the corresponding heterocycles 5f and 5g in 72% and 67% yields. Alkene- and alkyne substituents were also tolerated, although with a slightly lower yield of 52% and 62% for benzisoxazoles 5h and 5i. The alkyne group is amenable to further functionalisation by cycloaddition reactions, facilitating the synthesis of five-membered heterocycles such as isoxazoles, pyrazoles or triazoles.^46^ The phenyl- and benzyl-substituted heterocycles 5j and 5k were isolated in 58% and 60% yields. The ester derivative 5l was obtained in 70% yield. Furthermore, the carbamate 5m was isolated in 58% yield, demonstrating the compatibility with both electron-withdrawing and -donating moieties. The formyl amide 5n could be synthesised in 30% yield. When a Boc-protected unnatural amino acid was subjected to the reaction conditions, both the reductive cyclisation and deprotection of the Boc group occurred, resulting in 71% yield of the deprotected unnatural amino acid 5o. In medicinal chemistry, fluorine-containing moieties are often introduced to modify metabolic stability, basicity, lipophilicity and bioavailability.^47^ Particularly difluoromethyl and *gem*-difluorocyclobutyl substituents are commonly used as bioisosteres for hydroxy, thiol or amine groups and cyclohexyl rings.^48^ The corresponding benzisoxazoles 5p and 5q were isolated in good yields of 71% and 70%, respectively. Bicyclo[1.1.1]pentanes (BCPs) and cubanes are suitable bioisosteres for *para*-substituted benzenes due to their rigidity and ring strain which increases the *s* character and therefore also the C–H bond strength.^49^ The unsubstituted BCP derivative 5r was obtained in 58% yield and its trifluoromethyl-substituted analogue 5s in 69% yield. A drop in yield was observed for the corresponding ester derivative 5t which was isolated in 36% yield, possibly due to hydrolysis of the ester functionality during the acidic work-up. The cubane-substituted anthranil 5u could be isolated in 62% yield. Finally, the lactam-substituted benzisoxazole 5v could be obtained in 68% yield. Next, the influence of the electronic effects of various aryl substituents on the reduction of the nitro group was investigated. Electron-rich substrates gave the corresponding methyl-, methoxy- and trimethoxy-substituted benzisoxazoles 5w, 5x, and 5z in good yields of 77%, 70% and 75%, respectively. The electron-deficient trifluoromethyl-substituted 5z was obtained in 63% yield. Trifluoromethyl moieties are commonly used to modify physicochemical properties in drug design.^50^ Fluorinated arenes are useful substrates for nucleophilic aromatic substitutions and halo-substituted arenes in general are widely employed as starting materials in metal-catalysed coupling reactions for the synthesis of complex molecules in pharmaceutical and agricultural chemistry.^51^ However, they are often not tolerated in cathodic reactions due to electrochemical dehalogenation.^52^ The corresponding 2,1-benzisoxazoles 5aa–ac were isolated in moderate yields of up to 60% and no dehalogenation could be observed, enabling various post-functionalisations. Moreover, additional substituents were introduced at the α-position. The methyl- and dimethyl-substituted benzisoxazoles 5ad and 5ae were formed in 60% and 74% yields, respectively. The benzyl derivative 5af could be obtained in a good yield of 76%. The ester derivative 5ag was isolated in 63% yield and resembles a novel protected unnatural amino acid which might be a useful building block in medicinal chemistry towards new active pharmaceutical ingredients (APIs).^53^ Extending the alkyl chain at the α-position resulted in 5ah in 72% yield and resembles a substituted tryptamine derivative.

**Fig. 5:**
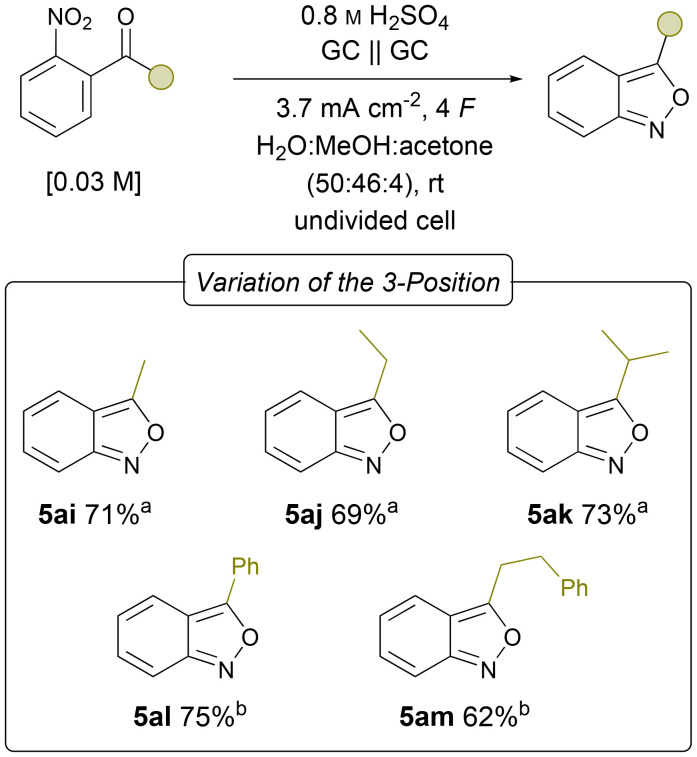
Scope of the electrochemical synthesis of 3-alkyl/-aryl substituted 2,1-benzisoxazoles; isolated yields; ^*a*^3.0 mmol scale, ^*b*^0.15 mmol scale.

Finally, the optimised conditions were applied to the synthesis of 3-alkyl substituted 2,1-benzixazoles (Fig. 5). Due to their low vapour pressure compounds 5ai–ak were synthesised on a 3 mmol scale. The simple methyl-substituted benzisoxazole 5ai could be obtained in 71% yield. Compared to the previously reported galvanostatic protocols, the yield could be further increased in this simple and more sustainable system. The ethyl and iso-propyl derivatives 5aj and 5ak could be synthesised in 69% and 73% yields, respectively. Phenyl substituents were tolerated as well, resulting in 75% yield of 5al and 62% of 5am on a 0.15 mmol scale. After exploring the scope of the reaction, a scale-up of the synthesis of 5a and 5ai was performed to demonstrate the scalability and synthetic utility of the developed protocol (Table 2).

**Table 2:** Scaling -up the electrochemical synthesis of 5a and 5ai

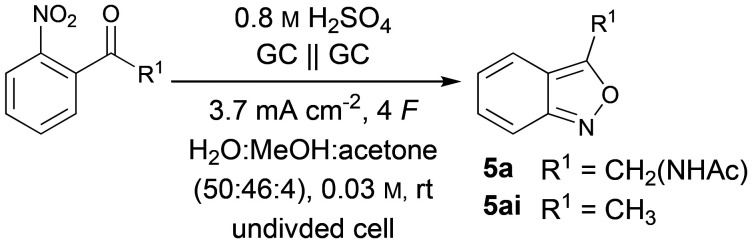			
Electrolysis cell	Scale/mmol	Yield of 5a	Yield of 5ai
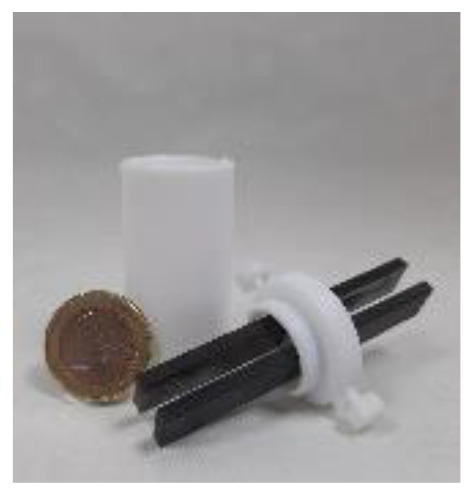	0.15	23.0 mg (81%)	—
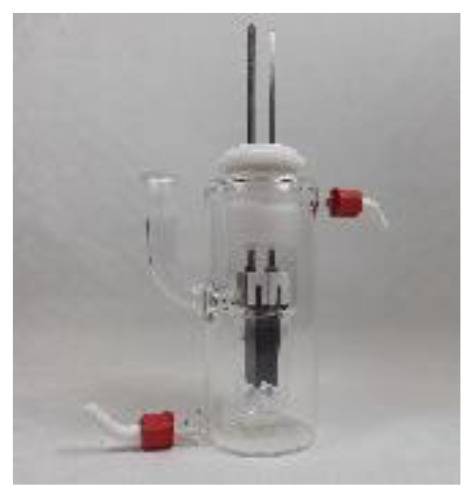	0.75	102 mg (71%)	—
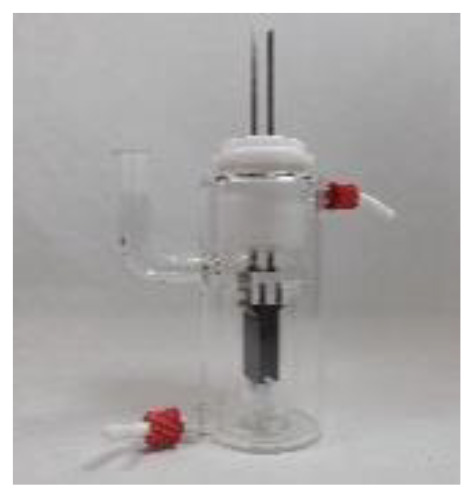	3.00	433 mg (77%)	284 mg (71%)
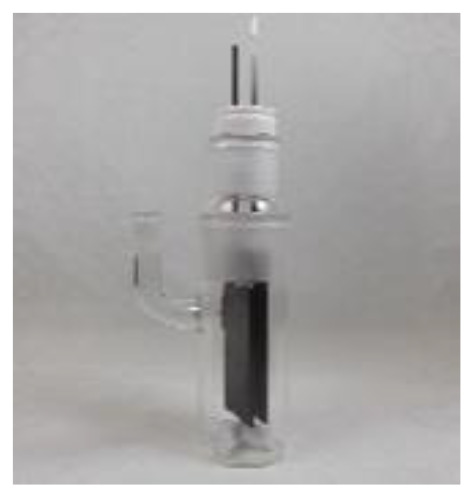	7.50	771 mg (54%)	675 mg (68%)

### Scale-up of the reductive cyclisation

On a 0.75 mmol scale, the desired benzisoxazole 5a was obtained in a good yield of 71% (0.102 g). Increasing the scale to 3.0 mmol resulted in a slightly better yield of 76% (0.433 g) of 5a and 71% (284 mg) of 5ai. After these promising results, the electrolysis was performed on a gram scale (7.5 mmol). However, 5a was obtained only in 54% (771 mg) yield possibly due to a deacetylation reaction of the product and subsequent degradation of the amine during electrolysis. The less substituted product 5ai was isolated without any significant loss in yield.

### Mechanistic studies

**Scheme 1:**
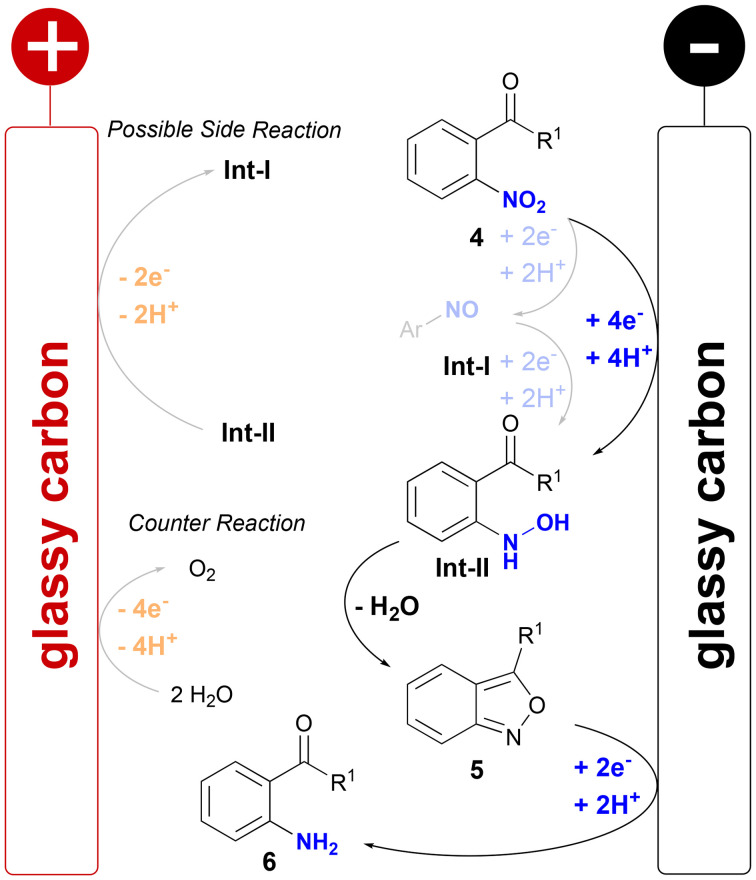
Proposed mechanism for the electrochemical formation of 2,1-benzisoxazoles 5 from 2-nitroacetophenones 4.

The mechanism of the electrochemical synthesis of 2,1-benzisoxazoles was investigated by cyclic voltammetry and isotopic labelling of 2′-nitroacetophenone (see the ESI† for details). CV studies showed a large reductive wave for 4a and 2′-nitroacetophenone (−0.56 V *vs.* FcH/FcH^+^) corresponding to the selective reduction of the nitro group to the corresponding hydroxyl amine Int-II. This reductive wave was not detected during the analysis of 5a and 5ai. Afterwards Int-II undergoes cyclo-condensation to form the desired products 5 (Scheme 1).

Additionally, a broad reductive wave (−1.09 V to −1.24 V *vs.* FcH/FcH^+^) was observed for the starting materials 4a and 2′-nitroacetophenone and the 2,1-benzisoxazoles 5a and 5ai. The cyclo-condensation of Int-II was investigated by the isotopic enrichment of the carbonyl oxygen of 2′-nitroacetophenone. After electrolysis no ^18^O was detected in the 2,1-benzisoxazole 5ai suggesting a nucleophilic attack of the hydroxylamine Int-II oxygen into the carbonyl function. This corresponds to the subsequent reduction of the 2,1-benzisoxazoles 5 to the corresponding 2′-aminoacetophenones 6, which were detected as a common side product by LC-MS analysis. Furthermore, two oxidative waves (+0.80 V to +1.16 V *vs.* FcH/FcH^+^) were observed during the analysis of 4 and 5 most likely corresponding to an oxidation of the 2′-aminoacetophenone 6 side products.

## Conclusions

In summary, the established electrochemical protocol provides facile, direct and sustainable access to 3-(acetamidoalkyl)-2,1-benzisoxazoles by cathodic reduction of inexpensive nitro arenes and subsequent cyclocondensation, only using sulphuric acid as the supporting electrolyte and acidic catalyst. The reaction was performed in an undivided cell under constant current conditions, underlining the simplicity of the method. Sustainability was ensured using carbon-based electrodes, a water/methanol mixture as environmentally benign solvent and electricity as a surrogate for conventional reagents. Moreover, the broad applicability of the developed conditions was demonstrated by the synthesis of 39 examples in up to 81% isolated yield. Particularly at the 3-position a wide variety of acylamidomethyl substituents could be introduced, in addition to various functional groups at the aryl ring including electron-donating and -withdrawing moieties as well as redox labile halides. The scale-up of the synthesis of 2,1-benzisoxazoles demonstrated the easy scalability of the developed method and its relevance for technical applications. Mechanistic studies were performed to investigate the underlining mechanism of this electrochemical synthesis. Overall, the established electrochemical methodology offers an attractive pathway to access 2,1-benzisoxazoles as important building blocks in medicinal chemistry and organic synthesis.

## Author contributions

M. S. L., J. W. and A. S. conceived the project together with S. R. W., M. S. L., J. W., A. S. and M. G. conducted the experiments and interpreted and analysed the results. D. S. performed the X-ray analysis. M. S. L., J. W., A. S. and S. R. W. wrote and reviewed the manuscript. S. R. W. supervised the project. All authors discussed the results and agreed to the manuscript.

## Data availability

The ESI† is available online and contains experimental and analytical data (NMR, CV studies and HRMS).

## Conflicts of interest

There are no conflicts to declare.

## Supplementary Material

OB-023-D4OB01875C-s001

OB-023-D4OB01875C-s002

## References

[cit1] Taylor R. D., MacCoss M., Lawson A. D. G. (2014). J. Med. Chem..

[cit2] Zhu X.-Q., He J.-J., Zhou B., Ye L.-W. (2023). Cell Rep. Phys. Sci..

[cit3] Zhu J., Mo J., Lin H.-Z., Chen Y., Sun H.-P. (2018). Bioorg. Med. Chem..

[cit4] Stebelska K. (2013). Ther. Drug Monit..

[cit5] Shetnev A., Kotov A., Kunichkina A., Proskurina I., Baykov S., Korsakov M., Petzer A., Petzer J. P. (2024). Mol. Divers..

[cit6] Pierce A. C., Jacobs M., Stuver-Moody C. (2008). J. Med. Chem..

[cit7] Rezazadeh M., Pordel M., Davoodnia A., Saberi S. (2015). Chem. Heterocycl. Compd..

[cit8] Chaker A., Najahi E., Chatriant O., Valentin A., Téné N., Treilhou M., Chabchoub F., Nepveu F. (2017). Arabian J. Chem..

[cit9] Moriwaki T. , Fürstner C., Riedl B., Ergüden J.-K., Böss F., Schmidt B., van der Staay F.-J., Schröder W., Schlemmer K.-H. and Yoshida N., US6589949B1, 2003

[cit10] Kardile R. D., Chao T.-H., Cheng M.-J., Liu R.-S. (2020). Angew. Chem., Int. Ed..

[cit11] Garia A., Grover J., Jain N. (2021). Eur. J. Org. Chem..

[cit12] Chattopadhyaya J. and Upadhayaya R. S., WO2009091324A1, 2009

[cit13] Lyssikatos J. P. , La Greca S. D. and Bingwei V. Y., WO2000012498A1, 2000

[cit14] Aidene M., Belkessam F., Soulé J.-F., Doucet H. (2016). ChemCatChem.

[cit15] Gao Y., Nie J., Huo Y., Hu X.-Q. (2020). Org. Chem. Front..

[cit16] Song Y., Xue R., Wang L., Li N., Fang Z., Fu Y., Chen D.-L., Zhu W., Zhang F. (2023). Green Chem..

[cit17] Jin H., Tian B., Song X., Xie J., Rudolph M., Rominger F., Hashmi A. S. K. (2016). Angew. Chem., Int. Ed..

[cit18] Wang Z.-H., Zhang H.-H., Wang D.-M., Xu P.-F., Luo Y.-C. (2017). Chem. Commun..

[cit19] Tiwari D. K., Phanindrudu M., Wakade S. B., Nanubolu J. B., Tiwari D. K. (2017). Chem. Commun..

[cit20] Bellamy F. D., Ou K. (1984). Tetrahedron Lett..

[cit21] Nord F. F. (1919). Ber. dtsch. Chem. Ges. B.

[cit22] Luján A. P., Bhat M. F., Marko E. E. A., Fodran P., Poelarends G. J. (2024). Chem. – Eur. J..

[cit23] Arcadi A., Chiarini M., Del Vecchio L., Marinelli F., Michelet V. (2016). Chem. Commun..

[cit24] Dickson N. J., Dyall L. K. (1980). Aust. J. Chem..

[cit25] Kotov A. D., Prokaznikov M. A., Antonova E. A., Rusakov A. I. (2014). Chem. Heterocycl. Compd..

[cit26] Wróbel Z. (1997). Synthesis.

[cit27] Waldvogel S. R., Janza B. (2014). Angew. Chem., Int. Ed..

[cit28] Francke R. (2022). Curr. Opin. Electrochem..

[cit29] Beil S. B., Pollok D., Waldvogel S. R. (2021). Angew. Chem., Int. Ed..

[cit30] Gütz C., Klöckner B., Waldvogel S. R. (2016). Org. Process Res. Dev..

[cit31] Möhle S., Zirbes M., Rodrigo E., Gieshoff T., Wiebe A., Waldvogel S. R. (2018). Angew. Chem., Int. Ed..

[cit32] Wiebe A., Gieshoff T., Möhle S., Rodrigo E., Zirbes M., Waldvogel S. R. (2018). Angew. Chem., Int. Ed..

[cit33] Yan M., Kawamata Y., Baran P. S. (2017). Chem. Rev..

[cit34] Rosen B. R., Werner E. W., O'Brien A. G., Baran P. S. (2014). J. Am. Chem. Soc..

[cit35] Winter J., Prenzel T., Wirtanen T., Gálvez-Vázquez M. d. J., Hofman K., Schollmeyer D., Waldvogel S. R. (2024). Cell Rep. Phys. Sci..

[cit36] Koleda O., Prenzel T., Winter J., Hirohata T., Gálvez-Vázquez M. d. J., Schollmeyer D., Inagi S., Suna E., Waldvogel S. R. (2023). Chem. Sci..

[cit37] Rodrigo E., Waldvogel S. R. (2019). Chem. Sci..

[cit38] Winter J., Prenzel T., Wirtanen T., Schollmeyer D., Waldvogel S. R. (2023). Chem. – Eur. J..

[cit39] (a) Lund H. , in Advances in Heterocyclic Chemistry V12, ed. A. R. Katritzky, Elsevier textbooks, s.l., 1st edn, 1970, vol. 12, pp. 213–316

[cit40] Kim B. H., Jun Y. M., Choi Y. R., Lee D. B., Baik W. (1998). Heterocycles.

[cit41] Hosseini S., Bawel S. A., Mubarak M. S., Peters D. G. (2019). ChemElectroChem.

[cit42] Wirtanen T., Prenzel T., Tessonnier J.-P., Waldvogel S. R. (2021). Chem. Rev..

[cit43] Rodrigo E., Baunis H., Suna E., Waldvogel S. R. (2019). Chem. Commun..

[cit44] Beil S. B., Müller T., Sillart S. B., Franzmann P., Bomm A., Holtkamp M., Karst U., Schade W., Waldvogel S. R. (2018). Angew. Chem., Int. Ed..

[cit45] Lips S., Waldvogel S. R. (2019). ChemElectroChem.

[cit46] Ramani A., Desai B., Patel M., Naveen T. (2022). Asian J. Org. Chem..

[cit47] Böhm H.-J., Banner D., Bendels S., Kansy M., Kuhn B., Müller K., Obst-Sander U., Stahl M. (2004). ChemBioChem.

[cit48] Zafrani Y., Yeffet D., Sod-Moriah G., Berliner A., Amir D., Marciano D., Gershonov E., Saphier S. (2017). J. Med. Chem..

[cit49] Locke G. M., Bernhard S. S. R., Senge M. O. (2019). Chem. – Eur. J..

[cit50] Abula A., Xu Z., Zhu Z., Peng C., Chen Z., Zhu W., Aisa H. A. (2020). J. Chem. Inf. Model..

[cit51] Amii H., Uneyama K. (2009). Chem. Rev..

[cit52] Gütz C., Bänziger M., Bucher C., Galvão T. R., Waldvogel S. R. (2015). Org. Process Res. Dev..

[cit53] Suhas R., Chandrashekar S., Gowda D. C. (2011). Eur. J. Med. Chem..

