# Supplementary material for: Simple and versatile electrochemical synthesis of highly substituted 2,1-benzisoxazoles
Source: Org Biomol Chem. 2024 Dec 3;23(10):2391–9. doi: 10.1039/d4ob01875c (PMC11632592; doi:10.1039/d4ob01875c)
Supplement: OB-023-D4OB01875C-s001 [file OB-023-D4OB01875C-s001.pdf]

Supplementary Information (SI) for Organic & Biomolecular Chemistry.
This journal is © The Royal Society of Chemistry 2024

Electronic Supporting Information (ESI)

Simple and Versatile Electrochemical Synthesis of Highly Substituted 2,1-Benzisoxazoles

Marola S. Lenhard, Johannes Winter, Alexander Sandvoß, María de Jesús Gálvez-Vázquez, Dieter Schollmeyer and Siegfried R. Waldvogel

1. General Information S2
2. Mechanistic Studies S6
2.1. CV measurements S6
2.2. ^{18}O-Labeling Experiment S12
3. NMR Quantification S13
4. Optimisation of the Electrolytic Conditions S14
5. General Protocols S16
5.1. General Protocol for the Synthesis of Substituted 1-(2-Nitrophenyl)alkan-1-one *O*-tosyl oximes (GP 1) S16
5.2. General protocol for the synthesis of 4-ethoxycarbonyl-5-(2-nitrophenyl)oxazoles (GP 2) S16
5.3. General protocol for the synthesis of 2-nitro-1-(2-nitrophenyl)alkan-1-ones (GP 3) S17
5.4. General Protocol for the Synthesis of Substituted 1-(2-Nitrophenyl)alkan-1-one *O*-tosyl oximes (GP 4) S17
5.5. General protocol for the synthesis of *N*-(2-nitrophenacyl)acylamides using acid chlorides or anhydrides (GP 5) S17
5.6. General protocol for the synthesis of *N*-(2-Nitrophenacyl)acylamides using carboxylic acids (GP 6) S18
5.7. General Protocol for the Electrochemical Synthesis of substituted 2,1-benzo[*c*]isoxazoles (GP 7) 18
6. Scale-up of the Electrochemical Reductive Synthesis of 3-(*N*-Acetamidomethyl)-benzo[*c*]isoxazole (5a) and 3-Methylbenzo[*c*]isoxazole (5ai) S20
7. Preparation of Products and Analytical Data S21
7.1. 1-(2-nitrophenyl)alkan-1-ones (11-14) S22
7.2. 1-(2-nitrophenyl)alkan-1-one *O*-tosyl oximes (15-19) S24
7.3. 4-Ethoxycarbonyl-5-(2-nitrophenyl)oxazoles (20–25) S27
7.4. 2-Amino-1-(2-nitrophenyl)alkan-1-one hydrochlorides (26-37) S29
7.5. *N*-(2-Nitrophenacyl)acylamides (4a–4ah, 6a, 6a’) S32
7.6. 3-(*N*-Acylaminoalkyl)-benzo[*c*]isoxazoles S44
8. Crystallographic Data S57
9. NMR Spectra S59
10. Supplemental References 164

1. General Information

If not stated otherwise, all reactions were performed under ambient conditions and chemicals in analytical grade were used as purchased without further purification. Milli-Q® water was obtained using Simplicity® System (UV) (*Merck KGaA*, Darmstadt, Germany) for chromatography purposes. Anhydrous solvents were prepared by a solvent purification system SPS-5 (*M. Braun Incorporated*, Stratham, USA).

Chromatography
Thin layer chromatography was performed using DC silica gel 60 F254 on aluminum plates (*Merck KGaA,* Darmstadt, Germany). A UV lamp (λ = 254 nm, NU-4 KL, *Benda*, Wiesloch, Germany) was used for detection.
Preparative column chromatography was accomplished using different prepacked puriFlash® column *(Interchim SAS*, Montluçon Cedex, France) using a puriFlash® XS 520 Plus system (Interchim *SAS*, Montluçon Cedex, France).
Reversed phase column chromatography of the different products was performed on a Puriflash® PF-30C18HP-F0080 (*Interchim SAS*, Montluçon Cedex, France) column using a Sepacore® system with a Büchi Control Unit C-620, Büchi Pump Modules C-605, a UV detector Büchi UV photometer C-635, and Büchi Fraction Collector C-660 (*Büchi-Labortechnik GmbH*, Essen, Germany) using different mixtures of water (0.1% formic acid (*v/v*)) and acetonitrile as eluents.

High Resolution Mass Spectrometry
Mass spectra *via* electrospray-ionization (ESI+/ESI– and APCI+/APCI-) mass spectrometry were recorded using an Agilent 6545 QTOF-MS (*Agilent*, Santa Clara (CA), USA). Mass-charge ratios (*m*/*z*) were obtained for the characterized compounds.

X-ray Crystallography
The measurements of the crystal structures were carried out on a STOE IPDS-2T (*STOE & Cie GmbH*, Darmstadt, Germany) using a Mo source with graphite tube monochromator.

High Performance Liquid Chromatography (HPLC)
Analysis of crude reaction mixtures, purified products and method development was performed using a modular system LC-20A *Prominence* (*Shimadzu Deutschland GmbH*, Duisburg, Germany), UV/VIS-detector SPD-20A/AV (*Shimadzu Deutschland GmbH*, Duisburg, Germany), and LCMS-2020 Single Quadrupole (*Shimadzu Deutschland GmbH*, Duisburg, Germany). Analytical separation was performed using an Eurospher II 100-5 C-18-Trennsäule (*Knauer Wissenschaftliche Geräte GmbH*, Berlin, Germany) column (length of 150 mm, diameter of 4 mm, pore size of 100 Å, particle size 5 µm). As eluents, acetonitrile, and water with 5% (*v*/*v*) acetonitrile and formic acid (0.1% (*v*/*v*)) were used. Given retention times were obtained at λ = 254 nm.

Nuclear Magnetic Resonance (NMR) Spectroscopy
Nuclear magnetic resonance experiments were recorded using a nuclear magnetic resonance spectrometer Avance III HD300 (*Bruker*, Karlsruhe, Germany) ^{1}H NMR (300 MHz), ^{19}F NMR (282 MHz), Avance II 400 (*Bruker*, Karlsruhe, Germany) ^{1}H NMR (400 MHz), ^{13}C NMR (101 MHz), ^{19}F NMR (376 MHz) and Avance III 600 (*Bruker*, Karlsruhe, Germany) ^{1}H NMR (600 MHz), ^{13}C NMR (151 MHz). The spectra were recorded using deuterated solvents. To normalize the spectra obtained, reference was made to the existing solvent signal of non-deuterated fractions according to the data provided by *Fulmer et al.*:[1] $CDCl_3$ (^{1}H NMR: δ = 7.26 ppm, ^{13}C NMR: δ = 77.2 ppm), dichloromethane-d_2 (^{1}H NMR: δ = 5.32 ppm, ^{13}C NMR: δ =53.8 ppm), acetonitrile-d_3 (^{1}H NMR δ = 1.94 ppm, ^{13}C NMR: δ = 118.3 ppm), CD_3OD (^{1}H NMR δ = 3.31 ppm, ^{13}C NMR: δ =49.0 ppm) and DMSO-[D6] (^{1}H NMR δ = 2.50 ppm, ^{13}C NMR: δ =39.5 ppm). Besides ^{1}H, ^{13}C and ^{19}F NMR experiments, the 2D techniques ^{1}H,^{1}H COSY, ^{1}H,^{13}C HSQC and ^{1}H,^{13}C HMBC were used assisting to assign the signals. The following abbreviations were used to describe the signals: s (singlet), d (doublet), t (triplet), dd (doublet of doublets), td (triplet of doublets), m (multiplet), q (quartet), sep (septet), ddd (doublet of doublets of doublets). The spectra obtained were evaluated with MestReNova 14.2.0-26256 (*Mestrelab Research S.L.*, Spain).

Cyclic Voltammetry (CV) Measurements

The mechanism of the reaction was studied by cyclic voltammetry using an electrochemical glass cell (figure S1) equipped with a glassy carbon tip electrode (with a diameter of 4 mm), a glassy carbon disc (working electrode, diameter: 2 mm), glassy carbon rod (counter electrode) and a Ag/AgCl (saturated LiCl in ethanol, *Metrohm AG*, Herisau, Switzerland) as reference electrode, respectively. The electrode potentials are reported with reference to the redox system ferrocene/ferrocenium (FcH/FcH^{+}).

Cyclic voltammograms were measured using a potentiostat/galvanostat PGSTAT302N (*Metrohm AG*, Herisau, Switzerland) with a scan rate of 50 mV·s^{-1} in a methanol (HPLC LC-MS grade, *VWR International GmbH*, Darmstadt, Germany), acetone (analytical grade, *VWR International GmbH*, Darmstadt, Germany) and water solution (48:2:50 *v*/*v*) containing 0.8 M of H_2SO_4 (analytical reagent grade, *Fisher Scientific GmbH*, Schwerte, Germany) and 5 mM of the corresponding molecule. Prior to the CV measurements, the electrolyte was degassed with an argon flow for 25 min. An argon flow was kept flowing over the electrolyte during the measurements.

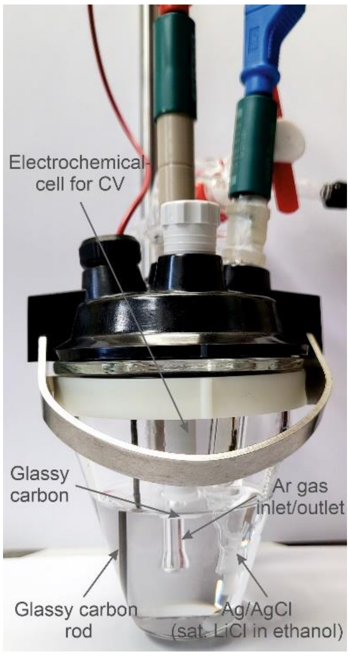

Figure S1: Electrochemical cell for cyclic voltammetry measurements.

Electrode Materials

Electrode material, purity, and their supplier are listed in table S1. Boron-doped diamond (BDD) electrodes were treated prior to electrosynthesis in 20% aqueous sulfuric acid (100 C) with current density of 10 mA·cm^{-2} by polarizing subsequently anodically and then cathodically. After the treatment, the cathode was rinsed with water, methanol, and dried.

Table S1: Electrode materials, purity, and their supplier.

Entry	Electrode Material	Purity	Supplier
1	Boron-doped diamond (DIACHEM™)	15 µm boron-doped diamond layer on silicon	*CONDIAS GmbH*, Itzehoe, Germany
2	Glassy carbon (Sigradur G)	-	*HTW*, Thierhaupten, Germany

Electrochemical Set-Up

Electrochemical reactions were carried out using a multichannel galvanostat HMP4040 (*Rohde & Schwarz*, München, Germany). The different cells used for screening or batch reactions are described below.

Screening Reactions

Teflon™ cells with a volume of 5 mL were used for the undivided set-up (figure S2, left). Divided Teflon™ screening cells with a volume of 7 mL were equipped with a glass frit as separator material as shown (figure S2, right). Glass frits used as separator materials were pre-treated in the corresponding electrolyte prior to use. Stirring bars were used during electrolysis in each cell. The described screening systems are commercially available as IKA Screening System Package (*IKA™ Werke GmbH & Co. KG*, Staufen, Germany).[1] Electrochemical reactions under argon atmosphere were performed with available parts of the IKA screening systems (*IKA Werke GmbH & Co. KG*, Staufen, Germany) were used equipped with rubber sealed lids developed in the mechanical workshop of the Johannes Gutenberg University Mainz as reported before with glassy carbon electrodes (0.8 cm·7 cm·0.2cm).[2]

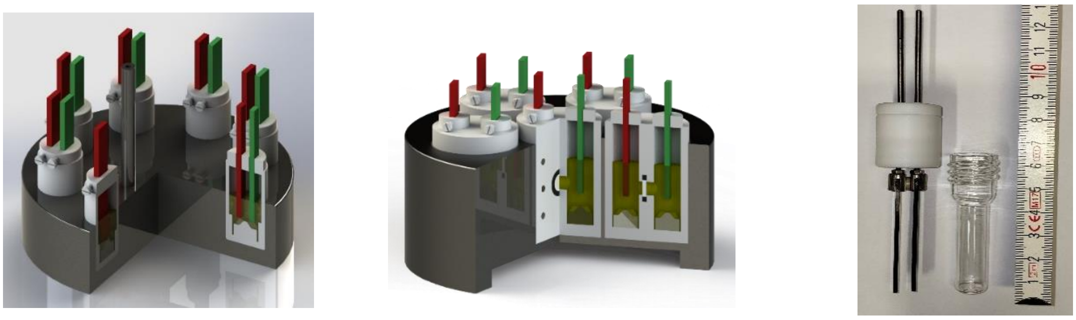

Figure S2: Undivided screening set-up (left) and divided screening set-up (right).[1,2]

Scale-up Reactions

Scale-up experiments were performed in undivided 25 mL, 100 mL and 250 mL batch-type cells with a PTFE stopper and sleeve, electrodes, and electrode holders (figure S2). A TDK-Lambda Z+ series (*TDK-Lambda UK Limited*, Devon, United Kingdom) or a multichannel power supply HMP4040 (*Rohde & Schwarz*, München, Germany) were used as power sources. In the undivided 5 mL electrolysis set-up glassy carbon electrodes with dimensions of 7 cm·1 cm·0.3 cm were used. In the undivided 25 mL and 100 mL electrolysis set-up glassy carbon electrodes with identical dimensions of 6 cm·2 cm·0.3 cm were used. In the undivided 250 mL electrolysis set-up glassy carbon electrodes with identical dimensions of 12 cm·3.7 cm·0.3 cm were used. The batch-type cells of the scale-up experiments are commercially available as SynLectro™ Starter Kit (*Merck KGaA*, Darmstadt, Germany).[3]

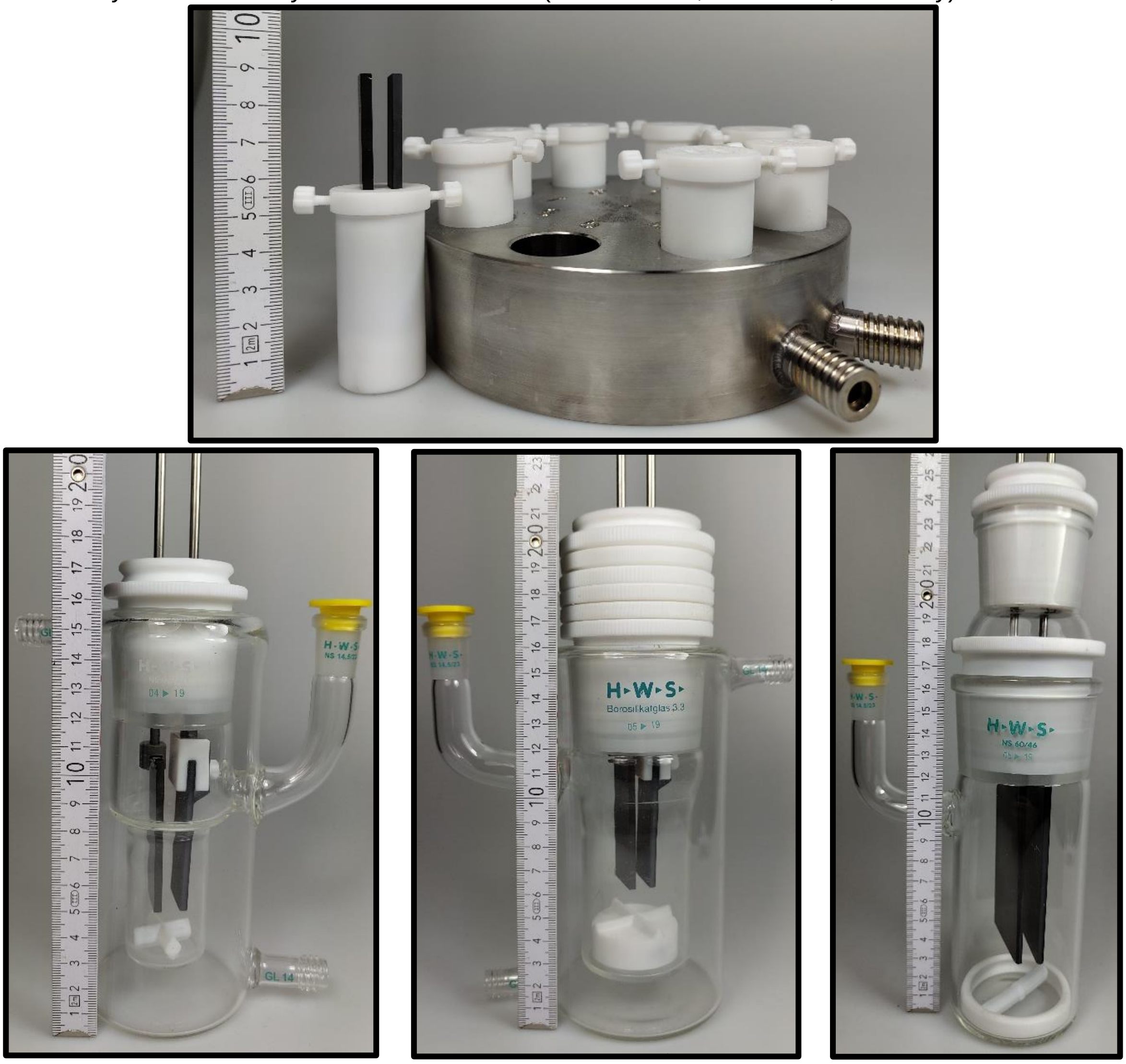

Figure S3: Different batch-type cells; size compared to a ruler: top: 5 mL undivided Teflon™ screening cells with glassy carbon and BDD electrodes and screening cell holder with integrated radiator loop; bottom left: 25 mL undivided glass cell with glassy carbon and BDD electrodes; bottom centre: 100 mL undivided glass cell with glassy carbon and BDD electrodes; bottom right: 300 mL divided glass cell with glassy carbon and BDD electrodes.[3]

2. Mechanistic Studies

2.1. CV measurements

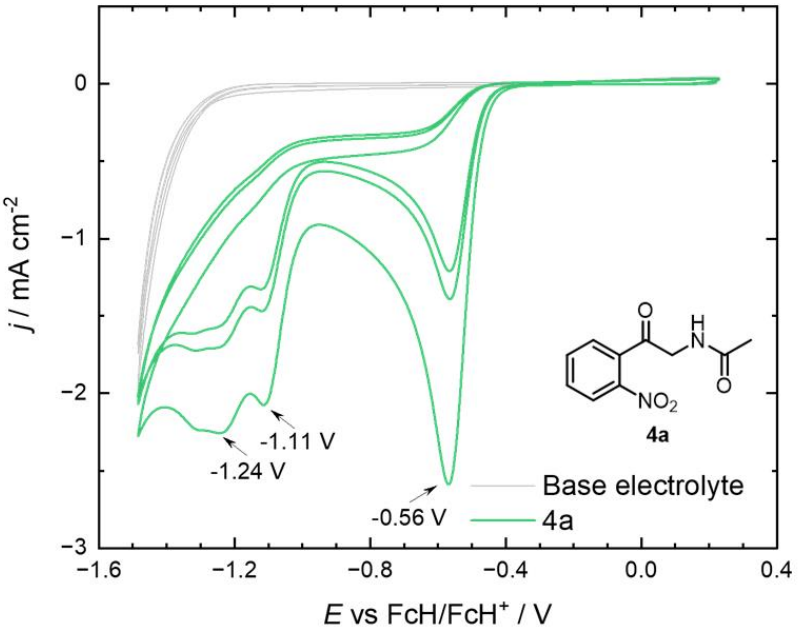

Figure S4: Cyclic voltammogram of **4a** with 0.8 M H_2SO_4 as additive; only the cathodic potential range.

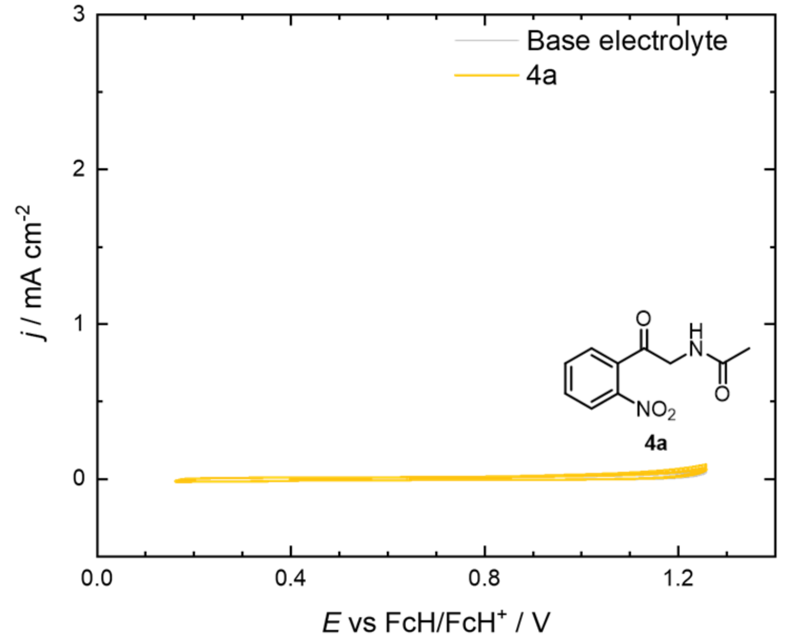

Figure S5: Cyclic voltammogram of **4a** with 0.8 M H_2SO_4 as additive; only the anodic potential range.

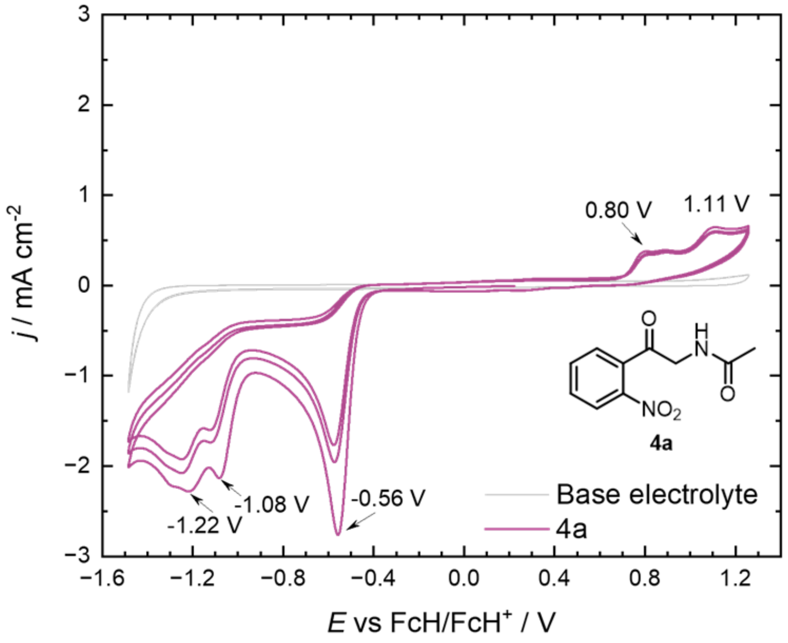

Figure S6: Cyclic voltammogram of **4a** with 0.8 M H_2SO_4 as additive; the full potential range.

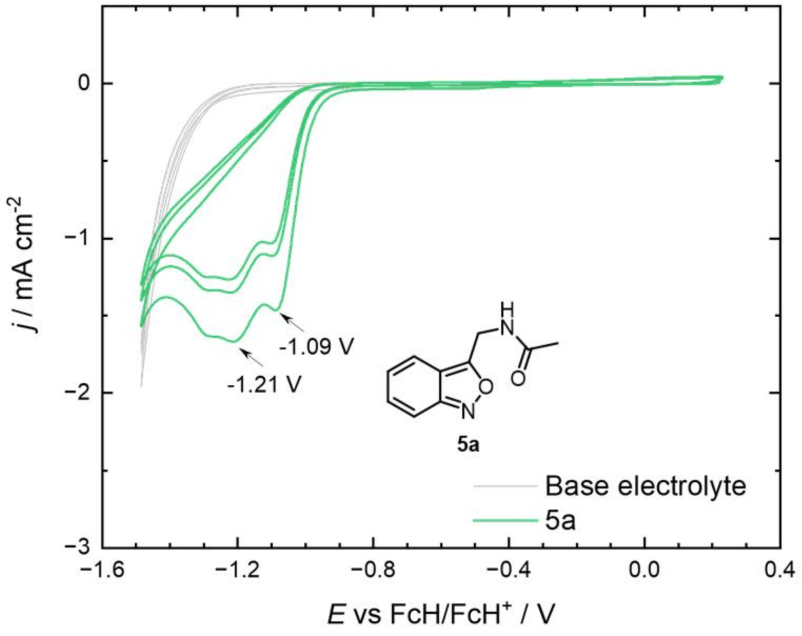

Figure S7: Cyclic voltammogram of **5a** with 0.8 M H_2SO_4 as additive; only the cathodic potential range.

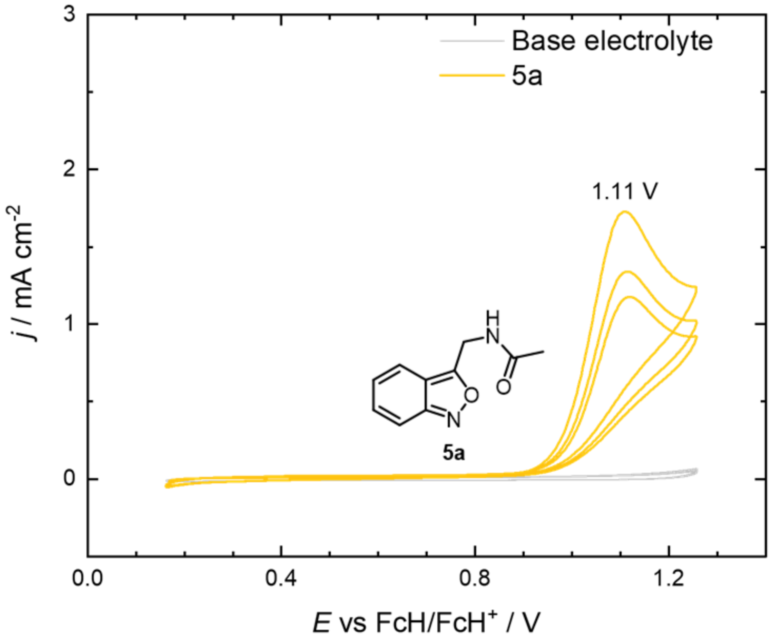

Figure S8: Cyclic voltammogram of **5a** with 0.8 M H_2SO_4 as additive; only the anodic potential range.

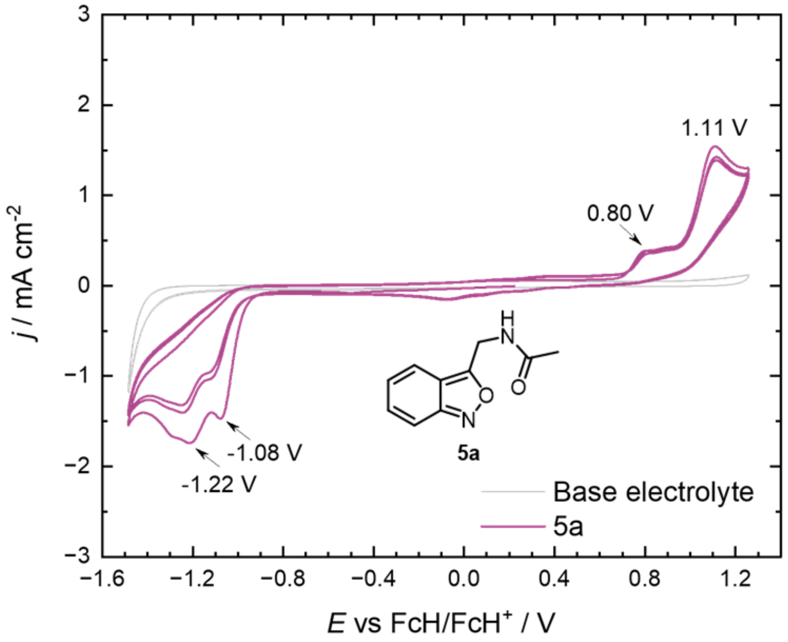

Figure S9: Cyclic voltammogram of **5a** with 0.8 M H_2SO_4 as additive; the full potential range.

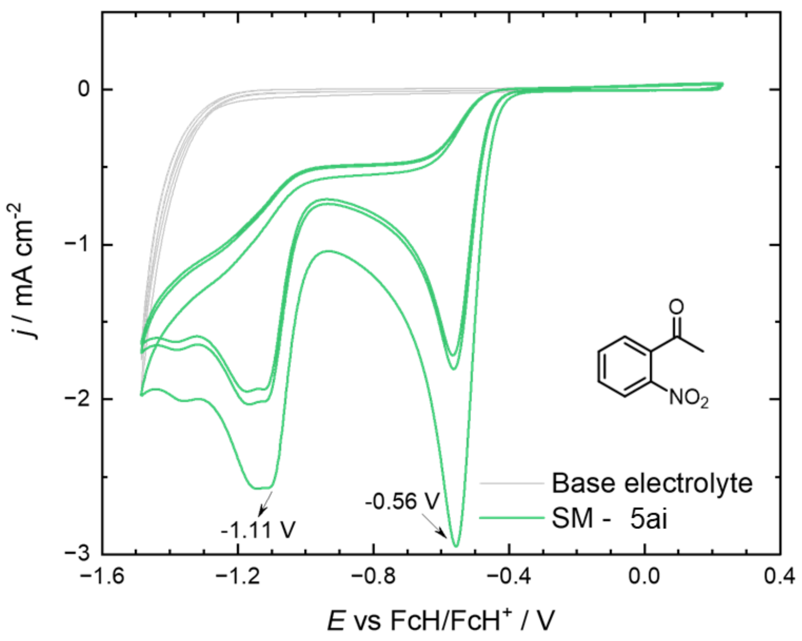

Figure S10: Cyclic voltammogram of 2-nitro-acetophenone with 0.8 M H_2SO_4 as additive; only the cathodic potential range.

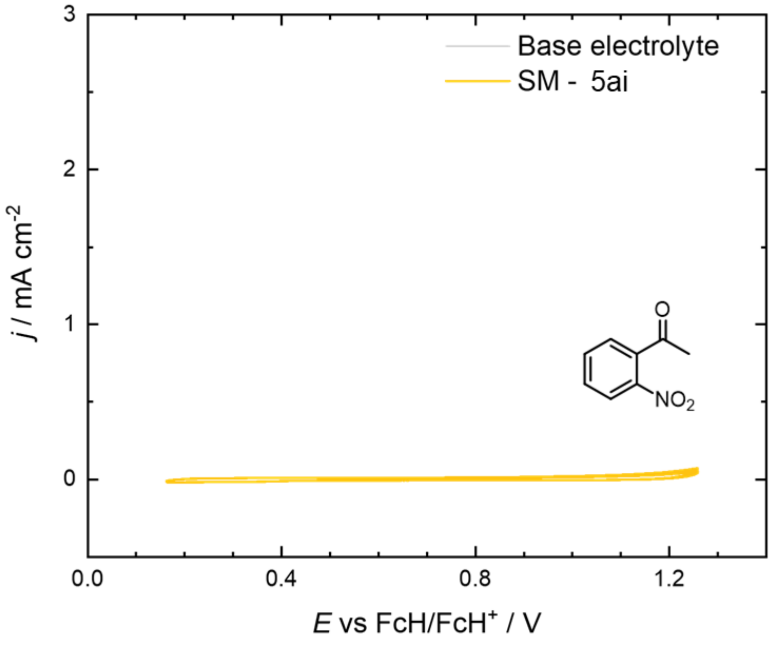

Figure S11: Cyclic voltammogram of 2-nitro-acetophenone with 0.8 M H_2SO_4 as additive; only the anodic potential range.

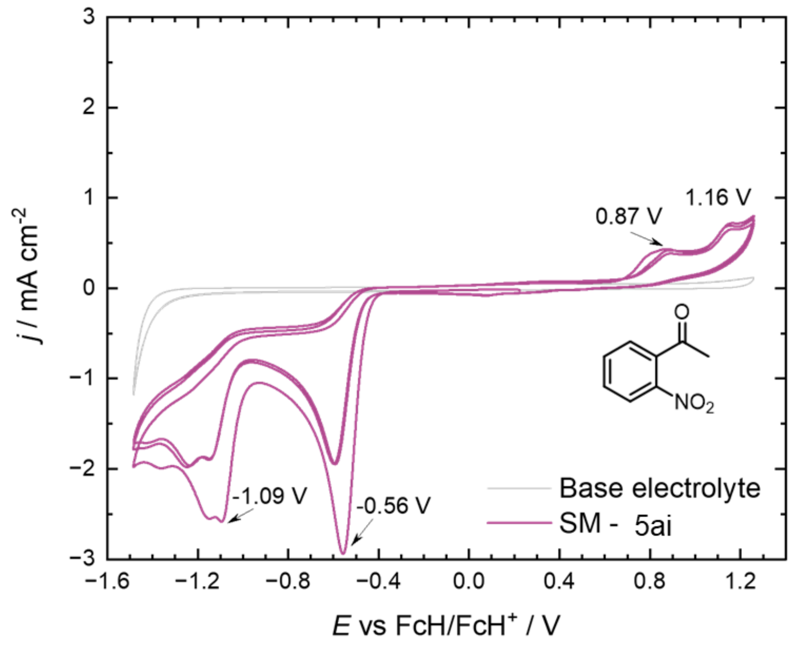

Figure S12: Cyclic voltammogram of 2-nitro-acetophenone with 0.8 M H_2SO_4 as additive; the full potential range.

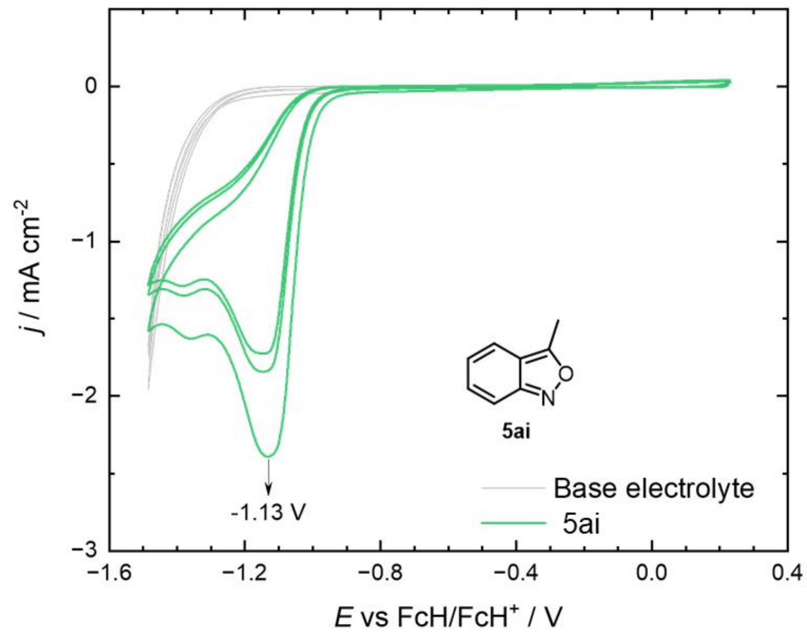

Figure S13: Cyclic voltammogram of **5ai** with 0.8 M H_2SO_4 as additive; only the cathodic potential range.

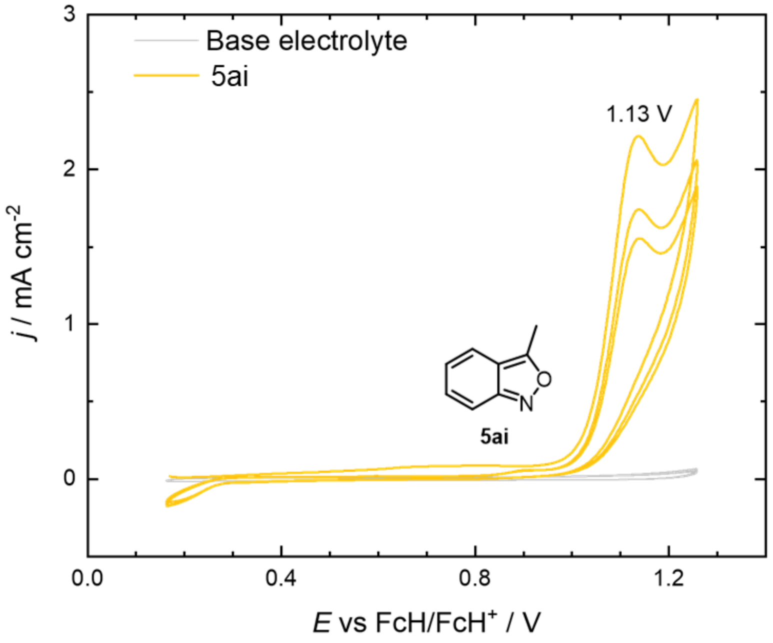

Figure S14: Cyclic voltammogram of **5ai** with 0.8 M H_2SO_4 as additive; only the anodic potential range.

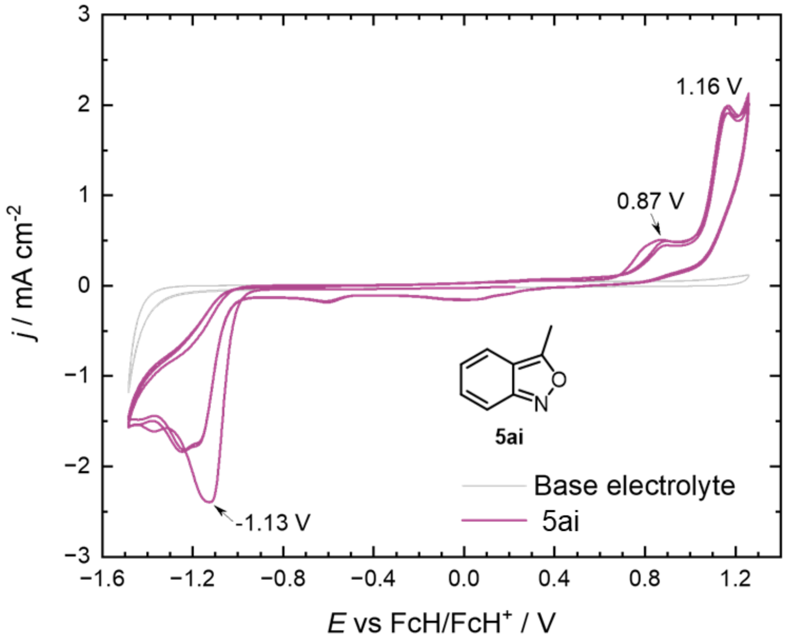

Figure S15: Cyclic voltammogram of **5ai** with 0.8 M H_2SO_4 as additive; the full potential range.

2.2. ^{18}O-Labeling Experiment

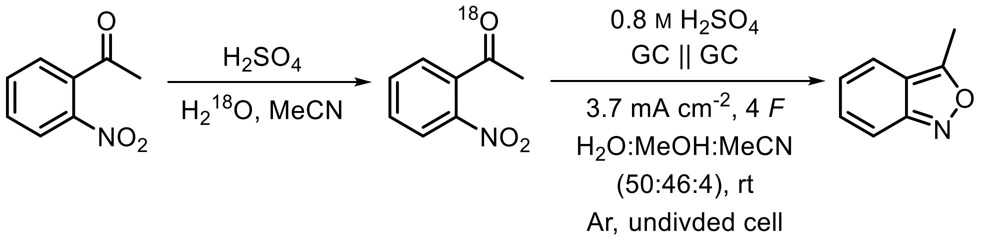

According to the literature[4], under argon atmosphere 2'-nitroacetophenone (24.8 mg, 0.15 mmol) was dissolved in 0.2 mL of anhydrous acetonitrile, 2.5 mL of $H_2{}^{18}O$ (97%, *Deutero GmbH*, Kastellaun, Germany) and 200 µL of concentrated sulphuric acid (analytical reagent grade, *Fisher Scientific GmbH*, Schwerte, Germany) were added to a flame dried electrolysis cell (figure S2). The resulting mixture was stirred for 24 h at room temperature. The isotopic enrichment of the ketone was determined by MS analysis (95% ^{18}O).

Afterwards, 2.5 mL of anhydrous methanol was added. The electrolysis was performed under constant current conditions (current density j = 3.7 mA·cm^{-2} for 57.8 C). Acetonitrile was used instead of acetone to suppress a decreased isotopic enrichment. After the reaction, 100 µL were diluted with 0.5 mL of acetonitrile and the isotopic composition of the crude reaction mixture was determined by MS analysis. No incorporation of the ^{18}O labelled oxygen atom into the 3-methylbenzo[*c*]isoxazole (**5ah**) was detected by MS analysis.

3. NMR Quantification

After work-up of the crude reaction mixture according to general protocol **7** (**GP7**) 0.1 mmol (16.8 mg) of 1,3,5-trimethoxybenzene was added. The mixture was completely dissolved in 1 mL of DMSO-[D6] and analysed by NMR. The signals were assigned according to figure S8.

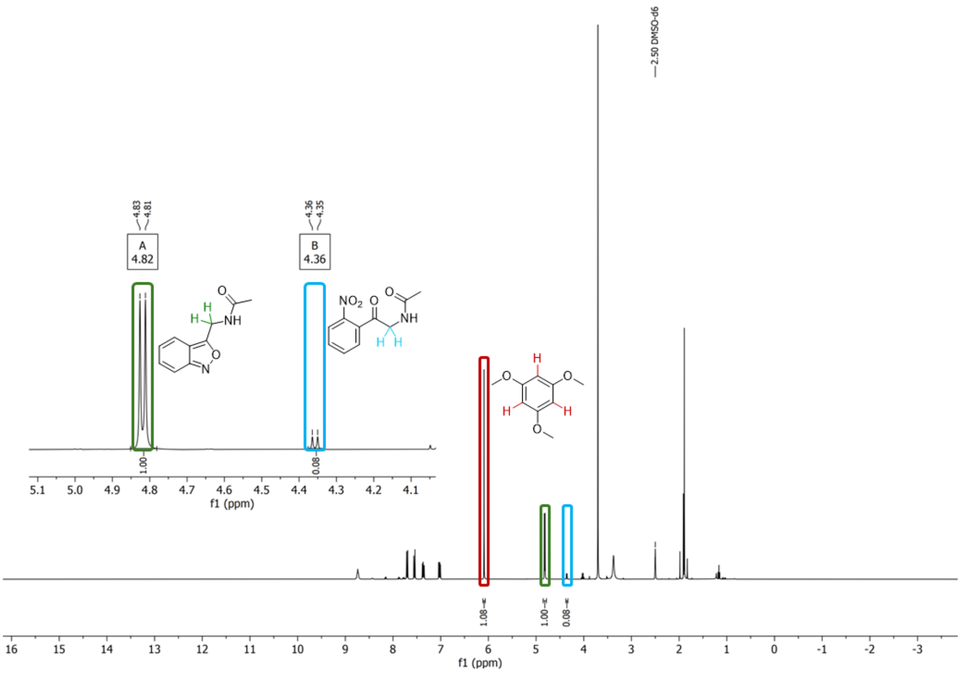

Figure S16: Assignment of the ^{1}H NMR signals for NMR quantification of the optimisation of the electrolytic conditions.

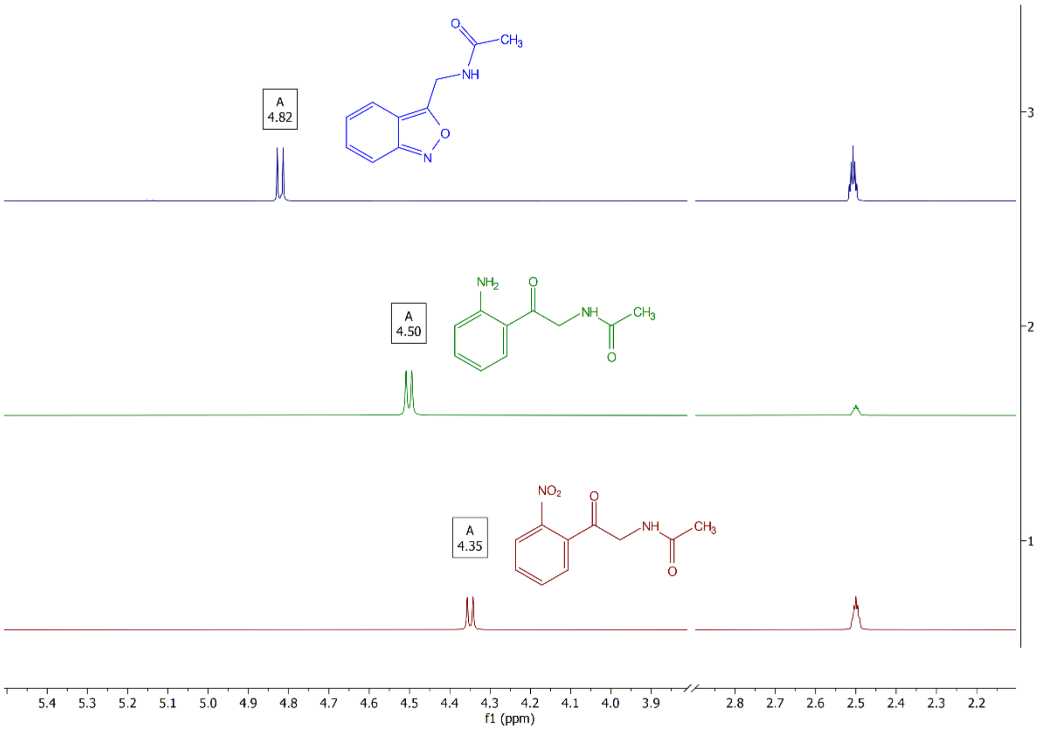

Figure S17: Assignment of the ^{1}H NMR signals for NMR quantification of the optimisation of electrolytic conditions.

4. Optimisation of the Electrolytic Conditions

Table S2: Optimisation of the electrolytic reaction conditions for the synthesis of 3-(*N*-acetamidomethyl)-benzo[*c*]isoxazole **5a**.

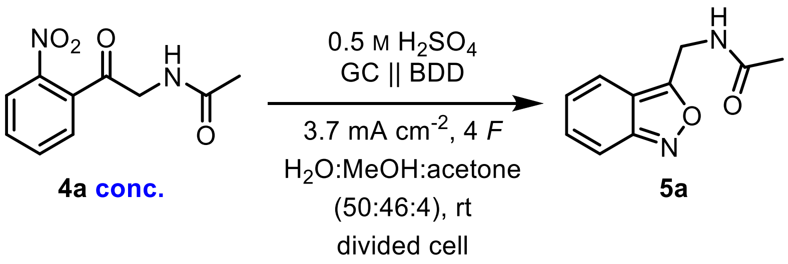

Entry	Substrate Concentration	Yield[a] [%]
1	0.02 M	2
2	0.03 M	72
3	0.04 M	51

[a]Yield determined by ^{1}H NMR, internal standard: 1,3,5-trimethoxybenzene.

Table S3: Optimisation of the electrolytic reaction conditions for the synthesis of 3-(*N*-acetamidomethyl)-benzo[*c*]isoxazole **5a**.

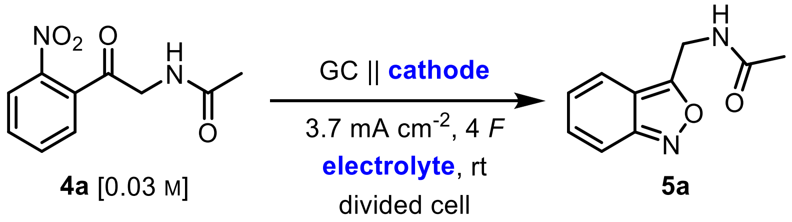

Entry	Cathode	Electrolyte	Yield[a] [%]
1	BDD	0.5 M H_2SO_4 in water:methanol:acetone (50:46:4)	72
2	BDD	1.0 M H_2SO_4 in water:methanol:acetone (50:46:4)	52
3	BDD	5 M AcOH/NaOAc (0.27 M) in methanol	41
4	BDD	5 M HCOOH/NaHCOO (0.5 M) in methanol	62
5	BDD	5 M HCOOH/NaHCOO (0.27 M) in methanol	65
6	BDD	5 M HCOOH/NaHCOO (0.5 M) + 0.2 mL TFA in methanol	n.d.
7	BDD	1 M NH_4COO + 0.2 mL TFA in methanol	56
8	BDD	1 M $MeSO_3H$ in methanol	n.d.
9	BDD	0.5 M H_2SO_4 in methanol	51
10	BDD	1.0 M H_2SO_4 in methanol	33
11	GC	1.0 M H_2SO_4 in methanol	52
12	GC	0.5 M H_2SO_4 in water:methanol:acetone (25:71:4)	36
13	GC	1.0 M H_2SO_4 in water:methanol:acetone (50:46:4)	79

[a]Yield determined by ^{1}H NMR, internal standard: 1,3,5-trimethoxybenzene; n.d. = not detected; TFA = trifluoroacetic acid.

Table S4: Optimisation of the electrolytic reaction conditions for the synthesis of 3-(*N*-acetamidomethyl)-benzo[*c*]isoxazole **5a**.

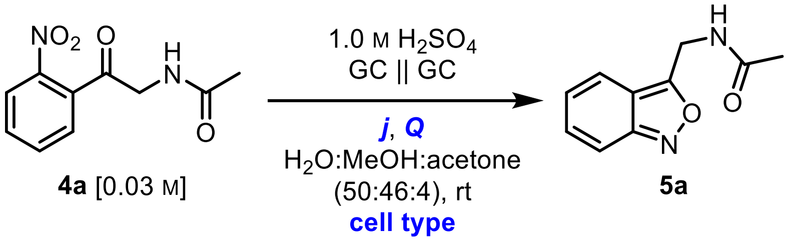

4a [0.03 M] **5a**

Entry	Cell Type	Current Density *j* / mA cm^{-2}	Amount of applied charge *Q* / *F*	Yield[a] [%]
1	divided	3.7	4.0	79
2	undivided	3.7	4.0	82
3	undivided	3.7	4.5	81
4	undivided	3.7	5.0	41
5	undivided	4.1	4.0	76
6	undivided	4.5	4.0	57

[a]Yield determined by ^{1}H NMR, internal standard: 1,3,5-trimethoxybenzene.

Table S5: Optimisation of the electrolytic reaction conditions for the synthesis of 3-(*N*-acetamidomethyl)-benzo[*c*]isoxazole **5a**.

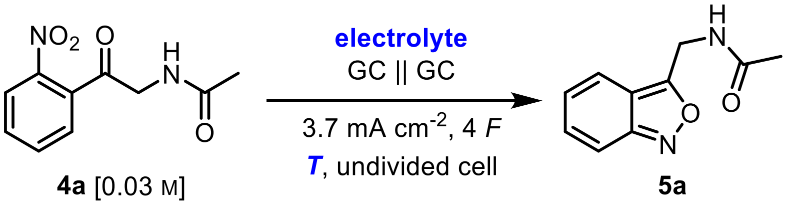

4a [0.03 M] **5a**

Entry	Electrolyte	Temperature / °C	Yield[a] [%]
1	1.0 M H_2SO_4 in water:methanol:acetone (50:46:4)	rt	82
2	1.0 M H_2SO_4 in water:methanol (1:1)	rt	70
3	1.0 M H_2SO_4 in water:methanol (1:3)	rt	28
4	1.0 M H_2SO_4 in water:ethanol (1:1)	rt	77
5	0.5 M NBu_4HSO_4 in water:methanol:acetone (50:46:4)	rt	69
6	1.0 M NBu_4HSO_4 in water:methanol:acetone (50:46:4)	rt	64
7	0.5 M $KHSO_4$ in water:methanol:acetone (50:46:4)	rt	82
8	1.0 M $KHSO_4$ in water:methanol:acetone (50:46:4)	rt	80
9	0.5 M NH_4HCOO + 0.5 M TFA in methanol	rt	18
10	0.5 M NH_4HCOO + 1.0 M TFA in methanol	rt	14
11	0.1 M H_2SO_4 in water:methanol:acetone (50:46:4)	rt	79
12	0.2 M H_2SO_4 in water:methanol:acetone (50:46:4)	rt	80
13	0.3 M H_2SO_4 in water:methanol:acetone (50:46:4)	rt	80
14	0.4 M H_2SO_4 in water:methanol:acetone (50:46:4)	rt	76
15	0.5 M H_2SO_4 in water:methanol:acetone (50:46:4)	rt	85
16	0.6 M H_2SO_4 in water:methanol:acetone (50:46:4)	rt	83
17	0.7 M H_2SO_4 in water:methanol:acetone (50:46:4)	rt	84
18	0.8 M H_2SO_4 in water:methanol:acetone (50:46:4)	rt	87
19	0.9 M H_2SO_4 in water:methanol:acetone (50:46:4)	rt	81
20	0.8 M H_2SO_4 in water:methanol:acetone (50:46:4)	50	17

[a]Yield determined by ^{1}H NMR, internal standard: 1,3,5-trimethoxybenzene; TFA = trifluoroacetic acid.

5. General Protocols

5.1. General Protocol for the Synthesis of Substituted 1-(2-Nitrophenyl)alkan-1-one *O*-tosyl oximes (GP 1)

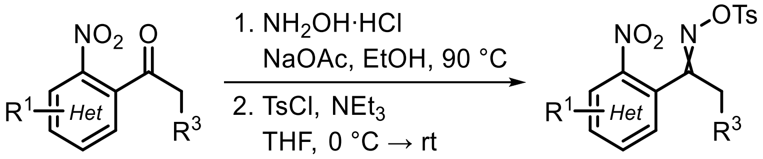

The corresponding 1-(2-nitrophenyl)ethan-1-one (1.0 eq.), sodium acetate (2.0 eq.) and hydroxylamine hydrochloride (1.5 eq.) were reacted in ethanol at 90 °C. After completion of the reaction (TLC) 50 mL of water was added and the ethanol was removed under reduced pressure. The aqueous fraction was extracted three times with 50 mL of ethyl acetate. The combined organic fractions were washed once with 50 mL of brine, dried over sodium sulphate and the solvent was removed under reduced pressure. Under nitrogen atmosphere the crude product was dissolved in anhydrous THF, triethylamine (2.0 eq.) was added, and the solution was cooled to 0 °C. Tosyl chloride (1.3 eq.) was dissolved in anhydrous THF and added dropwise. The reaction mixture was allowed to warm to room temperature and stirred until completion of the reaction (LC-MS). Afterwards, 50 mL of water was added and the THF was removed under reduced pressure. The aqueous fraction was extracted three times with 50 mL of ethyl acetate. The combined organic fractions were washed once with 50 mL of water, once with 50 mL of brine and dried over sodium sulphate. The solvent was removed under reduced pressure until the product precipitated. The solids were collected by vacuum filtration, washed twice with cold ethanol, and dried under a vacuum.

5.2. General protocol for the synthesis of 4-ethoxycarbonyl-5-(2-nitrophenyl)oxazoles (GP 2)

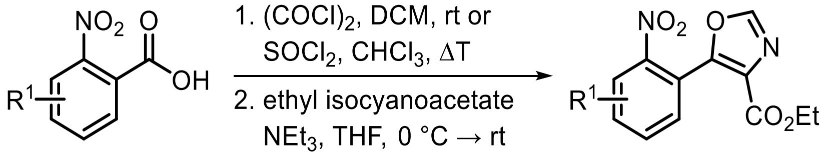

Under nitrogen atmosphere the corresponding 2-nitrobenzoic acid (1.0 eq.) was dissolved in dichloromethane or chloroform. Oxalyl chloride or thionyl chloride (1.5 eq.) was added. If oxalyl chloride was used, three drops of DMF were added. The solution was stirred at room temperature or reflux for 24 h. Afterwards, the solvent was removed under reduced pressure. The crude acid chloride was directly used without further purification. Triethylamine (3.0 eq.) was dissolved in anhydrous THF under inert atmosphere and ethyl isocyanoacetate (1.0 eq.) was added slowly. The mixture was stirred for 1 h at room temperature before it was cooled to 0 °C. The crude acid chloride was added to the solution and the mixture was stirred until completion of the reaction (LC-MS). Afterwards, 50 mL of water was added, and the aqueous fraction was extracted three times with 50 mL of ethyl acetate. The combined organic fractions were washed once with 50 mL of 1 M hydrochloric acid, once with 50 mL of 1 M aqueous sodium carbonate solution, and once with 50 mL of brine and dried over sodium sulphate. The solvent was removed under reduced pressure. The crude products were purified using silica flash column chromatography.

5.3. General protocol for the synthesis of 2-nitro-1-(2-nitrophenyl)alkan-1-ones (GP 3)

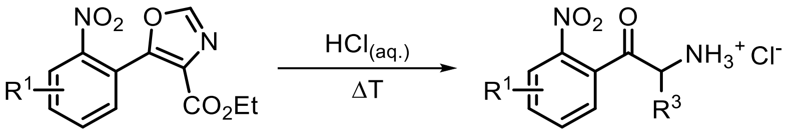

The corresponding 4-ethoxycarbonyl-5-(2-nitrophenyl)oxazole (1.0 eq.) was dissolved in 25 mL of 6 M hydrochloric acid and were reacted at 95 °C for 6 h. Afterwards, the mixture was cooled to room temperature and the aqueous fraction was washed two times with 25 mL of diethyl ether. The solvent of the aqueous fraction was removed under reduced pressure and the crude product was used without further purification.

5.4. General Protocol for the Synthesis of Substituted 1-(2-Nitrophenyl)alkan-1-one *O*-tosyl oximes (GP 4)

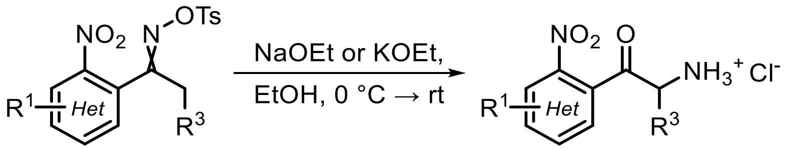

Under argon atmosphere the corresponding 1-(2-nitrophenyl)alkan-1-one *O*-tosyl oxime (1.0 eq.) was suspended in anhydrous ethanol and cooled to 0 °C. A solution of sodium ethoxide (20% *w*/*v*) or a freshly prepared saturated potassium ethoxide solution in ethanol was added dropwise (2.0 eq.). The reaction mixture was allowed to warm to room temperature and stirred until completion of the reaction (LC-MS). Resulting solids were filtered off and the filtrate was treated with 6 M hydrochloric acid until a pH value of 1 was reached. The aqueous fraction was washed two times with 25 mL of diethyl ether. The solvent of the aqueous fraction was removed under reduced pressure and the crude product was used without further purification.

5.5. General protocol for the synthesis of *N*-(2-nitrophenacyl)acylamides using acid chlorides or anhydrides (GP 5)

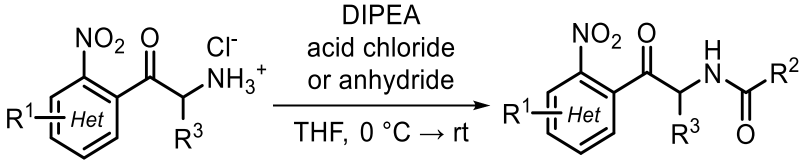

Under nitrogen atmosphere the corresponding 2-amino-1-(2-nitrophenyl)alkan-1-one hydrochloride (1.0 eq.) and acid chloride or anhydride (1.5 eq.) were suspended in anhydrous THF and cooled to 0 °C. diisopropylethylamine (DIPEA, 2.0 eq.) was added in one portion and the reaction mixture was allowed to warm to room temperature. After completion of the reaction (TLC) 50 mL of water was added and the THF was removed under reduced pressure. The aqueous fraction was extracted three times with 50 mL of ethyl acetate. The combined organic fractions were washed once with 50 mL of brine, dried over sodium sulphate and the solvent was removed under reduced pressure. The crude products were purified using silica flash column chromatography.

5.6. General protocol for the synthesis of *N*-(2-Nitrophenacyl)acylamides using carboxylic acids (GP 6)

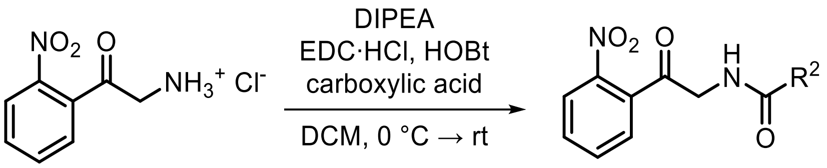

Under nitrogen atmosphere the corresponding carboxylic acid (1.2 eq.), 1-ethyl-3-(3-dimethylaminopropyl)carbodiimide hydrochloride (EDC·HCl, 1.2 eq.) and 1-hydroxybenzotriazole (only for chiral carboxylic acids; HOBt, 1.2 eq.) were dissolved in dichloromethane, cooled to 0 °C and stirred for 15 min. 2-amino-1-(2-nitrophenyl)ethan-1-one hydrochloride (1.0 eq.) was added in one portion and the reaction mixture was stirred for 24 h allowed to warm to room temperature. 50 mL of water was added and the fractions were separated. The aqueous fraction was extracted three times with 50 mL of dichloromethane. The combined organic fractions were washed twice with 50 mL of 1 M hydrochloric acid, once with 50 mL of water, once with 50 mL of brine and dried over sodium sulphate. The solvent was removed under reduced pressure and the crude products were purified using silica flash column chromatography.

5.7. General Protocol for the Electrochemical Synthesis of substituted 2,1-benzo[*c*]isoxazoles (GP 7)

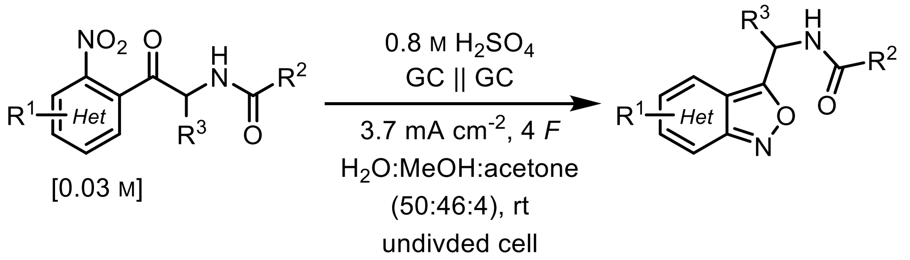

5 mL Undivided Teflon™ Screening Cell: 0.15 mmol of the starting material was dissolved in 0.2 mL of acetone and 2.3 mL of methanol in the undivided cell. 2.0 mL of 2.0 M sulphuric acid and 0.5 mL of water was added. The electrodes (1 cm·7 cm·0.3 cm) immersed 1.5 cm into the solution resulting in an area of 1.5 cm^{-2}. The electrolysis was performed under constant current conditions (current density j = 3.7 $mA{\cdot}cm^{-2}$ for 57.9 C). After the electrolysis the reaction mixture was diluted with 5 mL of water and extracted three times with 10 mL of ethyl acetate. The aqueous fraction was analysed by LC-MS to ensure the complete extraction of the product. If necessary, the aqueous fraction was extracted additionally twice with 10 mL of ethyl acetate. The combined organic fractions were washed twice with 10 mL of 1 M hydrochloric acid once with 5 mL of brine, dried over sodium sulphate and the solvent was removed under reduced pressure. If the aniline formed during electrolysis was not removed by the 1 M hydrochloric acid extraction the organic fraction was additionally washed twice with 10 mL of 2 M hydrochloric acid. The crude product was purified by silica column chromatography (SiO_2) or reverse phase column chromatography (C_{18}).

25 mL Undivided Glass Cell: 0.75 mmol of the starting material was dissolved in 1 mL of acetone, 11.5 mL of methanol in the undivided cell and 10 mL of 2.0 M sulphuric acid and 2.5 mL of water was added was added. The electrodes (2 cm·6 cm·0.3 cm) immersed 3.0 cm into the solution resulting in an area of 6.0 cm^{-2}. The electrolysis was performed under constant current conditions (current density j = 3.7 $mA{\cdot}cm^{-2}$ for 289.5 C). After the electrolysis the reaction mixture was diluted with 15 mL of water and 15 mL of brine and extracted four times with 30 mL of ethyl acetate. The aqueous fraction was analysed by LC-MS to ensure the complete extraction of the product. If necessary, the aqueous fraction was extracted additionally twice with 30 mL of ethyl acetate. The combined organic fractions were washed twice with 20 mL of 1 M hydrochloric acid, once with 15 mL of brine, dried over sodium sulphate and the solvent was removed under reduced pressure. The crude product was purified by silica column chromatography (SiO_2).

100 mL Undivided Glass Cell: 3.0 mmol of the starting material was dissolved in 4 mL of acetone, 46 mL of methanol in the undivided cell and 40 mL of 2.0 M sulphuric acid and 10 mL of water was added. The electrodes (2 cm·6 cm·0.3 cm) immersed 4.7 cm into the solution resulting in an area of 9.4 cm^{-2}. The electrolysis was performed under constant current conditions (current density *j* = 3.7 mA·cm^{-2} for 1157.8 C). After the electrolysis the reaction mixture was diluted with 25 mL of water and 15 mL of brine and extracted four times with 30 mL of ethyl acetate. The aqueous fraction was analysed by LC-MS to ensure the complete extraction of the product. If necessary, the aqueous fraction was extracted additionally twice with 30 mL of ethyl acetate. The combined organic fractions were washed twice with 30 mL of 1 M hydrochloric acid, once with 25 mL of brine, dried over sodium sulphate and the solvent was removed under reduced pressure. The crude product was purified by silica column chromatography (SiO_2).

250 mL Undivided Glass Cell: 7.5 mmol of the starting material was dissolved in 10 mL of acetone, 115 mL of methanol in the undivided cell and 100 mL of 2.0 M sulphuric acid and 25 mL of water was added. The electrodes (3.7 cm·13 cm·0.3 cm) immersed 8.0 cm into the solution resulting in an area of 29.6 cm^{-2}. The electrolysis was performed under constant current conditions (current density *j* = 3.7 mA·cm^{-2} for 3039.3 C). After the electrolysis the reaction mixture was diluted with 50 mL of water and the organic solvent was removed under reduced pressure. The aqueous fraction was extracted four times with 50 mL of ethyl acetate. The aqueous fraction was analysed via LC-MS to ensure the complete extraction of the product. If necessary, the aqueous fraction was extracted additionally twice with 50 mL of ethyl acetate. The combined organic fractions were washed twice with 30 mL of 1 M hydrochloric acid, once with 50 mL of brine, dried over sodium sulphate and the solvent was removed under reduced pressure. The crude product was purified by silica column chromatography (SiO_2).

6. Scale-up of the Electrochemical Reductive Synthesis of 3-(*N*-Acetamidomethyl)-benzo[*c*]isoxazole (5a) and 3-Methylbenzo[*c*]isoxazole (5ai)

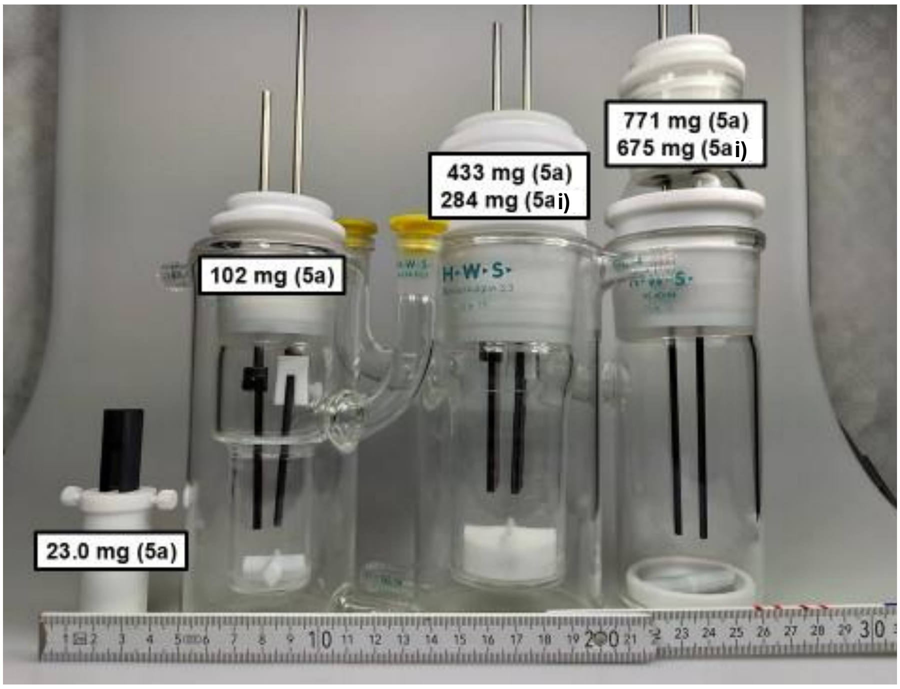

Figure S18: Different batch-type undivided cells used for the scale-up of the reaction. The ruler depicted on the bottom is in cm.

Table S1: Scale-up of the electrochemical synthesis of 5a and 5ai.

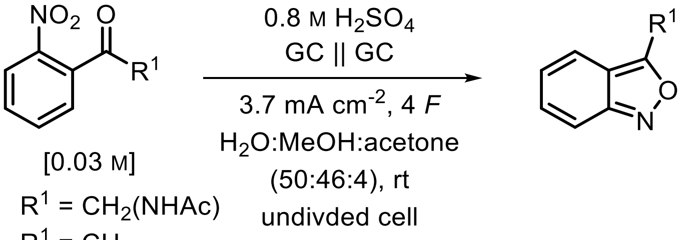

[0.03 M]

4a & 5a R^1 = CH_2(NHAc)

5ai R^1 = CH_3

Cell Volume [mL]	Scale [mmol]	Applied Charge	Yield [%] of 5a	Yield [%] of 5ai
5	0.15	57.9 C<br>2.9 h	23.0 mg<br>(0.122 mmol, 81%)	-
25	0.75	289.5 C<br>3.6 h	102 mg<br>(0.535 mmol, 71%)	-
100	3.0	1157.8 C<br>9.2 h	433 mg<br>(2.3 mmol, 77%)	284 mg<br>(2.1 mmol, 71%)
250	7.5	3039.3 C<br>7.7 h	771 mg<br>(4.0 mmol, 54%)	675 mg<br>(5.1 mmol, 68%)

Isolated yields.

7. Preparation of Products and Analytical Data

N-Isobut-1-enylmorpholine (8)

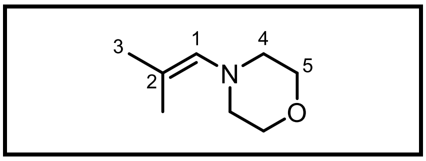

Isobutyraldehyde (46.0 mL, 0.5 mol, 1.0 eq.) and morpholine (43.6 mL, 0.5 mol, 1.0 eq.) were slowly mixed in a round bottom flask under cooling. The flask was equipped with a *Dean-Stark* trap which was filled with an excess of the isobutyraldehyde. The mixture was heated under reflux until the water formation stopped (48 h). The excess aldehyde was removed at atmospheric pressure. 51.40 g (0.364 mol, 73%) of the product was obtained as a colourless liquid after distillation under reduced pressure (40 – 42 °C, 3 mbar).

^{1}H NMR (400 MHz, $CDCl_3$) δ [ppm]: 5.25 (s, 1H, *H*-1), 3.68 – 3.63 (m, 4H, *H*-4), 2.55 – 2.48 (m, 4H, *H*-5), 1.61 (s, 3H, *H*-3), 1.54 (s, 3H, *H*-3).
^{13}C NMR (101 MHz, $CDCl_3$) δ [ppm]: 135.2, 123.4, 66.9, 53.0, 22.2, 17.3.
HR-MS (ESI+): *m/z* for $C_8H_{15}NO+H^+$, $[M+H]^+$ calculated: 142.1226; found: 142.1222.
The analytical data matches with the data reported in literature.[5]

Methyl 2-nitro-3,4,5-trimethoxybenzoic acid (9)

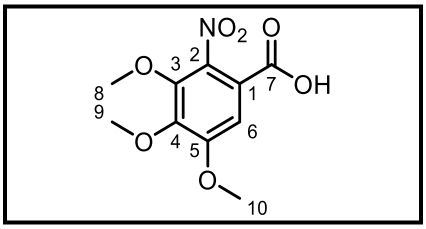

Methyl 2-nitro-3,4,5-trimethoxybenzoate (10.0 g, 37 mmol, 1.0 eq.) and potassium hydroxide (2.1 g, 37 mol, 1.0 eq.) were dissolved in 50 mL of methanol. The mixture was heated to 40 °C for 3 h. Afterwards, 50 mL of water were added and the aqueous fraction was washed two times with 50 mL of diethyl ether. The aqueous fraction was treated with 6 M hydrochloric acid until a pH value of 1 was reached. The aqueous fraction was extracted three times with 50 mL of diethyl ether and the solvent was removed under reduced pressure. 8.5 g (33 mmol, 90%) of the product was obtained as a colourless solid.

^{1}H NMR (400 MHz, CD_3CN) δ [ppm]: 7.32 (s, 1H, *H*-6), 3.93 (s, 3H), 3.92 (s, 4H), 3.91 (s, 3H).
^{13}C NMR (101 MHz, CD_3CN) δ [ppm]: 164.2, 155.4, 147.3, 146.4, 140.9, 110.1, 63.3, 61.8, 57.3.
HR-MS (ESI+): *m/z* for $C_{10}H_{11}NO_7+Na^+$, $[M+Na]^+$ calculated: 280.0428; found: 280.0430.

1-(2-nitrophenyl)prop-2-en-1-ol (10)

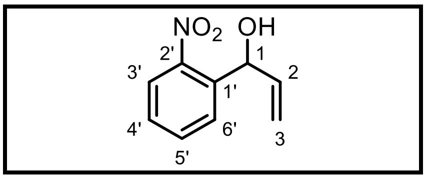

2-Nitrobenzaldehyde (6.04 g, 40.0 mmol, 1.0 eq.) was dissolved in 100 mL of anhydrous THF and cooled to –78°c prior to dropwise addition of vinylmagnesium bromide (68.6 mL, 48.0 mmol, 0.7 M, 1.2 eq.). The mixture was stirred for 3 h at –78°C and was slowly warmed to room temperature over 17 h. 50 mL of saturated ammonium chloride solution were added, and the aqueous phase was extracted three times with 50 mL of ethyl acetate. The combined organic phases were dried over sodium sulphate and the solvent was removed under reduced pressure. 4.87 g, (27.2 mmol, 68%) of the product was obtained as a slightly red oil after silica flash column chromatography (cyclohexane:ethyl acetate SIHC: 120 g regular silica column; particle size: 25 µm) using 10 → 25% ethyl acetate

^{1}H NMR (400 MHz, $CDCl_3$) δ [ppm]: 7.89 (dd, *J* = 8.1, 1.4 Hz, 1H, *H*-3'), 7.75 (dd, *J* = 7.9, 1.5 Hz, 1H, *H*-6'), 7.63 (td, *J* = 7.6, 1.4 Hz, 1H, *H*-5'), 7.43 (ddd, *J* = 8.6, 7.4, 1.5 Hz, 1H, *H*-4'), 6.06 (ddd, *J* = 17.1, 10.5, 5.2 Hz, 1H, *H*-2), 5.77 (dt, *J* = 5.3, 1.6 Hz, 1H, *H*-1), 5.39 (dt, *J* = 17.1, 1.4 Hz, 1H, *H*-3), 5.24 (dt, *J* = 10.5, 1.3 Hz, 1H, *H*-3), 2.69 (s, 1H, 1-O*H*).
^{13}C NMR (101 MHz, $CDCl_3$) δ [ppm]: 148.4, 138.1, 137.6, 133.7, 128.9, 128.6, 124.6, 116.3, 70.0.

HR-MS (ESI+): *m/z* for $C_9H_8NO_2+Na^+$, $[M+H-H_2O]^+$ calculated: 162.0550; found: 162.0544..

7.1. 1-(2-nitrophenyl)alkan-1-ones (11-14)

1-(2-Nitrophenyl)propan-1-one (11)

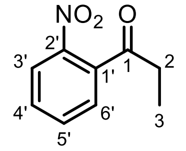

Under nitrogen atmosphere sodium hydride (60% dispersion in mineral oil, 2.40 g, 60 mmol, 1.2 eq.) was suspended in 150 mL of anhydrous THF and cooled to 0 °C. Dimethyl methylmalonate (8.71 mL, 50 mmol, 1.0 eq.) was added slowly and the suspension was stirred for 1 h at 0°C (strong hydrogen evolution started after an incubation time of 5 min which should be considered when choosing the reaction vessel). 2-nitrobenzoyl chloride (9.28 g, 50 mmol, 1.0 eq.) was diluted with 50 mL of anhydrous THF and added dropwise to the reaction mixture. The reaction mixture was allowed to warm to room temperature and stirred for 24 h. The reaction mixture was added to 100 mL of water under constant stirring. The THF was removed under reduced pressure. Afterwards. the aqueous fraction was extracted three times with 75 mL of ethyl acetate. The combined organic fractions were washed once with 50 mL of brine, dried over sodium sulphate and the solvent was removed under reduced pressure. The crude product was used without further purification in the next step. In a round bottom flask, to the crude product 40 mL of water, 40 mL of glacial acetic acid and 20 mL of concentrated sulphuric acid were added and heated at 100 °C. After completion of the reaction (LC-MS) the reaction mixture was neutralised with saturated sodium carbonate solution. The mixture was extracted three times with 75 mL of ethyl acetate. The combined organic fractions were washed twice with 1 M sodium hydroxide, once with 50 mL of water, once with 50 mL of brine and dried over sodium sulphate. The solvent was removed under reduced pressure. 6.09 g (34 mmol, 68 %) of the product were obtained as a slightly yellow oil after silica flash column chromatography (cyclohexane:ethyl acetate SIHC: 80 g regular silica column; particle size: 25 µm) using 0 → 10% ethyl acetate.

^{1}H NMR (400 MHz, CD_2Cl_2) δ [ppm]: 8.08 (dd, *J* = 8.3 Hz, 1.2 Hz, 1H, *H*-3'), 7.74 (td, *J* = 7.5 Hz, 1.2 Hz, 1H, *H*-5'), 7.61 (ddd, *J* = 8.3 Hz, 7.5 Hz, 1.5 Hz, 1H, *H*-4'), 7.42 (dd, *J* = 7.6 Hz, 1.5 Hz, 1H, *H*-6'), 2.80 (q, *J* = 7.2 Hz, 2H, *H*-2), 1.21 (t, *J* = 7.2 Hz, 3H, *H*-3).
^{13}C NMR (101 MHz, CD_2Cl_2) δ [ppm]: 203.4, 146.1, 138.3, 134.7, 130.9, 127.7, 124.7, 36.5, 8.2.
HR-MS (APCI-): *m/z* for $C_9H_9NO_3$-H^-, $[M-H]^-$ calculated: 178.0510; found: 178.0501.

2-Methyl-1-(2-nitrophenyl)propan-1-one (12)

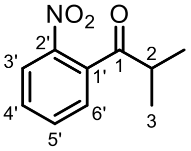

Under nitrogen atmosphere N-Isobut-1-enylmorpholine (**8**, 14.12 g, 0.1 mol, 1.0 eq.) was dissolved in 150 mL of anhydrous THF and cooled to 0 °C. 2-Nitrobenzoyl chloride (18.56 g, 0.1 mol, 1.0 eq.) in 50 mL of anhydrous THF was added dropwise. The reaction mixture was allowed to warm to room temperature and stirred for 24 h. The solids were collected by vacuum filtration. The hygroscopic solids were dissolved in 100 mL of water and 30 mL of concentrated hydrochloric acid were added. The resulting solution was stirred at 70 °C. After completion of the reaction (LC-MS) the mixture was cooled to room temperature and extracted three times with 50 mL of ethyl acetate. The combined organic fractions were washed once with 50 mL of brine, dried over sodium sulphate and the solvent was removed under reduced pressure. 5.085 g (26 mmol, 26%) of the product were obtained as a slightly red oil after silica flash column chromatography (cyclohexane:ethyl acetate SIHC: 80 g regular silica column; particle size: 25 µm) using 0 → 10% ethyl acetate.

^{1}H NMR (400 MHz, CD_2Cl_2) δ [ppm]: 8.12 (dd, *J* = 8.3 Hz, 1.2 Hz, 1H, *H*-3'), 7.74 (td, *J* = 7.6 Hz, 1.2 Hz, 1H, *H*-5'), 7.62 (ddd, *J* = 8.3 Hz, 7.5 Hz, 1.5 Hz, 1H, *H*-4'), 7.39 (dd, *J* = 7.6 Hz, 1.5 Hz, 1H, *H*-6'), 2.98 (hept, *J* = 6.9 Hz, 1H, *H*-2), 1.20 (d, *J* = 6.9 Hz, 6H, *H*-3).
^{13}C NMR (101 MHz, CD_2Cl_2) δ [ppm]: 206.8, 146.4, 137.9, 134.6, 130.8, 128.4, 124.8, 41.2, 18.5.
HR-MS (ESI+): *m/z* for $C_{10}H_{11}NO_3$+H^+, $[M+H]^+$ calculated: 194.0812; found: 194.0811.

1-(2-Nitrophenyl)-2-phenylethan-1-one (13)

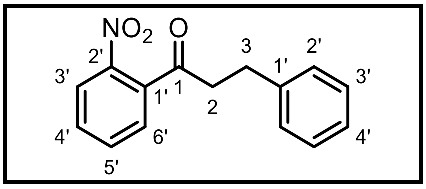

Under nitrogen atmosphere sodium hydride (60% dispersion in mineral oil, 2.23 g, 55 mmol, 1.1 eq.) was suspended in 150 mL of anhydrous THF and cooled to 0 °C. Dimethyl benzylmalonate (12.22 g, 55 mmol, 1.1 eq.) was added slowly and the suspension was stirred for 1 h at 0°C (strong hydrogen evolution started after an incubation time of 5 min which should be considered when choosing the reaction vessel). 2-nitrobenzoyl chloride (9.28 g, 50 mmol, 1.0 eq.) was diluted with 50 mL of anhydrous THF and added dropwise to the reaction mixture. The reaction mixture was allowed to warm to room temperature and stirred for 24 h. The reaction mixture was added to 100 mL of water under constant stirring. The THF was removed under reduced pressure. Afterwards. the aqueous fraction was extracted three times with 75 mL of ethyl acetate. The combined organic fractions were washed once with 50 mL of brine, dried over sodium sulphate and the solvent was removed under reduced pressure. The crude product was used without further purification in the next step. In a round bottom flask, to the crude product 40 mL of water, 40 mL of glacial acetic acid and 20 mL of concentrated sulphuric acid were added and heated at 100 °C. After completion of the reaction (LC-MS) the reaction mixture was neutralised with saturated sodium carbonate solution. The mixture was extracted three times with 75 mL of ethyl acetate. The combined organic fractions were washed twice with 1 M sodium hydroxide, once with 50 mL of water, once with 50 mL of brine and dried over sodium sulphate. The solvent was removed under reduced pressure. 7.00 g (27 mmol, 49 %) of the product were obtained as a slightly yellow oil after silica flash column chromatography (cyclohexane:ethyl acetate SIHC: 80 g regular silica column; particle size: 25 µm) using 3 → 17% ethyl acetate.

^{1}H NMR (400 MHz, CD_2Cl_2) δ [ppm]: 8.09 (dd, J = 8.2 Hz, 1.2 Hz, 1H, *H*-3'), 7.72 (td, J = 7.5 Hz, 1.2 Hz, 1H, *H*-5'), 7.61 (ddd, J = 8.2 Hz, 7.5 Hz, 1.5 Hz, 1H, *H*-4'), 7.40 – 7.21 (m, 6H, *H*-4',*H*-2'', *H*-3'', *H*-4''), 3.19 – 3.08 (m, 4H, *H*-2, *H*-3).
^{13}C NMR (101 MHz, CD_2Cl_2) δ [ppm]: 201.9, 146.2, 141.2, 138.1, 134.8, 131.2, 129.0, 128.9, 127.9, 126.7, 124.8, 44.8, 30.4.
HR-MS (ESI+): *m/z* for $C_{15}H_{13}NO_3$+H$^+$, [M+H]$^+$ calculated: 256.0968; found: 256.0970.

1-(2-Nitrophenyl)propen-1-one (14)

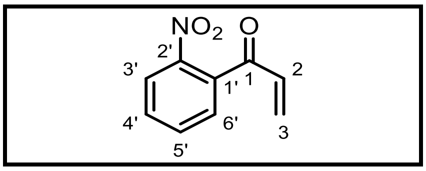

1-(2-Nitrophenyl)prop-2-en-1-ol (**10**, 5.76 g, 32 mmol, 1.0 eq.) was dissolved in 150 mL dichloromethane and the solution was cooled to 0 °C. Dess-Martin-Periodinan (16.34 g, 39 mmol, 1.2 eq.) was added in portions and the mixture was stirred for 24 h. The crude mixture was filtered over Celite™ 545. The organic fraction was washed three times with 50 mL half-saturated sodium carbonate solution, once with 50 mL brine, dried over sodium sulphate and the solvent was removed. 2.04 g (12 mmol, 38 %) of the product were obtained as a slightly yellow oil after silica flash column chromatography (cyclohexane:ethyl acetate SIHC: 80 g regular silica column; particle size: 25 µm) using 0 → 17% ethyl acetate.

^{1}H NMR (400 MHz, CD_2Cl_2) δ [ppm]: 8.12 (dd, J = 8.2 Hz, 1.2 Hz, 1H, *H*-3'), 7.77 (td, J = 7.5 Hz, 1.2 Hz, 1H, *H*-5'), 7.66 (ddd, J = 8.2 Hz, 7.5 Hz, 1.5 Hz, 1H, *H*-4'), 7.45 (dd, J = 7.5 Hz, 1.5 Hz, 1H, *H*-6'), 6.62 (dd, J = 17.7 Hz, 10.7 Hz, 1H, *H*-2), 6.05 (d, J = 10.7 Hz, 1H, *H*-3), 5.86 (d, J = 17.7 Hz, 1H, *H*-3).
^{13}C NMR (101 MHz, CD_2Cl_2) δ [ppm]: 193.6, 147.1, 136.7, 135.6, 134.5, 131.8, 131.2, 129.2, 124.7.
HR-MS (ESI+): *m/z* for $C_9H_7NO_3$+H$^+$, [M+H]$^+$ calculated: 178.0499; found: 178.0493.

7.2. 1-(2-nitrophenyl)alkan-1-one *O*-tosyl oximes (15-19)

1-(5-Chloro-2-nitrophenyl)ethan-1-one *O*-tosyl oxime (*E*/*Z*-Mixture) (15)

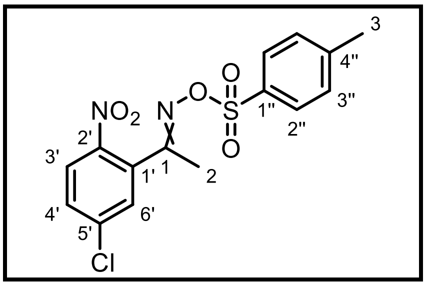

According to **GP1** 1-(5-chloro-2-nitrophenyl)ethan-1-one (5.00 g, 25 mmol, 1.0 equiv), sodium acetate (3.19 g, 39 mmol, 1.6 eq.) and hydroxylamine hydrochloride (2.61 g, 38 mmol, 1.5 eq.) were reacted in 130 mL of ethanol. The crude oxime was used without further purification after work-up.
The crude 1-(5-chloro-2-nitrophenyl)ethan-1-one oxime (*E*/*Z*-mixture, 5.00 g), tosyl chloride (4.89 g, 26 mmol, 1.3 eq.) and triethylamine (3.6 mL) were reacted in 100 mL of anhydrous THF. 4.97 g (23 mmol, 58%) of the product were obtained as an off-white solid.

Major: ^{1}H NMR (400 MHz, $CDCl_3$) δ [ppm]: 8.03 (d, *J* = 8.8 Hz, 1H, *H*-3'), 7.83 (d, *J* = 8.4 Hz, 2H, *H*-2''), 7.55 (dd, *J* = 8.8 Hz, 2.3 Hz, 1H, *H*-4'), 7.36 – 7.32 (m, 3H, *H*-6', *H*-3''), 2.45 (s, 3H, *H*-3), 2.30 (s, 3H, *H*-2).
Minor: ^{1}H NMR (400 MHz, $CDCl_3$) δ [ppm]: 8.15 (d, *J* = 8.9 Hz, 1H, *H*-3'), 7.78 – 7.73 (m, 2H, *H*-2''), 7.56 (ddd, *J* = 8.8 Hz, 5.2 Hz, 2.2 Hz, 1H, *H*-4'), 7.39 – 7.30 (m, 2H, *H*-3''), 7.14 (d, *J* = 2.2 Hz, 1H, *H*-6'), 2.47 (s, 3H, *H*-3), 2.30 (s, 3H, *H*-2).
^{13}C NMR (101 MHz, $CDCl_3$) δ [ppm]: 163.5, 161.5, 145.6, 145.5, 141.4, 140.7, 132.6, 132.4, 132.2, 131.2, 130.9, 130.8, 130.7, 129.9, 128.9, 128.0, 126.5, 126.5, 21.9, 21.9, 21.6, 18.0.
HR-MS (ESI+): *m/z* for $C_{15}H_{13}{}^{35}ClN_2O_5S_2+Na^+$, $[M+Na]^+$ calculated: 391.0126; found: 391.0122.

1-(5-Bromo-2-nitrophenyl)ethan-1-one *O*-tosyl oxime (*E*/*Z*-Mixture) (16)

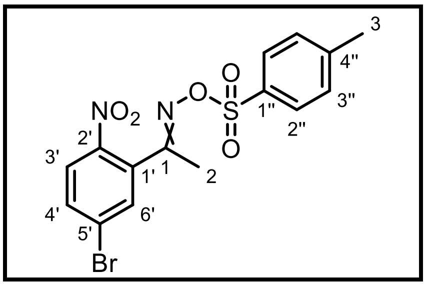

According to **GP1** 1-(5-bromo-2-nitrophenyl)ethan-1-one (6.89 g, 28 mmol, 1.0 eq.), sodium acetate (4.99 g, 56 mmol, 2.0 eq.) and hydroxylamine hydrochloride (2.93 g, 42 mmol, 1.5 eq.) were reacted in 150 mL of ethanol. The crude oxime was used without further purification after work-up.
The crude 1-(5-bromo-2-nitrophenyl)ethan-1-one oxime (*E*/*Z*-mixture, 6.83 g), tosyl chloride (6.54 g, 34 mmol, 1.3 eq.) and triethylamine (7.1 mL) were reacted in 100 mL of anhydrous THF. 5.76 g (14 mmol, 50%) of the product were obtained as an off-white solid.

^{1}H NMR (400 MHz, CD_3CN) δ [ppm]: 8.09 (d, *J* = 8.8 Hz, 1H, *H*-3'), 7.97 (d, *J* = 8.8 Hz, 1H, *H*-3'), 7.86 – 7.75 (m, 4H, *H*-4', *H*-2''), 7.73 – 7.69 (m, 2H), 7.53 (d, *J* = 2.2 Hz, 1H, *H*-6'), 7.47 (d, *J* = 2.2 Hz, 1H, *H*-6'), 7.46 – 7.39 (m, 4H, *H*-3''), 2.46 (s, 3H, *H*-3), 2.44 (s, 3H, *H*-3), 2.27 (s, 3H, *H*-2), 2.27 (s, 3H, *H*-2).
^{13}C NMR (101 MHz, CD_3CN) δ [ppm]: 165.3, 163.6, 147.1, 147.0, 135.2, 134.9, 134.2, 133.2, 132.9, 132.7, 131.7, 131.3, 130.9, 130.9, 129.9, 129.2, 127.7, 127.4, 21.7, 21.7, 21.4, 17.9.
HR-MS (ESI+): *m/z* for $C_{15}H_{13}{}^{79}BrN_2O_5S+Na^+$, $[M+Na]^+$ calculated: 412.9801; found: 412.9802.

1-(2-Nitrophenyl)propan-1-one *O*-tosyl oxime (17)

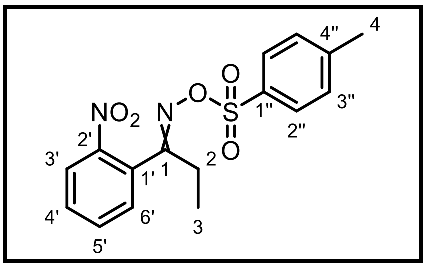

According to **GP1** 1-(2-nitrophenyl)propan-1-one (**9**, 5.38 g, 30 mmol, 1.0 eq.), sodium acetate (4.99 g, 60 mmol, 2.0 eq.) and hydroxylamine hydrochloride (3.13 g, 45 mmol, 1.5 eq.) were reacted in 150 mL of ethanol. The crude oxime was used without further purification after work-up.
The crude 1-(2-nitrophenyl)propan-1-one oxime (*E*/*Z*-mixture), tosyl chloride (7.44 g, 39 mmol, 1.3 eq.) and triethylamine (8.0fz mL) were reacted in 100 mL of anhydrous THF. 3.11 g (9 mmol, 30%) of the product were obtained as a colourless oil after silica flash column chromatography (cyclohexane:ethyl acetate SIHC: 80 g regular silica column; particle size: 25 µm) using 0 → 10% ethyl acetate.

^{1}H NMR (400 MHz, CD_3CN) δ [ppm]: 8.20 (dd, *J* = 8.3 Hz, 1.2 Hz, 1H, *H*-3'), 7.82 (td, *J* = 7.6 Hz, 1.2 Hz, 1H, *H*-5'), 7.76 – 7.65 (m, 3H, *H*-4', *H*-2''), 7.43 (dt, *J* = 8.0 Hz, 0.8 Hz, 2H, *H*-3''), 7.27 (dd, *J* = 7.7 Hz, 1.4 Hz, 1H, *H*-6'), 2.64 (qd, *J* = 7.2 Hz, 4.7 Hz, 2H, *H*-2), 2.46 (s, 3H, *H*-4), 1.08 (t, *J* = 7.4 Hz, 3H, *H*-3).
^{13}C NMR (101 MHz, CD_3CN) δ [ppm]: 168.3, 146.9, 135.6, 133.1, 131.8, 130.8, 130.8, 129.6, 129.3, 129.1, 125.7, 29.7, 21.7, 10.2.
HR-MS (ESI+): *m/z* for $C_{16}H_{16}N_2O_5$+H^+, $[M+H]^+$ calculated: 349.0853; found: 349.0844.

1-(2-Nitrophenyl)-2-methylpropan-1-one *O*-tosyl oxime (18)

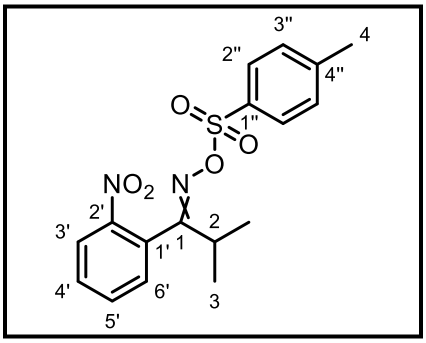

According to **GP1** 2-methyl-1-(2-nitrophenyl)propan-1-one (**10**, 1.16 g, 6 mmol, 1.0 eq.), sodium acetate (0.99 g, 12 mmol, 2.0 eq.) and hydroxylamine hydrochloride (0.63 g, 9 mmol, 1.5 eq.) were reacted in 50 mL of ethanol. The crude oxime was used without further purification after work-up.
The crude 2-methyl-1-(2-nitrophenyl)propan-1-one oxime (*E*/*Z*-mixture), tosyl chloride (1.72 g, 9 mmol, 1.5 eq.) and triethylamine (1.7 mL) were reacted in 100 mL of anhydrous THF. 1.49 g (6 mmol, 67%) of the product were obtained as a colourless oil after silica flash column chromatography (cyclohexane:ethyl acetate SIHC: 80 g regular silica column; particle size: 25 µm) using 0 → 10% ethyl acetate.

^{1}H NMR (400 MHz, CD_2Cl_2, major) δ [ppm]: 8.22 (dd, *J* = 8.3 Hz, 1.2 Hz, 1H, *H*-3'), 7.81 (td, *J* = 7.6 Hz, 1.2 Hz, 1H, *H*-5'), 7.76 – 7.67 (m, 1H, *H*-4', *H*-2''), 7.46 – 7.41 (m, 1H, *H*-3''), 7.22 (dd, *J* = 7.6 Hz, 1.4 Hz, 1H, *H*-6'), 2.85 (hept, *J* = 6.9 Hz, 1H, *H*-2), 2.46 (s, 3H, *H*-4), 1.14 (d, *J* = 6.9 Hz, 3H, *H*-3), 1.09 (d, *J* = 6.9 Hz, 1H, *H*-3).
^{1}H NMR (400 MHz, CD_2Cl_2, minor) δ [ppm]: 1.22 (d, *J* = 6.9 Hz, 3H, *H*-3)
^{13}C NMR (101 MHz, CD_2Cl_2, major) δ [ppm]: 171.1, 146.9, 135.5, 133.1, 131.8, 130.7, 129.7, 129.6, 128.8, 125.9, 36.2, 21.7, 19.8, 19.7.
HR-MS (ESI+): *m/z* for $C_{17}H_{18}N_2O_5S$+H^+, $[M+H]^+$ calculated: 362.1009; found: 362.1005.

1-(2-Nitrophenyl)-2-phenylethan-1-one *O*-tosyl oxime (19)

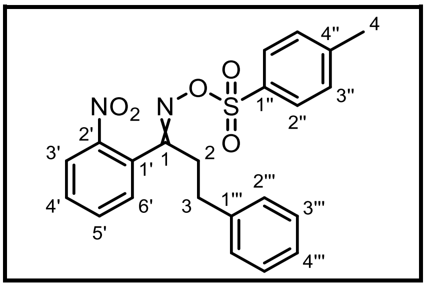

According to **GP1** 1-(2-nitrophenyl)-3-phenylpropan-1-one (**11**, 5.00 g, 20 mmol, 1.0 equiv), sodium acetate (2.49 g, 30 mmol, 1.6 eq.) and hydroxylamine hydrochloride (2.04 g, 29 mmol, 1.5 eq.) were reacted in 130 mL of ethanol. The crude oxime was used without further purification after work-up.

The crude 1-(2-nitrophenyl)-3-phenylpropan-1-one oxime (*E*/*Z*-mixture, 4.87 g), tosyl chloride (3.92 g, 21 mmol, 1.3 eq.) and triethylamine (3.0 mL) were reacted in 100 mL of anhydrous THF. 5.11 g (12 mmol, 67%) of the product were obtained as an off-white solid.

^{1}H NMR (400 MHz, CD_3CN) δ [ppm]: 8.19 (dd, J = 8.3, 1.2 Hz, 1H), 8.11 – 8.04 (m, 1H), 7.82 – 7.59 (m, 7H), 7.52 – 7.37 (m, 3H), 7.34 – 7.11 (m, 15H), 7.08 (dd, J = 8.4, 1.3 Hz, 1H), 7.01 – 6.96 (m, 6H), 6.84 (ddd, J = 8.5, 7.0, 1.3 Hz, 1H), 3.18 – 2.73 (m, 7H), 2.65 – 2.56 (m, 2H), 2.46 (s, 3H), 2.38 (s, 3H).

^{13}C NMR (101 MHz, CD_3CN) δ [ppm]: 174.6, 166.7, 146.9, 146.7, 143.6, 143.5, 141.9, 141.3, 139.8, 136.7, 135.6, 135.2, 133.0, 131.9, 130.8, 130.3, 129.8, 129.6, 129.4, 129.4, 129.3, 129.3, 129.1, 127.2, 127.1, 127.1, 126.8, 125.7, 125.3, 121.1, 119.4, 37.4, 35.8, 31.9, 31.4, 21.8, 21.5.

HR-MS (ESI+): *m/z* for $C_{22}H_{20}N_2O_5S+Na^+$, $[M+Na]^+$ calculated: 447.0985; found: 447.0982.

7.3. 4-Ethoxycarbonyl-5-(2-nitrophenyl)oxazoles (20–25)

4-Ethoxycarbonyl-5-(5-methyl-nitrophenyl)oxazole (20)

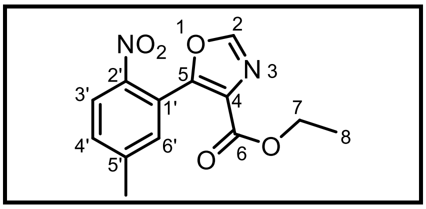

According to **GP2** 5-methyl-2-nitrobenzoic acid (4.53 g, 25 mmol, 1.0 eq.) and thionylchloride (2.7 mL, 38 mmol, 1.5 eq.) were reacted in 25 mL of chloroform at reflux.

The crude acid chloride, triethylamine (10.5 mL, 75 mmol, 3.0 eq.) and ethyl isocyanoacetate (2.7 mL, 25 mmol, 1.0 eq.) were reacted. 1.79 g (6 mmol, 26%) of the product were obtained as an off-white solid after silica flash column chromatography (cyclohexane:ethyl acetate; regular silica 120 g, particle size: 25 µm, SIHP) using 20 → 40% ethyl acetate.

^{1}H NMR (400 MHz, CD_3CN) *δ* [ppm]: 8.14 (s, 1H, *H*-2), 8.07 (d, *J* = 8.4 Hz, 1H, *H*-3'), 7.56 (ddd, *J* = 8.3 Hz, 1.9 Hz, 0.8 Hz, 1H, *H*-4'), 7.52 – 7.50 (m, 1H, *H*-6'), 4.15 (q, *J* = 7.1 Hz, 2H, *H*-7), 2.47 (s, 3H, *H*-1''), 1.12 (t, *J* = 7.1 Hz, 3H, *H*-8).
^{13}C NMR (101 MHz, CD_3CN) *δ* [ppm]:161.9, 152.7, 152.1, 147.2, 146.1, 133.9, 133.0, 129.2, 125.9, 123.3, 62.0, 21.3, 14.1.
HR-MS (ESI+): *m/z* for $C_{13}H_{12}N_2O_5$+Na^+, $[M+Na]^+$ calculated: 299.0638; found: 299.0636

4-Ethoxycarbonyl-5-(5-methoxy-2-nitrophenyl)oxazole (21)

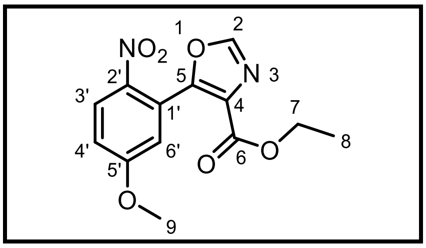

According to **GP2** 5-methoxy-2-nitrobenzoic acid (3.83 g, 19 mmol, 1.0 eq.) and thionylchloride (2.1 mL, 29 mmol, 1.5 eq.) were reacted in 20 mL of chloroform at reflux.

The crude acid chloride, triethylamine (8.1 mL, 58 mmol, 3.0 eq.) and ethyl isocyanoacetate (2.1 mL, 19 mmol, 1.0 eq.) were reacted. 1.86 g (6.4 mmol, 33%) of the product were obtained as an off-white solid after silica flash column chromatography (cyclohexane:ethyl acetate; regular silica 120 g, particle size: 25 µm, SIHP) using 20 → 40% ethyl acetate.

^{1}H NMR (400 MHz, CD_3CN) *δ* [ppm]: 8.23 (d, *J* = 9.1 Hz, 1H, *H*-3'), 8.17 (s, 1H, *H*-2), 7.27 – 7.19 (m, 2H, *H*-4', *H*-6'), 4.18 (q, *J* = 7.1 Hz, 2H, *H*-7), 3.94 (s, 3H), 1.15 (t, *J* = 7.2 Hz, 3H, *H*-8).
^{13}C NMR (101 MHz, CD_3CN) *δ* [ppm]: 164.1, 161.8, 152.6, 152.0, 142.3, 129.3, 128.5, 125.8, 119.0, 116.9, 61.9, 57.3, 14.1.
HR-MS (ESI+): *m/z* for $C_{13}H_{12}N_2O_6$+H^+, $[M+H]^+$ calculated: 315.0588; found: 315.0587.

4-Ethoxycarbonyl-5-(3,4,5-trimethoxy-2-nitrophenyl)oxazole (22)

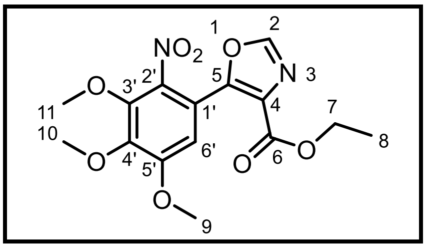

According to **GP2** 3,4,5-trimethoxy-2-nitrobenzoic acid (**8**, 5.00 g, 19 mmol, 1.0 eq.) and thionylchloride (2.1 mL, 29 mmol, 1.5 eq.) were reacted in 20 mL of chloroform at reflux.

The crude acid chloride, triethylamine (8.1 mL, 58 mmol, 3.0 eq.) and ethyl isocyanoacetate (2.1 mL, 19 mmol, 1.0 eq.) were reacted. 5.21 g (15 mmol, 76%) of the product were obtained as an off-white solid after silica flash column chromatography (cyclohexane:ethyl acetate; regular silica 120 g, particle size: 25 µm, SIHP) using 20 → 40% ethyl acetate.

^{1}H NMR (400 MHz, $CDCl_3$) *δ* [ppm]: 7.90 (s, 1H, *H*-2), 7.24 (s, 1H, *H*-6'), 4.36 (q, *J* = 7.1 Hz, 2H, *H*-7), 4.01 (s, 3H, *H*-11), 3.97 (s, 3H, *H*-10), 3.96 (s, 3H, *H*-9), 1.36 (t, *J* = 7.1 Hz, 3H, *H*-8).
^{13}C NMR (101 MHz, $CDCl_3$) *δ* [ppm]: 161.2, 154.8, 150.6, 150.2, 147.1, 144.7, 139.0, 129.1, 116.0, 109.9, 62.8, 61.8, 61.4, 56.7, 14.3.
HR-MS (ESI+): *m/z* for $C_{15}H_{16}N_2O_8$+Na^+, $[M+Na]^+$ calculated: 375.0799; found: 375.0800.

4-Ethoxycarbonyl-5-(4-trifluoromethyl-2-nitrophenyl)oxazole (23)

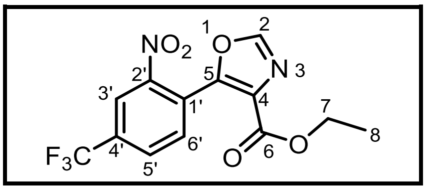

According to **GP2** 4-trifluoromethyl-2-nitrobenzoyl chloride (6.34 g, 25 mmol, 1.0 eq.), triethylamine (10.5 mL, 75 mmol, 3.0 eq.) and ethyl isocyanoacetate (2.7 mL, 25 mmol, 1.0 eq.) were reacted. 5.86 g (18 mmol, 71%) of the product were obtained as an off-white solid after silica flash column chromatography (cyclohexane:ethyl acetate; regular silica 120 g, particle size: 25 µm, SIHP) using 20 → 40% ethyl acetate.

^{1}H NMR (400 MHz, CD_3CN) δ [ppm]: 8.45 (dt, *J* = 1.9 Hz, 0.7 Hz, 1H, *H*-3'), 8.20 (s, 1H, *H*-2), 8.16 – 8.11 (m, 1H, *H*-5'), 7.95 (dt, *J* = 8.1 Hz, 0.7 Hz, 1H, *H*-6'), 4.17 (q, *J* = 7.1 Hz, 2H, *H*-7), 1.14 (t, *J* = 7.1 Hz, 3H, *H*-8).
^{13}C NMR (101 MHz, CD_3CN) δ [ppm]: 161.7, 152.8, 150.7, 149.6, 134.9, 133.7 (q, *J* = 34.3 Hz), 131.0 (q, *J* = 3.6 Hz), 130.2, 127.0, 123.8 (q, *J* = 272.4 Hz), 123.3 (q, *J* = 3.9 Hz).62.3, 14.1.
^{19}F NMR (376 MHz, CD_3CN) δ [ppm]: -63.7 (s).
HR-MS (ESI+): *m/z* for $C_{13}H_9F_3N_2O_5$+Na$^+$, [M+Na]$^+$ calculated: 353.0356; found: 353.0360.

4-Ethoxycarbonyl-5-(4-fluoro-nitrophenyl)oxazole (24)

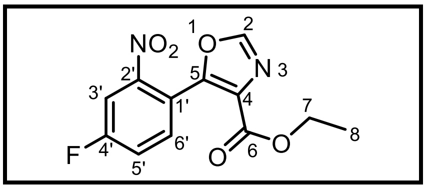

According to **GP2** 4-fluoro-2-nitrobenzoic acid (2.78 g, 15 mmol, 1.0 eq.) and oxalylchloride (1.9 mL, 23 mmol, 1.5 eq.) were reacted in 30 mL of dichloromethane at room temperature.
The crude acid chloride, triethylamine (6.3 mL, 45 mmol, 3.0 eq.) and ethyl isocyanoacetate (1.6 mL, 15 mmol, 1.0 eq.) were reacted. 2.70 g (10 mmol, 64%) of the product were obtained as an off-white solid after silica flash column chromatography (cyclohexane:ethyl acetate; regular silica 120 g, particle size: 25 µm, SIHP) using 20 → 40% ethyl acetate.

^{1}H NMR (400 MHz, $CDCl_3$) δ [ppm]: 7.98 (s, 1H, *H*-2), 7.88 (dd, *J* = 8.1 Hz, 2.6 Hz, 1H, *H*-3'), 7.68 (dd, *J* = 8.6 Hz, 5.3 Hz, 1H, *H*-6'), 7.45 (ddd, *J* = 8.6 Hz, 7.2 Hz, 2.6 Hz, 1H, *H*-5'), 4.27 (q, *J* = 7.1 Hz, 2H, *H*-7), 1.26 (t, *J* = 7.1 Hz, 3H, *H*-8).
^{13}C NMR (101 MHz, $CDCl_3$) δ [ppm]: 164.5, 162.0, 161.0, 150.7, 150.6, 149.3 (d, *J* = 8.6 Hz), 134.5 (d, *J* = 8.4 Hz), 128.9, 120.4 (d, *J* = 21.5 Hz), 118.5 (d, *J* = 4.0 Hz), 113.1 (d, *J* = 27.1 Hz), 61.7, 14.1.
^{19}F NMR (376 MHz, $CDCl_3$) δ [ppm]: -104.7 – -104.8 (m).
HR-MS (ESI+): *m/z* for $C_{12}H_9FN_2O_5$+Na$^+$, [M+Na]$^+$ calculated: 303.0388; found: 303.0381.

4-Ethoxycarbonyl-5-(2-nitrophenyl)oxazole (25)

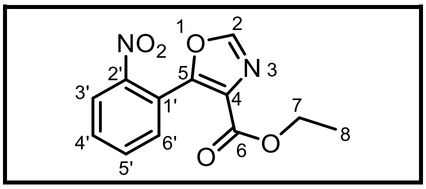

According to **GP2** 2-nitrobenzoyl chloride (5.00 g, 20 mmol, 1.0 eq.), triethylamine (8.0 mL, 60 mmol, 3.0 eq.) and ethyl isocyanoacetate (2.2 mL, 20 mmol, 1.0 eq.) were reacted. 3.00 g (11 mmol, 57%) of the product were obtained as an off-white solid after silica flash column chromatography (cyclohexane:ethyl acetate; regular silica 120 g, particle size: 25 µm, SIHP) using 20 → 40% ethyl acetate.

^{1}H NMR (400 MHz, CD_3CN) δ [ppm]: 8.17 (dd, *J* = 8.2 Hz, 1.3 Hz, 1H, *H*-6'), 8.15 (s, 1H, *H*-2), 7.83 (td, *J* = 7.6 Hz, 1.4 Hz, 1H, *H*-5'), 7.79 – 7.74 (m, 1H, *H*-4'), 7.72 (dd, *J* = 7.6 Hz, 1.5 Hz, 1H, *H*-3'), 4.15 (q, *J* = 7.1 Hz, 2H, *H*-7), 1.12 (t, *J* = 7.1 Hz, 3H, *H*-8).
^{13}C NMR (101 MHz, CD_3CN) δ [ppm]: 161.9, 152.4, 152.3, 149.4, 134.5, 133.5, 132.8, 129.4, 125.8, 123.3, 62.0, 14.1.
HR-MS (ESI+): *m/z* for $C_{12}H_{10}N_2O_5$+Na$^+$, [M+Na]$^+$ calculated: 285.0482; found: 285.0479.

7.4. 2-Amino-1-(2-nitrophenyl)alkan-1-one hydrochlorides (26-37)

Further purification of the 2-amino-1-(2-nitrophenyl)alkan-1-one hydrochlorides proofed to be challenging as the free base and extended heating during recrystallisation resulted in the formation of the corresponding pyrazines. The hydrochloride salts were therefore used without further purification.

2-Amino-1-(2-nitrophenyl)ethan-1-one hydrochlorides (26)

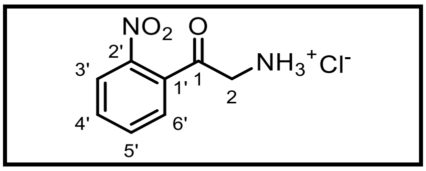

2-Bromo-2'-(2-nitrophenyl)ethenone (24.98 g, 0.1 mol, 1.0 eq.) was dissolved in 500 mL of dichloromethane using a mechanical stirrer. Hexamethylenetetramine (14.36 g, 0.1 mol, 1.0 eq.) was added in one portion and the reaction mixture was stirred at room temperature for 24 h. The solids were collected by filtration, washed three times with dichloromethane and dried using a vacuum. The crude salt was directly used without further purification.

The solids were transferred to a round bottom flask, 300 mL of ethanol and 40 mL of concentrated hydrochloric acid was added. The solids dissolved over time. The reaction mixture was stirred for 24 h resulting in precipitation of the product. The solids were filtered, washed three times with diethyl ether and dried using vacuum. 13.52 g (62 mmol, 62%) of the product was obtained as a beige solid.

2-Amino-1-(5-methyl-2-nitrophenyl)ethan-1-one hydrochlorides (27)

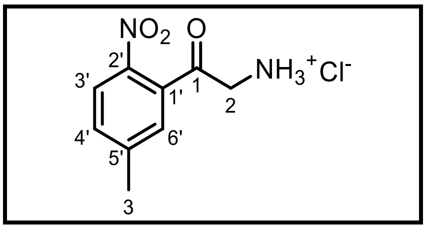

According to **GP4** 4-ethoxycarbonyl-5-(3-methyl-2-nitrophenyl)-oxazole (**20**, 1.50 g, 5.4 mmol, 1.0 eq.) was reacted in 25 mL of 6 M hydrochloric acid. 0.95 g (4.1 mmol, 75%) of the crude product were obtained as an off-white solid.

2-Amino-1-(5-methyl-2-nitrophenyl)ethan-1-one hydrochlorides (28)

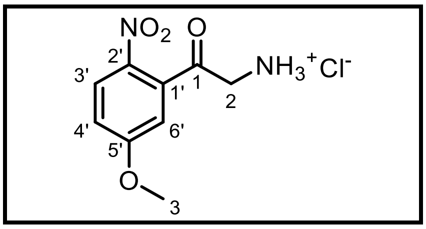

According to **GP4** 4-ethoxycarbonyl-5-(5-methoxy-2-nitrophenyl)-oxazole (**21**, 3.00 g, 10 mmol, 1.0 eq.) was reacted in 25 mL of 6 M hydrochloric acid for 16 h. 1.29 g (5.6 mmol, 18%) of the crude product were obtained as an off-white solid.

2-Amino-1-(2-nitro-3,4,5-trimethoxyphenyl)ethan-1-one hydrochloride (29)

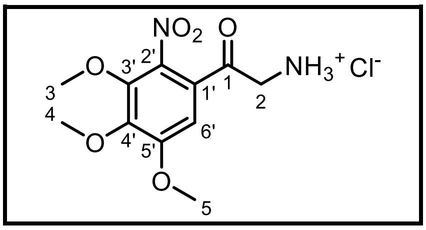

According to **GP4** 4-ethoxycarbonyl-5-(3,4,5-trimethoxy-2-nitrophenyl)-oxazole (**22**, 2.00 g, 5.7 mmol, 1.0 eq.) was reacted in 25 mL of 6 M hydrochloric acid. 0.70 g (2.2 mmol, 40%) of the crude product were obtained as an off-white solid.

2-Amino-1-(2-nitro-4-trifluoromethylphenyl)ethan-1-one hydrochloride (30)

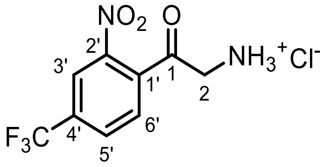

According to **GP4** 4-ethoxycarbonyl-5-(4-trifluoromethyl-2-nitrophenyl)oxazole (**23**, 2.00 g, 6.1 mmol, 1.0 eq.) was reacted in 25 mL of 6 M hydrochloric acid. 0.35 g (1.2 mmol, 20%) of the crude product was obtained as an off-white solid.

2-Amino-1-(4-fluoro-2-nitrophenyl)ethan-1-one hydrochloride (31)

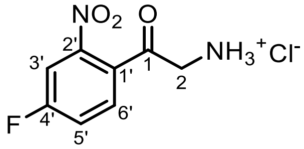

According to **GP4** 4-ethoxycarbonyl-5-(4-fluoro-2-nitrophenyl)-oxazole (**24**, 1.00 g, 3.6 mmol, 1.0 eq.) was reacted in 25 mL of 6 M hydrochloric acid. 0.34 g (1.4 mmol, 41%) of the crude product were obtained as an off-white solid.

2-Amino-1-(5-chloro-2-nitrophenyl)ethan-1-one hydrochloride (32)

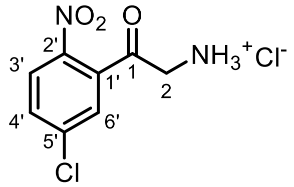

According to **GP3** 1-(5-chloro-2-nitrophenyl)ethan-1-one *O*-tosyl oxime (**15**, 0.79 g, 2.0 mmol, 1.0 eq.) and sodium ethoxide (0.28 g, 4.1 mmol, 2.0 eq.) were reacted in 20 mL of anhydrous ethanol.
0.49 g (1.9 mmol, 96%) of the crude product were obtained as an off-white solid.

2-Amino-1-(5-bromo-2-nitrophenyl)ethan-1-one hydrochloride (33)

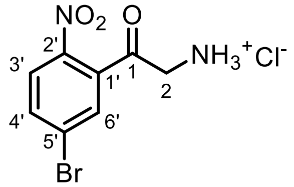

According to **GP3** 1-(5-bromo-2-nitrophenyl)ethan-1-one *O*-tosyl oxime (**16**, 2.00 g, 4.8 mmol, 1.0 eq.) and sodium ethoxide (0.66 g, 9.7 mmol, 2.0 eq.) were reacted in 50 mL of anhydrous ethanol.
0.60 g (2.0 mmol, 42%) of the crude product were obtained as an off-white solid.

2-Amino-1-(2-nitrophenyl)propan-1-one hydrochloride (34)

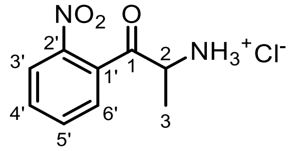

According to **GP3** 1-(2-nitrophenyl)propan-1-one *O*-tosyl oxime (**17**, 2.00 g, 5.7 mmol, 1.0 eq.) and sodium ethoxide (0.78 g, 11 mmol, 2.0 eq.) were reacted in 55 mL of anhydrous ethanol.
1.10 g (4.8 mmol, 83%) of the crude product were obtained as an off-white solid.

2-Amino-1-(2-nitrophenyl)-2-methylpropan-1-one hydrochloride (35)

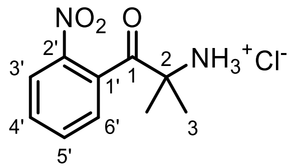

According to **GP3** 1-(2-nitrophenyl)-2-methylpropan-1-one *O*-tosyl oxime (**18**, 2.00 g, 5.5 mmol, 1.0 eq.) and potassium ethoxide (0.93 g, 11 mmol, 2.0 eq.) were reacted in 20 mL of anhydrous ethanol.
0.78 g (3.2 mmol, 58%) of the crude product were obtained as an off-white solid.

2-Amino-1-(2-nitrophenyl)-2-phenylethan-1-one hydrochloride (36)

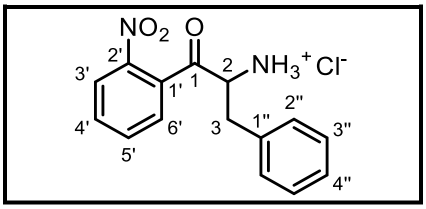

According to **GP3** 1-(2-nitrophenyl)-2-phenylethan-1-one *O*-tosyl oxime (**19**, 5.00 g, 12 mmol, 1.0 eq.) and potassium ethoxide (1.98 g, 14 mmol, 2.0 eq.) were reacted in 120 mL of anhydrous ethanol.
1.19 g (3.9 mmol, 33%) of the crude product were obtained as an off-white solid.

2-Amino-2-ethoxycarbonyl)1-(2-nitrophenyl)propan-1-one hydrochloride (37)

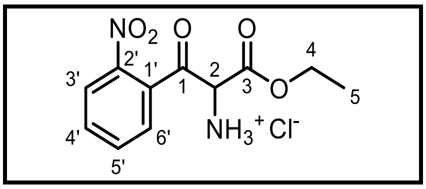

According to **GP4** 4-ethoxycarbonyl-5-(2-nitrophenyl)-oxazole (**25**, 2.00 g, 8 mmol, 1.0 eq.) was reacted in 25 mL of 3 M hydrochloric acid in methanol at 60 °C for 3 h. 1.51 g (6.0 mmol, 78%) of the crude product were obtained as an off-white solid.

3-Amino-1-(2-nitrophenyl)propan-1-one hydrochloride (38)

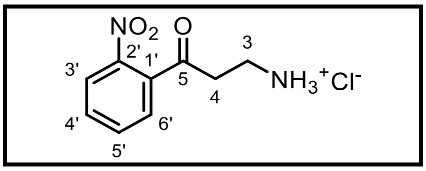

1-(2-Nitrophenyl)propen-1-one (**14**, 2.04 g, 12 mmol, 1.0 eq.) and potassium phthalimide (2.35 g, 13 mmol, 1.1 eq.) were dissolved in 100 mL of methanol. Triethylamine (3.1 mL, 23 mmol, 1.9 eq.) was added and the mixture was heated under reflux for 48 h. 50 mL of water was added and the methanol was removed under reduced pressure. The aqueous fraction was extracted three times with 50 mL of ethyl acetate. The collected organic fractions were washed twice with 50 mL of half-saturated sodium carbonate solution, once with 50 mL of water and once with 50 mL of brine. The organic fractions were dried over sodium sulphate and the solvent was removed under reduced pressure. 10 mL of glacial acetic acid and 20 mL of concentrated hydrochloric acid were added, and the suspension was heated under reflux for 72 h. The aqueous fraction was washed three times with 50 mL of ether and the solvent was removed under reduced pressure. 0.31 g (1.3 mmol, 11%) of the crude product were obtained as a beige solid.

7.5. *N*-(2-Nitrophenacyl)acylamides (4a–4ah, 6a, 6a')

N-(2-Nitrophenacyl)acetamide (4a)

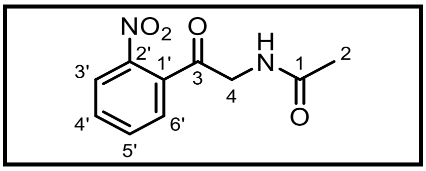

According to general protocol **GP5**, 2-amino-1-(2-nitrophenyl)ethan-1-one hydrochloride (**26**, 10.6 g, 49 mmol, 1.0 eq.), acetyl chloride (3.5 mL, 49 mmol, 1.0 eq.) and DIPEA (17.0 mL, 98 mmol, 2.0 eq.) were reacted in 100 mL of anhydrous dichloromethane 3.19 g (14 mmol, 34%) of the product was obtained as an off-white solid after silica flash column chromatography (cyclohexane:ethyl acetate; regular silica 80 g, particle size: 25 µm, SIHP) using 60 → 80% ethyl acetate.

^{1}H NMR (400 MHz, DMSO-[D6]) δ [ppm]: 8.42 (t, *J* = 5.6 Hz, 1H, 4-N*H*), 8.16 (dd, *J* = 8.2 Hz, 1.2 Hz, 1H, *H*-3'), 7.88 (td, *J* = 7.6 Hz, 1.2 Hz, 1H, *H*-5'), 7.78 (ddd, *J* = 8.1 Hz, 7.5 Hz, 1.5 Hz, 1H, *H*-4'), 7.70 (dd, *J* = 7.6 Hz, 1.5 Hz, 1H, *H*-6'), 4.35 (d, *J* = 5.7 Hz, 2H, *H*-4), 1.82 (s, 3H, *H*-2).
^{13}C NMR (101 MHz, DMSO-[D6]) δ [ppm]: 199.1, 169.8, 145.8, 134.5, 131.8, 128.4, 124.3, 48.1, 22.1.
HR-MS (ESI+): *m/z* for $C_{10}H_{10}N_2O_4$+Na$^+$, [M+Na]$^+$ calculated: 245.0533; found: 245.0528.

N-(2-Nitrophenacyl)propanamide (4b)

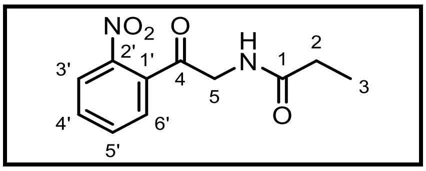

According to general protocol **GP5**, 2-amino-1-(2-nitrophenyl)ethan-1-one hydrochloride (**26**, 1.08 g, 5 mmol, 1.0 eq.), propionyl chloride (0.9 mL, 10 mmol, 2.0 eq.) and DIPEA (2.1 mL, 15 mmol, 3.0 eq.) were reacted in 50 mL of anhydrous THF. 0.233 g (1.0 mmol, 20%) of the product was obtained as an off-white solid after silica flash column chromatography (cyclohexane:ethyl acetate SIHP: 40 g regular silica column; particle size: 15 µm) using 10 → 60% ethyl acetate.

^{1}H NMR (400 MHz, CD_3CN) δ [ppm]: 8.13 (dd, *J* = 8.2 Hz, 1.2 Hz, 1H, *H*-3'), 7.81 (td, *J* = 7.6 Hz, 1.2 Hz, 1H, *H*-5'), 7.70 (ddd, *J* = 8.2 Hz, 7.5 Hz, 1.5 Hz, 1H, *H*-4'), 7.57 (dd, *J* = 7.6 Hz, 1.5 Hz, 1H, *H*-6'), 6.81 (s, 1H, 5-N*H*), 4.34 (d, *J* = 5.7 Hz, 2H, *H*-5), 2.12 (q, *J* = 7.6 Hz, 2H, *H*-2), 0.96 (t, *J* = 7.6 Hz, 3H, *H*-3).
^{13}C NMR (101 MHz, CD_3CN) δ [ppm]: 200.7, 175.0, 146.9, 136.4, 135.5, 132.3, 129.3, 125.2, 49.3, 29.5, 10.1.
HR-MS (ESI+): *m/z* for $C_{11}H_{12}N_2O_4$+H$^+$, [M+H]$^+$ calculated: 237.0870; found: 237.0875.

N-(2-Nitrophenacyl)isobutyramide (4c)

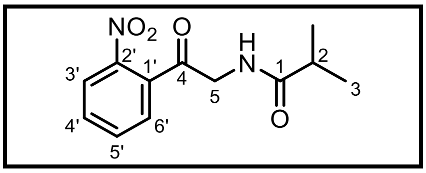

According to general protocol **GP5**, 2-amino-1-(2-nitrophenyl)ethan-1-one hydrochloride (**26**, 1.08 g, 5 mmol, 1.0 eq.), isobutyryl chloride (0.6 mL, 6 mmol, 1.2 eq.) and DIPEA (2.1 mL, 15 mmol, 3.0 eq.) were reacted in 50 mL of anhydrous THF. 0.532 g (2.1 mmol, 42%) of the product was obtained as an off-white solid after silica flash column chromatography (cyclohexane:ethyl acetate SIHC: 80 g regular silica column; particle size: 25 µm) using 10 → 60% ethyl acetate.

^{1}H NMR (400 MHz, CD_3CN) δ [ppm]: 8.12 (dd, *J* = 8.3 Hz, 1.2 Hz, 1H, *H*-3'), 7.79 (td, *J* = 7.5 Hz, 1.2 Hz, 1H, *H*-5'), 7.68 (ddd, *J* = 8.3 Hz, 7.5 Hz, 1.5 Hz, 1H, *H*-4'), 7.54 (dd, *J* = 7.6 Hz, 1.5 Hz, 1H, *H*-6'), 6.86 (s, 1H, 5-N*H*), 4.31 (d, *J* = 5.8 Hz, 2H, *H*-5), 2.33 (hept, *J* = 7.0 Hz, 1H, *H*-2), 0.93 (d, *J* = 7.0 Hz, 6H, *H*-3).
^{13}C NMR (101 MHz, CD_3CN) δ [ppm]: 201.1, 178.1, 146.9, 136.4, 135.4, 132.2, 129.4, 125.1, 49.2, 35.4, 19.7.
HR-MS (ESI+): *m/z* for $C_{12}H_{14}N_2O_4$+H$^+$, [M+H]$^+$ calculated: 251.1026; found: 251.1023.

N-(2-Nitrophenacyl)pivaloylamide (4d)

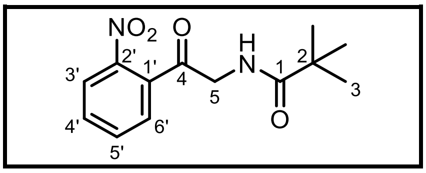

According to general protocol **GP5**, 2-amino-1-(2-nitrophenyl)ethan-1-one hydrochloride (**26**, 1.08 g, 5 mmol, 1.0 eq.), pivalic anhydride (1.1 mL, 6 mmol, 1.2 eq.) and DIPEA (2.5 mL, 15 mmol, 3.0 eq.) were reacted in 50 mL of anhydrous THF. 0.520 g (2.0 mmol, 40%) of the product was obtained as an slightly yellow oil after silica flash column chromatography (cyclohexane:ethyl acetate SIHC: 80 g regular silica column; particle size: 25 µm) using 5 → 50% ethyl acetate.

^{1}H NMR (400 MHz, CD_3CN) δ [ppm]: 8.11 (dd, *J* = 8.3 Hz, 1.2 Hz, 1H, *H*-3'), 7.79 (td, *J* = 7.5 Hz, 1.2 Hz, 1H, *H*-5'), 7.68 (ddd, *J* = 8.2 Hz, 7.5 Hz, 1.5 Hz, 1H, *H*-4'), 7.54 (dd, *J* = 7.6 Hz, 1.5 Hz, 1H, *H*-6'), 6.82 (s, 1H, 5-N*H*), 4.27 (d, *J* = 5.8 Hz, 2H, *H*-5), 1.00 (s, 9H, *H*-3).

^{13}C NMR (101 MHz, CD_3CN) δ [ppm]: 201.3, 179.5, 146.9, 136.5, 135.4, 132.1, 129.7, 125.0, 49.3, 39.1, 27.5.

HR-MS (ESI+): *m/z* for $C_{13}H_{16}N_2O_4$+H$^+$, [M+H]$^+$ calculated: 265.1183; found: 265.1184.

N-(2-Nitrophenacyl)heptanamide (4e)

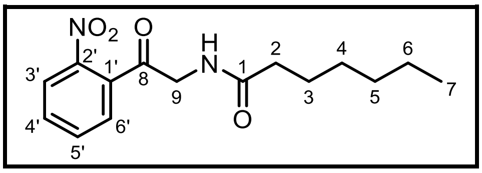

According to general protocol **GP5**, 2-amino-1-(2-nitrophenyl)ethan-1-one hydrochloride (**26**, 1.08 g, 5 mmol, 1.0 eq.), heptanoyl chloride (0.9 mL, 6 mmol, 1.2 eq.) and DIPEA (2.5 mL, 15 mmol, 3.0 eq.) were reacted in 50 mL of dichloromethane. 0.625 g (2.1 mmol, 43%) of the product was obtained as an off-white solid after silica flash column chromatography (cyclohexane:ethyl acetate; regular silica 40 g, particle size: 30 µm, SIHP) using 8 → 62% ethyl acetate.

^{1}H NMR (400 MHz, $CDCl_3$) δ [ppm]: 8.13 (dd, *J* = 8.2 Hz, 1.2 Hz, 1H, *H*-3'), 7.75 (td, *J* = 7.5 Hz, 1.2 Hz, 1H, *H*-5'), 7.63 (ddd, *J* = 8.2 Hz, 7.5 Hz, 1.5 Hz, 1H, *H*-4'), 7.53 (dd, *J* = 7.5 Hz, 1.5 Hz, 1H, *H*-6'), 6.40 (t, *J* = 5.3 Hz, 1H, 9-N*H*), 4.47 (d, *J* = 5.2 Hz, 2H, *H*-9), 2.26 – 2.17 (m, 2H. *H*-2), 1.62 – 1.49 (m, 2H, *H*-3), 1.35 – 1.20 (m, 6H, *H*-4, *H*-5, *H*-6), 0.92 – 0.81 (m, 3H, *H*-7).

^{13}C NMR (101 MHz, $CDCl_3$) δ [ppm]: 198.8, 173.8, 145.9, 135.7, 134.7, 131.2, 128.1, 124.4, 49.2, 36.4, 31.6, 29.0, 25.6, 22.6, 14.1.

HR-MS (ESI+): *m/z* for $C_{15}H_{20}N_2O_4$+H$^+$, [M+H]$^+$ calculated: 293.1496; found: 293.1494.

N-(2-Nitrophenacyl)cyclobutanecarboxamide (4f)

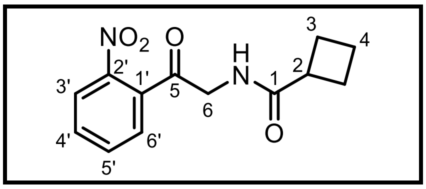

According to general protocol **GP5**, 2-amino-1-(2-nitrophenyl)ethan-1-one hydrochloride (**26**, 1.08 g, 5 mmol, 1.0 eq.), cyclobutylcarbonyl chloride (0.7 mL, 6 mmol, 1.2 eq.) and DIPEA (2.1 mL, 15 mmol, 3.0 eq.) were reacted in 50 mL of anhydrous THF. 0.565 g (2.2 mmol, 44%) of the product was obtained as an off-white solid after silica flash column chromatography (cyclohexane:ethyl acetate SIHP: 40 g regular silica column; particle size: 30 µm) using 10 → 60% ethyl acetate.

^{1}H NMR (400 MHz, CD_3CN) δ [ppm]: 8.10 (dd, *J* = 8.2 Hz, 1.2 Hz, 1H, *H*-3'), 7.79 (td, *J* = 7.5 Hz, 1.2 Hz, 1H, *H*-5'), 7.68 (ddd, *J* = 8.2 Hz, 7.5 Hz, 1.5 Hz, 1H, *H*-4'), 7.55 (dd, *J* = 7.6 Hz, 1.5 Hz, 1H, *H*-6'), 6.76 (s, 1H, 6-N*H*), 4.31 (d, *J* = 5.8 Hz, 1H, *H*-6), 3.05 – 2.92 (m, 1H, *H*-2), 2.07 – 1.95 (m, 4H, *H*-3), 1.93 – 1.82 (m, 1H, *H*-4), 1.78 – 1.67 (m, 1H, *H*-4).

^{13}C NMR (101 MHz, CD_3CN) δ [ppm]: 200.9, 176.0, 146.9, 136.4, 135.4, 132.2, 129.4, 125.1, 49.2, 39.9, 25.7, 18.8.

HR-MS (ESI+): *m/z* for $C_{13}H_{14}N_2O_4$+H$^+$, [M+H]$^+$ calculated: 263.1026; found: 263.1025.

N-(2-Nitrophenacyl)cyclohexanecarboxamide (4g)

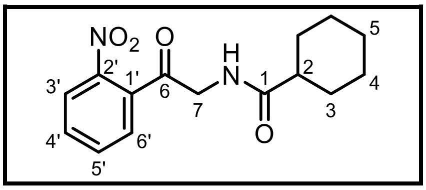

According to general protocol **GP5**, 2-amino-1-(2-nitrophenyl)ethan-1-one hydrochloride (**26**, 1.08 g, 5 mmol, 1.0 eq.), cyclohexanecarbonyl chloride (0.7 mL, 6 mmol, 1.2 eq.) and DIPEA (2.5 mL, 15 mmol, 3.0 eq.) were reacted in 50 mL of anhydrous THF. 0.667 g (2.3 mmol, 46%) of the product was obtained as an beige solid after silica flash column chromatography (cyclohexane:ethyl acetate SIHC: 80 g regular silica column; particle size: 25 µm) using 5 → 50% ethyl acetate.

^{1}H NMR (400 MHz, CD$_3$CN) δ [ppm]: 8.12 (dd, *J* = 8.3 Hz, 1.2 Hz, 1H, *H*-3'), 7.79 (td, *J* = 7.6 Hz, 1.2 Hz, 1H, *H*-5'), 7.68 (ddd, *J* = 8.3 Hz, 7.5 Hz, 1.5 Hz, 1H, *H*-4'), 7.54 (dd, *J* = 7.6 Hz, 1.5 Hz, 1H, *H*-6'), 6.78 (s, 1H, 7-N*H*), 4.30 (d, *J* = 5.8 Hz, 2H, *H*-7), 2.12 – 2.04 (m, 1H, *H*-2), 1.75 – 1.51 (m, 5H, *H*-3, *H*-4), 1.31 – 1.09 (m, 5H, *H*-3, *H*-4, *H*-5).

^{13}C NMR (101 MHz, CD$_3$CN) δ [ppm]: 201.2, 177.2, 146.9, 136.5, 135.4, 132.2, 129.5, 125.1, 49.2, 45.2, 30.2, 26.5, 26.3.

HR-MS (ESI+): *m/z* for $C_{15}H_{18}N_2O_4$+H$^+$, [M+H]$^+$ calculated: 291.1339; found: 291.1336.

3-Methyl-N-(2-(2-nitrophenyl)-2-oxoethyl)but-2-enamide (4h)

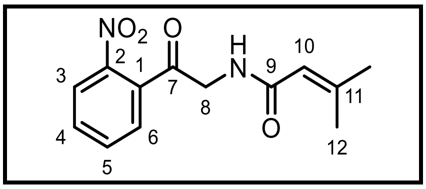

According to general protocol **GP5**, 2-amino-1-(2-nitrophenyl)ethan-1-one hydrochloride (**26**, 1.08 g, 5 mmol, 1.0 eq.), 3-methylbut-2-enoyl chloride (0.6 mL, 6 mmol, 1.2 eq.) and DIPEA (2.5 mL, 15 mmol, 3.0 eq.) were reacted in 50 mL of anhydrous THF. 0.541 g (2.1 mmol, 42%) of the product was obtained as an beige solid after silica flash column chromatography (cyclohexane:ethyl acetate SIHC: 80 g regular silica column; particle size: 25 µm) using 10 → 60% ethyl acetate.

^{1}H NMR (400 MHz, CD$_3$CN) δ [ppm]: 8.11 (dd, *J* = 8.2 Hz, 1.2 Hz, 1H, *H*-3'), 7.80 (td, *J* = 7.5 Hz, 1.2 Hz, 1H, *H*-5'), 7.69 (ddd, *J* = 8.3, 7.5, 1.5 Hz, 0H), 7.60 (dd, *J* = 7.6, 1.5 Hz, 0H), 6.76 (s, 0H), 5.67 – 5.63 (m, 0H), 4.38 (d, *J* = 5.7 Hz, 1H), 2.06 (d, *J* = 1.3 Hz, 1H), 1.81 (d, *J* = 1.4 Hz, 1H).

^{13}C NMR (101 MHz, CD$_3$CN) δ [ppm]: 200.6, 167.7, 152.6, 147.0, 136.4, 135.4, 132.3, 129.3, 125.2, 118.6, 49.2, 27.1, 19.8.

HR-MS (ESI+): *m/z* for $C_{13}H_{14}N_2O_4$+H$^+$, [M+H]$^+$ calculated: 263.1026; found: 263.1029.

N-(2-(2-Nitrophenyl)-2-oxoethyl)but-2-ynamide (4i)

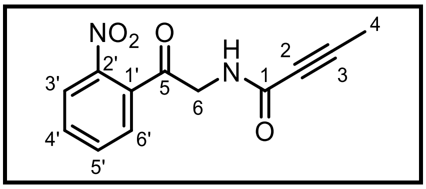

According to general protocol **GP5**, 2-amino-1-(2-nitrophenyl)ethan-1-one hydrochloride (**26**, 2.51 g, 12 mmol, 1.0 eq.), but-2-ynoyl chloride (1.1 mL, 13 mmol, 1.1 eq.) and DIPEA (5.8 mL, 35 mmol, 3.0 eq.) were reacted in 70 mL of anhydrous THF. 0.481 g (2.0 mmol, 17%) of the product was obtained as an yellow solid after silica flash column chromatography (cyclohexane:ethyl acetate SIHC: 80 g regular silica column; particle size: 25 µm) using 10 → 50% ethyl acetate

^{1}H NMR (400 MHz, CD$_3$CN) δ [ppm]: 8.13 (dd, *J* = 8.2 Hz, 1.2 Hz, 1H, *H*-3'), 7.81 (td, *J* = 7.5 Hz, 1.2 Hz, 1H, *H*-5'), 7.71 (ddd, *J* = 8.2 Hz, 7.5 Hz, 1.5 Hz, 1H, *H*-4'), 7.55 (dd, *J* = 7.6 Hz, 1.5 Hz, 1H, *H*-6'), 7.19 (s, 1H, 6-N*H*), 4.39 (d, *J* = 5.9 Hz, 2H, *H*-6), 1.93 (s, 3H, *H*-4).

^{13}C NMR (101 MHz, CD$_3$CN) δ [ppm]: 199.2, 154.2, 146.9, 136.0, 135.5, 132.5, 129.2, 125.4, 85.2, 74.9, 49.4, 3.5.

HR-MS (ESI+): *m/z* for $C_{12}H_{10}N_2O_4$+H$^+$, [M+H]$^+$ calculated: 247.0713; found: 247.0709.

N-(2-Nitrophenacyl)benzamide (4j)

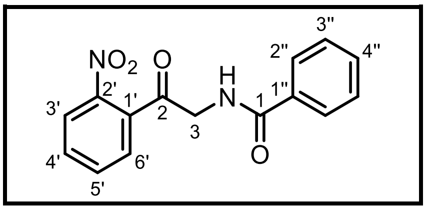

According to general protocol **GP5**, 2-amino-1-(2-nitrophenyl)ethan-1-one hydrochloride (**26**, 1.08 g, 5 mmol, 1.0 eq.), benzoyl chloride (0.6 mL, 6 mmol, 1.2 eq.) and DIPEA (2.5 mL, 15 mmol, 3.0 eq.) were reacted in 50 mL of anhydrous THF. 0.524 g (1.8 mmol, 36%) of the product was obtained as an off-white solid after silica flash column chromatography (cyclohexane:ethyl acetate SIHC: 80 g regular silica column; particle size: 25 µm) using 5 → 55% ethyl acetate.

^{1}H NMR (400 MHz, CD_3CN) δ [ppm]: 8.10 (dd, *J* = 8.2 Hz, 1.2 Hz, 1H, *H*-3'), 7.82 – 7.72 (m, 3H, *H*-5', *H*-2''), 7.71 – 7.58 (m, 3H, *H*-4', *H*-6', 3-N*H*), 7.56 – 7.49 (m, 1H, *H*-4''), 7.47 – 7.41 (m, 2H, *H*-3''), 4.57 (d, *J* = 5.7 Hz, 2H, *H*-3).
^{13}C NMR (101 MHz, CD_3CN) δ [ppm]: 200.2, 168.3, 146.9, 136.2, 135.4, 134.7, 132.7, 132.3, 129.5, 129.3, 128.0, 125.2, 49.8.
HR-MS (ESI+): *m/z* for $C_{15}H_{12}N_2O_4$+H^+, $[M+H]^+$ calculated: 285.0870; found: 285.0867.

N-(2-Nitrophenacyl)phenacylamide (4k)

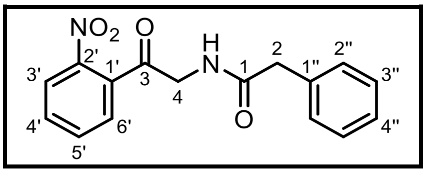

According to general protocol **GP5**, 2-amino-1-(2-nitrophenyl)ethan-1-one hydrochloride (**26**, 1.08 g, 5 mmol, 1.0 eq.), phenylacetyl chloride (0.7 mL, 6 mmol, 1.2 eq.) and DIPEA (2.5 mL, 15 mmol, 3.0 eq.) were reacted in 50 mL of anhydrous THF. 0.701 g (2.4 mmol, 47%) of the product was obtained as an beige solid after silica flash column chromatography (cyclohexane:ethyl acetate SIHC: 80 g regular silica column; particle size: 25 µm) using 5 → 50% ethyl acetate.

^{1}H NMR (400 MHz, CD_3CN) δ [ppm]: 8.08 (dd, *J* = 8.2 Hz, 1.2 Hz, 1H, *H*-3'), 7.74 (td, *J* = 7.5 Hz, 1.2 Hz, 1H, *H*-5'), 7.66 (ddd, *J* = 8.2 Hz, 7.5 Hz, 1.5 Hz, 1H, *H*-4'), 7.50 (dd, *J* = 7.5 Hz, 1.5 Hz, 1H, *H*-6'), 7.33 – 7.20 (m, 3H, *H*-2''), 7.18 – 7.13 (m, 2H, *H*-3'', *H*-4''), 6.93 (s, 1H, 4-N*H*), 4.34 (d, *J* = 5.7 Hz, 2H, *H*-4), 3.45 (s, 2H, *H*-2).
^{13}C NMR (101 MHz, CD_3CN) δ [ppm]: 200.5, 172.1, 146.8, 136.6, 136.2, 135.4, 132.3, 130.1, 129.5, 129.2, 127.7, 125.2, 49.5, 43.2.
HR-MS (ESI+): *m/z* for $C_{16}H_{14}N_2O_4$+H^+, $[M+H]^+$ calculated: 299.1026; found: 299.1025.

Ethyl 2-((2-(2-nitrophenyl)-2-oxoethyl)amino)-2-oxoacetate (4l)

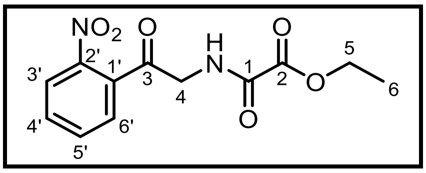

According to general protocol **GP5**, 2-amino-1-(2-nitrophenyl)ethan-1-one hydrochloride (**26**, 1.08 g, 5 mmol, 1.0 eq.), ethyl 2-chloro-2-oxoacetate (0.6 mL, 6 mmol, 1.2 eq.) and DIPEA (2.5 mL, 15 mmol, 3.0 eq.) were reacted in 50 mL of anhydrous THF. 0.618 g (2.2 mmol, 44%) of the product was obtained as an beige solid after silica flash column chromatography (cyclohexane:ethyl acetate SIHC: 80 g regular silica column; particle size: 25 µm) using 15 → 60% ethyl acetate.

^{1}H NMR (400 MHz, CD_3CN) δ [ppm]: 8.13 (dd, *J* = 8.2 Hz, 1.2 Hz, 1H, *H*-3'), 8.01 (s, 1H, 4-N*H*), 7.83 (td, *J* = 7.6 Hz, 1.2 Hz, 1H, *H*-5'), 7.72 (ddd, *J* = 8.2 Hz, 7.5 Hz, 1.5 Hz, 1H, *H*-4'), 7.60 (dd, *J* = 7.6 Hz, 1.5 Hz, 1H, *H*-6'), 4.51 (d, *J* = 5.9 Hz, 2H, *H*-4), 4.28 (q, *J* = 7.1 Hz, 2H, *H*-5), 1.31 (t, *J* = 7.1 Hz, 3H, *H*-6).
^{13}C NMR (101 MHz, CD_3CN) δ [ppm]: 198.2, 160.9, 158.1, 146.9, 135.5, 132.7, 129.1, 125.4, 63.8, 49.5, 14.2.
HR-MS (ESI+): *m/z* for $C_{12}H_{12}N_2O_6$+H^+, $[M+H]^+$ calculated: 281.0768; found: 281.0765.

Methyl (2-(2-nitrophenyl)-2-oxoethyl)carbamate (4m)

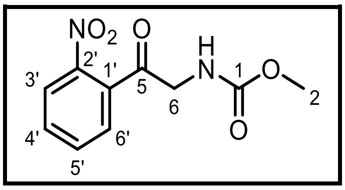

According to general protocol **GP5**, 2-amino-1-(2-nitrophenyl)ethan-1-one hydrochloride (**26**, 1.08 g, 5.0 mmol, 1.0 eq.), methyl chloroformate (0.47 g, 5.0 mmol, 1.0 eq.) and DIPEA (0.9 mL, 6.0 mmol, 1.1 eq.) were reacted in 75 mL of anhydrous dichloromethane. 0.45 g (1.9 mmol, 38%) of the product was obtained as an off-white solid after silica flash column chromatography (cyclohexane:ethyl acetate; regular silica 40 g, particle size: 15 µm, SIHP) using 60 → 80% ethyl acetate.

^{1}H NMR (400 MHz, CD_3CN) *δ* [ppm]: 8.16 – 8.10 (m, 1H, *H*-3'), 7.82 (td, *J* = 7.6 Hz, 1.2 Hz, 1H, *H*-5'), 7.71 (ddd, *J* = 8.2 Hz, 7.5 Hz, 1.5 Hz, 1H, *H*-4'), 7.58 – 7.53 (m, 1H, *H*-6'), 6.00 (s, 1H, 6-N*H*), 4.31 (d, *J* = 6.0 Hz, 2H, *H*-6), 3.59 (s, 3H, *H*-2).
^{13}C NMR (101 MHz, CD_3CN) *δ* [ppm]: 200.4, 158.1, 147.0, 136.1, 135.5, 132.4, 129.1, 125.3, 52.8, 50.9.
HR-MS (ESI+): *m/z* for $C_{10}H_{10}N_2O_5$+Na^+, $[M+Na]^+$ calculated: 261.0482; found: 261.0477.

***N*-(2-(2-Nitrophenyl)-2-oxoethyl)formamide (4n)**

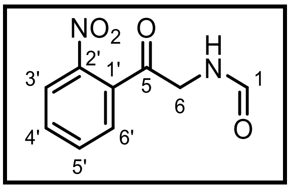

60 mL of acetic anhydride and 30 mL of formic acid were stirred at 0 °C for 1 h before sodium formate (0.91 g, 13 mmol, 1.3 eq.) was added and stirred for 1 h. Afterwards, 2-amino-1-(2-nitrophenyl)ethan-1-one hydrochloride (**26**, 2.17 g, 10 mmol, 1.0 eq.) was added in one portion at 0 °C and stirred for 1 h. The mixture was poured on 50 mL of water. The aqueous fraction was extracted three times with 50 mL of CH_2Cl_2. The combined organic fractions were washed with 100 mL of sodium carbonate solution and 100 mL of brine. Afterwards the organic extracts were dried over sodium sulphate and the solvent was removed. 0.68 g (3.3 mmol, 33%) of the product was obtained as an off-white solid after silica flash column chromatography (cyclohexane:ethyl acetate; regular silica 40 g, particle size: 15 µm, SIHP) using 40 → 80% ethyl acetate.

^{1}H NMR (400 MHz, CD_3CN) *δ* [ppm]: 8.14 (dd, *J* = 8.3 Hz, 1.2 Hz, 2H, *H*-3', *H*-1), 7.83 (td, *J* = 7.6 Hz, 1.2 Hz, 1H, *H*-5'), 7.72 (ddd, *J* = 8.3 Hz, 7.5 Hz, 1.5 Hz, 1H, *H*-4'), 7.59 (dd, *J* = 7.6 Hz, 1.5 Hz, 1H, *H*-6'), 6.97 (s, 1H, 6-N*H*), 4.45 (dd, *J* = 5.8 Hz, 0.7 Hz, 1H, 6-*H*).
^{13}C NMR (101 MHz, CD_3CN) *δ* [ppm]: 199.4, 162.3, 147.0, 136.0, 135.5, 132.5, 129.1, 125.4, 48.0.
HR-MS (ESI+): *m/z* for $C_9H_8N_2O_4$+Na^+, $[M+Na]^+$ calculated: 231.0376; found: 231.0374.

***tert*-Butyl (*S*)-(1-((2-(2-nitrophenyl)-2-oxoethyl)amino)-1-oxobutan-2-yl)carbamate (4o)**

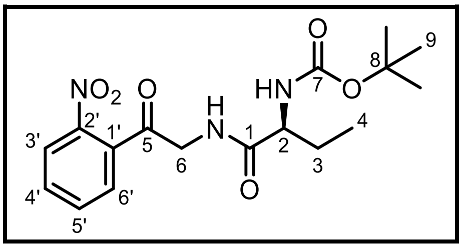

According to general protocol **GP6**, 2-amino-1-(2-nitrophenyl)ethan-1-one hydrochloride (**26**, 1.08 g, 5 mmol, 1.0 eq.), (*S*)-2-((tert-butoxycarbonyl)amino)butanoic acid (1.02 g, 5 mmol, 1.0 eq.), EDC·HCl (1.15 g, 6 mmol, 1.2 eq.), HOBt (0.92 g, 6 mmol, 1.2 eq.) and DIPEA (1.7 mL, 10 mmol, 2.0 eq.) were reacted in 50 mL of dichloromethane. 0.935 g (2.6 mmol, 51%) of the product was obtained as a highly viscous yellow oil after silica flash column chromatography (cyclohexane:ethyl acetate SIHC: 80 g regular silica column; particle size: 25 µm) using 5 → 55% ethyl acetate.

^{1}H NMR (400 MHz, CD_2Cl_2) *δ* [ppm]: 8.12 (dd, *J* = 8.2 Hz, 1.2 Hz, 1H, *H*-3'), 7.77 (td, *J* = 7.5 Hz, 1.2 Hz, 1H, *H*-5'), 7.65 (ddd, *J* = 8.2 Hz, 7.5 Hz, 1.5 Hz, 1H, *H*-4'), 7.54 (dd, *J* = 7.5 Hz, 1.5 Hz, 1H, *H*-6'), 7.06 (s, 1H. 6-N*H*), 5.16 (s, 1H, 2-N*H*), 4.54 – 4.39 (m, 2H, *H*-6), 4.06 (s, 1H, *H*-2), 1.77 (tq, *J* = 13.4 Hz, 6.6 Hz, 1H, *H*-3), 1.56 (dp, *J* = 13.4 Hz, 7.5 Hz, 1H, *H*-3), 1.43 (s, 9H, *H*-9), 0.89 (t, *J* = 7.4 Hz, 3H, *H*-4).
^{13}C NMR (101 MHz, CD_2Cl_2) *δ* [ppm]: 198.7, 173.0, 156.0, 146.2, 135.8, 134.9, 131.6, 128.4, 124.7, 80.2, 56.1, 49.3, 28.4, 26.1, 10.1.
HR-MS (ESI+): *m/z* for $C_{17}H_{23}N_3O_6$+Na^+, $[M+Na]^+$ calculated: 388.1479; found: 388.1469.

N-(2-Nitrophenacyl)difluoroacetamide (4p)

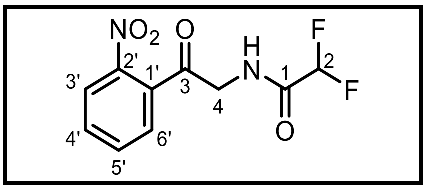

According to general protocol **GP5**, 2-amino-1-(2-nitrophenyl)ethan-1-one hydrochloride (**26**, 1.08 g, 5 mmol, 1.0 eq.), difluoroaceticacid anhydride (0.7 mL, 6 mmol, 1.2 eq.) and DIPEA (2.5 mL, 15 mmol, 3.0 eq.) were reacted in 50 mL of anhydrous THF. 0.540 g (2.1 mmol, 42%) of the product was obtained as an off-white solid after silica flash column chromatography (cyclohexane:ethyl acetate SIHC: 80 g regular silica column; particle size: 25 µm) using 10 → 62% ethyl acetate.

^{1}H NMR (400 MHz, CD_3CN) δ [ppm]: 8.15 (dd, *J* = 8.2 Hz, 1.2 Hz, 1H, *H*-3'), 7.84 (td, *J* = 7.5 Hz, 1.2 Hz, 1H, *H*-5'), 7.73 (ddd, *J* = 8.2 Hz, 7.5 Hz, 1.5 Hz, 1H, *H*-4'), 7.67 – 7.57 (m, 2H, *H*-6', 4-N*H*), 6.04 (t, *J* = 53.8 Hz, 1H, *H*-2), 4.50 (d, *J* = 5.8 Hz, 2H, *H*-4).

^{13}C NMR (101 MHz, CD_3CN) δ [ppm]: 198.2, 163.9, 163.7, 146.9, 135.6, 132.7, 129.2, 125.5, 109.5 (t, *J* = 248.1 Hz), 48.9.

^{19}F NMR (376 MHz, CD_3CN) δ [ppm]: -129.06 (d, *J* = 53.8 Hz).

HR-MS (ESI+): *m/z* for $C_{10}H_8F_2N_2O_4$+Na^+, $[M+Na]^+$ calculated: 281.0344; found: 281.0348.

3,3-Difluoro-N-(2-(2-nitrophenyl)-2-oxoethyl)cyclobutane-1-carboxamide (4q)

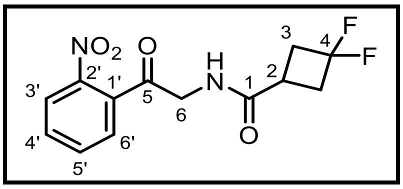

According to general protocol **GP6**, 2-amino-1-(2-nitrophenyl)ethan-1-one hydrochloride (**26**, 1.08 g, 5.0 mmol, 1.0 eq.), EDC·HCl (1.15 g, 6.0 mmol, 1.2 eq.), 3,3-difluorocyclobutane-1-carboxylic acid (0.68 g, 5.0 mmol, 1.0 eq.) and DIPEA (1.8 mL, 11 mmol, 2.1 eq.) were reacted in 75 mL of anhydrous dichloromethane. 0.390 g (1.3 mmol, 26%) of the product was obtained as an off-white solid after silica flash column chromatography (cyclohexane:ethyl acetate; regular silica 40 g, particle size: 15 µm, SIHP) using 60 → 80% ethyl acetate.

^{1}H NMR (400 MHz, CD_3CN) δ [ppm]: 8.13 (dd, *J* = 8.2 Hz, 1.2 Hz, 1H, *H*-3'), 7.81 (td, *J* = 7.5 Hz, 1.2 Hz, 1H, *H*-5'), 7.70 (ddd, *J* = 8.2 Hz, 7.5 Hz, 1.5 Hz, 1H, *H*-4'), 7.56 (dd, *J* = 7.5 Hz, 1.5 Hz, 1H, *H*-6'), 6.99 (s, 1H, 6-N*H*), 4.37 (d, *J* = 5.8 Hz, 2H, *H*-6), 2.93 – 2.81 (m, 1H, *H*-2), 2.73 – 2.51 (m, 4H, *H*-3).

^{13}C NMR (101 MHz, CD_3CN) δ [ppm]: 200.3, 173.8, 173.8, 173.7, 146.9, 136.2, 135.5, 132.4, 129.3, 125.2, 120.6 (dd, *J* = 283.5, 269.6 Hz), 49.5, 38.9 (t, *J* = 23.9 Hz), 27.6 (dd, *J* = 14.3, 5.4 Hz).

^{19}F NMR (377 MHz, CD_3CN) δ [ppm]: - -82.8 – -83.6 (m, 1F, 4-C*F*$_2$), -97.46 (dddd, *J* = 191.7 Hz, 32.0 Hz, 17.5 Hz, 14.1 Hz, 1F, 4-C*F*$_2$).

HR-MS (ESI+): *m/z* for $C_{13}H_{12}F_2N_2O_4$+Na^+, $[M+Na]^+$ calculated: 321.0657; found: 321.0650.

N-(2-(2-Nitrophenyl)-2-oxoethyl)bicyclo[1.1.1]pentane-1-carboxamide (4r)

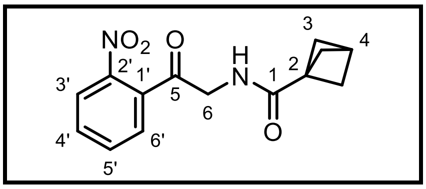

According to general protocol **GP6**, 2-amino-1-(2-nitrophenyl)ethan-1-one hydrochloride (**26**, 0.43 g, 2.0 mmol, 1.0 eq.), EDC·HCl (0.46 g, 2.4 mmol, 1.2 eq.), bicyclo[1.1.1]pentane-1-carboxylic acid (0.22 g, 2 mmol, 1.0 eq.) and DIPEA (0.7 mL, 4.2 mmol, 2.1 eq.) were reacted in 25 mL of anhydrous THF. 0.20 g (0.7 mmol, 37%) of the product was obtained as an off-white solid after silica flash column chromatography (cyclohexane:ethyl acetate; regular silica 40 g, particle size: 15 µm, SIHP) using 50 → 80% ethyl acetate.

^{1}H NMR (600 MHz, CD_3CN) δ [ppm]: 8.12 (dd, *J* = 8.3 Hz, 1.2 Hz, 1H, *H*-3'), 7.80 (td, *J* = 7.5 Hz, 1.1 Hz, 1H, *H*-5'), 7.69 (ddd, *J* = 8.2 Hz, 7.5 Hz, 1.4 Hz, 1H, *H*-4'), 7.53 (dd, *J* = 7.6 Hz, 1.4 Hz, 1H, *H*-6'), 6.84 (s, 1H, 6-N*H*), 4.29 (d, *J* = 5.9 Hz, 2H, *H*-6), 2.37 (s, 1H, *H*-4) 1.90 (s, 6H, *H*-3).

^{13}C NMR (151 MHz, CD_3CN) δ [ppm]: 200.5, 170.7, 146.8, 136.2, 135.4, 132.2, 129.4, 129.4, 125.1, 51.5, 48.9, 44.9, 27.6.

HR-MS (ESI+): *m/z* for $C_{14}H_{14}N_2O_4$+Na^+, $[M+Na]^+$ calculated: 297.0846; found: 297.0838.

N-(2-(2-Nitrophenyl)-2-oxoethyl)-3-(trifluoromethyl)bicyclo[1.1.1]pentane-1-carboxamide (4s)

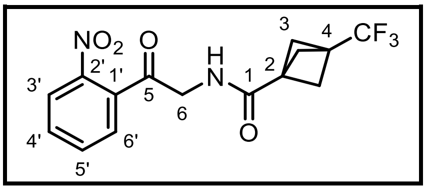

According to general protocol **GP6**, 2-amino-1-(2-nitrophenyl)ethan-1-one hydrochloride (**26**, 0.43 g, 2.0 mmol, 1.0 eq.), EDC·HCl (0.46 g, 2.4 mmol, 1.2 eq.), 3-(trifluoromethyl)bicyclo[1.1.1]pentane-1-carboxylic acid (0.36 g, 2.0 mmol, 1.0 eq.) and DIPEA (0.7 mL, 4.2 mmol, 2.1 eq.) were reacted in 25 mL of anhydrous dichloromethane. 0.210 g (0.6 mmol, 31%) of the product was obtained as an off-white solid after silica flash column chromatography (cyclohexane:ethyl acetate; regular silica 40 g, particle size: 15 µm, SIHP) using 50 → 70% ethyl acetate.

^{1}H NMR (600 MHz, $CDCl_3$) δ [ppm]: 8.17 (dd, *J* = 8.3 Hz, 1.1 Hz, 1H, *H*-3'), 7.78 (td, *J* = 7.5 Hz, 1.2 Hz, 1H, *H*-5'), 7.68 (ddd, *J* = 8.3 Hz, 7.5 Hz, 1.4 Hz, 1H, *H*-4'), 7.50 (dd, *J* = 7.6 Hz, 1.4 Hz, 1H, *H*-6'), 6.39 (t, *J* = 5.2 Hz, 1H, 6-N*H*), 4.48 (d, *J* = 5.2 Hz, 2H, *H*-6), 2.24 (s, 6H, *H*-3).
^{13}C NMR (151 MHz, $CDCl_3$) δ [ppm]: 197.8, 168.7, 145.9, 135.3, 134.8, 131.5, 128.0, 124.6, 122.5 (q, *J* = 275.8 Hz), 49.7 (q, *J* = 2.4 Hz), 49.1, 38.6, 36.7 (q, *J* = 39.3 Hz).
^{19}F NMR (282 MHz, $CDCl_3$) δ [ppm]: - 73.3 (s, 3F, 4-C*F*3).
HR-MS (ESI+): *m/z* for $C_{15}H_{14}F_3N_2O_4$+H^+, $[M+H]^+$ calculated: 343.0901; found: 343.0895.

Methyl 3-((2-(2-nitrophenyl)-2-oxoethyl)carbamoyl)bicyclo[1.1.1]pentane-1-carboxylate (4t)

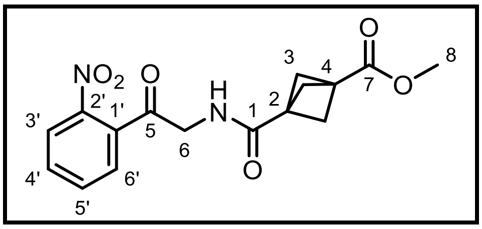

According to general protocol **GP6**, 2-amino-1-(2-nitrophenyl)ethan-1-one hydrochloride (**26**, 1.08 g, 5 mmol, 1.0 eq.), 3-(methoxycarbonyl)bicyclo[1.1.1]pentane-1-carboxylic acid (0.85 g, 5 mmol, 1.0 eq.), EDC·HCl (1.16 g, 6 mmol, 1.2 eq.) and DIPEA (1.7 mL, 10 mmol, 2.0 eq.) were reacted in 50 mL of dichloromethane. 0.854 g (2.6 mmol, 51%) of the product was obtained as a highly viscous yellow oil after silica flash column chromatography (cyclohexane:ethyl acetate SIHC: 80 g regular silica column; particle size: 25 µm) using 40 → 70% ethyl acetate.

^{1}H NMR (600 MHz, CD_3CN) δ [ppm]: 8.13 (dd, *J* = 8.3 Hz, 1.1 Hz, 1H, *H*-3'), 7.81 (td, *J* = 7.5 Hz, 1.1 Hz, 1H, *H*-5'), 7.70 (ddd, *J* = 8.8 Hz, 7.5 Hz, 1.4 Hz, 1H, *H*-4'), 7.53 (dd, *J* = 7.6 Hz, 1.4 Hz, 1H, *H*-6'), 6.95 (t, *J* = 5.7 Hz, 1H, 6-N*H*), 4.31 (d, *J* = 5.9 Hz, 2H, *H*-6), 3.62 (s, 3H, *H*-8), 2.12 (s, 6H, *H*-3).
^{13}C NMR (151 MHz, CD_3CN) δ [ppm]: 200.3, 170.5, 170.2, 146.8, 136.1, 135.5, 132.3, 129.3, 125.1, 52.7, 52.3, 48.9, 39.6, 37.6.
HR-MS (ESI+): *m/z* for $C_{16}H_{16}N_2O_6$+Na^+, $[M+Na]^+$ calculated: 355.0901; found: 355.0891.

Methyl 4-((2-(2-nitrophenyl)-2-oxoethyl)carbamoyl)cubane-1-carboxylate (4u)

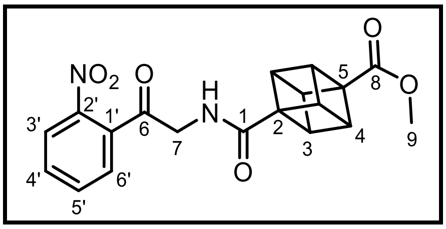

According to general protocol **GP6**, 2-amino-1-(2-nitrophenyl)ethan-1-one hydrochloride (**26**, 1.09 g, 5 mmol, 1.0 eq.), 4-(methoxycarbonyl)cubane-1-carboxylic acid (1.08 g, 5 mmol, 1.0 eq.), EDC·HCl (1.02 g, 6 mmol, 1.2 eq.) and DIPEA (2.0 mL, 11 mmol, 2.2 eq.) were reacted in 70 mL of THF. 0.777 g (2.1 mmol, 42%) of the product was obtained as a highly viscous oil after silica flash column chromatography (cyclohexane:ethyl acetate SIHC: 80 g regular silica column; particle size: 25 µm) using 5 → 50% ethyl acetate.

^{1}H NMR (400 MHz, CD_3CN) δ [ppm]: 8.14 – 8.09 (m, 1H, *H*-3'), 7.78 (tt, *J* = 7.6 Hz, 1.0 Hz, 1H, *H*-5'), 7.71 – 7.62 (m, 1H, *H*-4'), 7.55 (dd, *J* = 7.6 Hz, 1.4 Hz, 1H, *H*-6'), 6.61 (s, 1H, 7-N*H*), 4.49 – 4.46 (m, 2H, *H*-7), 4.20 – 4.11 (m, 6H, *H*-3, *H*-4), 3.67 (s, 3H, *H*-9).
^{13}C NMR (101 MHz, CD_3CN) δ [ppm]: 198.8, 172.1, 171.8, 146.2, 135.7, 134.9, 131.6, 128.4, 124.6, 57.9, 56.3, 51.8, 49.0, 47.4, 47.1.
HR-MS (ESI+): *m/z* for $C_{19}H_{16}N_2O_6$+H^+, $[M+H]^+$ calculated: 369.1081; found: 369.1077.

N-(2-Nitrophenacyl)pyrrolid-2-one (4v)

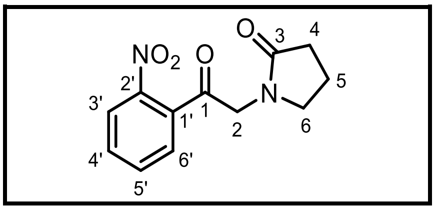

Under nitrogen atmosphere sodium hydride (60% dispersion in mineral oil, 0.44 g, 11 mmol, 1.1 eq.) was suspended in 10 mL of anhydrous THF and cooled to 0 °C. Pyrrolidin-2-one (0.91 mL, 12 mmol, 1.2 eq.) was added at 0 °C. The mixture was stirred for 1 h and was warmed to rt over 1 h. Afterwards, the mixture was again cooled to 0 °C, before 2-bromo-1-(2-nitrophenyl)ethanone (2.44 g, 10 mmol, 1.0 eq.) was added. The mixture was warmed to room temperature over 3 h and was stirred over night before 50 mL of water was added and the aquoeus fraction was extracted three times with 50 mL of ethyl acetate. The combined organic fractions were washed with brine and dried over sodium sulphate. The solvent was removed under reduced pressure. 0.20 g (0.8 mmol, 8%) of the product was obtained as a off-white solid after silica flash column chromatography (cyclohexane:ethyl acetate; regular silica 40 g, particle size: 15 µm, SIHP) using 40 → 80% ethyl acetate.

^{1}H NMR (400 MHz, CD_2Cl_2) δ [ppm]: 8.15 (dd, *J* = 8.3 Hz, 1.2 Hz, 1H, *H*-3’), 7.78 (td, *J* = 7.5 Hz, 1.2 Hz, 1H, *H*-4’), 7.66 (ddd, *J* = 8.3 Hz, 7.5 Hz, 1.5 Hz, 1H, *H*-5’), 7.53 (dd, *J* = 7.6 Hz, 1.5 Hz, 1H, *H*-6’), 4.43 (s, 2H, *H*-2), 3.57 – 3.45 (m, 2H, *H*-6), 2.35 (t, *J* = 8.1 Hz, 2H, *H*-4), 2.12 – 2.01 (m, 2H, *H*-5).
^{13}C NMR (101 MHz, CD_2Cl_2) δ [ppm]: 197.8, 176.0, 146.1, 135.9, 135.0, 131.5, 128.2, 124.8, 52.1, 47.8, 30.5, 18.5.
HR-MS (ESI+): *m/z* for $C_{12}H_{12}N_2O_4$+H^+, $[M+H]^+$ calculated: 249.0870; found: 249.0871.

N-(5-Methyl-2-nitrophenacyl)acetamide (4w)

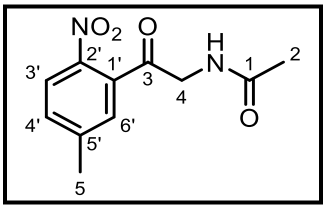

According to general protocol **GP5**, 2-amino-1-(5-methyl-2-nitrophenyl)ethan-1-one hydrochloride (**27**, 1.44 g, 6.2 mmol, 1.0 eq.), acetyl chloride (0.5 mL, 6.5 mmol, 1.1 eq.) and DIPEA (2.2 mL, 12 mmol, 2.0 eq.) were reacted in 60 mL of anhydrous dichloromethane. 0.22 g (0.9 mmol, 15%) of the product was obtained as an off-white solid after silica flash column chromatography (cyclohexane:ethyl acetate; regular silica 40 g, particle size: 15 µm, SIHP) using 60 → 80% ethyl acetate.

^{1}H NMR (400 MHz, $CDCl_3$) δ [ppm]: 8.05 (d, *J* = 8.4 Hz, 1H, *H*-3’), 7.41 (ddd, *J* = 8.5 Hz, 1.9 Hz, 0.8 Hz, 1H, *H*-4’), 7.26 (d, *J* = 1.4 Hz, 1H, *H*-6’), 6.43 (s, 1H, 4-N*H*), 4.46 (d, *J* = 5.2 Hz, 2H, *H*-4), 2.48 (s, 3H, *H*-5), 2.06 (s, 3H, *H*-2).
^{13}C NMR (101 MHz, $CDCl_3$) δ [ppm]: 198.7, 170.7, 146.7, 143.6, 135.8, 131.7, 128.2, 124.7, 49.5, 23.0, 21.7.
HR-MS (ESI+): *m/z* for $C_{11}H_{12}N_2O_4$+Na^+, $[M+Na]^+$ calculated: 259.0689; found: 259.0686.

N-(5-Methoxy-2-nitrophenacyl)acetamide (4x)

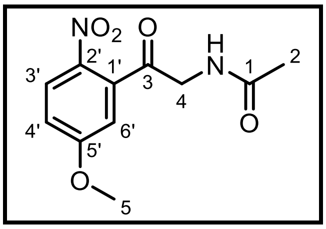

According to general protocol **GP5**, 2-amino-1-(5-methoxy-2-nitrophenyl)ethan-1-one hydrochloride (**28**, 1.20 g, 4.9 mmol, 1.0 eq.), acetyl chloride (0.3 mL, 4.9 mmol, 1.0 eq.) and DIPEA (1.7 mL, 9.7 mmol, 2.0 eq.) were reacted in 50 mL of anhydrous dichloromethane. 0.22 g (0.9 mmol, 18%) of the product was obtained as a off-white solid after silica flash column chromatography (cyclohexane:ethyl acetate; regular silica 40 g, particle size: 15 µm, SIHP) using 70 → 80% ethyl acetate.

^{1}H NMR (400 MHz, CD_3CN) δ [ppm]: 8.17 (d, *J* = 9.2 Hz, 1H, *H*-3’), 7.12 (dd, *J* = 9.2 Hz, 2.8 Hz, 1H, *H*-4’), 6.96 (d, *J* = 2.8 Hz, 1H, *H*-6’), 6.88 (s, 1H, 4-N*H*), 4.32 (d, *J* = 5.6 Hz, 2H, *H*-4), 3.91 (s, 3H, *H*-5), 1.87 (s, 3H, *H*-2).
^{13}C NMR (101 MHz, CD_3CN) δ [ppm]: 200.4, 171.2, 165.5, 139.7, 139.3, 128.0, 116.5, 113.8, 57.4, 49.6, 22.6.
HR-MS (ESI+): *m/z* for $C_{11}H_{12}N_2O_5$+Na^+, $[M+Na]^+$ calculated: 275.0638; found: 275.0632.

N-(2-Nitro-3,4,5-trimethoxyphenacyl)acetamide (4y)

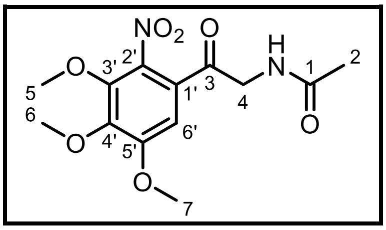

According to general protocol **GP5**, 2-amino-1-(2-nitro-3,4,5-trimethoxy-phenyl)ethan-1-one hydrochloride (**29**, 0.91 g, 3 mmol, 1.0 eq.), acetyl chloride (0.2 mL, 3 mmol, 1.0 eq.) and DIPEA (0.8 mL, 4 mmol, 1.3 eq.) were reacted in 50 mL of anhydrous dichloromethane. 0.311 g (1.0 mmol, 344%) of the product was obtained as a colourless solid after silica flash column chromatography (cyclohexane:ethyl acetate SIHC: 80 g regular silica column; particle size: 25 µm) using 30 → 60% ethyl acetate.

^{1}H NMR (400 MHz, CD_2Cl_2) δ [ppm]: 7.08 (s, 1H, *H*-6’), 6.35 (s, 1H, 4-N*H*), 4.62 (d, *J* = 4.8 Hz, 2H, *H*-4), 3.96 (s, 6H, *H*-5, *H*-7), 3.95 (s, 3H, *H*-6), 2.02 (s, 3H, *H*-2).
^{13}C NMR (101 MHz, CD_2Cl_2) δ [ppm]: 193.0, 170.4, 155.2, 147.2, 146.9, 138.8, 123.7, 107.2, 63.0, 61.6, 57.2, 47.6, 23.1.
HR-MS (ESI+): *m/z* for $C_{13}H_{16}N_2O_7$+H^+, $[M+H]^+$ calculated: 313.1030; found: 313.1028.

N-(2-Nitro-4-trifluoromethylphenacyl)acetamide (4z)

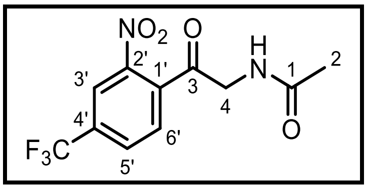

According to general protocol **GP5**, 2-amino-1-(4-trifluoromethyl-2-nitrophenyl)ethan-1-one hydrochloride (**30**, 0.35 g, 1.2 mmol, 1.0 eq.), acetyl chloride (0.1 mL, 1.2 mmol, 1.1 eq.) and DIPEA (0.4 mL, 2.4 mmol, 2.0 eq.) were reacted in 15 mL of anhydrous dichloromethane. 97 mg (0.3 mmol, 23%) of the product was obtained as a colourless solid after silica flash column chromatography (cyclohexane:ethyl acetate; regular silica 40 g, particle size: 15 µm, SIHP) using 40 → 80% ethyl acetate.

^{1}H NMR (400 MHz, CD_3CN) δ [ppm]: 8.45 – 8.44 (m, 1H, *H*-3‘), 8.12 (ddd, *J* = 8.0 Hz, 1.7 Hz, 0.7 Hz, 1H, *H*-5‘), 7.78 – 7.75 (m, 1H, *H*-6‘), 6.91 (s, 1H, 4-N*H*), 4.36 (d, *J* = 5.7 Hz, 2H, *H*-4), 1.85 (s, 3H, *H*-2).
^{13}C NMR (101 MHz, CD_3CN) δ [ppm]: 199.8, 171.3, 147.0, 140.0, 133.3 (q, *J* = 34.3 Hz), 132.3 (q, *J* = 3.6 Hz), 130.7, 123.8 (q, *J* = 272.1 Hz), 122.7 (q, *J* = 4.0 Hz), 49.5, 22.5.
^{19}F NMR (376 MHz, CD_3CN) δ [ppm]: -63.6.
HR-MS (ESI+): *m/z* for $C_{11}H_9F_3N_2O_4$+H^+, $[M+H]^+$ calculated: 291.0587; found: 291.0587.

N-(4-Fluoro-2-nitrophenacyl)acetamide (4aa)

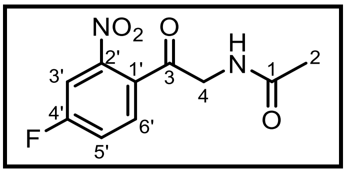

According to general protocol **GP5**, 2-amino-1-(4-fluoro-2-nitrophenyl)ethan-1-one hydrochloride (**31**, 0.34 g, 1.5 mmol, 1.0 eq.), acetyl chloride (0.1 mL, 1.5 mmol, 1.0 eq.) and DIPEA (0.5 mL, 2.9 mmol, 2.0 eq.) were reacted in 20 mL of anhydrous dichloromethane. 0.17 g (0.7 mmol, 48%) of the product was obtained as a off-white solid after silica flash column chromatography (cyclohexane:ethyl acetate; regular silica 40 g, particle size: 15 µm, SIHP) using 40 → 80% ethyl acetate.

^{1}H NMR (400 MHz, CD_3CN) δ [ppm]: 7.88 (dd, *J* = 8.6 Hz, 2.5 Hz, 1H, *H*-3’), 7.66 (dd, *J* = 8.6 Hz, 5.4 Hz, 1H, *H*-6’), 7.57 (ddd, *J* = 8.6 Hz, 7.9 Hz, 2.5 Hz, 1H, *H*-5’), 6.91 (s, 1H, 4-N*H*), 4.34 (d, *J* = 5.7 Hz, 2H, *H*-4), 1.87 (s, 3H, *H*-2).
^{13}C NMR (101 MHz, CD_3CN) δ [ppm]: 199.1, 171.4, 165.1, 162.6, 149.6 – 145.3 (m), 132.2 (d, *J* = 3.9 Hz), 131.7 (d, *J* = 8.9 Hz), 122.3 (d, *J* = 21.8 Hz), 113.1 (d, *J* = 27.4 Hz), 49.4, 22.5.
^{19}F NMR (376 MHz, CD_3CN) δ [ppm]: δ -107.97 – -108.05 (m).
HR-MS (ESI+): *m/z* for $C_{10}H_9FN_2O_4$+Na^+, $[M+Na]^+$ calculated: 263.0439; found: 263.0439.

N-(5-Chloro-2-nitrophenacyl)acetamide (4ab)

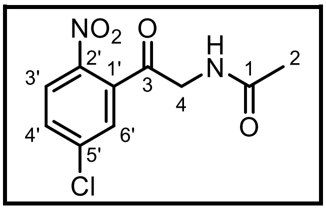

2-amino-1-(5-chloro-2-nitrophenyl)ethanone hydrochloride (**32**, 0.51 g, 2.0 mmol, 1.0 eq.) was dissolved in acetic anhydride (0.5 mL, 5.1 mmol, 2.5 eq.) and sodium acetate (0.33 g, 4.1 mmol, 2.0 eq.) was added. The mixture was stirred for 30 min at room temperature. Afterward 2 mL of water was added, and the mixture was stirred for 3 h. The liquid fraction was treated with saturated aqueous sodium bicarbonate solution, until neutral pH was reached. The aqueous fraction was extracted three times with 25 mL of ethyl acetate. The combined organic fractions were dried over sodium sulphate and the solvent was removed under reduced pressure. 0.25 g (1.0 mmol, 48%) of the product was obtained as an off-white solid after silica flash column chromatography (cyclohexane:ethyl acetate; regular silica 40 g, particle size: 15 µm, SIHP) using 60% ethyl acetate.

^{1}H NMR (400 MHz, CD_3CN) δ [ppm]: 8.13 (d, *J* = 8.8 Hz, 1H, *H*-3'), 7.69 (dd, *J* = 8.8 Hz, 2.3 Hz, 1H, *H*-4'), 7.57 (d, *J* = 2.3 Hz, 1H, *H*-6'), 6.89 (s, 1H, 4-N*H*), 4.33 (d, *J* = 5.7 Hz, 2H, *H*-4), 1.86 (s, 3H, *H*-2).
^{13}C NMR (101 MHz, CD_3CN) δ [ppm]: 199.2, 171.3, 145.3, 141.4, 138.2, 132.1, 129.2, 127.1, 49.5, 22.5.
HR-MS (ESI+): *m/z* for $C_{10}H_9{}^{35}ClNO_4+Na^+$, $[M+Na]^+$ calculated: 279.0143; found: 279.0142.

N-(5-Bromo-2-nitrophenacyl)acetamide (4ac)

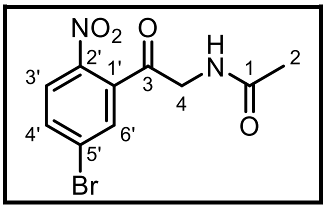

According to general protocol **GP5**, 2-amino-1-(5-bromo-2-nitrophenyl)ethan-1-one hydrochloride (**33**, 0.60 g, 2.0 mmol, 1.0 eq.), acetyl chloride (0.2 mL, 2.1 mmol, 1.1 eq.) and DIPEA (0.7 mL, 4.1 mmol, 2.0 eq.) were reacted in 20 mL of anhydrous dichloromethane. 0.13 g (0.5 mmol, 21%) of the product was obtained as an off-white solid after silica flash column chromatography (cyclohexane:ethyl acetate; regular silica 40 g, particle size: 15 µm, SIHP) using 60 → 80% ethyl acetate.

^{1}H NMR (400 MHz, CD_3CN) δ [ppm]: 8.04 (d, *J* = 8.7 Hz, 1H, *H*-3'), 7.78 (dd, *J* = 8.7 Hz, 2.0 Hz, 1H, *H*-4'), 7.66 (d, *J* = 2.1 Hz, 1H, *H*-6'), 6.27 (s, 1H, 4-N*H*), 4.46 (d, *J* = 5.1 Hz, 1H, *H*-4) 2.08 (s, 3H, *H*-2).
^{13}C NMR (101 MHz, $CDCl_3$) δ [ppm]: 196.9, 170.7, 144.6, 137.1, 134.4, 131.1, 130.3, 126.0, 49.4, 23.0.
HR-MS (ESI+): *m/z* for $C_{10}H_9{}^{79}BrNO_4+Na^+$, $[M+Na]^+$ calculated: 322.9638; found: 322.9629.

N-(1-(2-Nitrophenyl)-1-oxopropan-2-yl)acetamide (4ad)

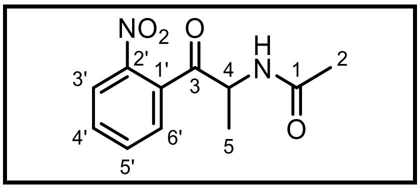

According to general protocol **GP6**, 2-amino-1-(2-nitrophenyl)propan-1-one hydrochloride (**34**, 0.46 g, 2.0 mmol, 1.0 eq.), acetic acid (0.1 mL, 2.0 mmol, 1.0 eq.), EDC HCl (0.46 mg, 2.4 mmol, 1.2 eq.) and DIPEA (0.7 mL, 4.2 mmol, 2.0 eq.) were reacted in 20 mL of anhydrous dichloromethane. 0.13 g (0.6 mmol, 28%) of the product was obtained as an slight yellow oil after silica flash column chromatography (cyclohexane:ethyl acetate; regular silica 40 g, particle size: 15 µm, SIHP) using 40 → 80% ethyl acetate.

^{1}H NMR (400 MHz, CD_3CN) δ [ppm]: 8.09 (dd, *J* = 8.2 Hz, 1.2 Hz, 1H, *H*-3'), 7.79 (td, *J* = 7.5 Hz, 1.2 Hz, 1H, *H*-5'), 7.68 (ddd, *J* = 8.2 Hz, 7.5 Hz, 1.5 Hz, 1H, *H*-4'), 7.62 (dd, *J* = 7.5 Hz, 1.5 Hz, 1H, *H*-6'), 6.81 (s, 1H, 4-N*H*), 5.01 (dq, *J* = 8.1 Hz, 7.2 Hz, 1H, *H*-4), 1.75 (s, 3H, *H*-2), 1.38 (d, *J* = 7.2 Hz, 3H, *H*-5).
^{13}C NMR (101 MHz, CD_3CN) δ [ppm]: 203.2, 170.4, 147.5, 136.4, 135.1, 132.2, 129.4, 125.2, 54.1, 22.6, 16.8.
HR-MS (ESI+): *m/z* for $C_{11}H_{12}N_2O_4+Na^+$, $[M+Na]^+$ calculated: 259.0689; found: 259.0683.

N-(2-Methyl-1-(2-nitrophenyl)-1-oxopropan-2-yl)acetamide (4ae)

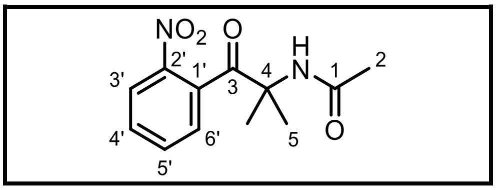

According to general protocol **GP5**, 2-amino-1-(2-nitrophenyl) 2-methylpropan-1-one hydrochloride (**35**, 0.78 g, 3.2 mmol, 1.0 eq.), acetyl chloride (0.2 mL, 3.2 mmol, 1.0 eq.) and DIPEA (1.1 mL, 6.4 mmol, 2.0 eq.) were reacted in 30 mL of anhydrous dichloromethane. 0.18 g (0.70 mmol, 22%) of the product was obtained as a sticky yellow oil after silica flash column chromatography (cyclohexane:ethyl acetate; regular silica 40 g, particle size: 15 µm, SIHP) using 50 → 70% ethyl acetate.

^{1}H NMR (600 MHz, CD_3CN) δ [ppm]: 8.03 (dt, J = 8.2 Hz, 1.1 Hz, 1H, H-3‘), 7.82 (dd, J = 7.8 Hz, 1.5 Hz, 1H, H-6‘), 7.74 (tt, J = 7.6 Hz, 1.1 Hz, 1H, H-5‘), 7.65 – 7.61 (m, 1H, H-4‘), 6.84 (s, 1H, 4-NH), 1.74 (s, 3H, H-2), 1.53 (s, 6H, H-5).
^{13}C NMR (151 MHz, CD_3CN) δ [ppm]: 205.1, 170.5, 147.5, 136.0, 134.5, 131.6, 130.2, 125.0, 62.6, 26.5, 23.1.
HR-MS (ESI+): m/z for $C_{12}H_{14}N_2O_4$+Na^+, $[M+Na]^+$ calculated: 273.0846; found: 273.0839.

N-(1-(2-Nitrophenyl)-1-oxo-3-phenylpropan-2-yl)acetamide (4af)

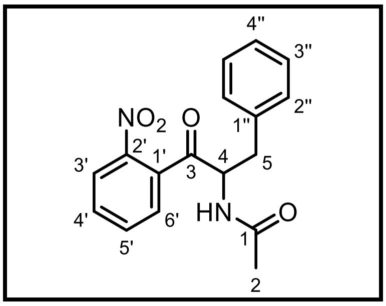

According to general protocol **GP3**, 2-amino-1-(2-nitrophenyl)-2-phenyl-ethan-1-one hydrochloride (**36**, 1.18 g, 3.8 mmol, 1.0 eq.), acetyl chloride (0.3 mL, 3.8 mmol, 1.0 eq.) and DIPEA (1.4 mL, 7.7 mmol, 2.0 eq.) were reacted in 40 mL of anhydrous dichloromethane. 0.32 g 10 mmol, 27%) of the product was obtained as a colourless oil after silica flash column chromatography (cyclohexane:ethyl acetate; regular silica 40 g, particle size: 15 µm, SIHP) using 40 → 60% ethyl acetate.

^{1}H NMR (400 MHz, CD_3CN) δ [ppm]: 8.10 (dd, J = 8.2 Hz, 1.2 Hz, 1H, H-3‘), 7.77 (td, J = 7.5 Hz, 1.2 Hz, 1H, H-5‘), 7.67 (ddd, J = 8.2 Hz, 7.5 Hz, 1.5 Hz, 1H, H-4‘), 7.57 (dd, J = 7.5 Hz, 1.5 Hz, 1H, H-6‘), 7.32 – 7.17 (m, 5H, H-2“, H-3“,H-4“), 6.79 (d, J = 9.0 Hz, 1H, 4-NH), 5.20 (ddd, J = 10.4 Hz, 9.0 Hz, 4.2 Hz, 1H, H-4), 3.41 (dd, J = 14.3 Hz, 4.2 Hz, 1H, H-5), 2.91 (dd, J = 14.3 Hz, 10.3 Hz, 1H, H-5), 1.60 (s, 3H, H-2).
^{13}C NMR (101 MHz, CD_3CN) δ [ppm]: 202.3, 170.5, 147.3, 138.8, 136.5, 135.2, 132.2, 130.1, 129.4, 129.3, 127.5, 125.2, 59.7, 37.0, 22.4.
HR-MS (ESI+): m/z for $C_{17}H_{16}N_2O_4$+H^+, $[M+H]^+$ calculated: 313.1183; found: 313,1169.

Ethyl 2-acetamido-3-(2-nitrophenyl)-3-oxopropanoate (4ag)

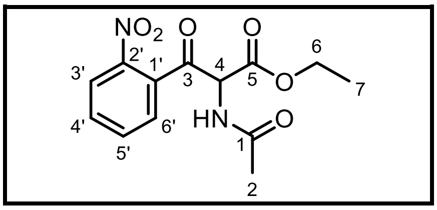

According to general protocol **GP5**, 2-amino-2-ethoxycarbonyl)1-(2-nitrophenyl)propan-1-one hydrochloride (**37**, 1.00 g, 4.0 mmol, 1.0 eq.), acetyl chloride (0.3 mL, 4.0 mmol, 1.0 eq.) and DIPEA (2.1 mL, 12 mmol, 3.0 eq.) were reacted in 40 mL of anhydrous dichloromethane. 0.63 g (2.1 mmol, 54%) of the product was obtained as a grey solid after reverse phase column chromatography (C_{18} silica gel, gradient: 35% → 45% acetonitrile)

Two rotamers are detected in the NMR spectra with a ratio of 88:12. The corresponding signals are partially overlapping. For the major rotamer the following signals were assigned.
^{1}H NMR (400 MHz, CD_3OD) δ [ppm]: 8.19 (dd, J = 8.2 Hz, 1.2 Hz, 1H, H-3‘), 7.86 (td, J = 7.5 Hz, 1.2 Hz, 1H, H-5‘), 7.77 (ddd, J = 8.2 Hz, 7.5 Hz, 1.5 Hz, 1H, H-4‘), 7.70 (dd, J = 7.6 Hz, 1.4 Hz, 1H, H-6‘), 4.09 (qd, J = 7.2 Hz, 4.9 Hz, 2H, H-6), 2.03 (s, 3H, H-2), 1.07 (t, J = 7.1 Hz, 3H, H-7).
^{13}C NMR (101 MHz, CD_3OD) δ [ppm]: 194.7, 173.2, 166.9, 147.5, 136.0, 135.5, 132.8, 129.7, 125.6, 125.4, 63.4, 22.0, 14.1.
HR-MS (ESI+): m/z for $C_{13}H_{14}N_2O_6$+Na^+, $[M+Na]^+$ calculated: 317.0744; found: 317.0734.

N-(3-(2-nitrophenyl)-3-oxopropyl)acetamide (4ah)

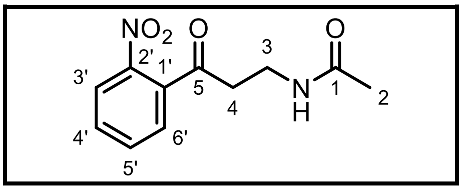

According to general protocol **GP5**, 3-amino-1-(2-nitrophenyl)propan-1-one hydrochloride (**38**, 0.305 g, 1.3 mmol, 1.0 eq.), acetyl chloride (0.1 mL, 1.6 mmol, 1.0 eq.) and DIPEA (0.5 mL, 2.6 mmol, 2.0 eq.) were reacted in 40 mL of anhydrous dichloromethane. 0.09 g (0.39 mmol, 30%) of the product was obtained as a colourless solid after silica flash column chromatography (cyclohexane:ethyl acetate; regular silica 40 g, particle size: 15 µm, SIHP) using 30 → 60% ethyl acetate.

^{1}H NMR (400 MHz, CD_2Cl_2) *δ* [ppm]: 8.09 (dd, *J* = 8.2 Hz, 1.2 Hz, 1H, *H*-3'), 7.75 (td, *J* = 7.5 Hz, 1.2 Hz, 1H, *H*-5'), 7.63 (ddd, *J* = 8.2 Hz, 7.5 Hz, 1.5 Hz, 1H, *H*-4'), 7.43 (dd, *J* = 7.5 Hz, 1.4 Hz, 1H, *H*-6'), 6.12 (s, 1H, 3-N*H*), 3.63 (q, *J* = 6.0 Hz, 2H, *H*-3), 3.02 (dd, *J* = 6.1, 5.4 Hz, 2H, *H*-4), 1.94 (s, 3H, *H*-2).
^{13}C NMR (101 MHz, CD_2Cl_2) *δ* [ppm]: 202.2, 170.5, 146.3, 137.7, 134.9, 131.4, 127.8, 125.0, 42.8, 34.7, 23.5.
HR-MS (ESI+): *m/z* for $C_{13}H_{14}N_2O_6$+Na^+, $[M+Na]^+$ calculated: 317.0744; found: 317.0734.

N-(2-Aminophenacyl)acetamide (6a)

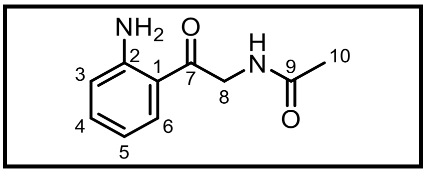

N-(2-Nitrophenacyl)acetamide (**4a**, 0.556 g, 2.5 mmol, 1.0 eq.) was dissolved in 50 mL of methanol. Platinum on carbon (5% *w/w*, 30 mg, 5 weight%) was added. The mixture was purged with argon and degassed under vacuum for three times. Afterwards a hydrogen ballon was added and the reaction mixture was stirred at room temperature. After completion of the reaction (LC-MS) the mixture was filtered using a Celite 545® plug. The solvent was removed under reduced pressure. 0.356 g (1.9 mmol, 76%) of the product was obtained as a yellow solid after silica flash column chromatography (cyclohexane:ethyl acetate; regular silica 40 g, particle size: 15 µm, SIHP) using 30 → 70% ethyl acetate.

^{1}H NMR (400 MHz, DMSO-[D6]) *δ* [ppm]: 8.12 (t, *J* = 5.7 Hz, 1H, 8-N*H*), 7.75 (dd, *J* = 8.2 Hz, 1.5 Hz, 1H, *H*-6), 7.26 (ddd, *J* = 8.5 Hz, 6.9 Hz, 1.5 Hz, 1H, *H*-4), 7.17 (s, 2H, 2-N*H*$_2$), 6.78 (dd, *J* = 8.5 Hz, 1.2 Hz, 1H, *H*-3), 6.54 (ddd, *J* = 8.2 Hz, 6.9 Hz, 1.2 Hz, 1H, *H*-5), 4.50 (d, *J* = 5.7 Hz, 2H, *H*-8), 1.92 (s, 3H, *H*-10).
^{13}C NMR (101 MHz, DMSO-[D6]) *δ* [ppm]: 196.8, 170.2, 151.5, 134.9, 130.8, 117.4, 115.3, 115.0, 46.1, 22.9.
HR-MS (ESI+): *m/z* for $C_{11}H_{12}N_2O_4$+H^+, $[M+H]^+$ calculated: 237.0870; found: 237.0864.

N-(2-Hydroxy-2-(2-nitrophenyl)ethyl)acetamide (6a')

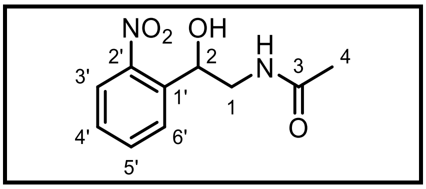

Under a nitrogen atmosphere *N*-(2-nitrophenacyl)acetamide (**4a**, 0.444 g, 2.0 mmol, 1.0 eq.) was dissolved in 30 mL of methanol and cooled to 0 °C. Sodium borohydride (38 mg, 1.0 mmol, 2.0 eq.) was added in one portion and was stirred 2 h at 0 °C. After completion of the reaction (TLC) 1 mL of glacial acetic acid and 20 mL of water were added. The methanol was removed under reduced pressure. The aqueous fraction was extracted three times with 20 mL ethyl acetate. The combined organic fractions were washed once with 20 mL of brine, dried over sodium sulphate and the solvent was removed under reduced pressure. 0.403 g (1.8 mmol, 90%) of the product was obtained as a colourless oil after silica flash column chromatography (cyclohexane:ethyl acetate; regular silica 25 g, particle size: 15 µm, SIHP) using 25 → 60% ethyl acetate.

^{1}H NMR (400 MHz, $CDCl_3$) *δ* [ppm]: 7.98 – 7.90 (m, 2H, *H*-3', *H*-6'), 7.70 – 7.63 (m, 1H, *H*-4'), 7.47 – 7.39 (m, 1H, *H*-5'), 6.32 (s, 1H, 1-N*H*), 5.35 (t, *J* = 4.6 Hz, 1H, *H*-2), 5.12 (s, 1H, 2-O*H*), 3.62 (m, 2H, *H*-1), 2.03 (s, 3H, H-*4*).
^{13}C NMR (101 MHz, $CDCl_3$) *δ* [ppm]: 173.5, 147.7, 137.8, 133.9, 128.9, 128.6, 124.7, 71.1, 48.1, 23.0.
HR-MS (ESI+): *m/z* for $C_{10}H_{12}N_2O_4$+H^+, $[M+H]^+$ calculated: 225.0870; found: 225.0862.

7.6. 3-(*N*-Acylaminoalkyl)-benzo[*c*]isoxazoles

3-(*N*-Acetamidomethyl)-benzo[*c*]isoxazole (5a)

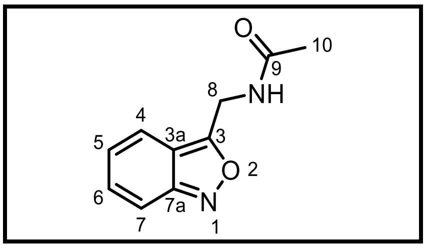

5 mL scale: According to general protocol **GP7**, *N*-(2-nitrophenacyl)acetamide (**4a**, 33.3 mg, 0.15 mmol, 1.0 eq.) was reacted. 23.0 mg (0.175 mmol, 81%) of the product were obtained as a colourless oil after reversed phase column chromatography (C18 silica gel 80 g, particle size: 30 µm, C18-HP) using 12 → 25% acetonitrile.

^{1}H NMR (400 MHz, DMSO-[D6]) δ [ppm]: 8.73 (t, *J* = 5.4 Hz, 1H, 8-N*H*), 7.70 (dt, *J* = 8.8 Hz, 1.1 Hz, 1H, *H*-4), 7.55 (dt, *J* = 9.1 Hz, 0.9 Hz, 1H, *H*-7), 7.37 (ddd, *J* = 9.1 Hz, 6.3 Hz, 1.0 Hz, 1H, *H*-6), 7.03 (ddd, *J* = 8.8 Hz, 6.3 Hz, 0.8 Hz, 1H, *H*-5), 4.81 (d, *J* = 5.9 Hz, 2H, *H*-8), 1.89 (s, 3H, *H*-10).
^{13}C NMR (101 MHz, DMSO-[D6]) δ [ppm]: 169.6, 166.4, 156.5, 131.5, 123.4, 120.6, 114.9, 114.2, 34.3, 22.3.
HR-MS (ESI+): *m/z* for $C_{10}H_{10}N_2O_2$+H$^+$, [M+H]$^+$ calculated: 191.0815; found: 191.0817.

3-(1-(*N*-Propanamido)-methyl)-benzo[*c*]isoxazole (5b)

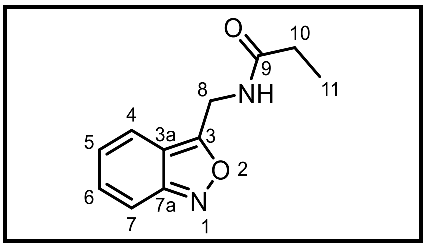

5 mL scale: According to general protocol **GP7**, *N*-(2-nitrophenacyl)propanamide (**4b**, 35.5 mg, 0.15 mmol, 1.0 eq.) was reacted. 21.2 mg (0.104 mmol, 69%) of the product were obtained as a pale yellow oil after silica flash column chromatography (cyclohexane:ethyl acetate; regular silica 25 g, particle size: 15 µm, SIHP) using 8 → 64% ethyl acetate.

^{1}H NMR (400 MHz, CD_2Cl_2) δ [ppm]: 7.60 (dt, *J* = 8.8, 1.1 Hz, 1H, *H*-4), 7.48 (dt, *J* = 9.1, 1.0 Hz, 1H, *H*-7), 7.28 (ddd, *J* = 9.1, 6.4, 1.0 Hz, 1H, *H*-6), 6.97 (ddd, *J* = 8.9, 6.4, 0.8 Hz, 1H, *H*-5), 6.44 (s, 1H, 8-N*H*), 4.90 (d, *J* = 6.0 Hz, 2H, *H*-8), 2.24 (q, *J* = 7.6 Hz, 2H, *H*-10), 1.11 (t, *J* = 7.6 Hz, 3H, *H*-11).
^{13}C NMR (101 MHz, CD_2Cl_2) δ [ppm]: 174.1, 165.2, 157.5, 131.5, 124.1, 120.4, 115.9, 115.0, 34.9, 29.6, 9.7.
HR-MS (ESI+): *m/z* for $C_{11}H_{12}N_2O_2$+H$^+$, [M+H]$^+$ calculated: 205.0972; found: 205.0965.

3-(1-(*N*-Isobutanamido)-methyl)-benzo[*c*]isoxazole (5c)

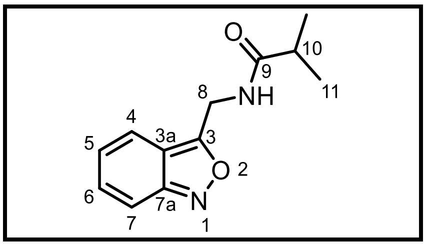

5 mL scale: According to general protocol **GP7**, *N*-(2-nitrophenacyl)isobutyramide (**4c**, 37.5 mg, 0.15 mmol, 1.0 eq.) was reacted. 19.0 mg (0.0874 mmol, 58%) of the product were obtained as a colourless oil after silica flash column chromatography (cyclohexane:ethyl acetate; regular silica 25 g, particle size: 15 µm, SIHP) using 8 → 64% ethyl acetate.

^{1}H NMR (400 MHz, CD_2Cl_2) δ [ppm]: 7.59 (d, *J* = 8.9 Hz, 1H, *H*-4), 7.52 – 7.45 (m, 1H, *H*-7), 7.28 (ddd, *J* = 9.2 Hz, 6.4 Hz, 1.0 Hz, 1H, *H*-6), 6.97 (ddd, *J* = 8.9 Hz, 6.4 Hz, 0.8 Hz, 1H, *H*-5), 6.46 (s, 1H, 8-N*H*), 4.90 (d, *J* = 6.0 Hz, 2H, *H*-8), 2.41 (hept, *J* = 6.9 Hz, 1H, *H*-10), 1.12 (d, *J* = 6.9 Hz, 6H, *H*-11).
^{13}C NMR (101 MHz, CD_2Cl_2) δ [ppm]: 177.3, 165.3, 157.6, 131.5, 124.1, 120.4, 115.4 (d, *J* = 84.7 Hz), 35.7, 35.0, 19.6.
HR-MS (ESI+): *m/z* for $C_{12}H_{14}N_2O_2$+H$^+$, [M+H]$^+$ calculated: 219.1128; found: 219.1123.

3-(*N*-Pivalamidomethyl)-benzo[*c*]isoxazole (5d)

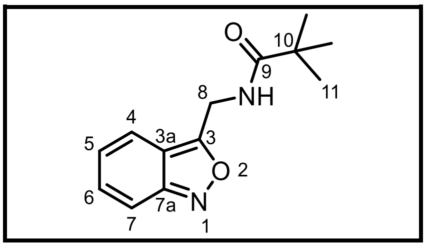

5 mL scale: According to general protocol **GP7**, *N*-(2-nitrophenacyl)pivaloylamide (**4d**, 39.6 mg, 0.15 mmol, 1.0 eq.), was reacted. 27.3 mg (0.118 mmol, 77%) of the product were obtained as a pale yellow solid after silica flash column chromatography (cyclohexane:ethyl acetate; regular silica 25 g, particle size: 30 µm, SIHP) using 4 → 38% ethyl acetate.

^{1}H NMR (400 MHz, CD_2Cl_2) δ [ppm]: 7.60 (dt, *J* = 8.8, 1.1 Hz, 1H, *H*-4), 7.49 (dt, *J* = 9.1, 1.0 Hz, 1H, *H*-7), 7.29 (ddd, *J* = 9.2, 6.4, 1.0 Hz, 1H, *H*-6), 6.98 (ddd, *J* = 8.9, 6.3, 0.8 Hz, 1H, *H*-5), 6.40 (s, 1H, 8-N*H*), 4.91 (d, *J* = 5.9 Hz, 2H, *H*-8), 1.19 (s, 9H, *H*-11).
^{13}C NMR (101 MHz, CD_2Cl_2) δ [ppm]: 178.7, 165.4, 157.6, 131.4, 124.1, 120.5, 115.9, 115.1, 39.1, 35.3, 27.6.
HR-MS (ESI+): *m/z* for $C_{13}H_{16}N_2O_2$+H^+, $[M+H]^+$ calculated: 233.1315; found: 233.1316.

3-(*N*-Heptanamidomethyl)-benzo[*c*]isoxazole (5e)

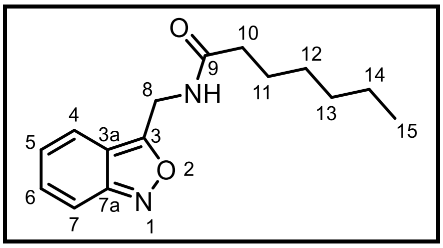

5 mL scale: According to general protocol **GP7**, *N*-(2-nitrophenacyl)heptanamide (**4e**, 43.8 mg, 0.15 mmol, 1.0 eq.) was reacted. 26.9 g (0.103 mmol, 69%) of the product were obtained as a pale yellow oil after silica flash column chromatography (cyclohexane:ethyl acetate; regular silica 25 g, particle size: 15 µm, SIHP) using 5 → 47% ethyl acetate.

^{1}H NMR (400 MHz, CD_2Cl_2) δ [ppm]: 7.61 (dt, *J* = 8.9, 1.1 Hz, 1H, *H*-4), 7.49 (dt, *J* = 9.1, 1.0 Hz, 1H, *H*-7), 7.29 (ddd, *J* = 9.1, 6.3, 1.0 Hz, 1H, *H*-6), 6.98 (ddd, *J* = 8.8, 6.4, 0.8 Hz, 1H, *H*-5), 6.23 (s, 1H, 8-N*H*), 4.91 (d, *J* = 6.1 Hz, 2H, *H*-8), 2.23 – 2.16 (m, 2H, *H*-10), 1.66 – 1.54 (m, 2H, *H*-11), 1.35 – 1.19 (m, 6H, *H*-12, *H*-13, *H*-14), 0.91 – 0.80 (m, 3H, *H*-15).
^{13}C NMR (101 MHz, CD_2Cl_2) δ [ppm]: 173.4, 165.2, 157.6, 131.5, 124.1, 120.5, 115.9, 115.1, 36.7, 34.9, 31.9, 29.3, 25.8, 22.9, 14.2.
HR-MS (ESI+): *m/z* for $C_{15}H_{20}N_2O_2$+H^+, $[M+H]^+$ calculated: 261.1598; found: 261.1592.

3-(*N*-Cyclobutanamidomethyl)-benzo[*c*]isoxazole (5f)

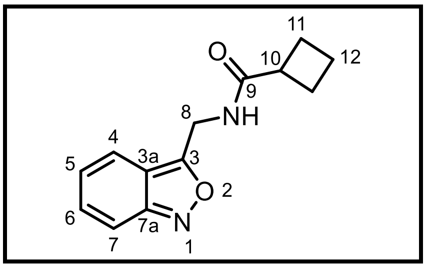

5 mL scale: According to general protocol **GP7**, *N*-(2-nitrophenacyl)cyclobutanecarboxamide (**4f**, 39.3 mg, 0.15 mmol, 1.0 eq.) was reacted. 24.9 mg (0.108 mmol, 72%) of the product was obtained as a colourless oil after silica flash column chromatography (cyclohexane:ethyl acetate; regular silica 25 g, particle size: 15 µm, SIHP) using 4 → 47% ethyl acetate.

^{1}H NMR (400 MHz, CD_2Cl_2) δ [ppm]: 7.60 (dt, *J* = 8.9 Hz, 1.1 Hz, 1H, *H*-4), 7.48 (dt, *J* = 9.1 Hz, 1.0 Hz, 1H, *H*-7), 7.28 (ddd, *J* = 9.1 Hz, 6.3 Hz, 1.0 Hz, 1H, *H*-6), 6.97 (ddd, *J* = 8.9 Hz, 6.3 Hz, 0.8 Hz, 1H, *H*-5), 6.29 (s, 1H, 8-N*H*), 4.90 (d, *J* = 6.0 Hz, 2H, *H*-8), 3.04 (pd, *J* = 8.5 Hz, 1.0 Hz, 1H, *H*-10), 2.30 – 2.18 (m, 2H, *H*-11), 2.17 – 2.07 (m, 2H, *H*-11), 2.01 – 1.91 (m, 1H, *H*-12), 1.89 – 1.80 (m, 1H, *H*-12).
^{13}C NMR (101 MHz, CD_2Cl_2) δ [ppm]: 175.3, 165.3, 157.5, 131.5, 124.1, 120.4, 115.9, 115.0, 40.0, 34.9, 25.6, 18.5.
HR-MS (ESI+): *m/z* for $C_{13}H_{14}N_2O_2$+H^+, $[M+H]^+$ calculated: 231.1128; found: 231.1126.

3-(*N*-Cyclohexanamidomethyl)-benzo[*c*]isoxazole (5g)

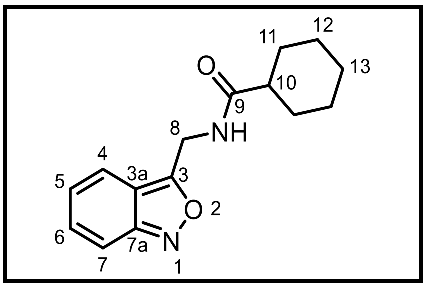

5 mL scale: According to general protocol **GP7**, *N*-(2-nitrophenacyl)cyclohexanecarboxamide (**4g**, 43.5 mg, 0.15 mmol, 1.0 eq.) was reacted. 26.0 mg (0.101 mmol, 67%) of the product were obtained as a colourless oil after silica flash column chromatography (cyclohexane:ethyl acetate; regular silica 25 g, particle size: 15 µm, SIHP) using 4 → 38% ethyl acetate.

^{1}H NMR (400 MHz, CD_3CN) δ [ppm]: 7.63 (dt, *J* = 8.9 Hz, 1.1 Hz, 1H, *H*-4), 7.48 (dt, *J* = 9.1 Hz, 1.0 Hz, 1H, *H*-7), 7.32 (ddd, *J* = 9.2 Hz, 6.4 Hz, 1.0 Hz, 1H, *H*-6), 7.10 (s, 1H, 8-N*H*), 6.98 (ddd, *J* = 8.9 Hz, 6.4 Hz, 0.8 Hz, 1H, *H*-5), 4.82 (d, *J* = 6.0 Hz, 2H, *H*-8), 2.20 – 2.10 (m, 1H, *H*-10), 1.79 – 1.67 (m, 4H, *H*-11, *H*-12), 1.66 – 1.58 (m, 1H, *H*-13), 1.42 – 1.12 (m, 5H, *H*-11, *H*-12, *H*-13).
^{13}C NMR (101 MHz, CD_3CN) δ [ppm]: 177.1, 167.1, 157.9, 132.2, 124.4, 121.3, 116.0, 115.2, 45.5, 35.4, 30.2, 26.5, 26.3.
HR-MS (ESI+): *m/z* for $C_{15}H_{18}N_2O_2+H^+$, $[M+H]^+$ calculated: 259.1441; found: 259.1429.

3-(*N*-(3,3-dimethylacryl)amidomethyl)-benzo[*c*]isoxazole (5h)

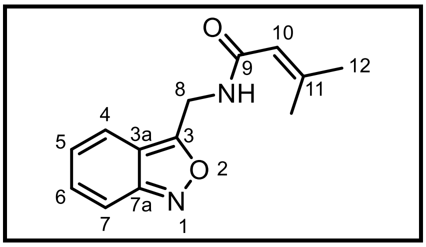

5 mL scale: According to general protocol **GP7**, 3-methyl-*N*-(2-(2-nitrophenyl)-2-oxoethyl)but-2-enamide (**4h**, 39.3 mg, 0.15 mmol, 1.0 eq.) was reacted. 18.1 mg (0.0786 mmol, 52%) of the product were obtained as a pale yellow oil after silica flash column chromatography (cyclohexane:ethyl acetate; regular silica 25 g, particle size: 15 µm, SIHP) using 4 → 38% ethyl acetate.

^{1}H NMR (400 MHz, CD_3CN) δ [ppm]: 7.64 (dt, *J* = 8.8 Hz, 1.1 Hz, 1H, *H*-4), 7.49 (dt, *J* = 9.1 Hz, 1.0 Hz, 1H, *H*-7), 7.32 (ddd, *J* = 9.1 Hz, 6.3 Hz, 1.0 Hz, 1H, *H*-6), 7.08 – 6.94 (m, 2H, *H*-5, 8-N*H*), 5.65 (p, *J* = 1.4 Hz, 1H, *H*-10), 4.86 (d, *J* = 6.1 Hz, 2H, *H*-8), 2.10 (d, *J* = 1.4 Hz, 3H, *H*-12), 1.82 (d, *J* = 1.4 Hz, 3H, *H*-12).
^{13}C NMR (101 MHz, CD_3CN) δ [ppm]: 167.7, 167.2, 158.0, 153.1, 132.2, 124.4, 121.4, 118.5, 116.1, 115.3, 35.2, 27.2, 19.8.
HR-MS (ESI+): *m/z* for $C_{13}H_{14}N_2O_2+H^+$, $[M+H]^+$ calculated: 231.1128; found: 231.1123.

3-(*N*-2-Butynamidomethyl)benzo[*c*]isoxazole (5i)

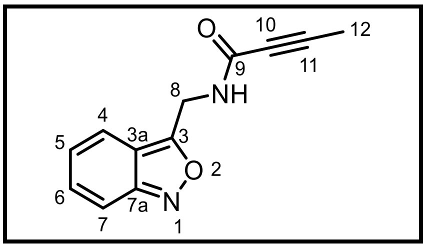

5 mL scale: According to general protocol **GP7**, *N*-(2-(2-nitrophenyl)-2-oxoethyl)but-2-ynamide (**4i**, 36.9 mg, 0.15 mmol, 1.0 eq.) was reacted. 20.0 mg (0.0934 mmol, 62%) of the product were obtained as a colourless oil after silica flash column chromatography (cyclohexane:ethyl acetate; regular silica 25 g, particle size: 15 µm, SIHP) using 8 → 64% ethyl acetate.

^{1}H NMR (400 MHz, CD_2Cl_2) δ [ppm]: 7.59 (d, *J* = 8.8 Hz, 1H, *H*-4), 7.50 (dd, *J* = 9.2 Hz, 1.0 Hz, 1H, *H*-7), 7.33 – 7.26 (m, 1H, *H*-6), 7.03 – 6.95 (m, 1H, *H*-5), 6.71 (s, 1H, 8-N*H*), 4.92 (d, *J* = 6.1 Hz, 3H, *H*-8), 1.93 (s, 3H, *H*-12).
^{13}C NMR (101 MHz, CD_2Cl_2) δ [ppm]: 164.1, 157.6, 153.6, 131.5, 124.4, 120.2, 116.0, 115.1, 85.2, 74.3, 35.1, 3.8.
HR-MS (ESI+): *m/z* for $C_{12}H_{10}N_2O_2+H^+$, $[M+H]^+$ calculated: 215.0815; found: 215.0810.

3-(*N*-Phenylamidomethyl)benzo[*c*]isoxazole (5j)

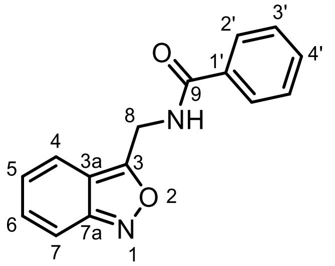

5 mL scale: According to general protocol **GP7**, *N*-(2-nitrophenacyl)benzamide (**4j**, 42.6 mg, 0.15 mmol, 1.0 eq.) was reacted. 22.1 mg (0.0876 mmol, 58%) of the product were obtained as a pale yellow solid after silica flash column chromatography (cyclohexane:ethyl acetate; regular silica 25 g, particle size: 15 µm, SIHP) using 4 → 38% ethyl acetate.

^{1}H NMR (400 MHz, CD_2Cl_2) δ [ppm]: 7.83 – 7.78 (m, 2H, *H*-4, *H*-4'), 7.65 (dt, *J* = 8.9, 1.1 Hz, 1H, *H*-7), 7.55 – 7.40 (m, 4H, *H*-2', *H*-3'), 7.28 (ddd, *J* = 9.1, 6.4, 1.0 Hz, 1H, *H*-6), 7.22 (s, 1H, 8-N*H*), 6.96 (ddd, *J* = 8.9, 6.4, 0.8 Hz, 1H, *H*-5), 5.11 (d, *J* = 6.0 Hz, 2H, *H*-8).
^{13}C NMR (101 MHz, CD_2Cl_2) δ [ppm]: 167.7, 165.0, 157.6, 134.0, 132.3, 131.5, 129.0, 127.5, 124.3, 120.5, 116.1, 115.0, 35.5.
HR-MS (ESI+): *m/z* for $C_{15}H_{12}N_2O_2$+H^+, $[M+H]^+$ calculated: 253.0972; found: 253.0972.

3-(*N*-Benzylamidomethyl)benzo[*c*]isoxazole (5k)

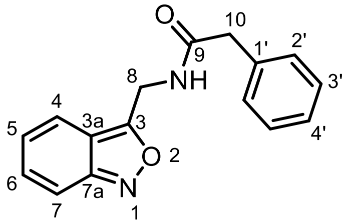

5 mL scale: According to general protocol **GP7**, *N*-(2-nitrophenacyl)phenacylamide (**4k**, 44.7 mg, 0.15 mmol, 1.0 eq.) was reacted. 24.1 mg (0.0905 mmol, 60%) of the product were obtained as an off-white solid after silica flash column chromatography (cyclohexane:ethyl acetate; regular silica 25 g, particle size: 15 µm, SIHP) using 4 → 38% ethyl acetate.

^{1}H NMR (400 MHz, CD_2Cl_2) δ [ppm]: 7.54 – 7.45 (m, 2H, *H*-4, *H*-7), 7.42 – 7.20 (m, 6H, *H*-6, *H*-2',*H*-3',*H*-4'), 6.94 (ddd, *J* = 8.8 Hz, 6.3 Hz, 0.8 Hz, 1H, *H*-5), 6.35 (s, 1H, 8-N*H*), 4.87 (d, *J* = 6.0 Hz, 2H, *H*-8), 3.58 (s, 2H, *H*-10).
^{13}C NMR (101 MHz, CD_2Cl_2) δ [ppm]: 171.2, 164.8, 157.5, 135.0, 131.4, 129.8, 129.3, 127.7, 124.1, 120.3, 115.9, 115.0, 43.6, 35.3.
HR-MS (ESI+): *m/z* for $C_{16}H_{14}N_2O_2$+H^+, $[M+H]^+$ calculated: 267.1128; found: 267.1121.

3-(*N*-(Ethoxy-2-oxoacet)amidomethyl)benzo[*c*]isoxazole (5l)

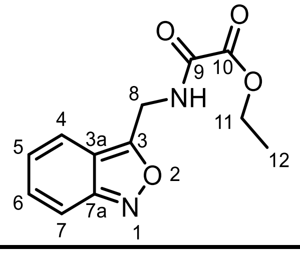

5 mL scale: According to general protocol **GP7**, ethyl 2-((2-(2-nitrophenyl)-2-oxoethyl)amino)-2-oxoacetate (**4l**, 42.0 mg, 0.15 mmol, 1.0 eq.) was reacted. 26.2 mg (0.106 mmol, 70%) of the product were obtained as a colourless oil after silica flash column chromatography (cyclohexane:ethyl acetate; regular silica 25 g, particle size: 15 µm, SIHP) using 4 → 47% ethyl acetate.

^{1}H NMR (400 MHz, CD_2Cl_2) δ [ppm]: 7.79 (s, 1H, 8-N*H*), 7.61 (dt, *J* = 8.8, 1.1 Hz, 1H, *H*-4), 7.52 (dt, *J* = 9.2, 1.0 Hz, 1H, *H*-7), 7.30 (ddd, *J* = 9.2, 6.4, 1.0 Hz, 1H, *H*-6), 7.01 (ddd, *J* = 8.9, 6.4, 0.8 Hz, 1H, *H*-5), 5.00 (d, *J* = 6.3 Hz, 2H, *H*-8), 4.31 (q, *J* = 7.1 Hz, 2H, *H*-11), 1.34 (t, *J* = 7.1 Hz, 3H, *H*-12).
^{13}C NMR (101 MHz, CD_2Cl_2) δ [ppm]: 163.2, 160.3, 157.6, 157.1, 131.5, 124.6, 120.0, 116.3, 115.2, 63.8, 35.1, 14.1.
HR-MS (ESI+): *m/z* for $C_{12}H_{12}N_2O_4$+H^+, $[M+H]^+$ calculated: 249.0870; found: 249.0857.

3-(*N*-(Methoxyform)amidomethyl)benzo[*c*]isoxazole (5m)

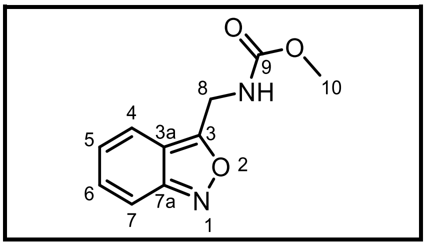

5 mL scale: According to general protocol **GP7**, methyl (2-(2-nitrophenyl)-2-oxoethyl)carbamate (**4m**, 35.7 mg, 0.15 mmol, 1.0 eq.) was reacted. 18.0 mg (0.0873 mmol, 58%) of the product were obtained as a colourless oil after silica flash column chromatography (cyclohexane:ethyl acetate; regular silica 25 g, particle size: 15 µm, SIHP) using 4 → 47% ethyl acetate.

^{1}H NMR (400 MHz, CD_2Cl_2) δ [ppm]: 7.61 (d, *J* = 8.8 Hz, 1H, *H*-4), 7.52 – 7.48 (m, 1H, *H*-7), 7.29 (ddd, *J* = 9.1 Hz, 6.3 Hz, 1.0 Hz, 1H, *H*-6), 6.99 (ddd, *J* = 8.8 Hz, 6.3 Hz, 0.8 Hz, 1H, *H*-5), 5.54 (s, 1H, 8-N*H*), 4.84 (d, *J* = 6.3 Hz, 2H, *H*-8), 3.67 (s, 3H, *H*-10).
^{13}C NMR (101 MHz, CD_2Cl_2) δ [ppm]: 165.1, 157.5, 157.3, 131.4, 124.2, 120.3, 115.9, 115.1, 52.9, 36.9.
HR-MS (ESI+): *m/z* for $C_{10}H_{10}N_2O_3$+H$^+$, [M+H]$^+$ calculated: 207.0764; found: 207.0753.

3-(*N*-Formamidomethyl)benzo[*c*]isoxazole (5n)

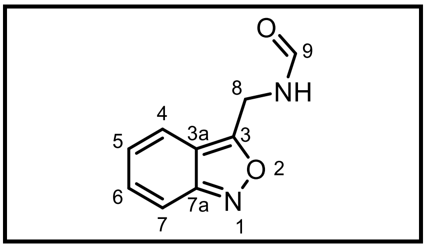

5 mL scale: According to general protocol **GP7**, *N*-(2-(2-nitrophenyl)-2-oxoethyl)formamide (**4n**, 31.2 mg, 0.15 mmol, 1.0 eq.) was reacted. 8.1 mg (0.0460 mmol, 30%) of the product were obtained as a colourless oil after silica flash column chromatography (cyclohexane:ethyl acetate; regular silica 25 g, particle size: 15 µm, SIHP) using 9 → 94% ethyl acetate.

^{1}H NMR (600 MHz, CD_2Cl_2) δ [ppm]: 8.27 (s, 1H, *H*-9), 7.62 (d, *J* = 8.8 Hz, 1H, *H*-4), 7.52 – 7.49 (m, 1H, *H*-7), 7.31 (ddd, *J* = 9.1 Hz, 6.3 Hz, 1.0 Hz, 1H, *H*-6), 7.00 (ddd, *J* = 8.8 Hz, 6.3 Hz, 0.8 Hz, 1H, *H*-5), 6.34 (s, 1H, 8-N*H*), 4.96 (d, *J* = 6.3 , Hz, 2H, *H*-8).
^{13}C NMR (151 MHz, CD_2Cl_2) δ [ppm]: 164.1, 161.3 (d, *J* = 2.3 Hz), 157.6, 131.5, 124.4, 120.1, 115.9, 115.1, 33.4.
HR-MS (ESI+): *m/z* for $C_9H_8N_2O_2$+H$^+$, [M+H]$^+$ calculated: 177.0659; found: 177.0656.

3-(*N*-(2-Aminobutan)amidomethyl)benzo[*c*]isoxazole (5o)

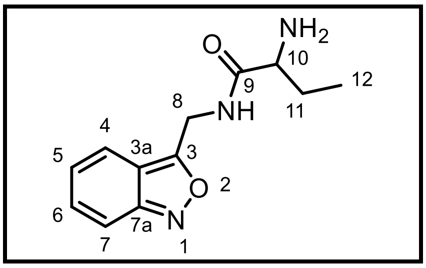

5 mL scale: According to general protocol **GP7**, *tert*-butyl (*S*)-(1-((2-(2-nitrophenyl)-2-oxoethyl)amino)-1-oxobutan-2-yl)carbamate (**4o**, 54.8 mg, 0.15 mmol, 1.0 eq.) was reacted and then stirred overnight. 24.9 mg (0.107 mmol, 71%) of the product were obtained as a yellow oil after reversed phase column chromatography (C18 silica gel 80 g, particle size: 30 µm, C18-HP) using 7 → 15% acetonitrile.

^{1}H NMR (400 MHz, DMSO-[D6]) δ [ppm]: 9.24 (s, 1H, 8-N*H*), 8.32 (s, 2H, 8-N*H*$_2$), 7.76 (d, *J* = 8.6 Hz, 1H, *H*-4), 7.55 (d, *J* = 9.0 Hz, 1H, *H*-7), 7.37 (dd, *J* = 9.0 Hz, 6.1 Hz, 1H, *H*-6), 7.08 – 6.99 (m, 1H, *H*-5), 4.90 (s, 2H, *H*-8), 3.59 (s, 1H, *H*-10), 1.66 (s, 2H, *H*-11), 0.81 (t, *J* = 6.2 Hz, 3H, *H*-12).
^{13}C NMR (101 MHz, DMSO-[D6]) δ [ppm]: 171.0, 165.7, 156.5, 131.5, 123.5, 120.7, 115.1, 114.2, 54.0, 34.2, 25.4, 9.2.
HR-MS (ESI+): *m/z* for $C_{12}H_{15}N_3O_2$+H$^+$, [M+H]$^+$ calculated: 234.1237; found: 234.1231.

3-(*N*-(2,2-Difluoroacet)amidomethyl)benzo[*c*]isoxazole (5p)

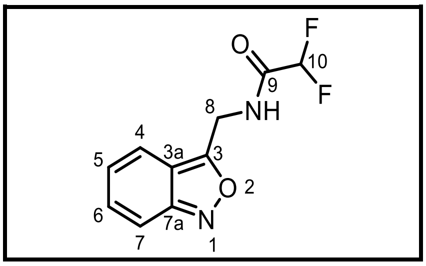

5 mL scale: According to general protocol **GP7**, *N*-(2-nitrophenacyl)difluoroacetamide (**4p**, 38.7 mg, 0.15 mmol, 1.0 eq.) was reacted. 23.9 mg (0.106 mmol, 71%) of the product were obtained as an off-white solid after silica flash column chromatography (cyclohexane:ethyl acetate; regular silica 25 g, particle size: 15 µm, SIHP) using 8 → 64% ethyl acetate.

^{1}H NMR (400 MHz, CD_3CN) δ [ppm]: 7.94 (s, 1H, 8-N*H*), 7.65 (d, *J* = 8.9 Hz, 1H, *H*-4), 7.55 – 7.49 (m, 1H, *H*-7), 7.35 (ddd, *J* = 9.1 Hz, 6.4 Hz, 1.0 Hz, 1H, *H*-6), 7.04 (ddd, *J* = 8.9 Hz, 6.4 Hz, 0.8 Hz, 1H, *H*-5), 6.04 (t, *J* = 53.7 Hz, 1H, *H*-10), 4.94 (d, *J* = 6.1 Hz, 2H, *H*-8).
^{13}C NMR (101 MHz, CD_3CN) δ [ppm]: 164.9, 163.9 (t, *J* = 25.5 Hz), 157.9, 132.3, 124.9, 120.9, 116.5, 115.4, 109.6 (t, *J* = 248.5 Hz), 35.0.
^{19}F NMR (376 MHz, CD_3CN) δ [ppm]: -129.31 (d, *J* = 53.7 Hz).
HR-MS (ESI+): *m/z* for $C_{10}H_8F_2N_2O_2+H^+$, $[M+H]^+$ calculated: 227.0627; found: 227.0614.

3-(*N*-(3,3-difluorocyclobutan)amidomethyl)benzo[*c*]isoxazole (5q)

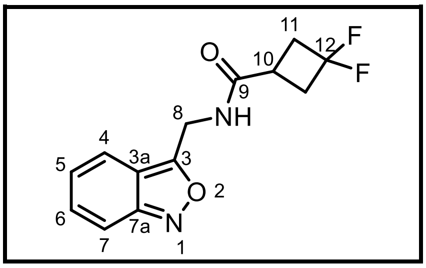

5 mL scale: According to general protocol **GP7**, 3,3-difluoro-*N*-(2-(2-nitrophenyl)-2-oxoethyl)cyclobutane-1-carboxamide (**4q**, 44.7 mg, 0.15 mmol, 1.0 eq.) was reacted. 28.3 mg (0.106 mmol, 70%) of the product were obtained as an off-white solid after silica flash column chromatography (cyclohexane:ethyl acetate; regular silica 25 g, particle size: 15 µm, SIHP) using 4 → 47% ethyl acetate.

^{1}H NMR (400 MHz, CD_2Cl_2) δ [ppm]: 7.59 (dt, *J* = 8.9 Hz, 1.1 Hz, 1H, *H*-4), 7.48 (dt, *J* = 9.2 Hz, 1.0 Hz, 1H, *H*-7), 7.30 (ddd, *J* = 9.2 Hz, 6.3 Hz, 1.0 Hz, 1H, *H*-6), 6.99 (ddd, *J* = 8.9 Hz, 6.3 Hz, 0.8 Hz, 1H, *H*-5), 6.70 (s, 1H8-N*H*), 4.92 (d, *J* = 6.0 Hz, 2H, *H*-8), 2.92 – 2.65 (m, 5H, *H*-10, *H*-11).
^{13}C NMR (101 MHz, CD_2Cl_2) δ [ppm]: 172.9 (t, *J* = 2.2 Hz), 164.6, 157.5, 131.7, 124.4, 120.2, 119.4 (dd, *J* = 284.9, 269.3 Hz), 116.0, 115.0, 38.9 (t, *J* = 24.2 Hz), 35.1, 28.0 (dd, *J* = 14.7, 4.7 Hz).
^{19}F NMR (376 MHz, CD_2Cl_2) δ [ppm]: -83.57 – -83.69 (m), -84.14 (m), -98.97 – -99.17 (m), -99.48 – -99.68 (m).
HR-MS (ESI+): *m/z* for $C_{13}H_{12}F_2N_2O2+H^+$, $[M+H]^+$ calculated: 267.0940; found: 267.0929.

3-(*N*-(Bicyclo[1.1.1]pentan)amidomethyl)benzo[*c*]isoxazole (5r)

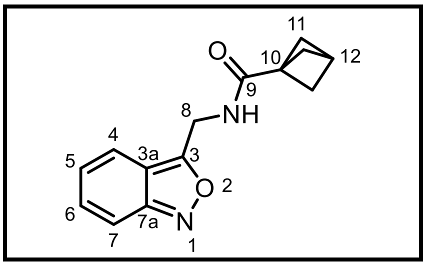

5 mL scale: According to general protocol **GP7**, *N*-(2-(2-nitrophenyl)-2-oxoethyl)bicyclo[1.1.1]pentane-1-carboxamide (**4r**, 41.1 mg, 0.15 mmol, 1.0 eq.) was reacted. 21.1 mg (0.0871 mmol, 58%) of the product were obtained as a colourless oil after silica flash column chromatography (cyclohexane:ethyl acetate; regular silica 25 g, particle size: 15 µm, SIHP) using 4 → 47% ethyl acetate.

^{1}H NMR (400 MHz, CD_2Cl_2) δ [ppm]: 7.58 (dt, *J* = 8.9 Hz, 1.1 Hz, 1H, *H*-4), 7.48 (dt, *J* = 9.2 Hz, 1.0 Hz, 1H, *H*-7), 7.28 (ddd, *J* = 9.2 Hz, 6.4 Hz, 1.0 Hz, 1H, *H*-6), 6.97 (ddd, *J* = 8.9 Hz, 6.4 Hz, 0.8 Hz, 1H, *H*-5), 6.50 (s, 1H, 8-N*H*), 4.88 (d, *J* = 6.1 Hz, 2H, *H*-8), 2.44 (s, 1H, *H*-12), 2.03 (s, 6H, *H*-11).
^{13}C NMR (101 MHz, CD_2Cl_2) δ [ppm]: 170.1, 164.9, 157.5, 131.5, 124.2, 120.4, 116.0, 115.0, 51.3, 44.6, 34.7, 27.4.
HR-MS (ESI+): *m/z* for $C_{14}H_{14}N_2O_2+H^+$, $[M+H]^+$ calculated: 243.1128; found: 243.1117.

3-(*N*-(3-Trifluoromethyl-bicyclo[1.1.1]pentan)amidomethyl)benzo[*c*]isoxazole (5s)

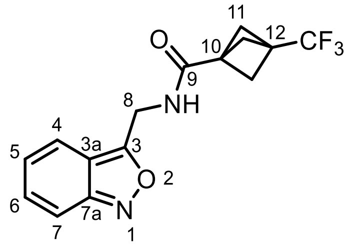

5 mL scale: According to general protocol **GP7**, *N*-(2-(2-nitrophenyl)-2-oxoethyl)-3-(trifluoromethyl)bicyclo[1.1.1]pentane-1-carboxamide (**4s**. 51.3 mg, 0.15 mmol, 1.0 eq.) was reacted. 32.0 mg (0.103 mmol, 69%) of the product were obtained as a colourless oil after silica flash column chromatography (cyclohexane:ethyl acetate; regular silica 25 g, particle size: 15 µm, SIHP) using 4 → 38% ethyl acetate.

^{1}H NMR (400 MHz, CD_2Cl_2) δ [ppm]: 7.59 (d, *J* = 8.9 Hz, 1H, *H*-4), 7.53 – 7.47 (m, 1H, *H*-7), 7.30 (ddd, *J* = 9.2 Hz, 6.4 Hz, 1.0 Hz, 1H, *H*-6), 7.00 (ddd, *J* = 8.9 Hz, 6.4 Hz, 0.8 Hz, 1H, *H*-5), 6.57 (s, 1H, 8-N*H*), 4.90 (d, *J* = 6.0 Hz, 2H, *H*-8), 2.22 (s, 9H, *H*-11).
^{13}C NMR (101 MHz, CD_2Cl_2) δ [ppm]: 168.6, 164.4, 157.6, 131.7, 124.5, 123.0 (q, *J* = 275.8 Hz), 120.2, 116.1, 115.1, 49.9 (q, *J* = 2.3 Hz), 38.9, 36.9 (q, *J* = 39.0 Hz), 34.7.
^{19}F NMR (376 MHz, CD_2Cl_2) δ [ppm]: -74.81.
HR-MS (ESI+): *m/z* for $C_{15}H_{13}F_3N_2O2+H^+$, $[M+H]^+$ calculated: 311.1002; found: 311.0992.

3-(*N*-(3-Methoxycarbonyl-bicyclo[1.1.1]pentan)amidomethyl)benzo[*c*]isoxazole (5t)

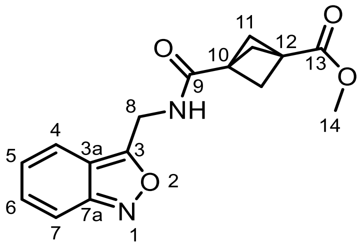

5 mL scale: According to general protocol **GP7**, methyl 3-((2-(2-nitrophenyl)-2-oxoethyl)carbamoyl)bicyclo[1.1.1]pentane-1-carboxylate (**4t**, 49.8 mg, 0.15 mmol, 1.0 eq.) was reacted. 16.1 mg (0.0536 mmol, 36%) of the product were obtained as a colourless oil after silica flash column chromatography (cyclohexane:ethyl acetate; regular silica 25 g, particle size: 15 µm, SIHP) using 6 → 64% ethyl acetate.

^{1}H NMR (400 MHz, CD_2Cl_2) δ [ppm]: 7.59 (d, *J* = 8.9 Hz, 1H, *H*-4), 7.52 – 7.44 (m, 1H, *H*-7), 7.29 (ddd, *J* = 9.1 Hz, 6.4 Hz, 1.0 Hz, 1H, *H*-6), 6.98 (ddd, *J* = 8.9 Hz, 6.4 Hz, 0.8 Hz, 1H, *H*-5), 6.53 (s, 1H. 8-N*H*), 4.89 (d, *J* = 6.1 Hz, 2H, *H*-8), 3.65 (s, 3H, *H*-14), 2.26 (s, 6H, *H*-11).
^{13}C NMR (101 MHz, CD_2Cl_2) δ [ppm]: 169.9, 169.4, 164.5, 157.6, 131.5, 124.3, 120.3, 116.1, 115.1, 52.6, 52.1, 39.4, 37.3, 34.7.
HR-MS (ESI+): *m/z* for $C_{16}H_{16}N_2O_2+H^+$, $[M+H]^+$ calculated: 301.1183; found: 301.1179.

3-(*N*-(4-Methoxycarbonyl-1-cuban)amidomethyl)benzo[*c*]isoxazole (5u)

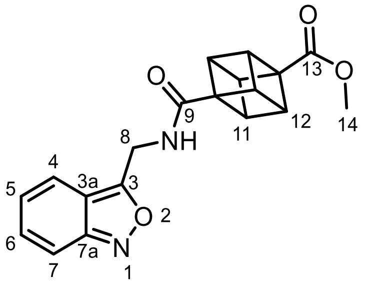

5 mL scale: According to general protocol **GP7**, methyl 4-((2-(2-nitrophenyl)-2-oxoethyl)carbamoyl)cubane-1-carboxylate (**4u**, 54.9 mg, 0.15 mmol) was reacted. 31.1 mg (0.0925 mmol, 62%) of the product were obtained as an colourless oil after silica flash column chromatography (cyclohexane:ethyl acetate; regular silica 25 g, particle size: 15 µm, SIHP) using 10 → 60% ethyl acetate.

^{1}H NMR (400 MHz, CD_2Cl_2) δ [ppm]: 7.61 (d, *J* = 8.8 Hz, 1H, *H*-4), 7.48 (d, *J* = 9.1 Hz, 1H, *H*-7), 7.29 (ddd, *J* = 9.1 Hz, 6.3 Hz, 1.0 Hz, 1H, *H*-6), 6.98 (ddd, *J* = 8.8 Hz, 6.3 Hz, 0.8 Hz, 1H, *H*-5), 6.60 (t, *J* = 6.0 Hz, 1H, 8-N*H*), 4.93 (d, *J* = 6.1 Hz, 2H, *H*-8), 4.16 (s, 6H, *H*-11, *H*-12), 3.66 (s, 3H, *H*-14).
^{13}C NMR (101 MHz, CD_2Cl_2) δ [ppm]: 171.7, 171.2, 164.6, 157.2, 131.2, 123.8, 120.0, 115.6, 114.6, 57.6, 55.9, 51.4, 47.0, 46.8, 34.2.
HR-MS (ESI+): *m/z* for $C_{19}H_{16}N_2O_4+H^+$, $[M+H]^+$ calculated: 337.1183; found: 337.1176.

3-((2-Oxopyrrolidinyl)methyl)benzo[*c*]isoxazole (5v)

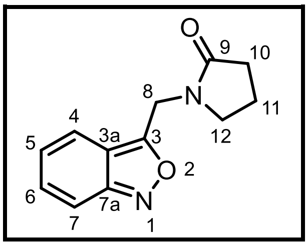

5 mL scale: According to general protocol **GP7**, *N*-(2-nitrophenacyl)pyrrolid-2-one (**4v**, 37.4 mg, 0.15 mmol) was reacted. 23.6 mg (0.102 mmol, 67%) of the product were obtained as an off-white solid after silica flash column chromatography (cyclohexane:ethyl acetate; regular silica 25 g, particle size: 15 µm, SIHP) using 12 → 90% ethyl acetate.

^{1}H NMR (400 MHz, CD_2Cl_2) δ [ppm]: 7.58 (dt, *J* = 8.8 Hz, 1.1 Hz, 1H, *H*-4), 7.52 (dt, *J* = 9.1 Hz, 1.0 Hz, 1H, *H*-7), 7.30 (ddd, *J* = 9.1 Hz, 6.4 Hz, 1.0 Hz, 1H, *H*-6), 6.99 (ddd, *J* = 8.8 Hz, 6.4 Hz, 0.8 Hz, 1H, *H*-5), 4.92 (s, 2H, *H*-8), 3.40 (t, *J* = 6.9 Hz, 2H, *H*-10), 2.36 (t, *J* = 8.1 Hz, 2H, *H*-12), 2.07 – 1.95 (m, 2H, *H*-11).
^{13}C NMR (101 MHz, CD_2Cl_2) δ [ppm]: 175.2, 164.0, 157.5, 131.5, 124.4, 120.1, 116.3, 115.2, 47.6, 37.9, 30.6, 18.1.
HR-MS (ESI+): *m/z* for $C_{12}H_{12}N_2O_2$+H$^+$, [M+H]$^+$ calculated: 217.0972; found: 217.0969.

3-(*N*-Acetamidomethyl)-5-methyl-benzo[*c*]isoxazole (5w)

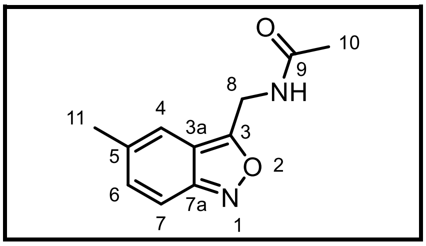

5 mL scale: According to general protocol **GP7**, *N*-(5-methyl-2-nitrophenacyl)acetamide (**4w**, 35.5 mg, 0.15 mmol) was reacted. 23.7 mg (0.116 mmol, 77%) of the product were obtained as an off-white solid after silica flash column chromatography (cyclohexane:ethyl acetate; regular silica 25 g, particle size: 15 µm, SIHP) using 10 → 60% ethyl acetate.

^{1}H NMR (400 MHz, CD_2Cl_2) δ [ppm]: 7.39 (d, *J* = 9.2 Hz, 1H, *H*-7), 7.29 (d, *J* = 1.2 Hz, 1H, *H*-4), 7.13 (dd, *J* = 9.2 Hz, 1.2 Hz, 1H, *H*-6), 6.60 (s, 1H, 8-N*H*), 4.83 (d, *J* = 6.0 Hz, 1H, *H*-8), 2.32 (s, 3H, *H*-11), 1.98 (s, 3H, *H*-10).
^{13}C NMR (101 MHz, CD_2Cl_2) δ [ppm]: 170.4, 163.5, 157.0, 135.0, 133.9, 117.3, 116.2, 114.7, 34.8, 23.1, 21.8.
HR-MS (ESI+): *m/z* for $C_{11}H_{11}N_2O_2$+H$^+$, [M+H]$^+$ calculated: 205.0972; found: 205.0962.

3-(*N*-Acetamidomethyl)-5-methoxy-benzo[*c*]isoxazole (5x)

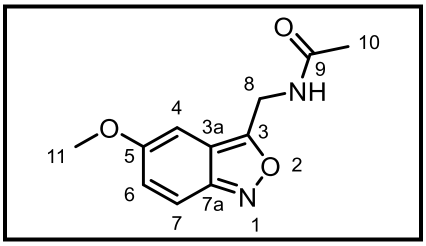

5 mL scale: According to general protocol **GP7**, *N*-(5-methoxy-2-nitrophenacyl)acetamide (**4x**, 37.8 mg, 0.15 mmol) was reacted. 23.2 mg (0.105 mmol, 70%) of the product were obtained as a beige solid after reversed phase silica flash column chromatography (water+0.5% formic acid:acetonitrile; regular C_{18} silica 80 g, particle size: 30 µm, SIHP) using 20 → 50% acetonitrile.

^{1}H NMR (400 MHz, CD_2Cl_2) δ [ppm]: 7.40 (d, *J* = 9.6 Hz, *H*-7), 7.00 (dd, *J* = 9.6 Hz, 2.2 Hz, 1H, *H*-6), 6.68 (d, *J* = 2.2 Hz, 1H, *H*-4), 6.44 (s, 1H, 8-N*H*), 4.81 (d, *J* = 5.9 Hz, 2H, *H*-8), 3.81 (s, 3H, *H*-11), 1.98 (s, 3H, *H*-10).
^{13}C NMR (101 MHz, CD_2Cl_2) δ [ppm]: 170.4, 162.5, 156.2, 155.8, 128.7, 116.6, 116.0, 93.6, 55.8, 34.7, 23.1
HR-MS (ESI+): *m/z* for $C_{11}H_{12}N_2O_3$+H$^+$, [M+H]$^+$ calculated: 221.0921; found: 221.0910.

3-(*N*-Acetamidomethyl)-3,4,5-trimethoxy-benzo[*c*]isoxazole (5y)

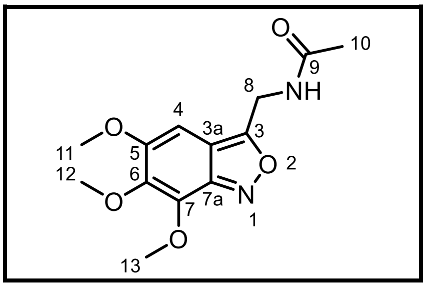

5 mL scale: According to general protocol **GP7**, *N*-(2-nitro-3,4,5-trimethoxyphenacyl)acetamide (**4y**, 46.7 mg, 0.15mmol) was reacted. 31.7 mg (0.112 mmol, 75%) of the product were obtained as a colourless solid after silica flash column chromatography (cyclohexane:ethyl acetate; regular silica 25 g, particle size: 15 µm, SIHP) using 15 → 65% ethyl acetate.

^{1}H NMR (400 MHz, CD_2Cl_2) δ [ppm]: 6.46 (s, 2H, *H*-4, 8-N*H*), 4.78 (d, *J* = 5.9 Hz, 1H, *H*-8), 4.13 (s, 3H, *H*-13), 3.89 (s, 3H, *H*-11), 3.83 (s, 3H, *H*-12), 1.99 (s, 3H, *H*-10).
^{13}C NMR (101 MHz, CD_2Cl_2) δ [ppm]: 170.6, 162.8, 153.1, 152.5, 144.1, 138.1, 113.7, 89.8, 61.8, 61.2, 56.5, 34.8, 23.2.
HR-MS (ESI+): *m/z* for $C_{13}H_{16}N_2O_5$+H^+, $[M+H]^+$ calculated: 281.1132; found: 281.1127.

3-(*N*-Acetamidomethyl)-5-trifluoromethyl-benzo[*c*]isoxazole (5z)

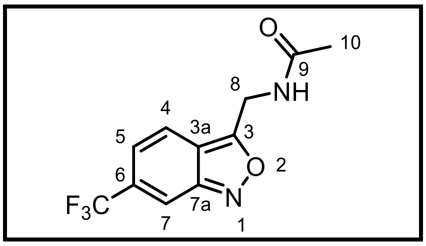

5 mL scale: According to general protocol **GP7**, *N*-(2-nitro-4-trifluoromethylphenacyl)acetamide (**4z**, 43.5 mg, 0.15 mmol) was reacted. 24.3 mg (0.0941 mmol, 63%) of the product were obtained as an off-white solid after silica flash column chromatography (cyclohexane:ethyl acetate; regular silica 25 g, particle size: 15 µm, SIHP) using 15 → 65% ethyl acetate.

^{1}H NMR (400 MHz, CD_2Cl_2) δ [ppm]: 7.89 (q, *J* = 1.3 Hz, 1H, *H*-7), 7.82 (dt, *J* = 9.1 Hz, 0.9 Hz, 1H, *H*-4), 7.11 (dd, *J* = 9.1 Hz, 1.3 Hz, 1H, *H*-5), 6.45 (s, 1H, 8-N*H*), 4.93 (d, *J* = 6.1 Hz, 2H, *H*-8), 2.00 (s, 3H, *H*-10).
^{13}C NMR (101 MHz, CD_2Cl_2) δ [ppm]: 170.5, 166.9, 156.3, 133.3 (q, *J* = 32.2 Hz), 123.9 (q, *J* = 272.7 Hz), 122.8, 119.7 (q, *J* = 2.7 Hz), 116.3, 114.3 (q, *J* = 5.4 Hz), 35.1, 23.0.
^{19}F NMR (376 MHz, CD_2Cl_2) δ [ppm]: -64.90.
HR-MS (ESI+): *m/z* for $C_{11}H_9F_3N_2O_2$+H^+, $[M+H]^+$ calculated: 259.0689; found: 259.0684.

3-(*N*-Acetamidomethyl)-4-fluoro-benzo[*c*]isoxazole (5aa)

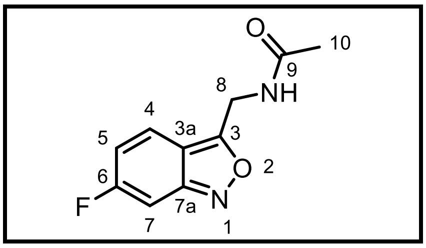

5 mL scale: According to general protocol **GP7**, *N*-(5-fluoro-2-nitrophenacyl)acetamide (**4aa**, 36.1 mg, 0.15 mmol) was reacted. 15.9 mg (0.0765 mmol, 51%%) of the product were obtained as an off-white solid after silica flash column chromatography (cyclohexane:ethyl acetate; regular silica 25 g, particle size: 15 µm, SIHP) using 10 → 60% ethyl acetate.

^{1}H NMR (400 MHz, CD_2Cl_2) δ [ppm]: 7.69 (ddd, *J* = 9.4 Hz, 5.3 Hz, 0.8 Hz, 1H, *H*-7), 7.09 (ddd, *J* = 9.6 Hz, 2.1 Hz, 0.8 Hz, 1H, *H*-4), 6.82 (ddd, *J* = 9.4 Hz, 8.8 Hz, 2.1 Hz, 1H, *H*-6), 6.22 (s, 1H, 8-N*H*), 4.88 (d, *J* = 6.1 Hz, 1H), 2.00 (s, 1H).
^{13}C NMR (101 MHz, CD_2Cl_2) δ [ppm]: 170.4, 166.1 – 165.5 (m), 163.2, 157.8 (d, *J* = 13.3 Hz), 123.5 (d, *J* = 11.4 Hz), 117.4 (d, *J* = 30.9 Hz), 113.9, 97.3 (d, *J* = 25.2 Hz), 34.9, 23.1.
^{19}F NMR (376 MHz, CD_2Cl_2) δ [ppm]: -106.08.
HR-MS (ESI+): *m/z* for $C_{10}H_9FN_2O_2$+H^+, $[M+H]^+$ calculated: 209.0721; found: 209.0712.

3-(Acetamidomethyl)-5-chloro-benzo[*c*]isoxazole (5ab)

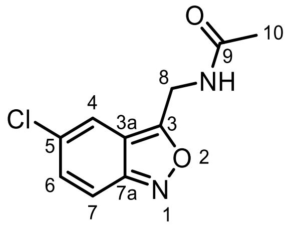

5 mL scale: According to general protocol **GP7**, *N*-(5-chloro-2-nitrophenacyl)acetamide (**4ab**, 38.5 mg, 0.15 mmol) was reacted. 20.1 mg (0.0895 mmol, 60%) of the product were obtained as an colourless solid after silica flash column chromatography (cyclohexane:ethyl acetate; regular silica 25 g, particle size: 15 µm, SIHP) using 10 → 60% ethyl acetate.

^{1}H NMR (400 MHz, CD_3CN) δ [ppm]: 7.70 (dd, *J* = 1.9 Hz, 0.9 Hz, 1H, *H*-4), 7.52 (dd, *J* = 9.4 Hz, 0.9 Hz, 1H, *H*-7), 7.26 (dd, *J* = 9.4 Hz, 1.9 Hz, 1H, *H*-5), 7.15 (s, 1H, 8-N*H*), 4.78 (d, *J* = 6.0 Hz, 2H, *H*-8), 1.92 (s, 3H, *H*-10).
^{13}C NMR (101 MHz, CD_3CN) δ [ppm]: 171.3, 167.1, 156.5, 133.6, 129.5, 119.9, 117.6, 116.4, 35.4, 22.8.
HR-MS (ESI-): *m/z* for $C_{10}H_9{}^{35}ClN_2O_2$-H^-, $[M-H]^-$ calculated: 223.0280; found: 223.0285.

3-(Acetamidomethyl)-5-bromo-benzo[*c*]isoxazole (5ac)

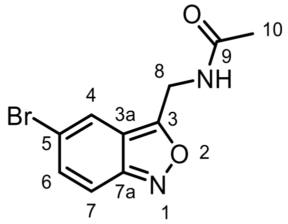

5 mL scale: According to general protocol **GP7**, *N*-(5-bromo-2-nitrophenacyl)acetamide (**4ac**, 44.9 mg, 0.15 mmol) was reacted. Additional 0.5 mL of acetone were added instead of water to increase the solubility of the substrate. 27.1 mg (0.0734 mmol, 49%) of the product were obtained as an off-white solid after silica flash column chromatography (cyclohexane:ethyl acetate; regular silica 25 g, particle size: 15 µm, SIHP) using 10 → 60% ethyl acetate.

^{1}H NMR (400 MHz, CD_2Cl_2) δ [ppm]: 7.84 (d, *J* = 0.8 Hz, 1H, *H*-4), 7.41 (dd, *J* = 9.4 Hz, 0.8 Hz, 1H, *H*-7), 7.32 (dd, *J* = 9.4 Hz, 1.7 Hz, 1H, *H*-6), 6.53 (s, 1H, 8-N*H*), 4.84 (d, *J* = 6.1 Hz, 2H, *H*-8), 1.99 (s, 3H, *H*-10).
^{13}C NMR (101 MHz, CD_2Cl_2) δ [ppm]: 170.5, 165.0, 156.0, 135.3, 122.5, 117.6, 117.0, 116.9, 34.9, 23.1.
HR-MS (ESI+): *m/z* for $C_{10}H_9{}^{79}BrN_2O_2$+H^+, $[M+H]^+$ calculated: 268.9920; found: 268.9913.

3-(Acetamidomethyl)benzo[*c*]isoxazole (5ad)

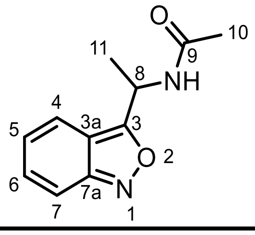

5 mL scale: According to general protocol **GP7**, *N*-(1-(2-nitrophenyl)-1-oxopropan-2-yl)acetamide (**4ad**, 35.2 mg, 0.15 mmol, 1.0 eq.) was reacted. 21.1 mg (0.103 mmol, 69%) of the product were obtained as an off-white solid after silica flash column chromatography (cyclohexane:ethyl acetate; regular silica 25 g, particle size: 15 µm, SIHP) using 12 → 90% ethyl acetate.

^{1}H NMR (400 MHz, CD_2Cl_2) δ [ppm]: 7.61 (dd, *J* = 8.8 Hz, 1.1 Hz, 1H, *H*-4), 7.48 (dd, *J* = 9.2 Hz, 1.0 Hz, 1H, *H*-7), 7.28 (ddd, *J* = 9.2 Hz, 6.3 Hz, 1.0 Hz, 1H, *H*-6), 6.96 (ddd, *J* = 8.8 Hz, 6.3 Hz, 0.8 Hz, 1H, *H*-5), 6.52 (s, 1H, 8-N*H*), 5.73 (p, *J* = 7.1 Hz, 1H, *H*-8), 1.96 (s, 3H, *H*-10), 1.67 (d, 3H, *J* = 7.1 Hz, *H*-10).
^{13}C NMR (101 MHz, CD_2Cl_2) δ [ppm]: 169.6, 168.4, 157.5, 131.4, 124.0, 120.5, 115.0, 114.8, 42.4, 23.2, 19.3.
HR-MS (ESI+): *m/z* for $C_{11}H_{12}N_2O_2$+H^+, $[M+H]^+$ calculated: 205.0972; found: 205.0971.

3-(Acetamidomethyl-1-methylethyl)benzo[*c*]isoxazole (5ae)

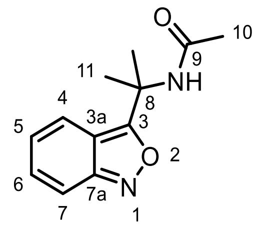

5 mL scale: According to general protocol **GP7**, *N*-(2-methyl-1-(2-nitrophenyl)-1-oxopropan-2-yl)acetamide (**4ae**, 37.5 mg, 0.15 mmol) was reacted. 24.3 mg (0.111 mmol, 74%) of the product were obtained as a colourless oil after silica flash column chromatography (dichloromethane:methanol; regular silica 25 g, particle size: 15 µm, SIHP) using 0 → 5% methanol.

^{1}H NMR (400 MHz, CD_2Cl_2) δ [ppm]: 7.57 (d, *J* = 8.9 Hz, 1H, *H*-4), 7.51 – 7.46 (m, 1H, *H*-7), 7.26 (ddd, *J* = 9.1 Hz, 6.4 Hz, 1.0 Hz, 1H, *H*-6), 6.92 (ddd, *J* = 8.9 Hz, 6.4 Hz, 0.8 Hz, 1H, *H*-5), 6.52 (s, 1H, 8-N*H*), 1.90 (s, 3H, *H*-10), 1.80 (s, 6H, *H*-11).
^{13}C NMR (101 MHz, CD_2Cl_2) δ [ppm]: 171.8, 169.9, 157.8, 131.0, 123.7, 121.1, 115.5, 113.9, 53.8, 27.5, 23.7.
HR-MS (ESI+): *m/z* for $C_{12}H_{14}N_2O_2$+H$^+$, [M+H]$^+$ calculated: 219.1128; found: 219.1121.

3-(Acetamidomethyl -2-phenylethyl)benzo[*c*]isoxazole (5af)

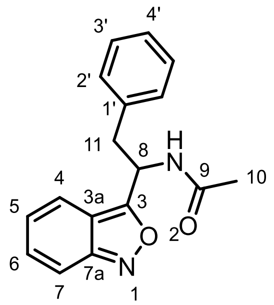

5 mL scale: According to general protocol **GP7**, *N*-(1-(2-nitrophenyl)-1-oxo-3-phenylpropan-2-yl)acetamide (**4af**, 46.8 mg, 0.15 mmol) was reacted. 24.3 mg (0.114 mmol, 76%) of the product were obtained as a yellow oil after silica flash column chromatography (cyclohexane:chloroform + 0.5% acetic acid; regular silica 25 g, particle size: 15 µm, SIHP) using 20 → 100% chloroform.

^{1}H NMR (400 MHz, CD_2Cl_2) δ [ppm]: 7.50 – 7.44 (m, 1H, *H*-4), 7.35 (dt, *J* = 8.9 Hz, 1.0 Hz, 1H, *H*-7), 7.29 – 7.16 (m, 4H, *H*-5, *H*-2‘,*H*-4‘), 7.09 (dd, *J* = 7.8 Hz, 1.7 Hz, 2H, *H*-3‘), 6.88 (ddd, *J* = 8.9 Hz, 6.3 Hz, 0.8 Hz, 1H, *H*-6), 6.31 (d, *J* = 8.4 Hz, 1H, 8-N*H*), 5.90 (dt, *J* = 8.4 Hz, 7.5 Hz, 1H, *H*-8), 3.34 (dd, *J* = 7.5 Hz, 1.5 Hz, 2H, *H*-11), 1.94 (s, 3H, *H*-10).
^{13}C NMR (101 MHz, CD_2Cl_2) δ [ppm]: 169.6, 166.7, 157.4, 136.6, 131.4, 129.5, 128.9, 127.4, 124.1, 120.1, 115.7, 115.0, 48.0, 40.1, 23.2.
HR-MS (ESI+): *m/z* for $C_7H_{16}N_2O_2$+H$^+$, [M+H]$^+$ calculated: 281.1285; found: 281.1279.

3-(Acetamidomethyl-1-(ethoxycarbonyl)methyl)benzo[*c*]isoxazole (5ag)

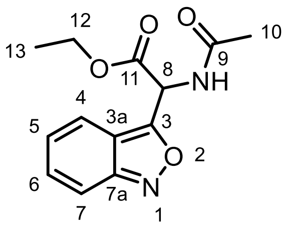

5 mL scale: According to general protocol **GP7**, ethyl 2-acetamido-3-(2-nitrophenyl)-3-oxopropanoate (**4ag**, 44.1 mg, 0.15 mmol) was reacted. 24.8 mg (0.0945 mmol, 63%) of the product were obtained as an off-white solid after reversed phase silica flash column chromatography (water+0.5% formic acid:acetonitrile; regular C_{18} silica 80 g, particle size: 30 µm, SIHP) using 20 → 50% acetonitrile.

^{1}H NMR (400 MHz, CD_2Cl_2) δ [ppm]: 7.65 (dd, *J* = 8.9 Hz, 1.0 Hz, 1H, *H*-4), 7.53 (dd, *J* = 9.2 Hz, 1.0 Hz, 1H, *H*-7), 7.32 (ddd, *J* = 9.2 Hz, 6.3 Hz, 0.9 Hz, 1H, *H*-6), 7.04 (ddd, *J* = 8.9 Hz, 6.3 Hz, 0.8 Hz, 1H, *H*-5), 6.88 (s, 1H, 8-N*H*), 6.28 (d, *J* = 7.4 Hz, 1H, *H*-8),4.34 – 4.13 (m, 2H, *H*-12), 2.04 (s, 3H, *H*-10), 1.20 (t, *J* = 7.1 Hz, 3H, *H*-13).
^{13}C NMR (101 MHz, CD_2Cl_2) δ [ppm]: 170.0, 167.7, 161.8, 157.8, 131.8, 125.2, 120.2, 116.5, 115.5, 63.6, 49.7, 23.1, 14.3.
HR-MS (ESI+): *m/z* for $C_{13}H_{14}N_2O_4$+H$^+$, [M+H]$^+$ calculated: 263.1026; found: 263.1024.

3-(2-Acetamidoethyl)benzo[*c*]isoxazole (5ah)

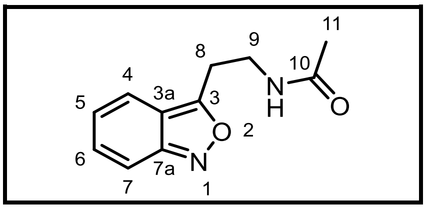

5 mL scale: According to general protocol **GP7**, *N*-(3-(2-nitrophenyl)-3-oxopropyl)acetamide (**4ah**, 35.4 mg, 0.15 mmol) was reacted. 22.0 mg (0.108 mmol, 72%) of the product were obtained as a colourless oil after silica flash column chromatography (dichloromethane:methanol; regular silica 25 g, particle size: 15 µm, SIHP) using 4 → 20% methanol.

^{1}H NMR (400 MHz, CD_2Cl_2) δ [ppm]: 7.49 – 7.44 (m, 2H, *H*-4, *H*-6), 7.27 (ddd, *J* = 9.4 Hz, 6.3 Hz, 0.9 Hz, 1H, *H*-6), 6.98 – 6.92 (m, 1H, *H*-5), 6.11 (s, 1H, 9-N*H*), 3.66 (q, *J* = 6.5 Hz, 2H, *H*-9), 3.37 (t, *J* = 6.5 Hz, 1H, *H*-8), 1.88 (s, 3H, *H*-11).
^{13}C NMR (101 MHz, CD_2Cl_2) δ [ppm]: 170.7, 167.5, 157.5, 131.5, 123.8, 120.3, 116.6, 115.2, 38.2, 27.5, 23.4.
HR-MS (ESI+): *m/z* for $C_{11}H_{12}N_2O_2+H^+$, $[M+H]^+$ calculated: 205.0972; found: 205.0965.

3-Methylbenzo[*c*]isoxazole (5ai)

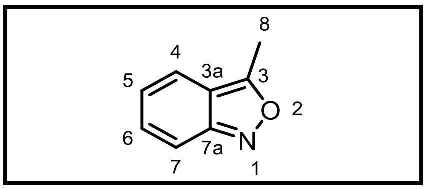

100 mL scale: According to general protocol **GP5**, 2-nitro-acetophenone (495.7 mg, 3.0 mmol) was reacted. 283.7 mg (2.13 mmol, 71%) of the product was obtained as a yellow oil after silica flash column chromatography (cyclohexane:ethyl acetate SIHP: 40 g regular silica column; particle size: 15 µm) using 0 → 10% ethyl acetate.
Highly volatile! Don't expose to a high vacuum for a long time.

^{1}H NMR (400 MHz, CD_2Cl_2) δ [ppm]: 7.50 – 7.41 (m, 2H, *H*-4, *H*-7), 7.26 (ddd, *J* = 9.1 Hz, 6.3 Hz, 1.0 Hz, 1H, *H*-6), 6.91 (dd, *J* = 8.8 Hz, 6.3 Hz, 1H, *H*-5), 2.75 (s, 3H, *H*-8).
^{13}C NMR (101 MHz, CD_2Cl_2) δ [ppm]: 166.3, 157.3, 131.1, 123.0, 120.3, 115.9, 114.9, 12.1.
HR-MS (ESI+): *m/z* for $C_8H_7NO+H^+$, $[M+H]^+$ calculated: 134.0600; found: 134.0601.

3-Ethylbenzo[*c*]isoxazole (5aj)

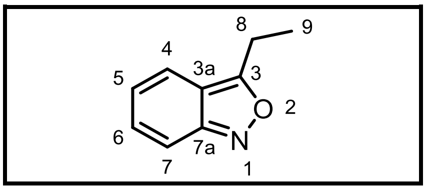

100 mL scale: According to general protocol **GP5**, 2-nitro-propiophenone (**11**, 537.7 mg, 3.0 mmol) was reacted. 306.0 mg (2.08 mmol, 69%) of the product was obtained as a yellow oil after silica flash column chromatography (cyclohexane:ethyl acetate SIHP: 40 g regular silica column; particle size: 15 µm) using 0 → 10% ethyl acetate.
Highly volatile! Don't expose to a high vacuum for a long time.

^{1}H NMR (400 MHz, CD_2Cl_2) δ [ppm]: 7.53 – 7.46 (m, 2H, *H*-4, *H*-7), 7.26 (ddd, *J* = 9.3 Hz, 6.3 Hz, 0.9 Hz, 1H, *H*-6), 6.96 – 6.88 (m, 1H, *H*-5), 3.17 (q, *J* = 7.6 Hz, 2H, *H*-8), 1.44 (t, *J* = 7.6 Hz, 3H, *H*-9).
^{13}C NMR (101 MHz, CD_2Cl_2) δ [ppm]: 171.1, 157.5, 131.2, 123.1, 120.6, 115.2, 115.2, 20.9, 12.4.
HR-MS (ESI+): *m/z* for $C_9H_9NO+H^+$, $[M+H]^+$ calculated: 148.0757; found: 148.0753.

3-Isopropylbenzo[*c*]isoxazole (5ak)

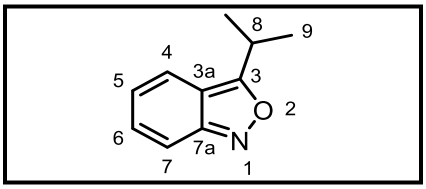

100 mL scale: According to general protocol **GP5**, 2-nitro-isobutyrophenone (**12**, 579.6 mg, 3.0 mmol) was reacted. 355.7 mg (2.20 mmol, 73%) of the product was obtained as a yellow oil after silica flash column chromatography (cyclohexane:ethyl acetate SIHP: 40 g regular silica column; particle size: 15 µm) using 0 → 10% ethyl acetate.
Highly volatile! Don't expose to a high vacuum for a long time.

^{1}H NMR (400 MHz, CD_2Cl_2) δ [ppm]: 7.56 (dt, *J* = 8.8 Hz, 1.0 Hz, 1H, *H*-4), 7.49 (dt, *J* = 9.0 Hz, 1.0 Hz, 1H, *H*-7), 7.26 (ddd, *J* = 9.0 Hz, 6.3 Hz, 1.0 Hz, 1H, *H*-6), 6.91 (ddd, *J* = 8.8 Hz, 6.3 Hz, 0.8 Hz, 1H, *H*-5), 3.59 (hept, *J* = 7.0 Hz, 1H, *H*-8), 1.49 (d, *J* = 7.0 Hz, 6H, *H*-9).
^{13}C NMR (101 MHz, CD_2Cl_2) δ [ppm]: 174.3, 157.6, 131.1, 123.0, 120.9, 115.3, 114.4, 28.7, 21.3.
HR-MS (ESI+): *m/z* for $C_{10}H_{11}NO+H^+$, $[M+H]^+$ calculated: 162.0913; found: 162.0908.

3-(2-Phenyl)-benzo[*c*]isoxazole (5al)

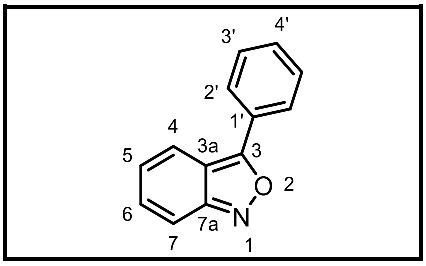

5 mL scale: According to general protocol **GP5**, 2-nitro-benzophenone (34.1 mg, 0.15 mmol) was reacted. 21.9 mg (0.112 mmol, 75%) of the product was obtained as a yellow oil after silica flash column chromatography (cyclohexane:ethyl acetate SIHP: 25 g regular silica column; particle size: 15 µm) using 0 → 5% ethyl acetate.

^{1}H NMR (400 MHz, CD_2Cl_2) δ [ppm]: 8.07 – 8.00 (m, 2H, *H*-2‘), 7.86 (dt, *J* = 8.9 Hz, 1.1 Hz, 1H, *H*-4), 7.64 – 7.54 (m, 3H, *H*-7, *H*-3‘), 7.56 – 7.47 (m, 1H, *H*-4‘), 7.35 (ddd, *J* = 9.2 Hz, 6.4 Hz, 1.0 Hz, 1H, *H*-6), 7.09 (ddd, *J* = 8.9 Hz, 6.4 Hz, 0.8 Hz, 1H, *H*-5).
^{13}C NMR (101 MHz, CD_2Cl_2) δ [ppm]: 164.9, 158.4, 131.2, 130.8, 129.8, 129.0, 127.1, 125.2, 121.2, 115.9, 114.9.
HR-MS (ESI+): *m/z* for $C_{13}H_9NO+H^+$, $[M+H]^+$ calculated: 196.0757; found: 196.0756.

3-(2-Phenylethyl)-benzo[*c*]isoxazole (5am)

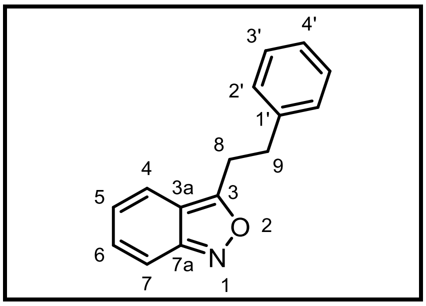

5 mL scale: According to general protocol **GP5**, 1-(2-nitrophenyl)-2-phenylethan-1-one (**13**, 38.3 mg, 0.15 mmol) was reacted. 20.6 mg (0.0923 mmol, 62%) of the product was obtained as a yellow oil after silica flash column chromatography (cyclohexane:ethyl acetate SIHP: 25 g regular silica column; particle size: 15 µm) using 0 → 5% ethyl acetate.

^{1}H NMR (400 MHz, CD_2Cl_2) δ [ppm]: 7.48 (d, *J* = 9.0 Hz, 1H, *H*-4), 7.35 – 7.16 (m, 7H, *H*-6, *H*-7, *H*-2‘,*H*-3‘,*H*-4‘), 6.88 (ddd, *J* = 9.0 Hz, 6.4 Hz, 0.8 Hz, 1H, *H*-5), 3.46 (t, *J* = 7.7 Hz, 2H, *H*-8), 3.17 (t, *J* = 7.8 Hz, 2H, *H*-9).
^{13}C NMR (101 MHz, CD_2Cl_2) δ [ppm]: 168.8, 157.4, 140.6, 131.1, 128.9, 128.7, 126.8, 123.2, 120.3, 115.8, 115.1, 34.3, 29.1.
HR-MS (ESI+): *m/z* for $C_{15}H_{13}NO+H^+$, $[M+H]^+$ calculated: 224.1070; found: 224.1070.

8. Crystallographic Data

3-(Acetamidomethyl)-5-bromo-benzo[*c*]isoxazole (5ac)

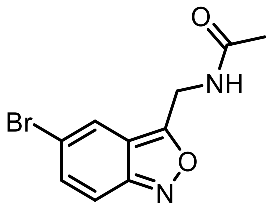

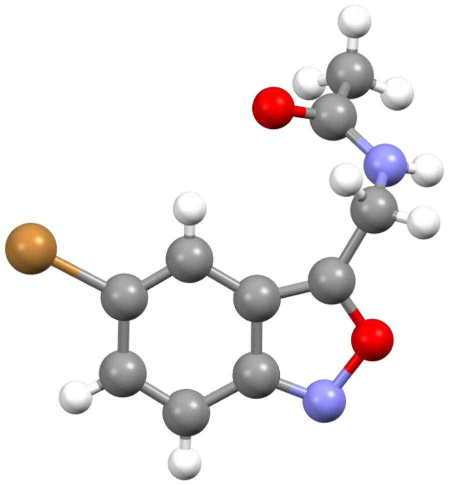

Crystallization was carried out by dissolving the compound in acetonitrile. Slow evaporating resulted in crystal formation.

CCDC Number	2389090	
Empirical formular	$C_{10}H_9BrN_2O_2$	
Moiety formular	$C_{10}H_9BrN_2O_2$	
Formular weight	269.10	
Temperature	120(2) K	
Wavelength, radiation type	0.71073A, MoKα	
Diffractometer	STOE IPDS 2T	
Crystal system	Triklinic	
Space group name, number	P -1, (2)	
Unit cell dimensions	a = 8.6341(5) Å	α = 72.973(5)°
	b = 9.4095(6) Å	β = 80.305(5)°
	c = 13.5968(8) Å	γ = 82.117(5)°
Volume	1036.60(11) $Å^3$	
Number of reflections	13360	
And range used for lattice parameters	3.07° <=Θ<= 28.35°	
Z	4	
Density (calculated)	1.724 Mg/m^3	
Absorption coefficient	3.946 mm^{-1}	
Absorption correction	Integration	
Max. and min. transmission	0.4233 and 0.0989	
F(000)	536	
Crystal size, colour and form	0.210 x 0.490 x 0.690 mm^3, colourless plate	
Theta range for data collection	3.069 bis 27.894°.	
Index ranges	-11<=h<=11, -12<=k<=10, -17<=l<=17	
Number of reflections:		
collected	9184	
independent	4937 [R_{int} = 0.0328]	
observed [I>2sigma(I)]	4329	
Completeness to theta = 25.2°	99.6%	
Refinement method	Full-matrix least-squares on F^2	
Data / restraints / parameters	4937 / 0 / 273	
Goodness-of-fit on F^2	1.174	
Final R indices [I>2sigma(I)]	R1 = 0.0430, wR2 = 0.1086	
R indices (all data)	R1 = 0.0508, wR2 = 0.1141	
Largest diff. peak and hole	1.174 und -0.989 eA^{-3}	

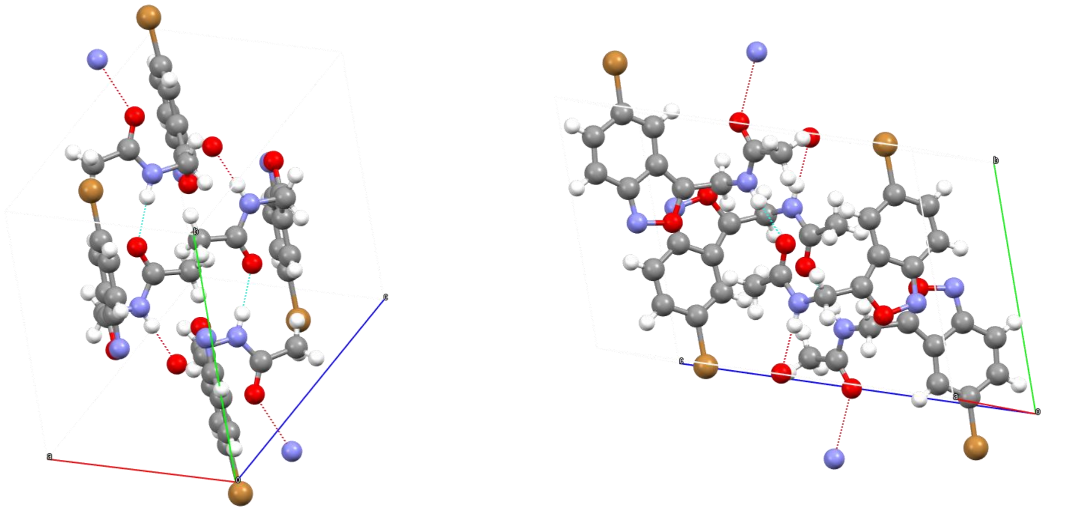

Figure S19: Packing of **5ac**.

9. NMR Spectra

^{1}H NMR spectrum (400 MHz, $CDCl_3$) of **8**.

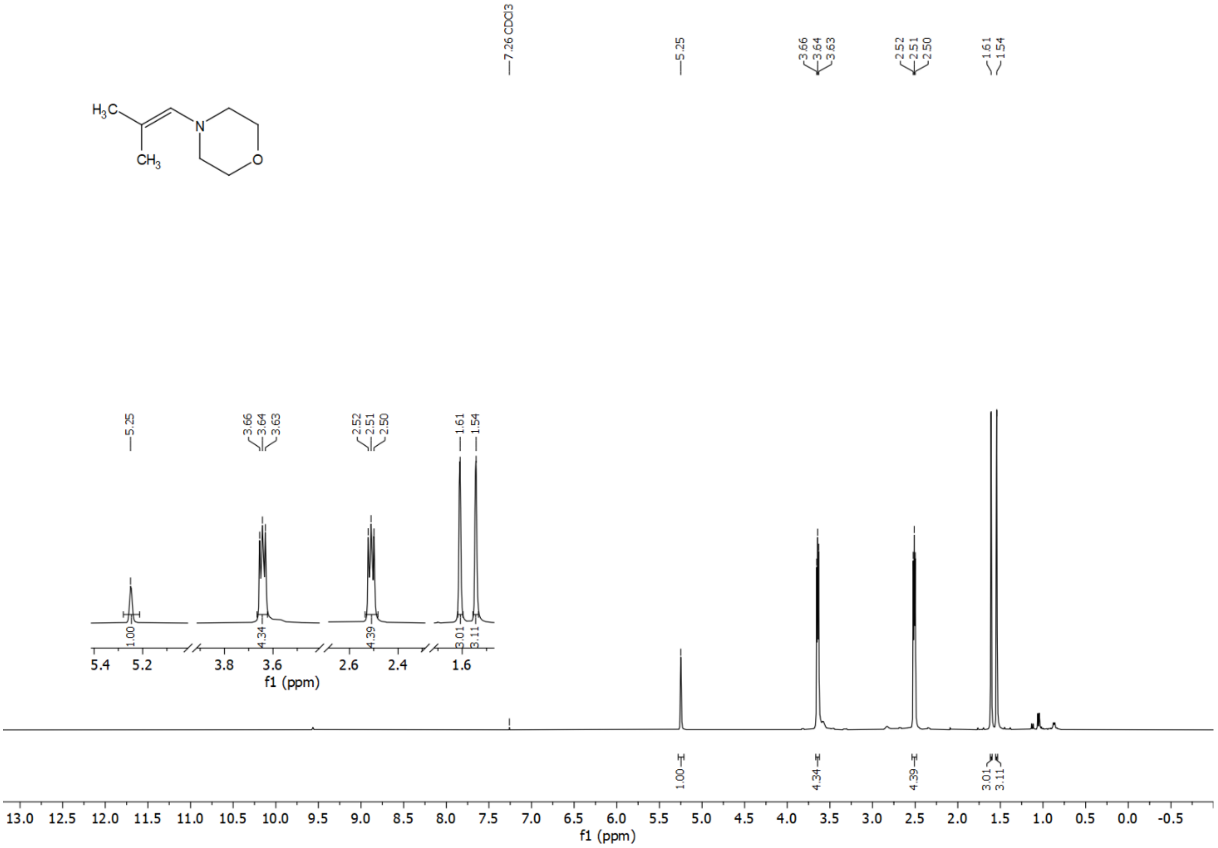

^{13}C NMR spectrum (101 MHz, $CDCl_3$) of **8**.

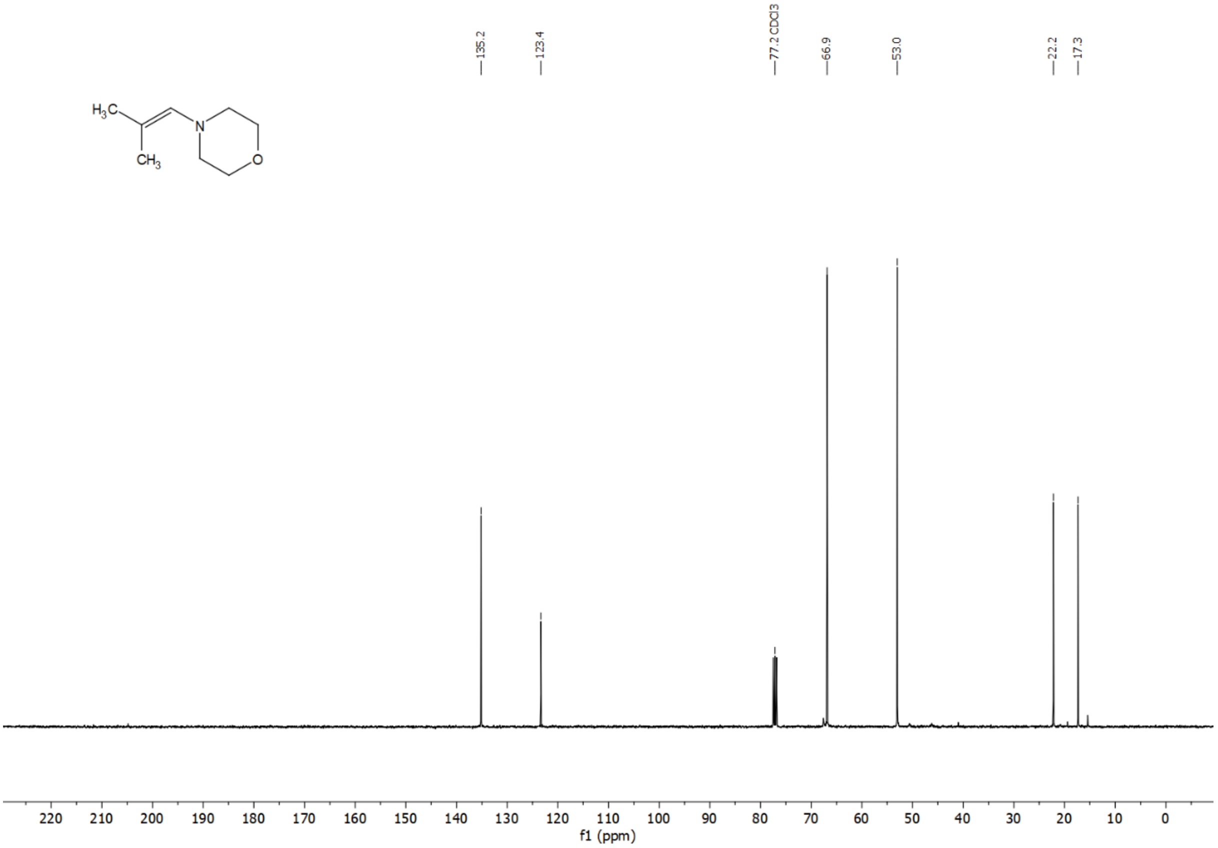

^{1}H NMR spectrum (400 MHz, CD_3CN) of **9**.

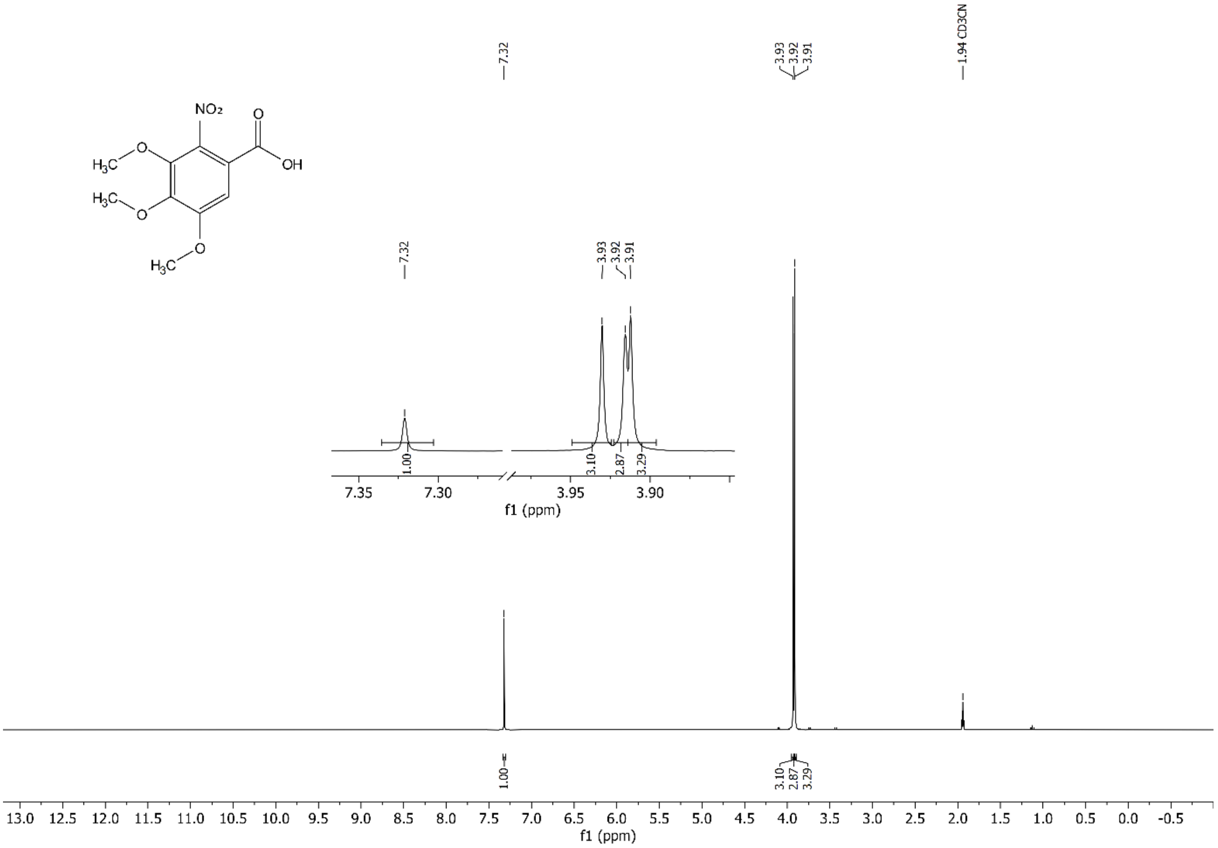

^{13}C NMR spectrum (101 MHz, CD_3CN) of **9**.

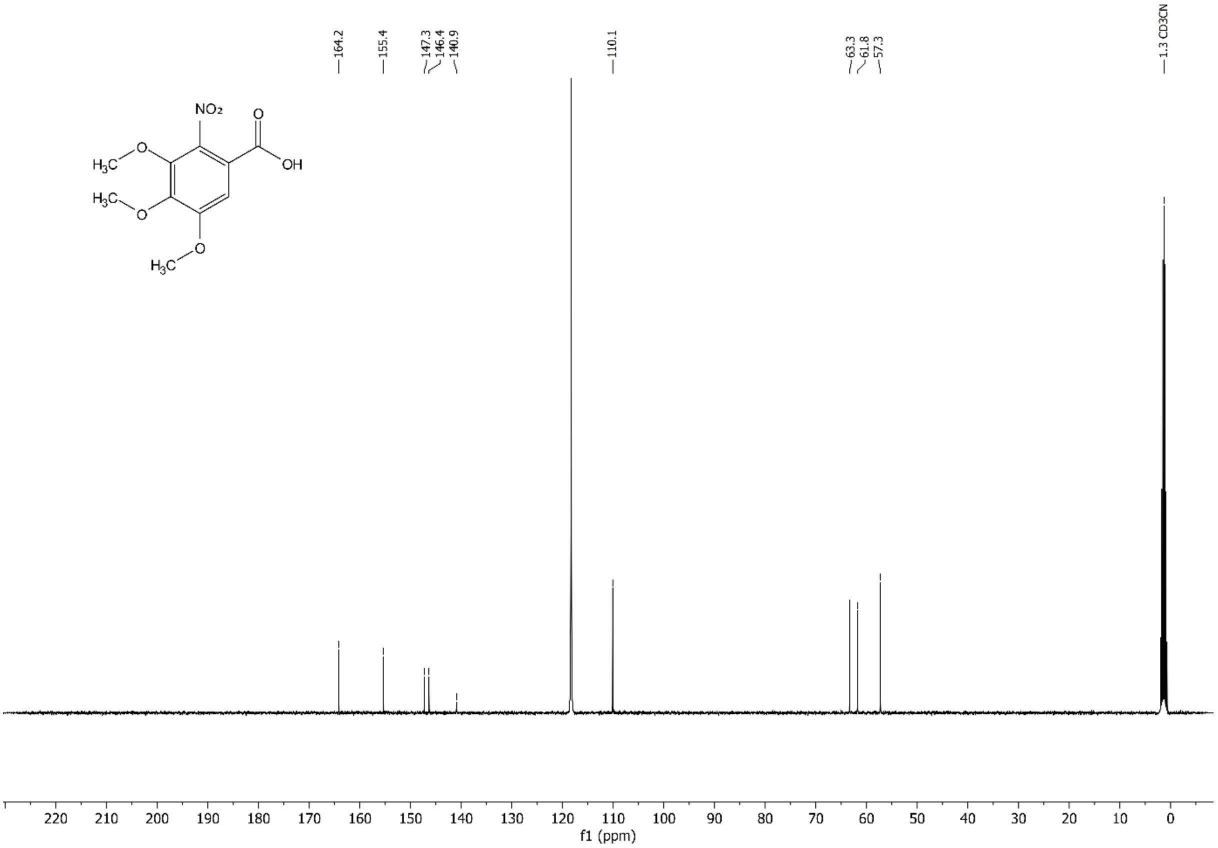

^{1}H NMR spectrum (400 MHz, CD_3CN) of **10**.

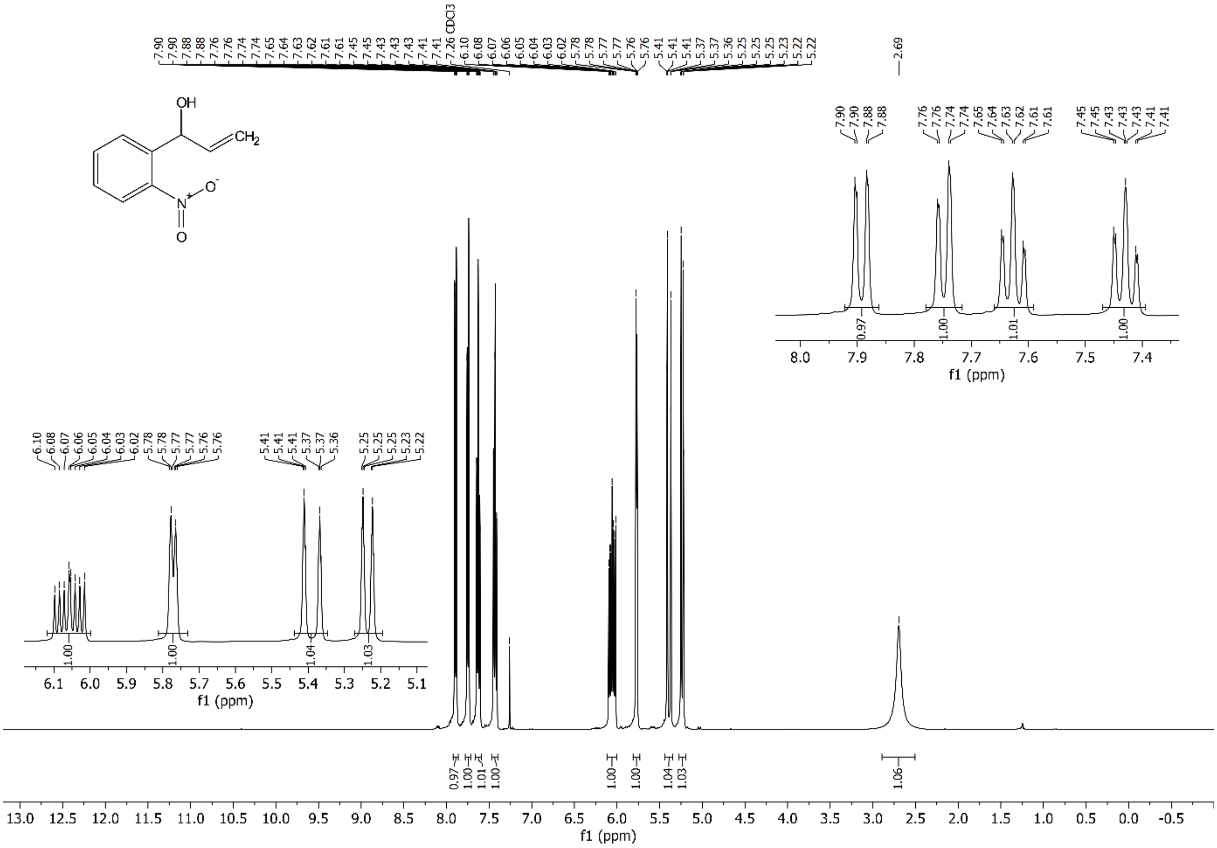

^{13}C NMR spectrum (101 MHz, CD_3CN) of **10**.

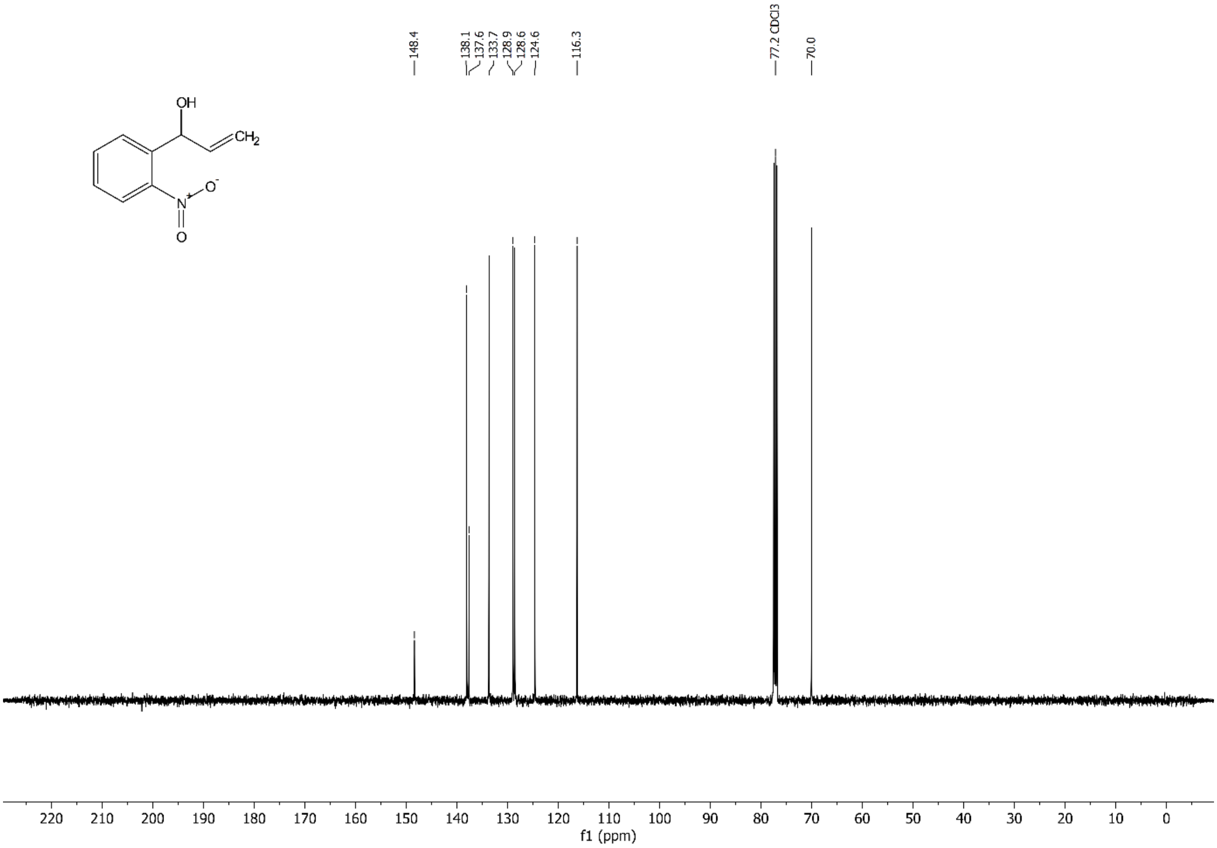

^{1}H NMR spectrum (400 MHz, CD_2Cl_2) of **11**.

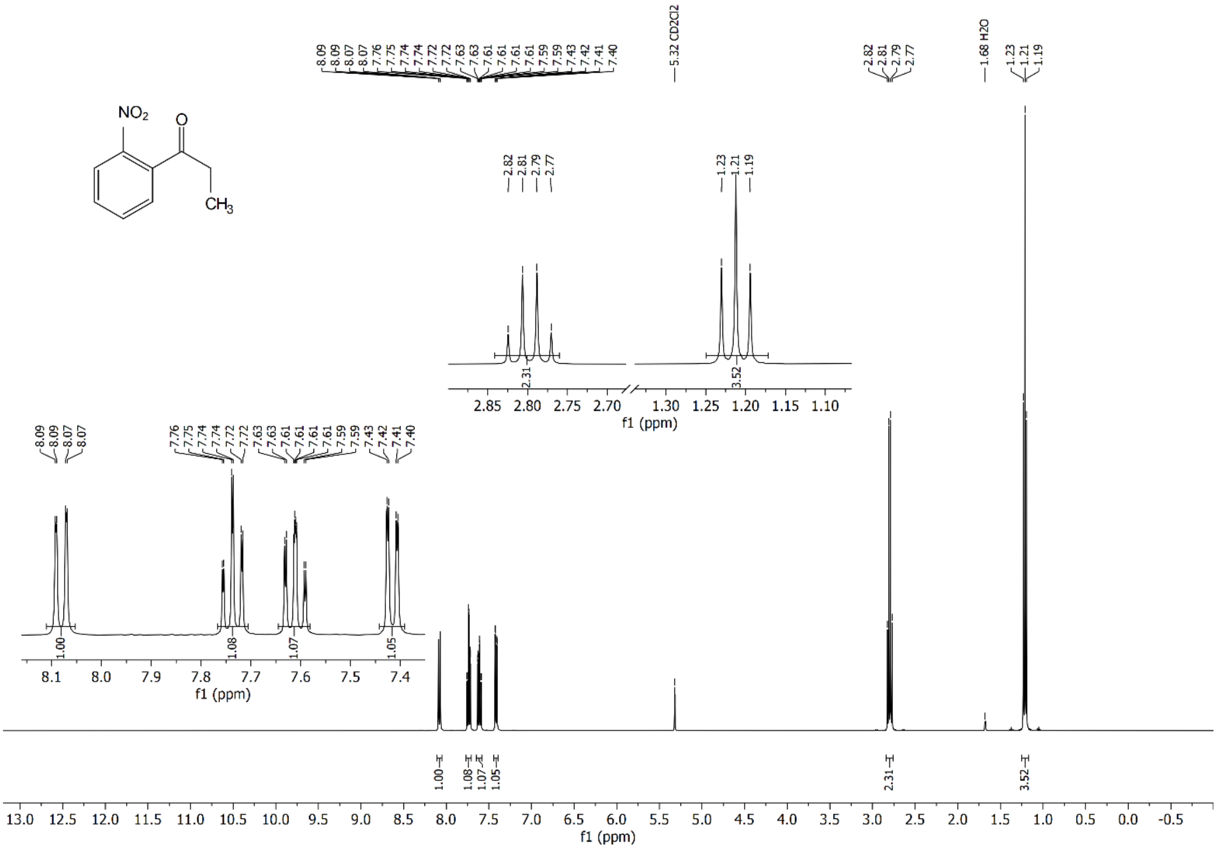

^{13}C NMR spectrum (101 MHz, CD_2Cl_2) of **11**.

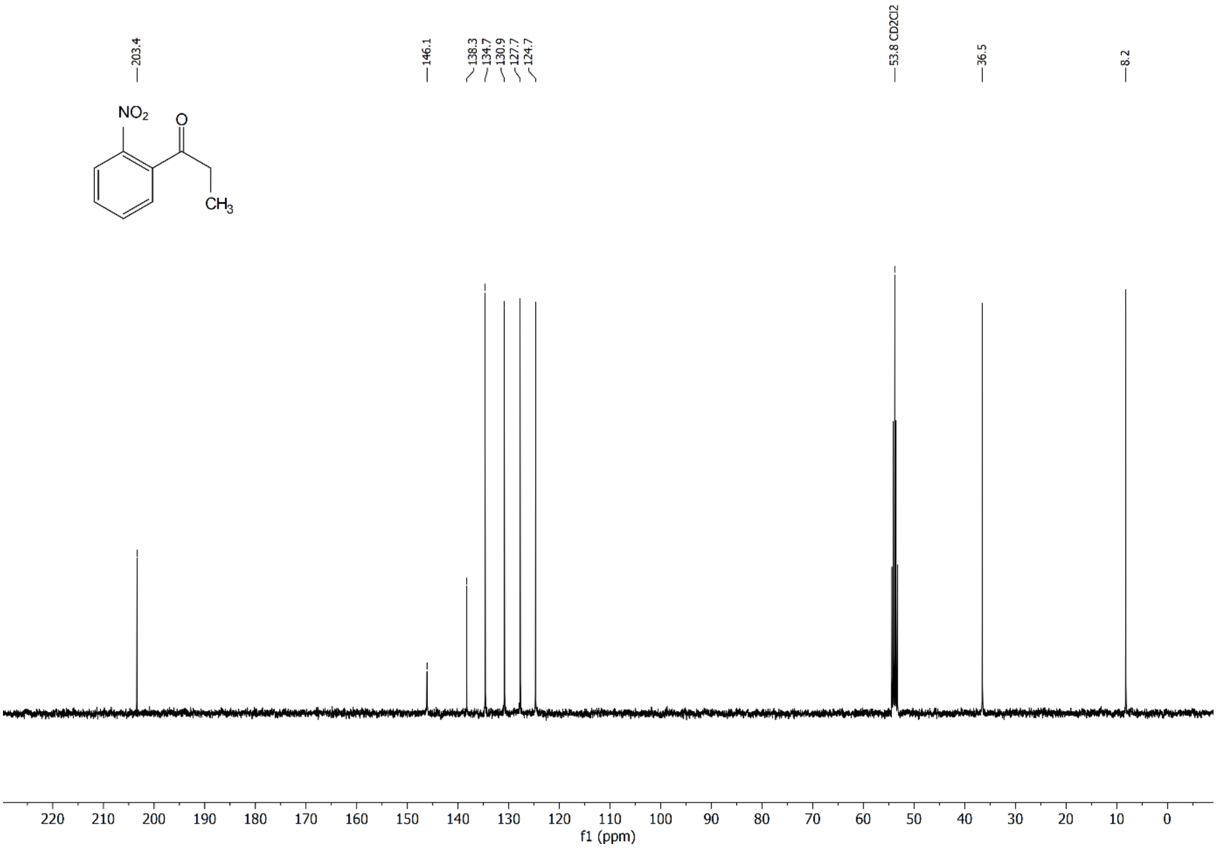

^1H NMR spectrum (400 MHz, CD_2Cl_2) of **12**.

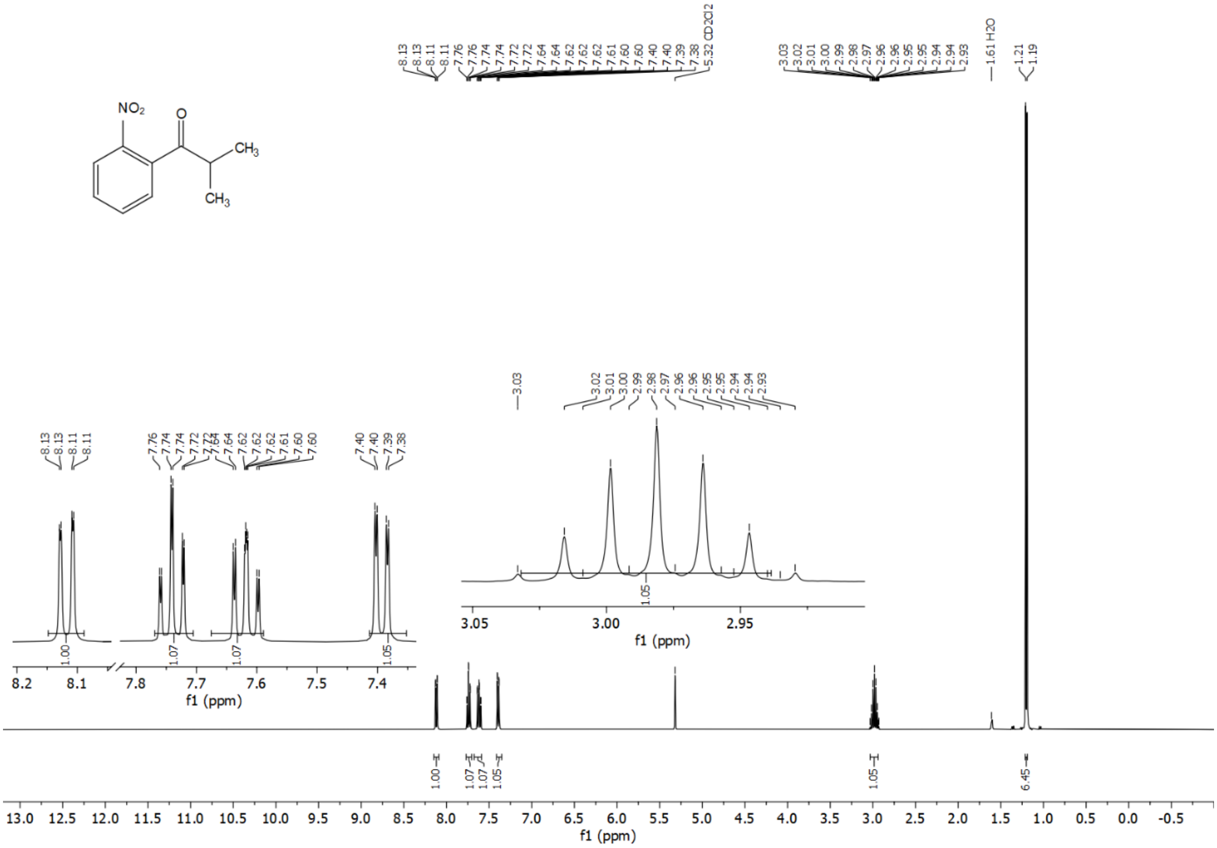

^{13}C NMR spectrum (101 MHz, CD_2Cl_2) of **12**.

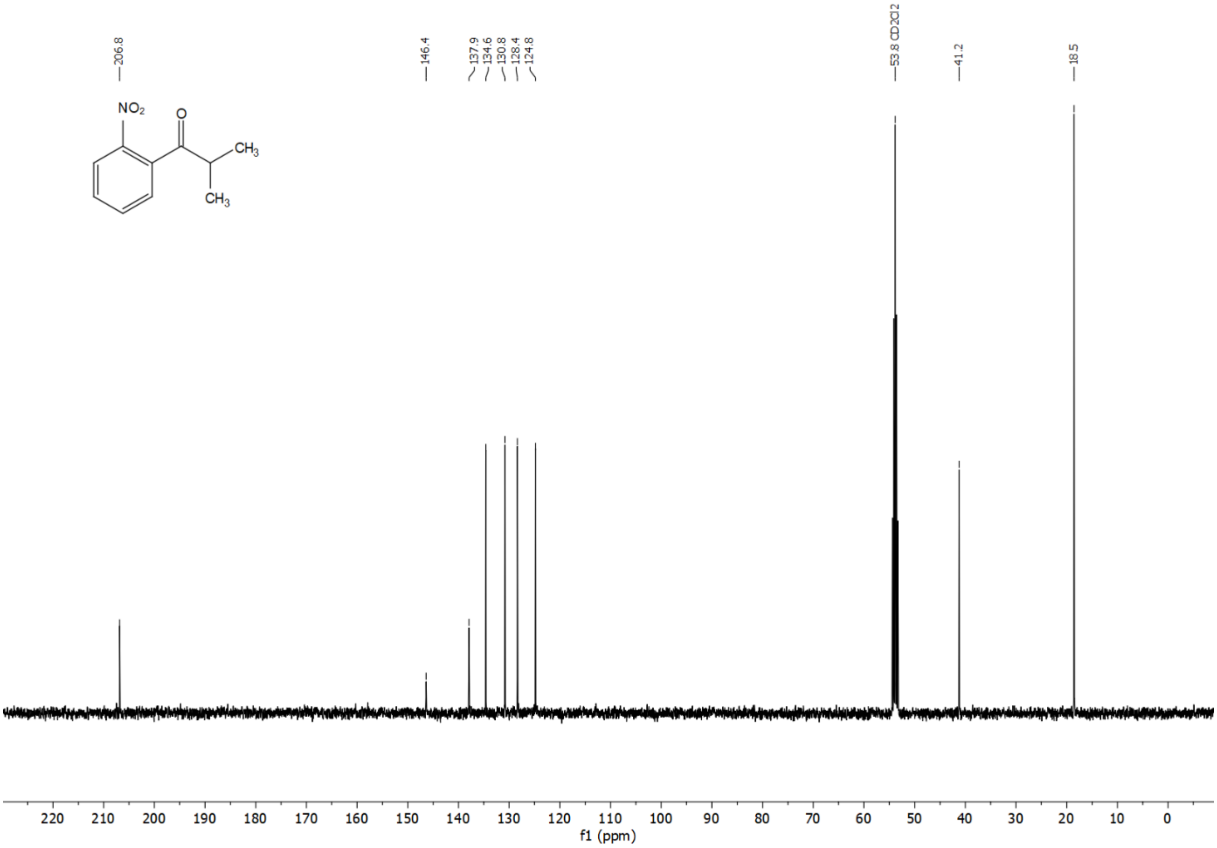

^{1}H NMR spectrum (400 MHz, CD_2Cl_2) of **13**.

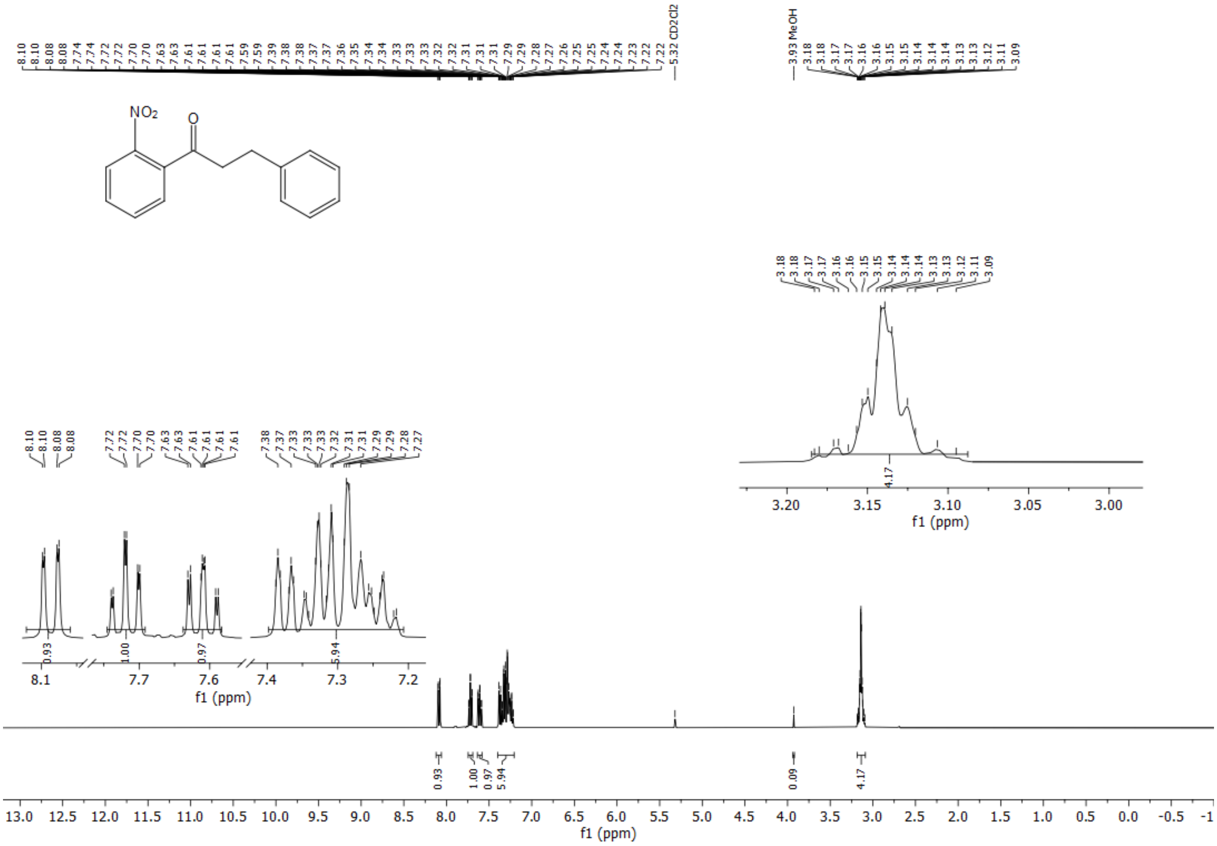

^{13}C NMR spectrum (101 MHz, CD_2Cl_2) of **13**.

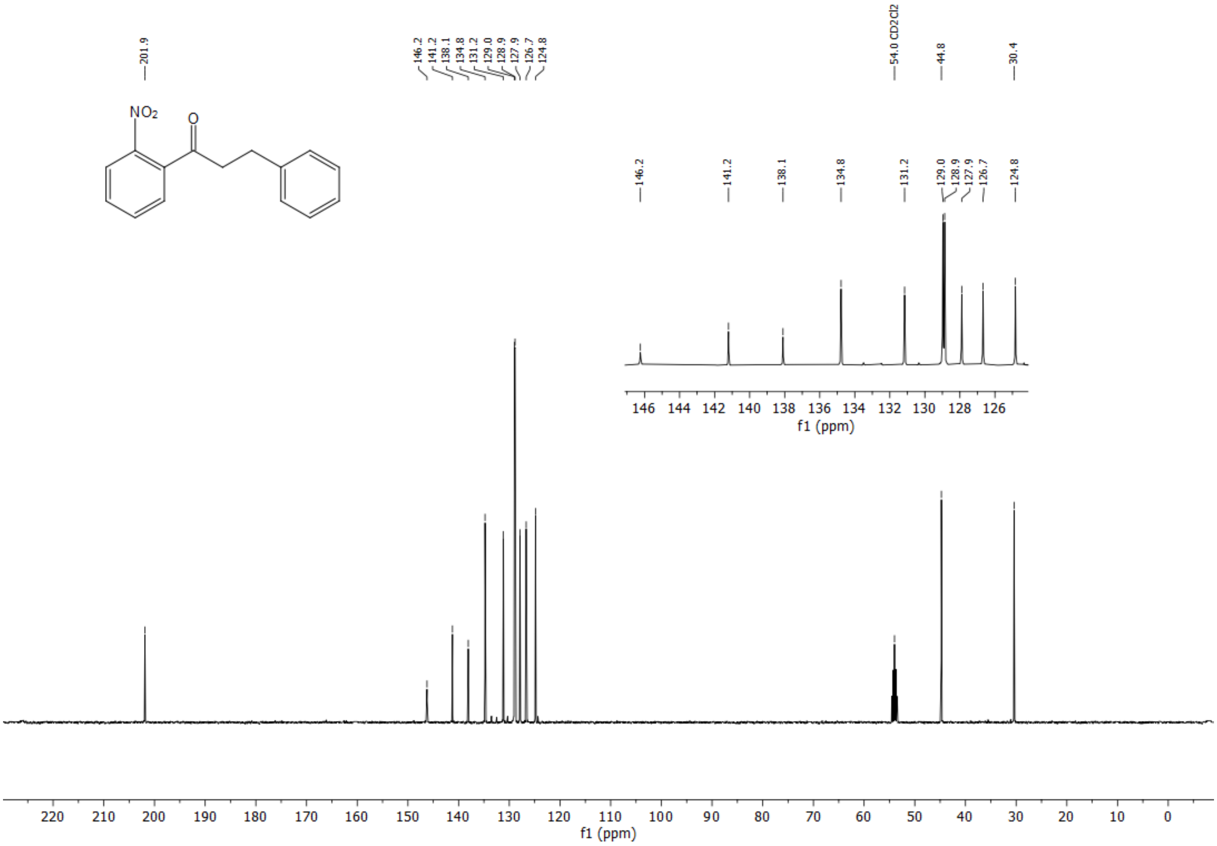

^{1}H NMR spectrum (400 MHz, CD_2Cl_2) of **14**.

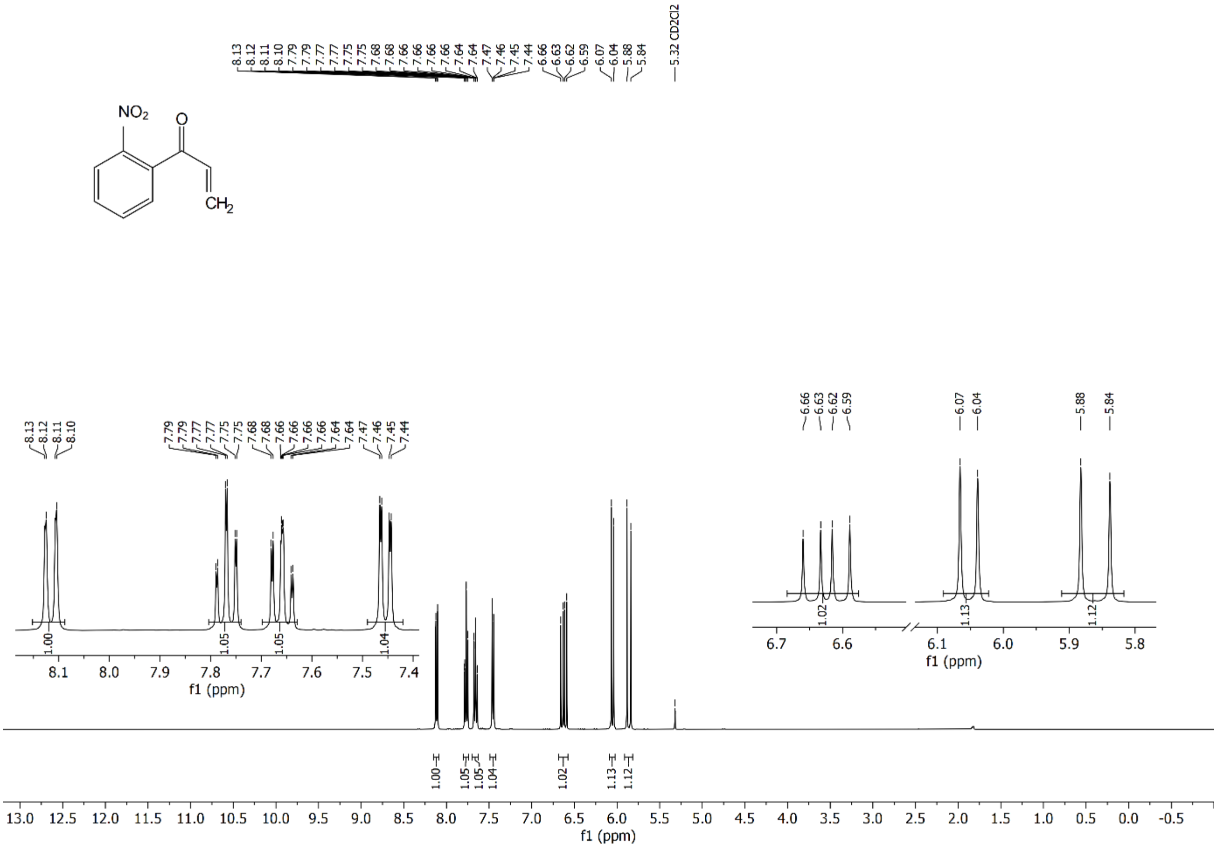

^{13}C NMR spectrum (101 MHz, CD_2Cl_2) of **14**.

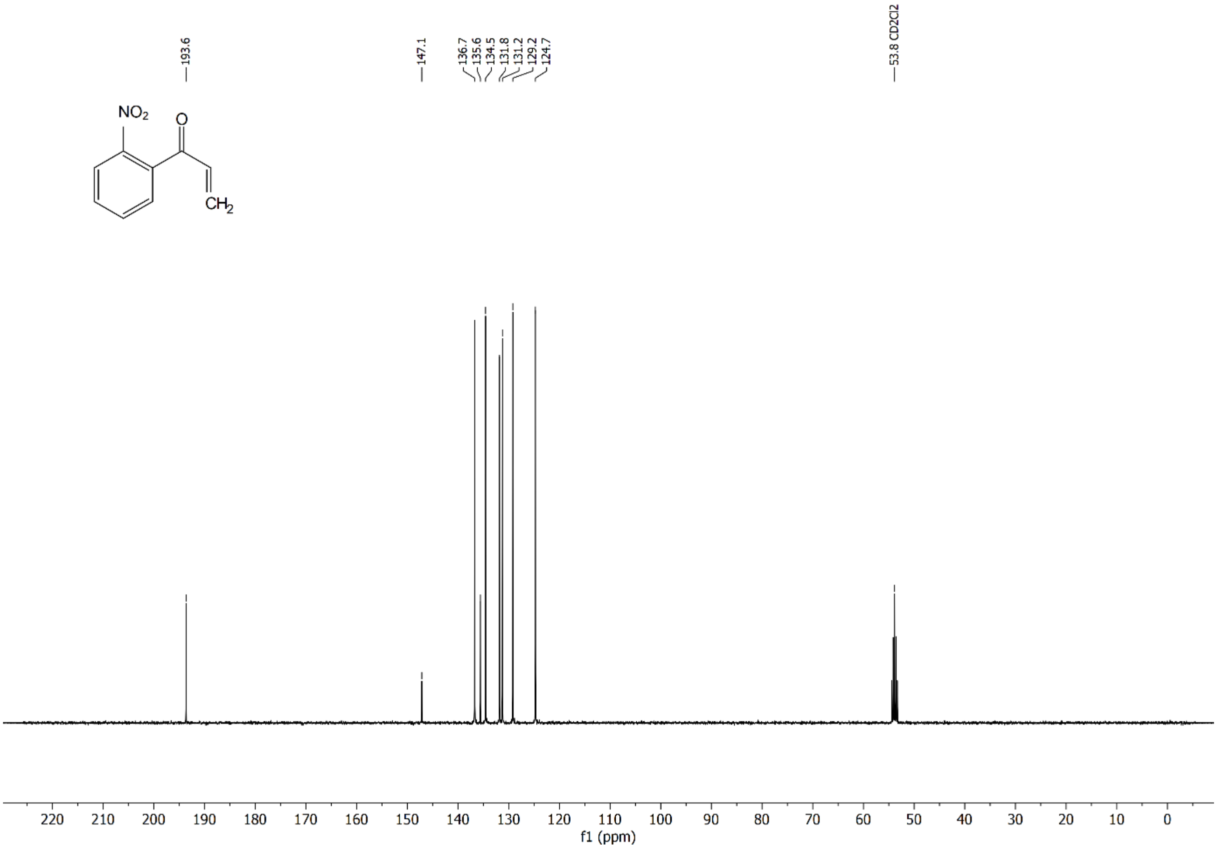

^{1}H NMR spectrum (400 MHz, $CDCl_3$) of **15**.

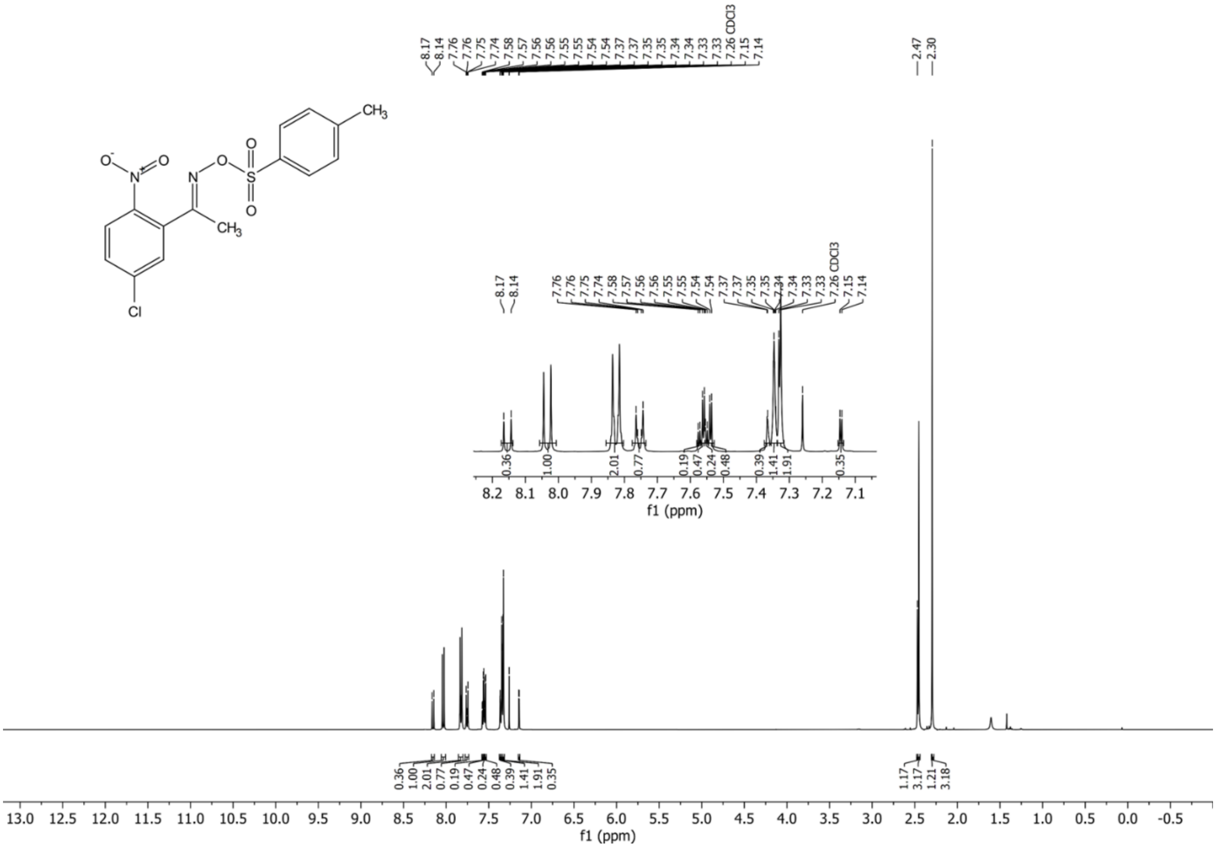

^{13}C NMR spectrum (101 MHz, $CDCl_3$) of **15.**

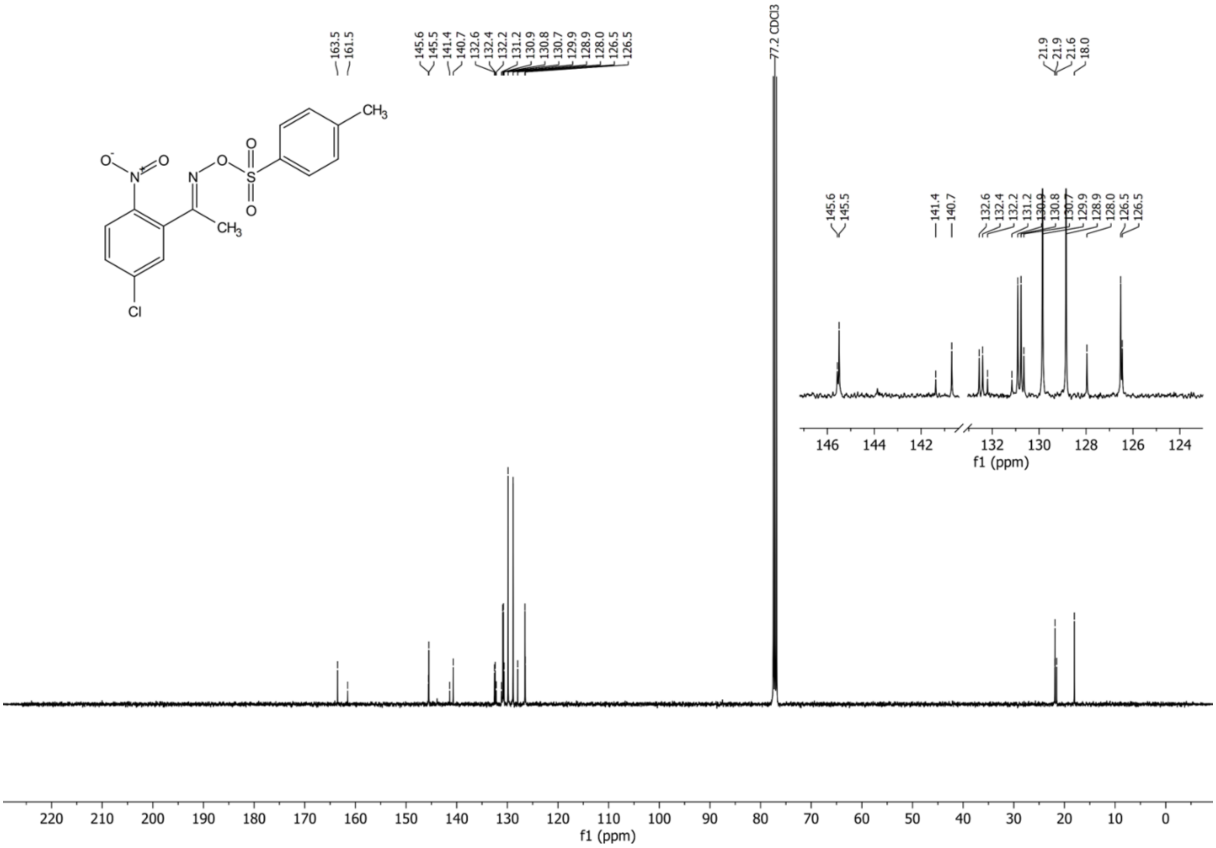

^{1}H NMR spectrum (400 MHz, CD_3CN) of **16.**

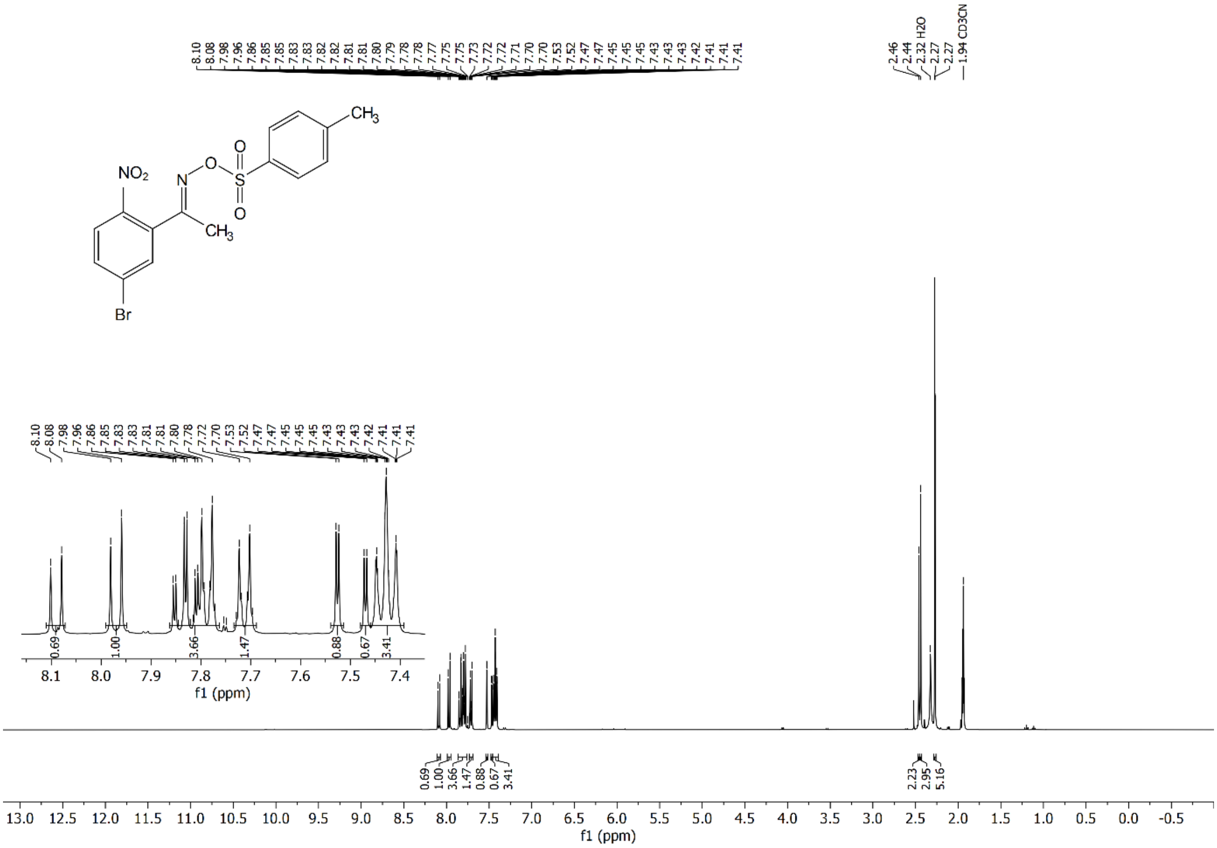

^{13}C NMR spectrum (101 MHz, CD_3CN) of **16**.

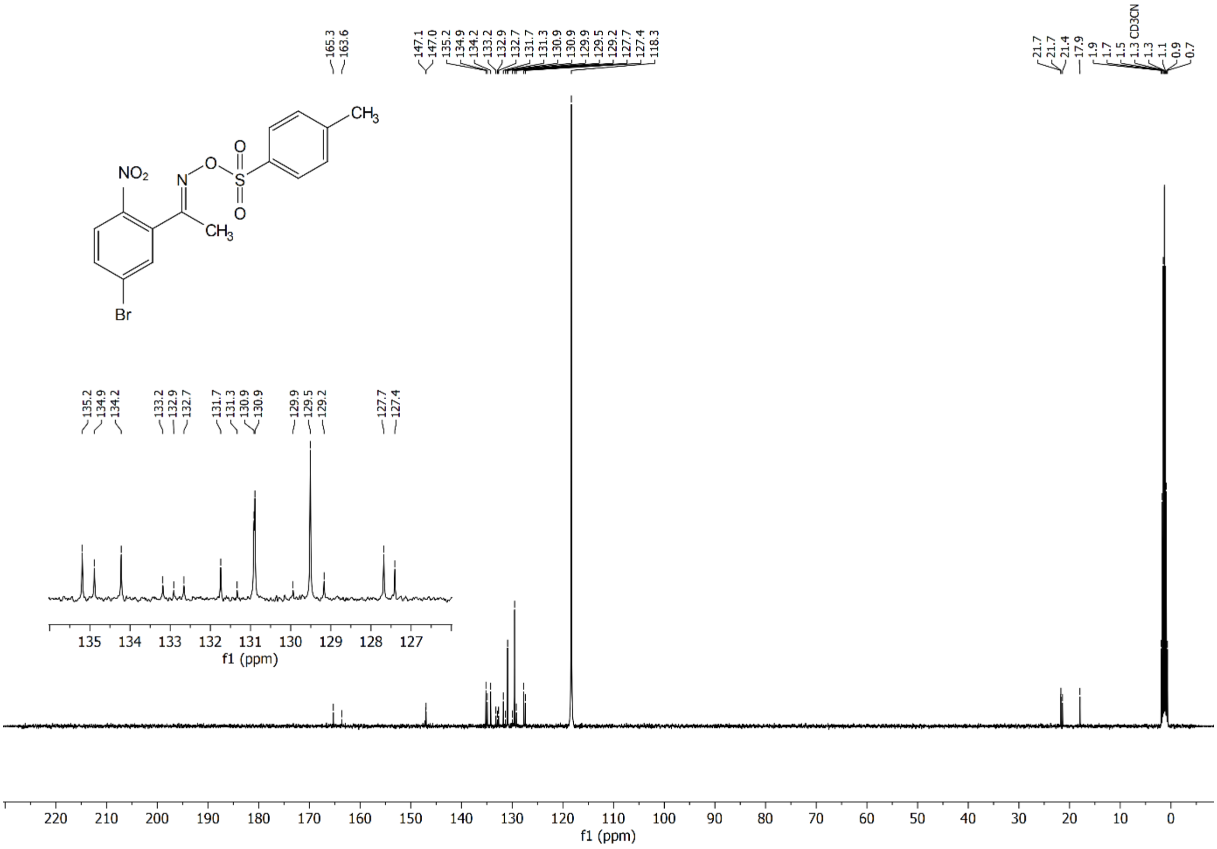

^{1}H NMR spectrum (400 MHz, CD_3CN) of **17**.

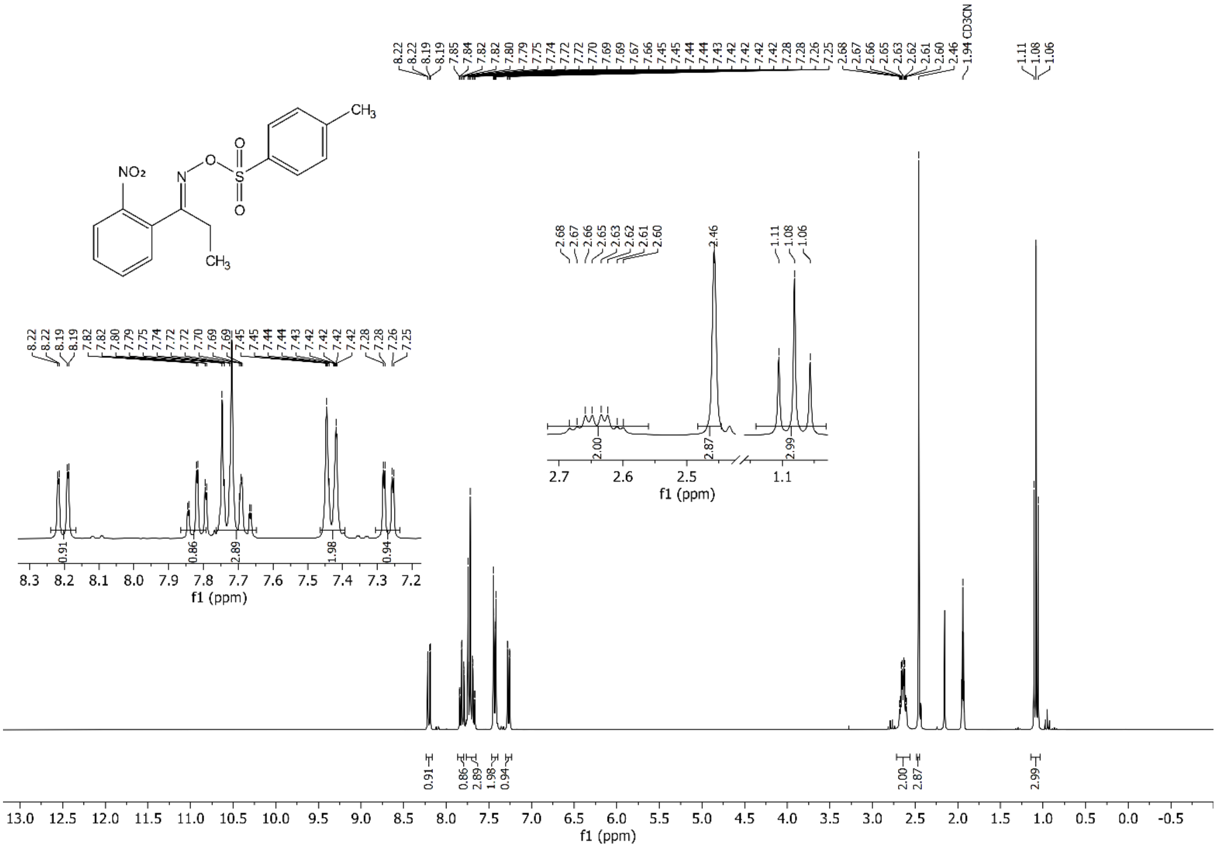

^{13}C NMR spectrum (101 MHz, CD_3CN) of **17**.

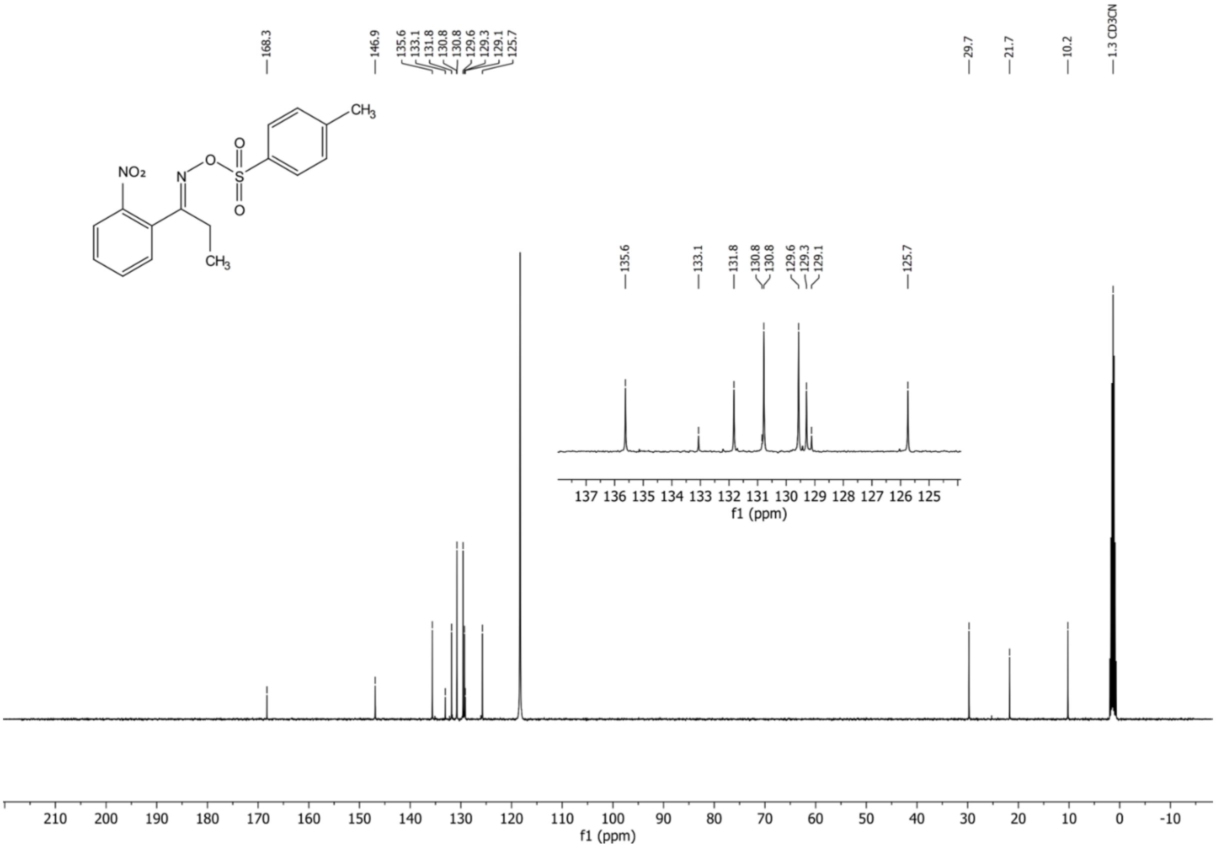

^{1}H NMR spectrum (400 MHz, CD_2Cl_2) of **18.**

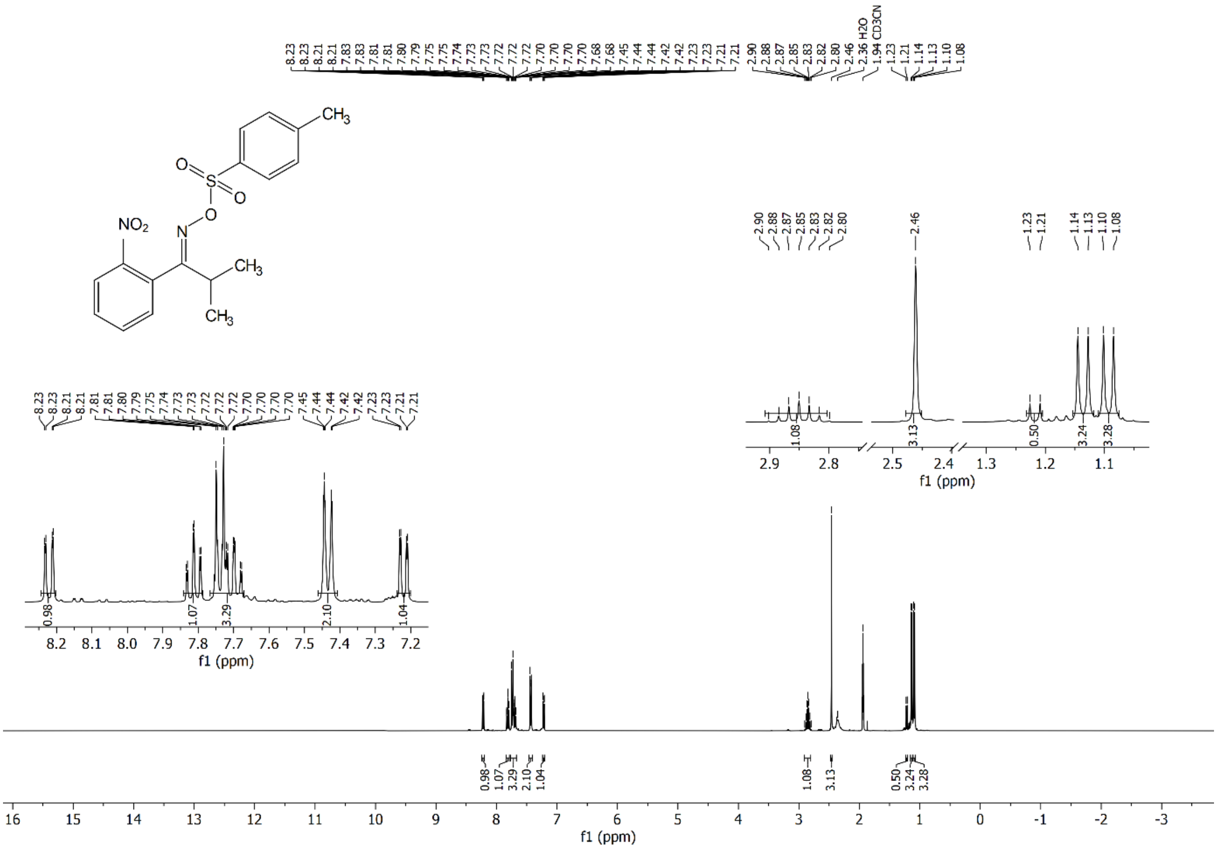

^{13}C NMR spectrum (101 MHz, CD_2Cl_2) of **18**.

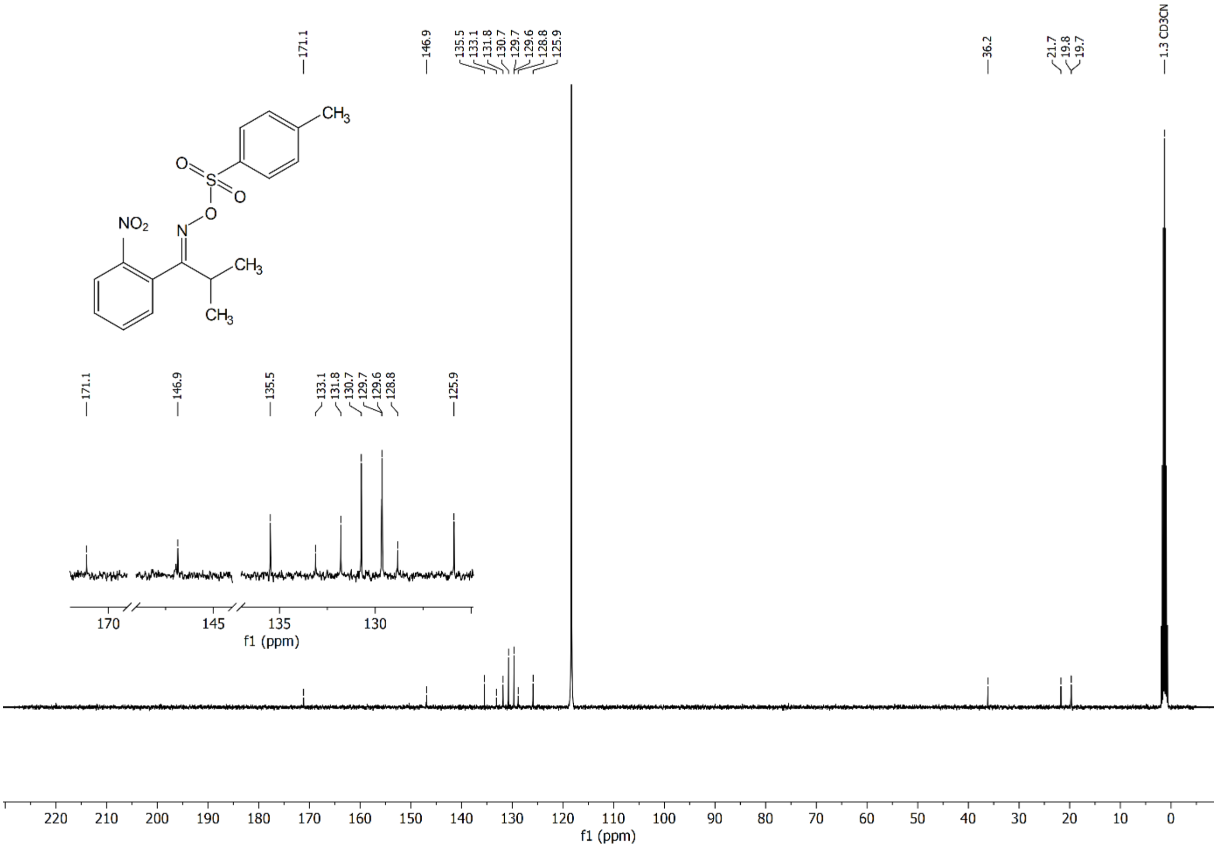

^{1}H NMR spectrum (400 MHz, CD_3CN) of **19.**

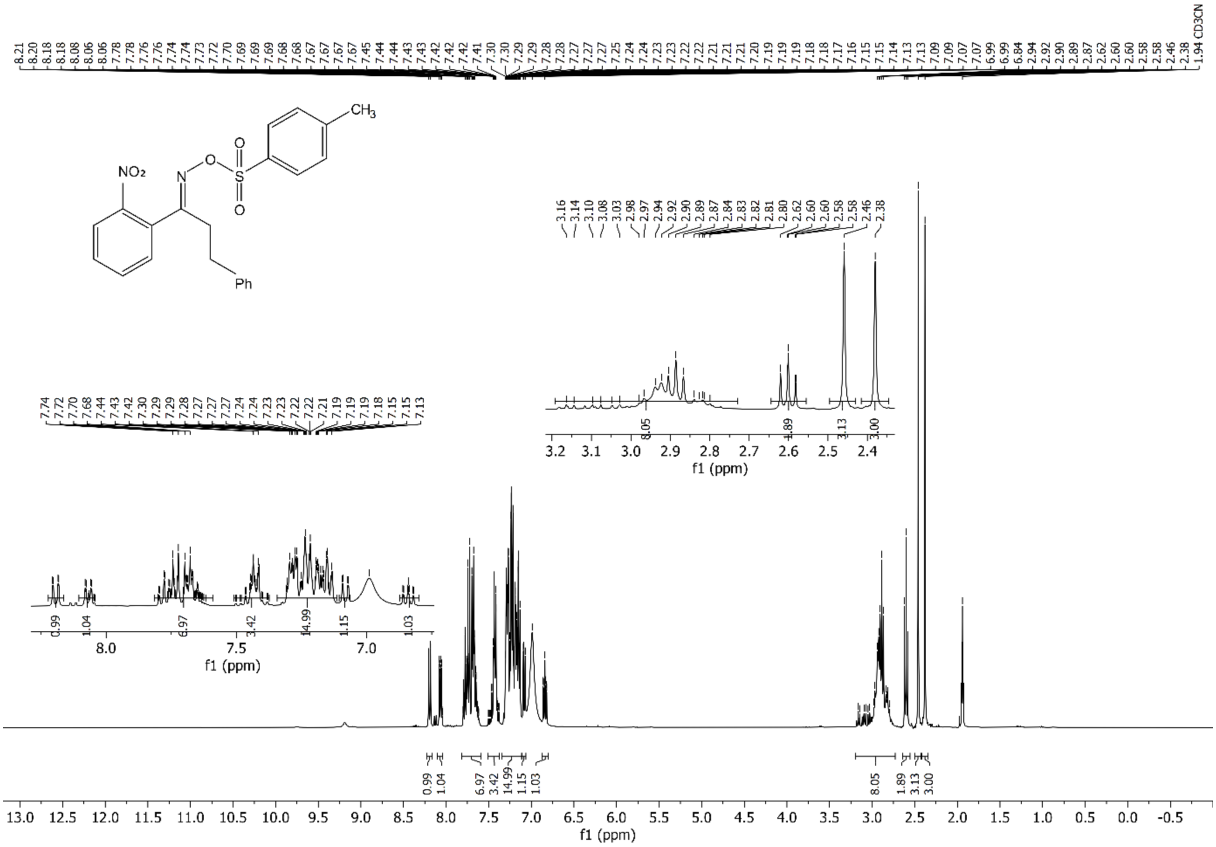

^{13}C NMR spectrum (101 MHz, CD_3CN) of **19**.

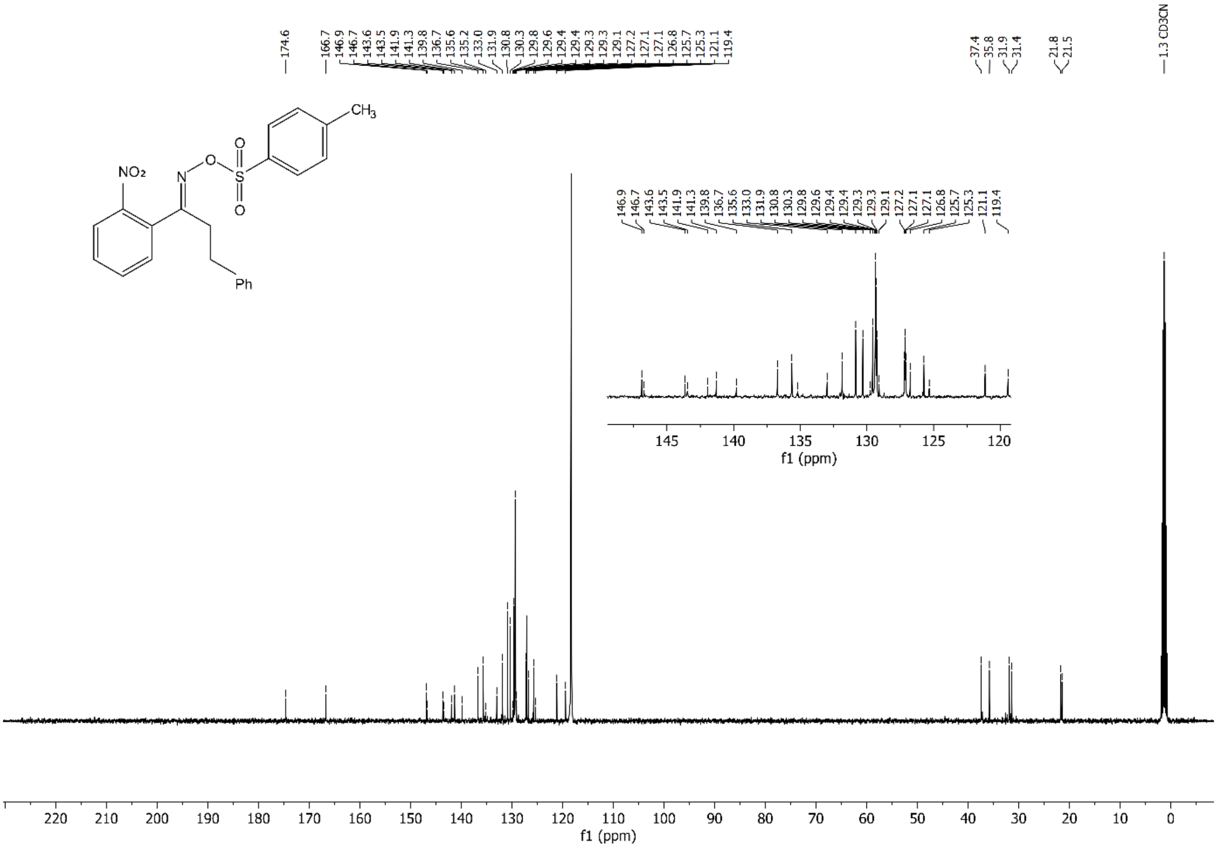

^{1}H NMR spectrum (400 MHz, CD_3CN) of **20**.

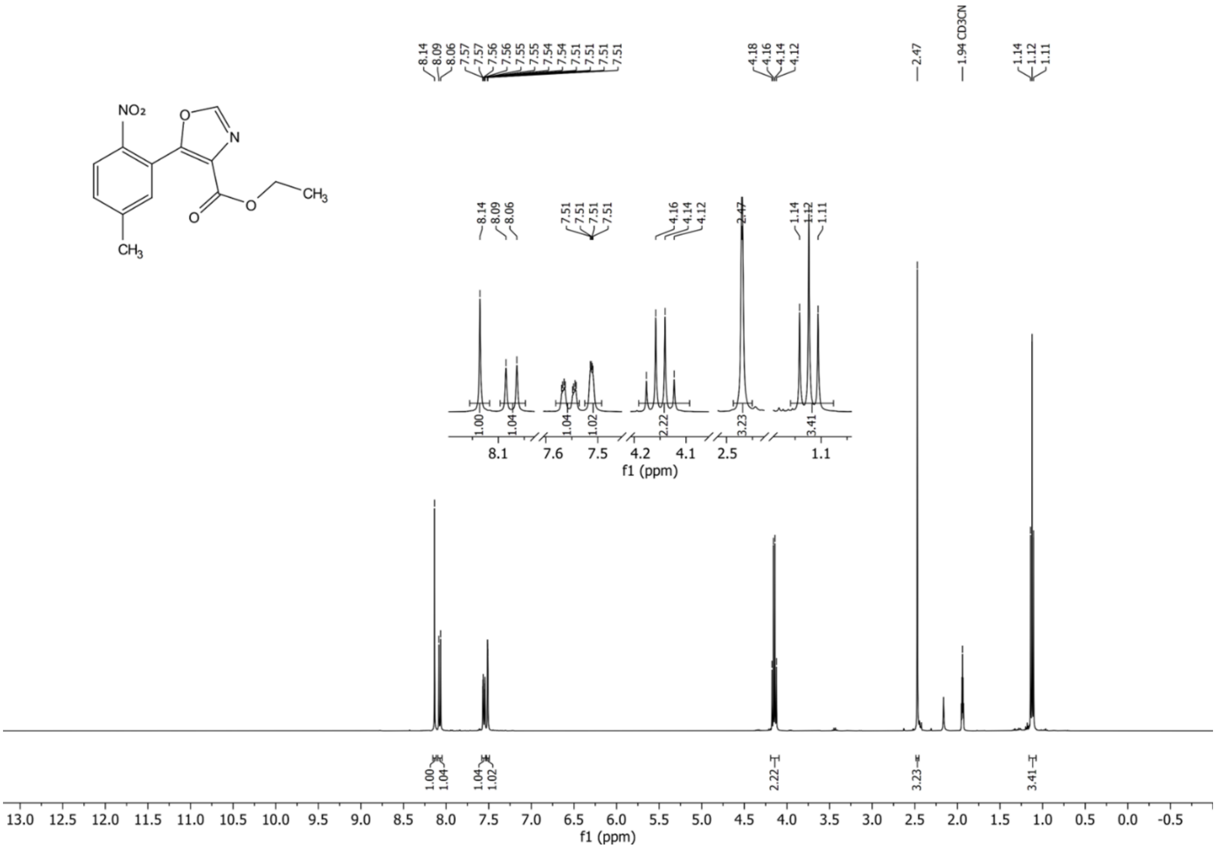

^{13}C NMR spectrum (101 MHz, CD_3CN) of **20.**

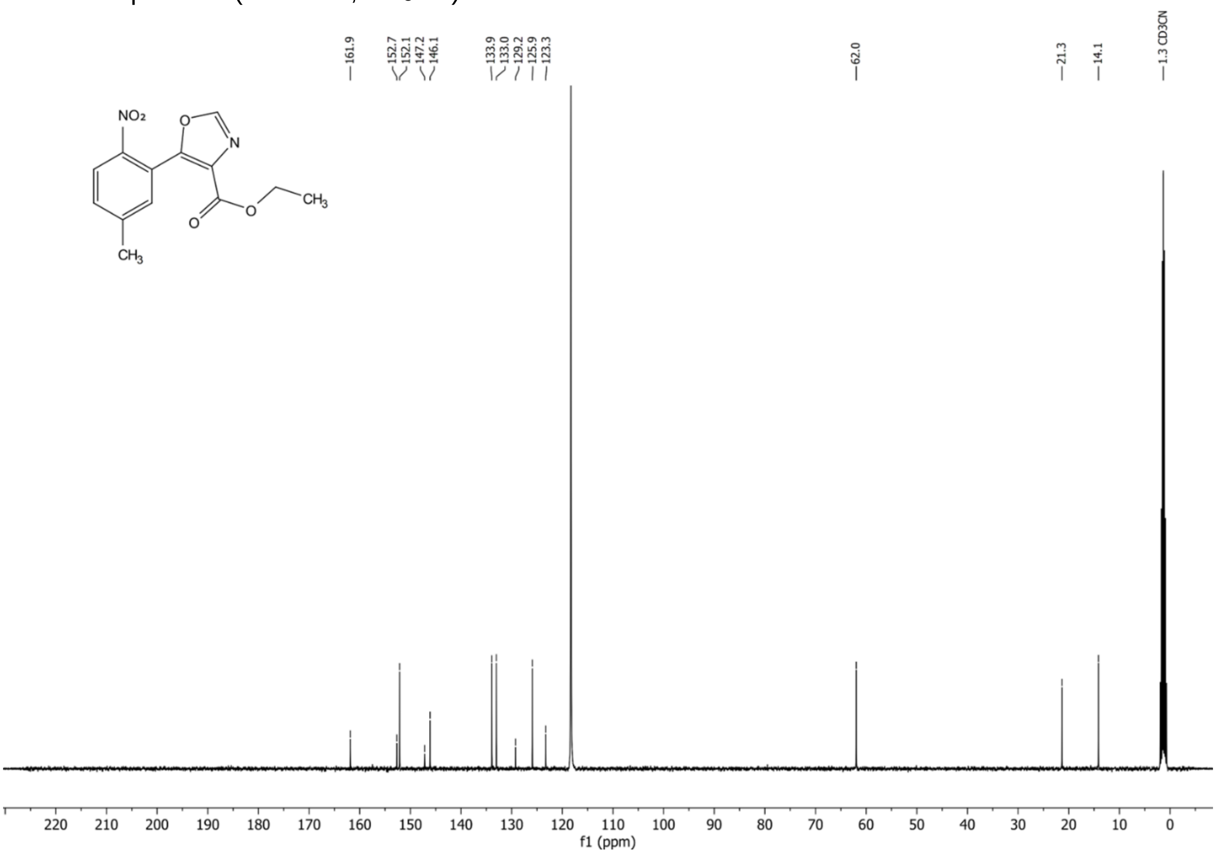

^{1}H NMR spectrum (400 MHz, CD_3CN) of **21**.

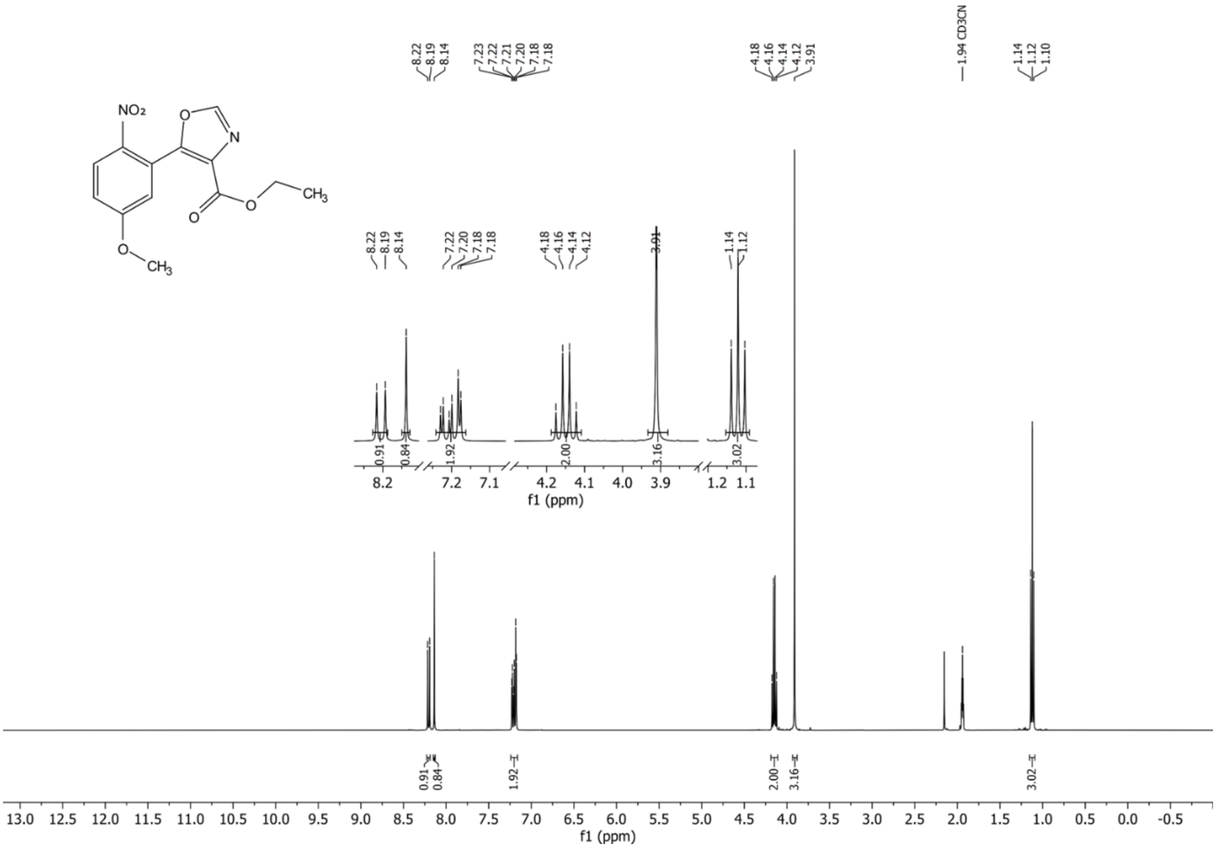

^{13}C NMR spectrum (101 MHz, CD_3CN) of **21.**

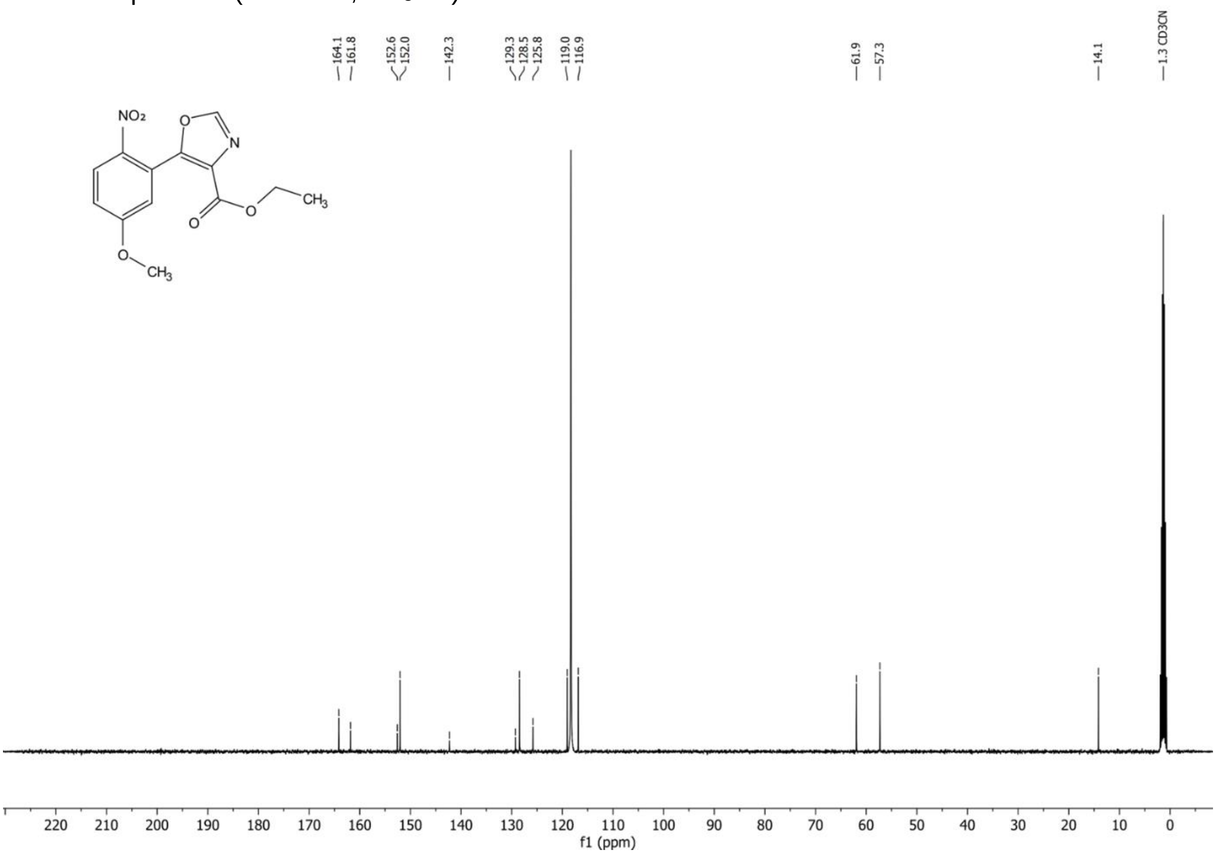

^{1}H NMR spectrum (400 MHz, $CDCl_3$) of **22**.

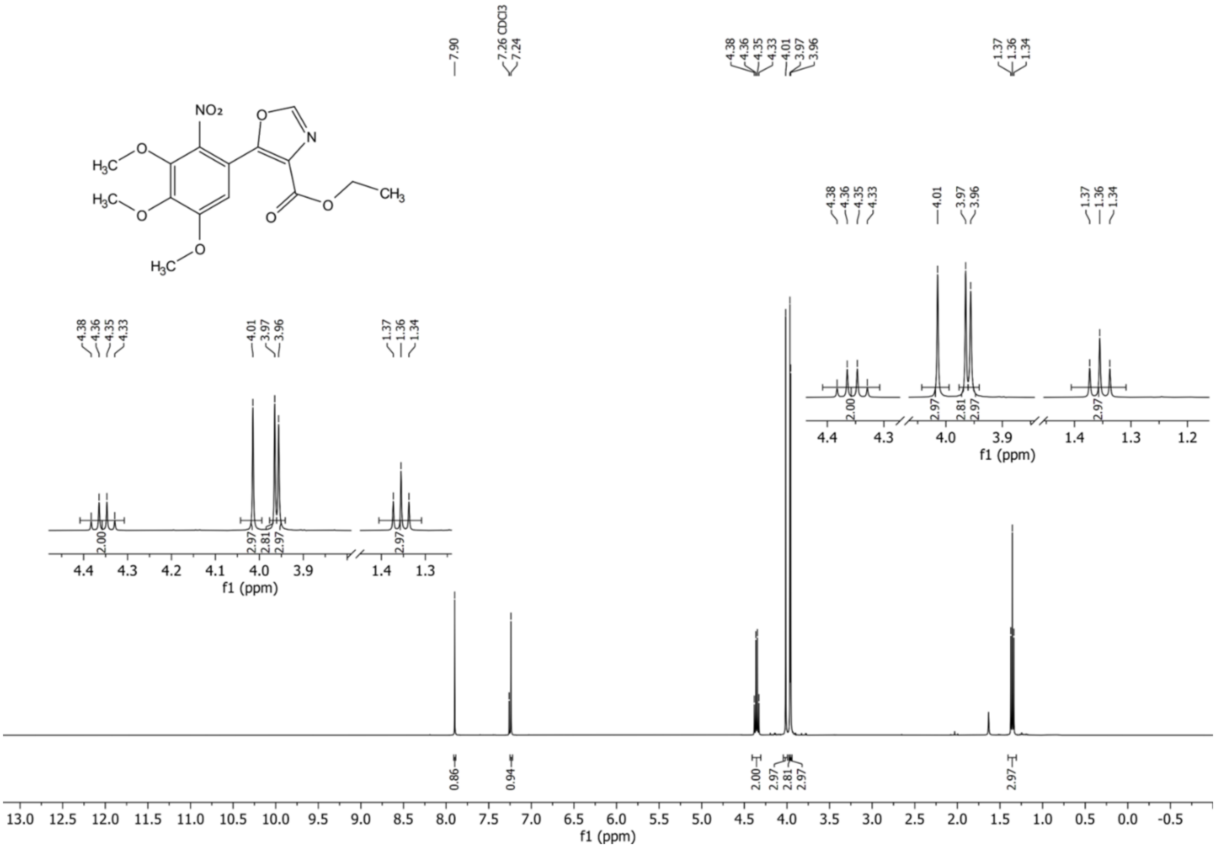

^{13}C NMR spectrum (101 MHz, $CDCl_3$) of **22.**

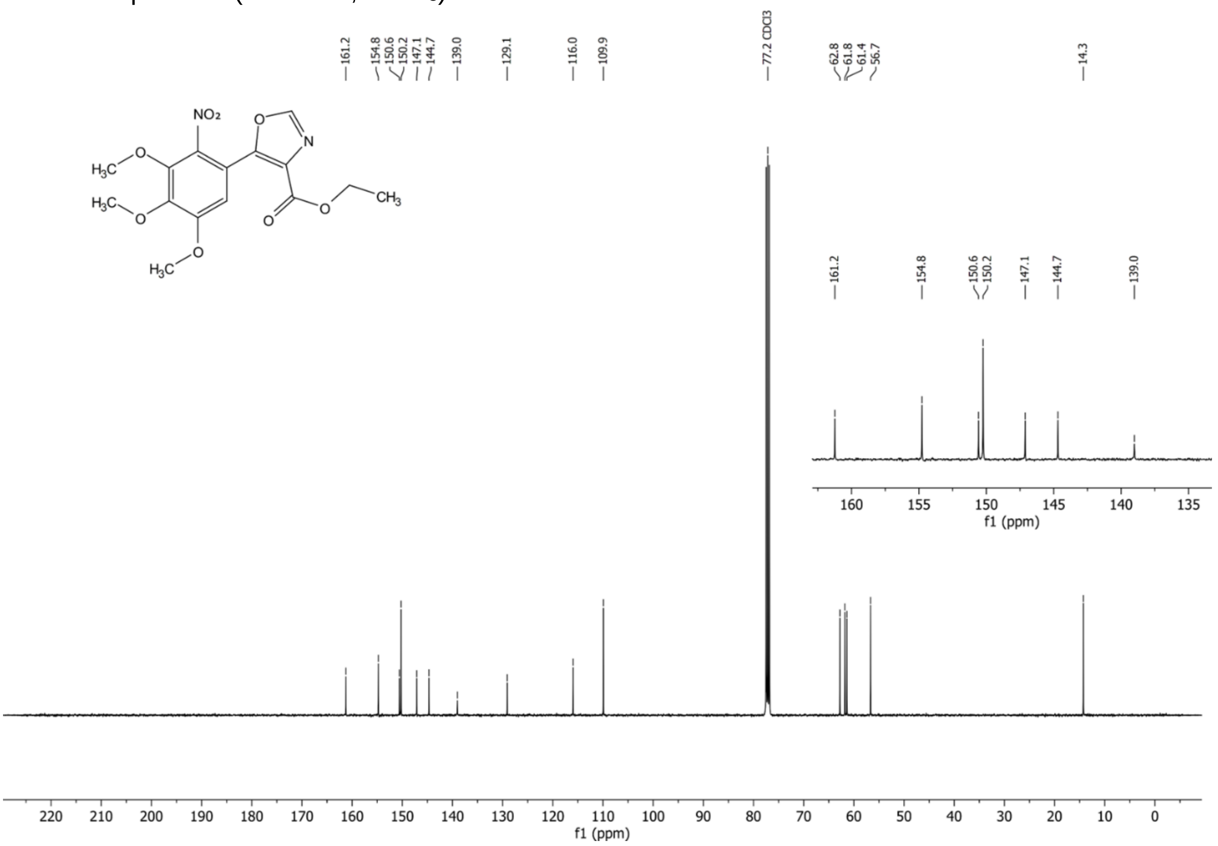

^{1}H NMR spectrum (400 MHz, CD_3CN) of **23**.

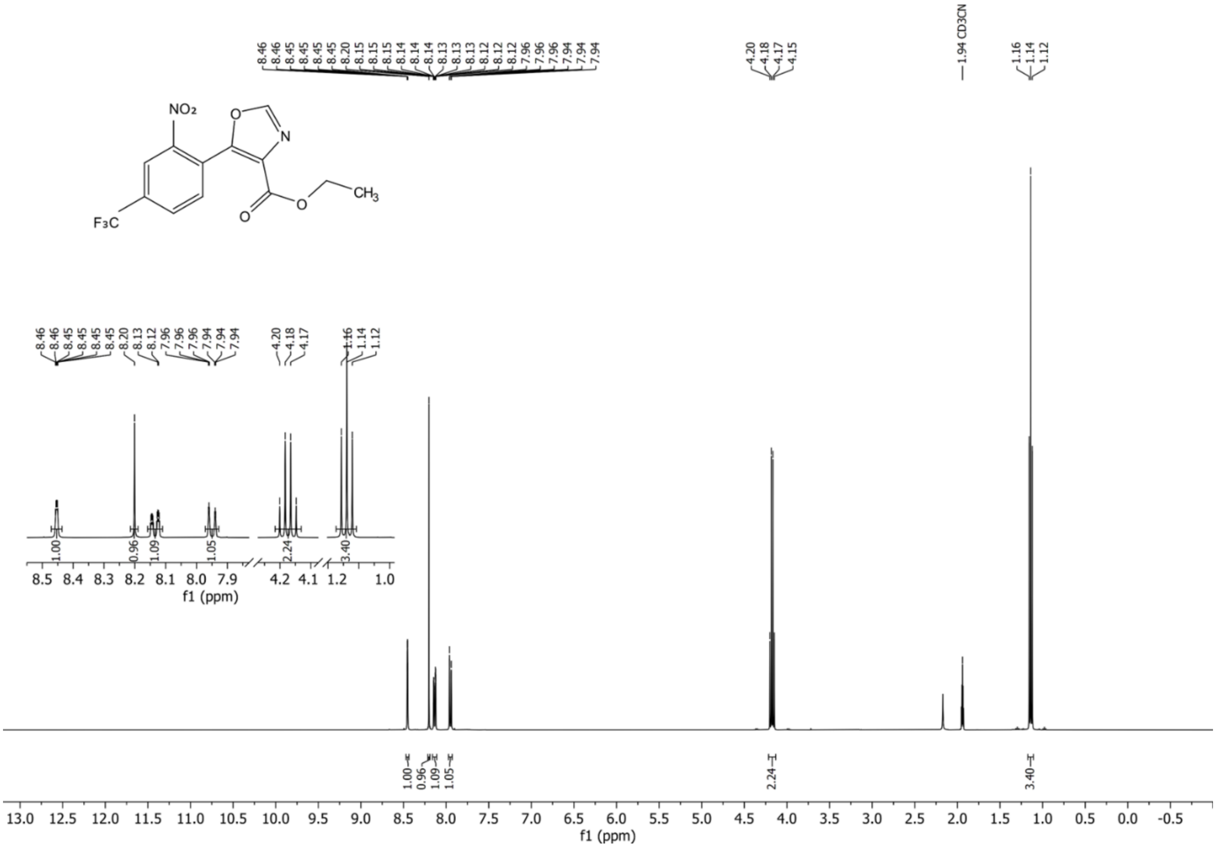

^{13}C NMR spectrum (101 MHz, CD_3CN) of **23**.

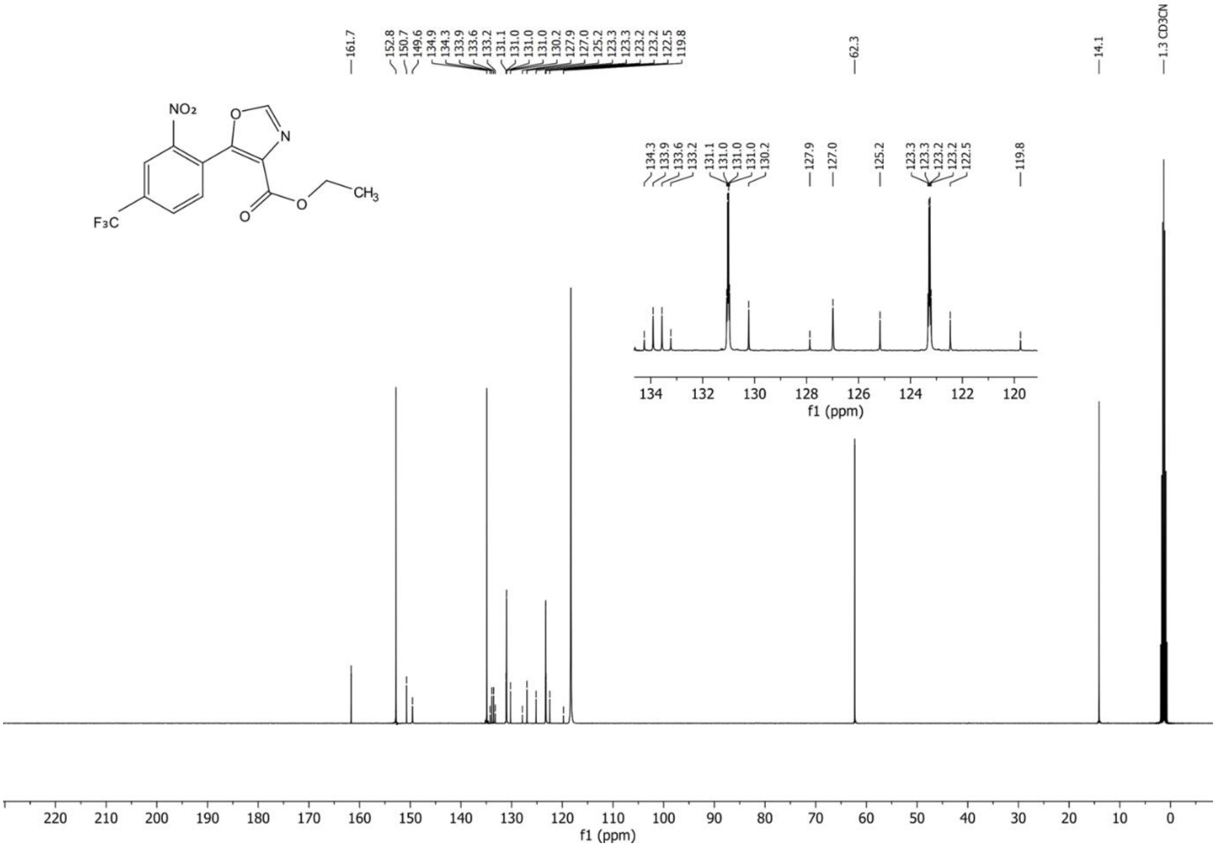

^{19}F NMR spectrum (376 MHz, CD_3CN) of **23**.

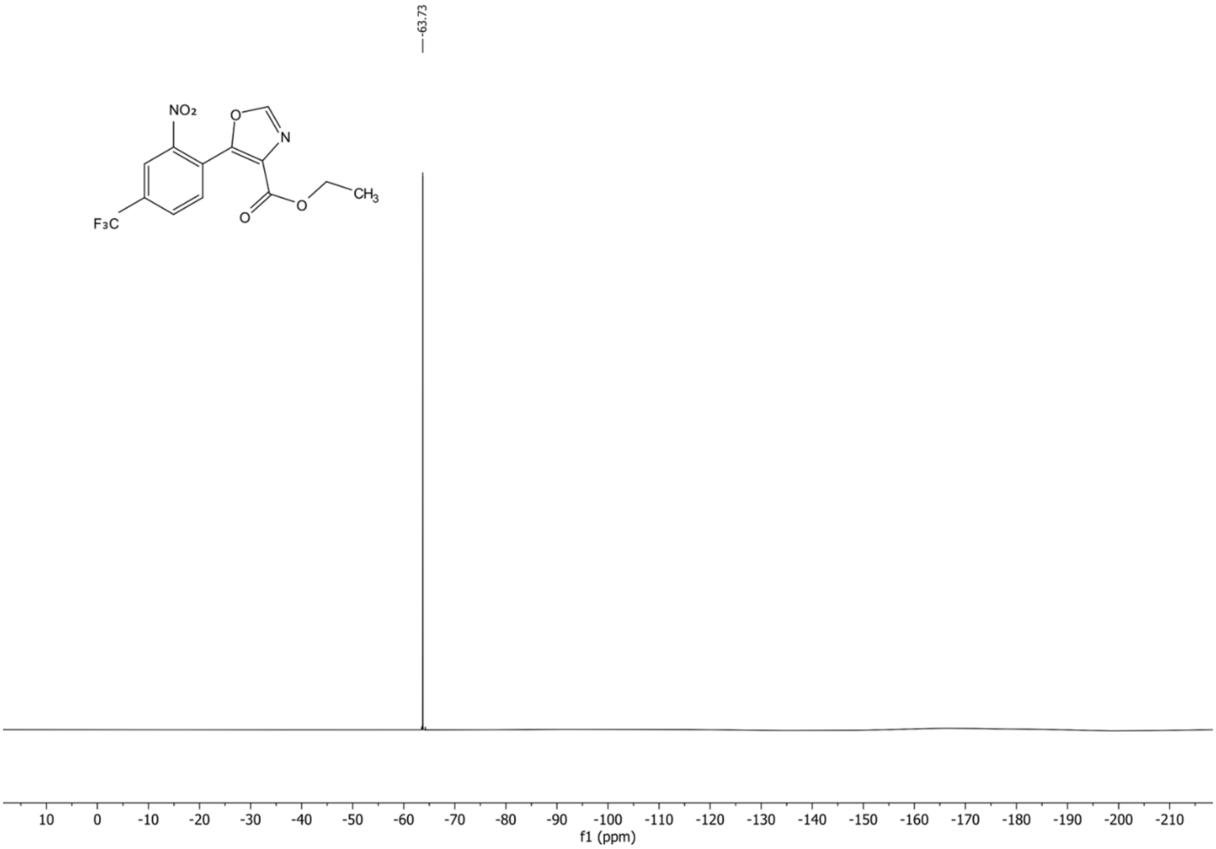

^{1}H NMR spectrum (400 MHz, $CDCl_3$) of **24**.

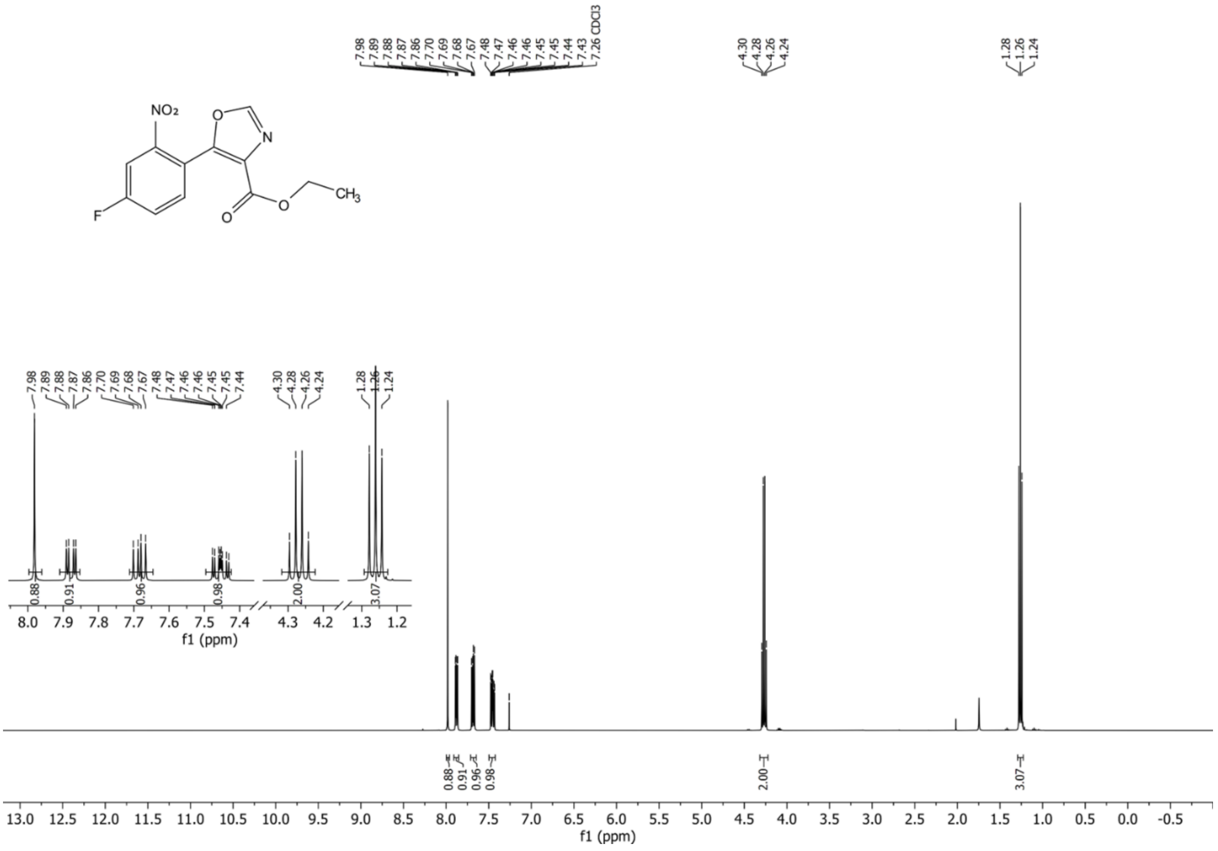

^{13}C NMR spectrum (101 MHz, $CDCl_3$) of **24**.

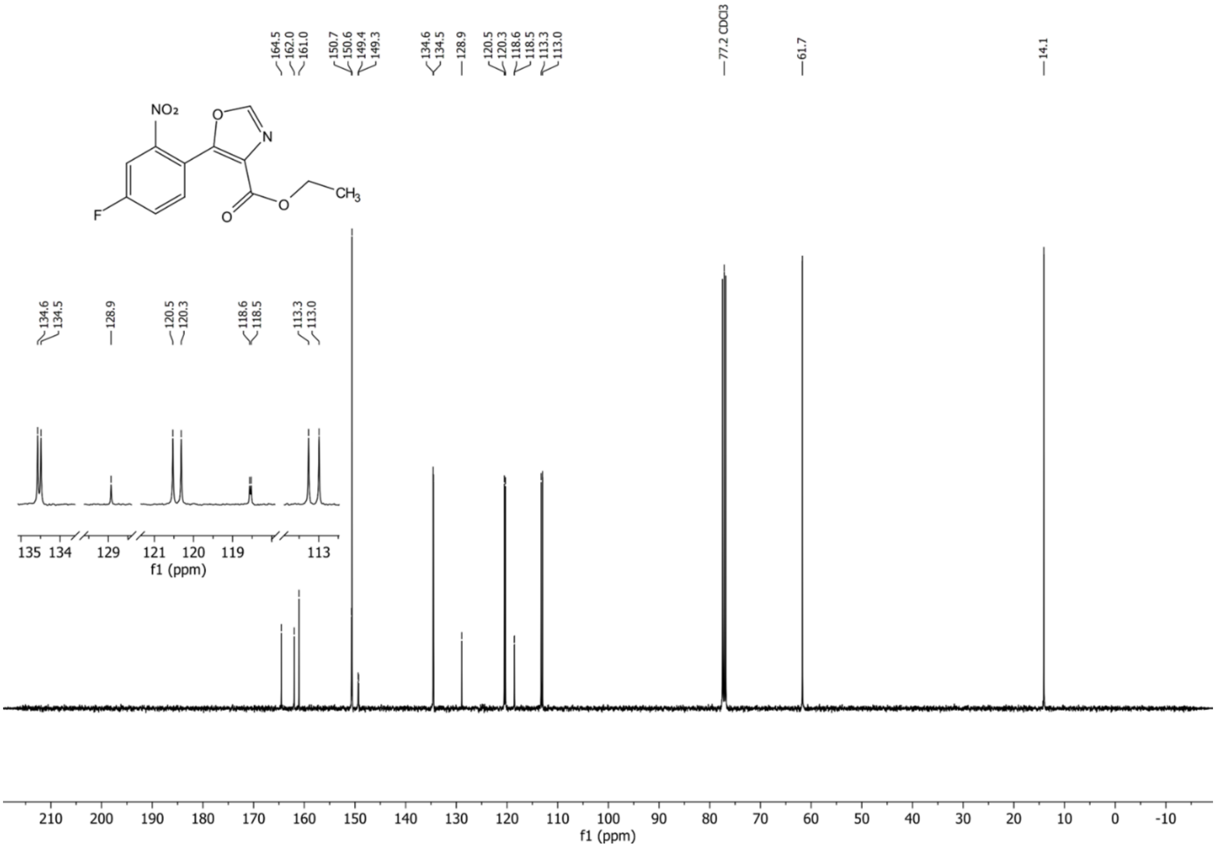

^{19}F NMR spectrum (376 MHz, CD_3CN) of **24.**

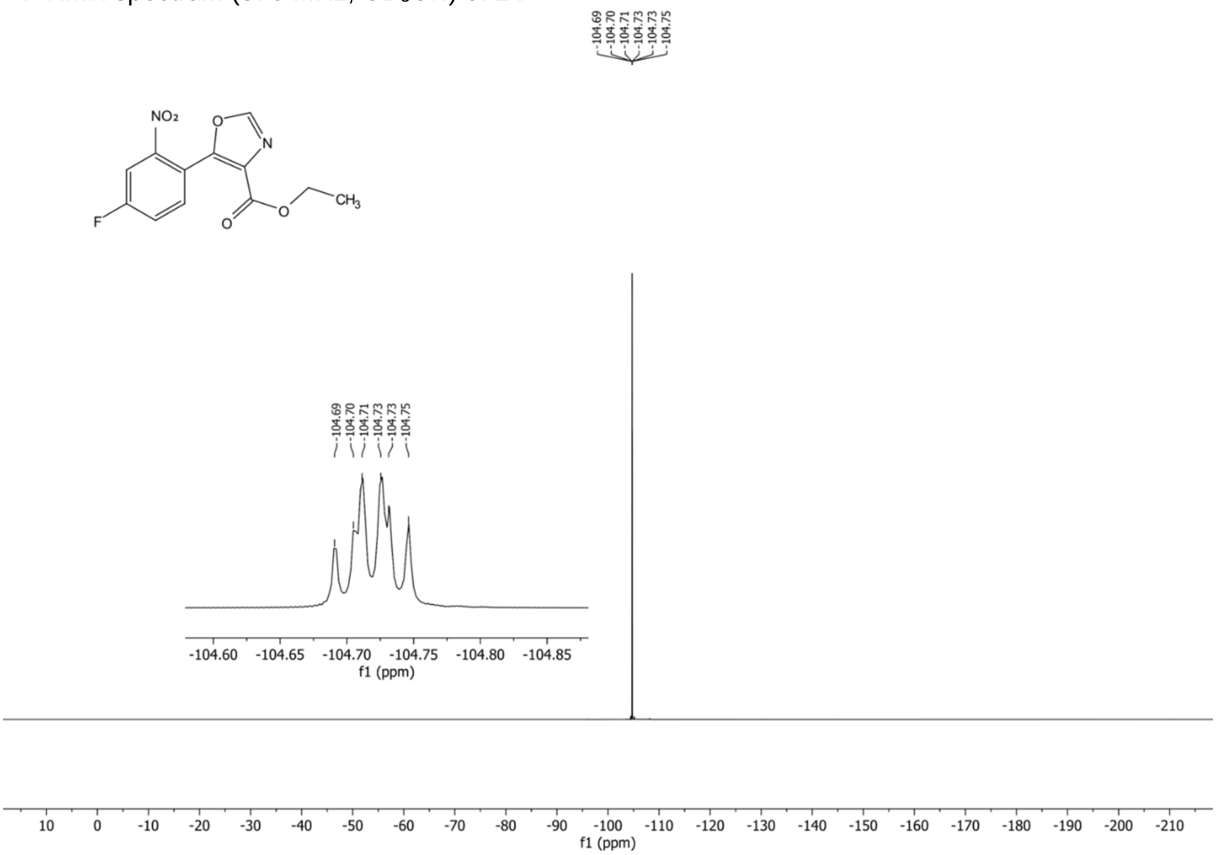

^{1}H NMR spectrum (400 MHz, CD_3CN) of **25**.

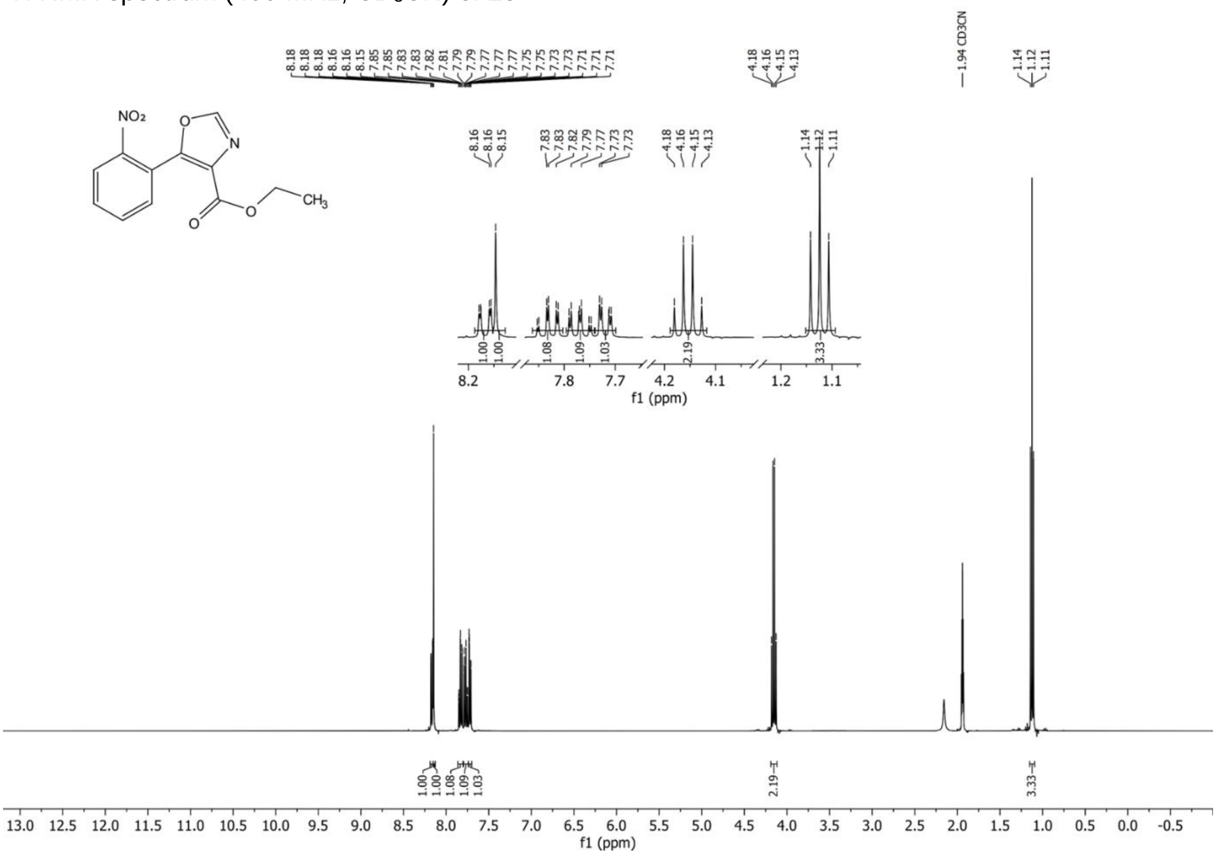

^{13}C NMR spectrum (101 MHz, CD_3CN) of **25**.

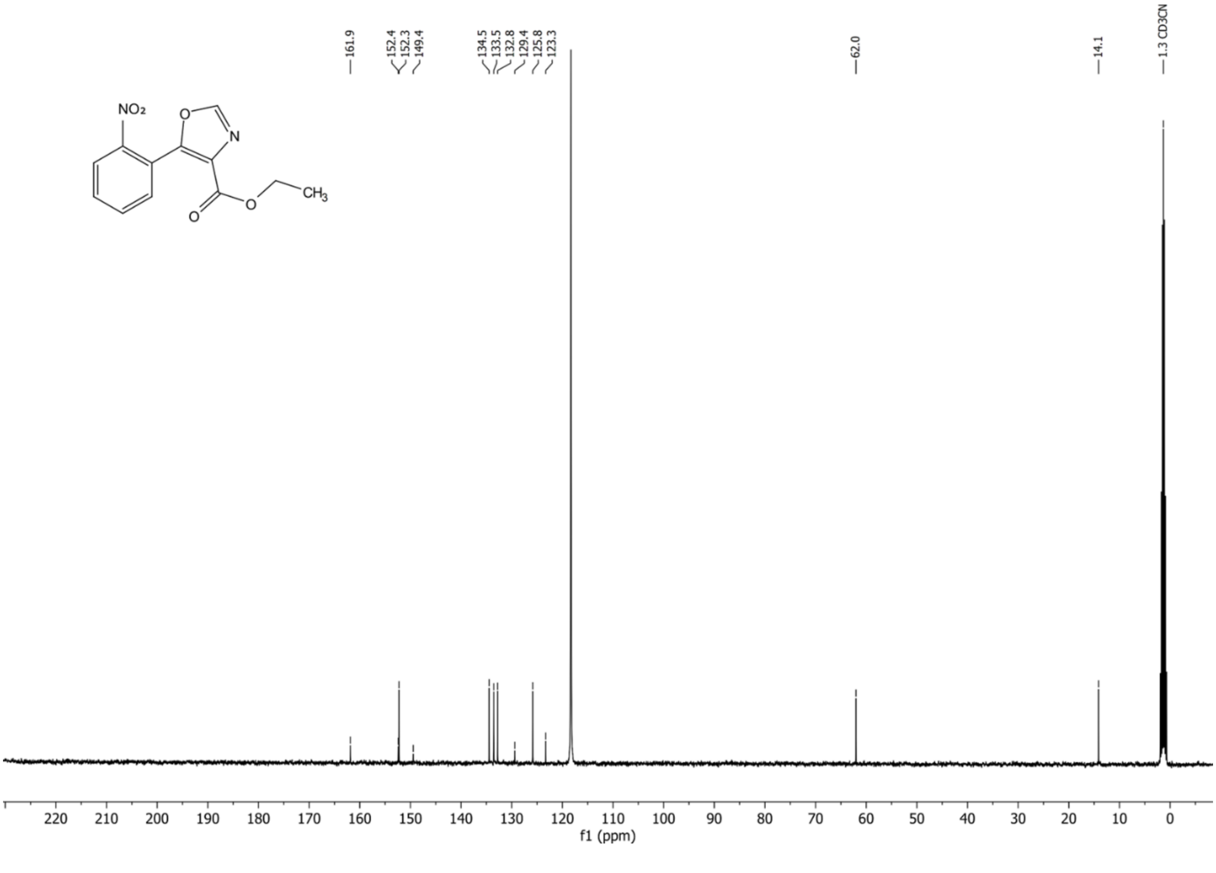

^{1}H NMR spectrum (400 MHz, DMSO-[D6]) of **4a**.

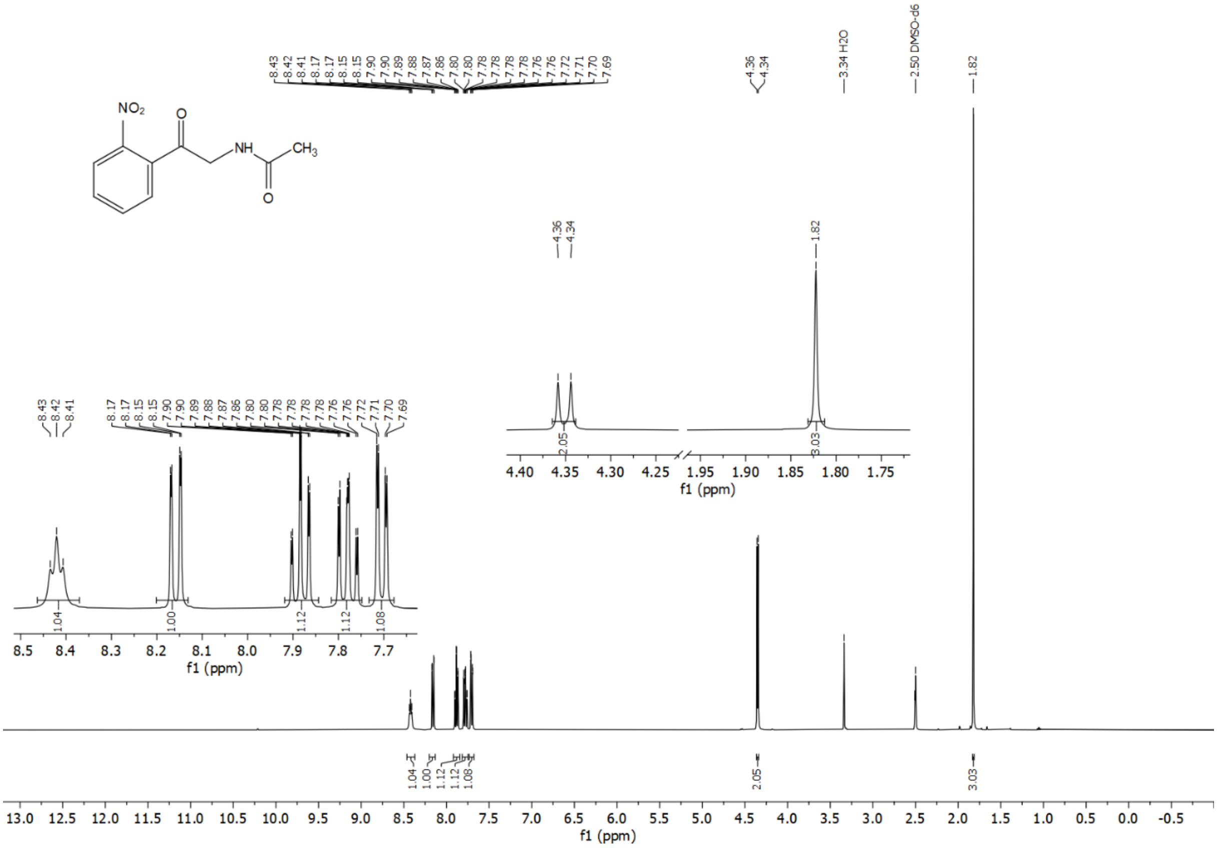

^{13}C NMR spectrum (101 MHz, DMSO-[D6]) of **4a**.

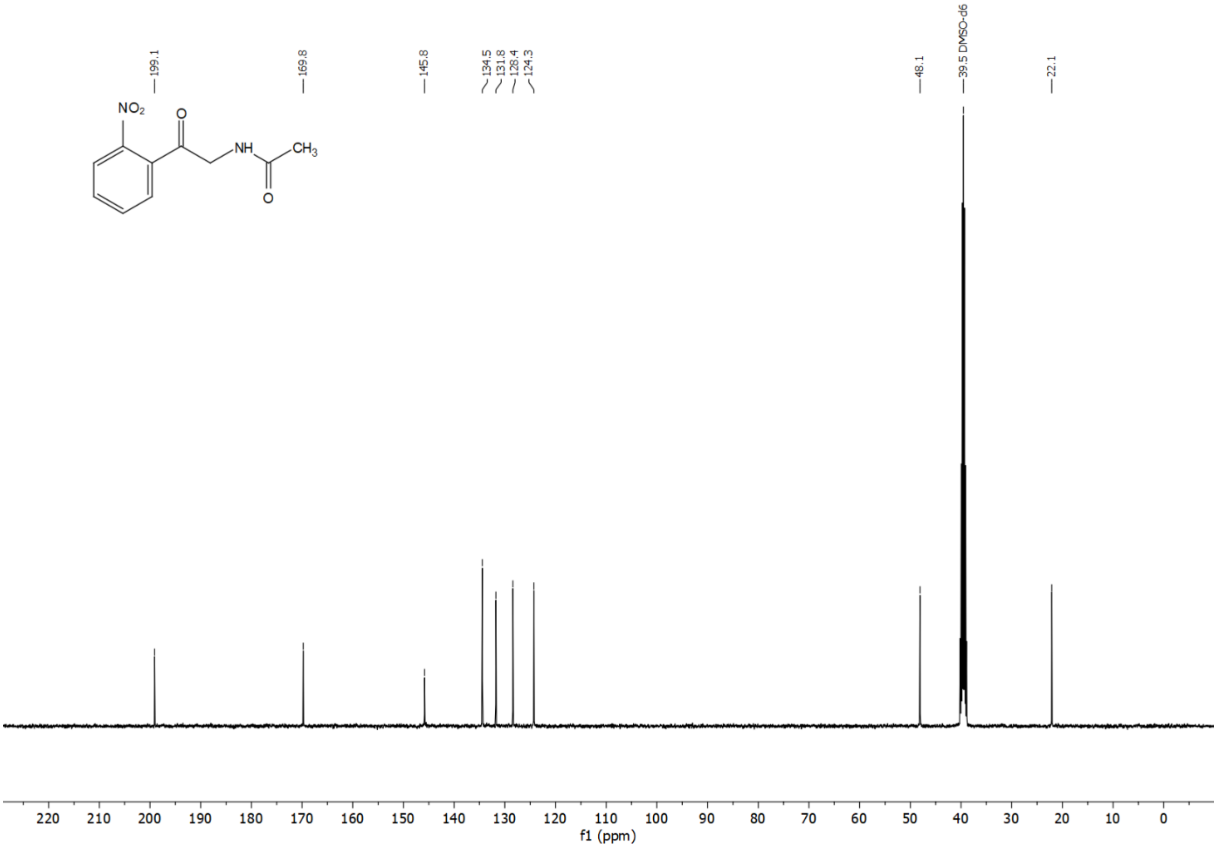

^{1}H NMR spectrum (400 MHz, CD_3CN) of **4b**.

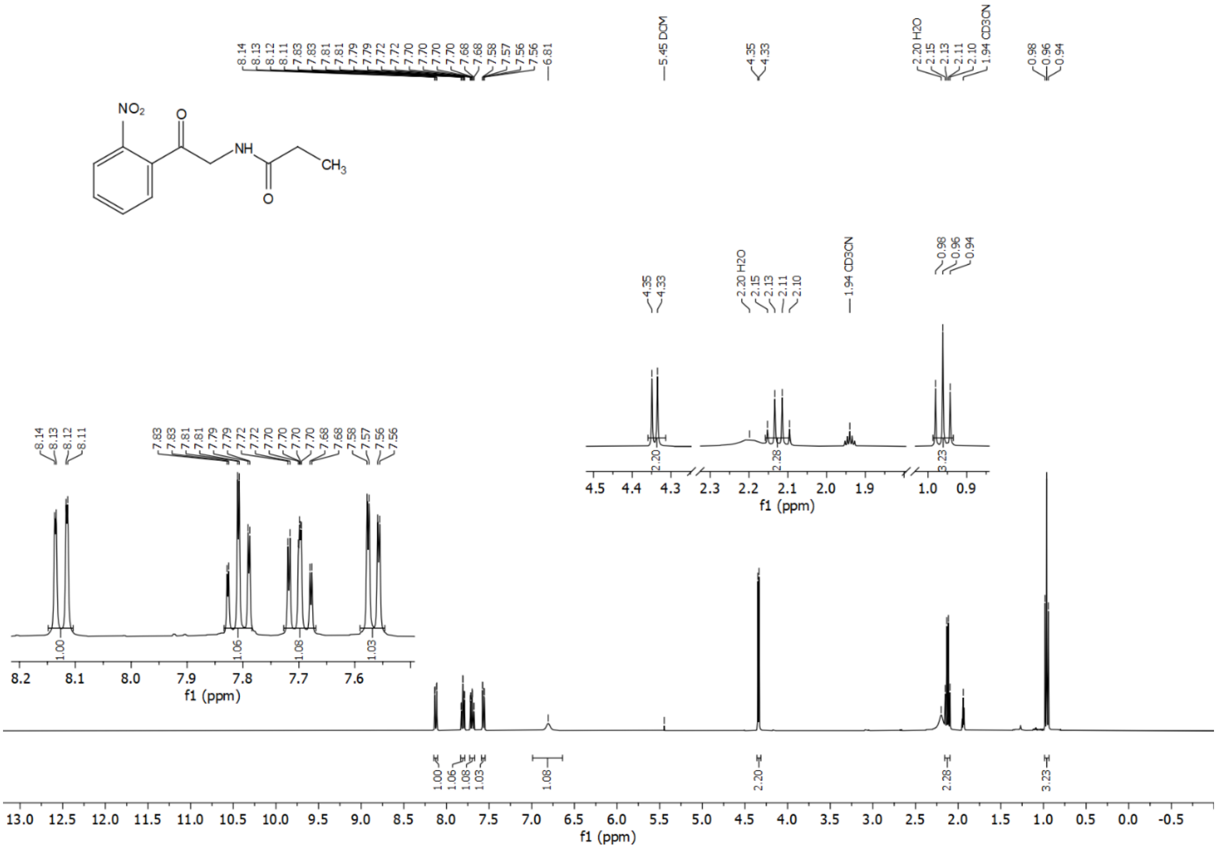

^{13}C NMR spectrum (101 MHz, CD_3CN) of **4b**.

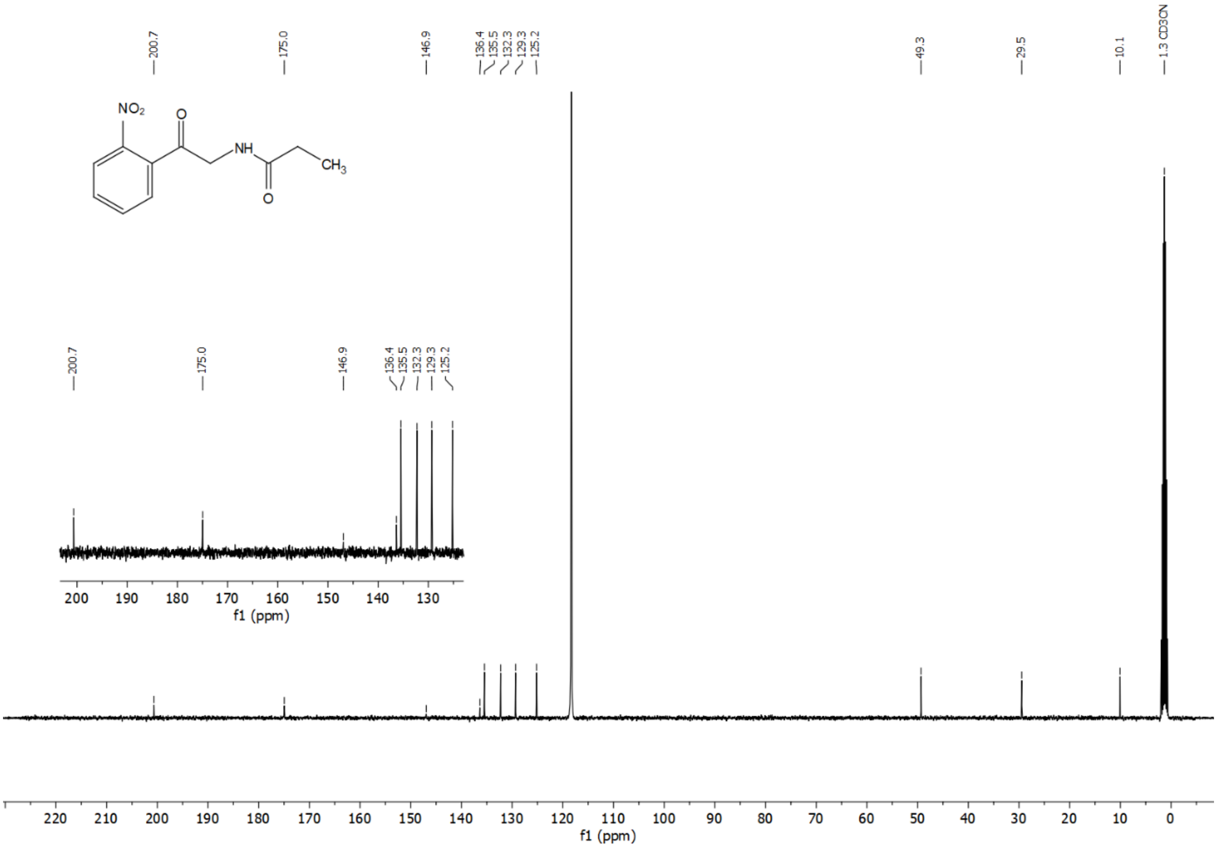

^{1}H NMR spectrum (400 MHz, CD_3CN) of **4c**.

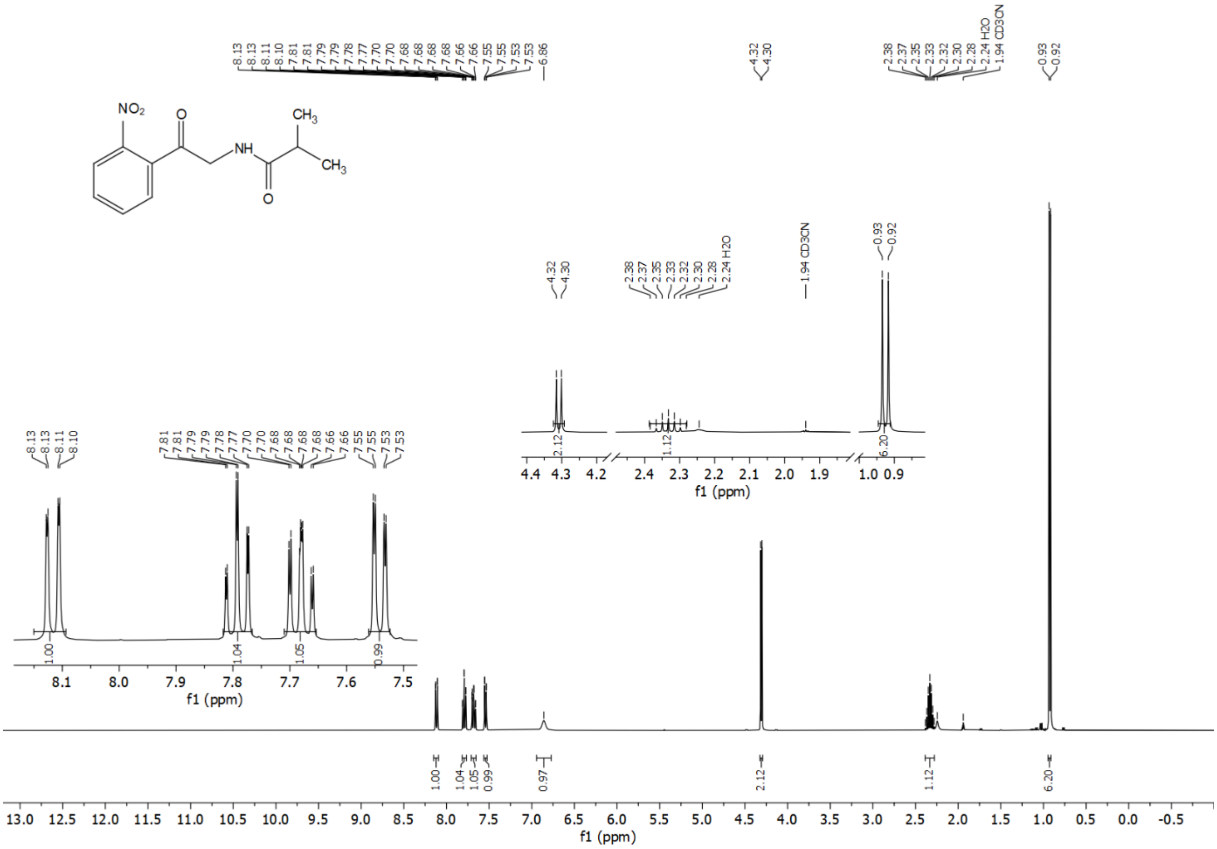

^{13}C NMR spectrum (101 MHz, CD_3CN) of **4c**.

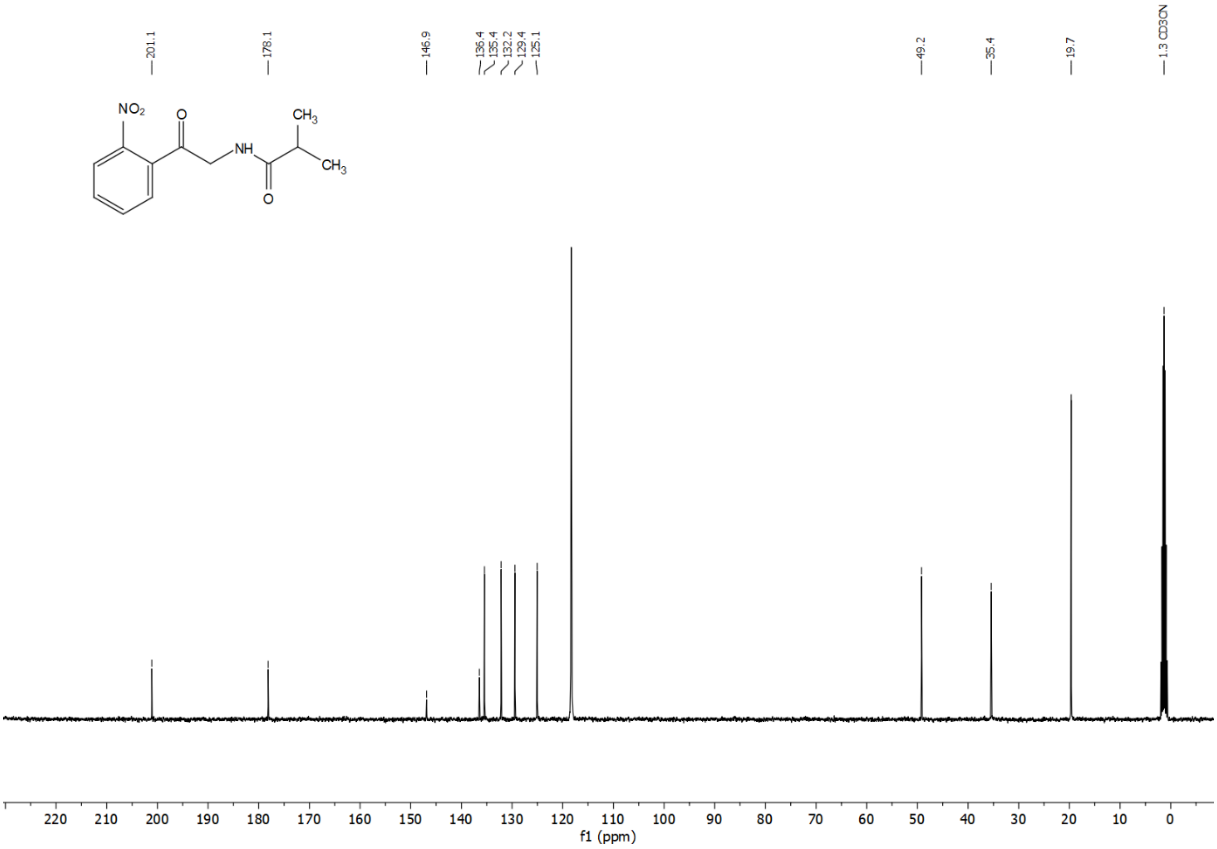

^{1}H NMR spectrum (400 MHz, CD_3CN) of **4d**.

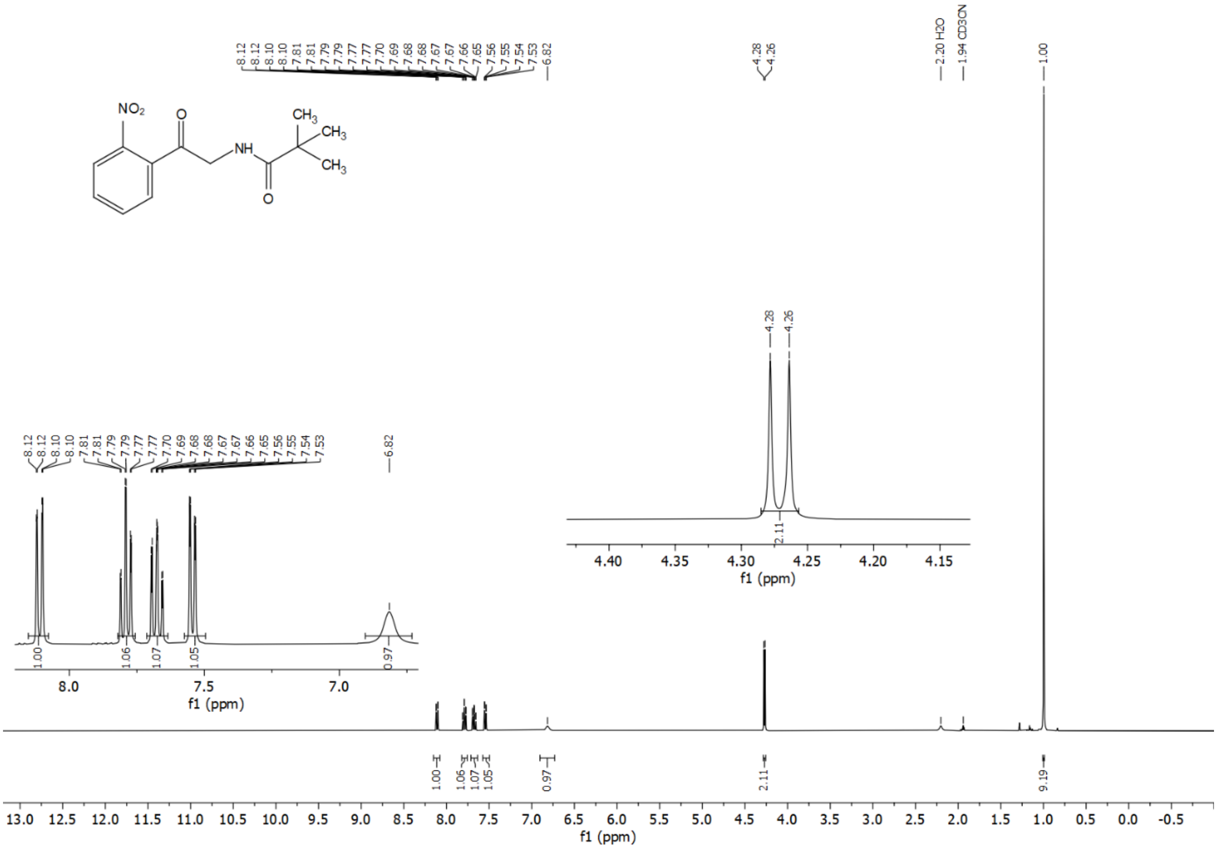

^{13}C NMR spectrum (101 MHz, CD_3CN) of **4d**.

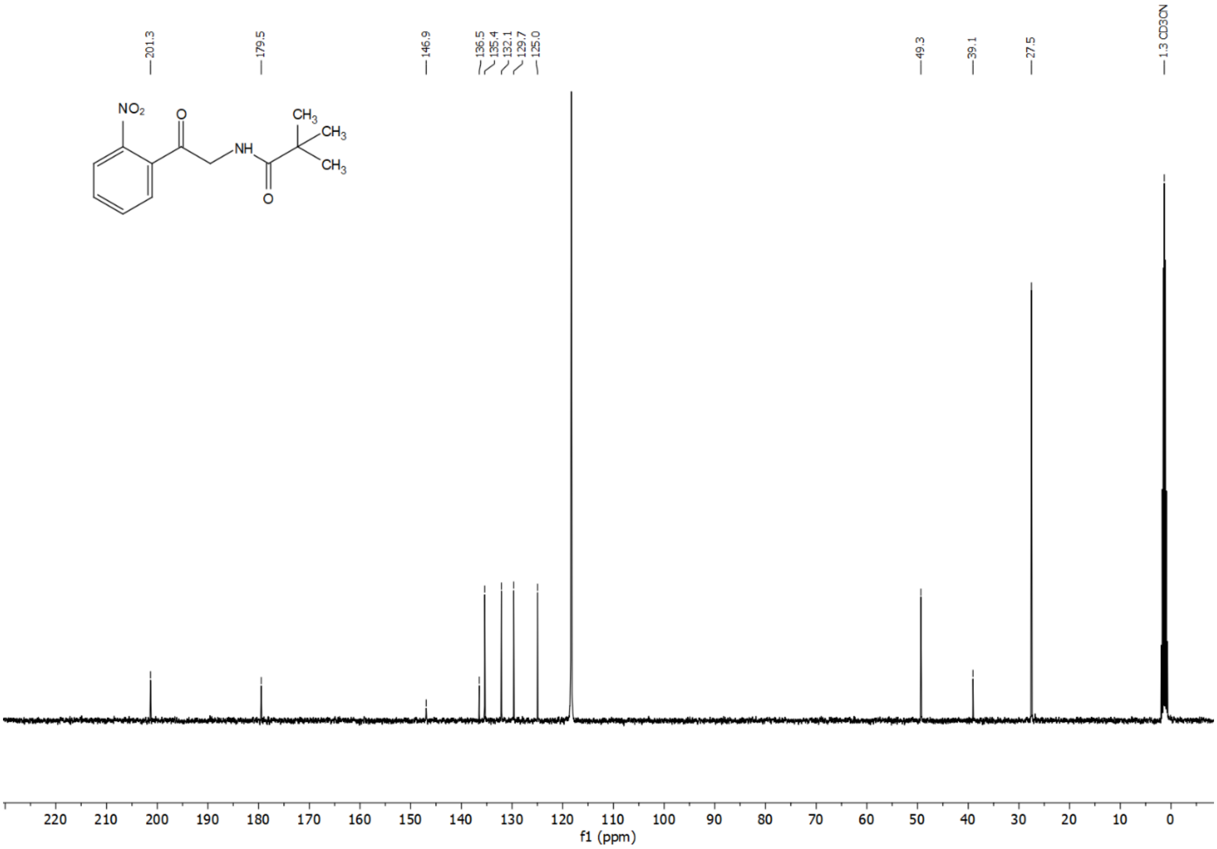

^{1}H NMR spectrum (400 MHz, $CDCl_3$) of **4e**.

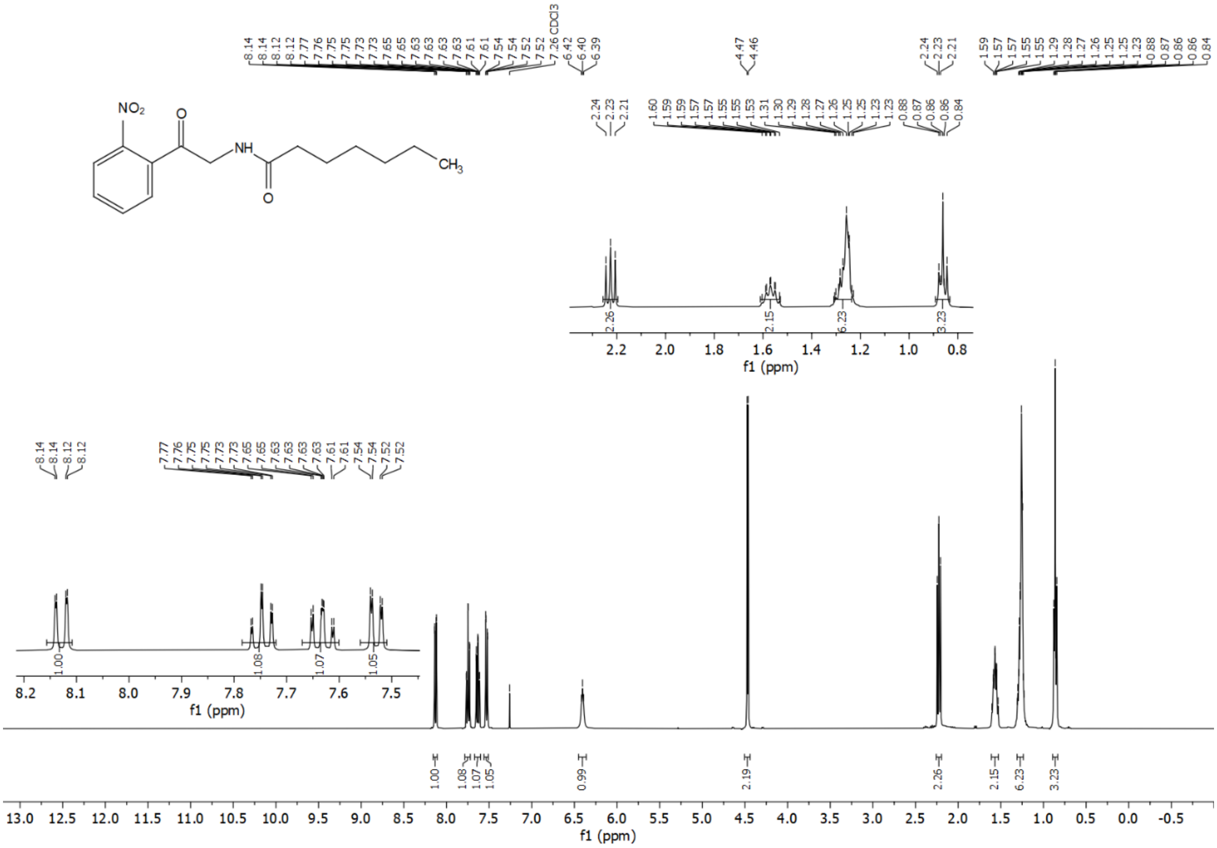

^{13}C NMR spectrum (101 MHz, $CDCl_3$) of **4e**.

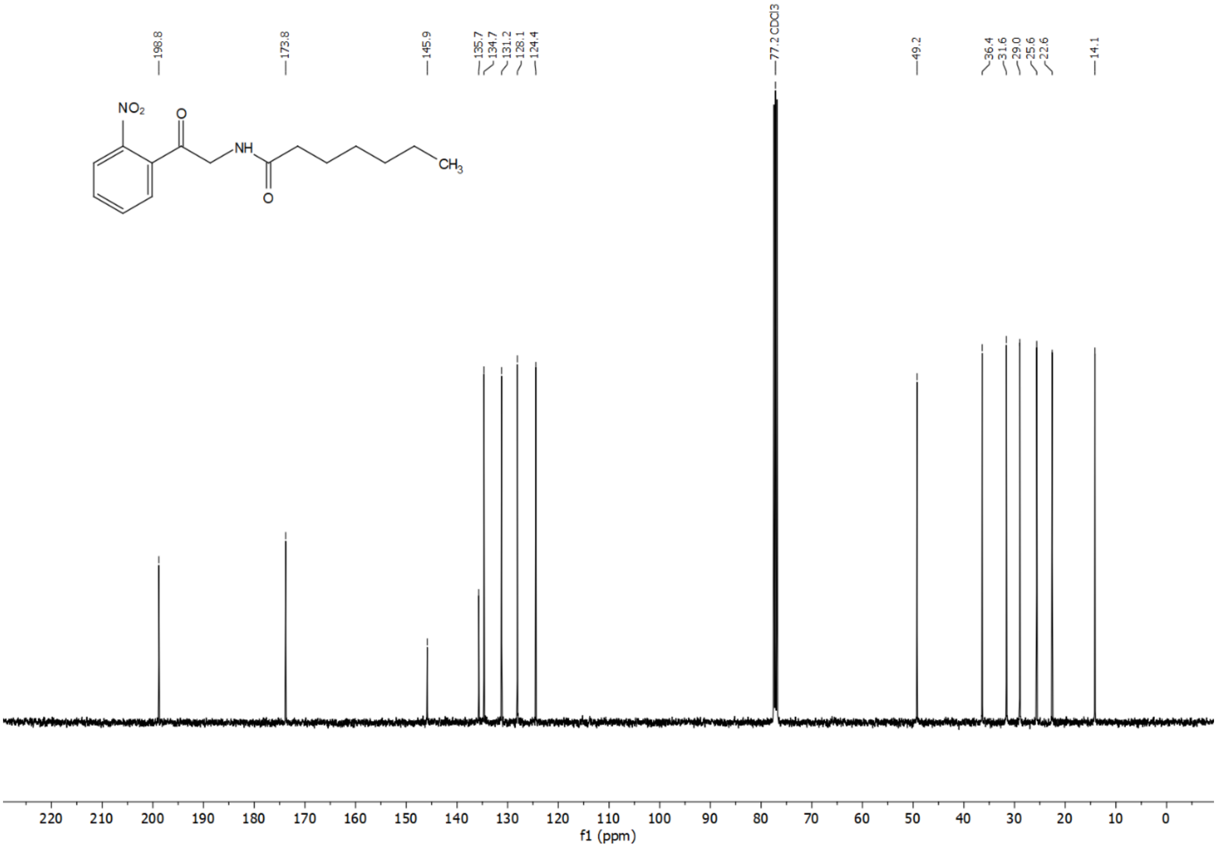

^{1}H NMR spectrum (400 MHz, CD_3CN) of **4f**.

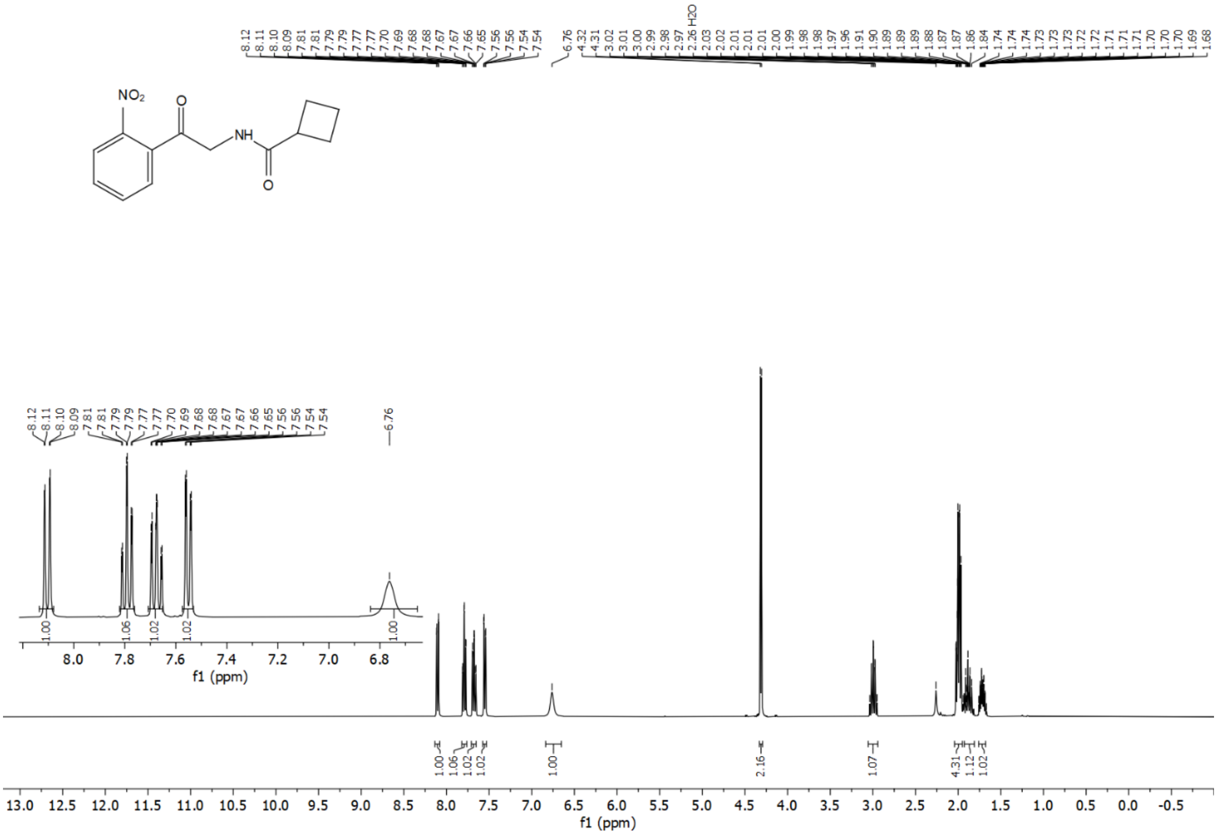

^{13}C NMR spectrum (101 MHz, CD_3CN) of **4f**.

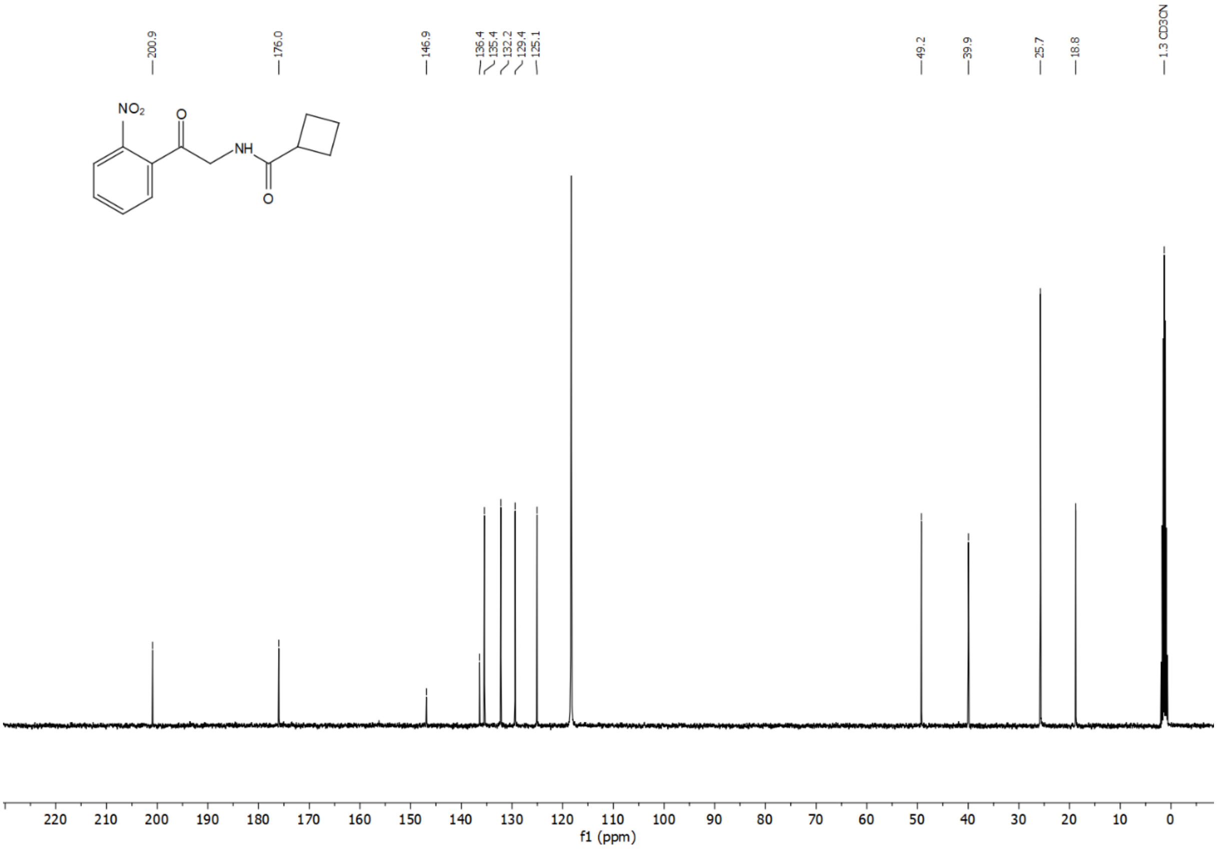

^{1}H NMR spectrum (400 MHz, CD_3CN) of **4g**.

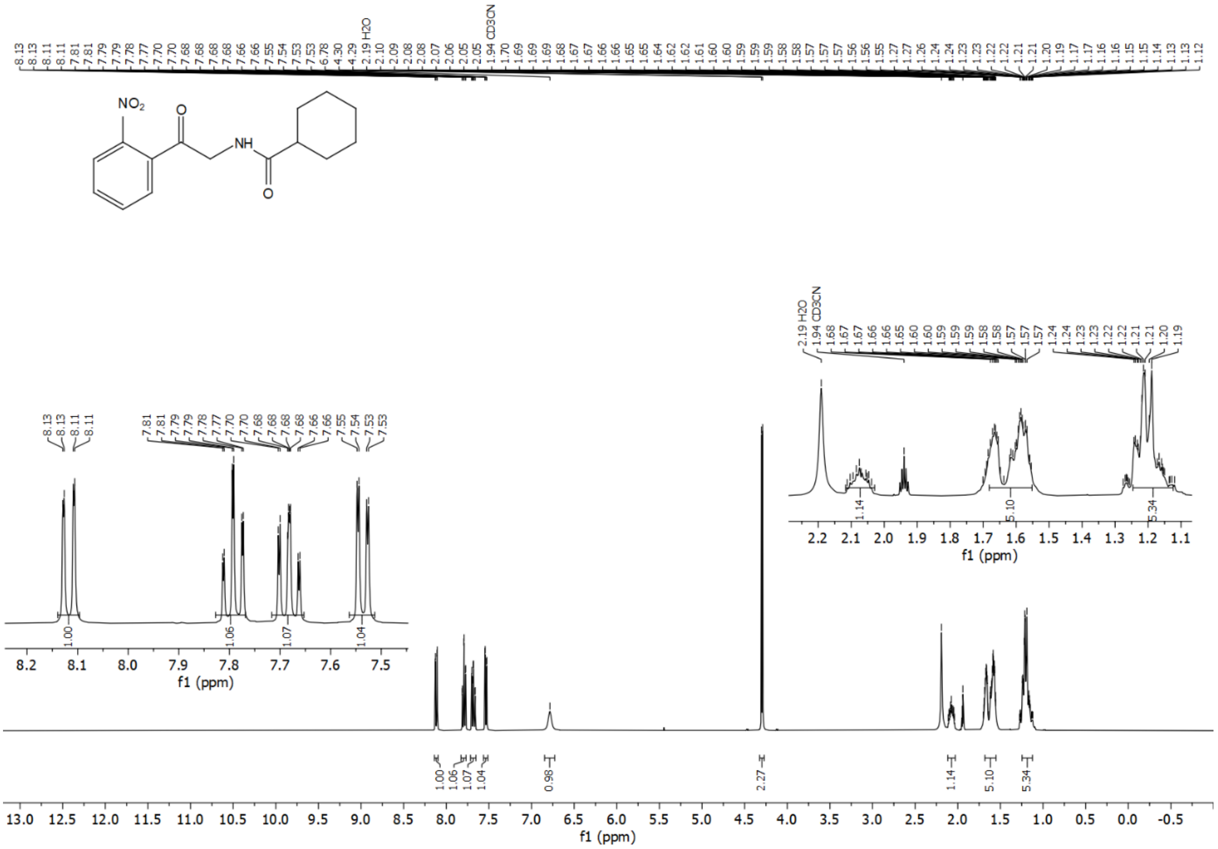

^{13}C NMR spectrum (101 MHz, CD_3CN) of **4g**.

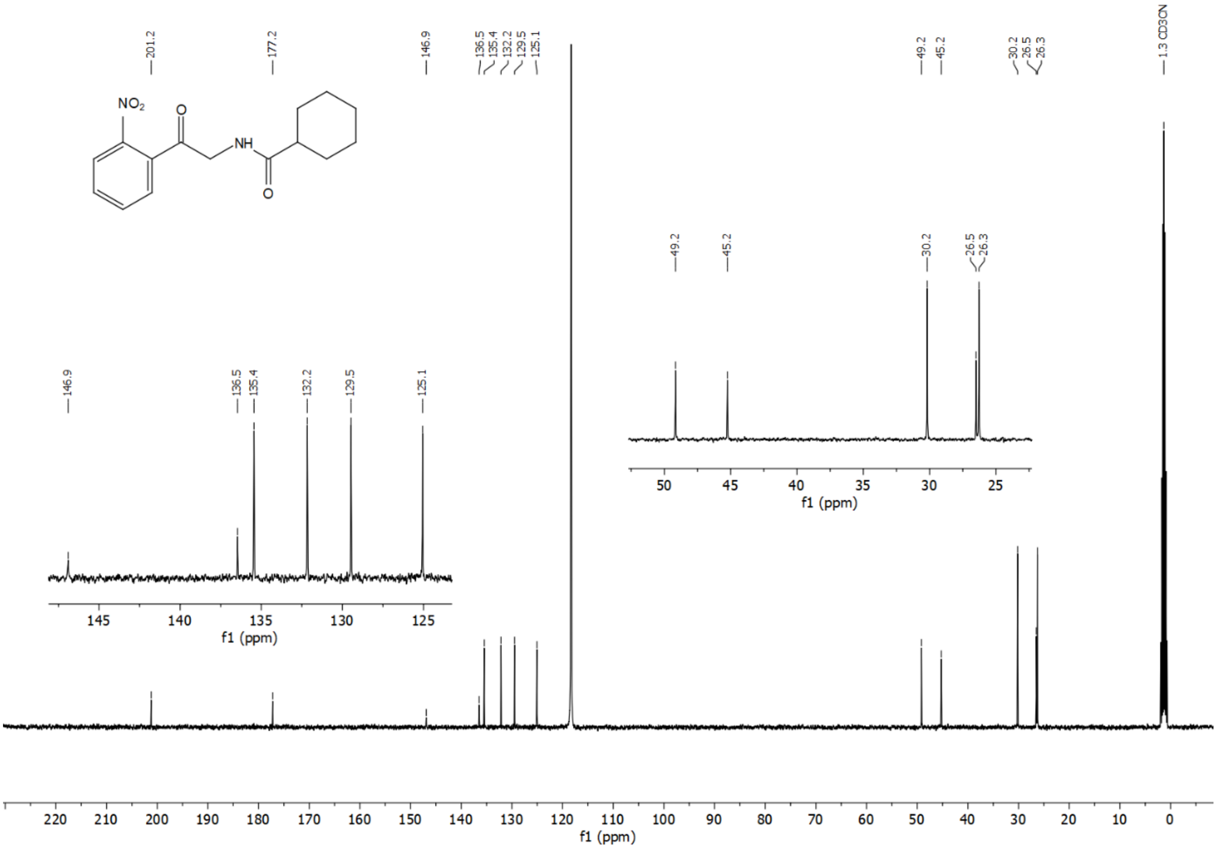

^{1}H NMR spectrum (400 MHz, CD_3CN) of **4h**.

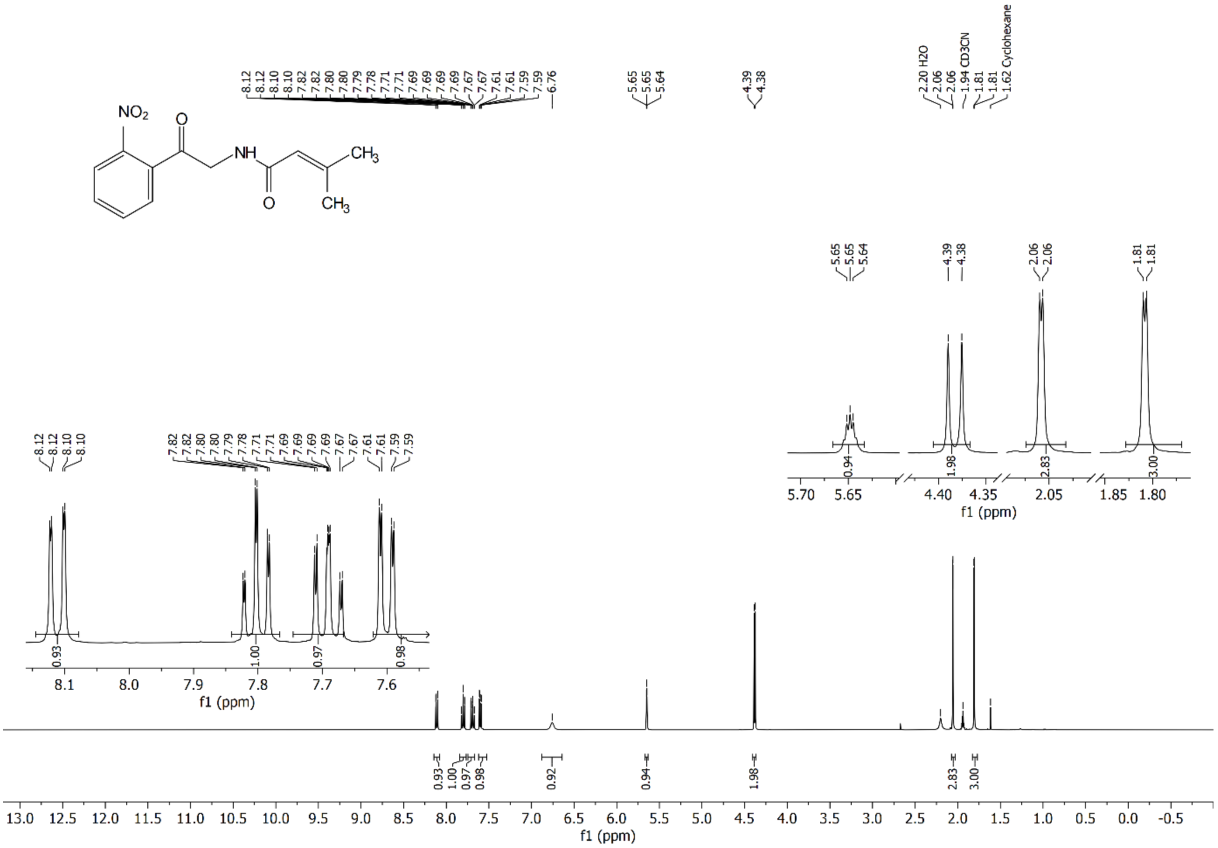

^{13}C NMR spectrum (101 MHz, CD_3CN) of **4h**.

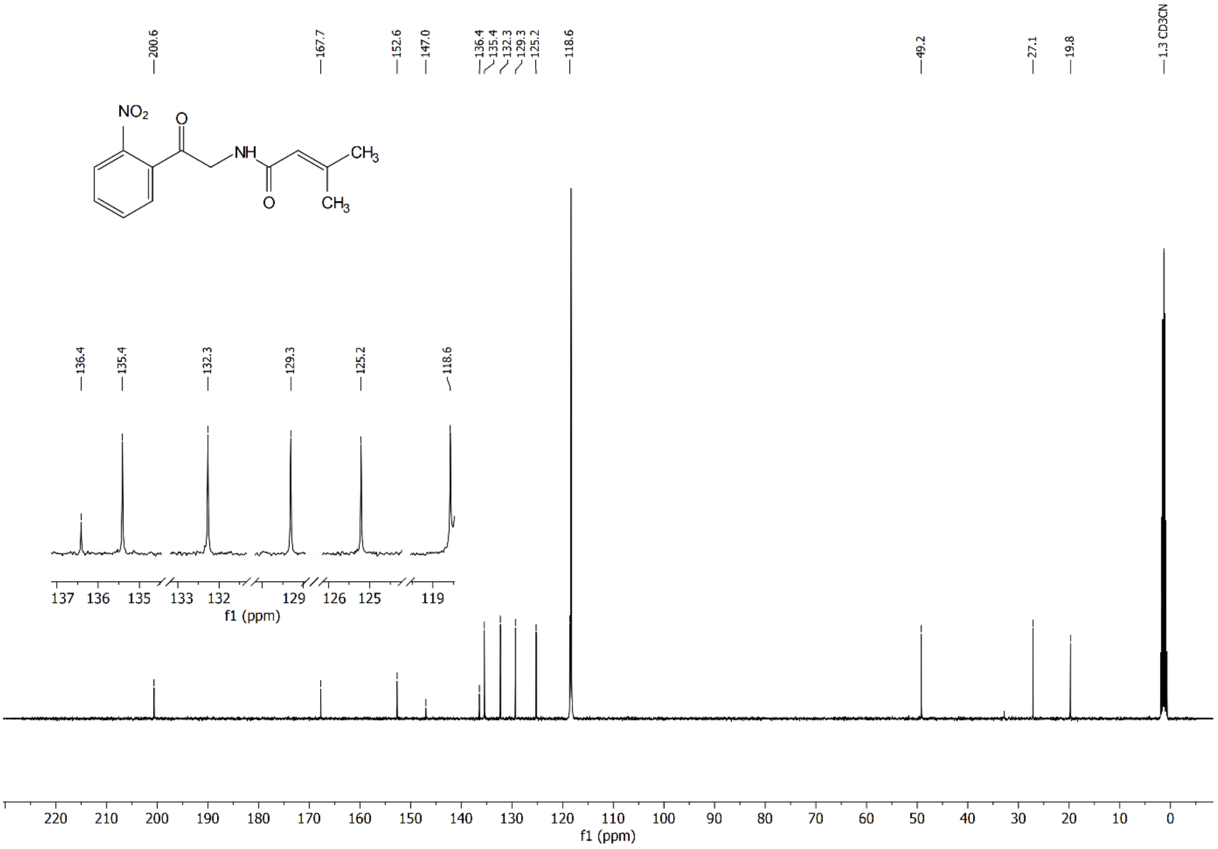

^{1}H NMR spectrum (400 MHz, CD_3CN) of **4i**.

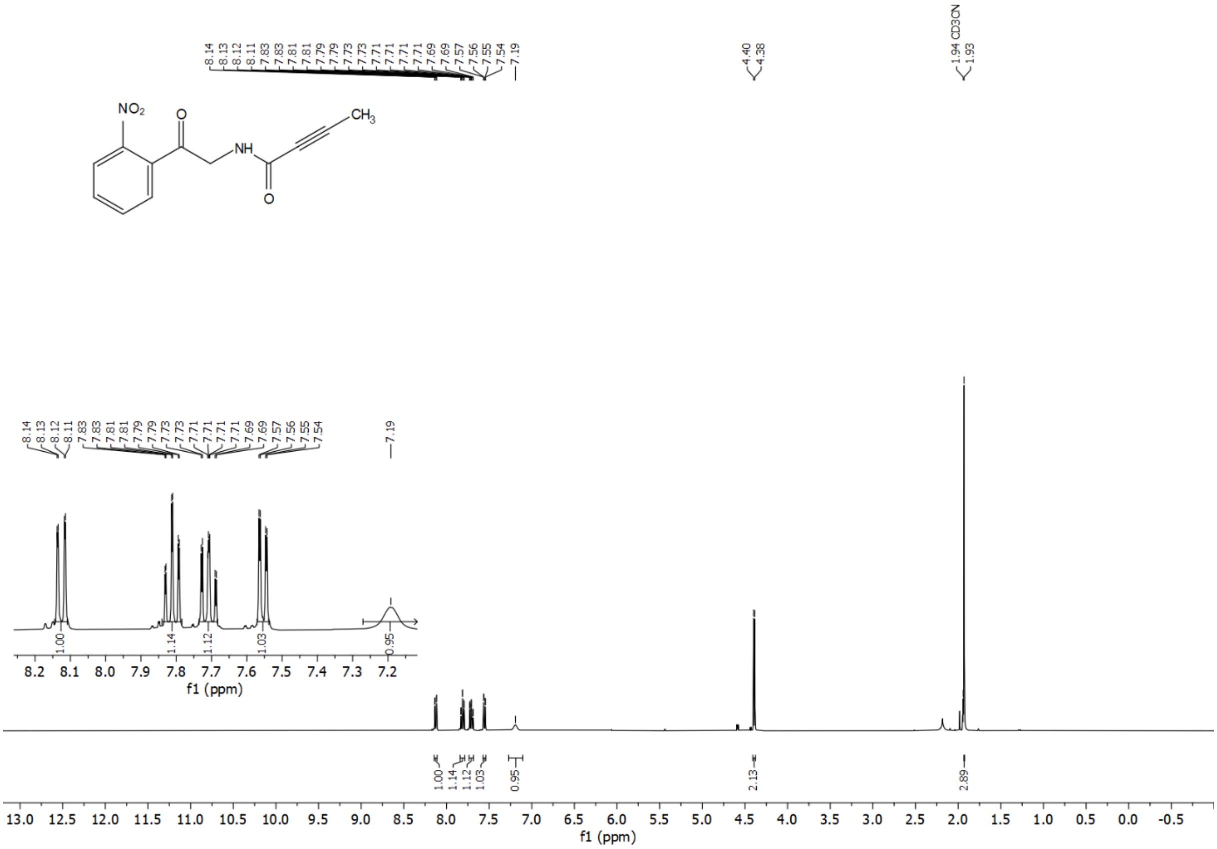

^{13}C NMR spectrum (101 MHz, CD_3CN) of **4i**.

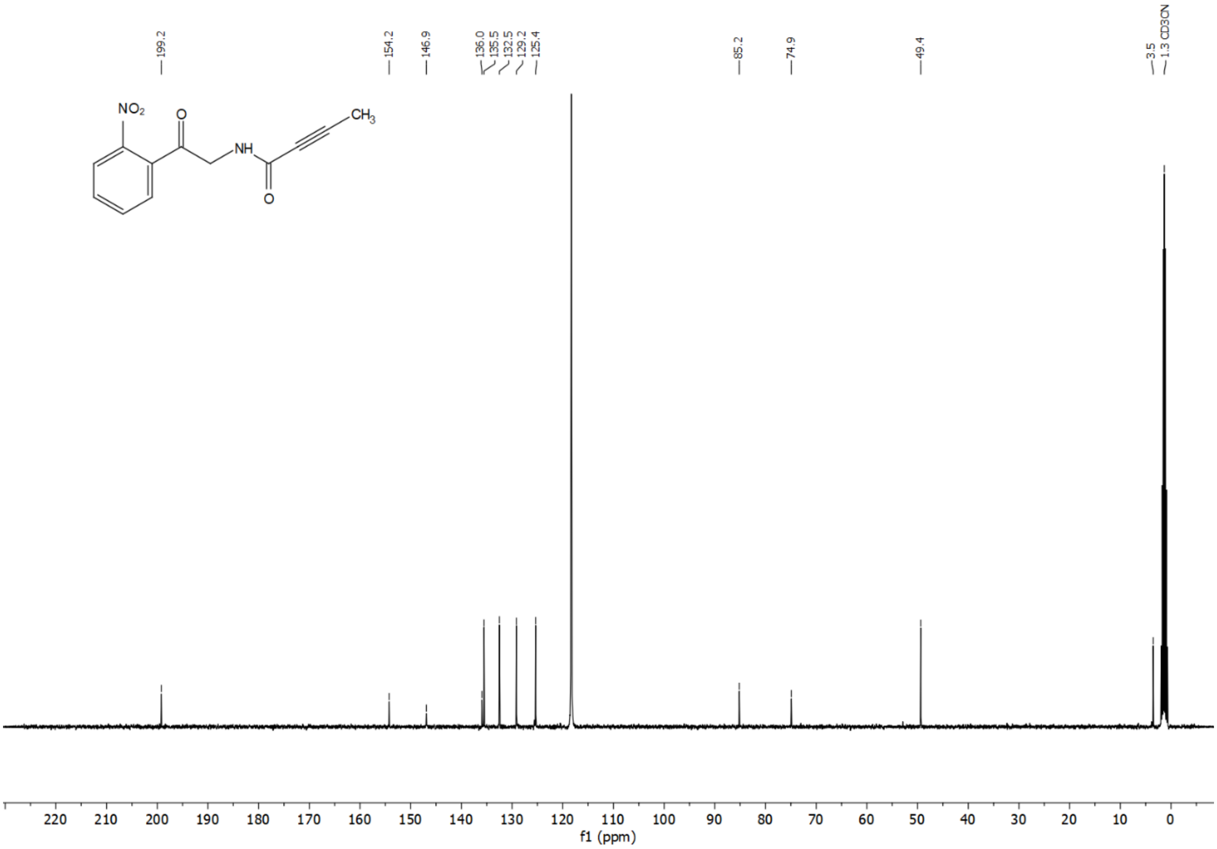

^{1}H NMR spectrum (400 MHz, CD_3CN) of **4j**.

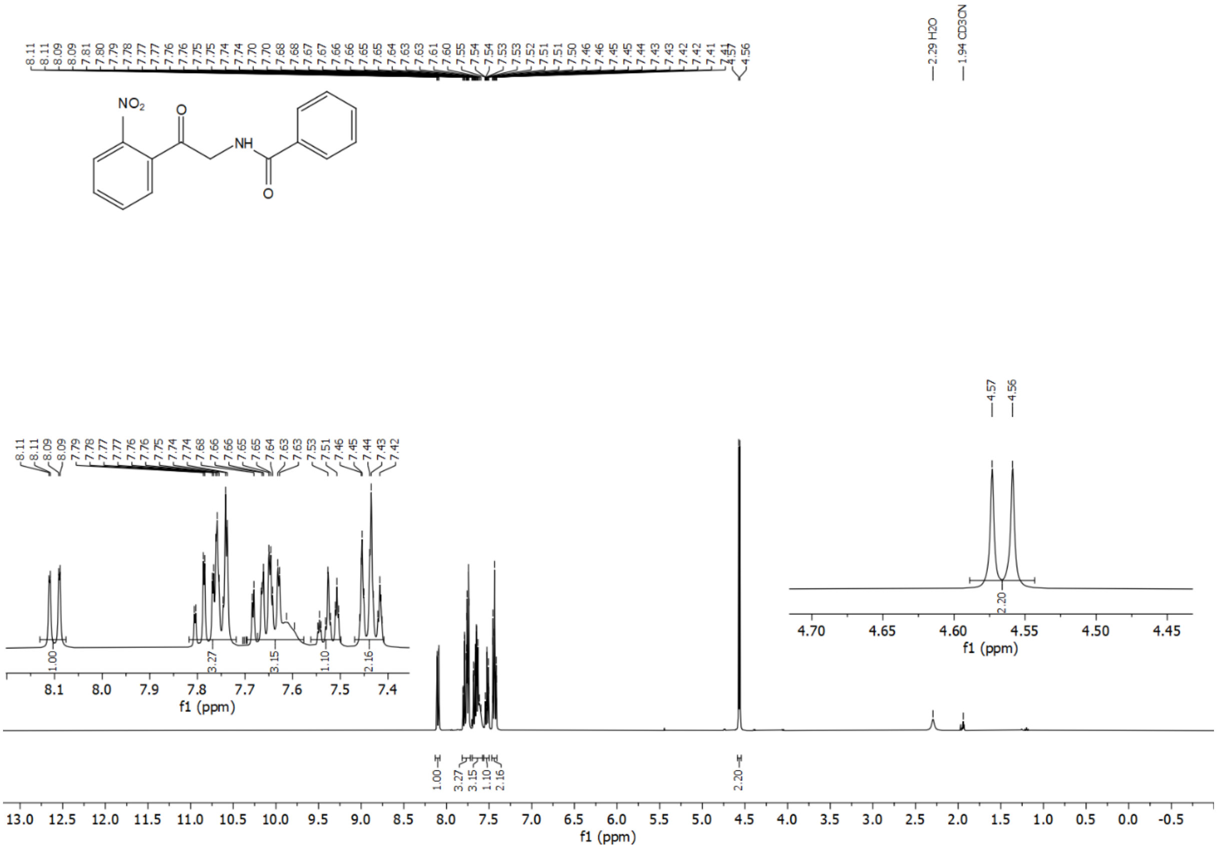

^{13}C NMR spectrum (101 MHz, CD_3CN) of **4j**.

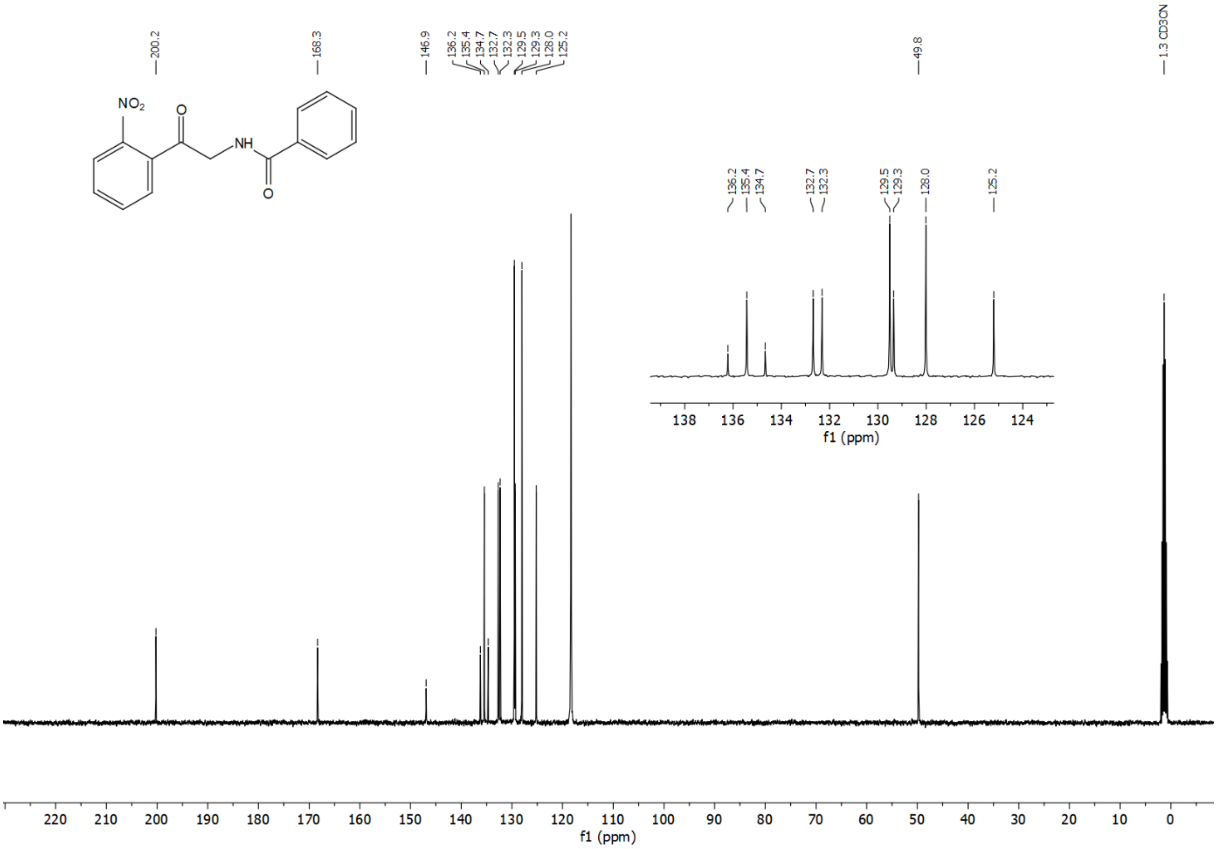

^1H NMR spectrum (400 MHz, CD_3CN) of **4k**.

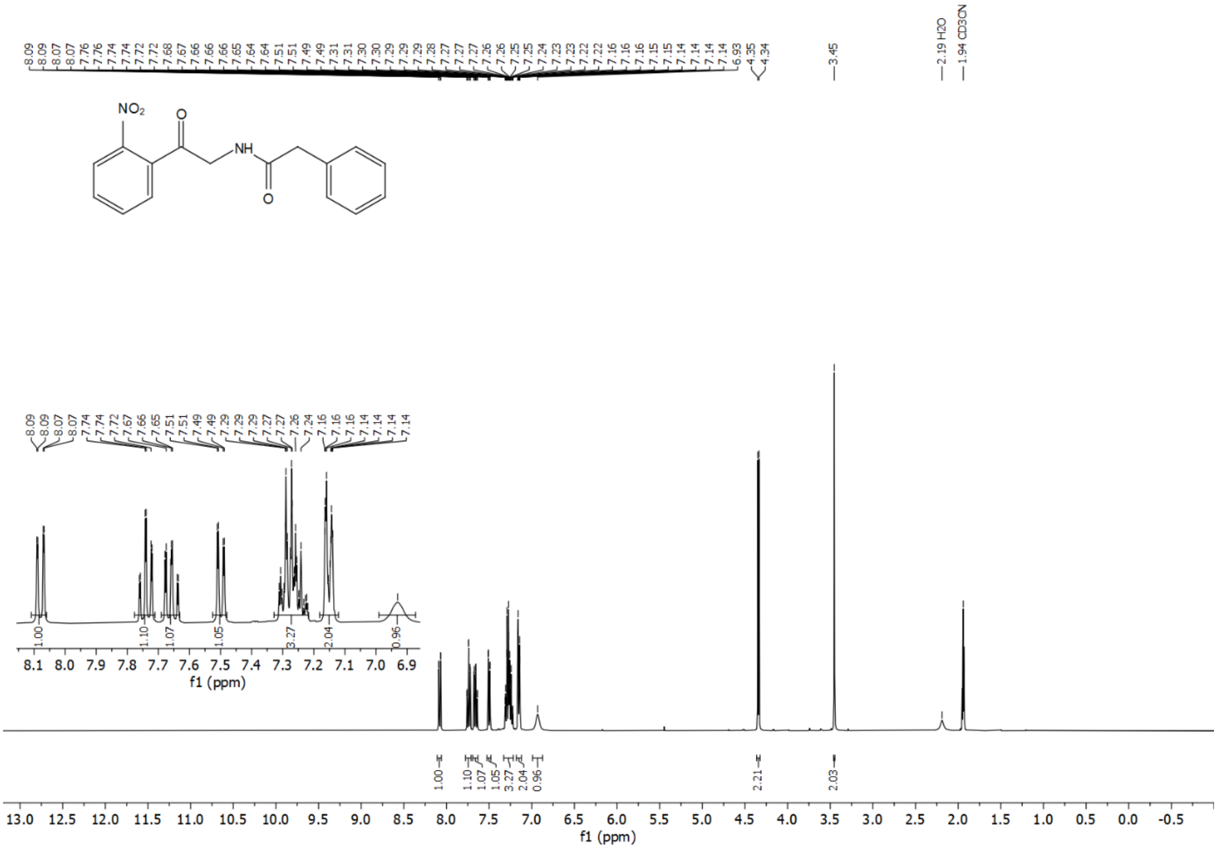

^{13}C NMR spectrum (101 MHz, CD_3CN) of **4k**.

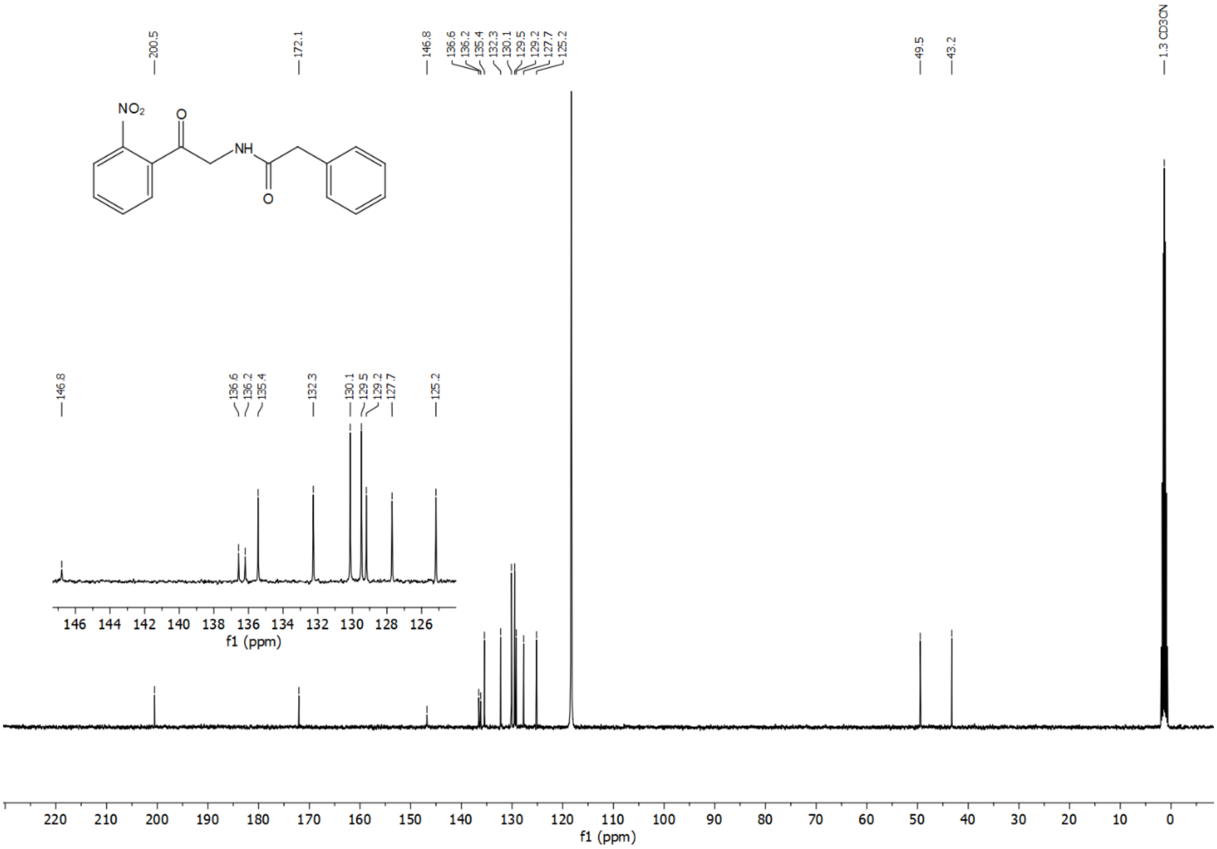

^{1}H NMR spectrum (400 MHz, CD_3CN) of **4l**.

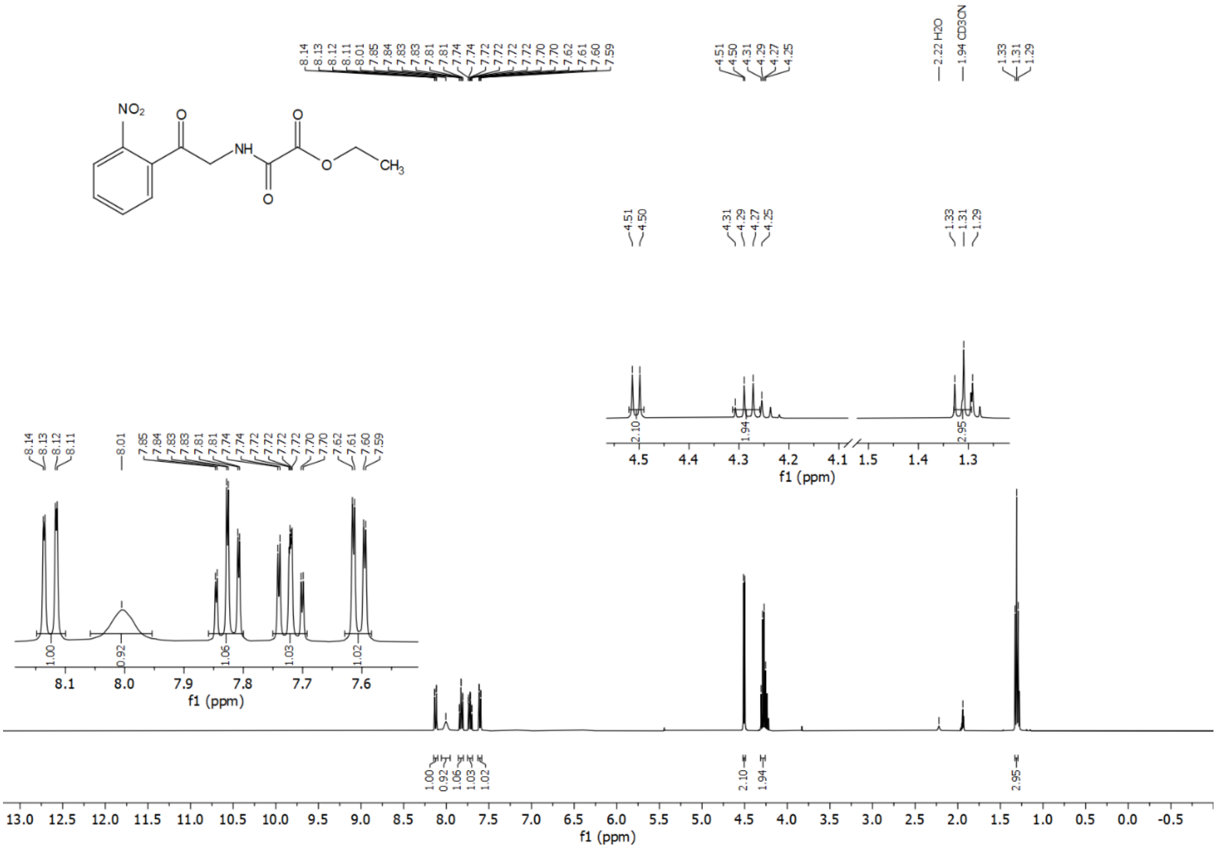

^{13}C NMR spectrum (101 MHz, CD_3CN) of **4l**.

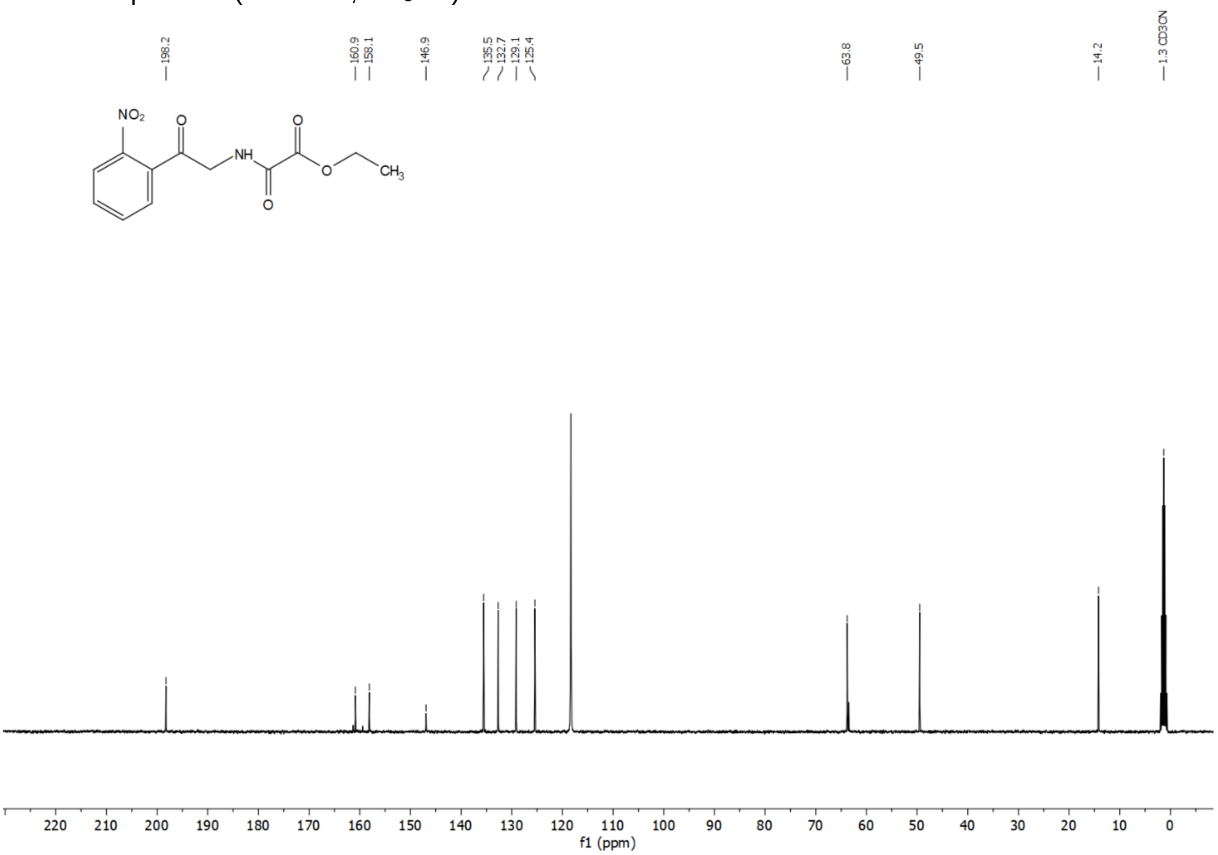

^{1}H NMR spectrum (400 MHz, CD_3CN) of **4m**.

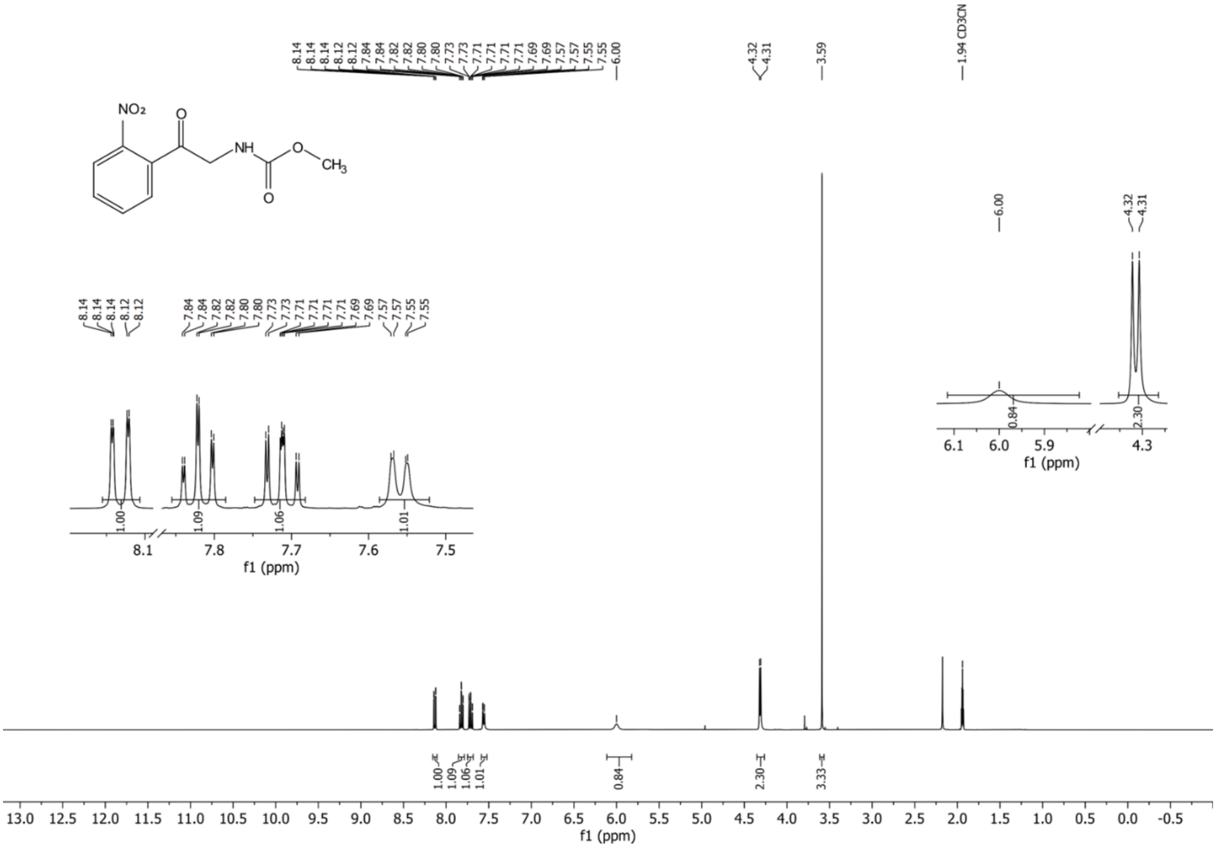

^{13}C NMR spectrum (101 MHz, CD_3CN) of **4m**.

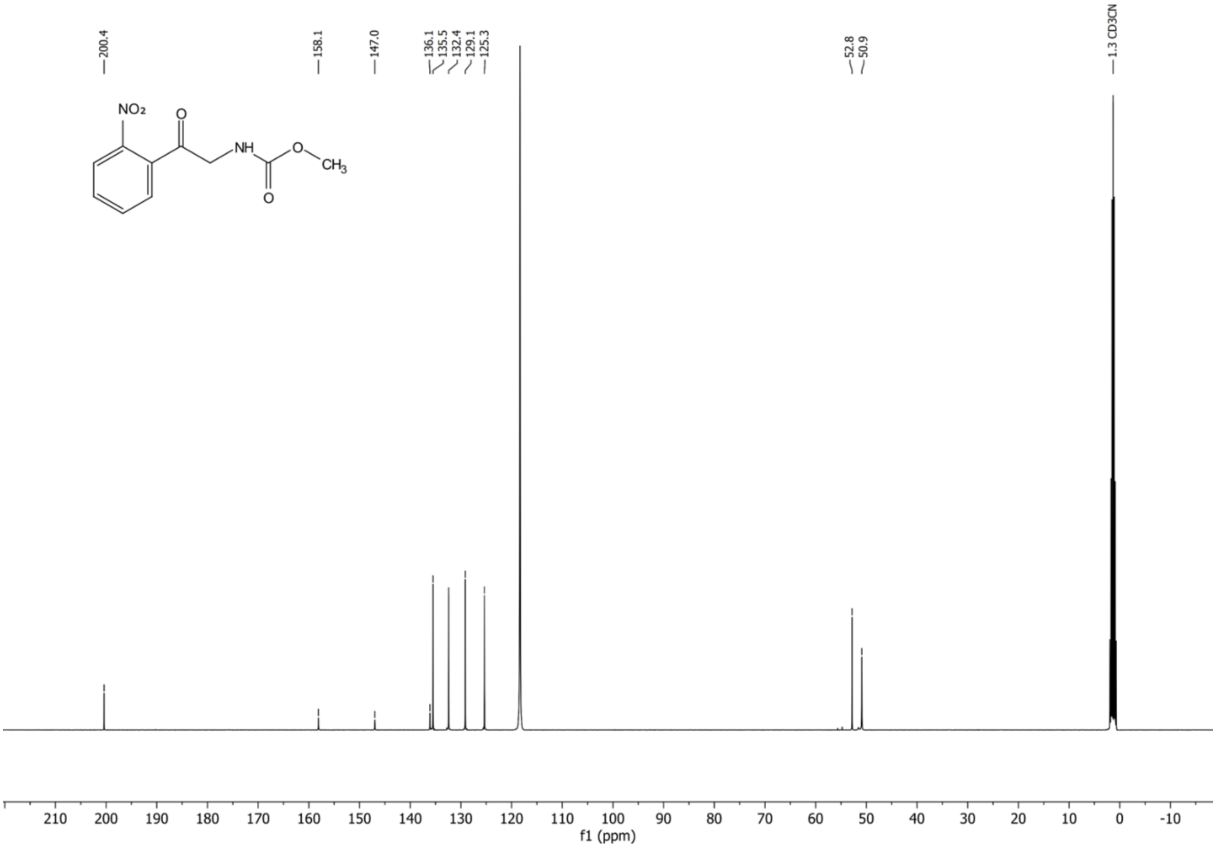

^{1}H NMR spectrum (400 MHz, CD_3CN) of **4n**.

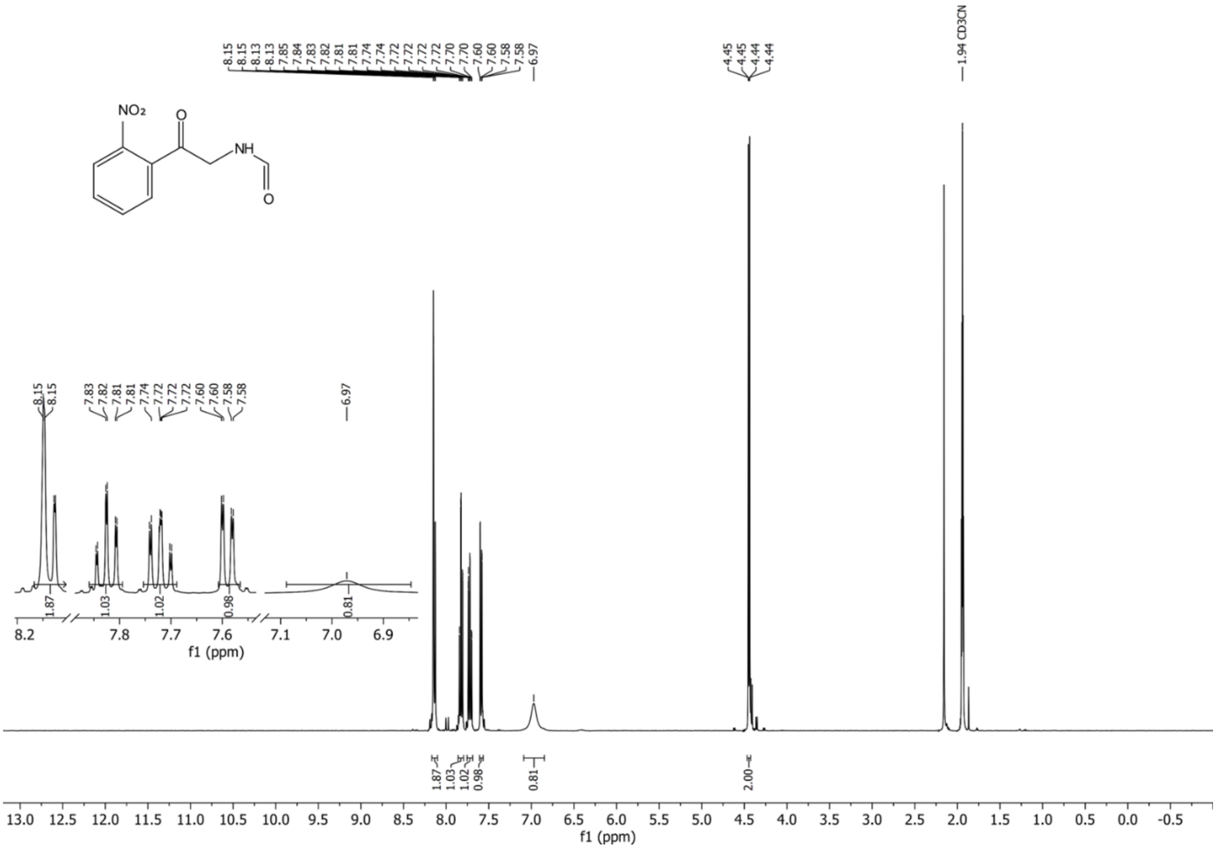

^{13}C NMR spectrum (101 MHz, CD_3CN) of **4n**.

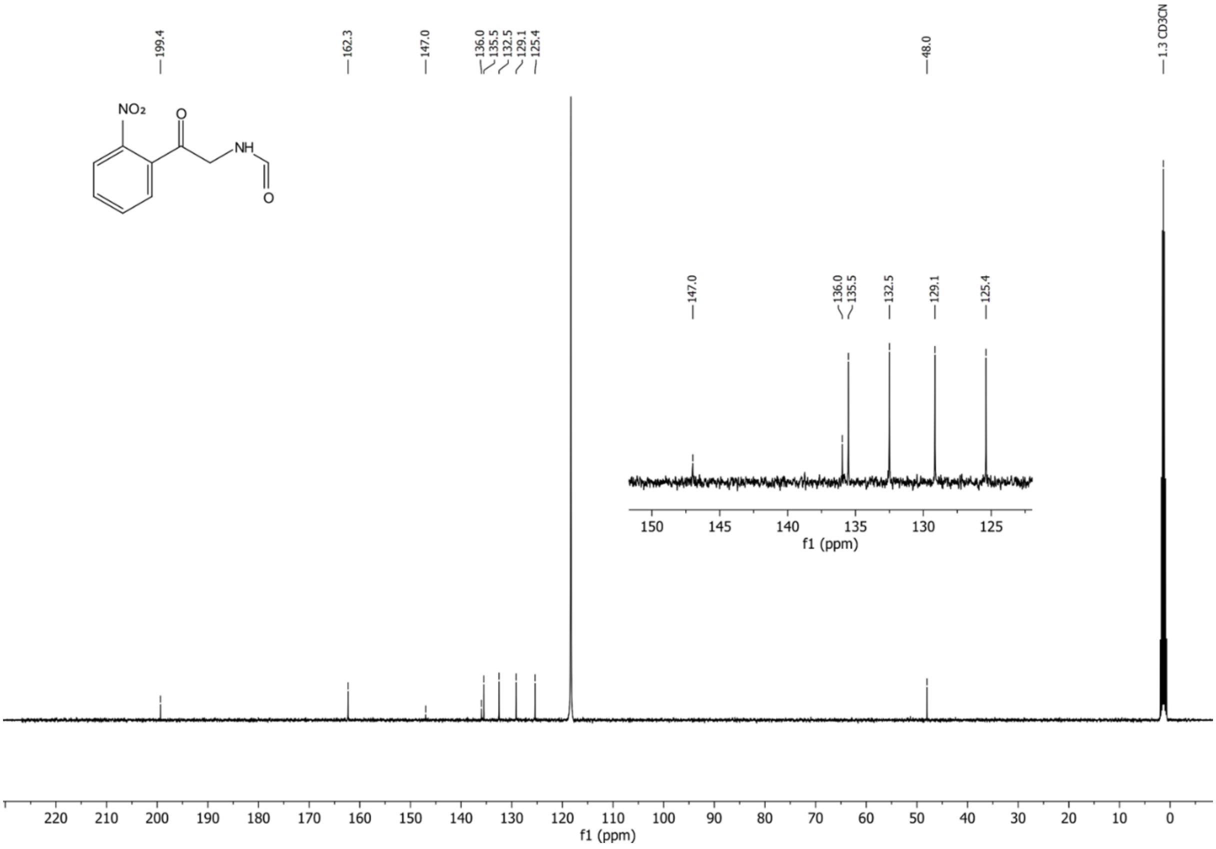

^{1}H NMR spectrum (400 MHz, CD_2Cl_2) of **4o**.

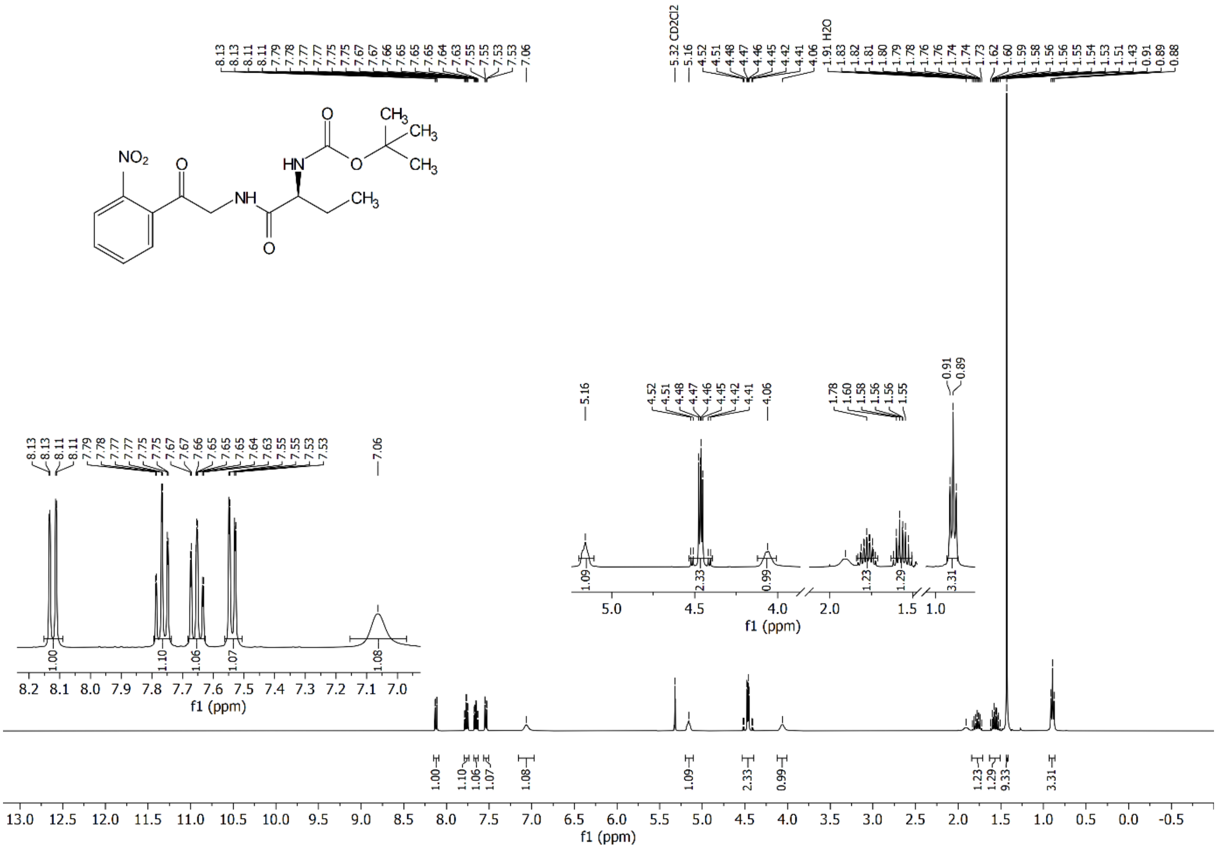

^{13}C NMR spectrum (101 MHz, CD_2Cl_2) of **4o**.

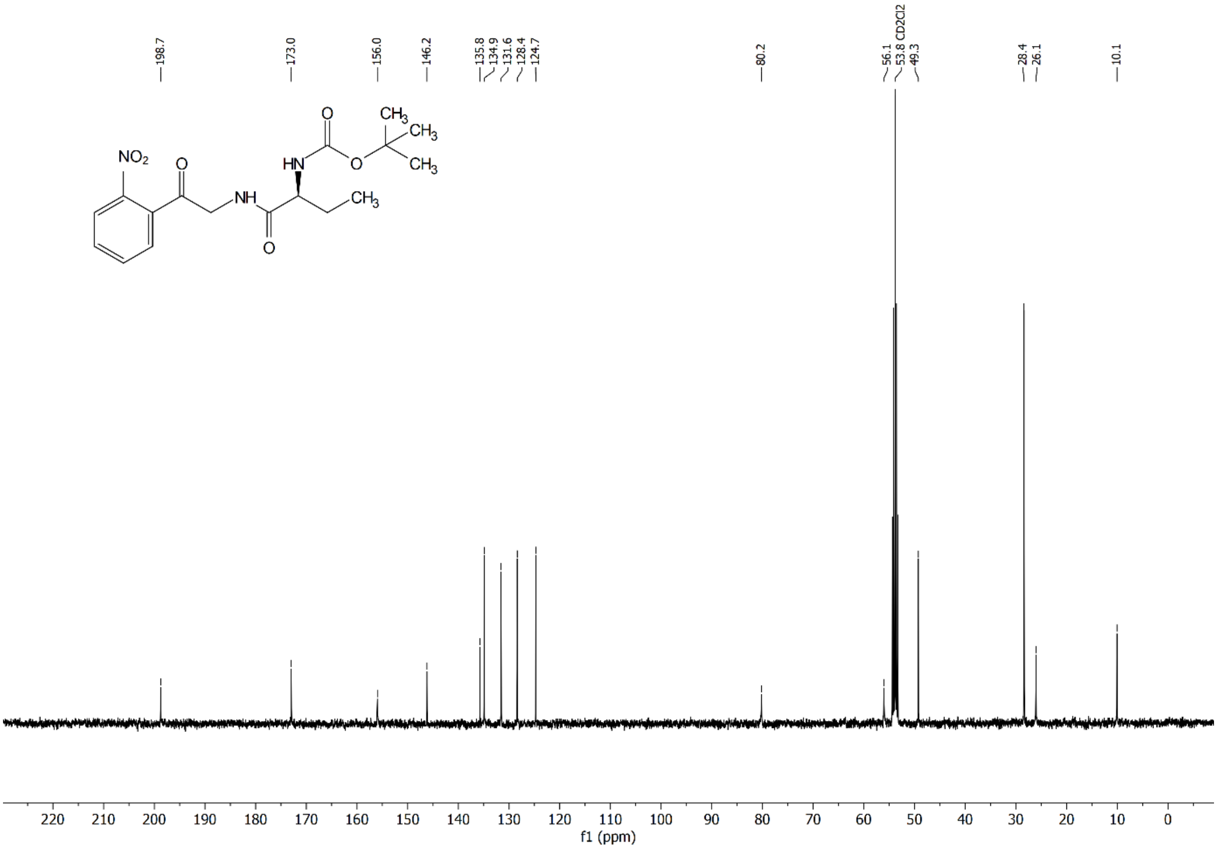

^{1}H NMR spectrum (400 MHz, CD_3CN) of **4p**.

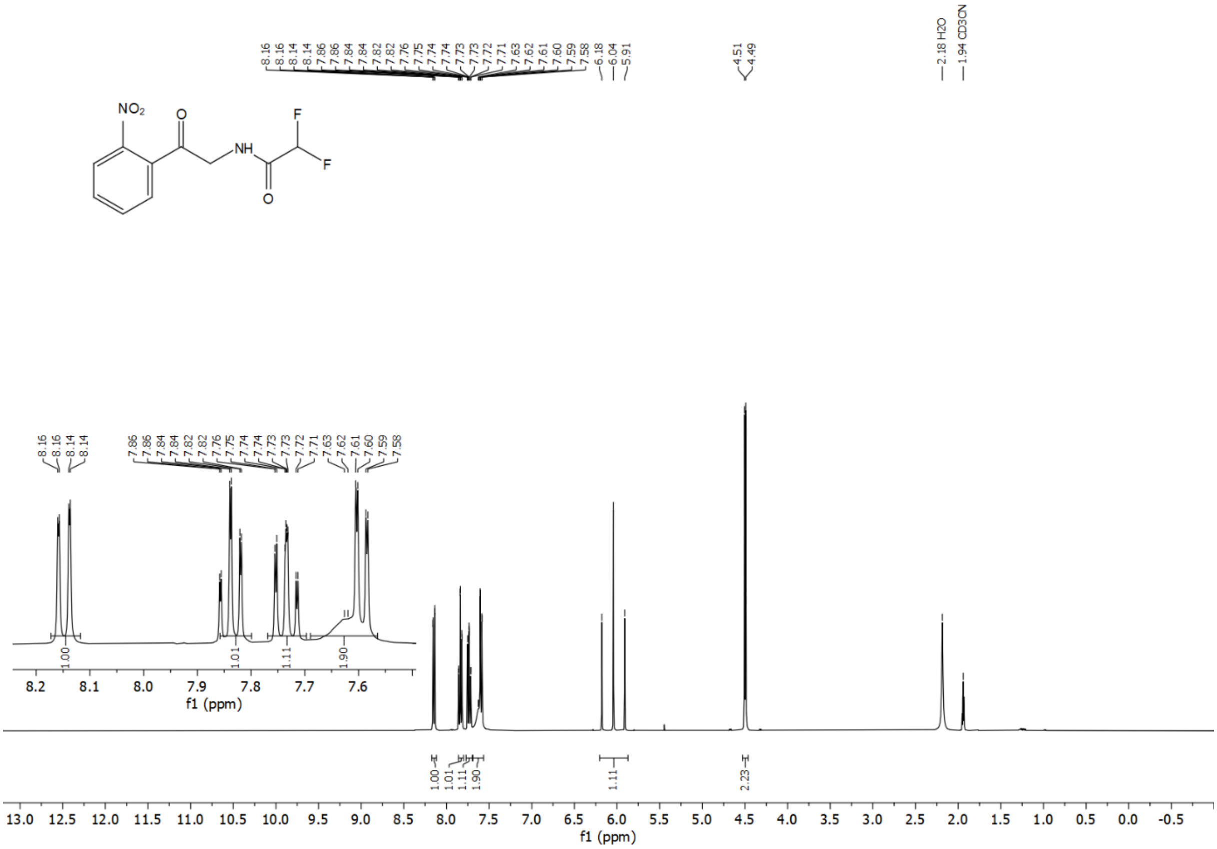

^{13}C NMR spectrum (101 MHz, CD_3CN) of **4p**.

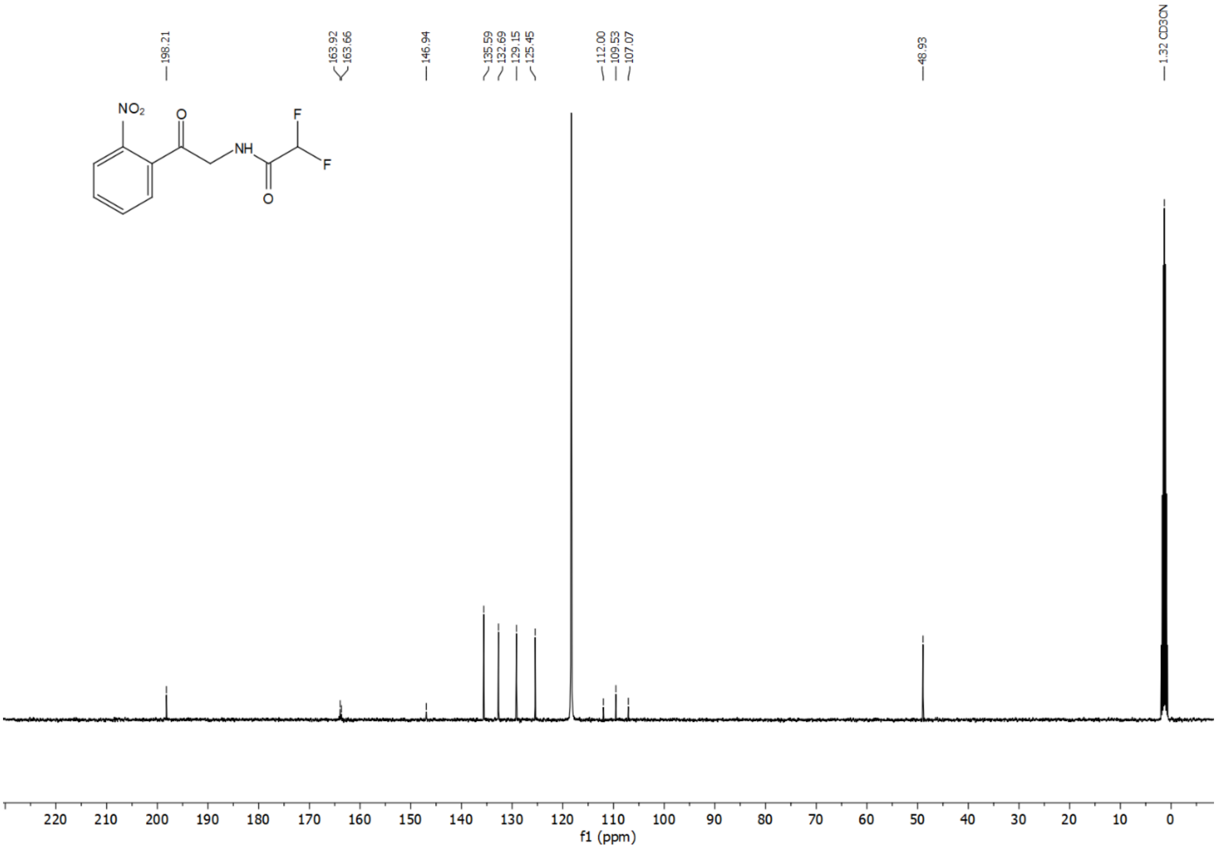

^{19}F NMR spectrum (376 MHz, CD_3CN) of **4p**.

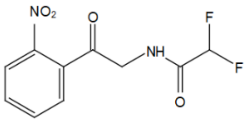

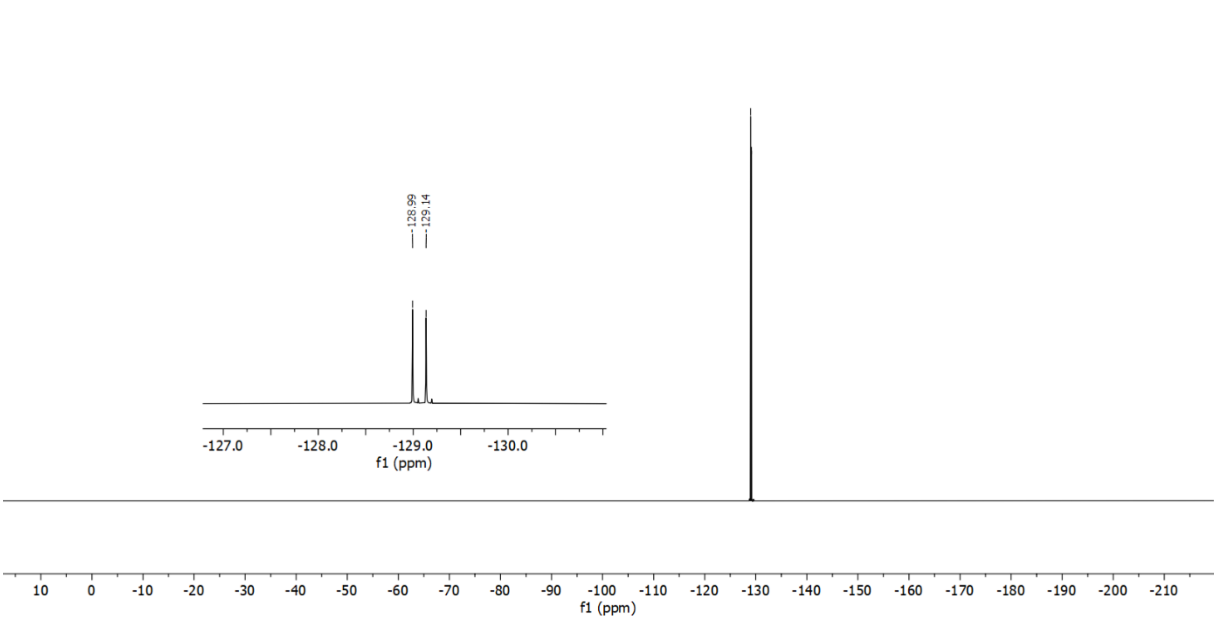

^{1}H NMR spectrum (400 MHz, CD_3CN) of **4q.**

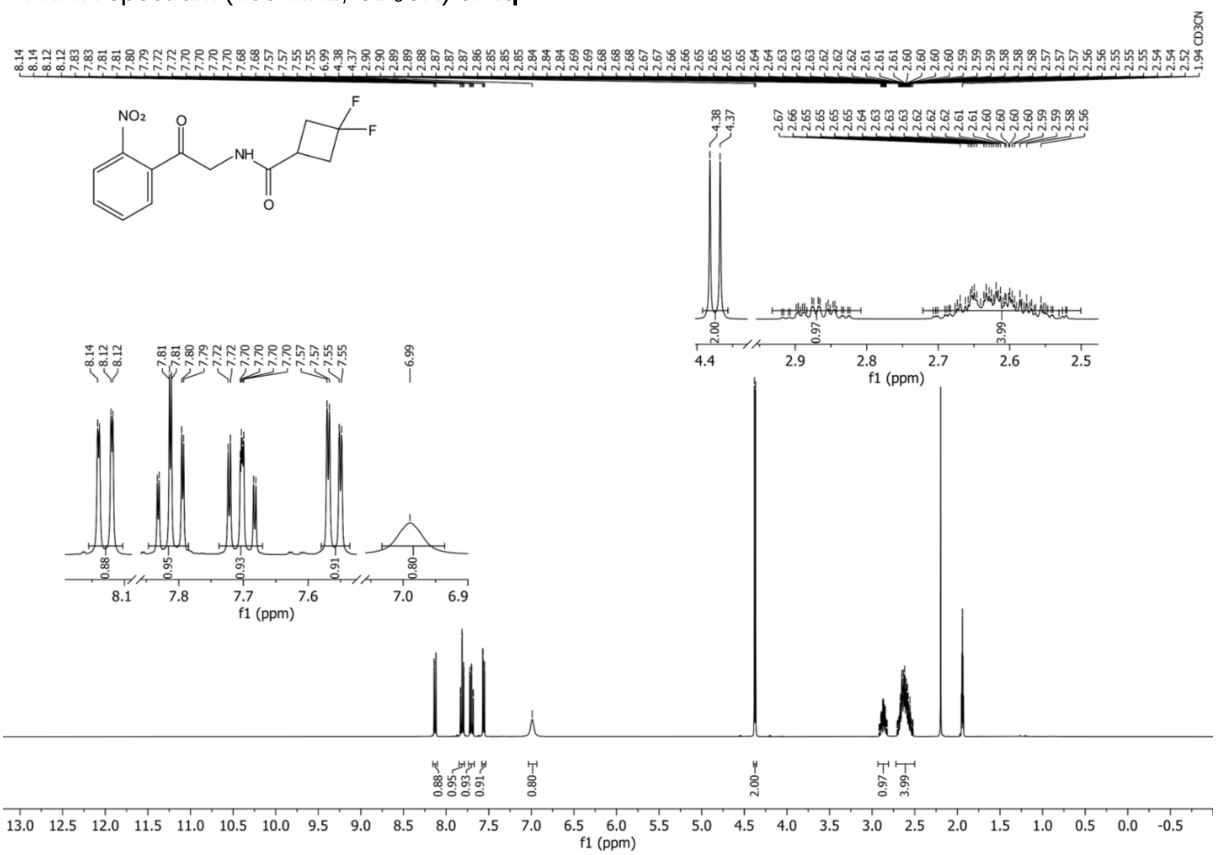

^{13}C NMR spectrum (101 MHz, CD_3CN) of **4q**.

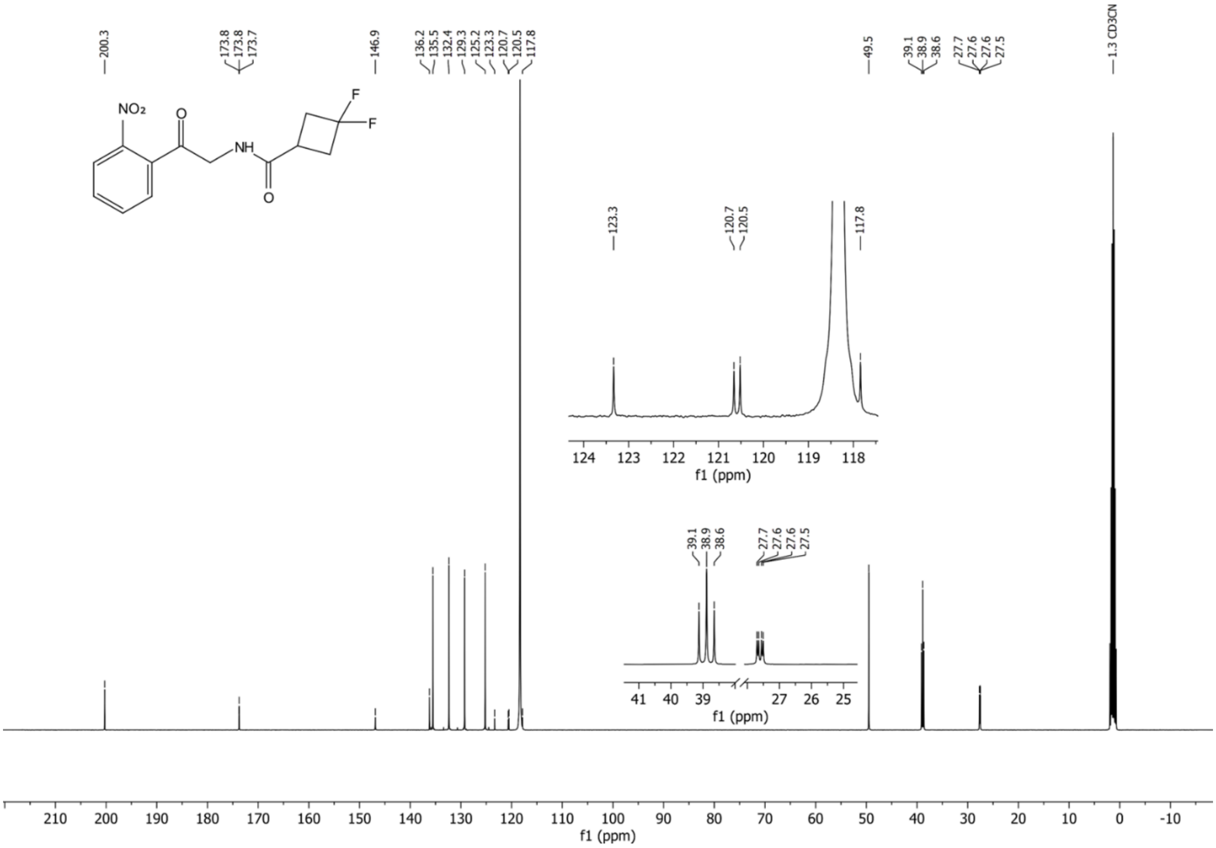

^{19}F NMR spectrum (376 MHz, CD_3CN) of **4q**.

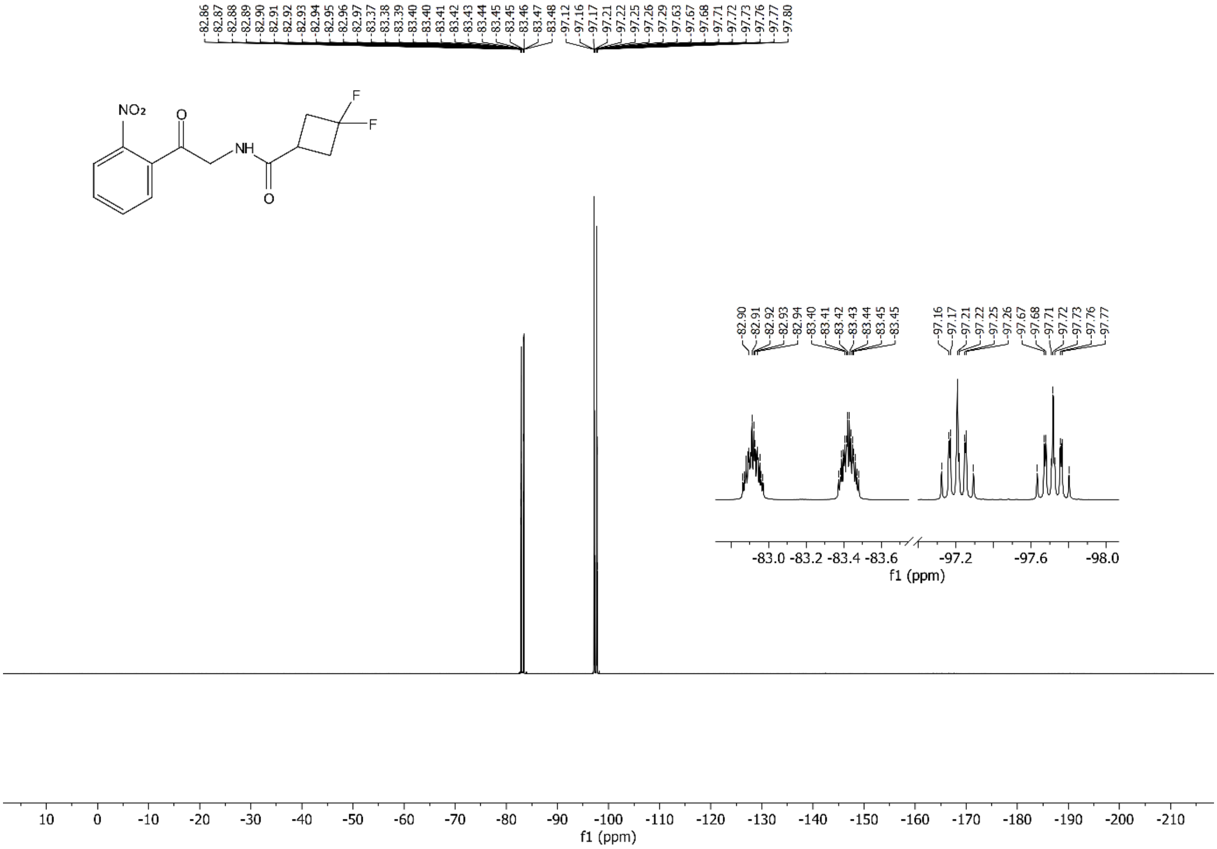

^1H NMR spectrum (600 MHz, CD_3CN) of **4r**.

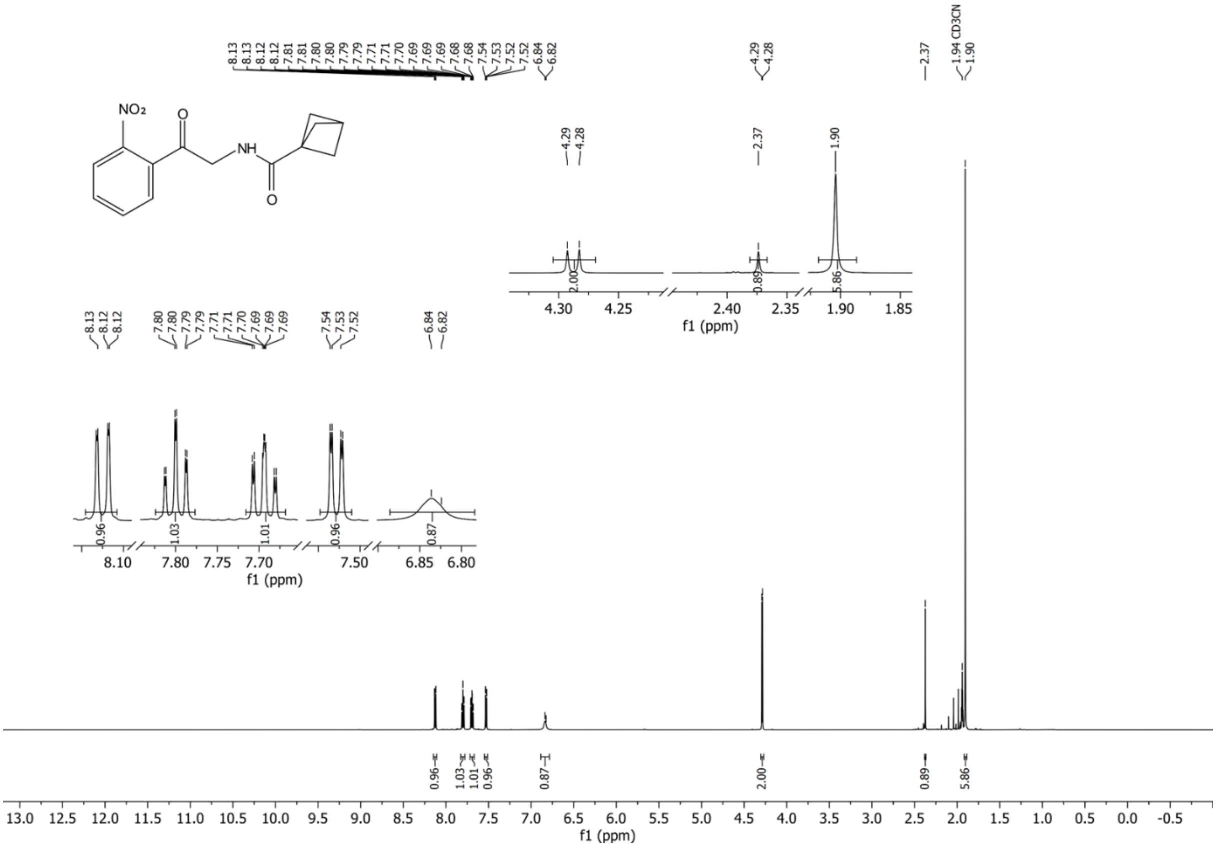

^{13}C NMR spectrum (151 MHz, CD_3CN) of **4r**.

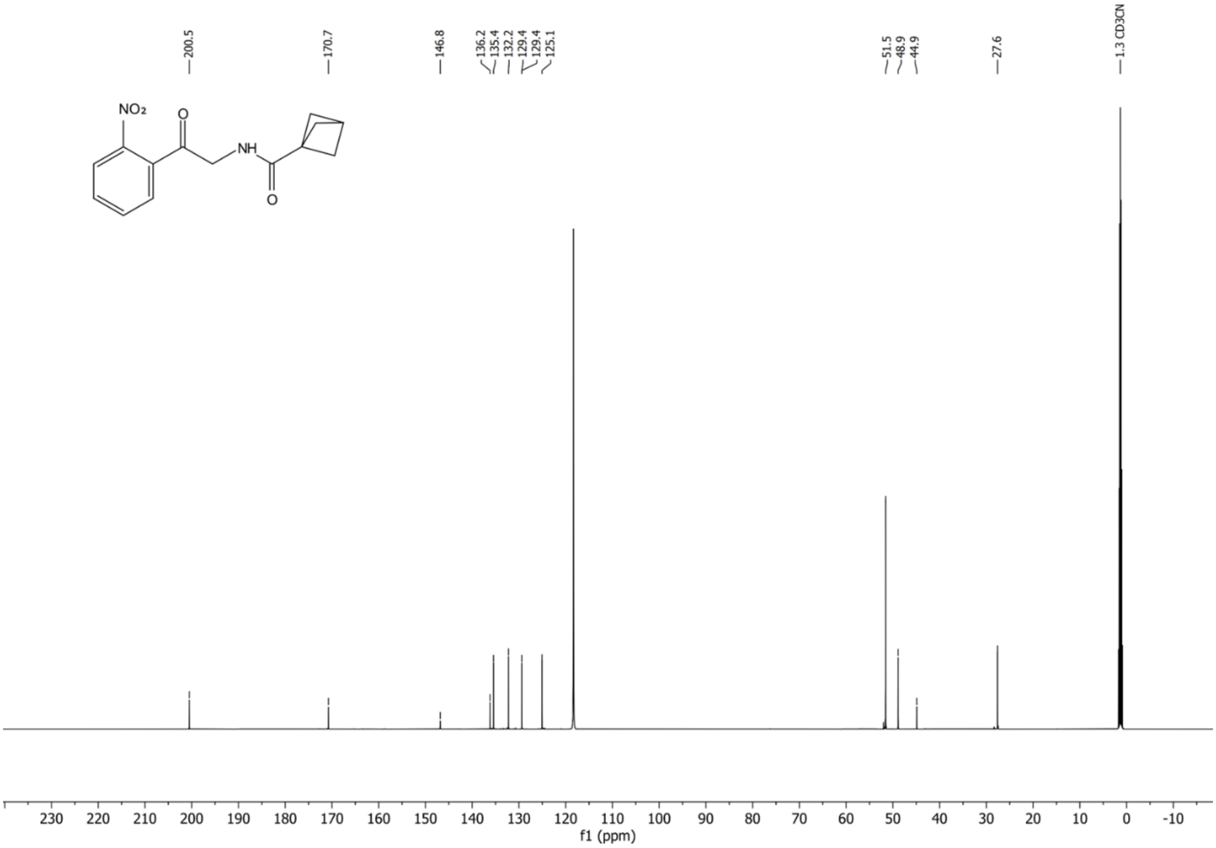

^{1}H NMR spectrum (600 MHz, $CDCl_3$) of **4s**.

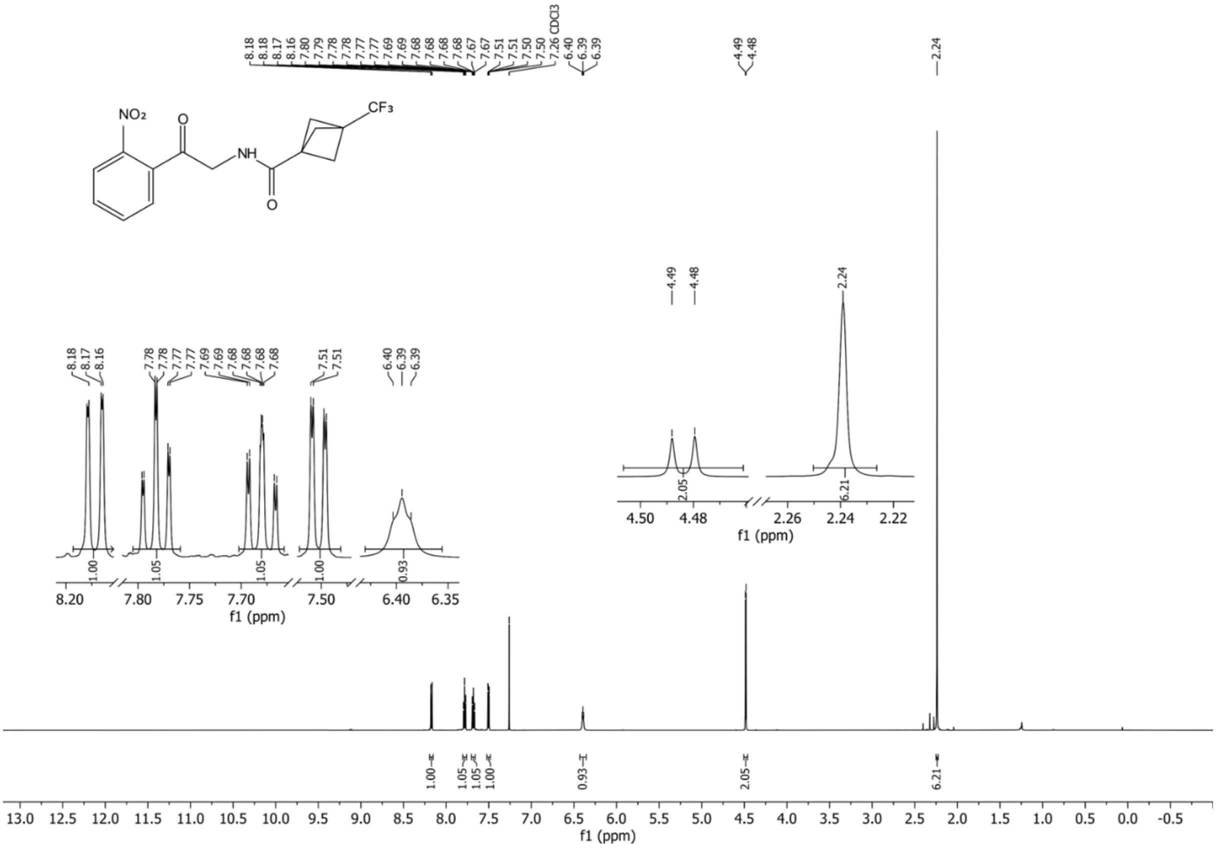

^{13}C NMR spectrum (151 MHz, $CDCl_3$) of **4s**.

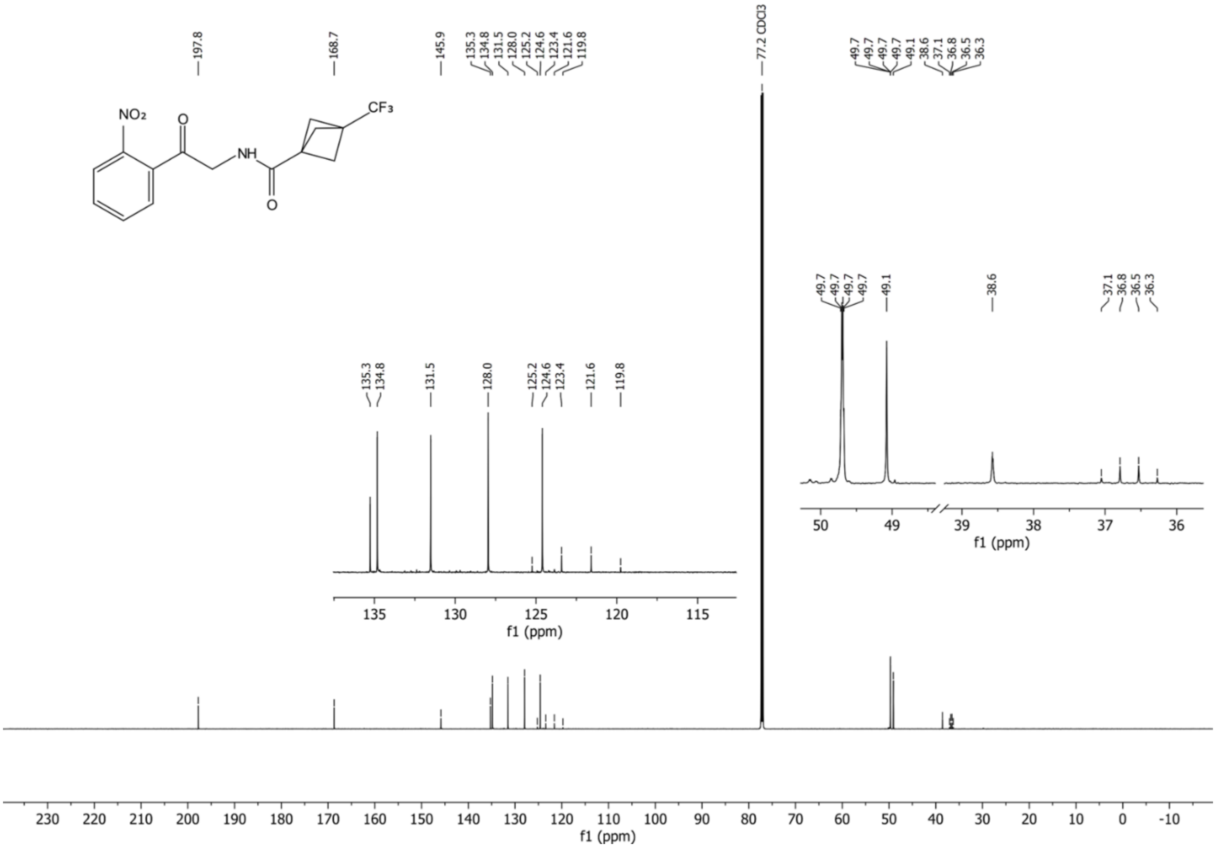

^{19}F NMR spectrum (282 MHz, CD_3CN) of **4s**.

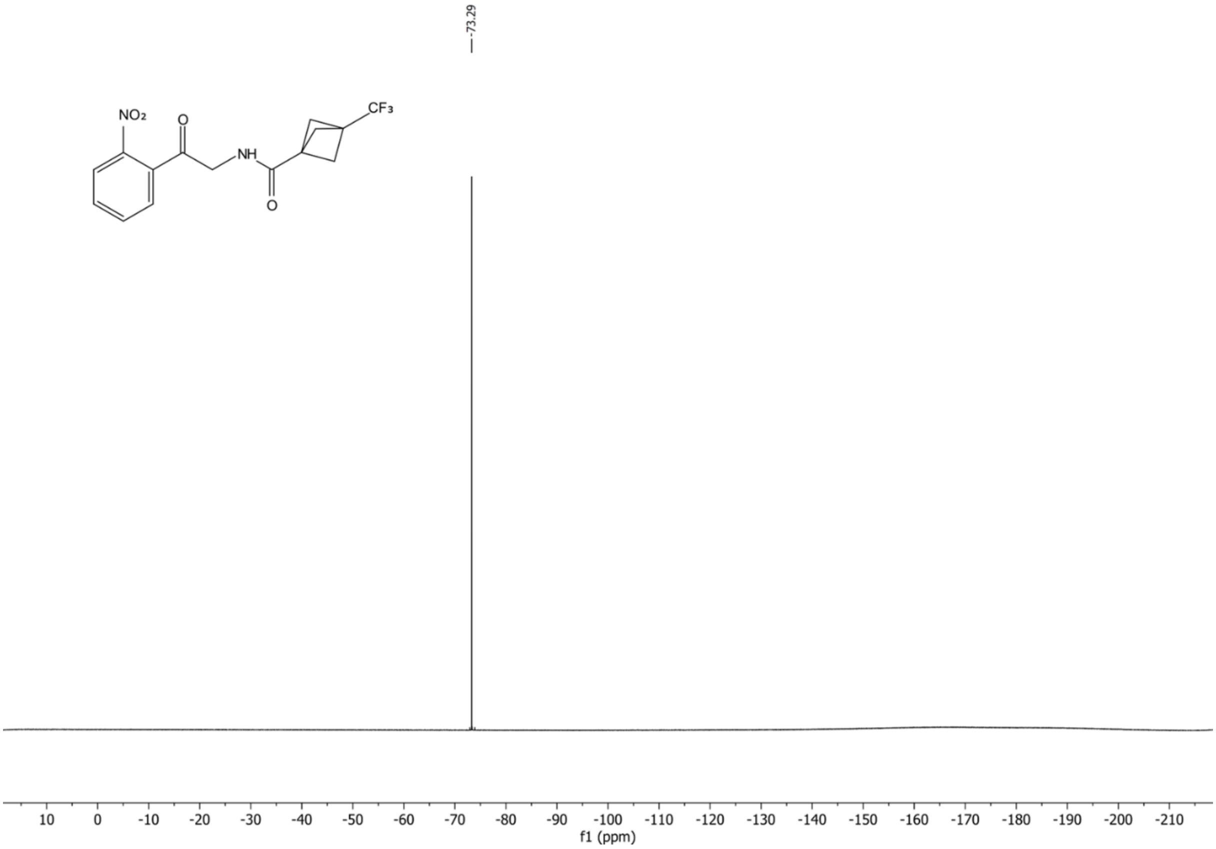

^{1}H NMR spectrum (600 MHz, CD_3CN) of **4t**.

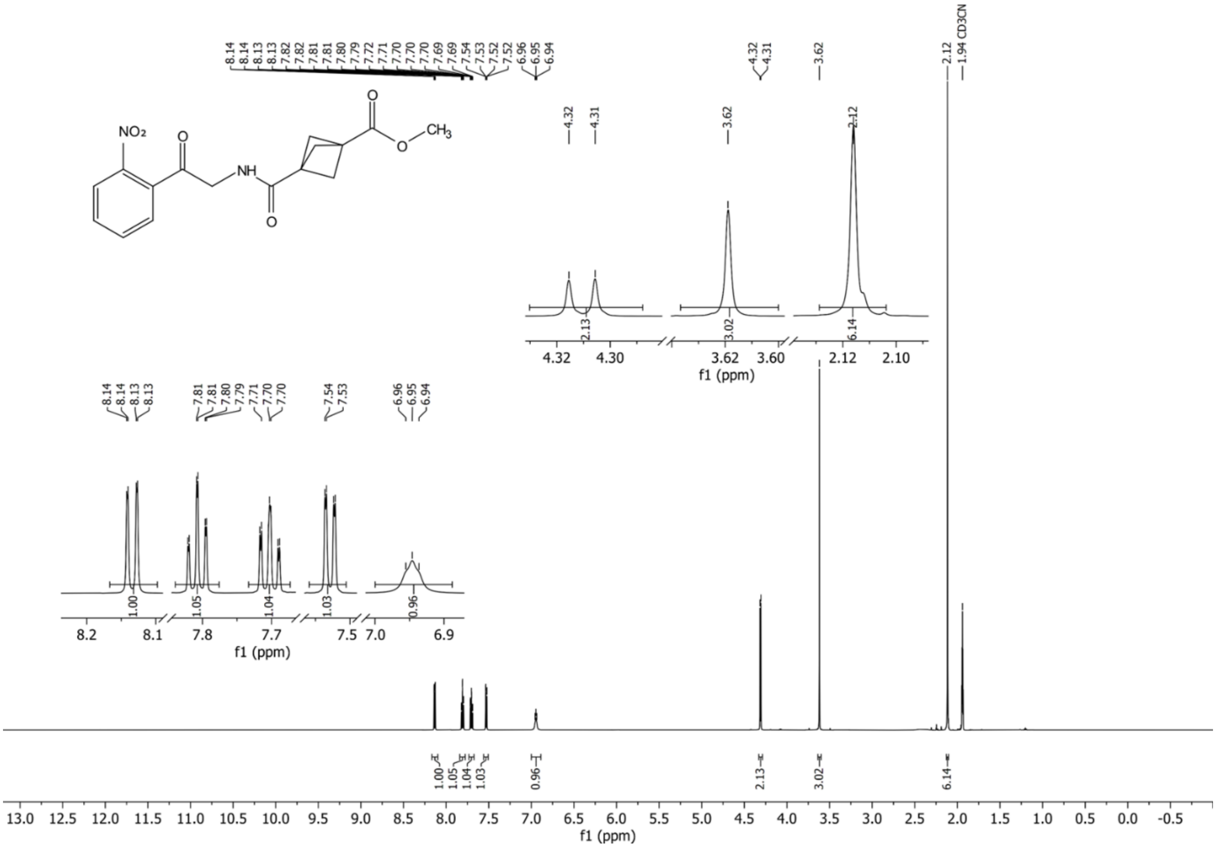

^{13}C NMR spectrum (151 MHz, CD_3CN) of **4t**.

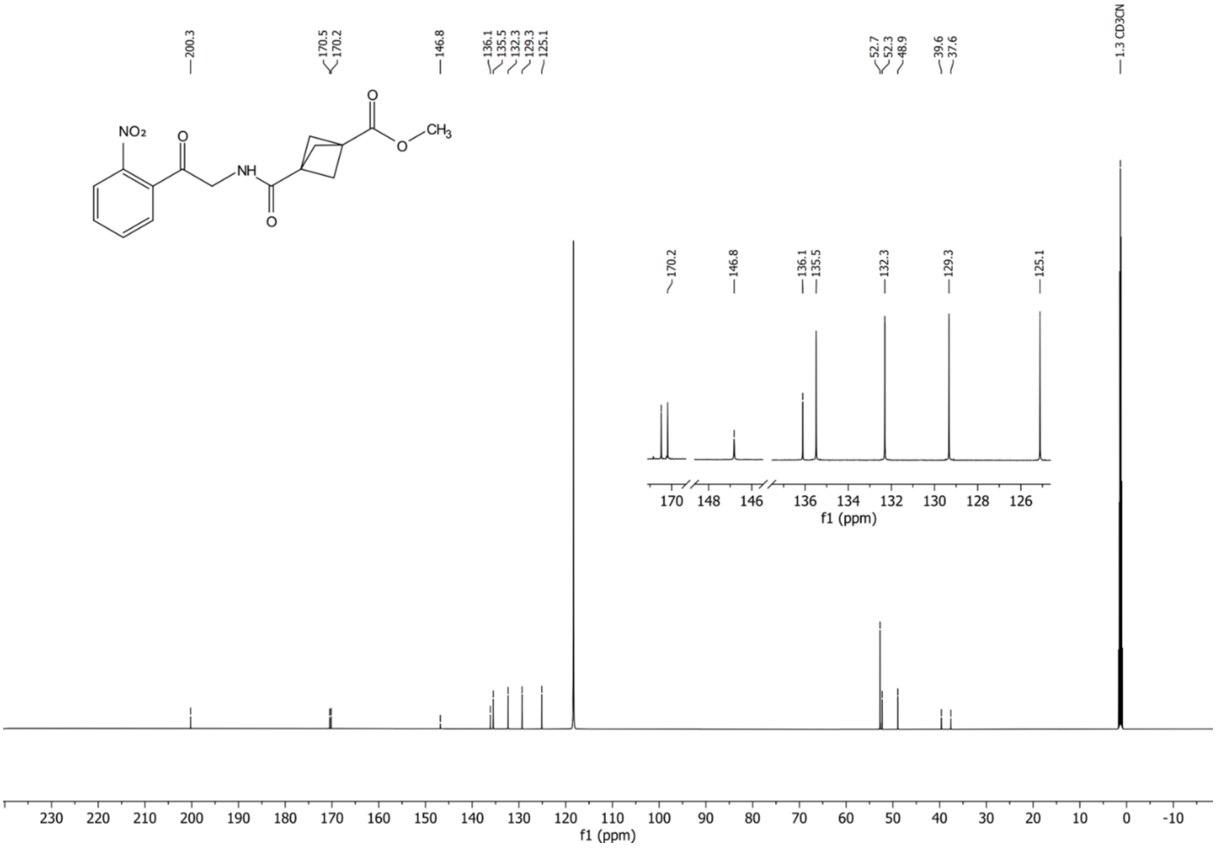

^{1}H NMR spectrum (400 MHz, CD_2Cl_2) of **4u**.

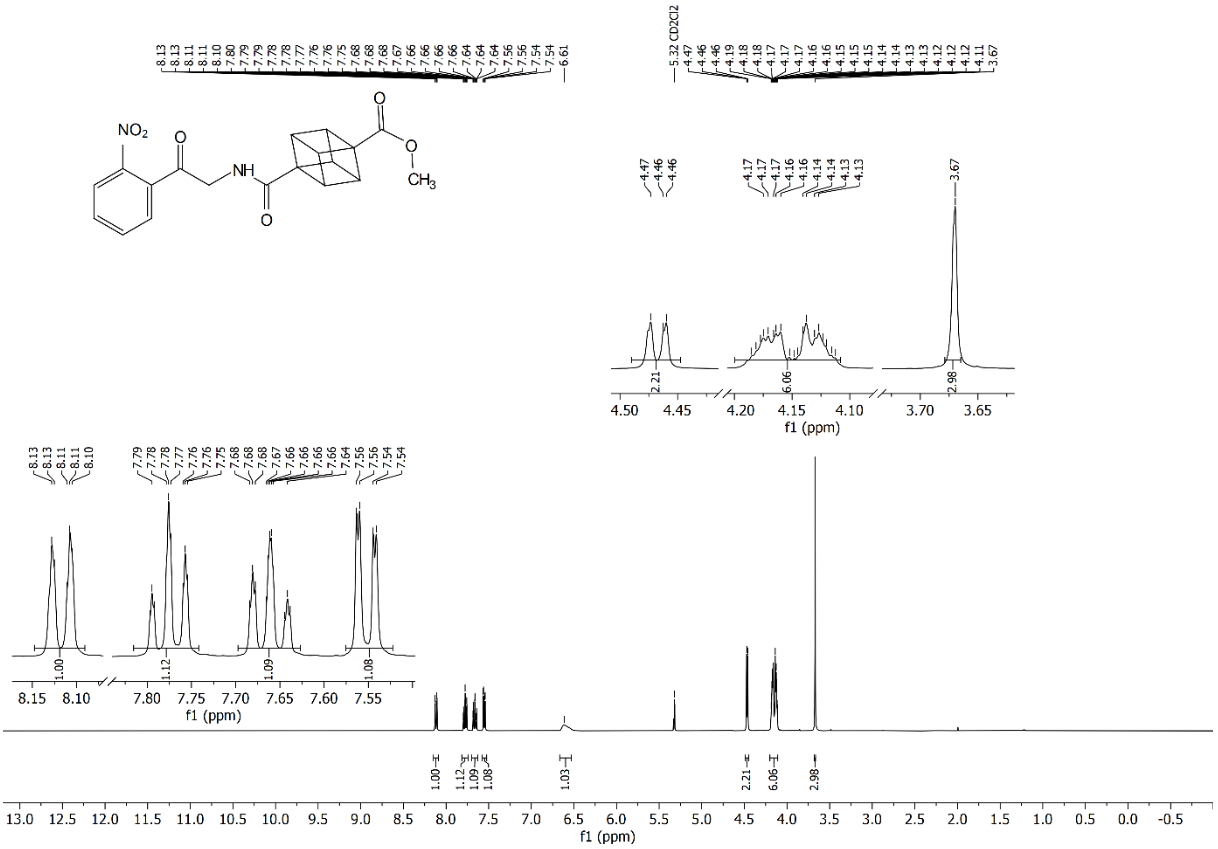

^{13}C NMR spectrum (101 MHz, CD_2Cl_2) of **4u**.

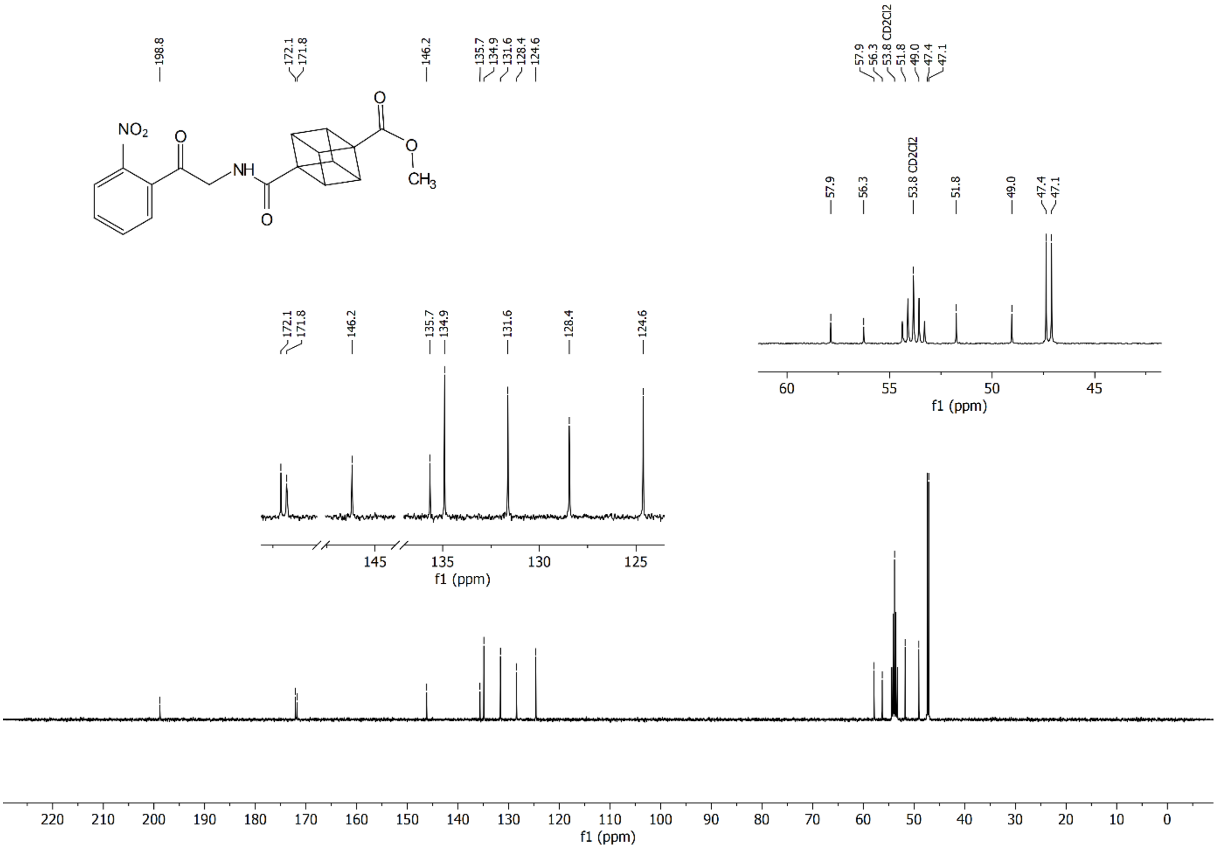

^{1}H NMR spectrum (400 MHz, CD_2Cl_2) of **4v**.

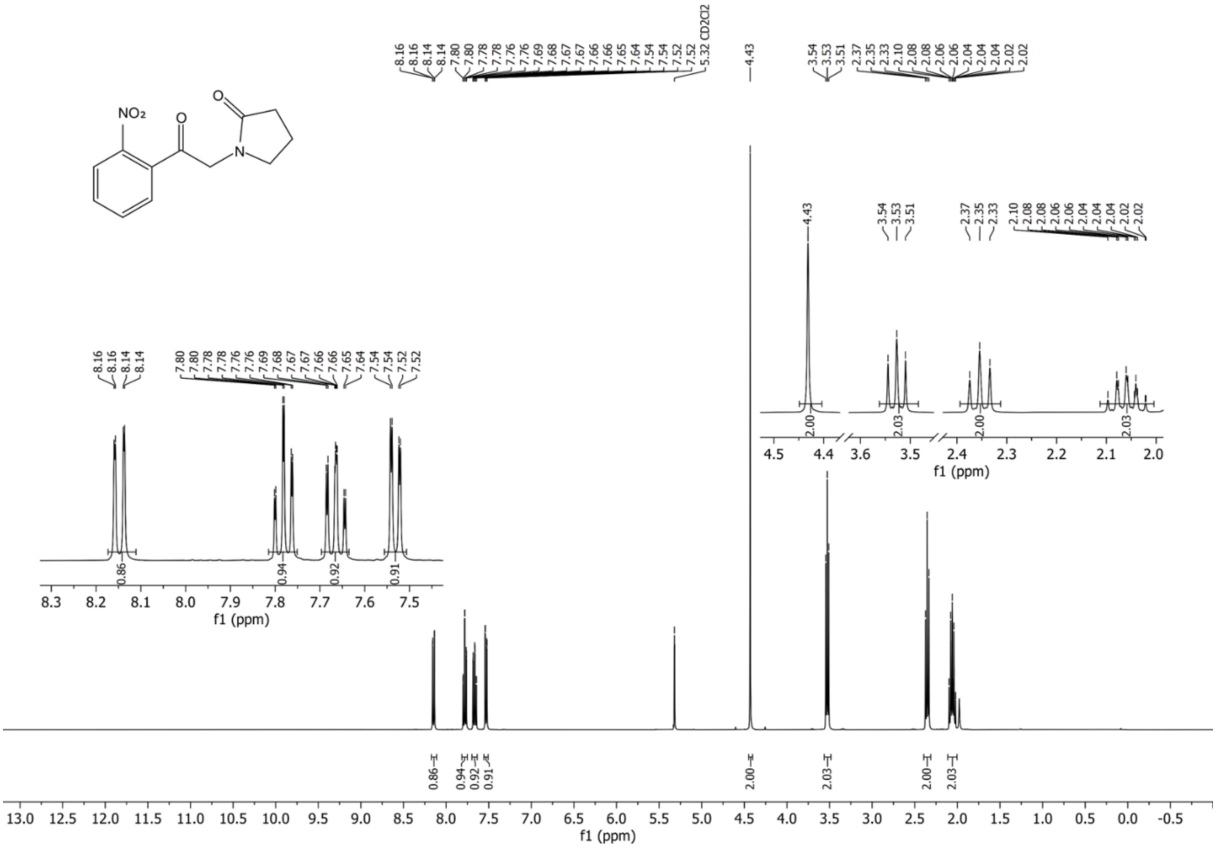

^{13}C NMR spectrum (101 MHz, CD_2Cl_2) of **4v**.

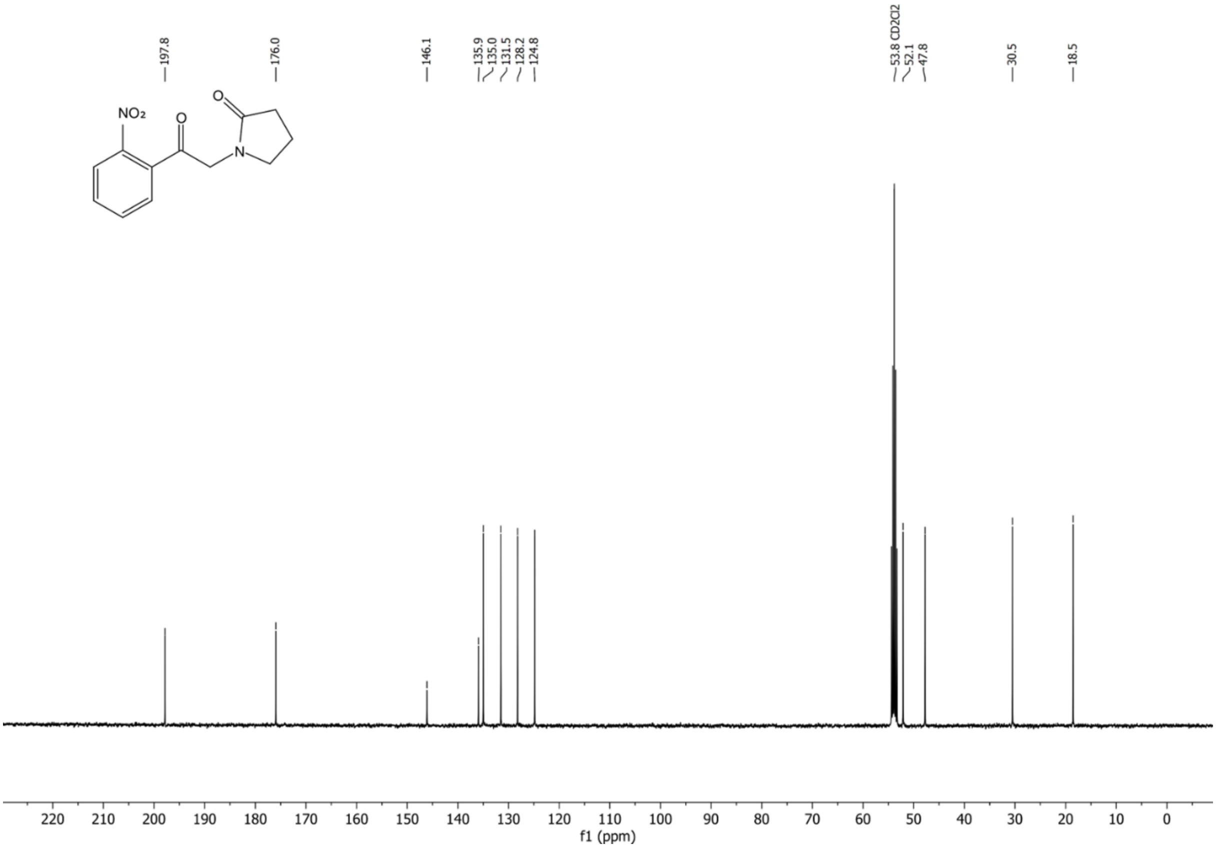

^{1}H NMR spectrum (400 MHz, $CDCl_3$) of **4w**.

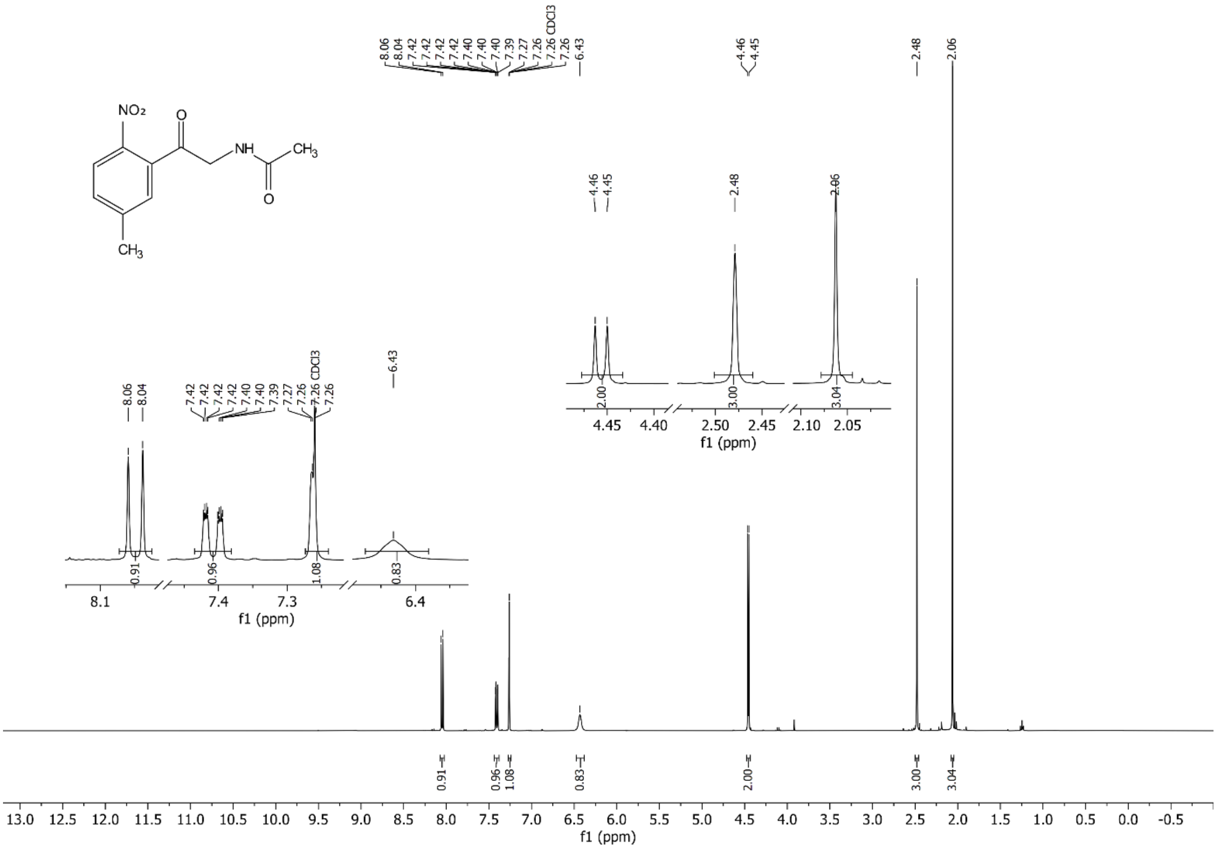

^{13}C NMR spectrum (101 MHz, $CDCl_3$) of **4w**.

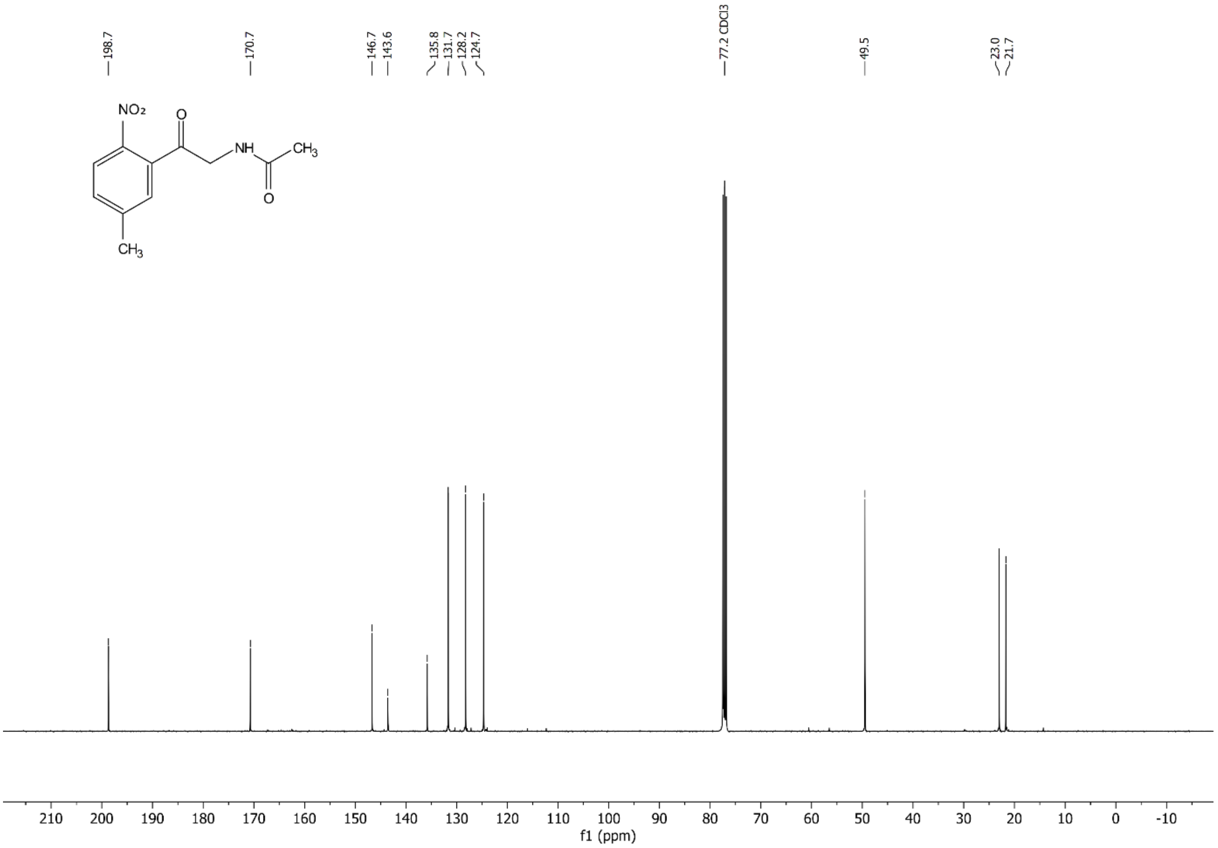

^{1}H NMR spectrum (400 MHz, CD_3CN) of **4x**.

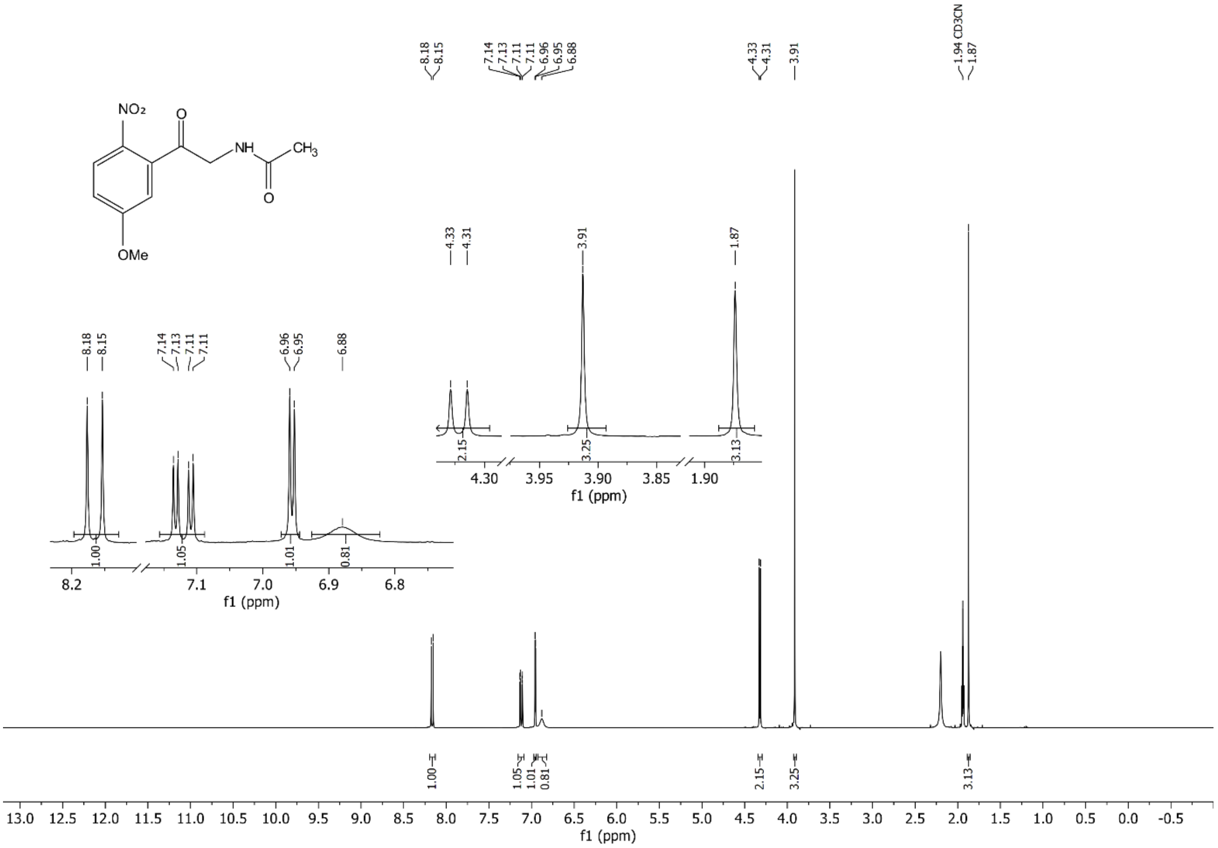

^{13}C NMR spectrum (101 MHz, CD_3CN) of **4x**.

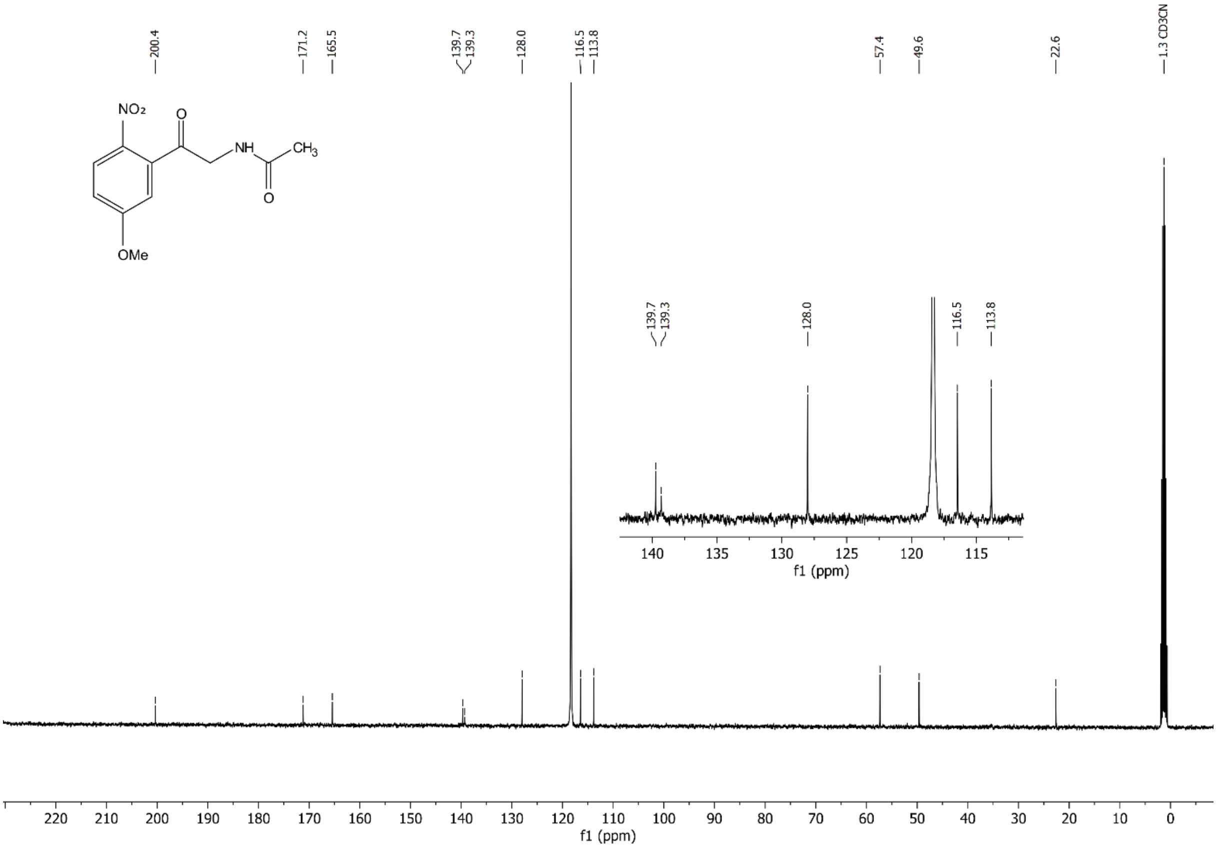

^{1}H NMR spectrum (400 MHz, CD_2Cl_2) of **4y**.

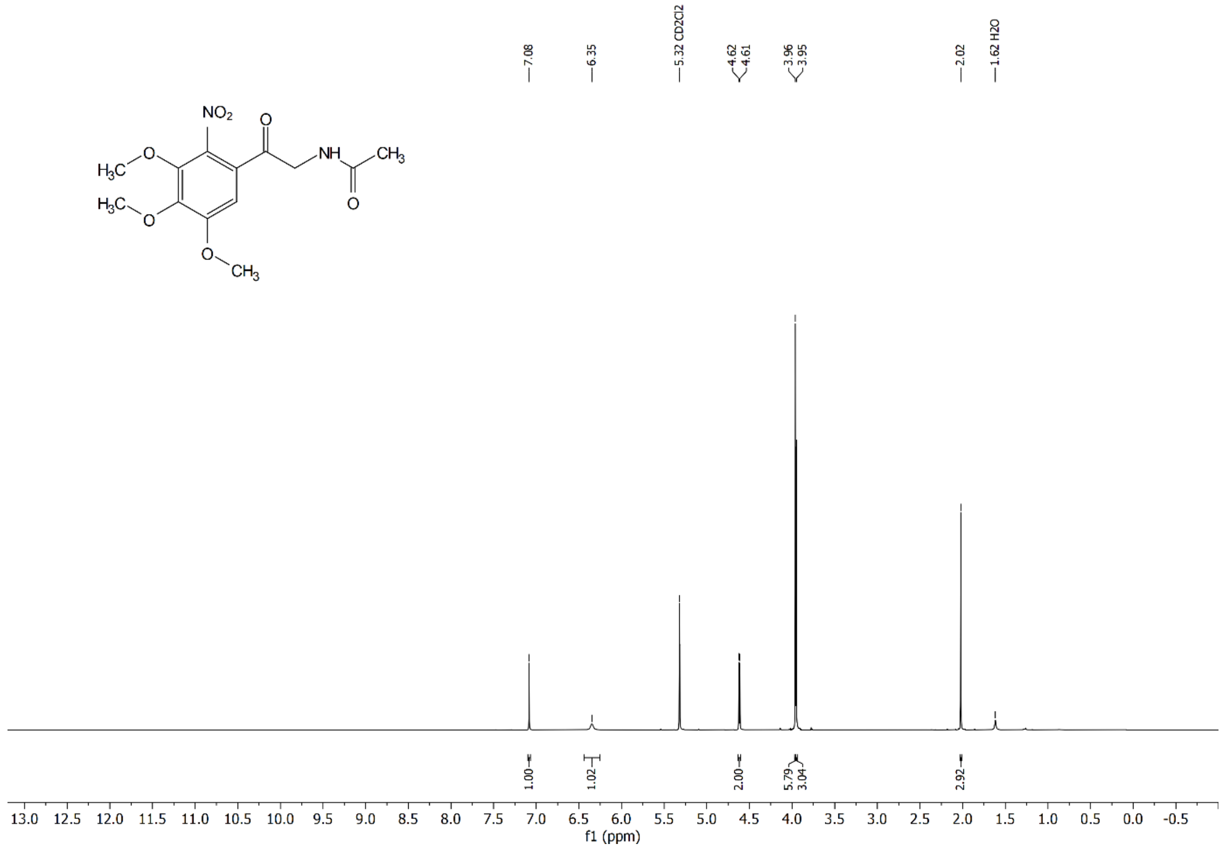

^{13}C NMR spectrum (101 MHz, CD_2Cl_2) of **4y**.

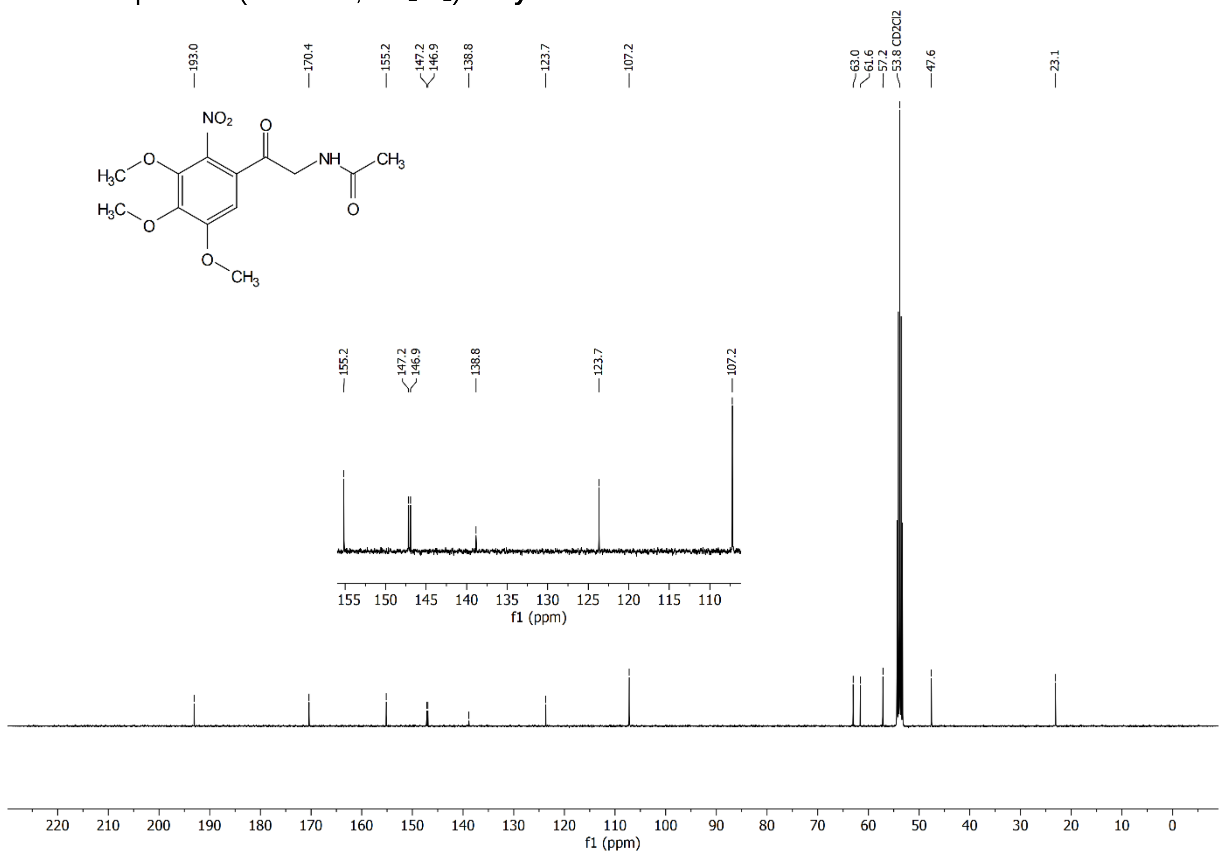

^1H NMR spectrum (400 MHz, CD_3CN) of **4z**.

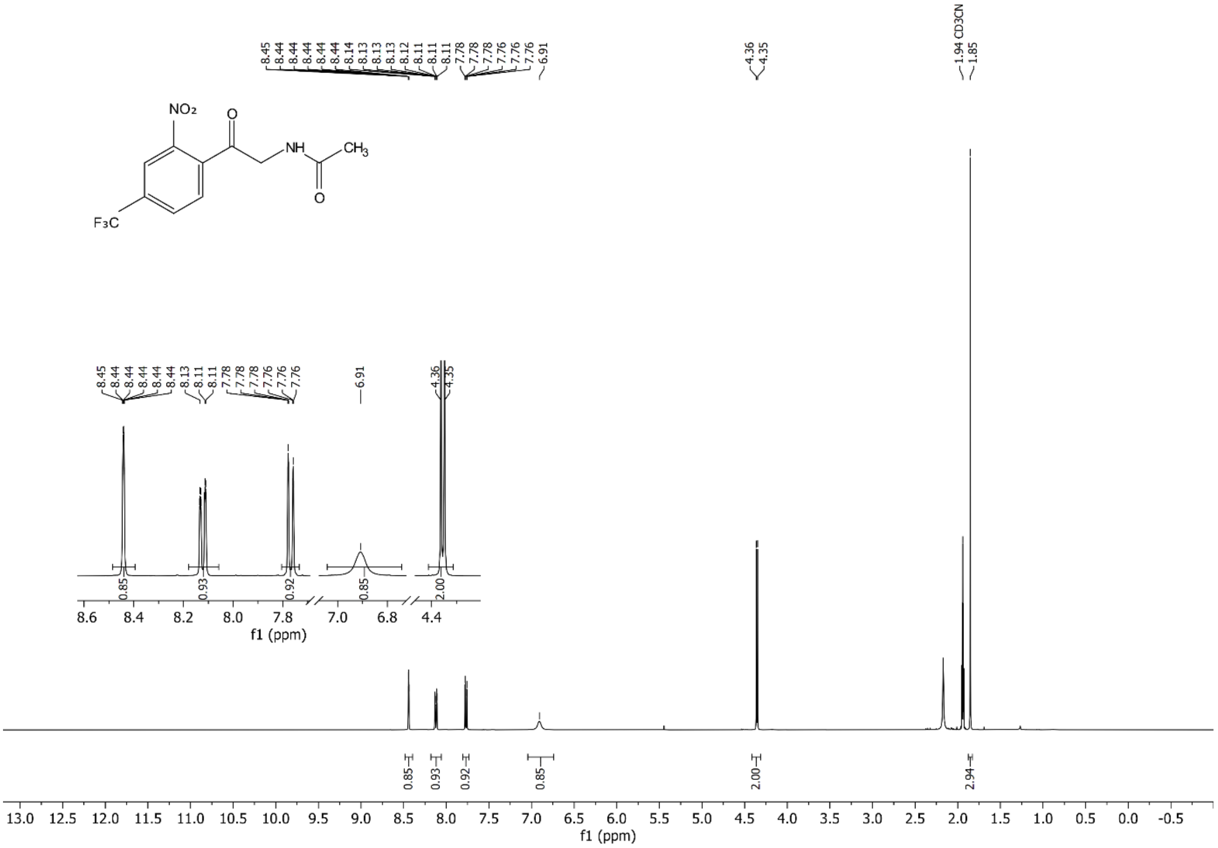

^{13}C NMR spectrum (101 MHz, CD_3CN) of **4z**.

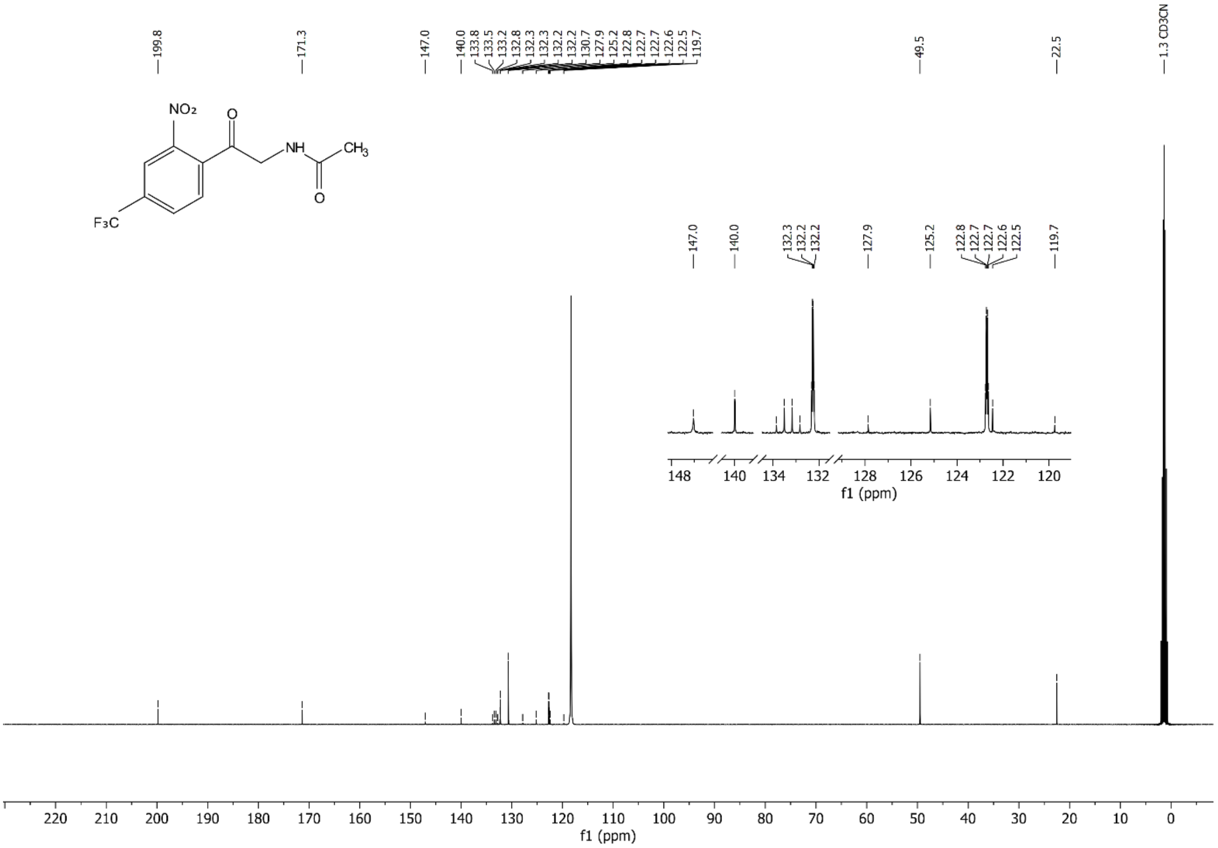

^{19}F NMR spectrum (377 MHz, CD_3CN) of **4z**.

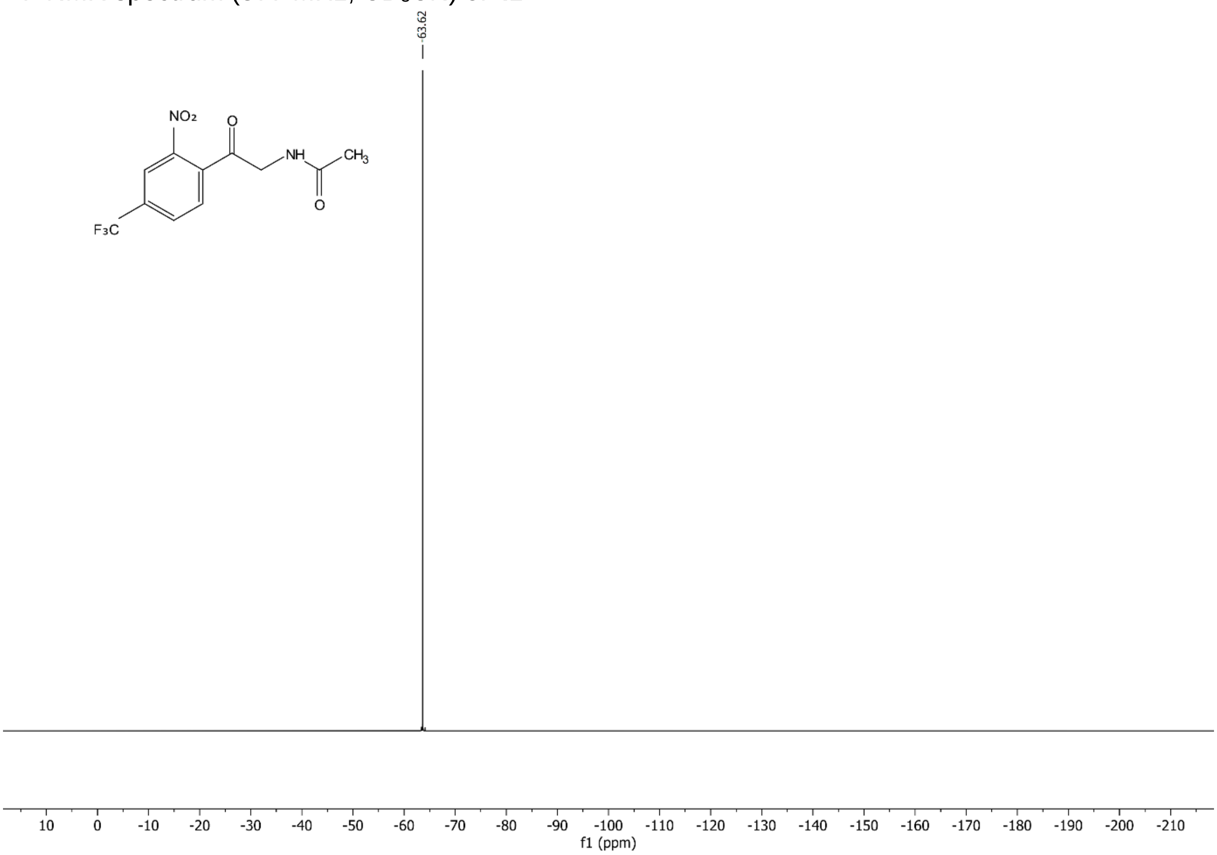

^{1}H NMR spectrum (400 MHz, CD_3CN) of **4aa**.

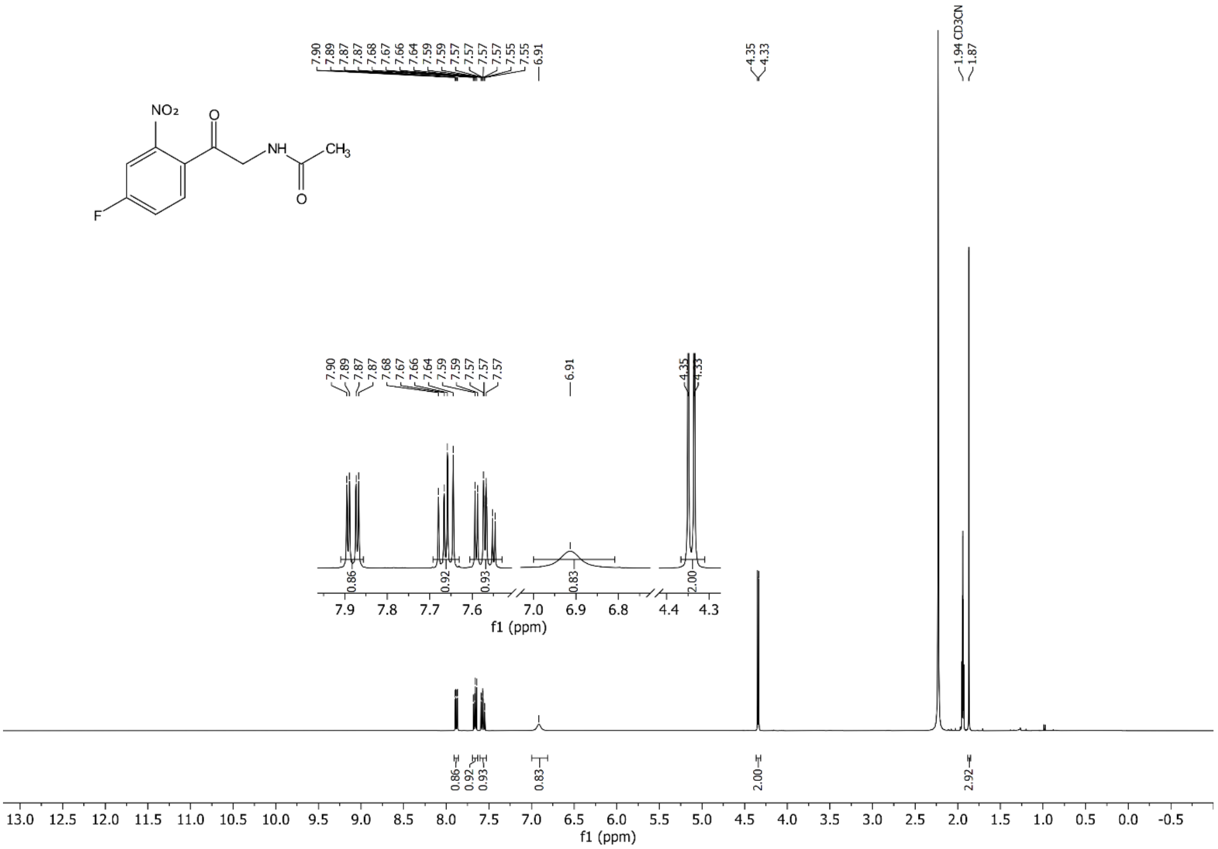

^{13}C NMR spectrum (101 MHz, CD_3CN) of **4aa**.

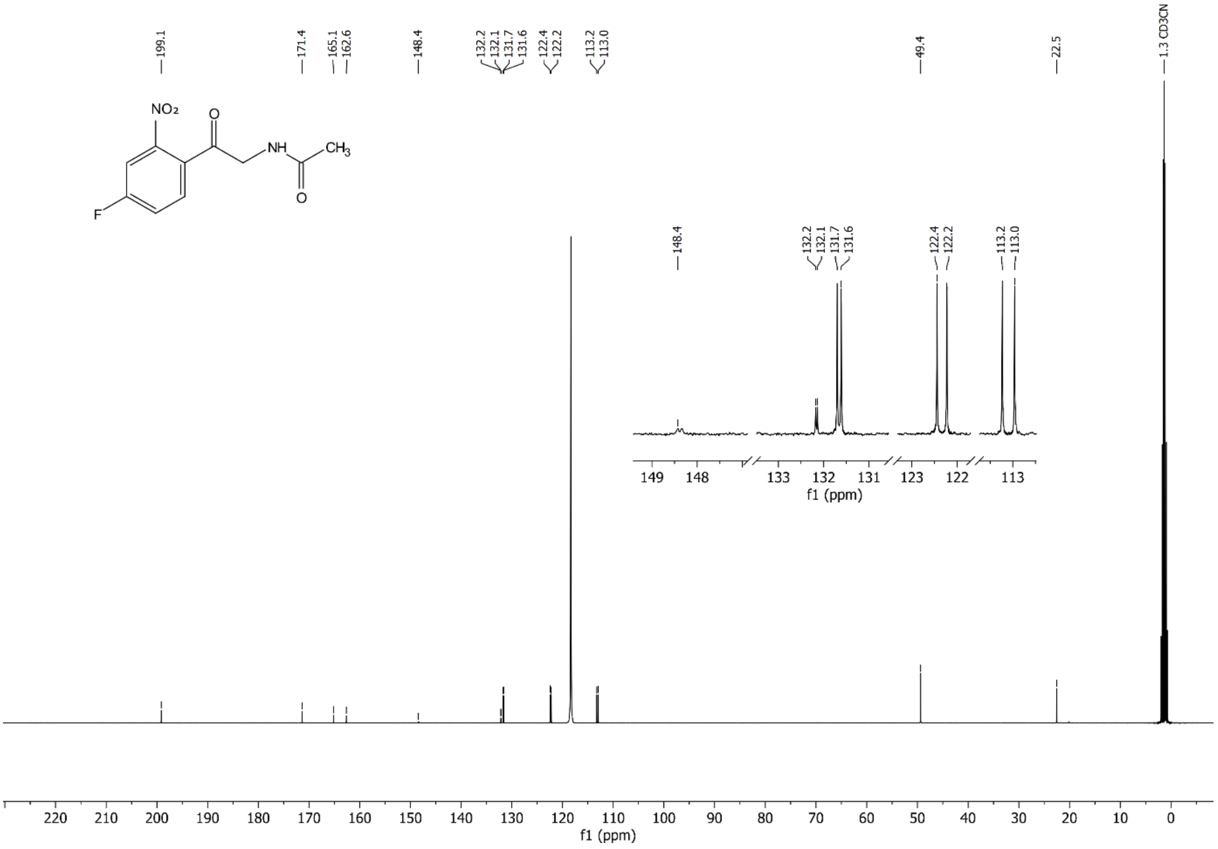

^{19}F NMR spectrum (377 MHz, CD_3CN) of **4aa**.

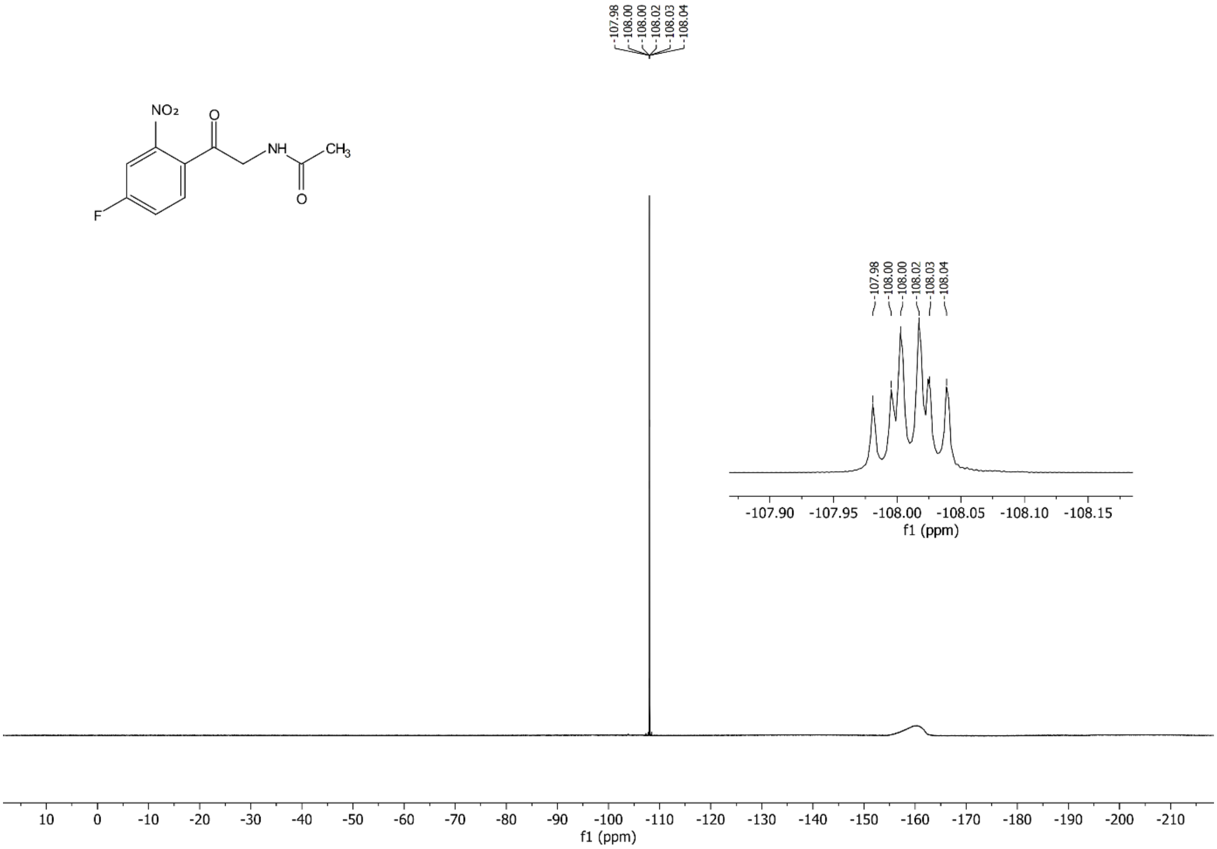

^{1}H NMR spectrum (400 MHz, CD_3CN) of **4ab**.

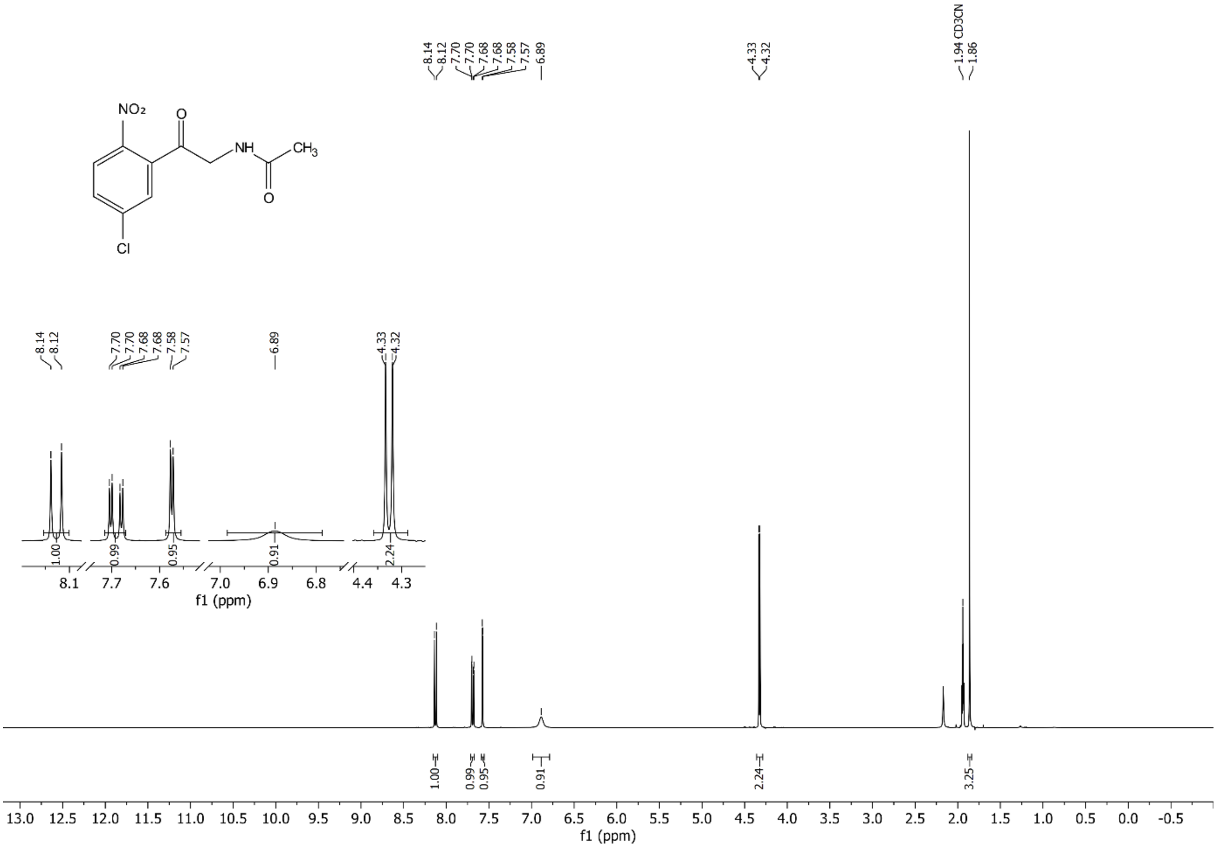

^{13}C NMR spectrum (101 MHz, CD_3CN) of **4ab**.

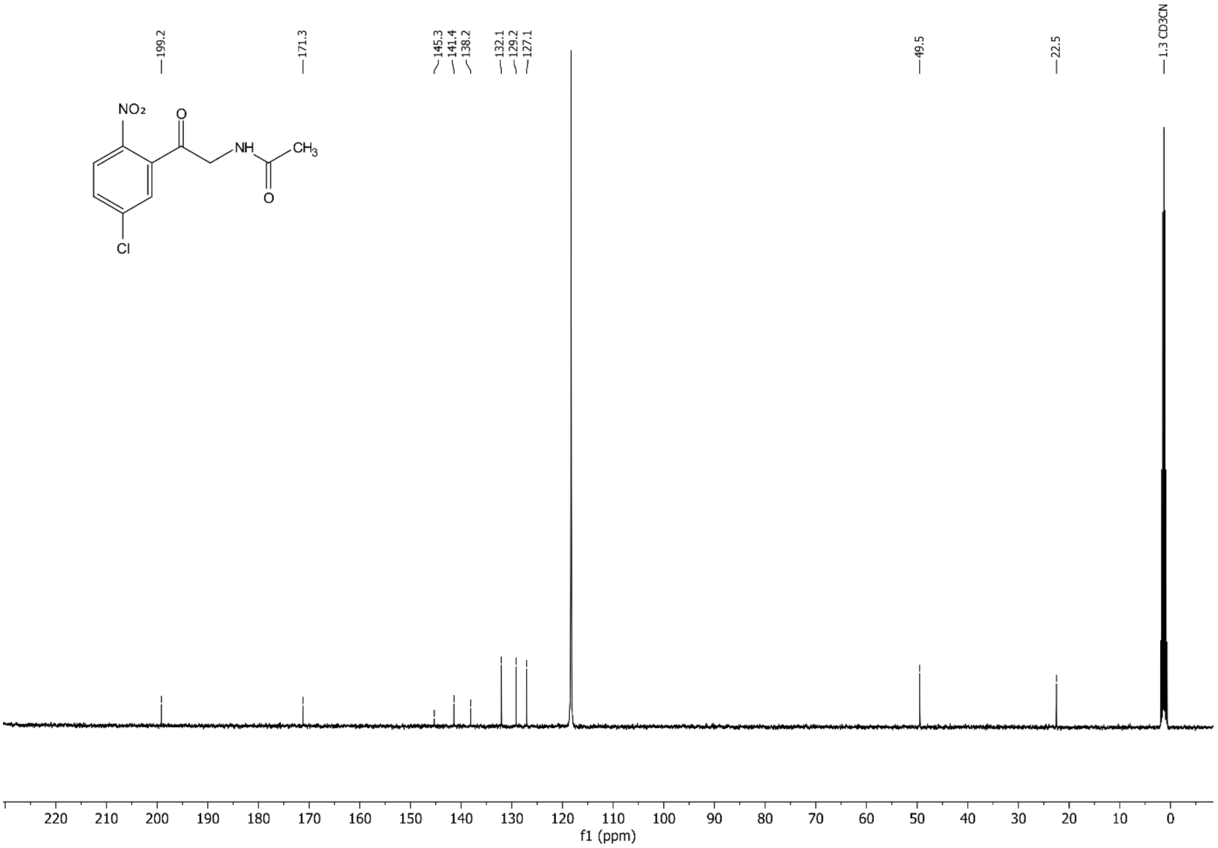

^{1}H NMR spectrum (400 MHz, $CDCl_3$) of **4ac**.

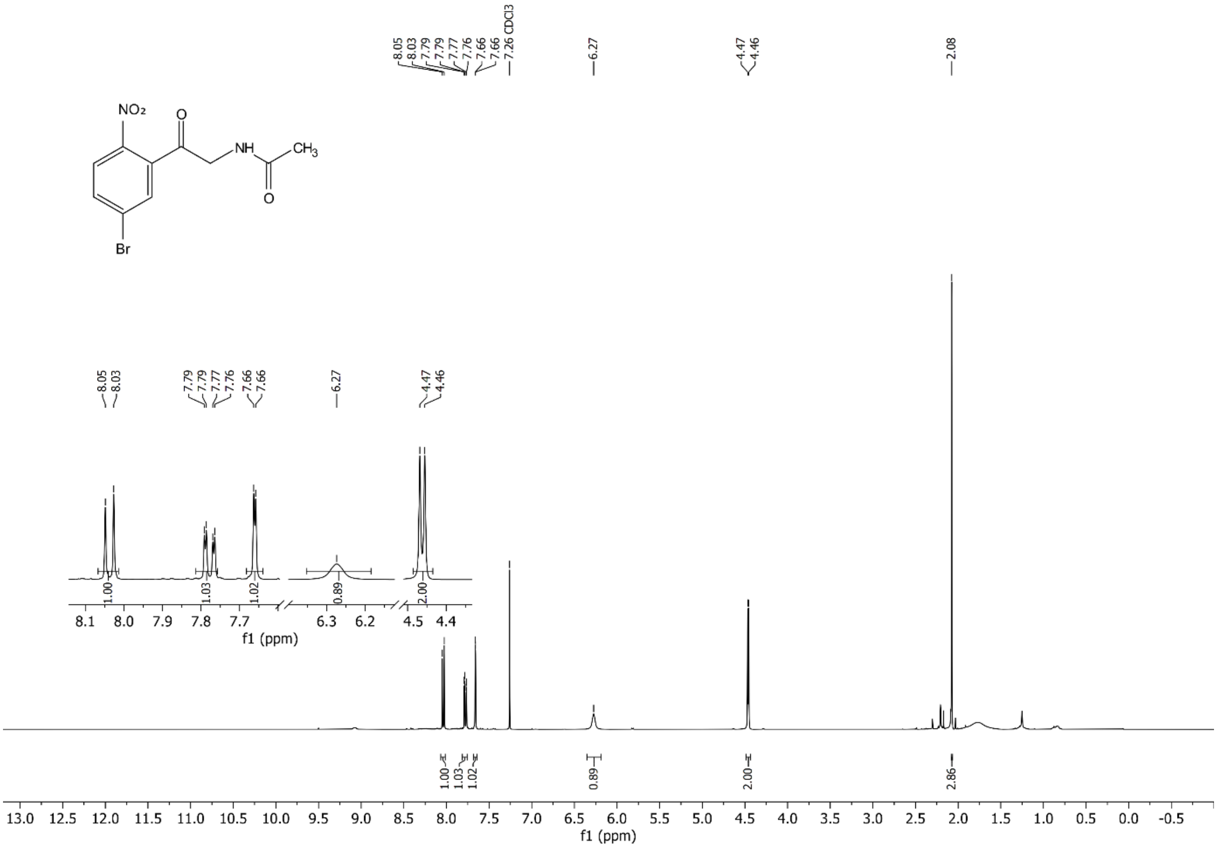

^{13}C NMR spectrum (101 MHz, $CDCl_3$) of **4ac**.

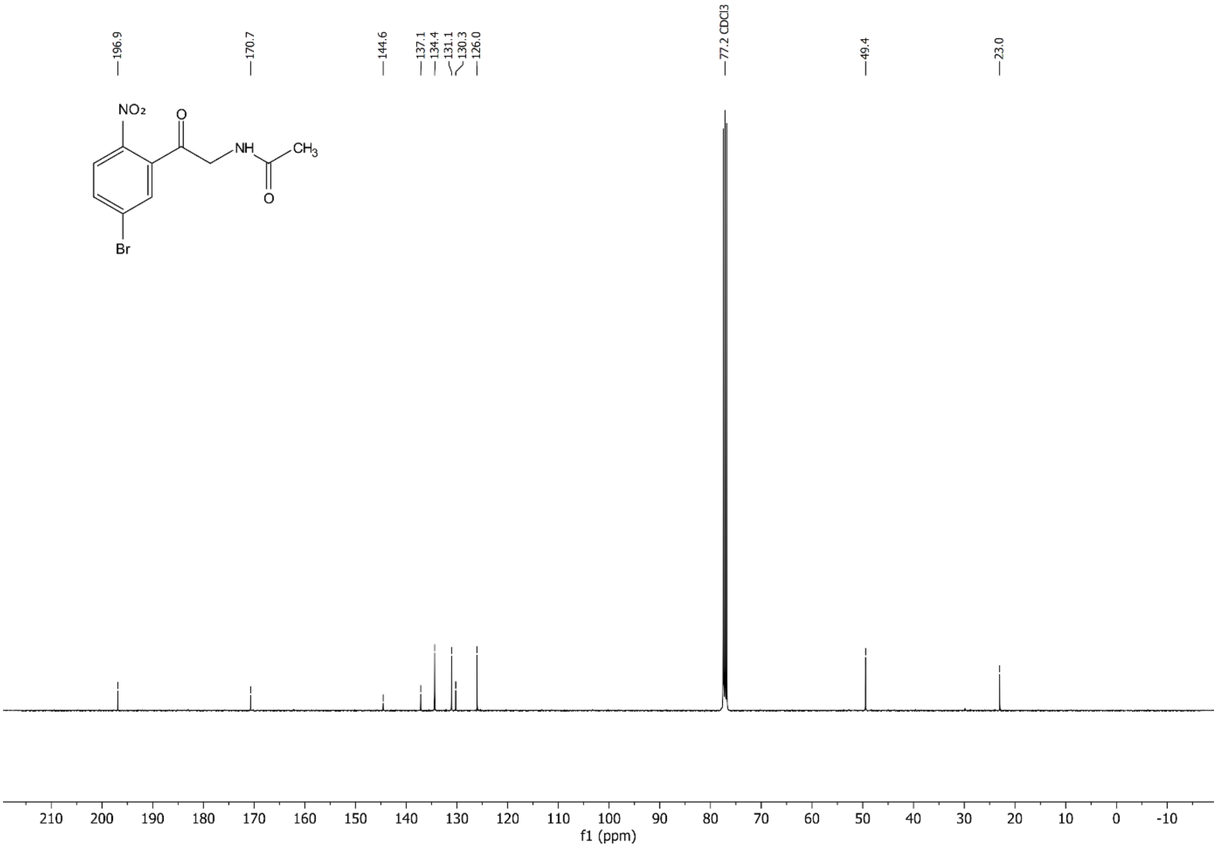

^{1}H NMR spectrum (400 MHz, CD_3CN) of **4ad**.

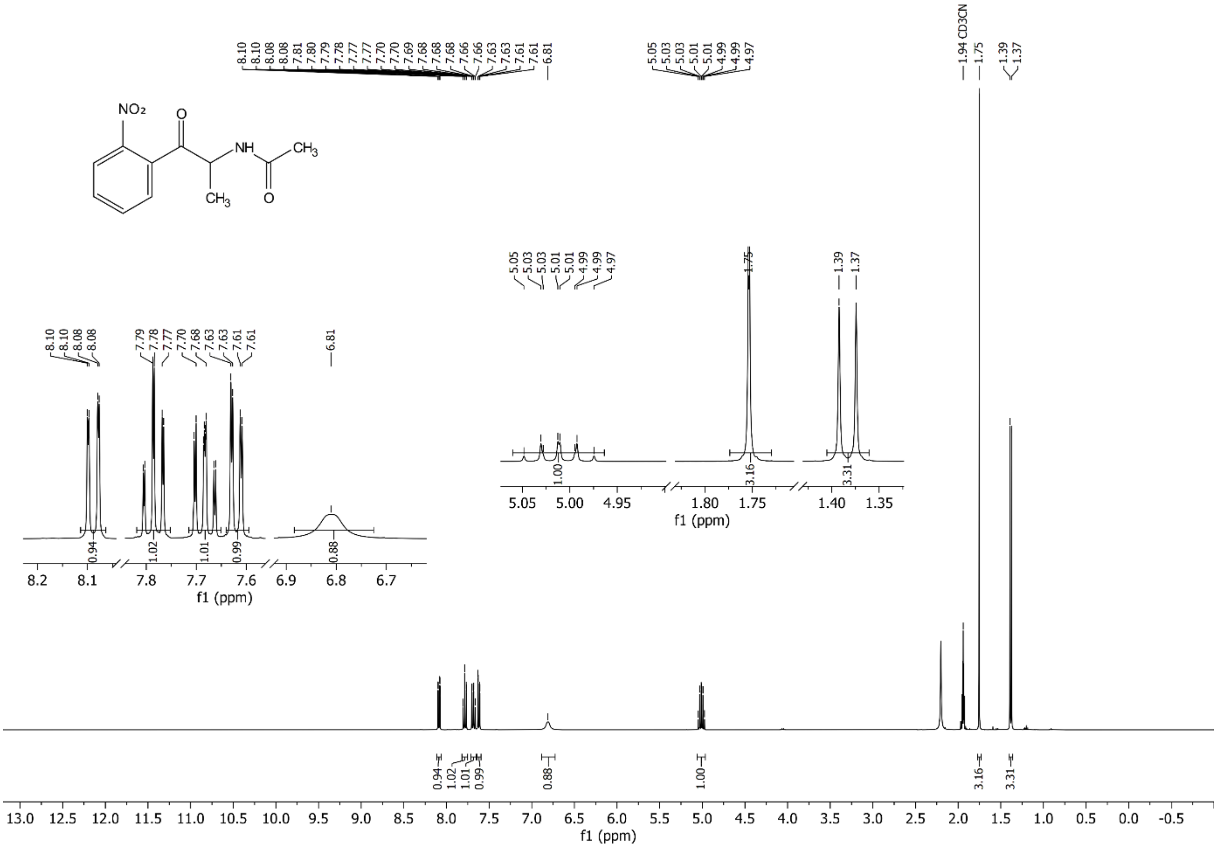

^{13}C NMR spectrum (101 MHz, CD_3CN) of **4ad**.

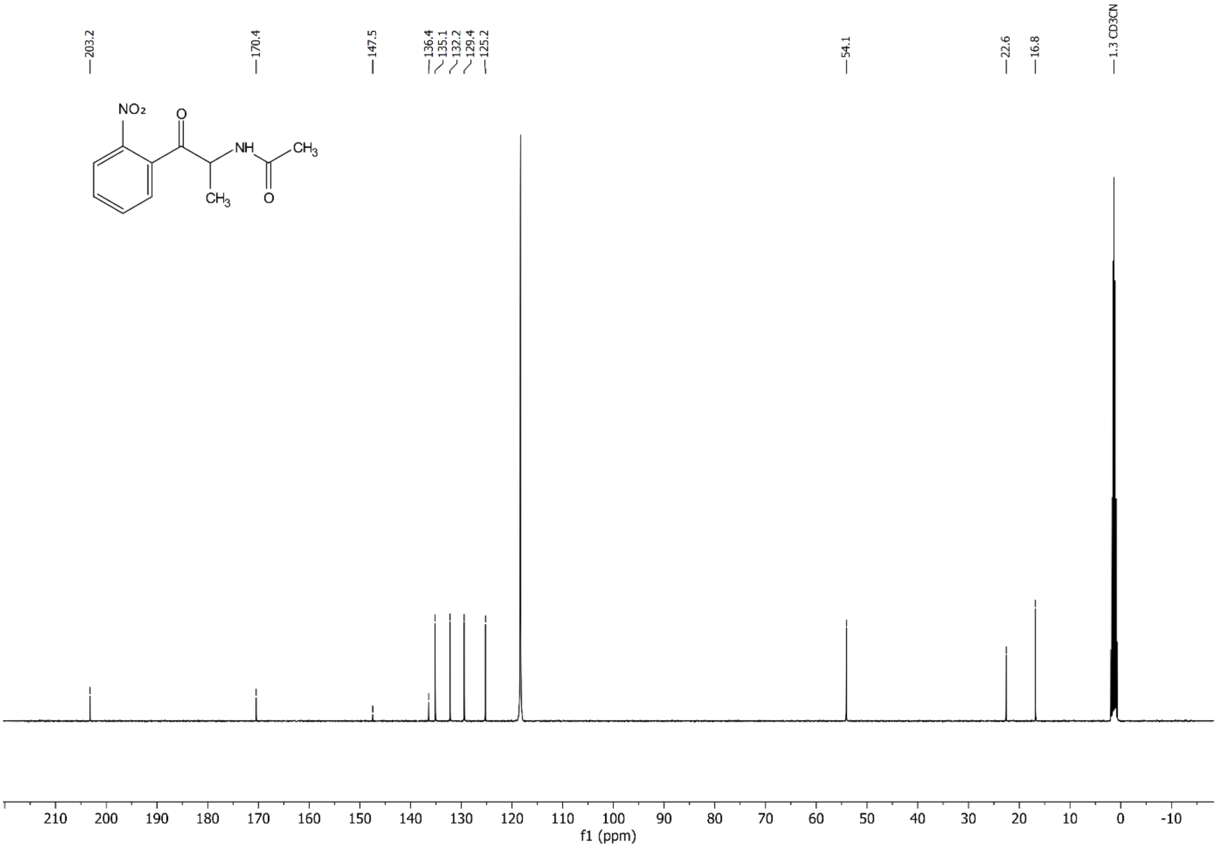

^{1}H NMR spectrum (600 MHz, CD_3CN) of **4ae**.

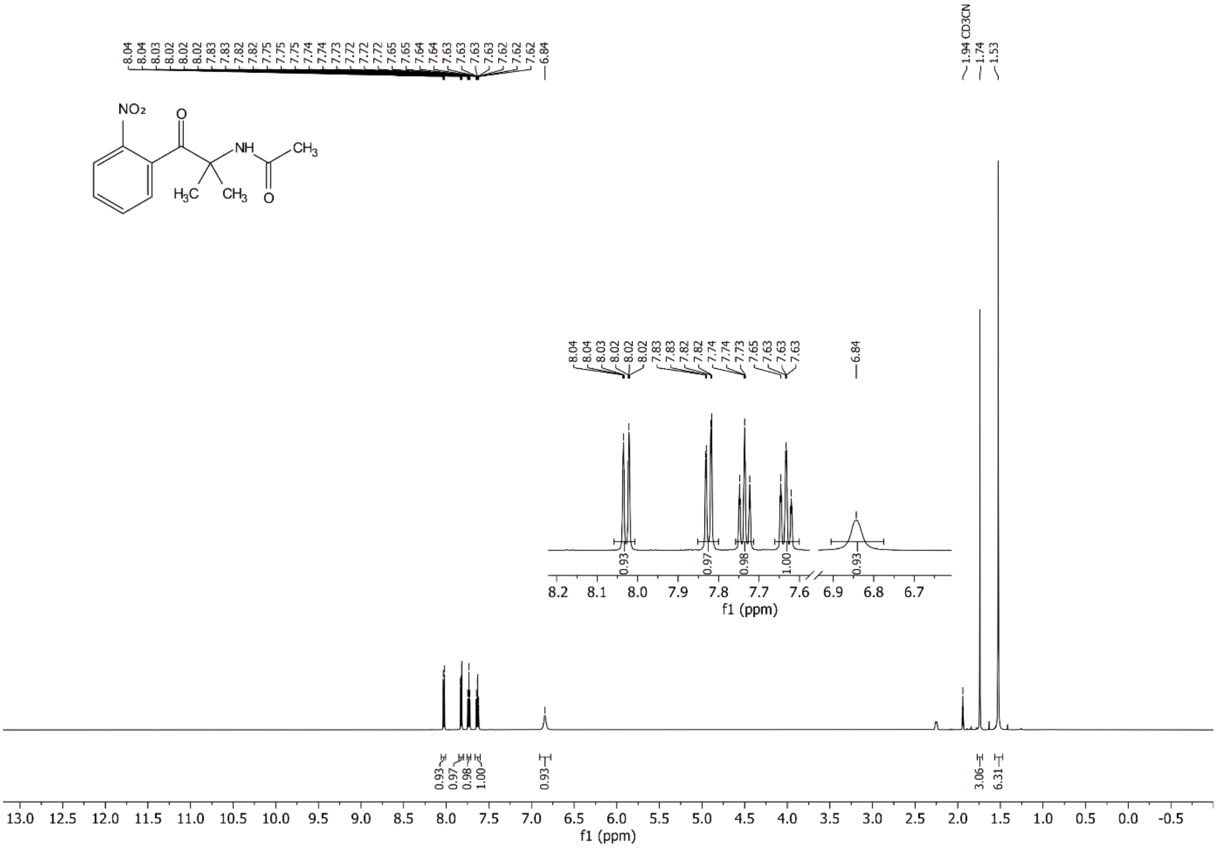

^{13}C NMR spectrum (151 MHz, CD_3CN) of **4ae**.

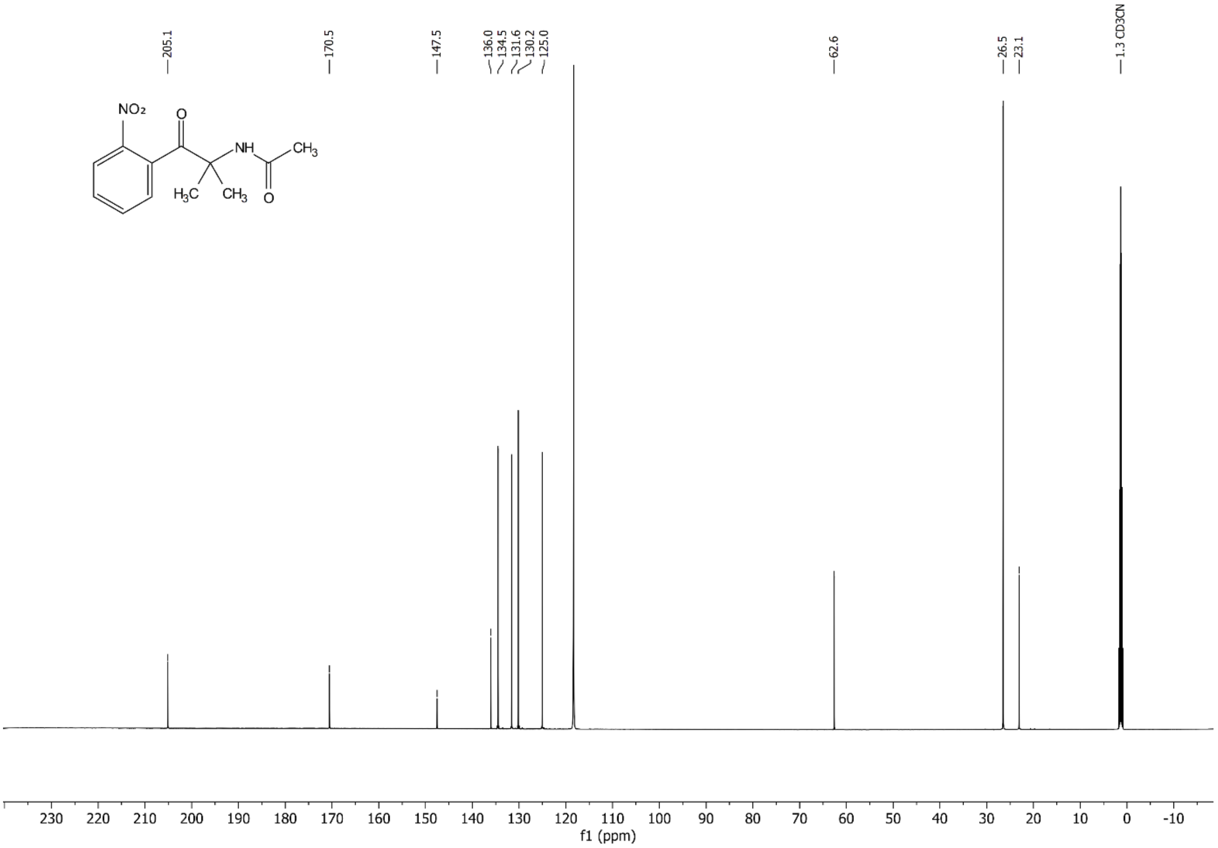

^{1}H NMR spectrum (400 MHz, CD_3CN) of **4af**.

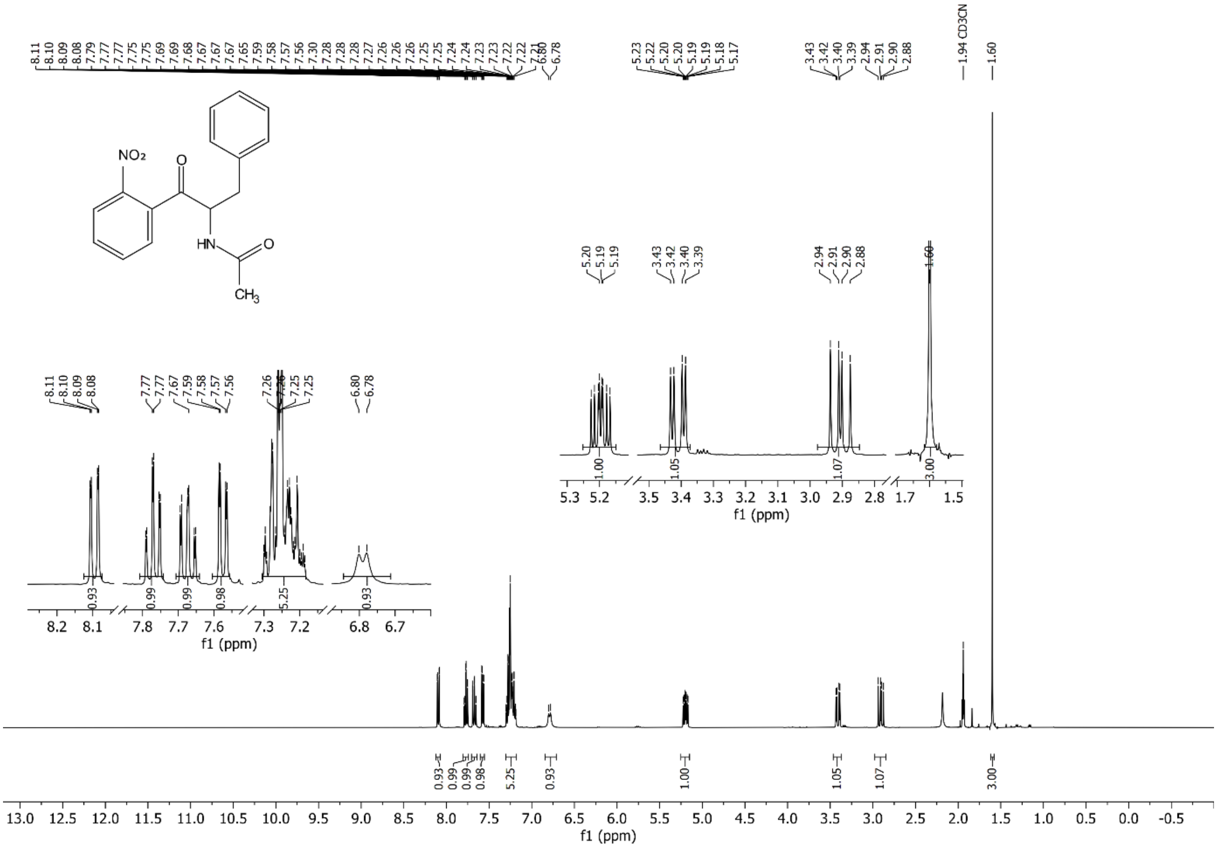

^{13}C NMR spectrum (101 MHz, CD_3CN) of **4af**.

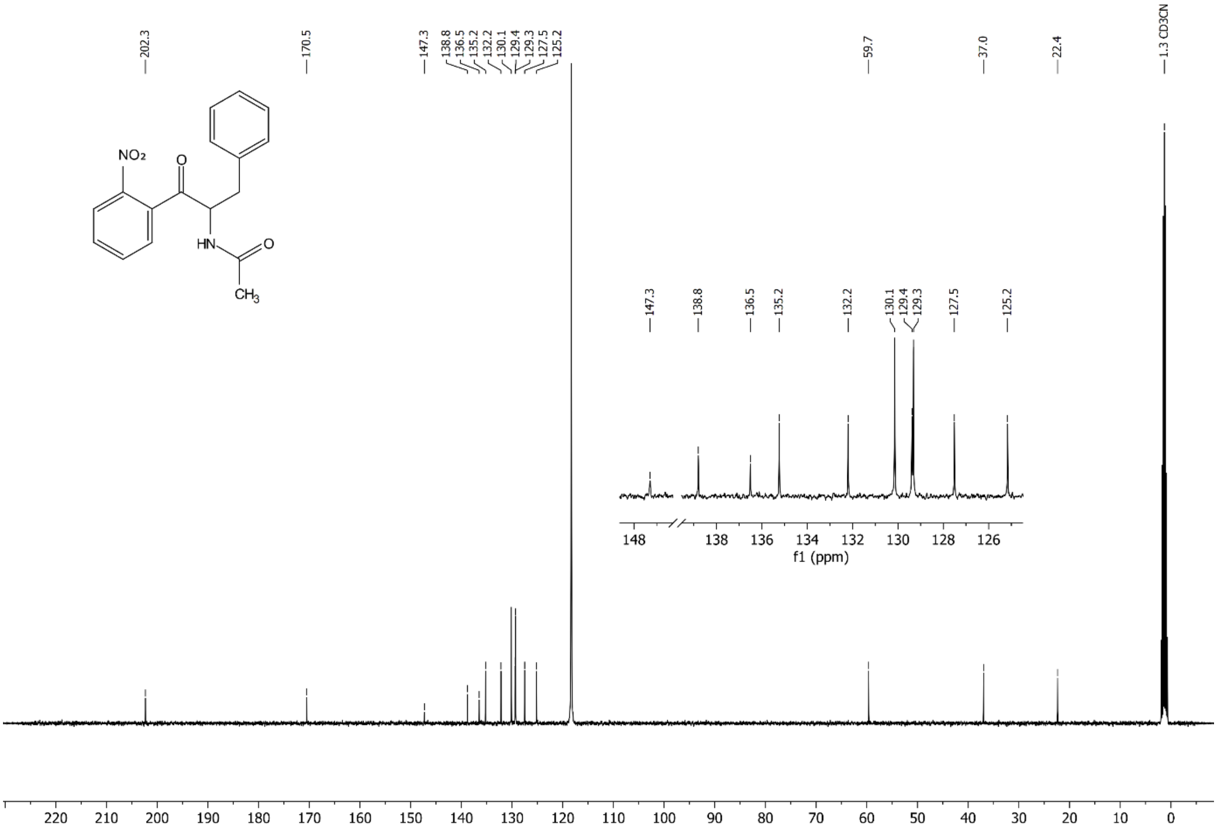

^{1}H NMR spectrum (400 MHz, CD_3OD) of **4ag**.

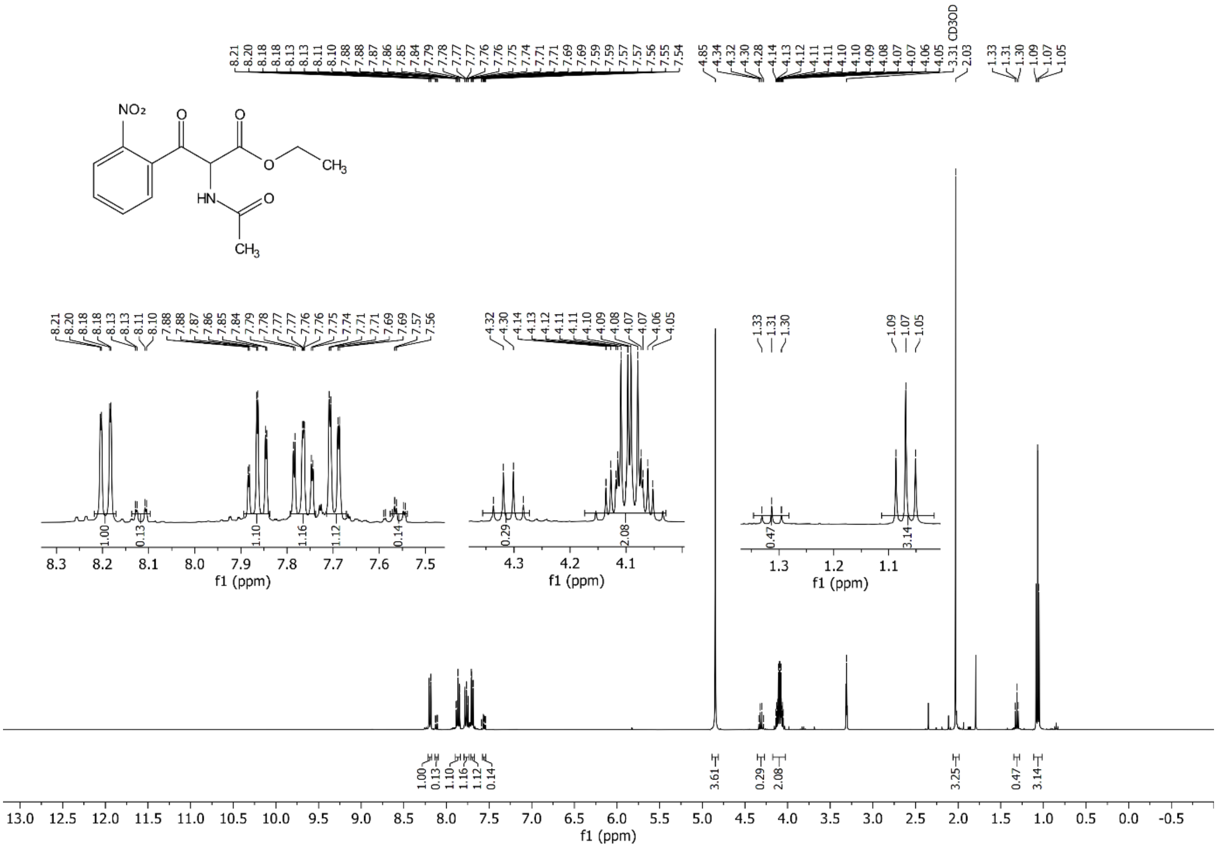

^{13}C NMR spectrum (101 MHz, CD_3OD) of **4ag**.

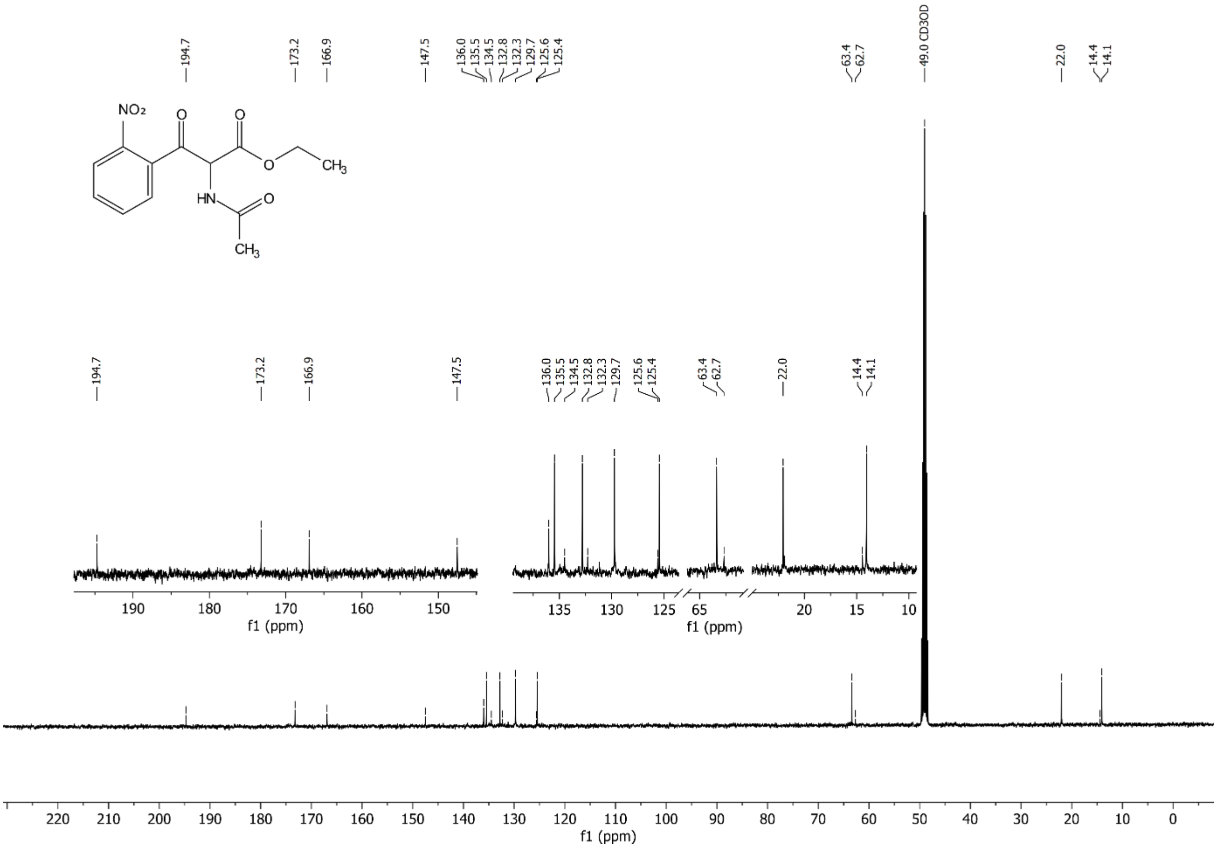

^{1}H NMR spectrum (400 MHz, CD_2Cl_2) of **4ah**.

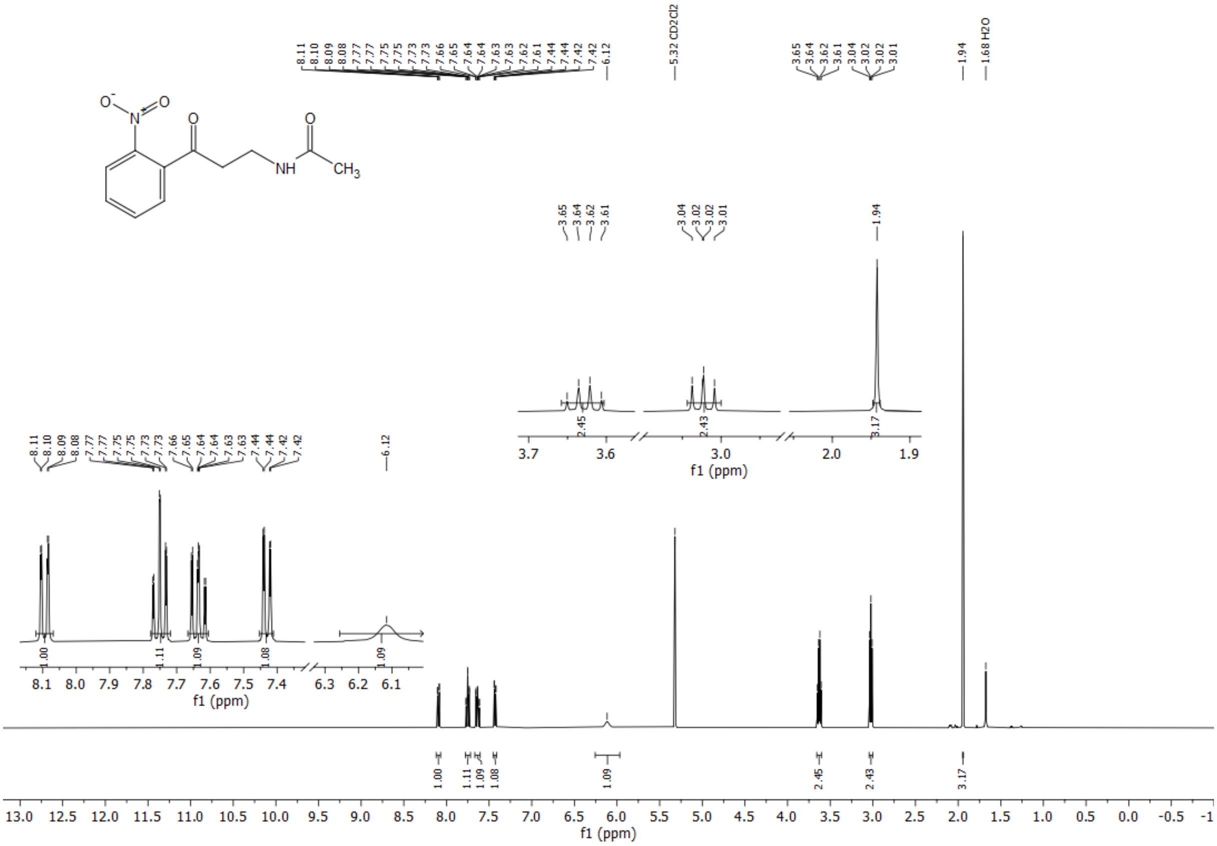

^{13}C NMR spectrum (101 MHz, CD_2Cl_2) of **4ah**.

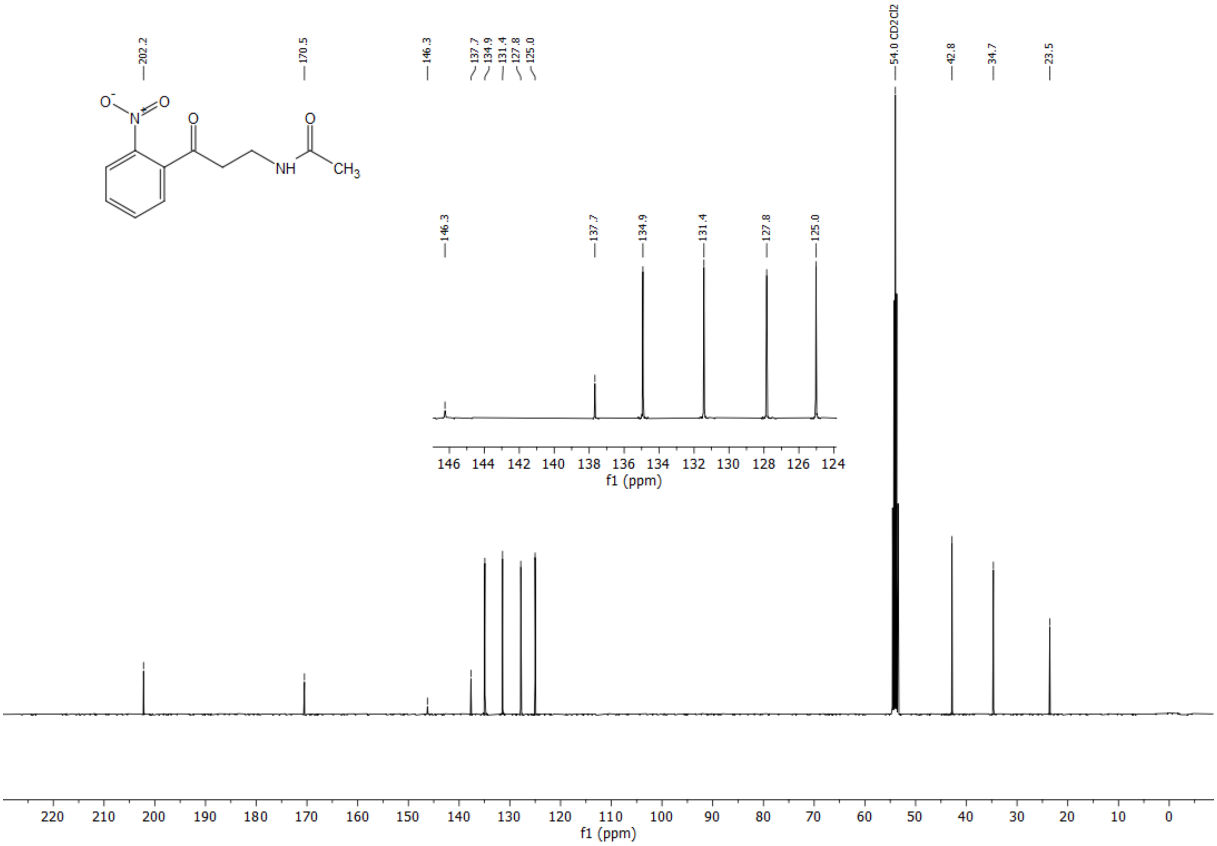

^{1}H NMR spectrum (400 MHz, DMSO-[D6]) of **6a**.

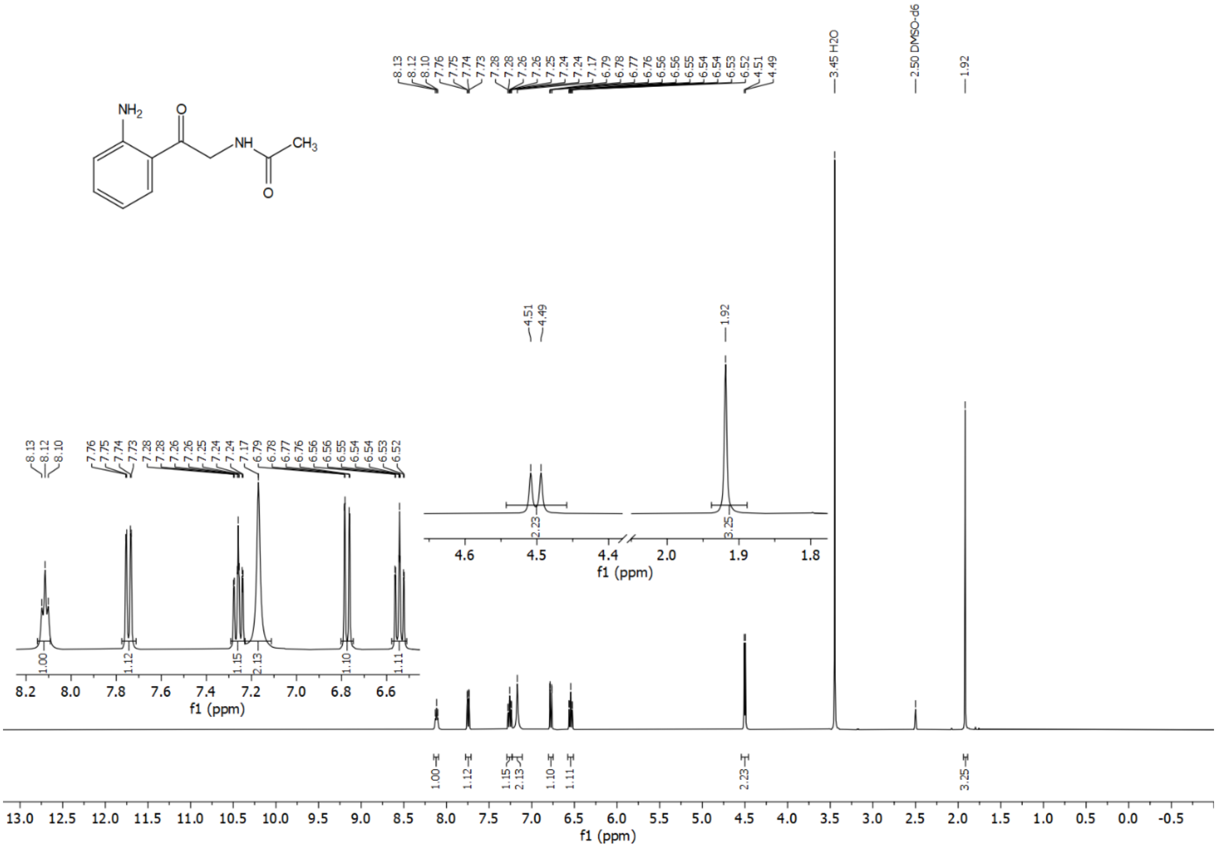

^{13}C NMR spectrum (101 MHz, DMSO-[D6]) of **6a.**

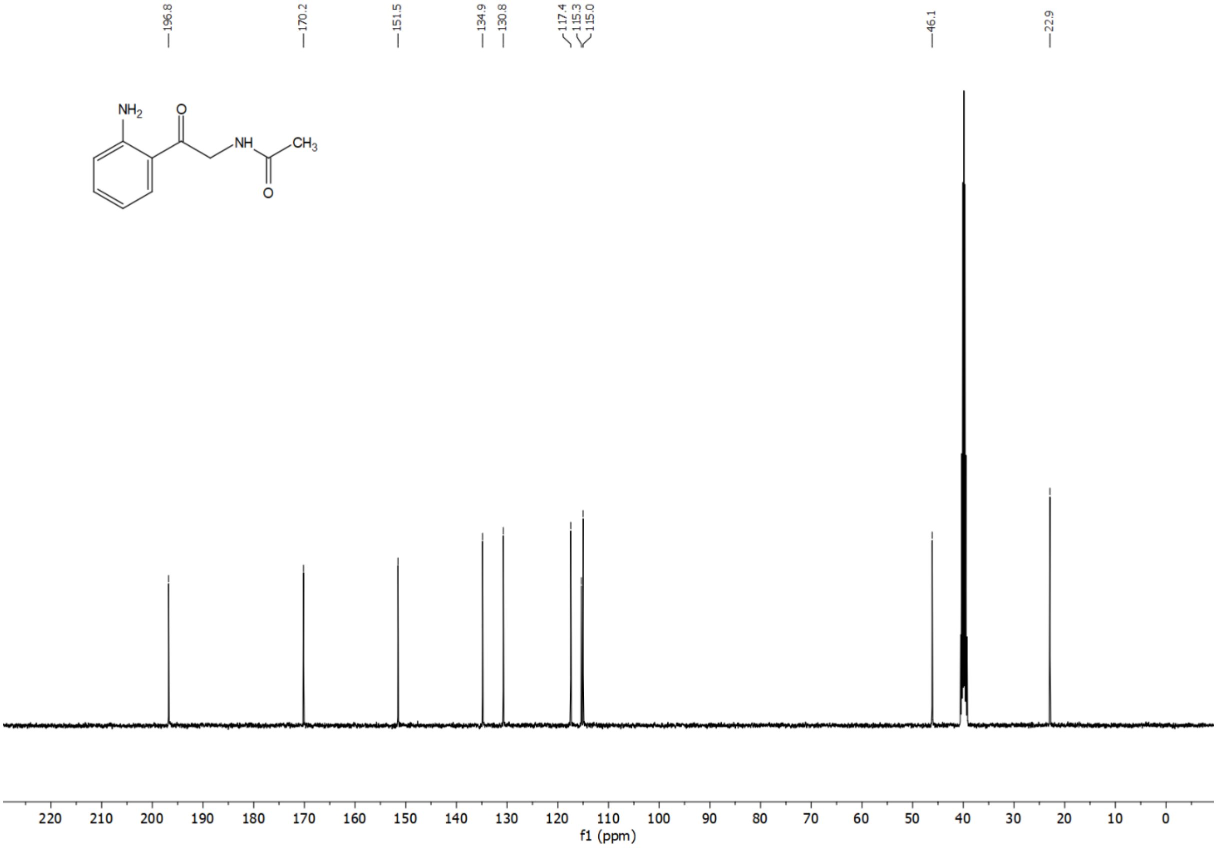

^{1}H NMR spectrum (400 MHz, $CDCl_3$) of **6a'**.

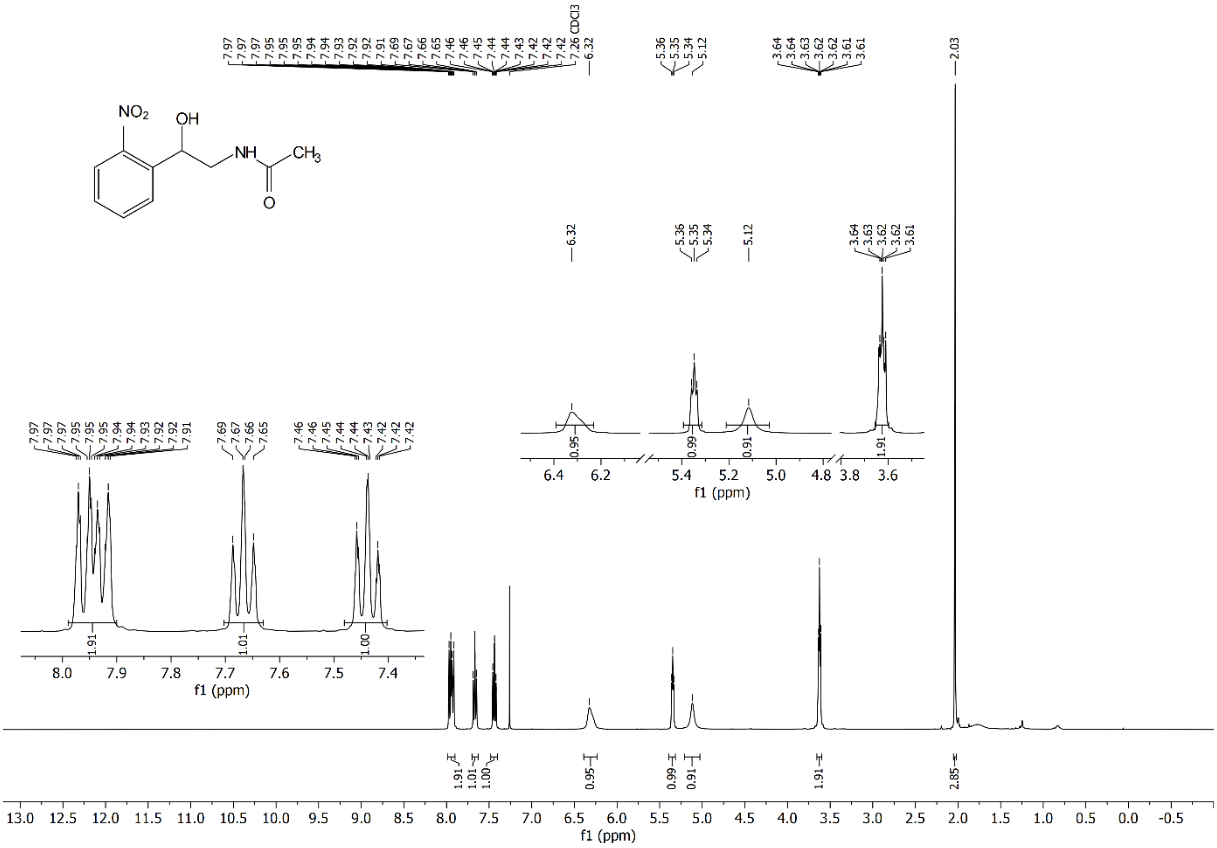

^{13}C NMR spectrum (101 MHz, $CDCl_3$) of **6a'**.

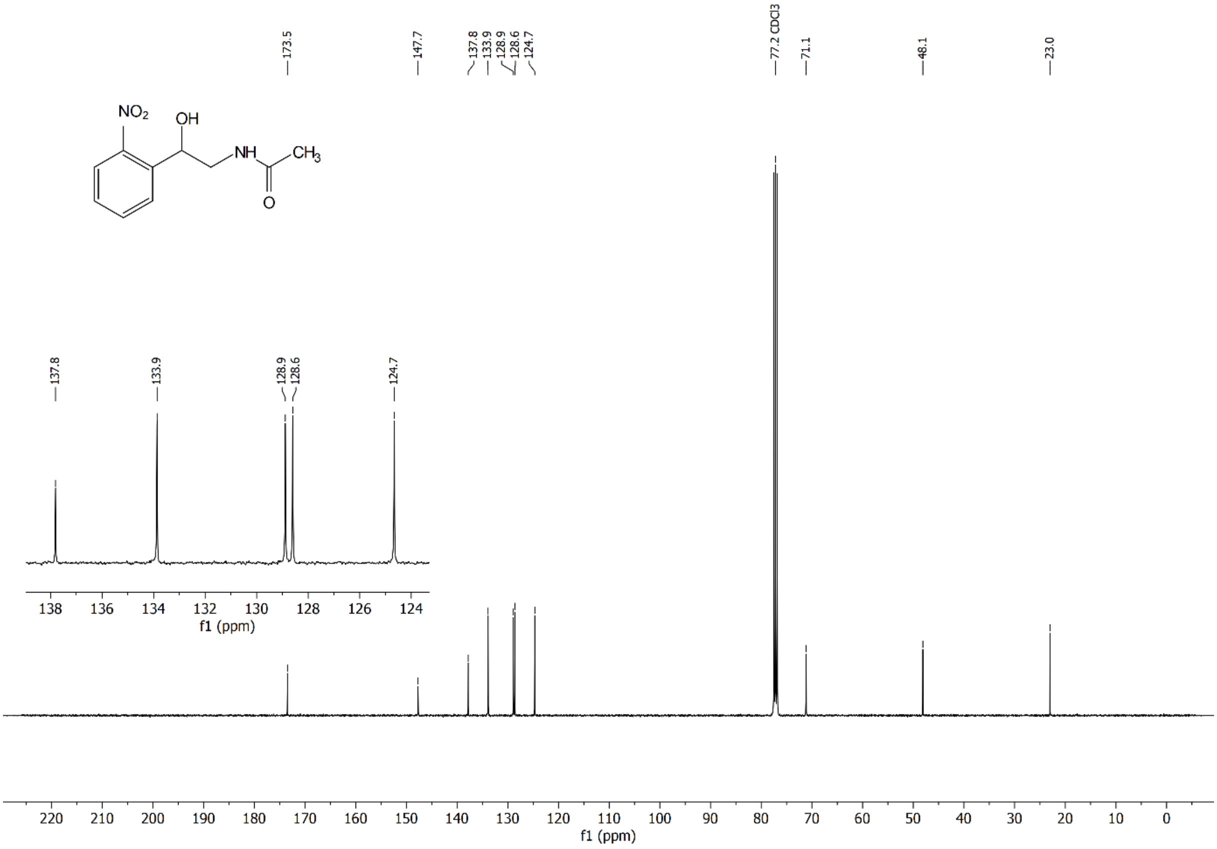

^{1}H NMR spectrum (400 MHz, DMSO-[D6]) of **5a**.

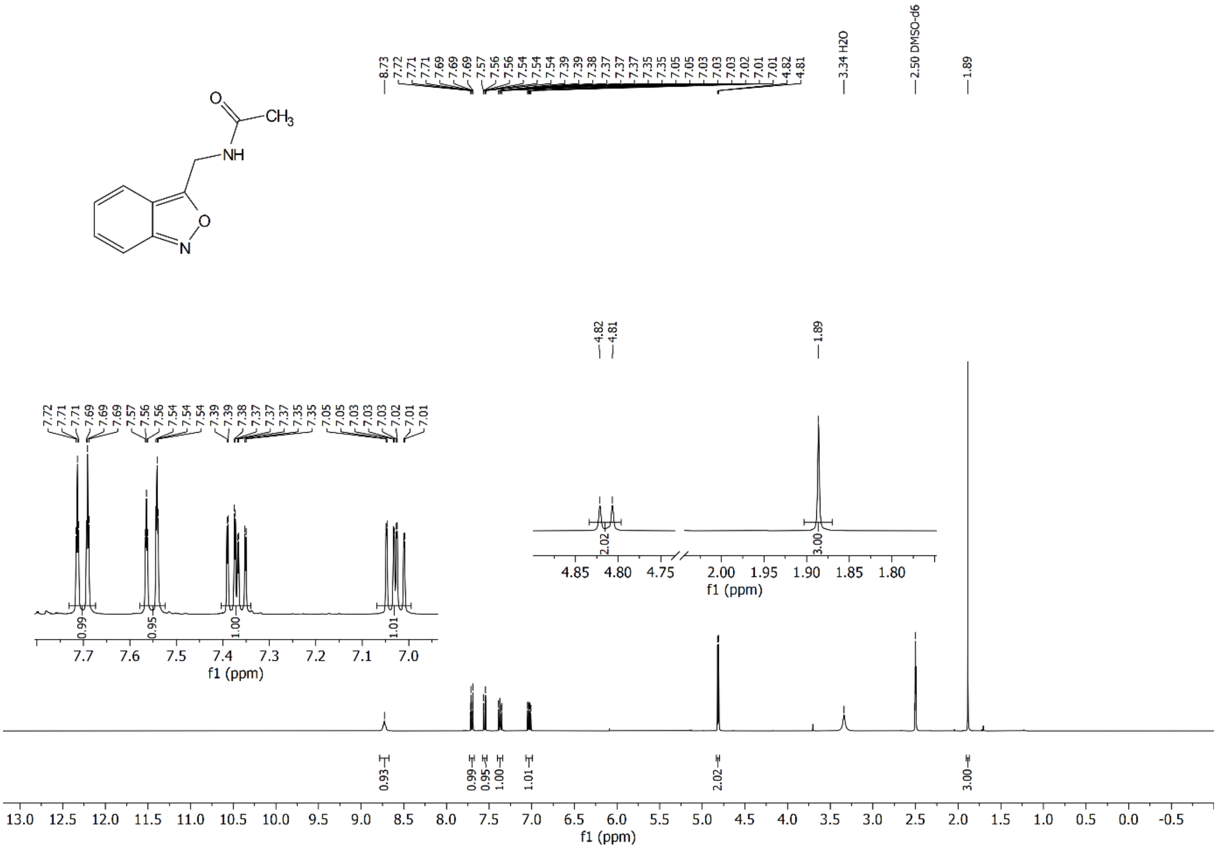

^{13}C NMR spectrum (101 MHz, DMSO-[D6]) of **5a**.

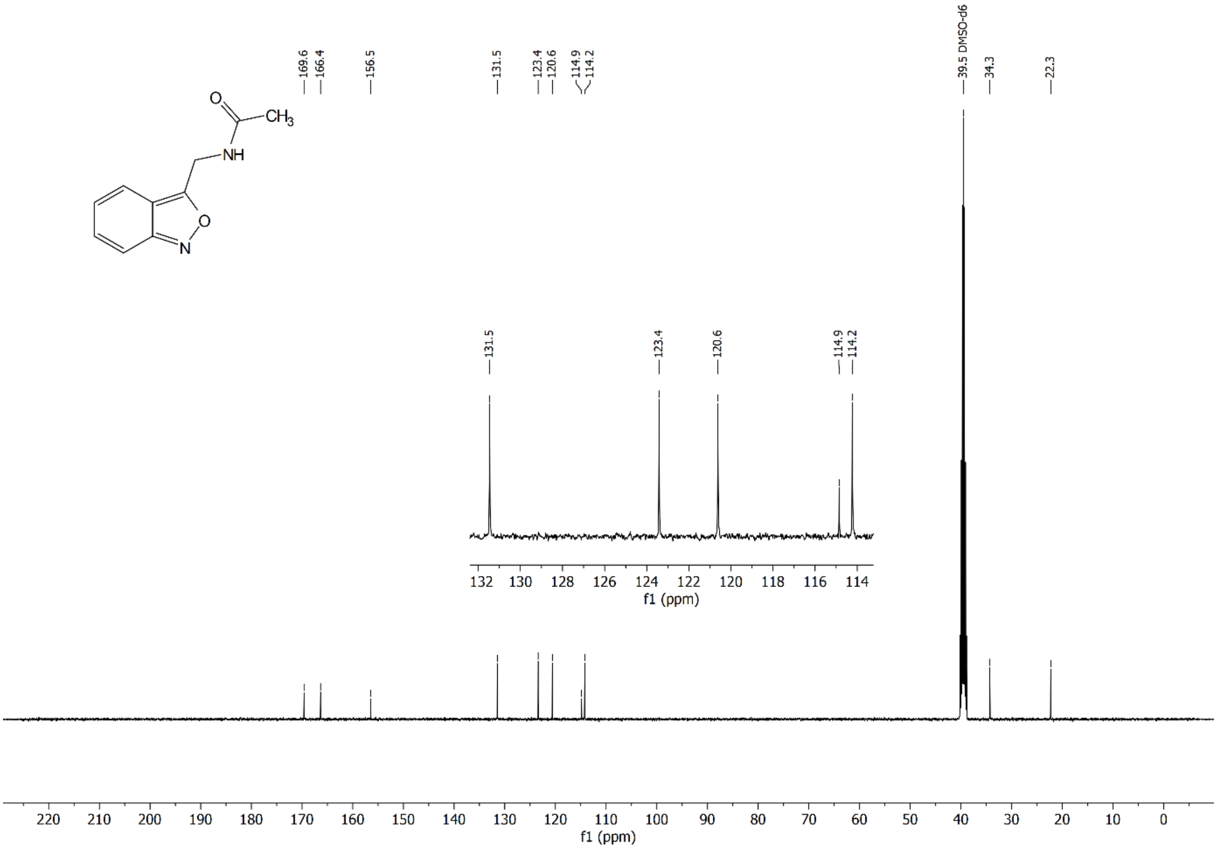

^{1}H NMR spectrum (400 MHz, $CDCl_3$) of **5b**.

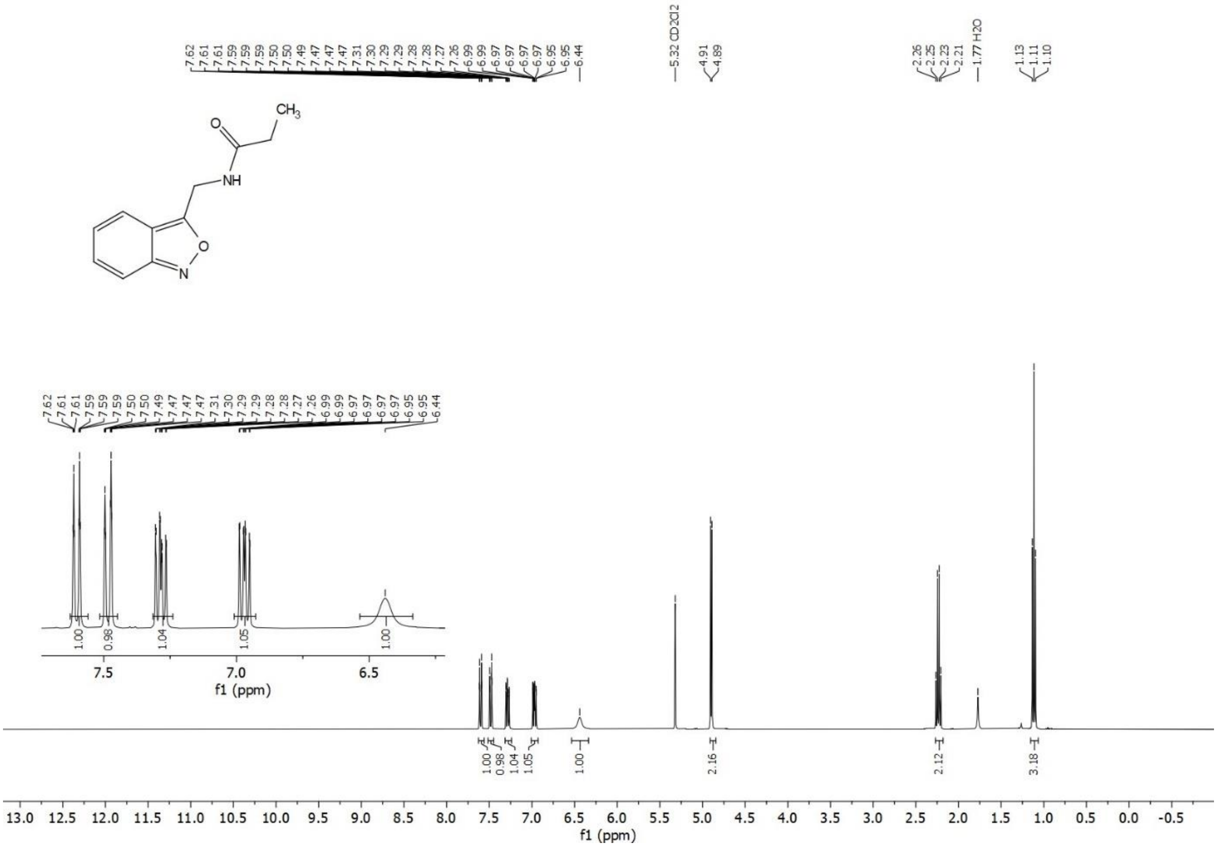

^{13}C NMR spectrum (101 MHz, CD_2Cl_2) of **5b**.

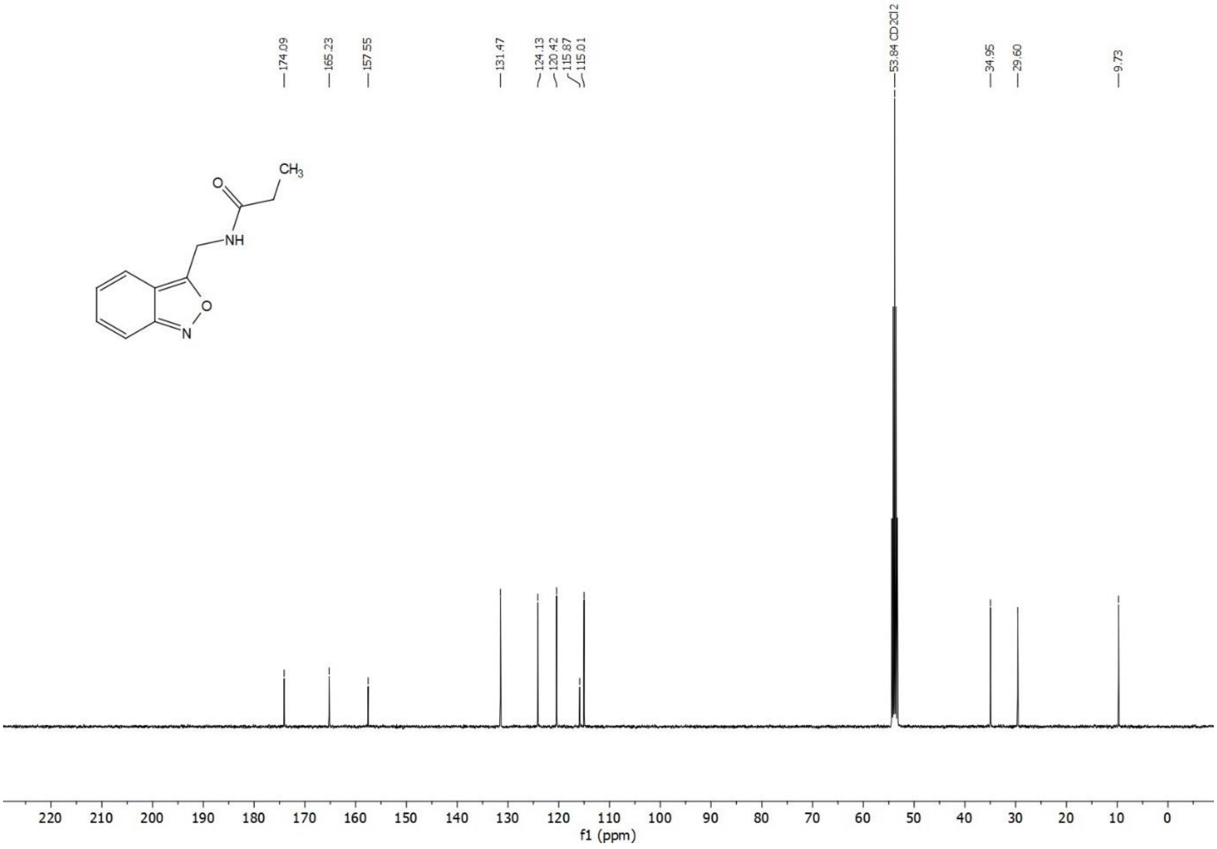

^{1}H NMR spectrum (400 MHz, CD_2Cl_2) of **5c**.

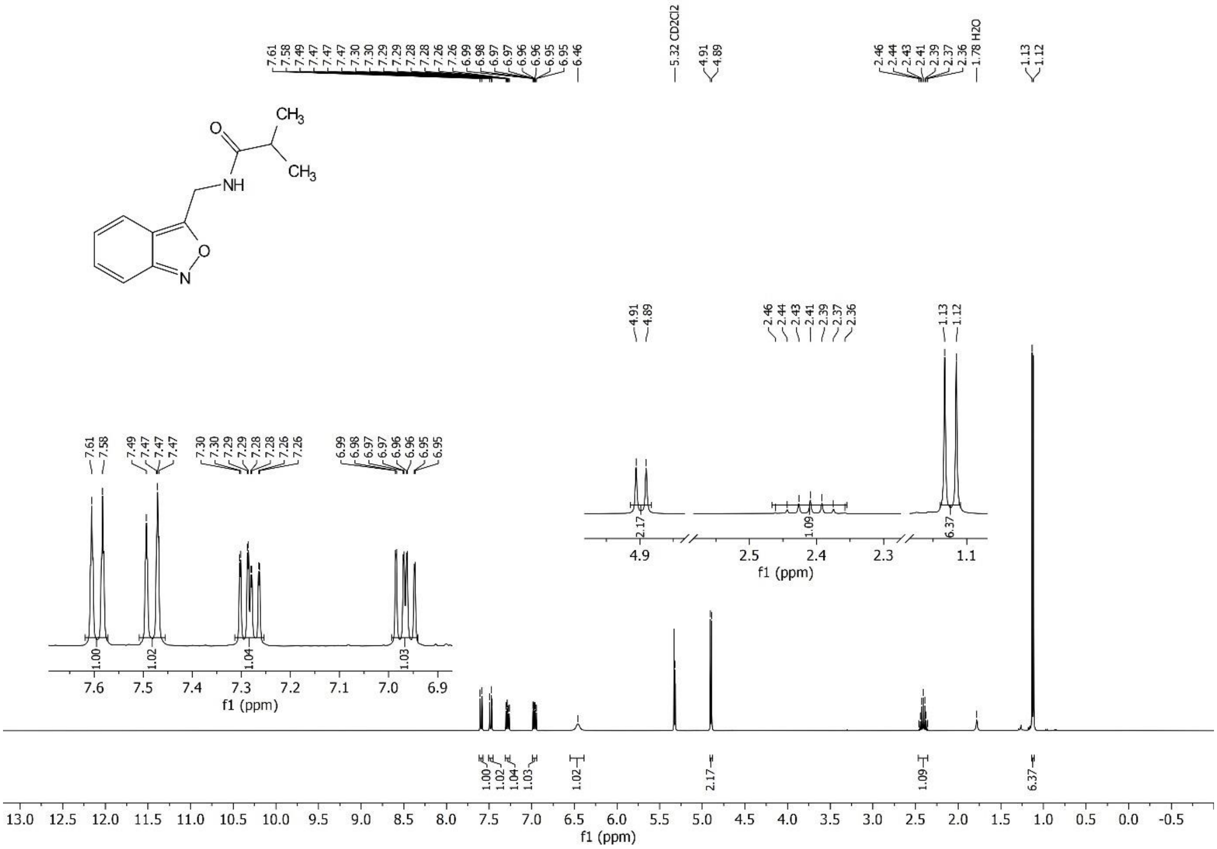

^{13}C NMR spectrum (101 MHz, CD_2Cl_2) of **5c**.

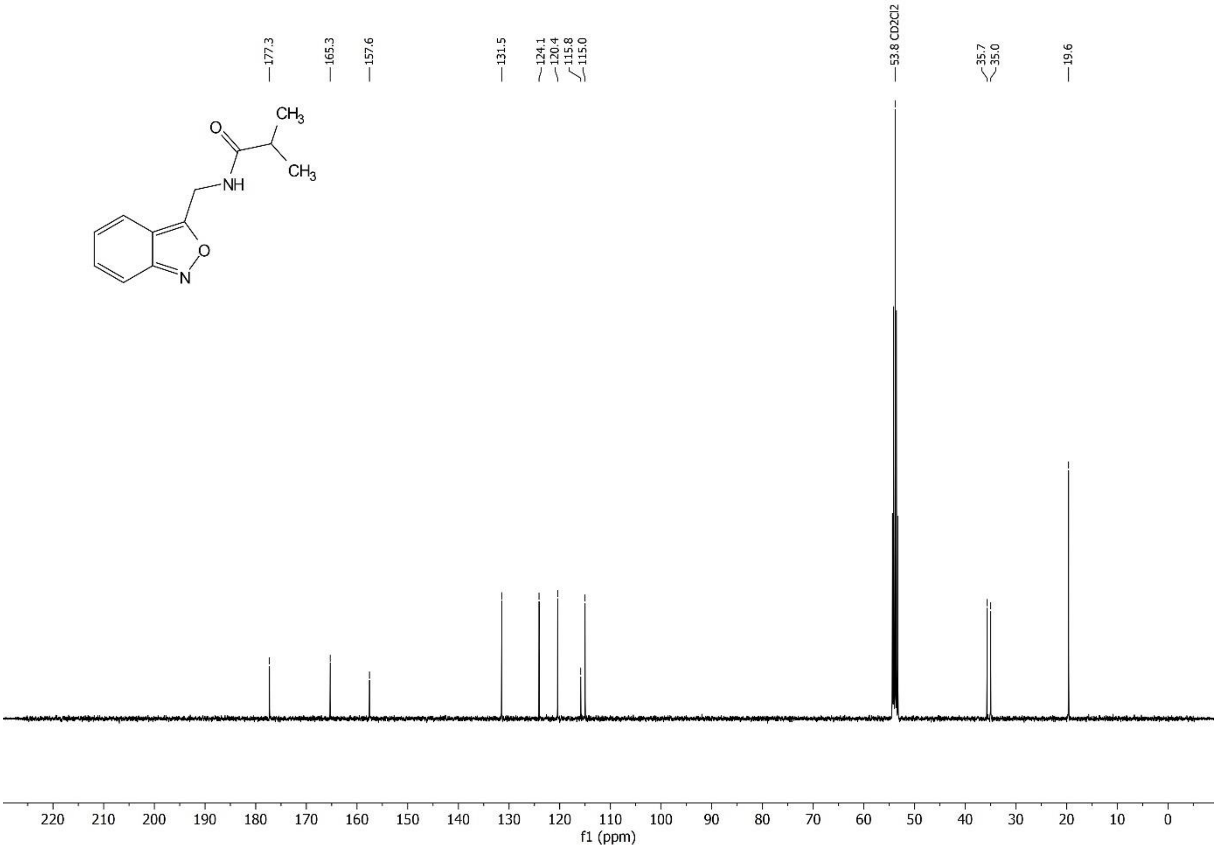

^{1}H NMR spectrum (400 MHz, CD_2Cl_2) of **5d**.

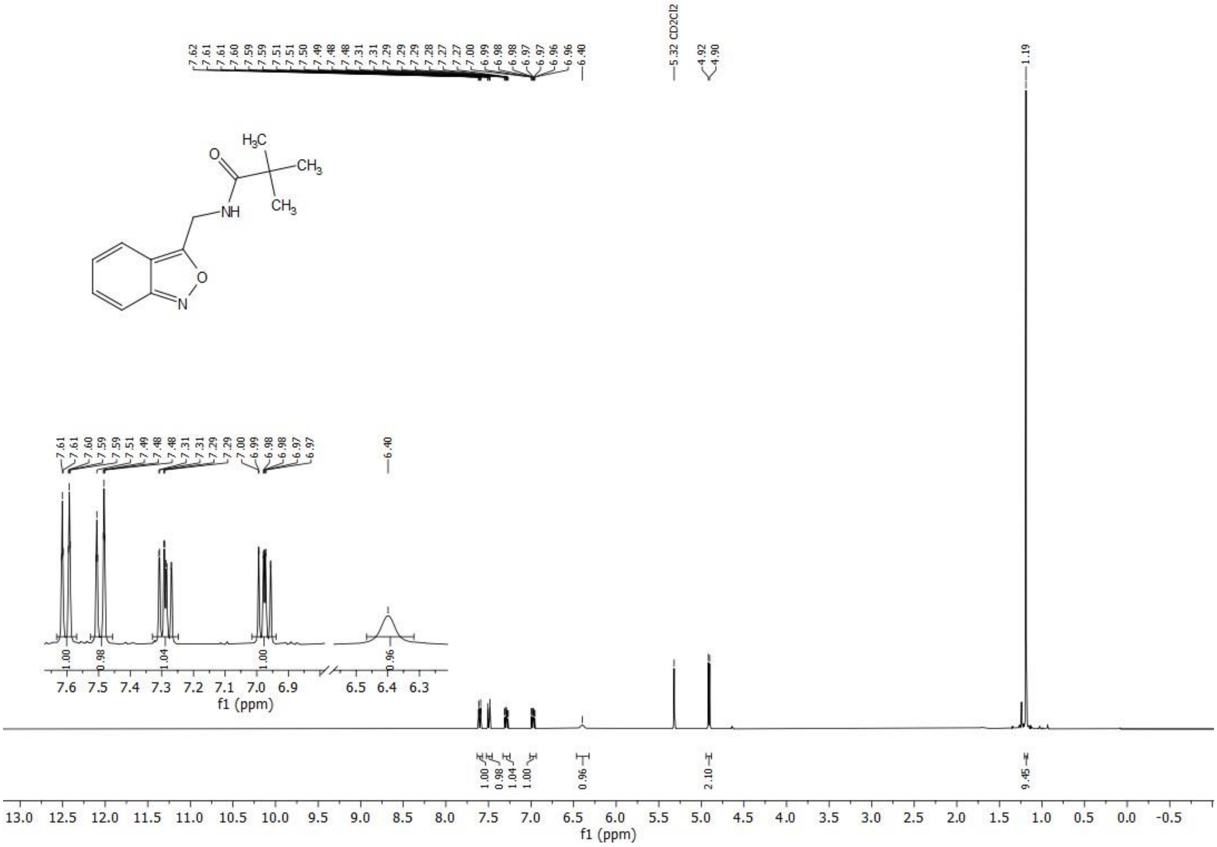

^{13}C NMR spectrum (101 MHz, CD_2Cl_2) of **5d**.

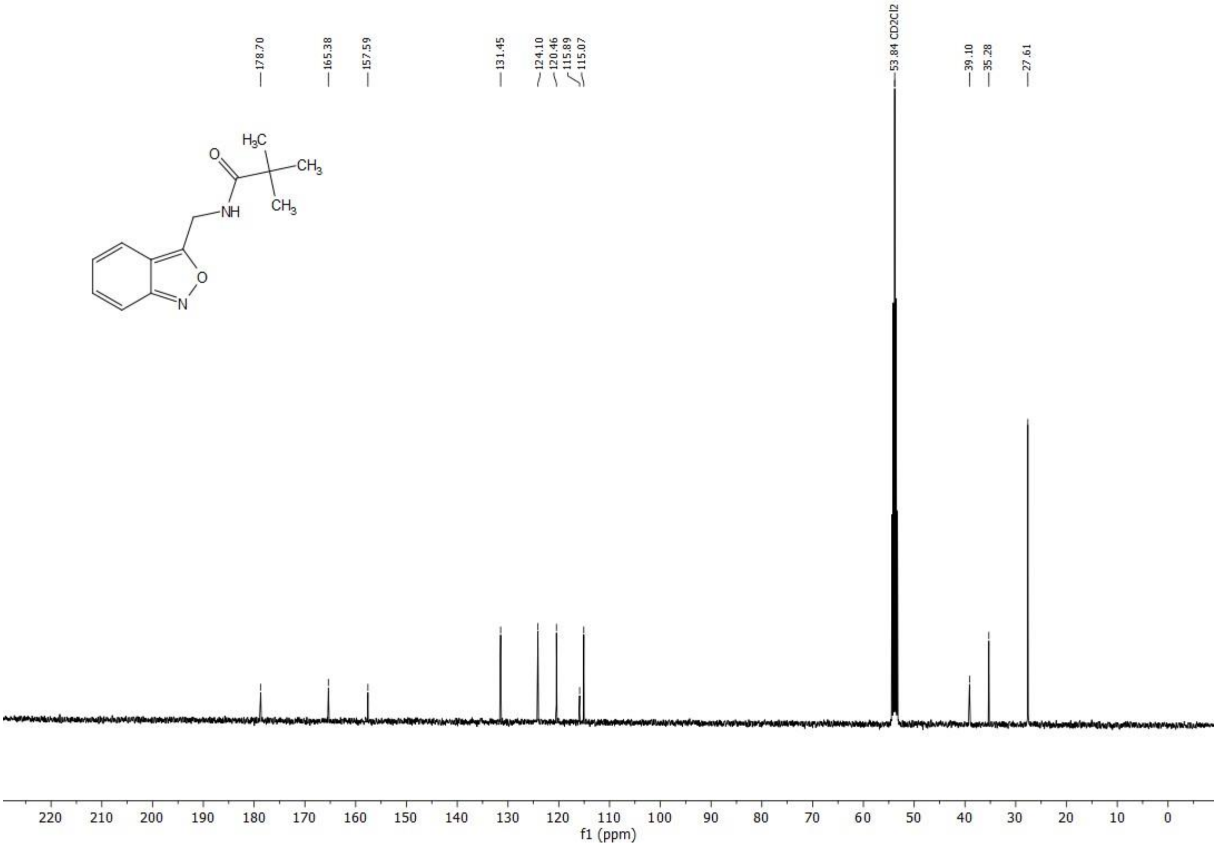

^{1}H NMR spectrum (400 MHz, CD_2Cl_2) of **5e**.

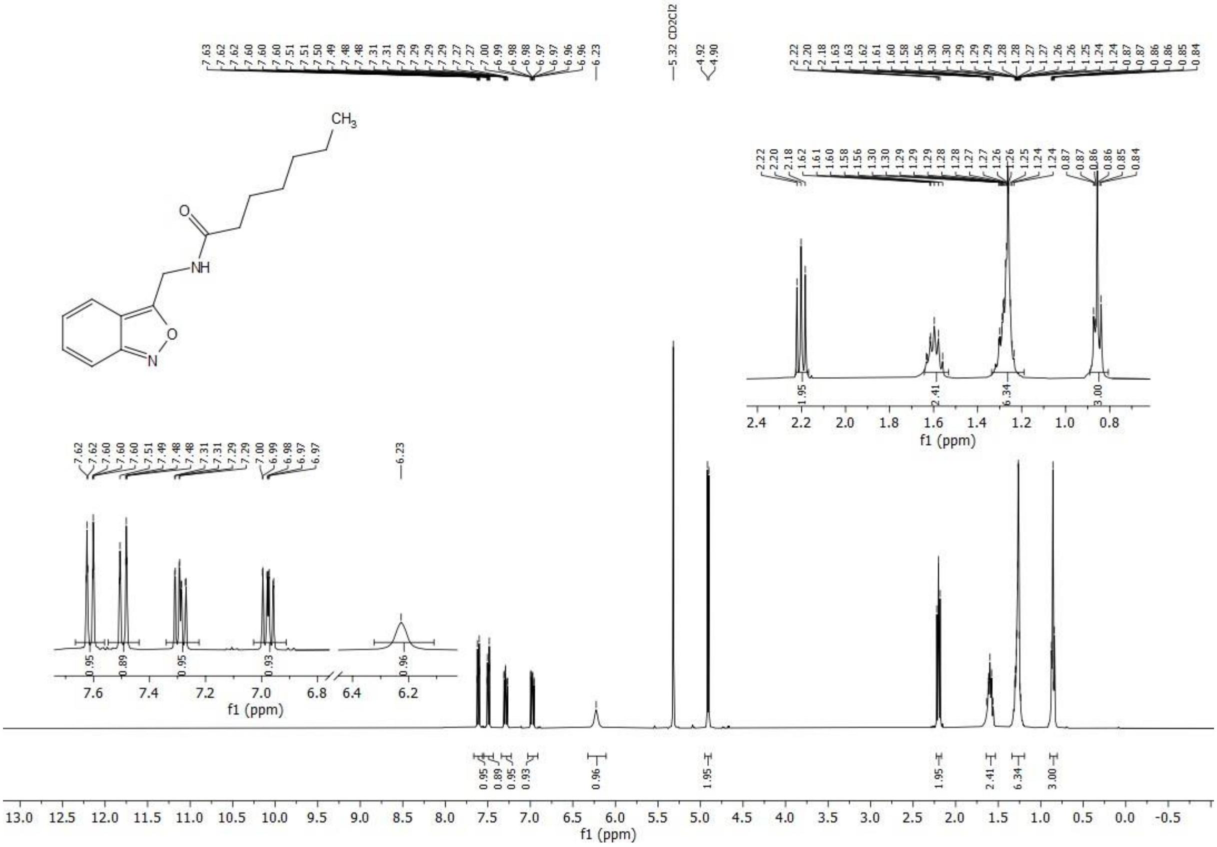

^{13}C NMR spectrum (101 MHz, CD_2Cl_2) of **5e**.

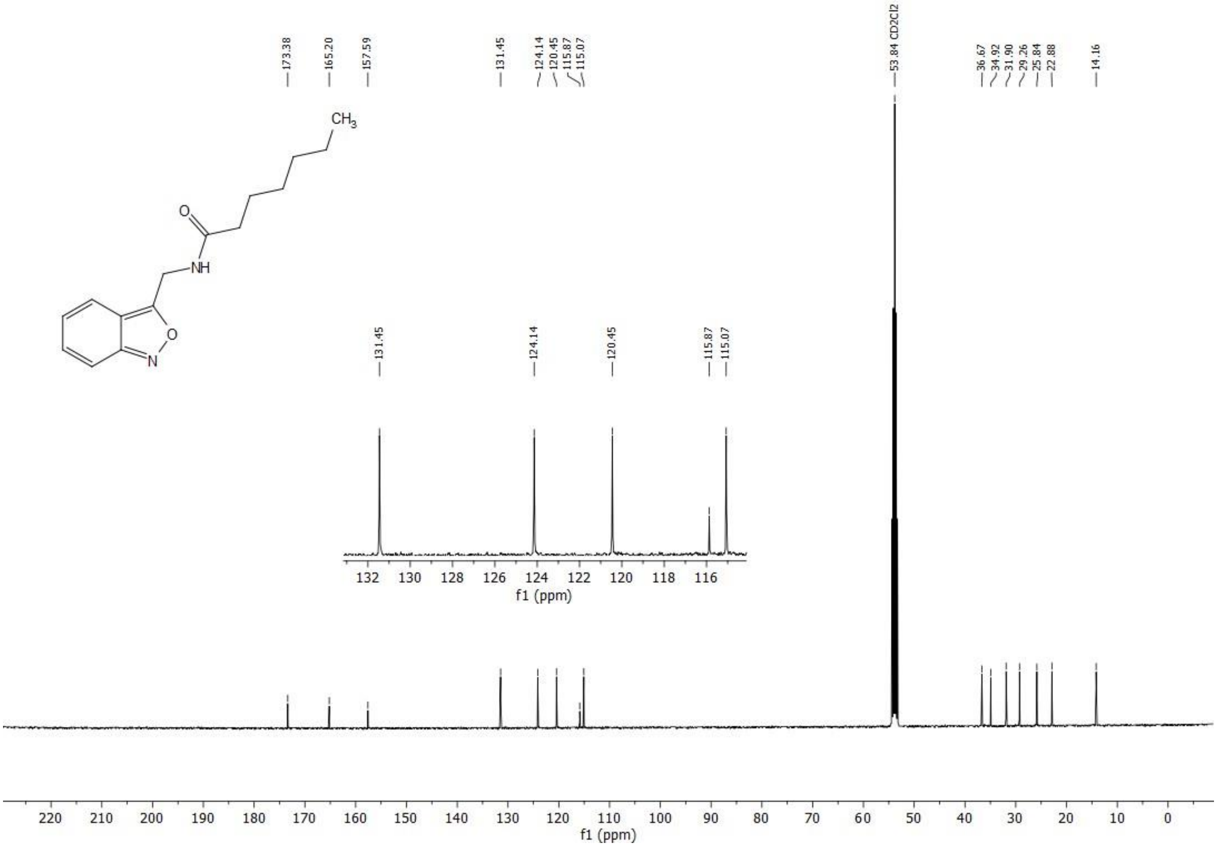

^{1}H NMR spectrum (400 MHz, CD_2Cl_2) of **5f**.

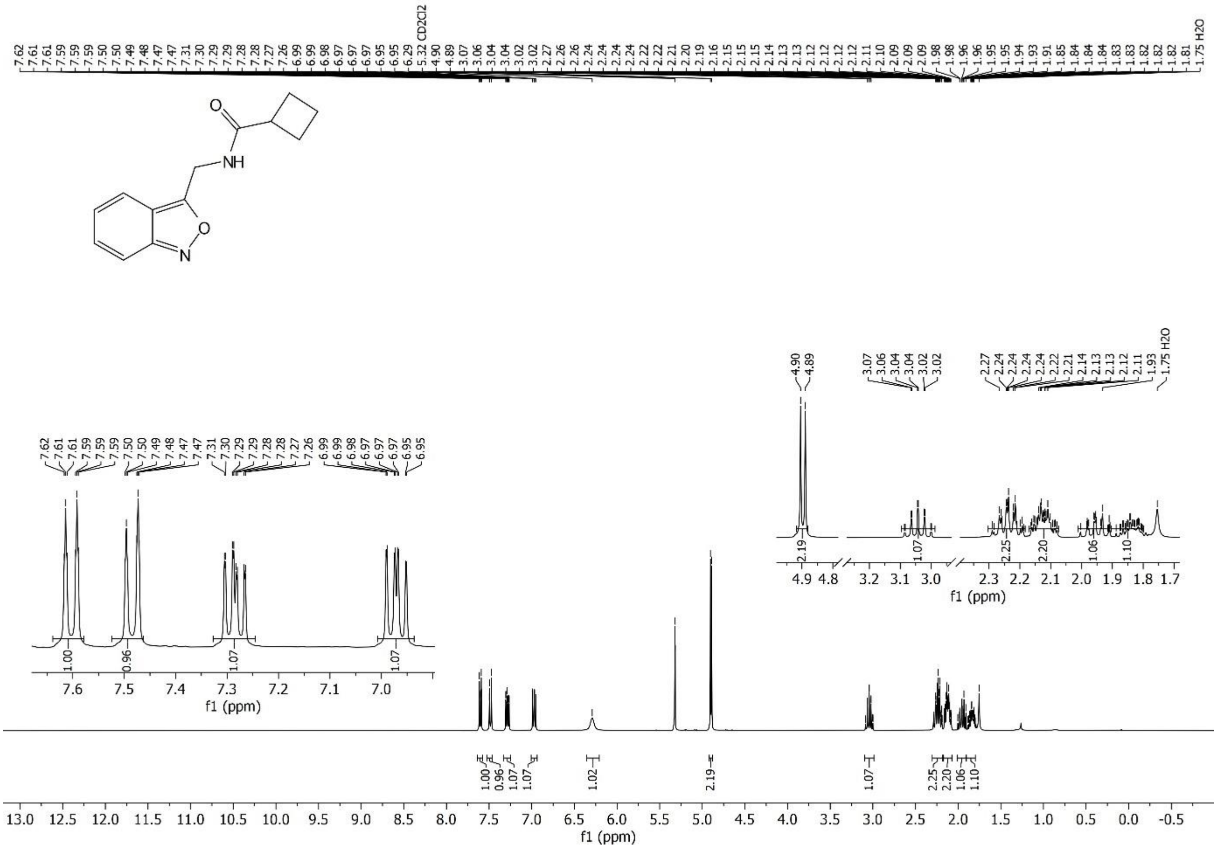

^{13}C NMR spectrum (101 MHz, CD_2Cl_2) of **5f**.

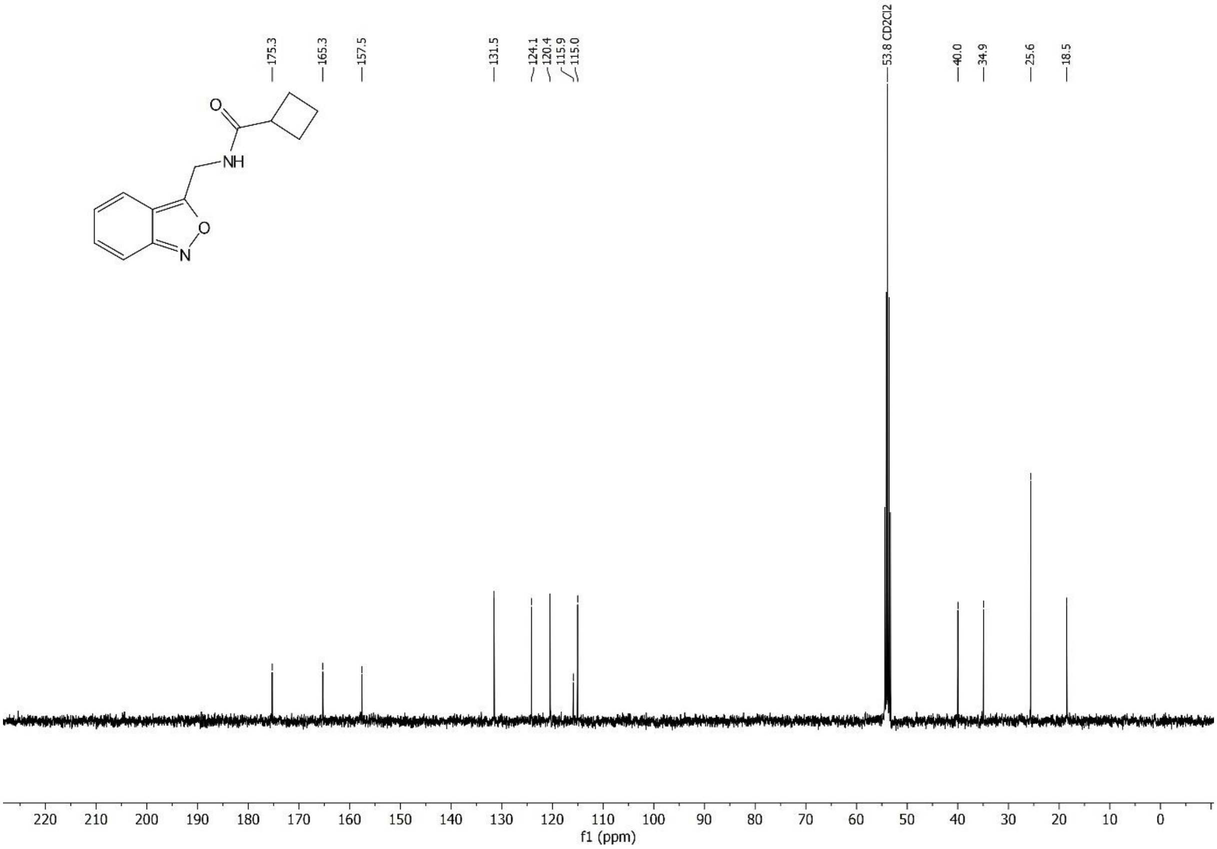

^{1}H NMR spectrum (400 MHz, CD_3CN) of **5g**.

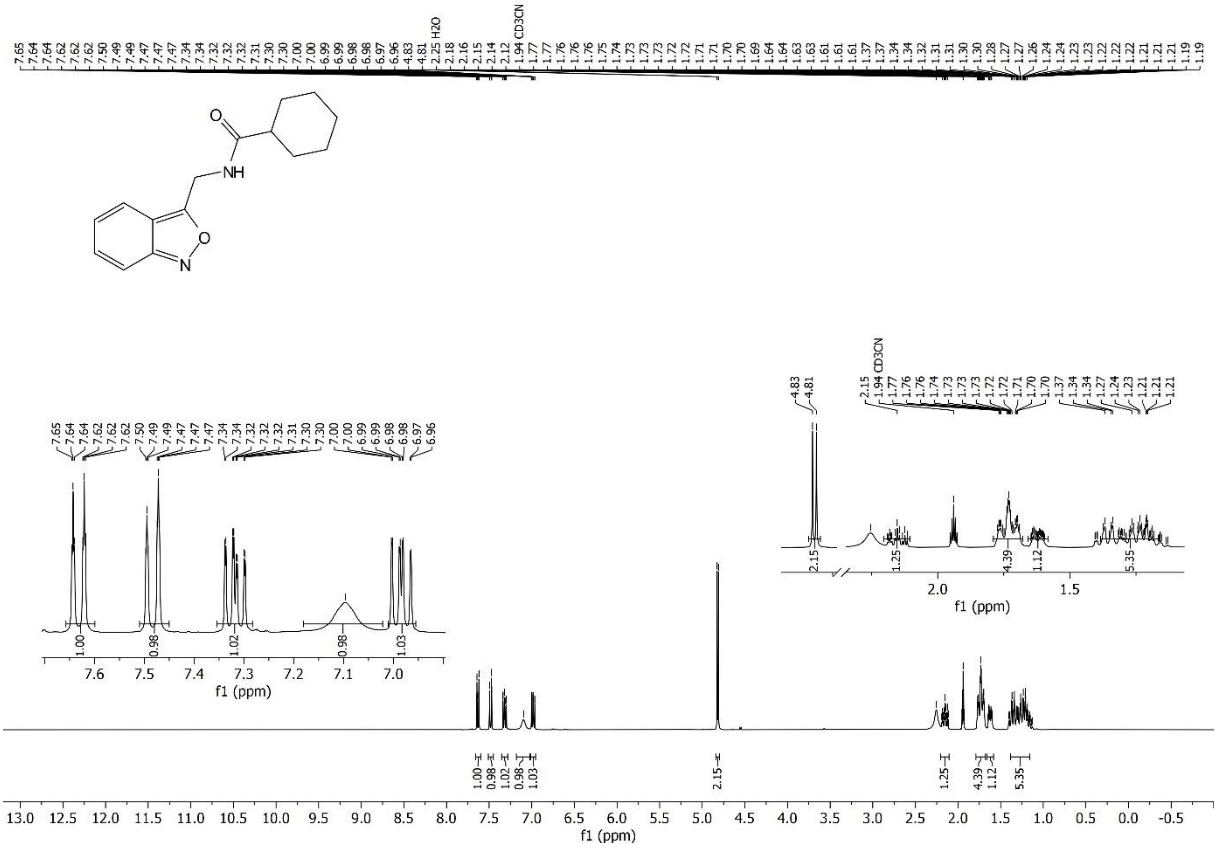

^{13}C NMR spectrum (101 MHz, CD_3CN) of **5g**.

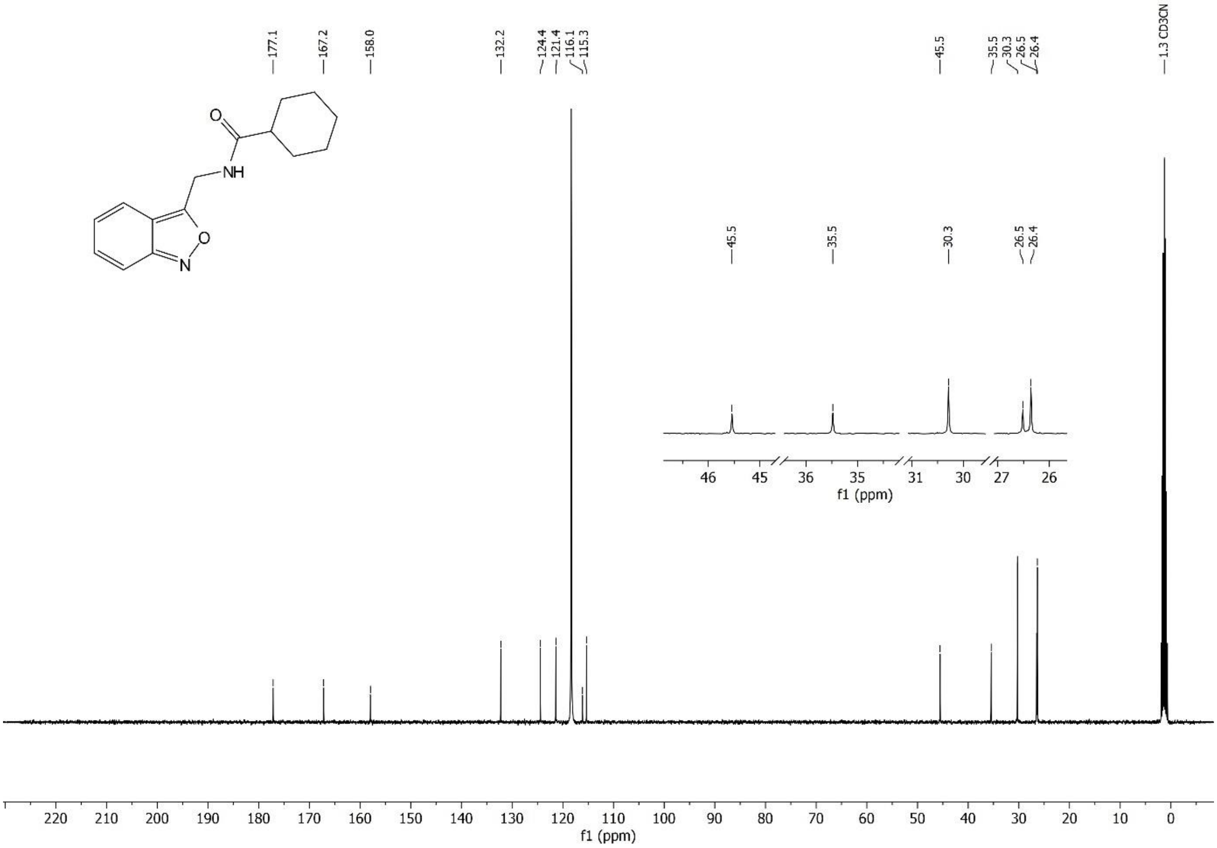

^{1}H NMR spectrum (400 MHz, CD_3CN) of **5h**.

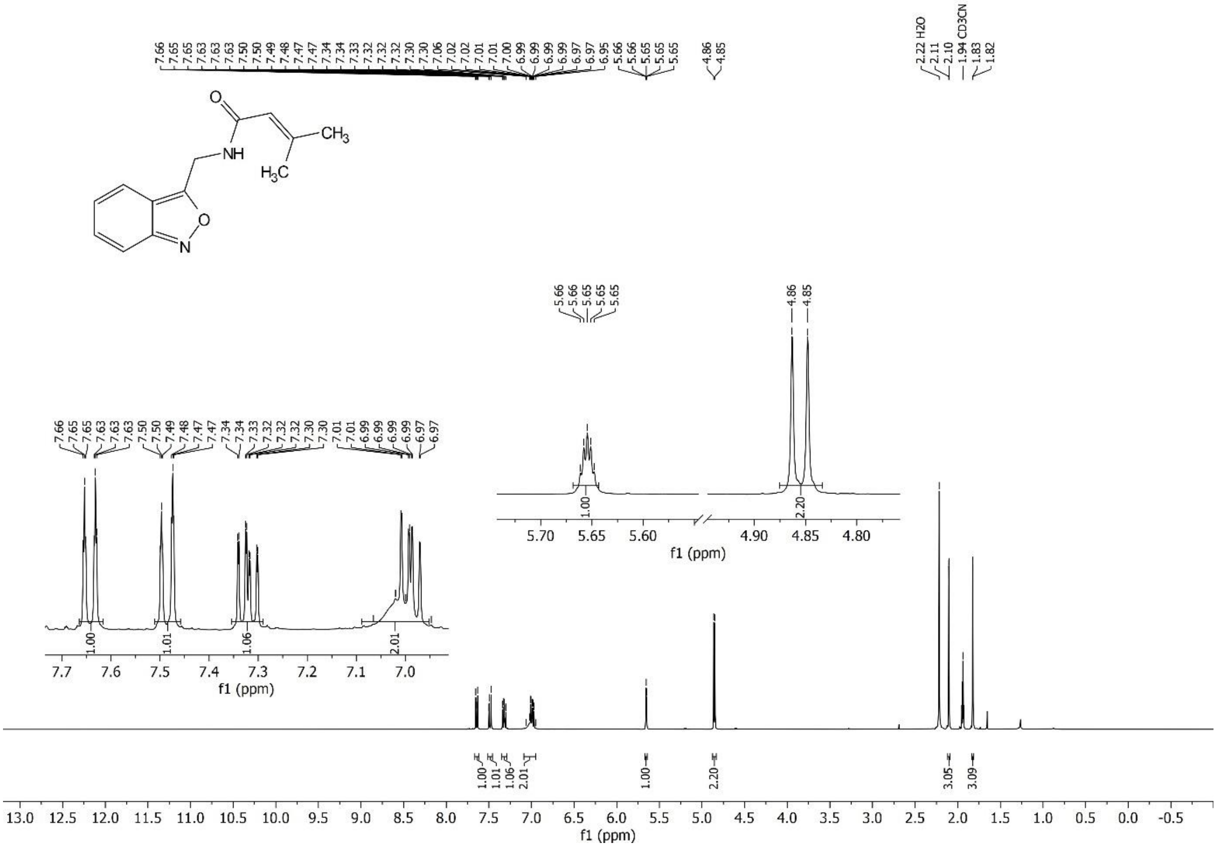

^{13}C NMR spectrum (101 MHz, CD_3CN) of **5h**.

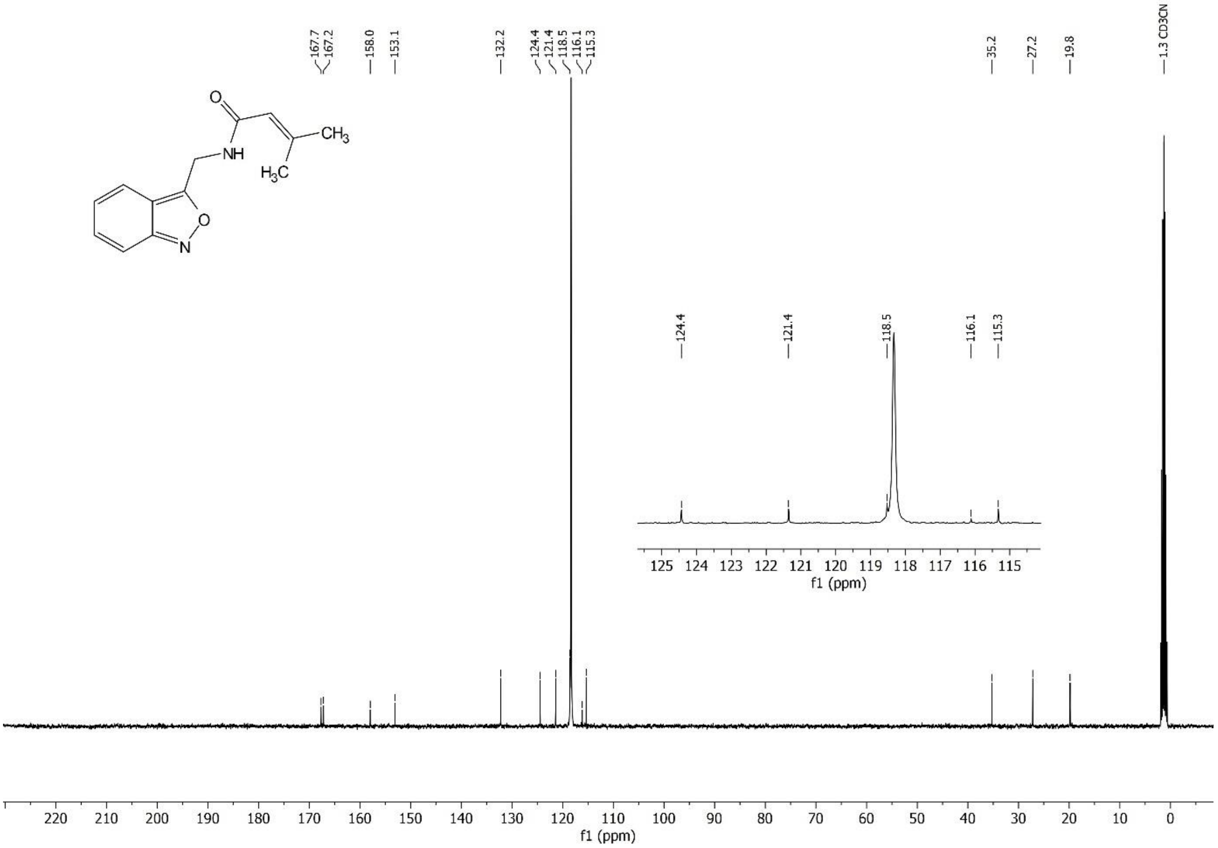

^1H NMR spectrum (400 MHz, CD_2Cl_2) of **5i**.

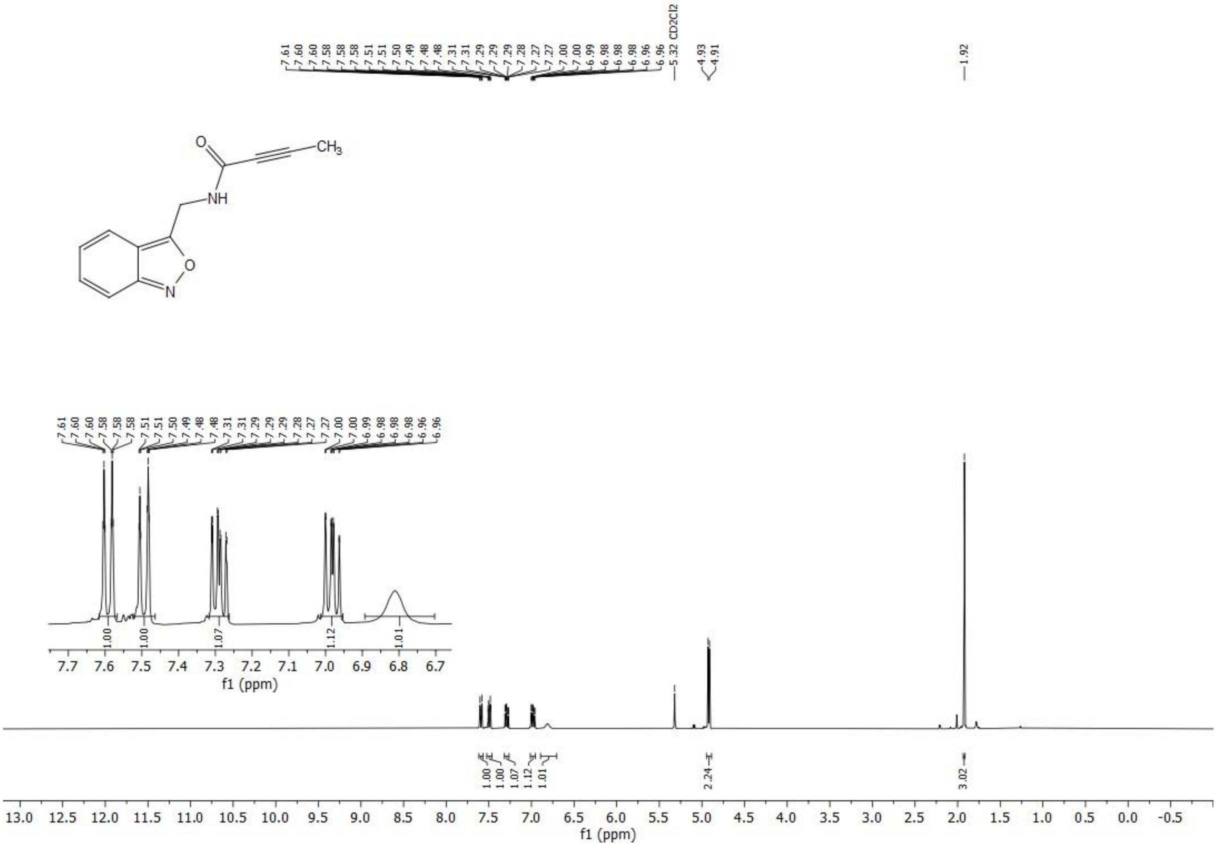

^{13}C NMR spectrum (101 MHz, CD_2Cl_2) of **5i**.

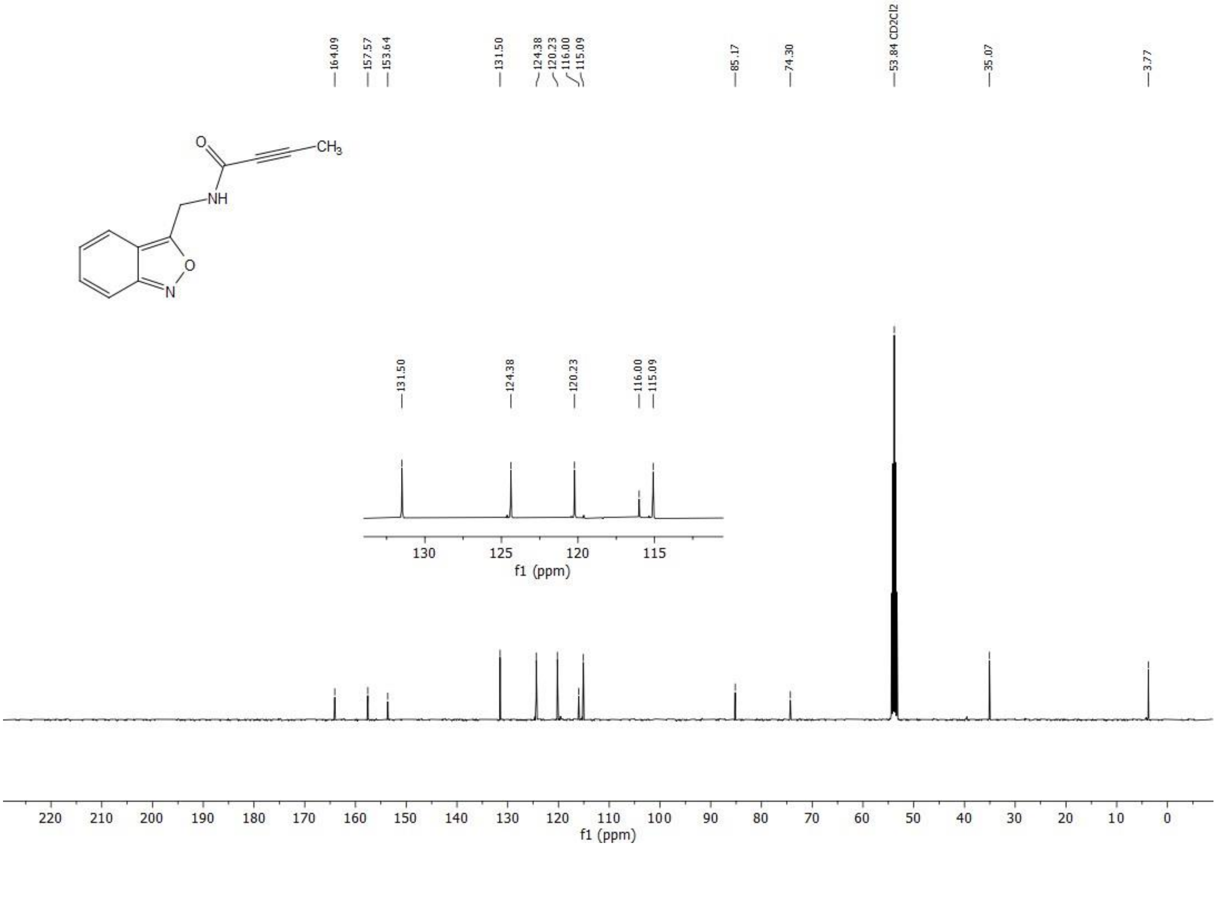

^{1}H NMR spectrum (400 MHz, CD_2Cl_2) of **5j**.

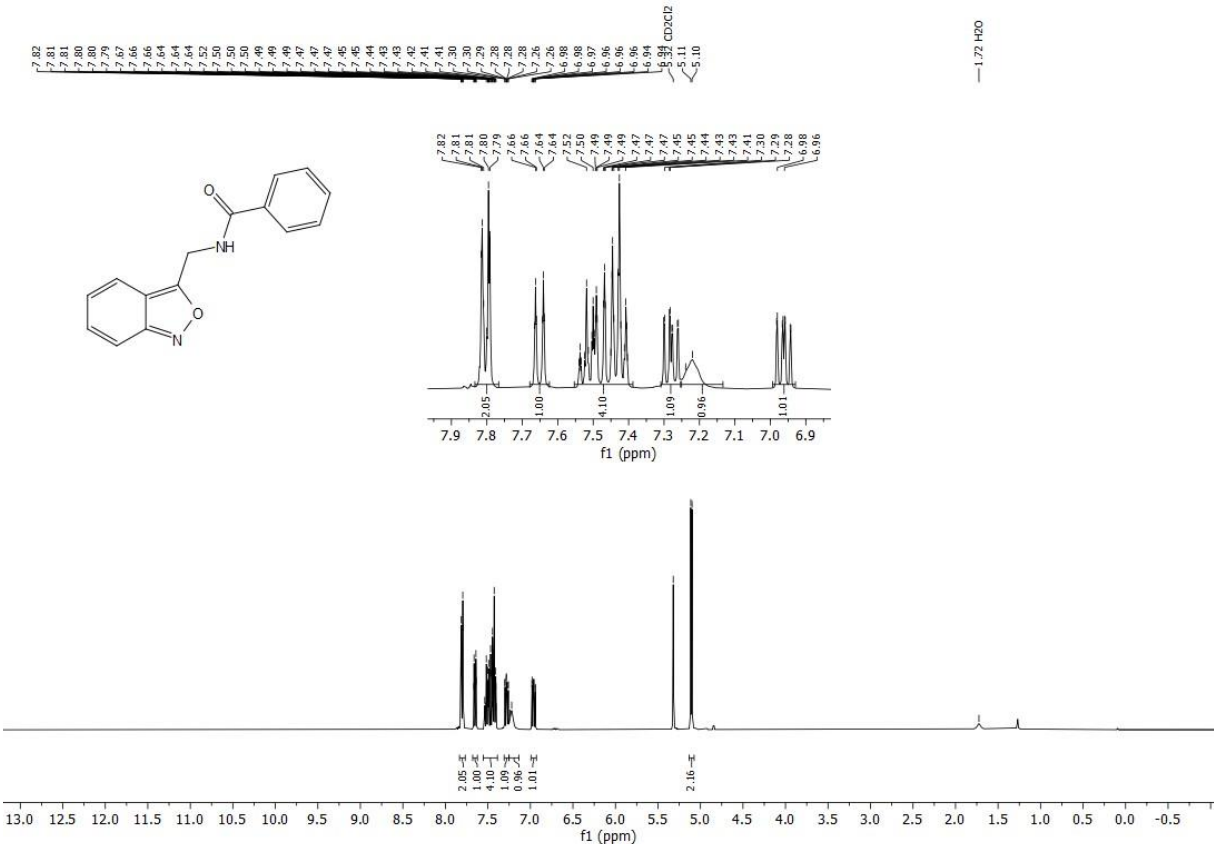

^{13}C NMR spectrum (101 MHz, CD_2Cl_2) of **5j**.

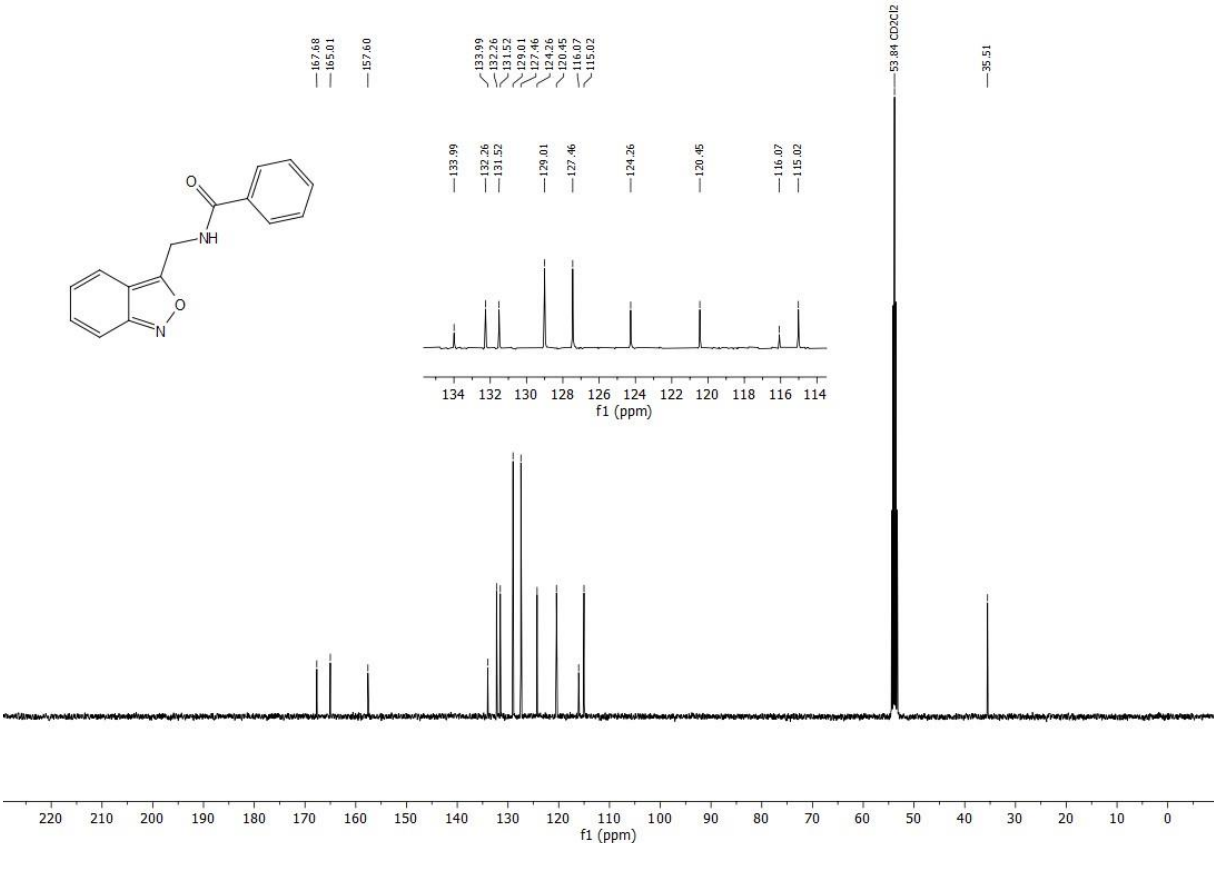

^{1}H NMR spectrum (400 MHz, CD_2Cl_2) of **5k**.

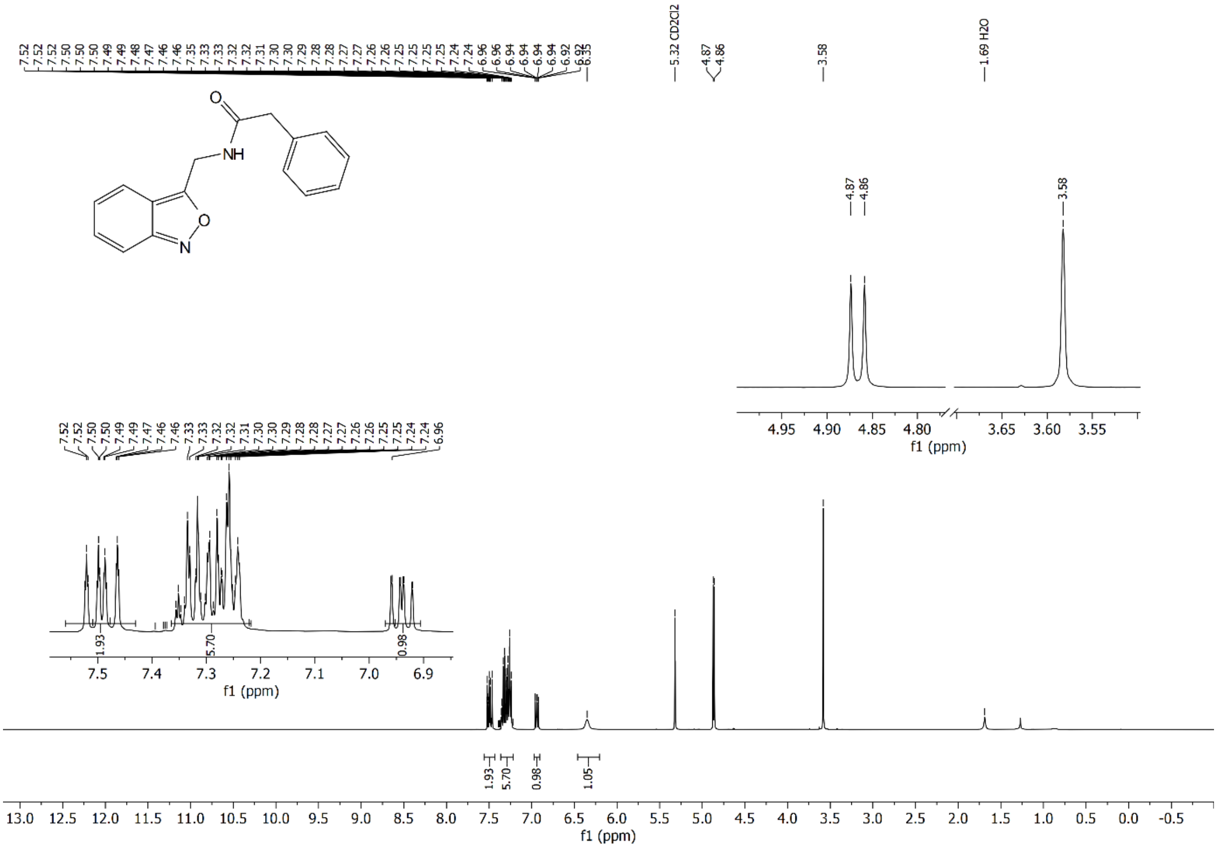

^{13}C NMR spectrum (101 MHz, CD_2Cl_2) of **5k**.

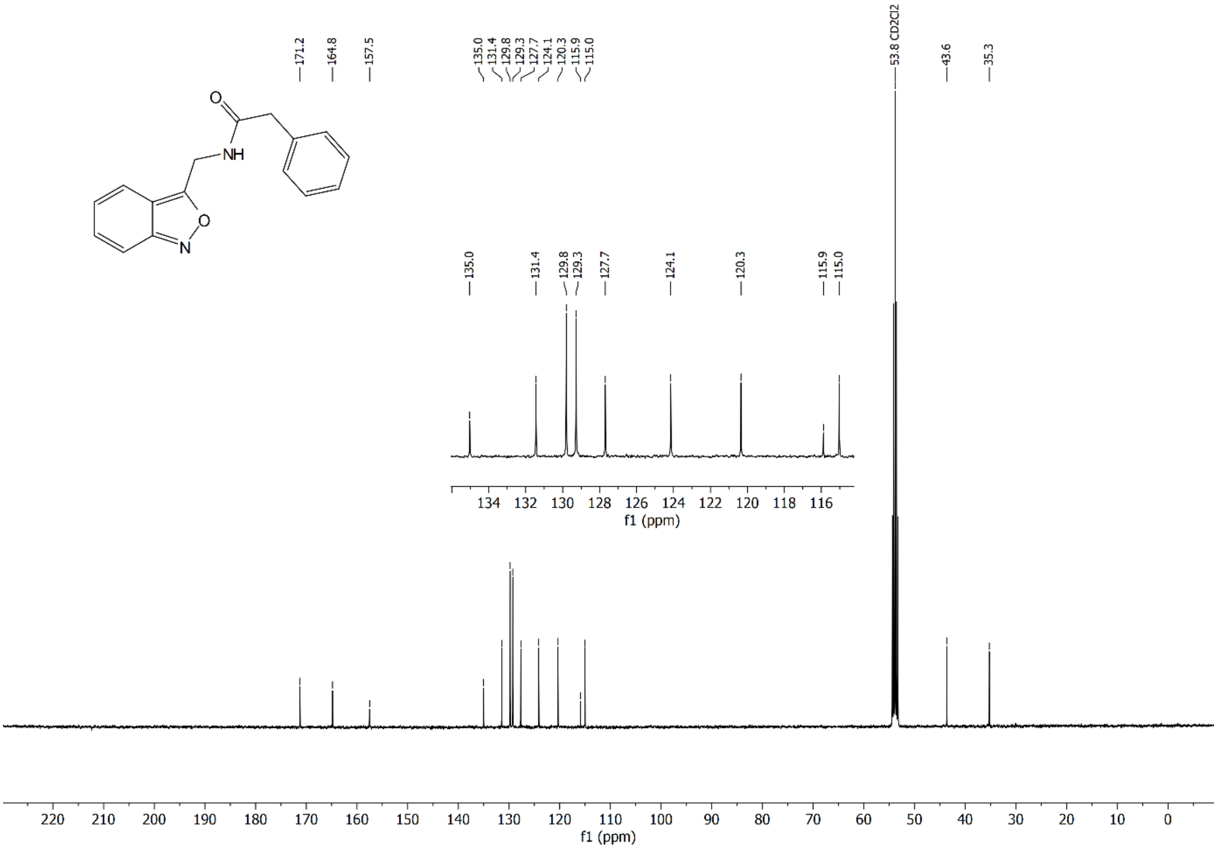

^{1}H NMR spectrum (400 MHz, CD_2Cl_2) of **5l**.

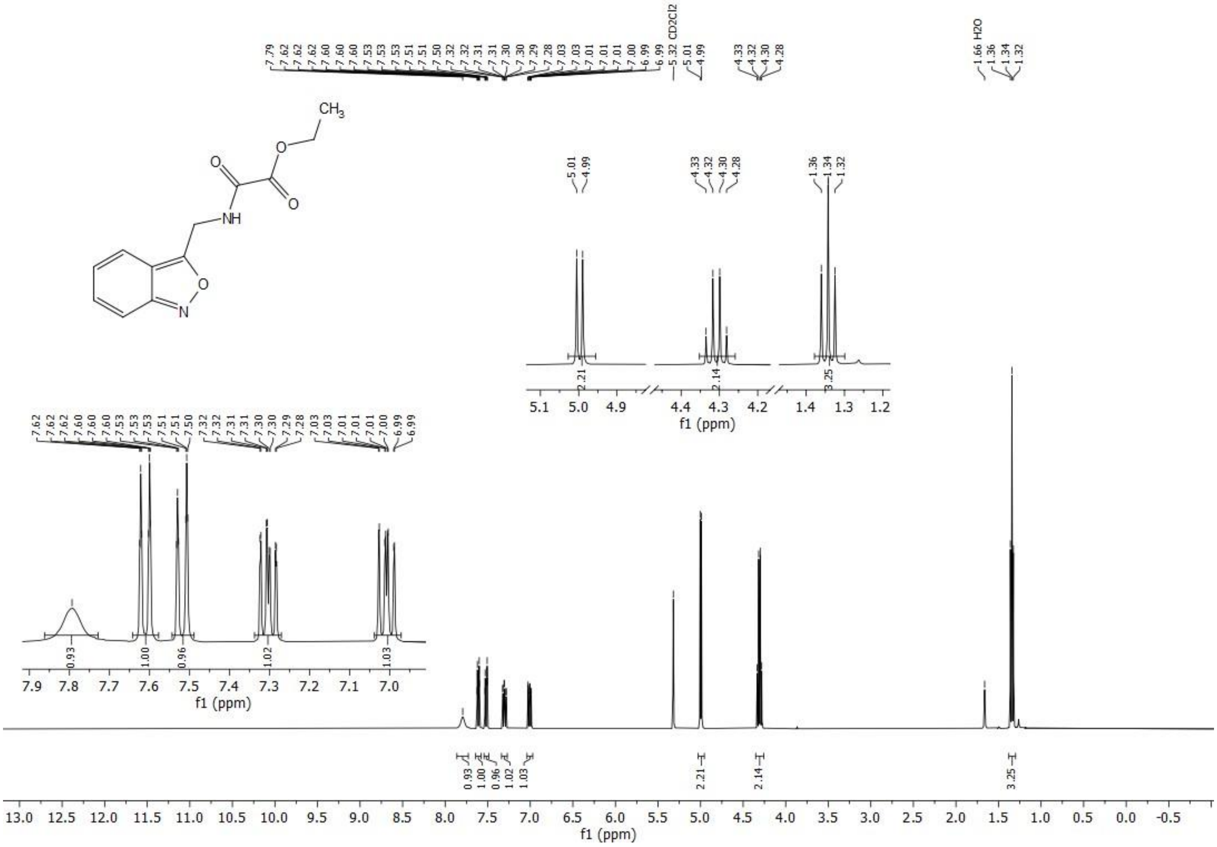

^{13}C NMR spectrum (101 MHz, CD_2Cl_2) of **5l**.

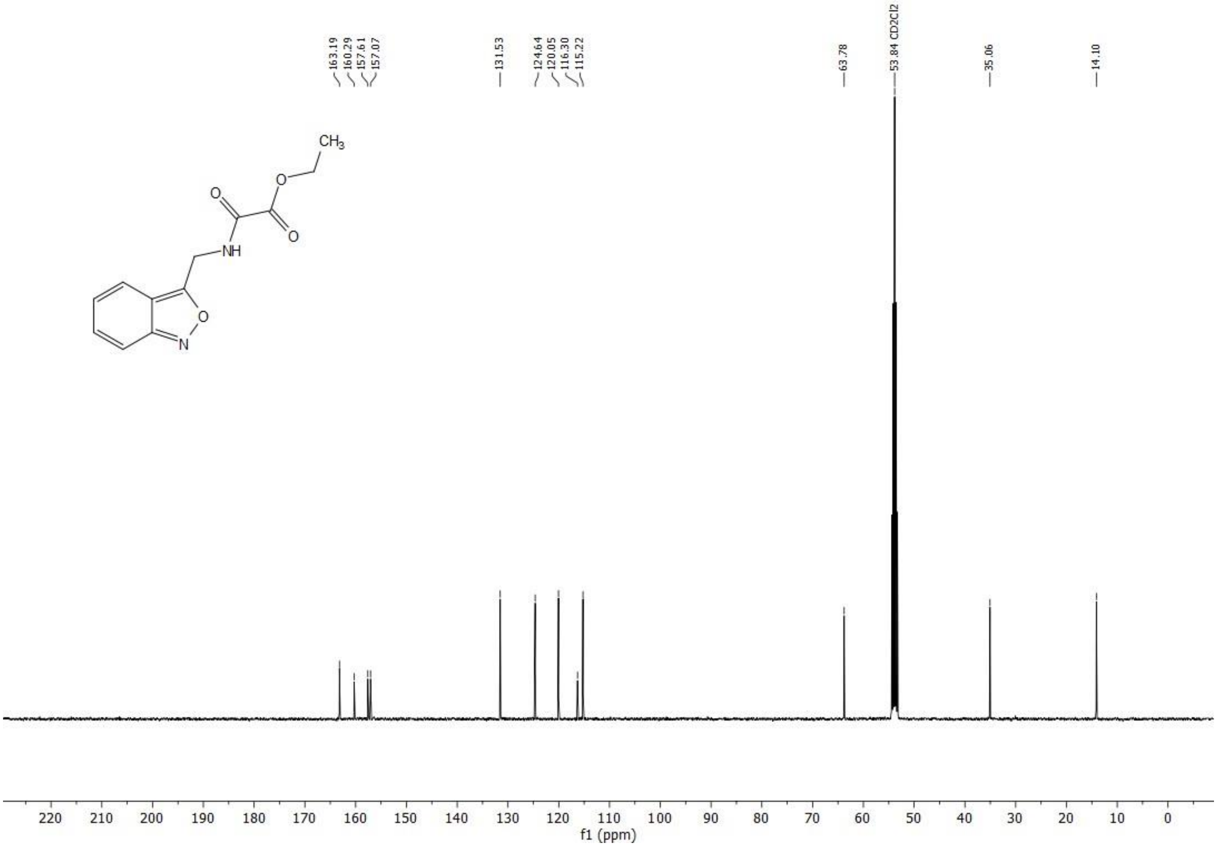

^{1}H NMR spectrum (400 MHz, CD_2Cl_2) of **5m**.

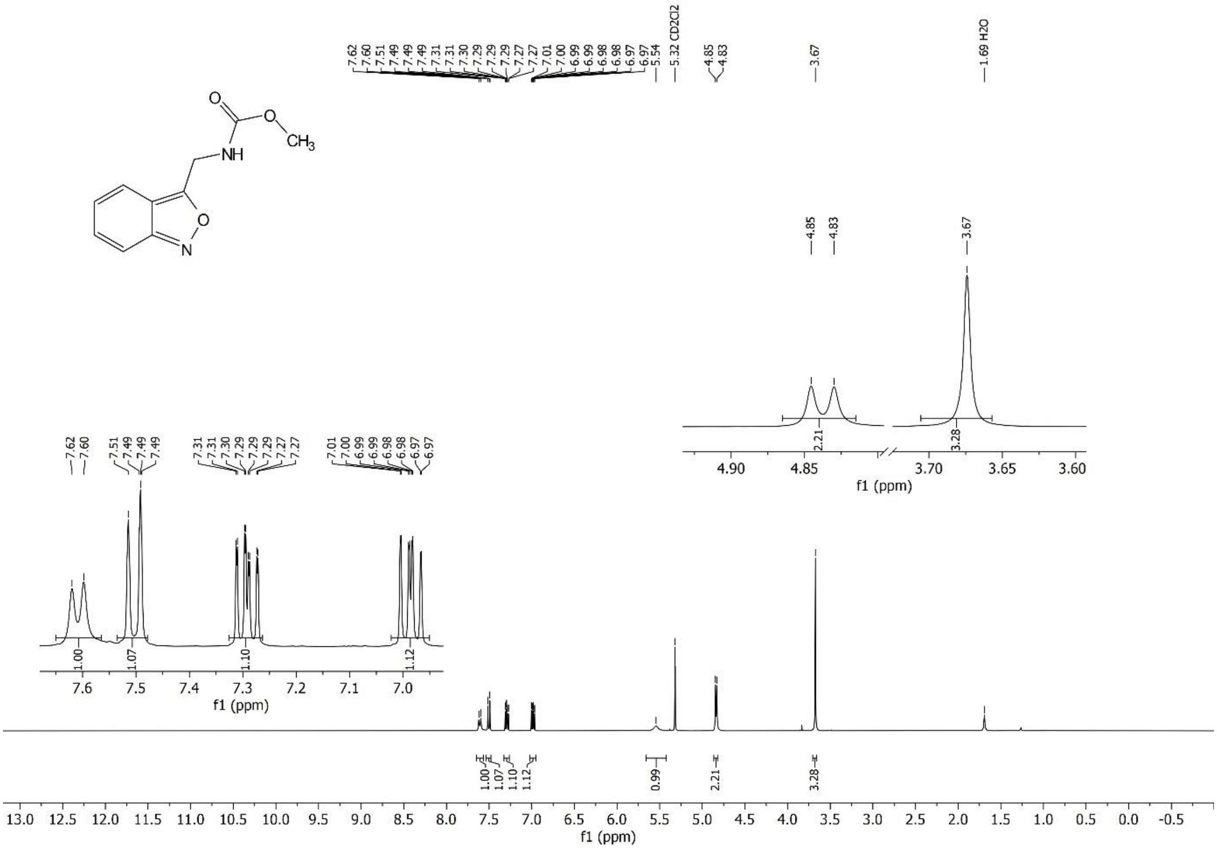

^{13}C NMR spectrum (101 MHz, CD_2Cl_2) of **5m**.

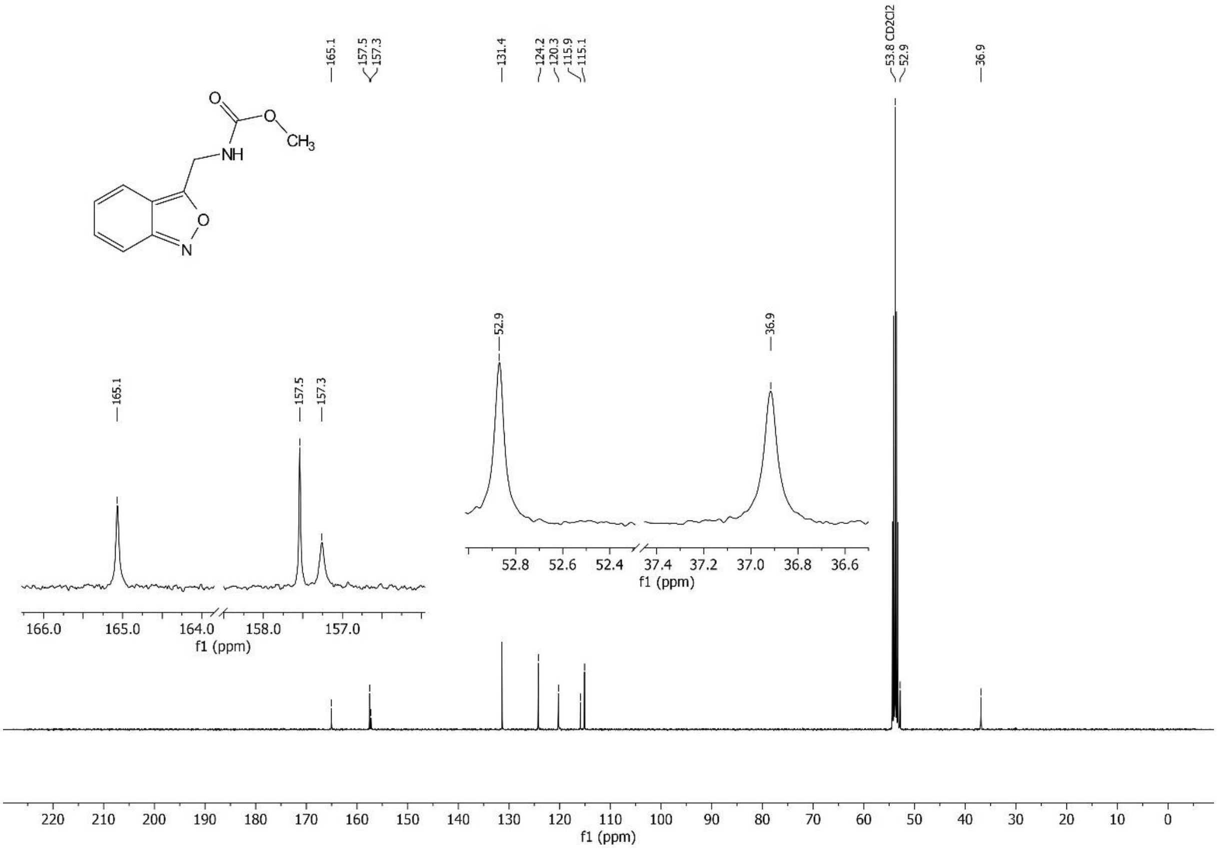

^1H NMR spectrum (600 MHz, CD_2Cl_2) of **5n**.

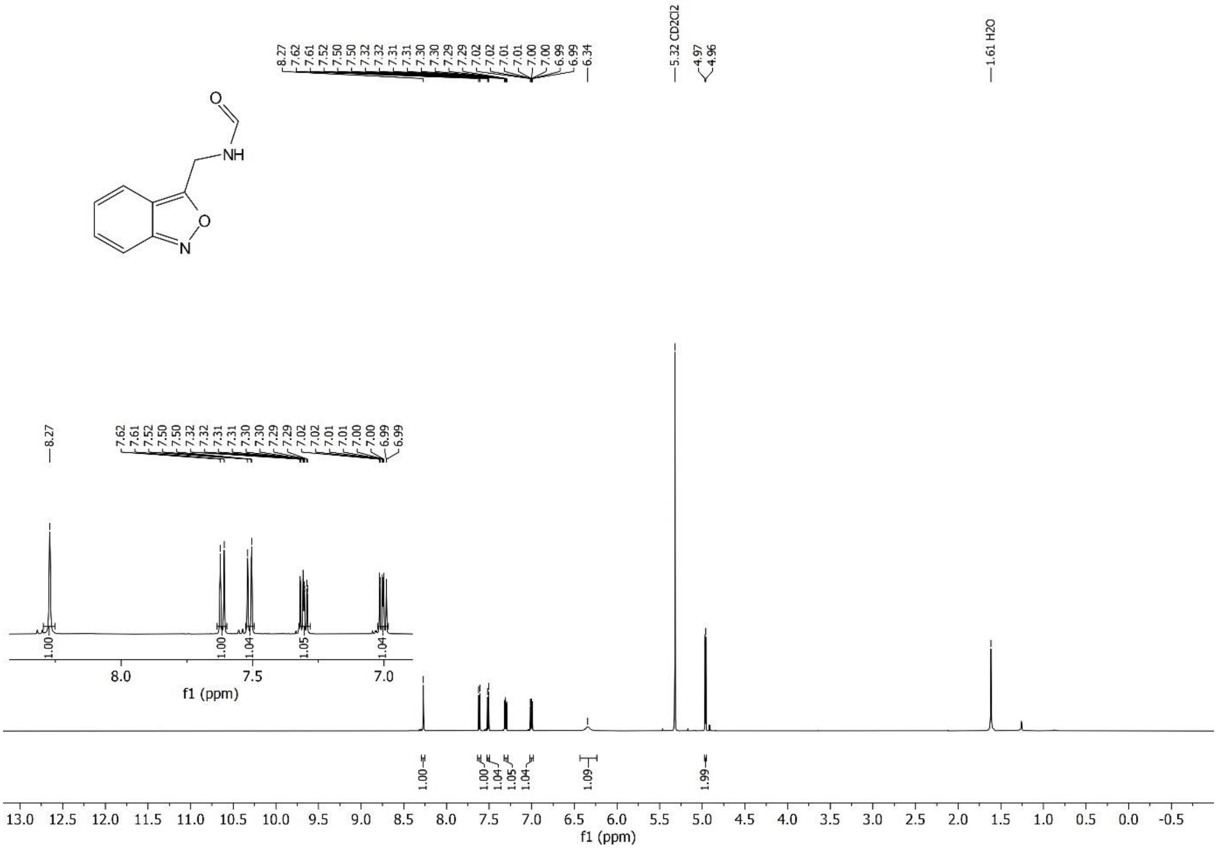

^{13}C NMR spectrum (151 MHz, CD_2Cl_2) of **5n**.

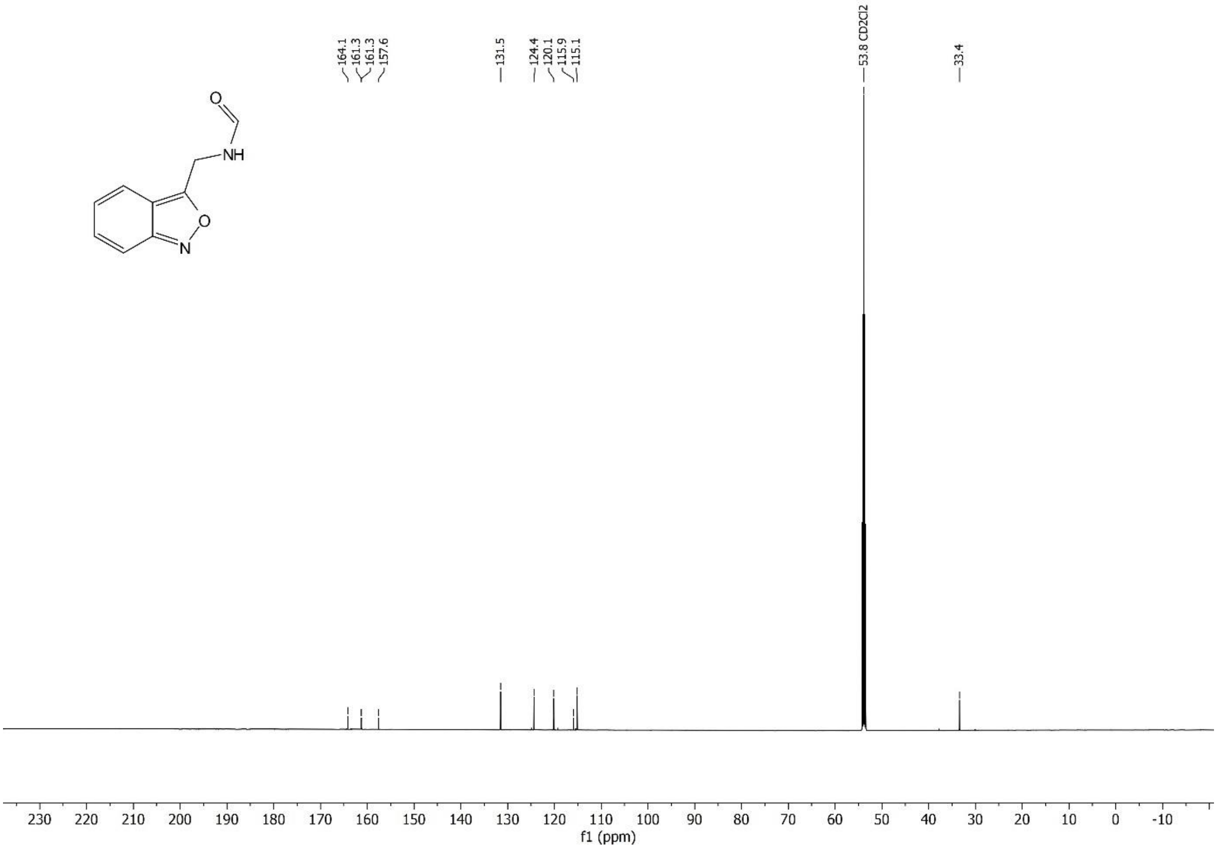

^{1}H NMR spectrum (400 MHz, DMSO-[D6]) of **5o**.

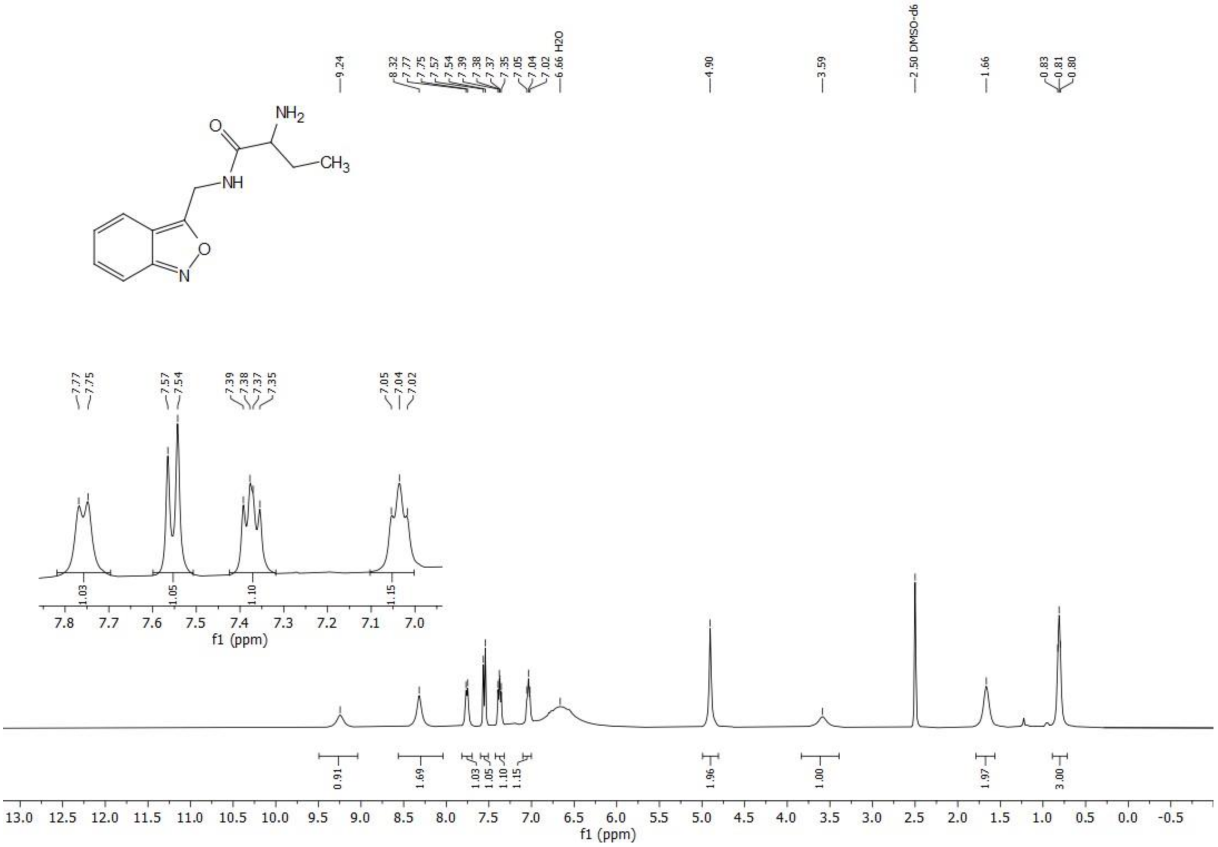

^{13}C NMR spectrum (101 MHz, DMSO-[D6]) of **5o**.

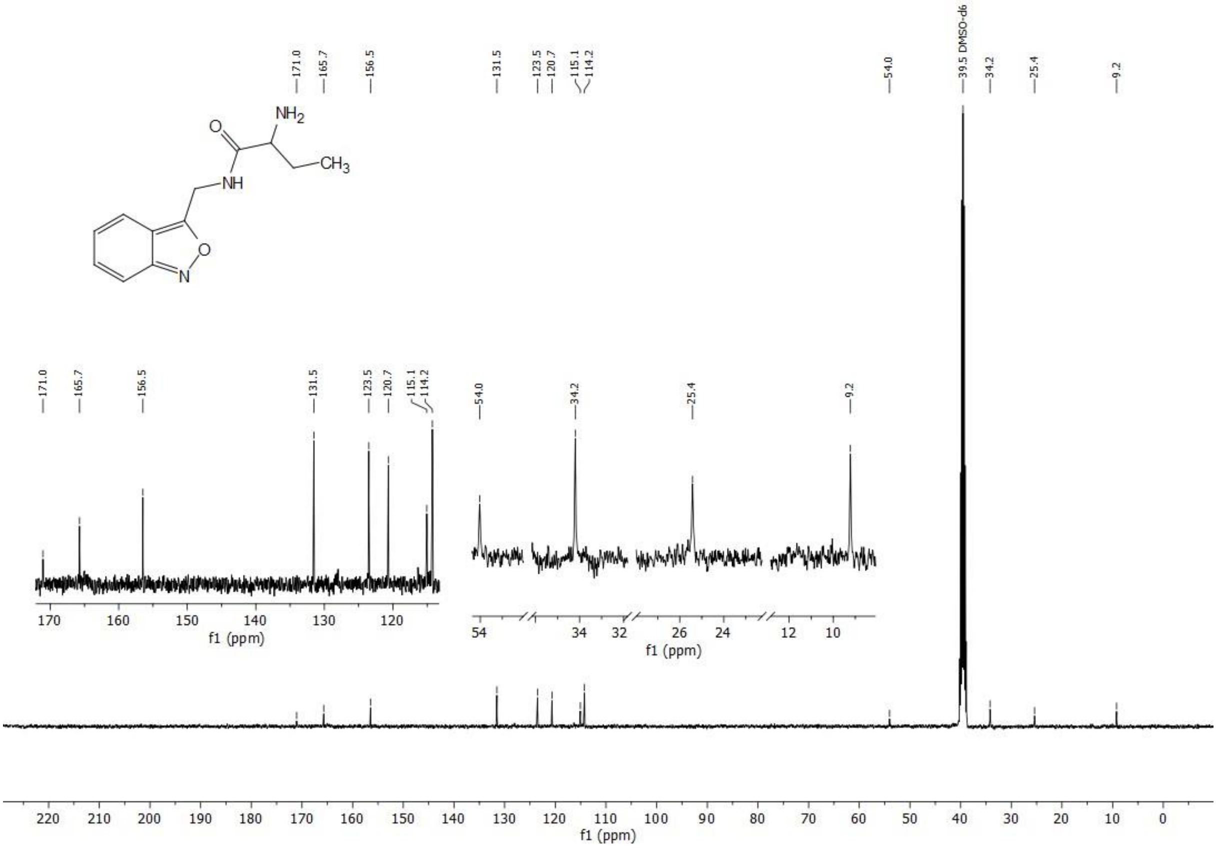

^{1}H NMR spectrum (400 MHz, CD_3CN) of **5p**.

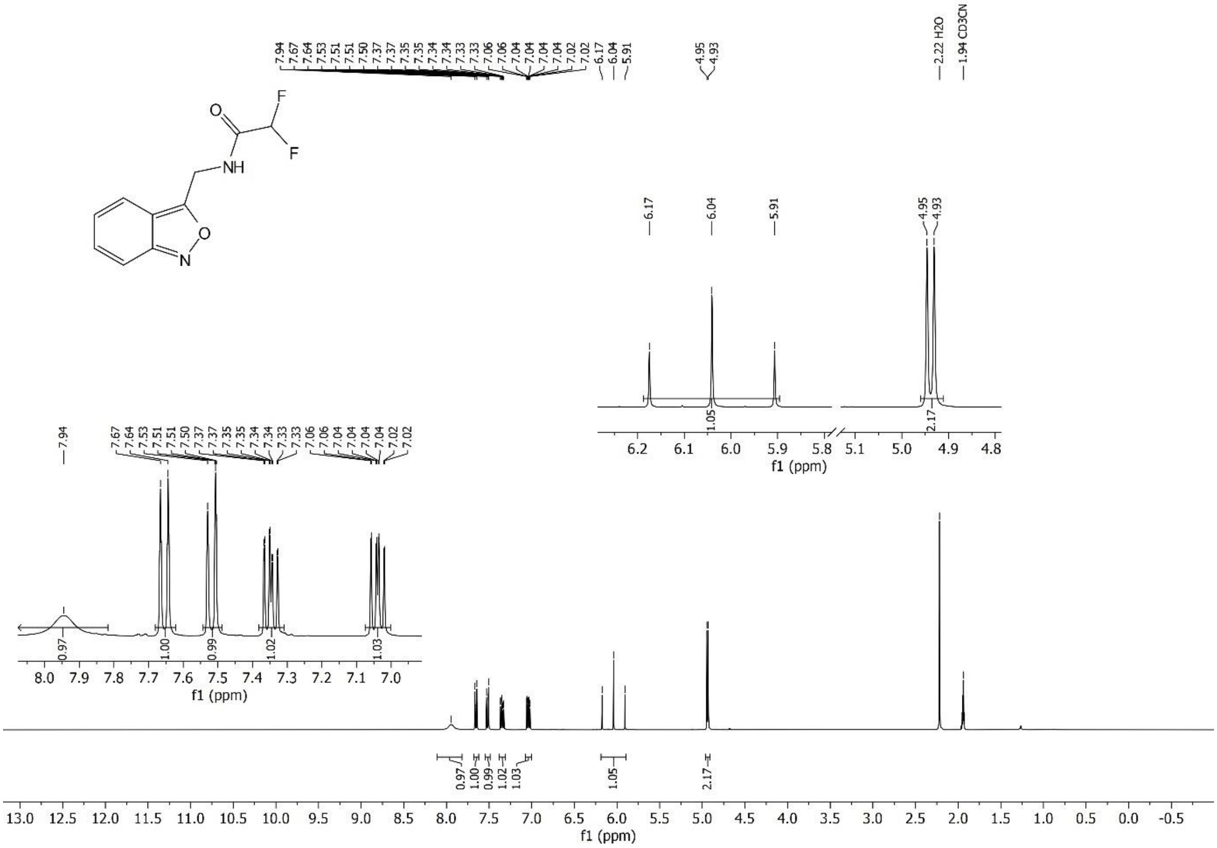

^{13}C NMR spectrum (101 MHz, CD_3CN) of **5p**.

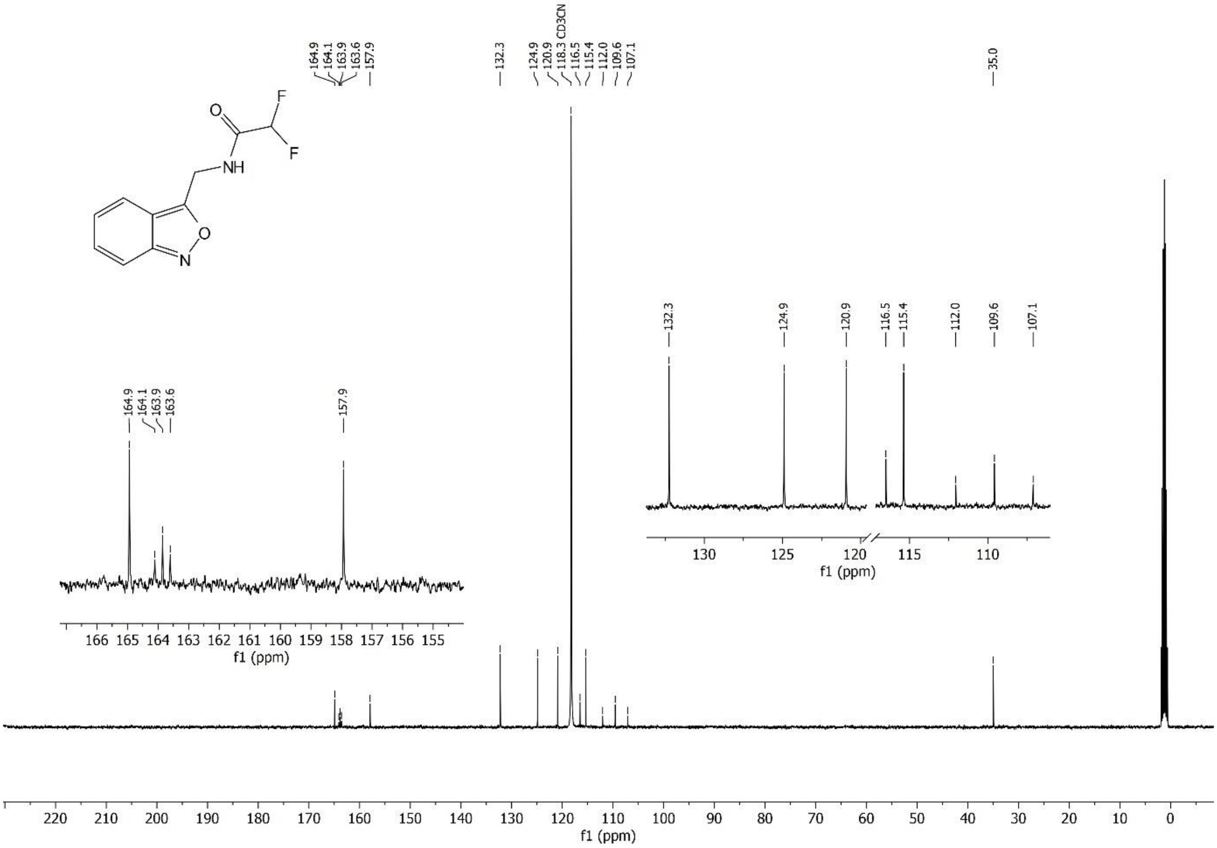

^{19}F NMR spectrum (376 MHz, CD_3CN) of **5p**.

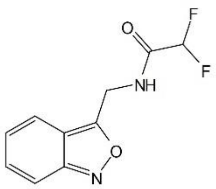

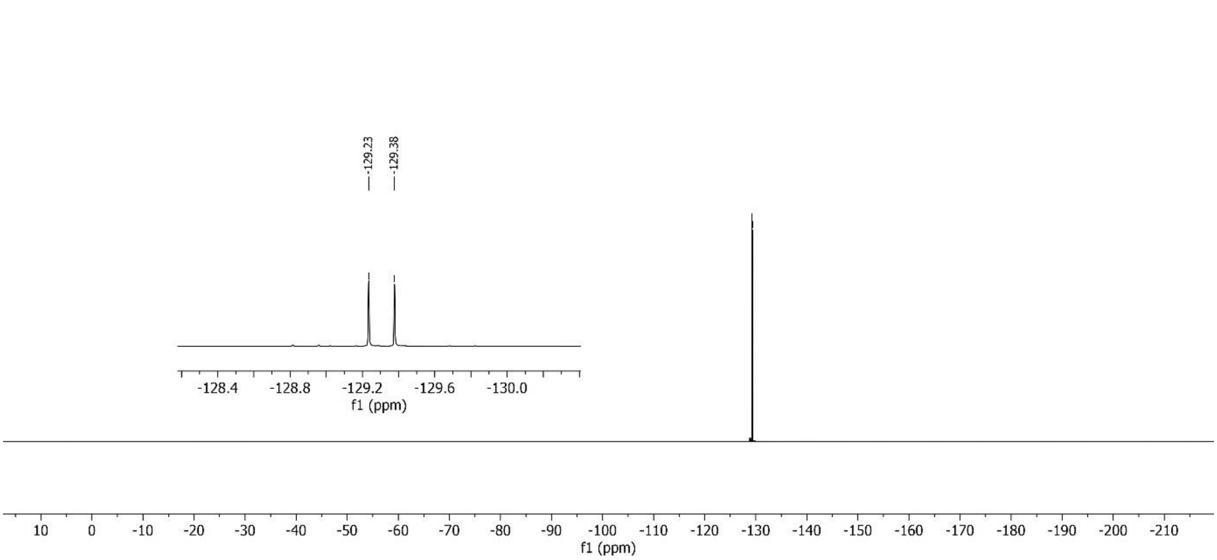

^{1}H NMR spectrum (400 MHz, CD_2Cl_2) of **5q**.

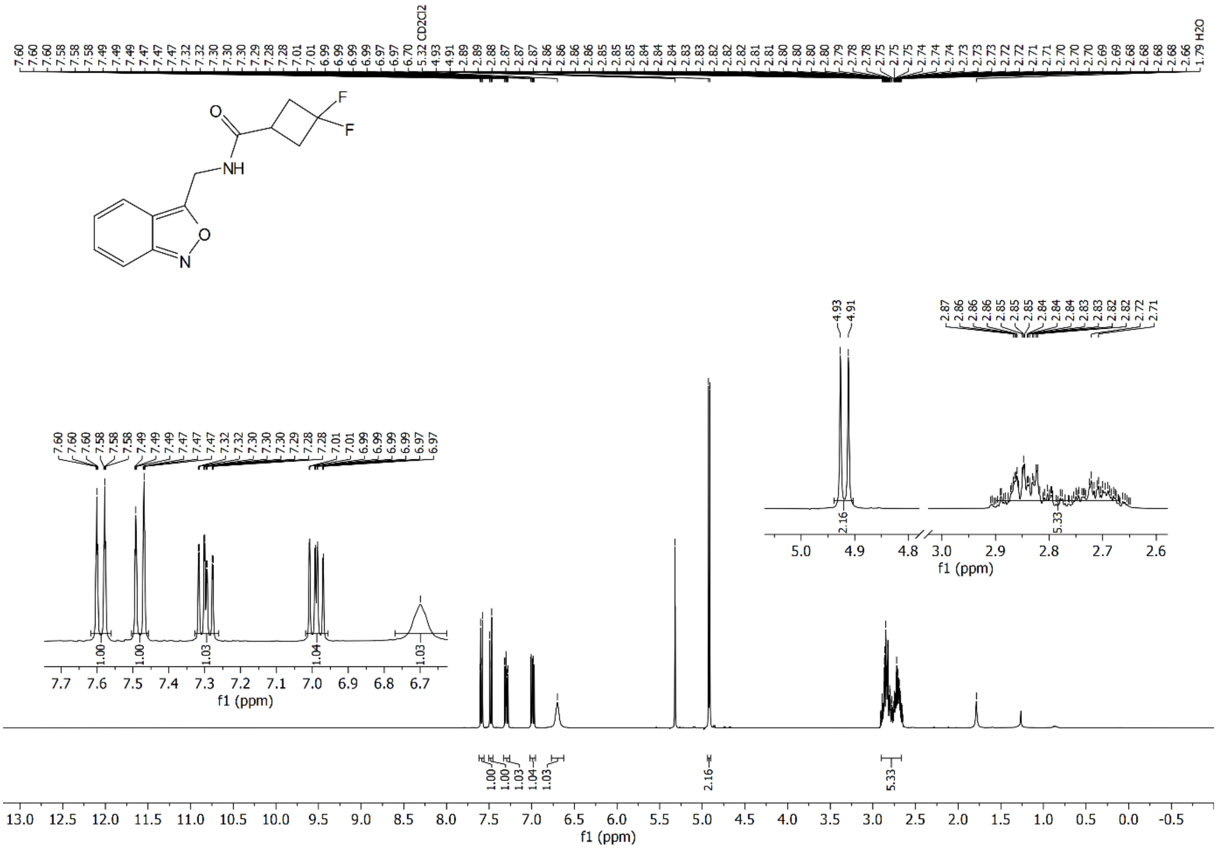

^{13}C NMR spectrum (101 MHz, CD_2Cl_2) of **5q**.

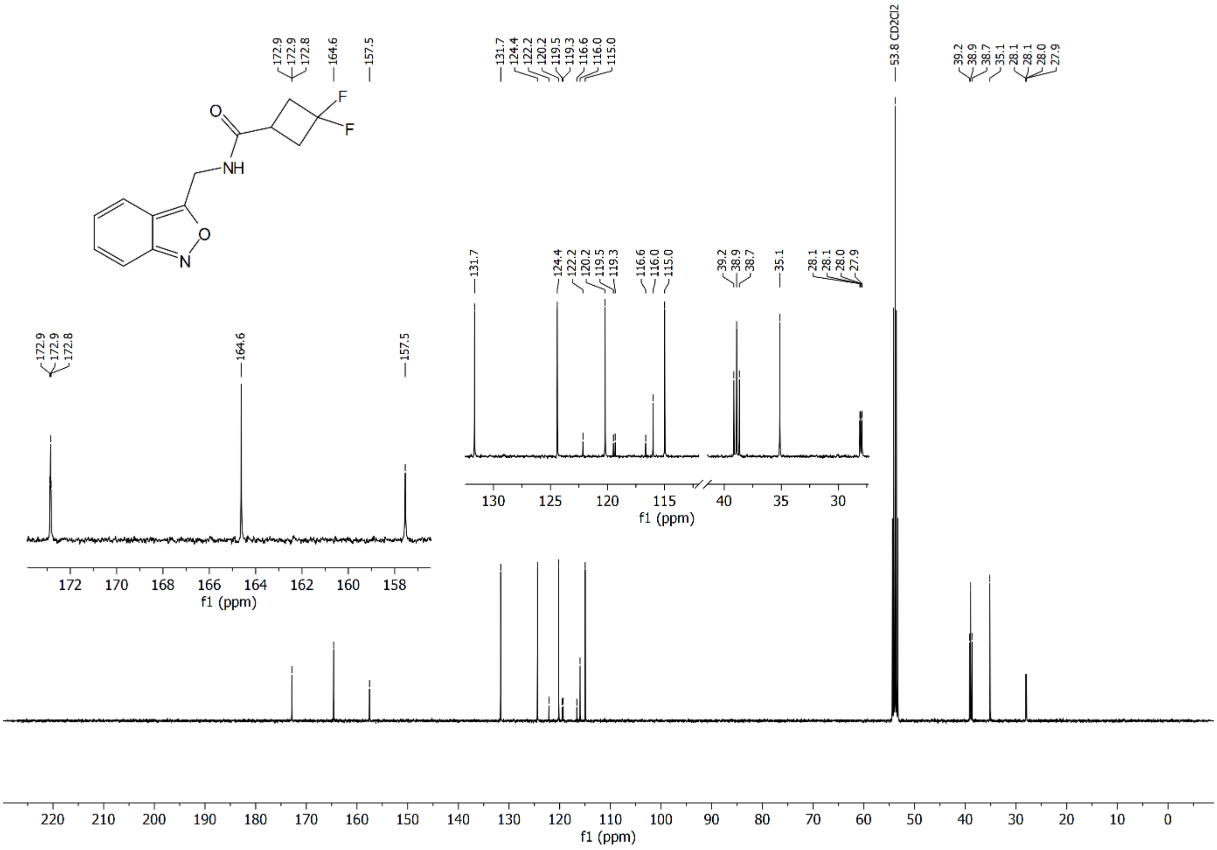

^{19}F NMR spectrum (376 MHz, CD_2Cl_2) of **5q**.

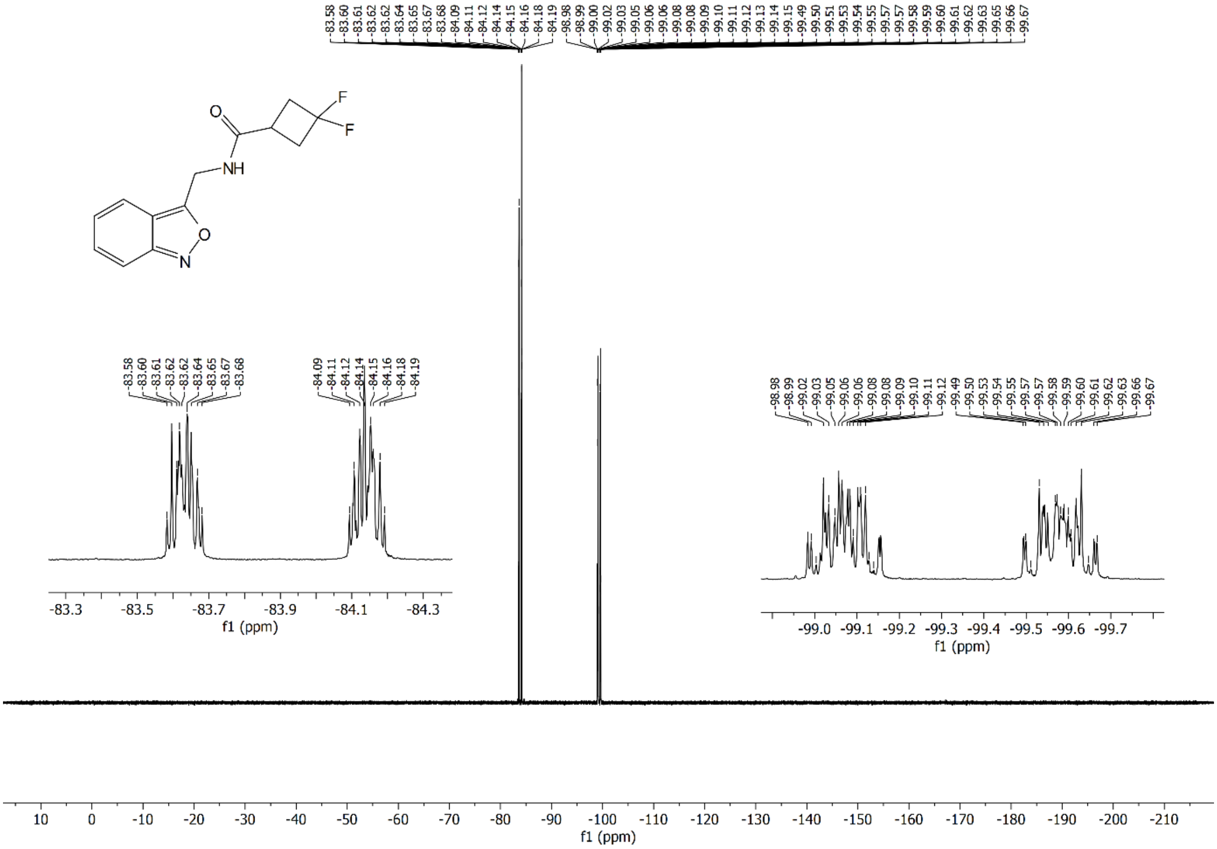

^{1}H NMR spectrum (400 MHz, CD_2Cl_2) of **5r**.

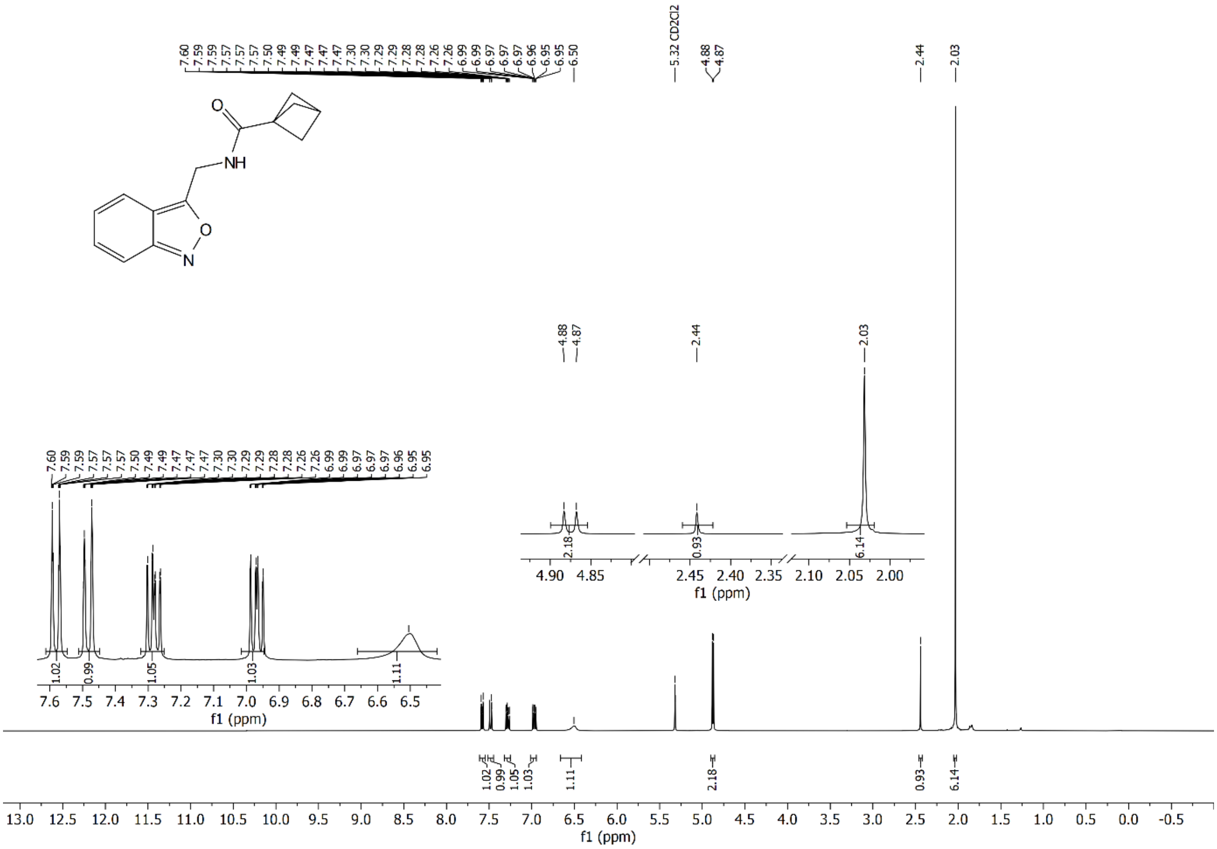

^{13}C NMR spectrum (101 MHz, CD_2Cl_2) of **5r**.

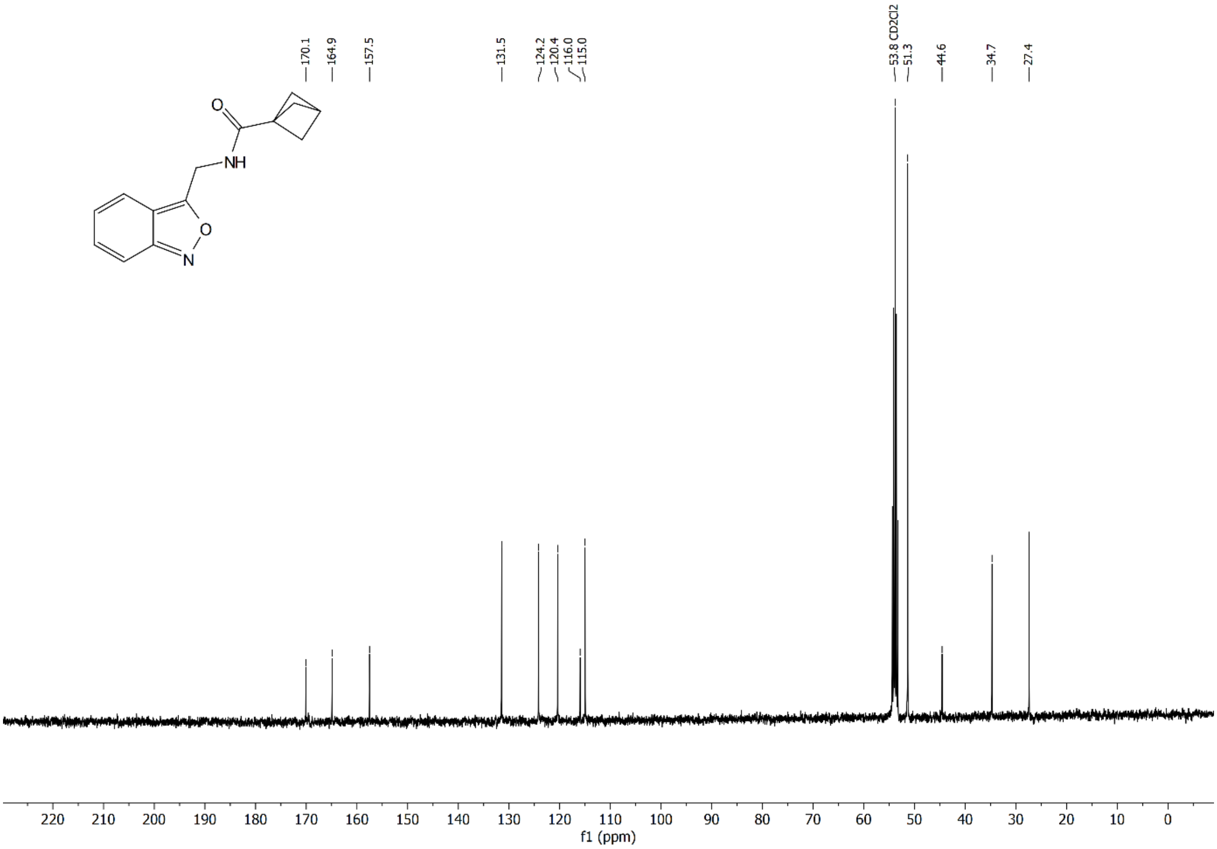

^{1}H NMR spectrum (400 MHz, CD_2Cl_2) of **5s**.

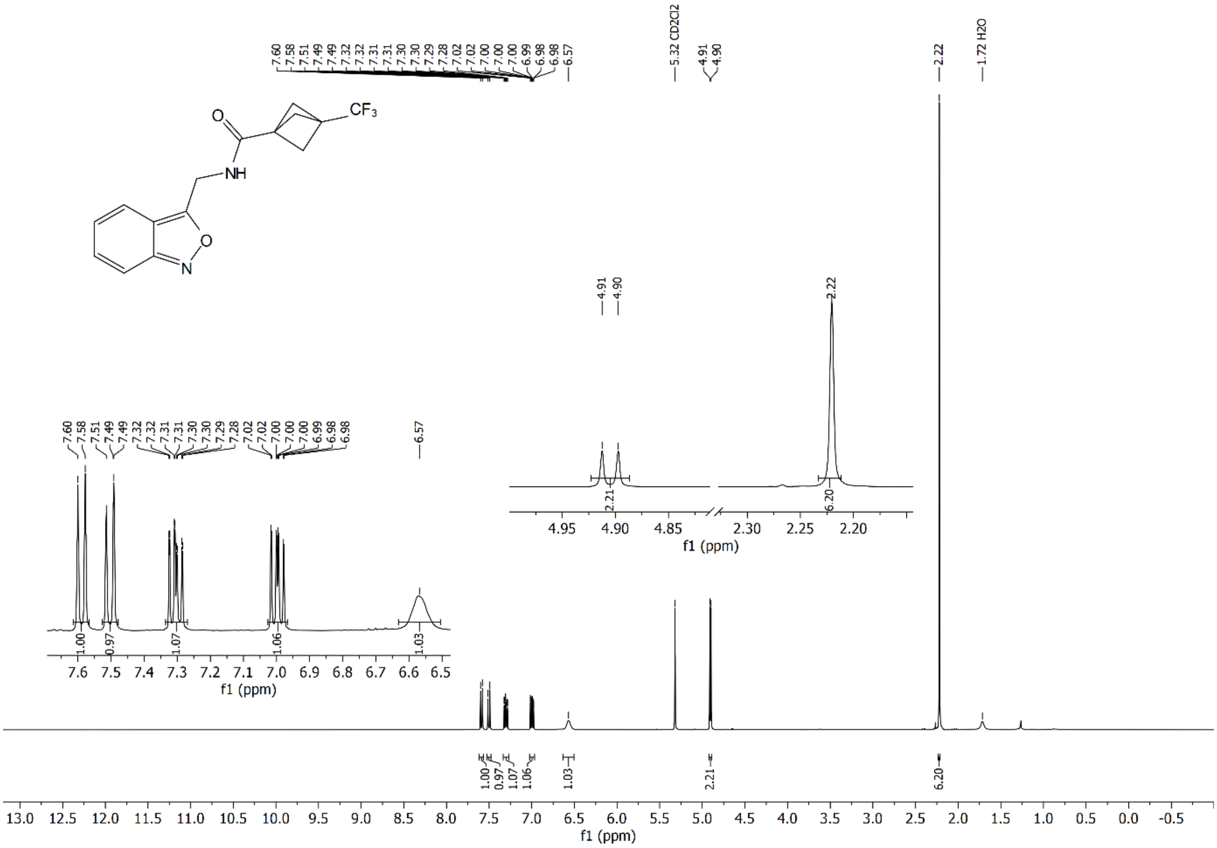

^{13}C NMR spectrum (101 MHz, CD_2Cl_2) of **5s**.

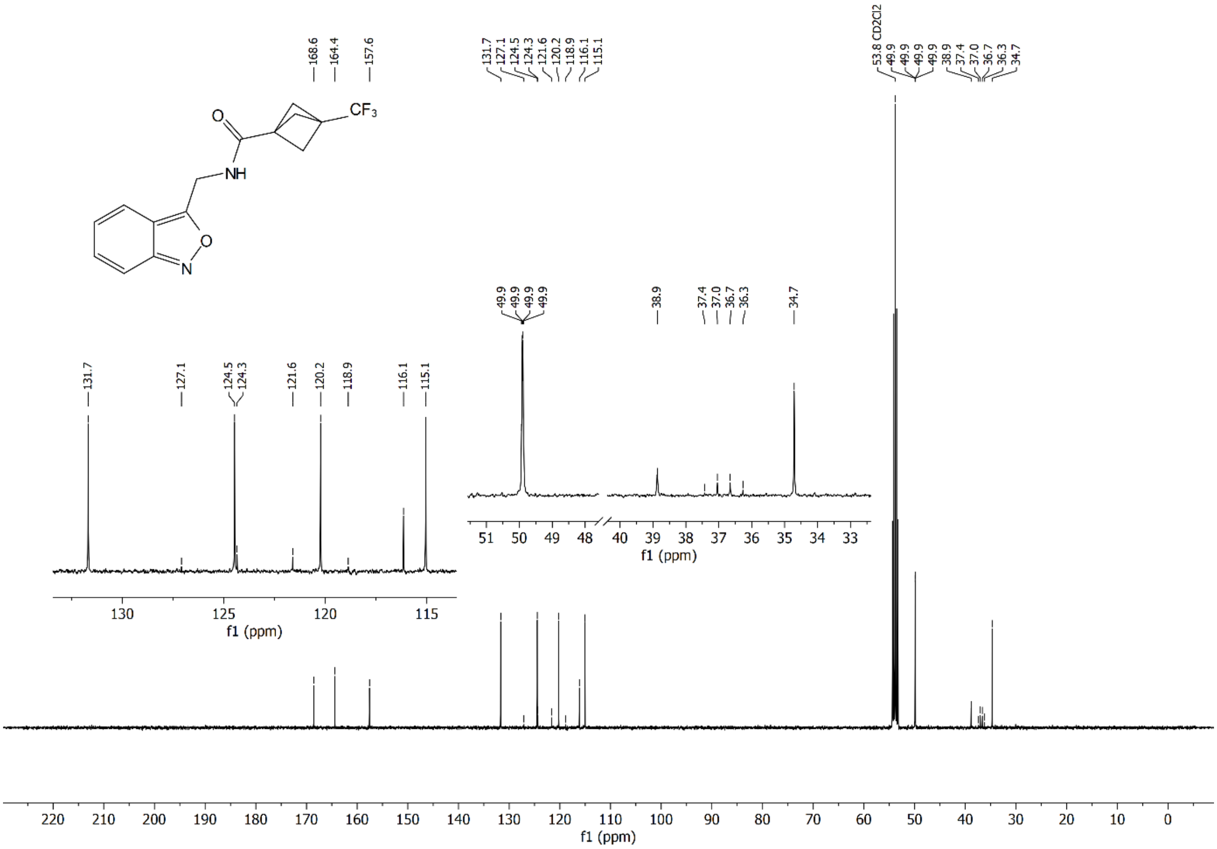

^{19}F NMR spectrum (376 MHz, CD_2Cl_2) of **5s**.

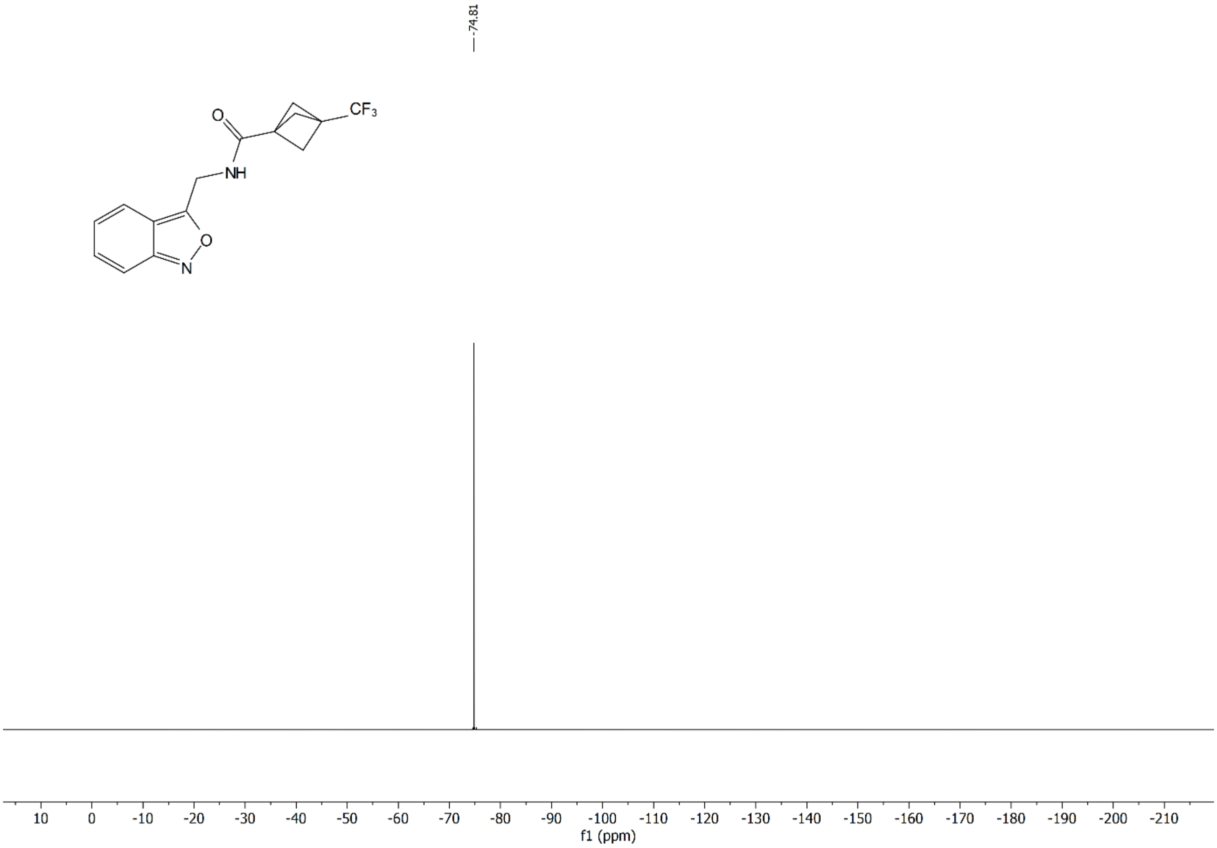

^{1}H NMR spectrum (400 MHz, CD_2Cl_2) of **5t**.

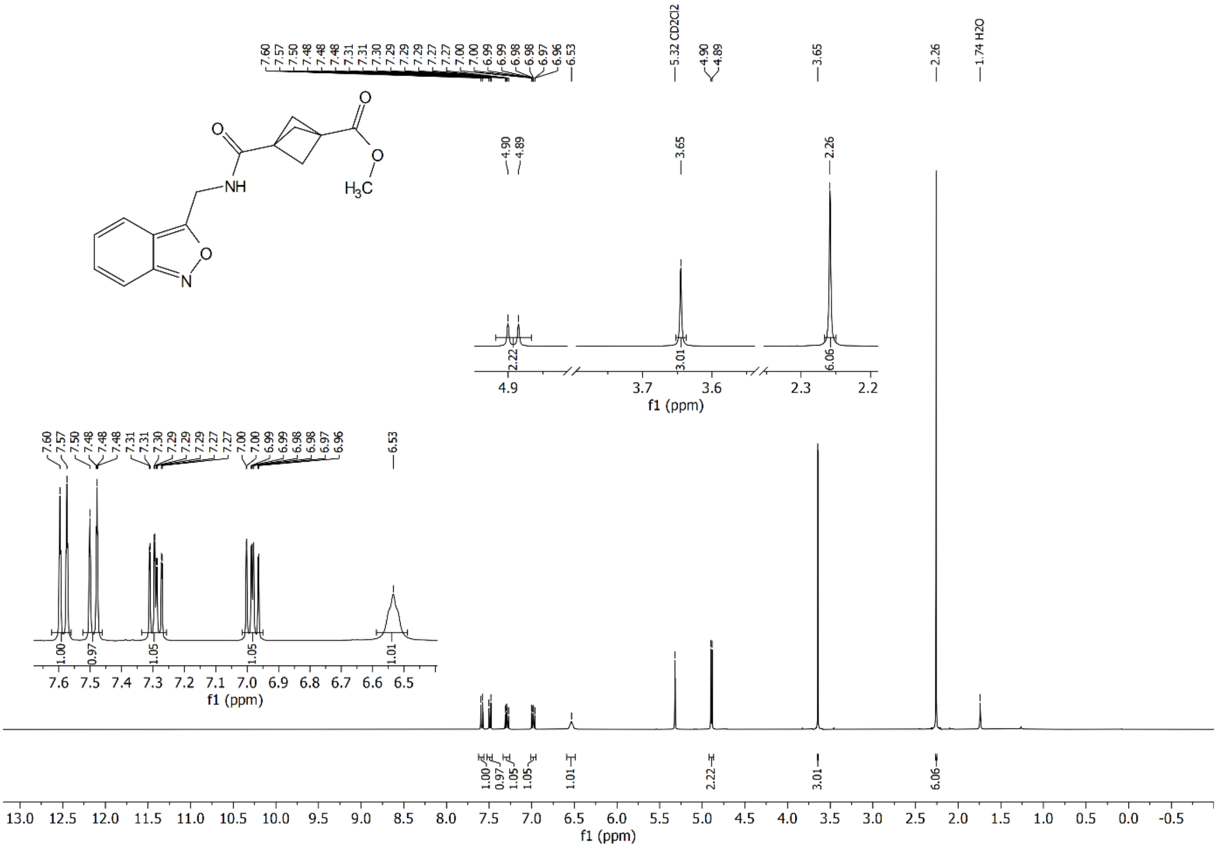

^{13}C NMR spectrum (101 MHz, CD_2Cl_2) of **5t**.

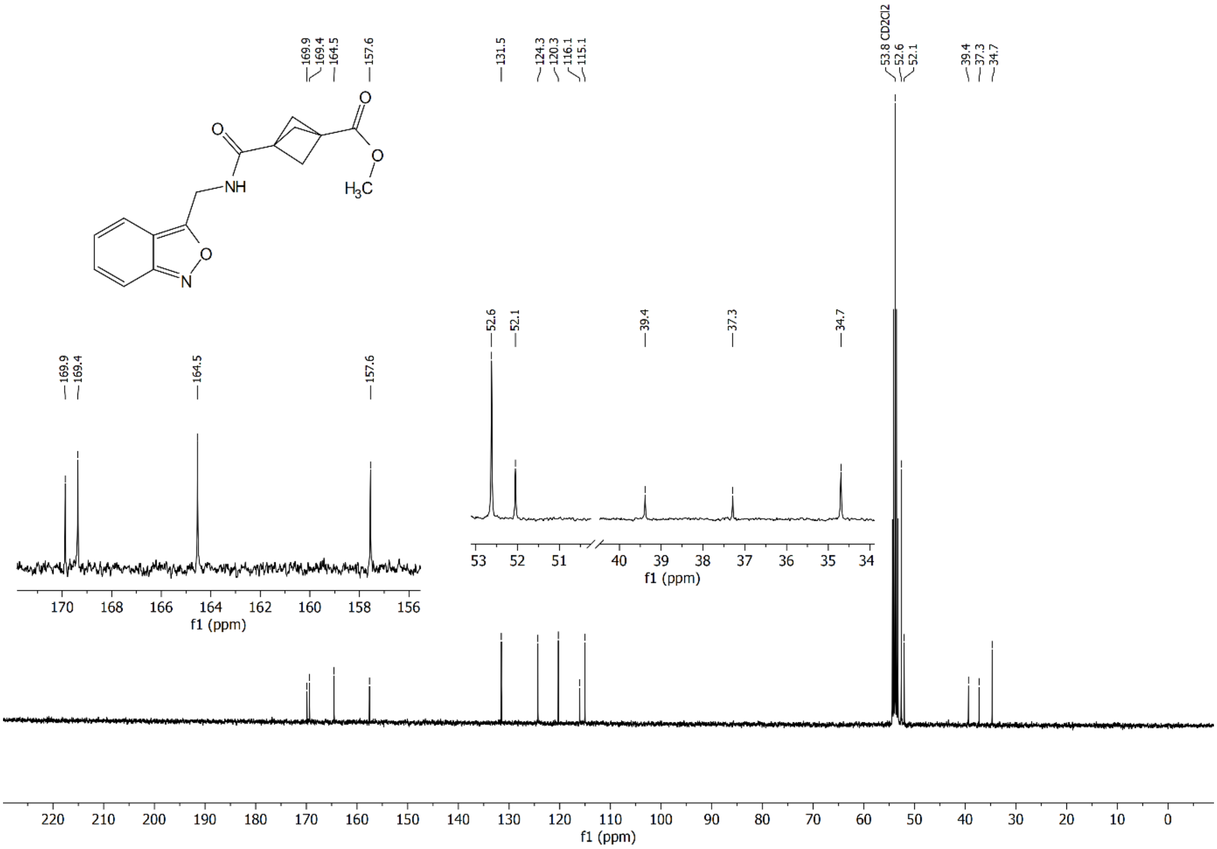

^{1}H NMR spectrum (400 MHz, CD_2Cl_2) of **5u**.

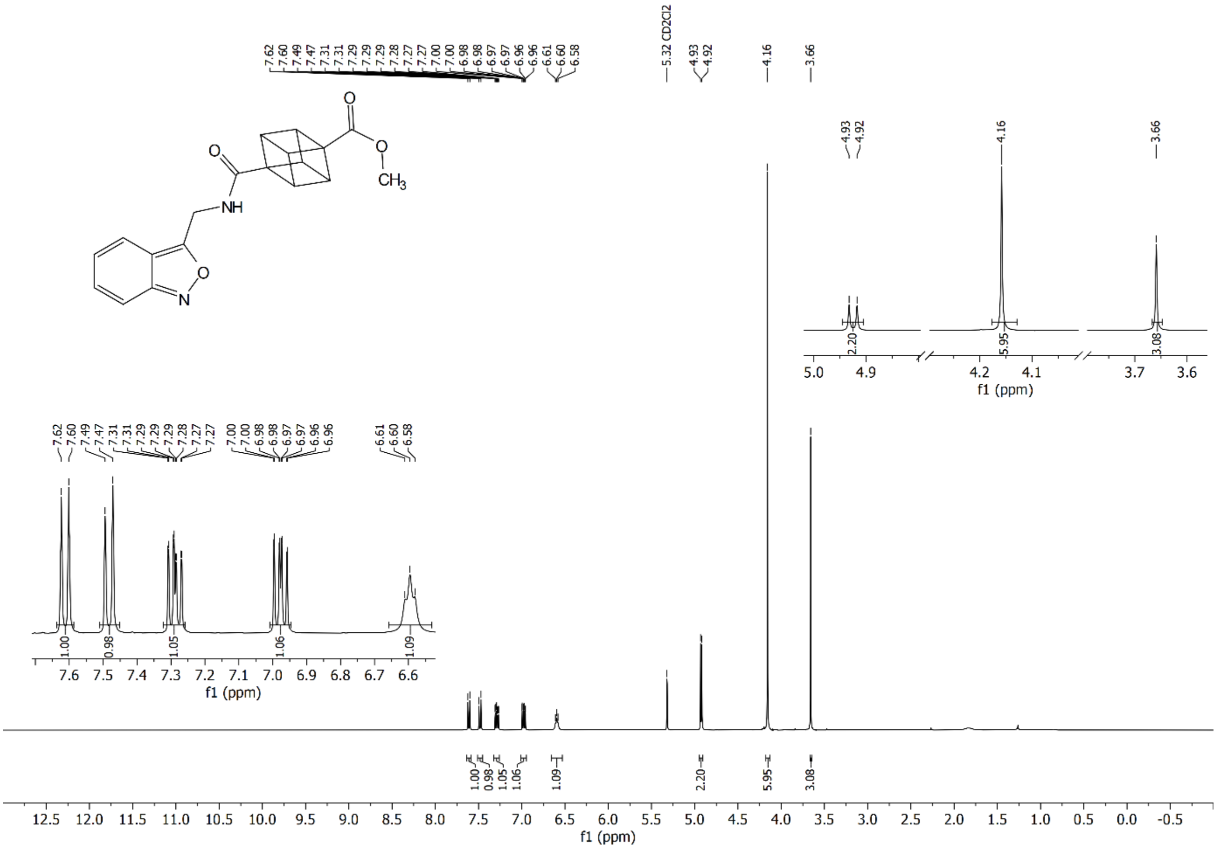

^{13}C NMR spectrum (101 MHz, CD_2Cl_2) of **5u**.

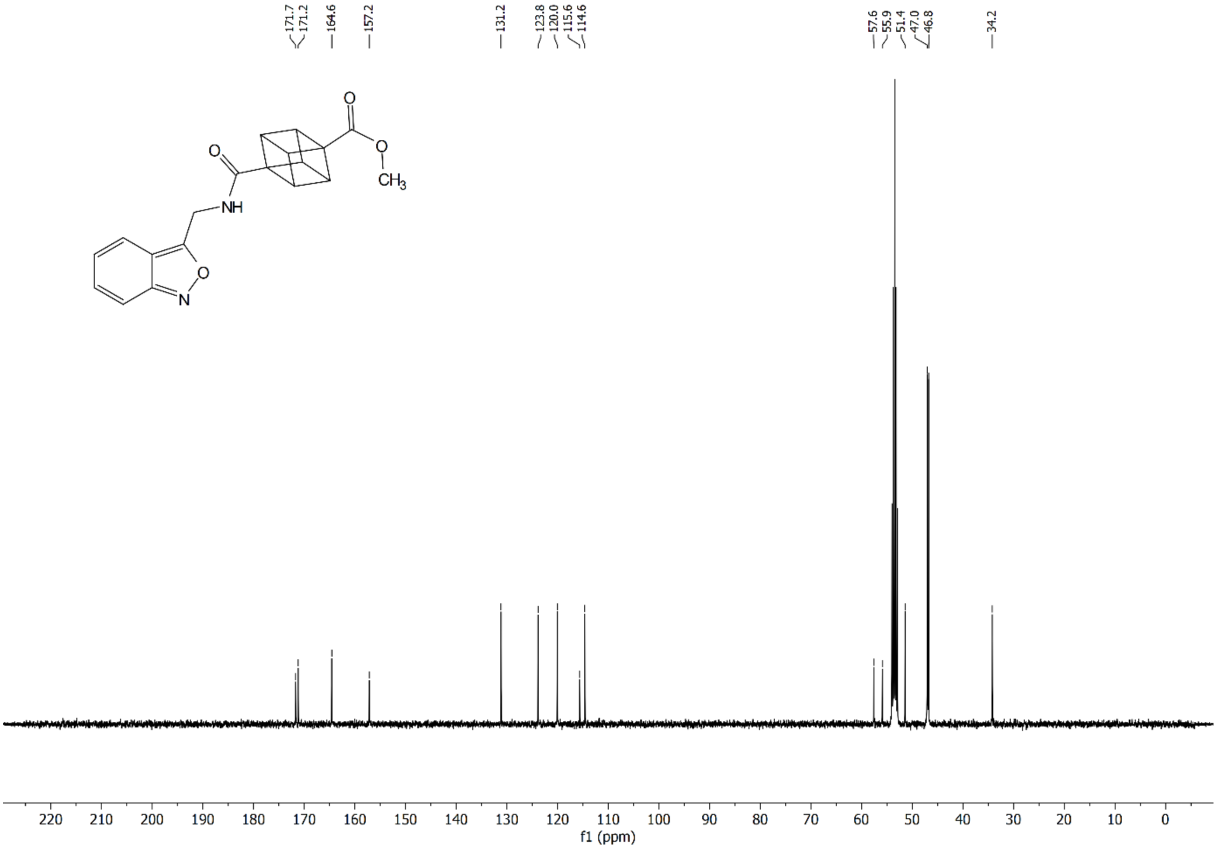

^{1}H NMR spectrum (400 MHz, CD_2Cl_2) of **5v**.

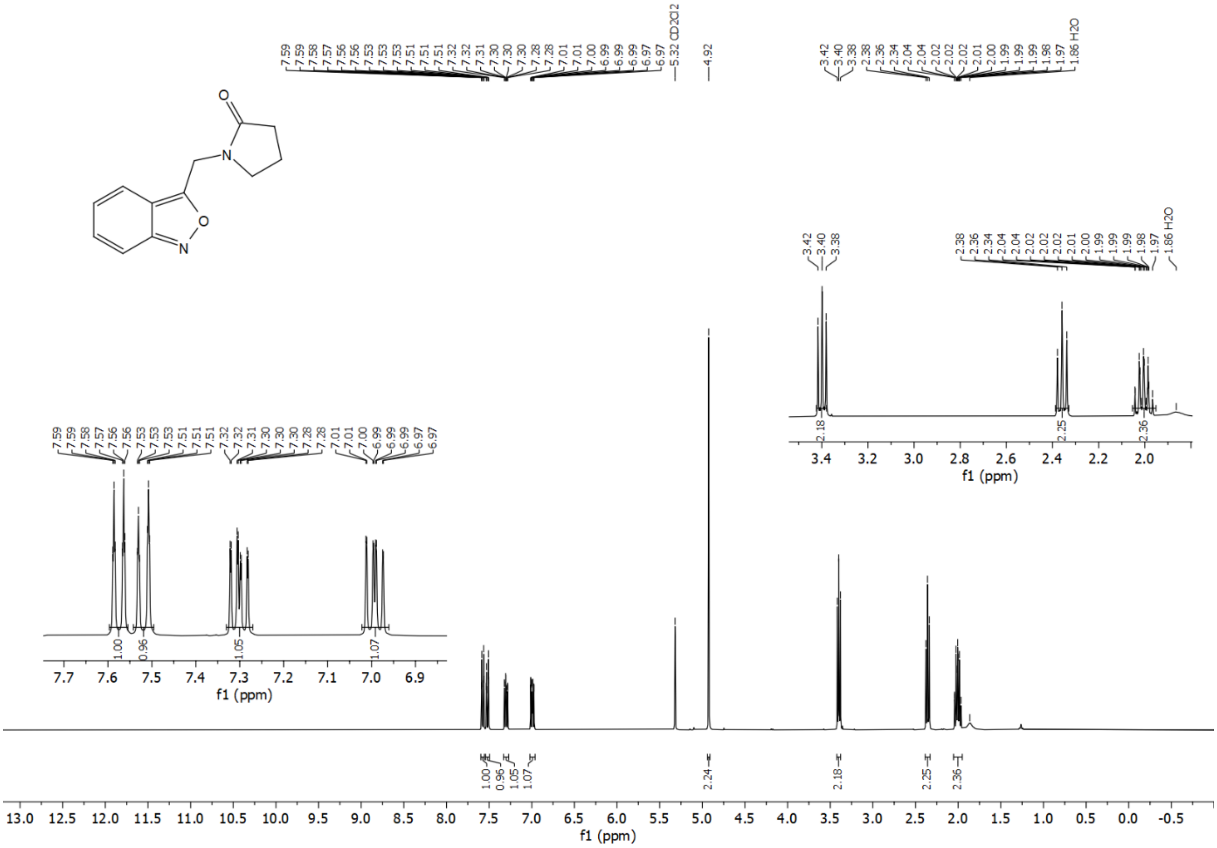

^{13}C NMR spectrum (101 MHz, CD_2Cl_2) of **5v**.

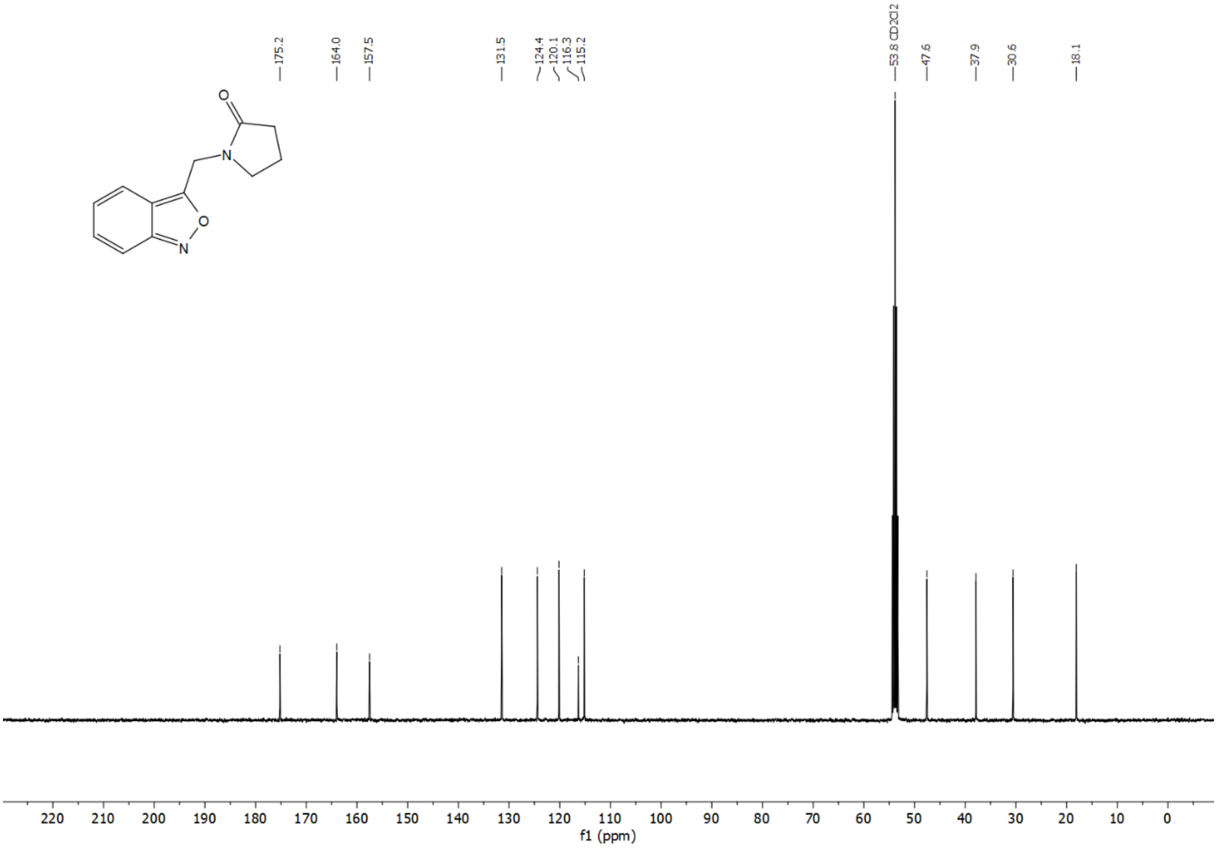

^{1}H NMR spectrum (400 MHz, CD_2Cl_2) of **5w**.

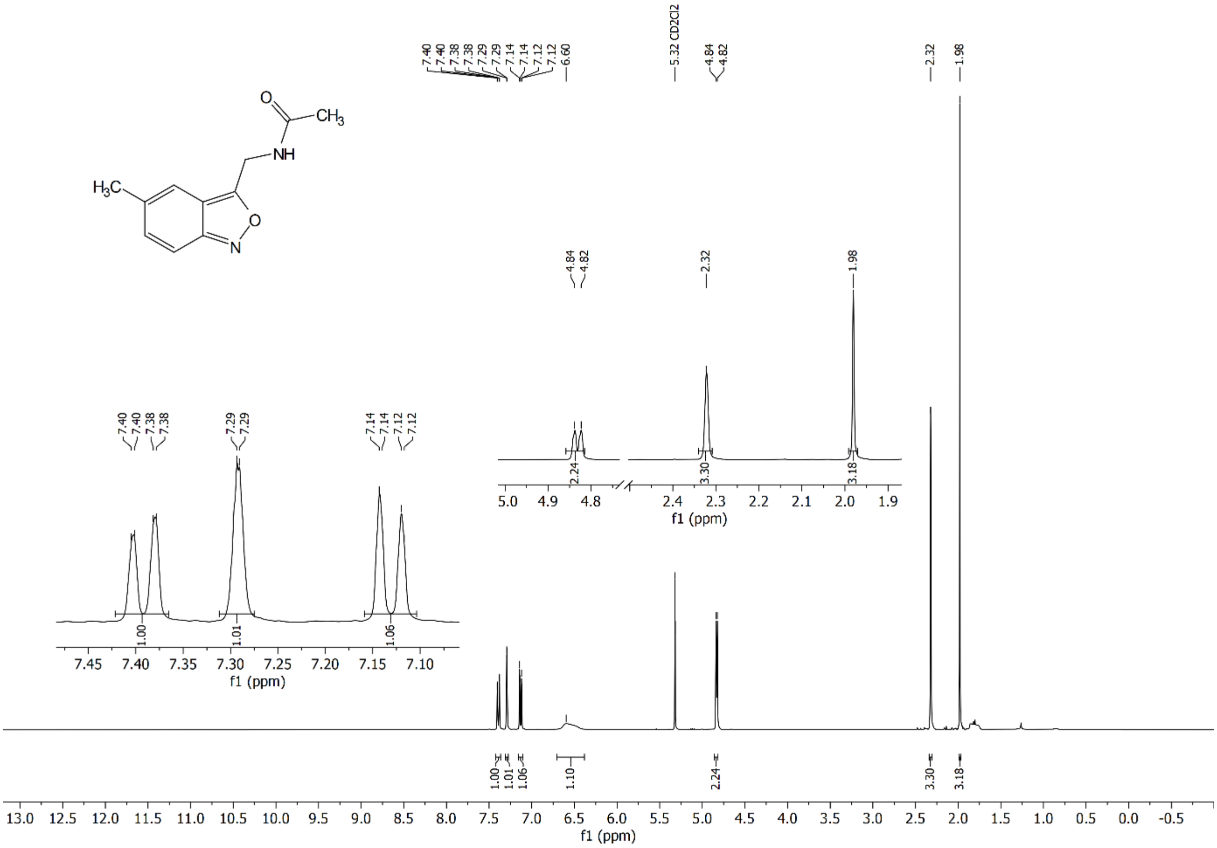

^{13}C NMR spectrum (101 MHz, CD_2Cl_2) of **5w**.

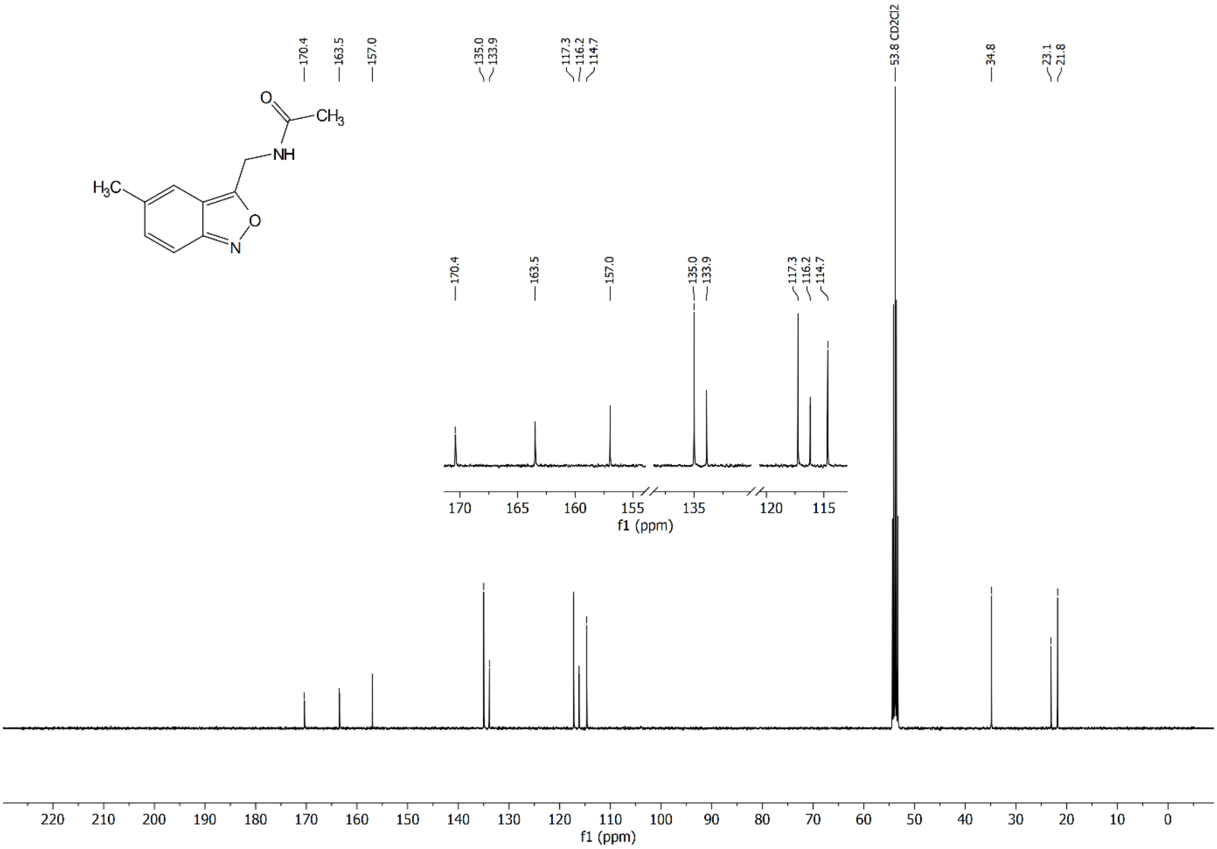

^{1}H NMR spectrum (400 MHz, CD_2Cl_2) of **5x**.

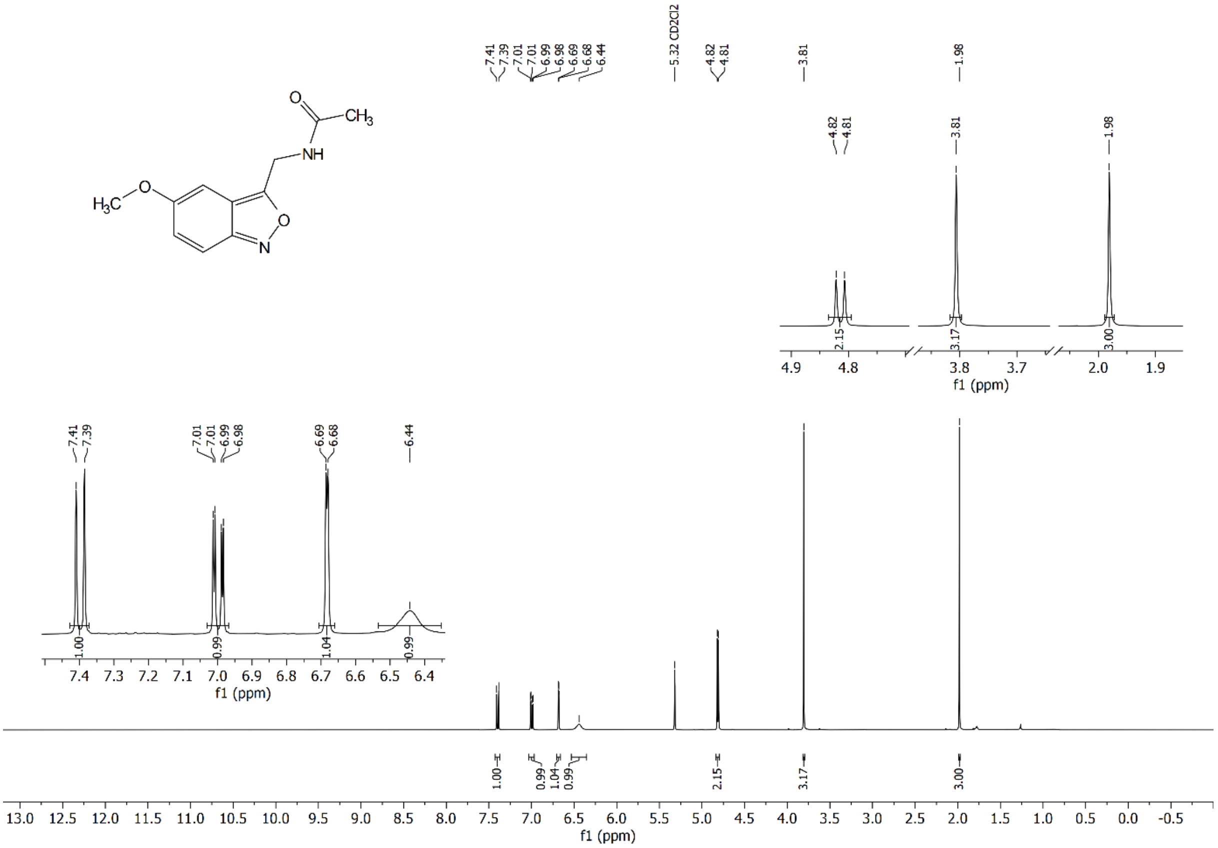

^{13}C NMR spectrum (101 MHz, CD_2Cl_2) of **5x**.

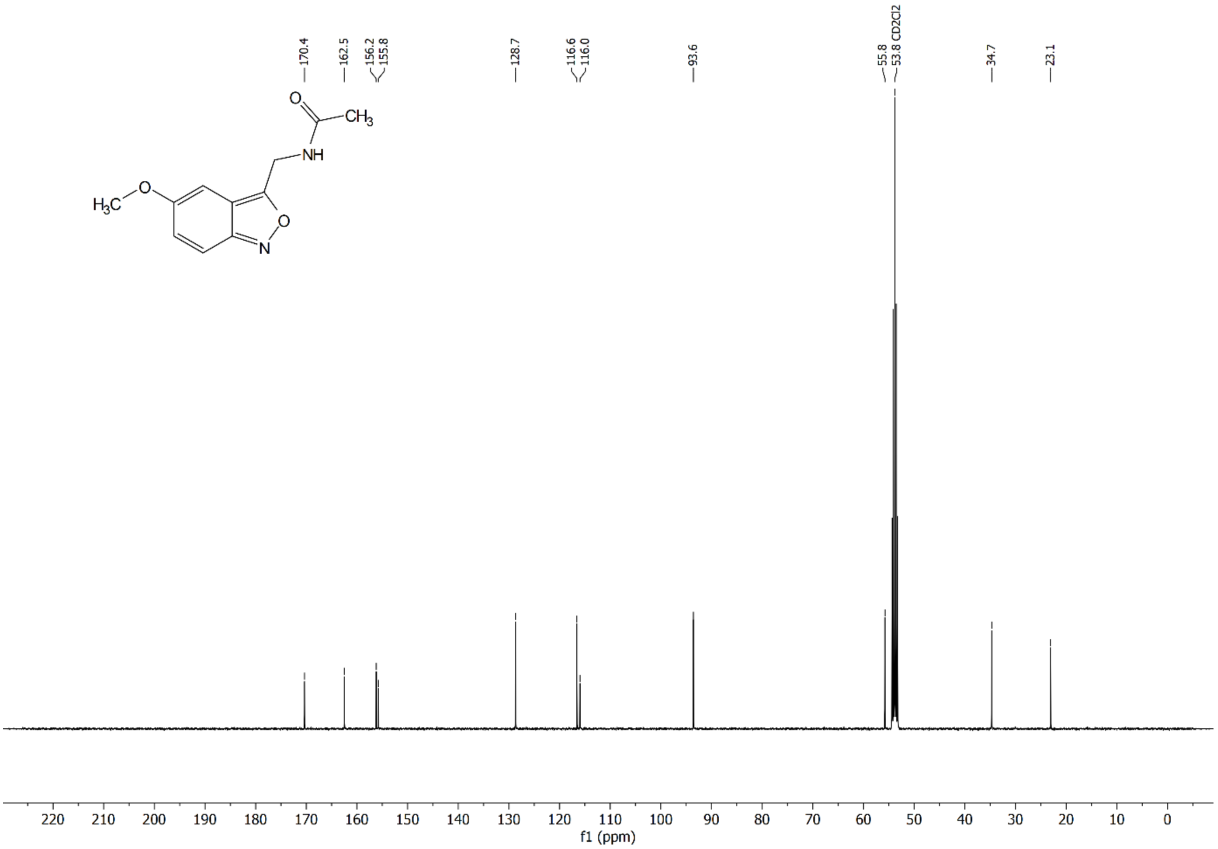

^{1}H NMR spectrum (400 MHz, CD_2Cl_2) of **5y**.

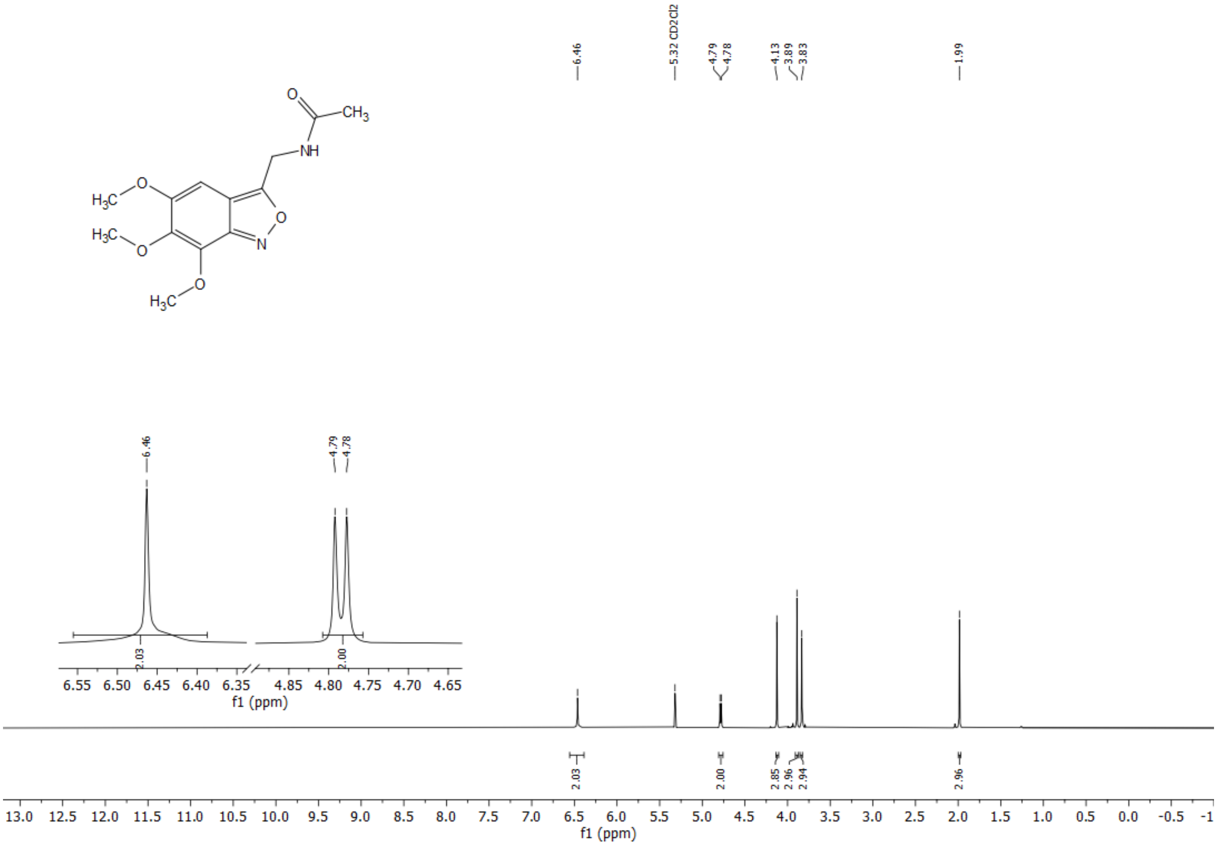

^{13}C NMR spectrum (101 MHz, CD_2Cl_2) of **5y**.

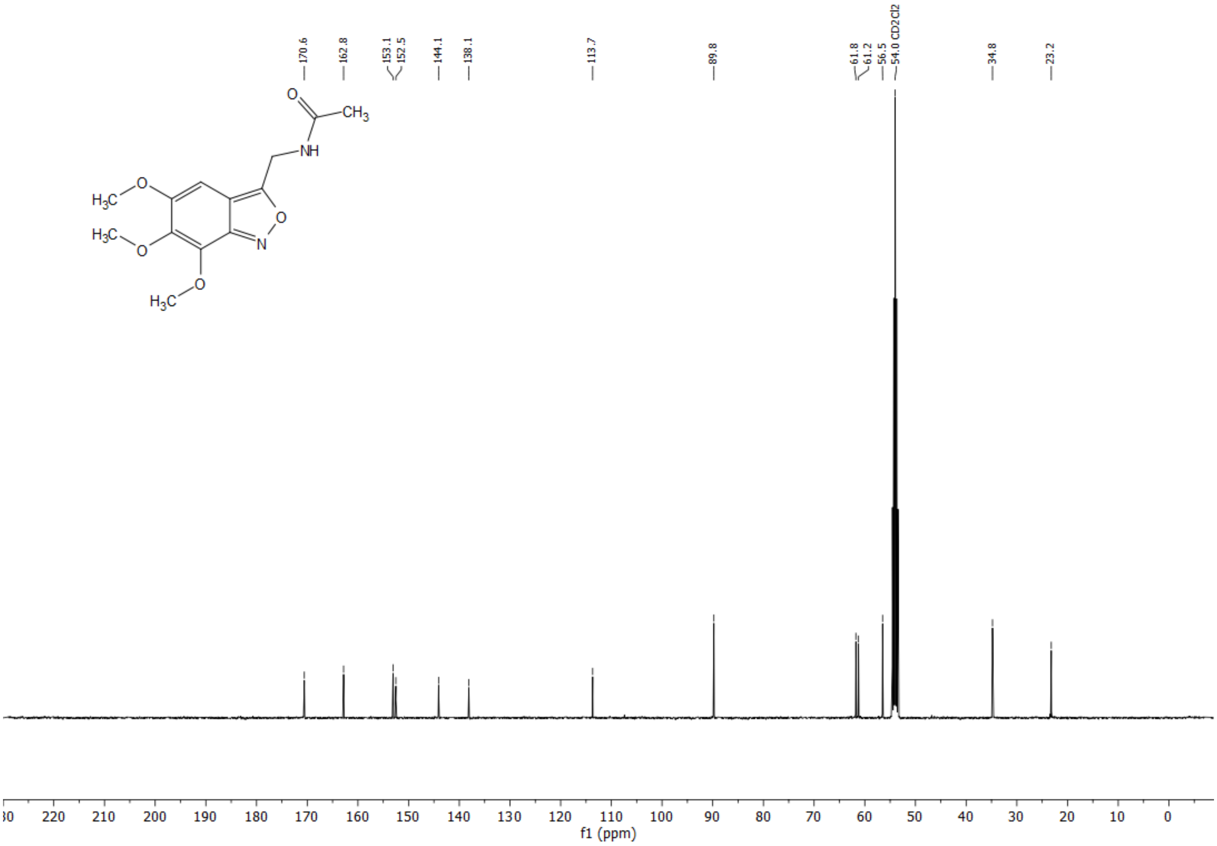

^{1}H NMR spectrum (400 MHz, CD_2Cl_2) of **5z**.

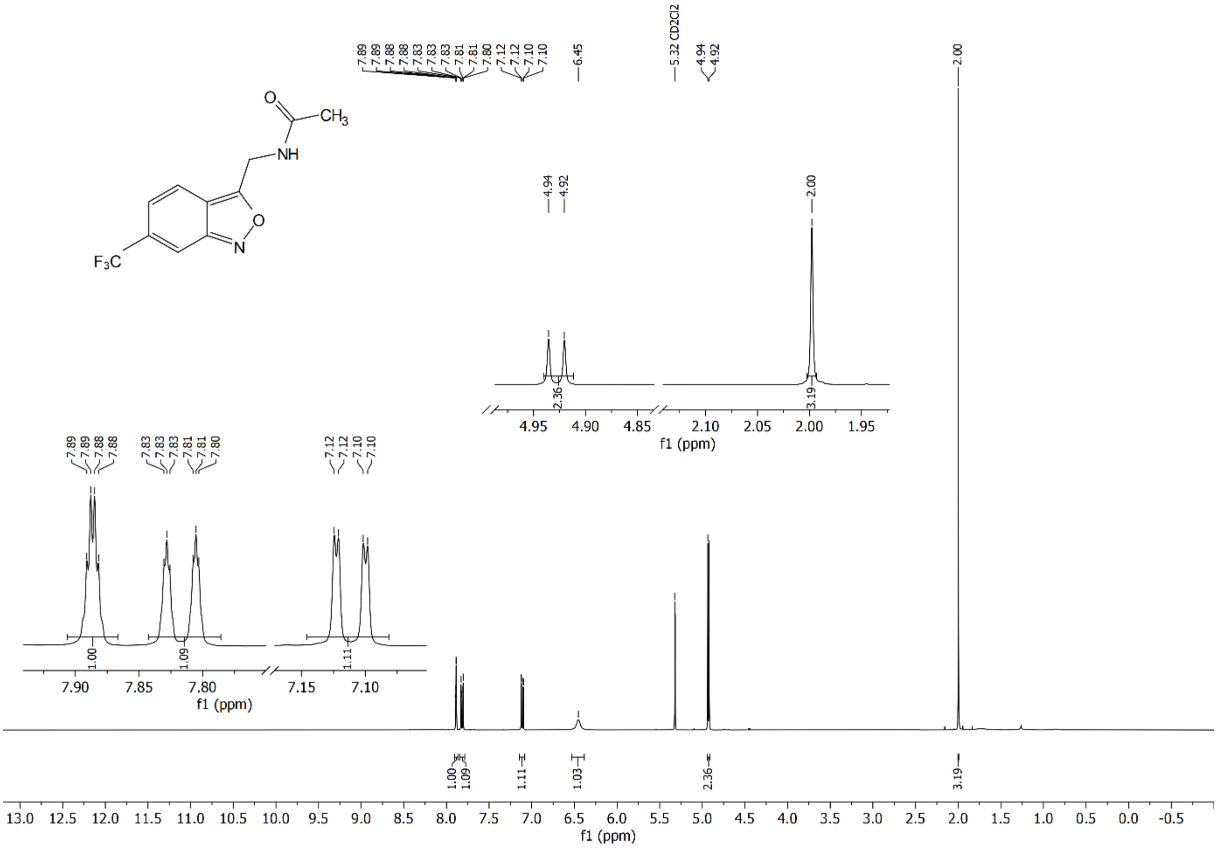

^{13}C NMR spectrum (101 MHz, CD_2Cl_2) of **5z**.

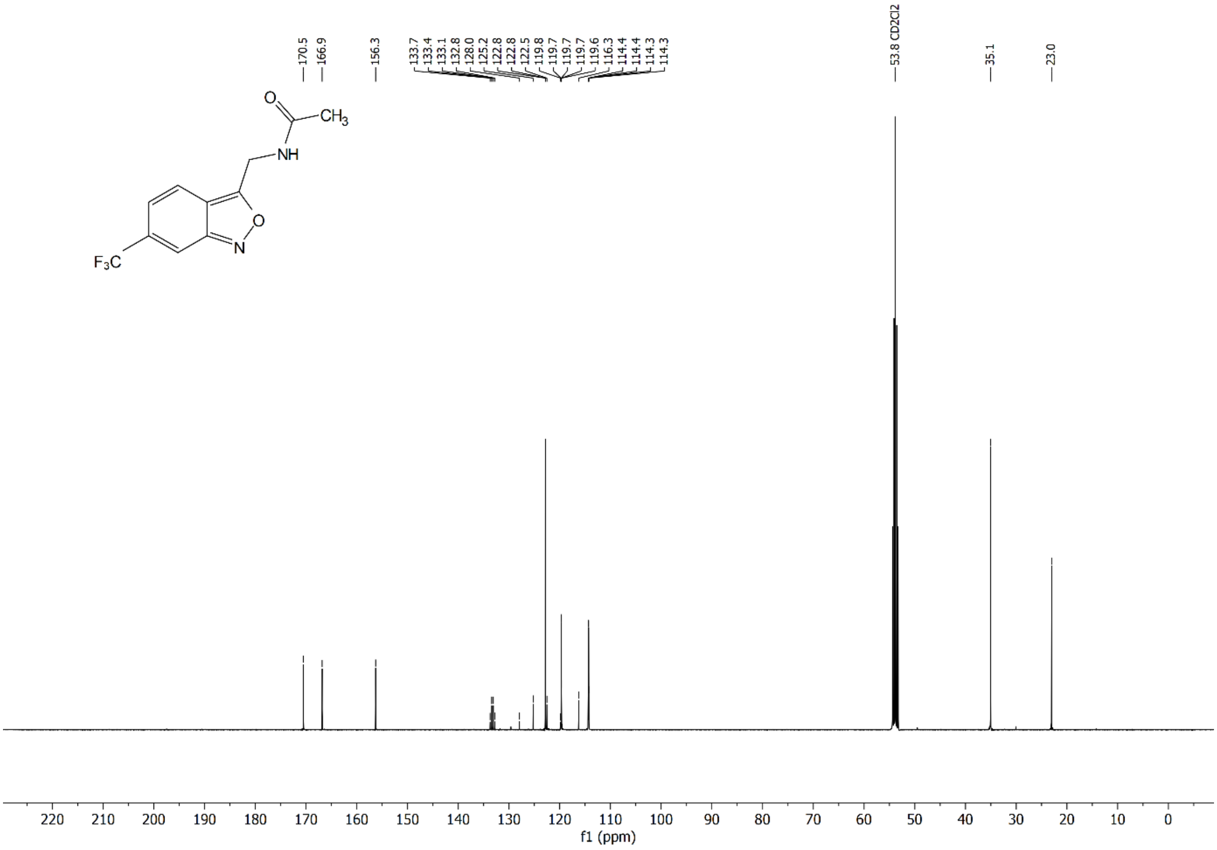

^{19}F NMR spectrum (376 MHz, CD_2Cl_2) of **5z**.

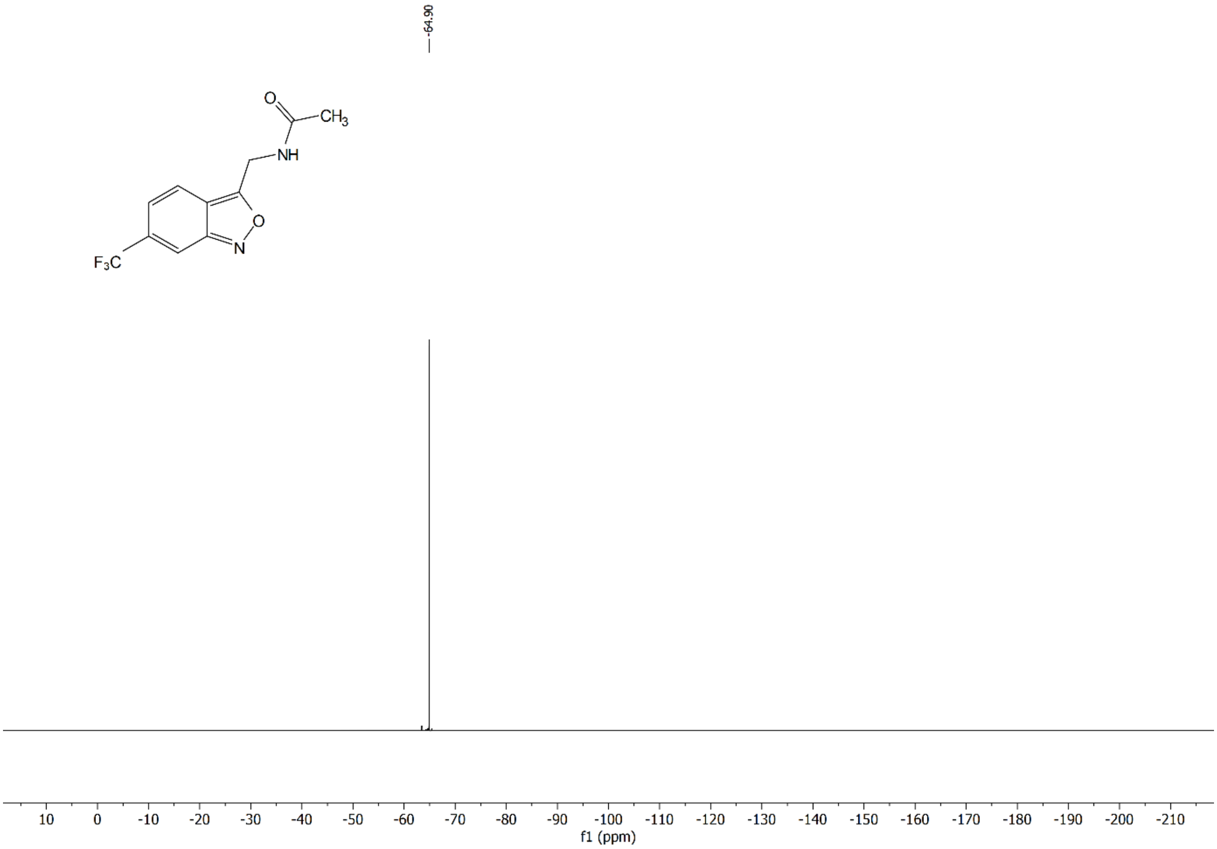

^{1}H NMR spectrum (400 MHz, CD_2Cl_2) of **5aa**.

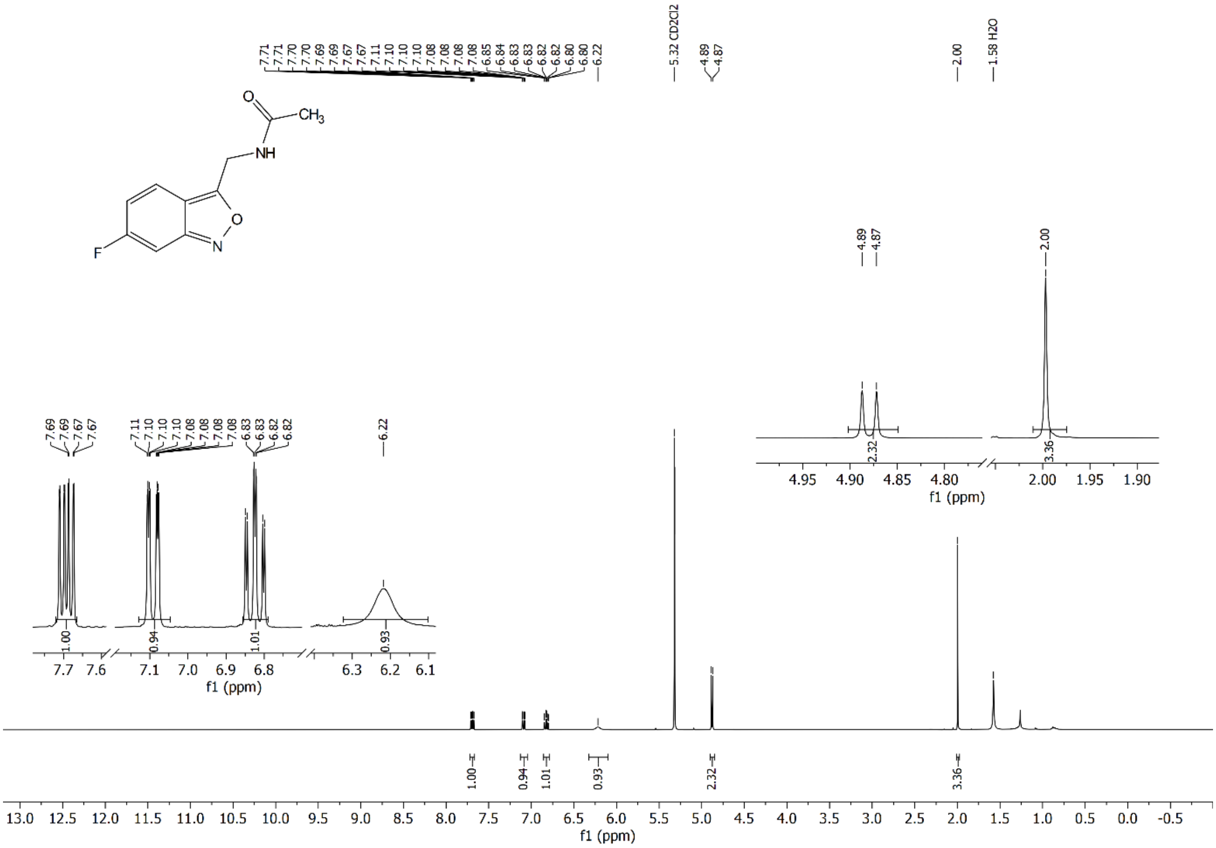

^{13}C NMR spectrum (101 MHz, CD_2Cl_2) of **5aa**.

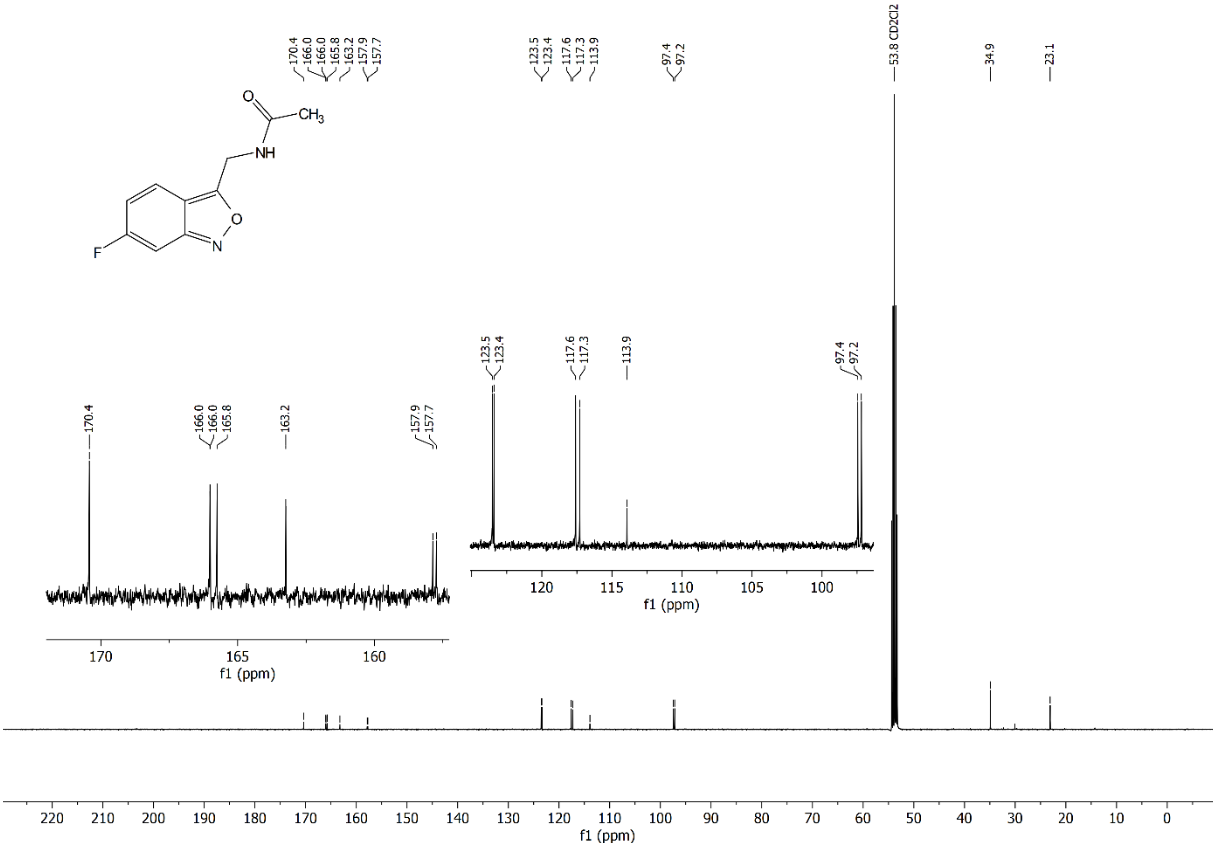

^{19}F NMR spectrum (376 MHz, CD_2Cl_2) of **5aa**.

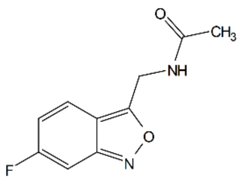

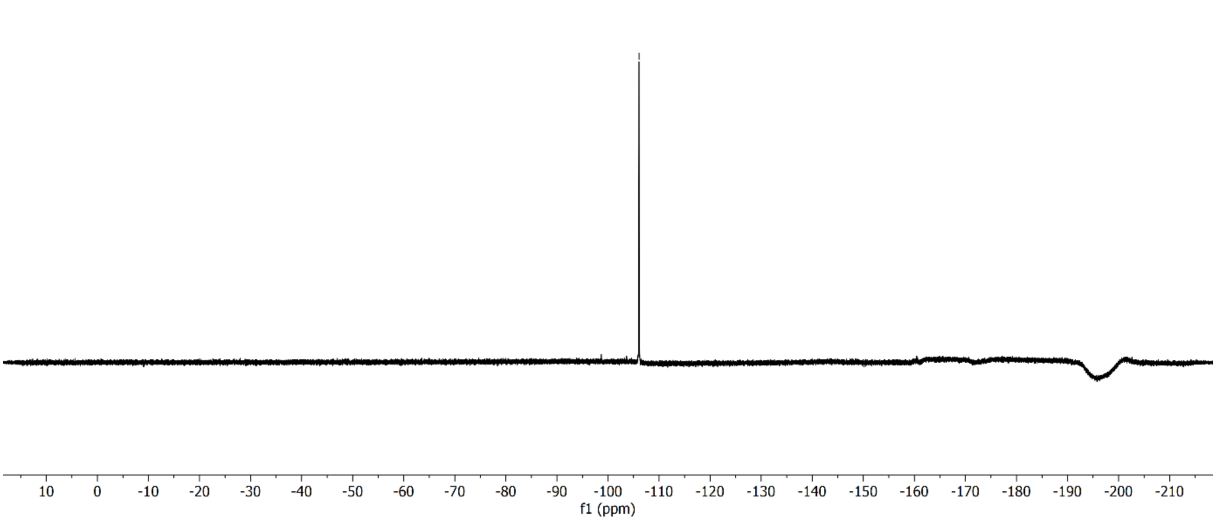

^{1}H NMR spectrum (400 MHz, CD_3CN) of **5ab**.

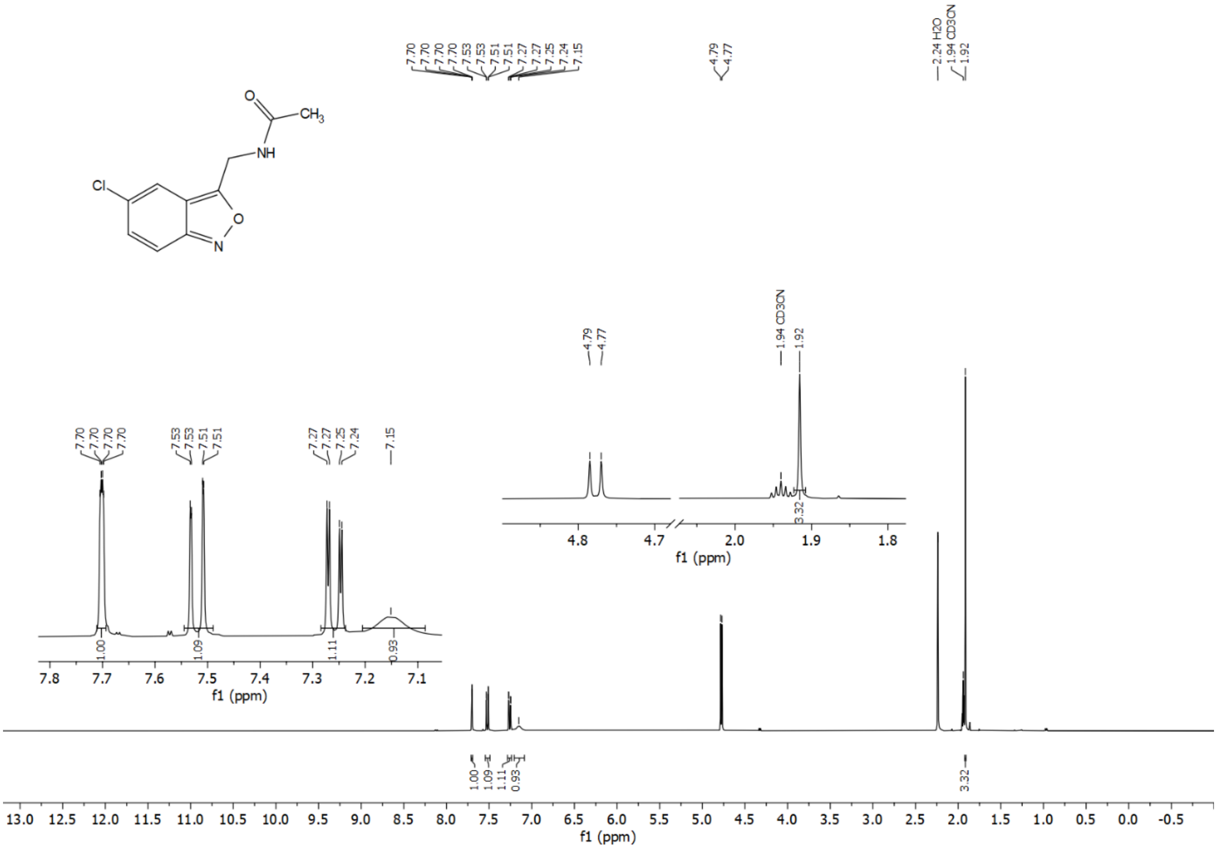

^{13}C NMR spectrum (101 MHz, CD_3CN) of **5ab**.

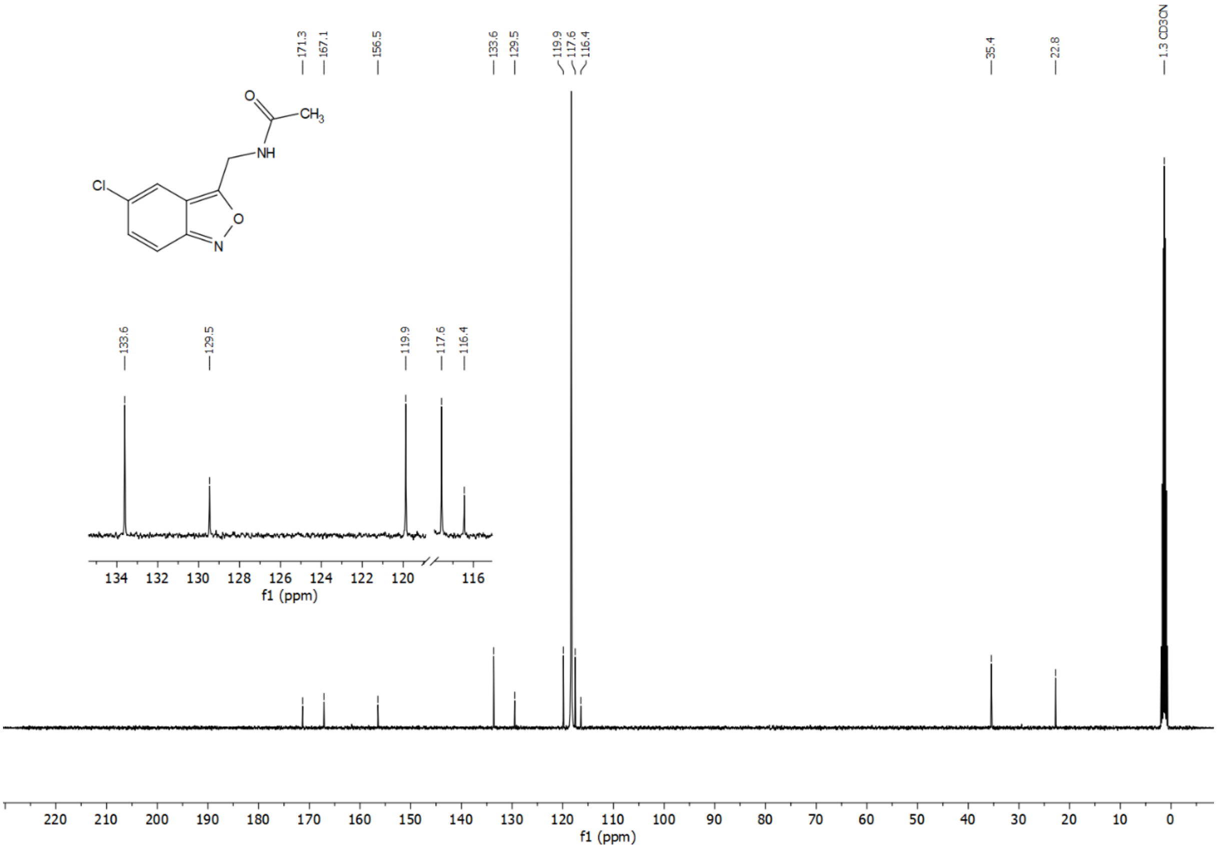

^{1}H NMR spectrum (400 MHz, CD_2Cl_2) of **5ac**.

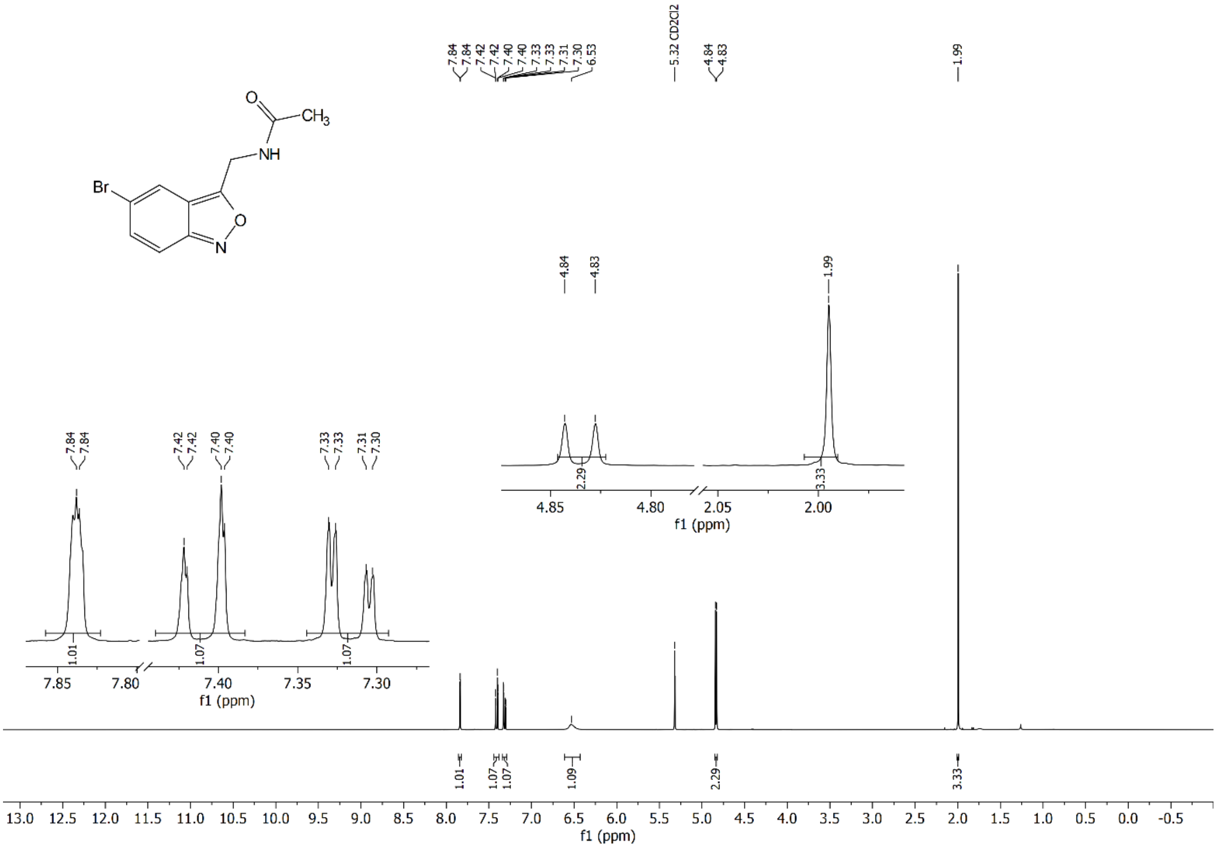

^{13}C NMR spectrum (101 MHz, CD_2Cl_2) of **5ac**.

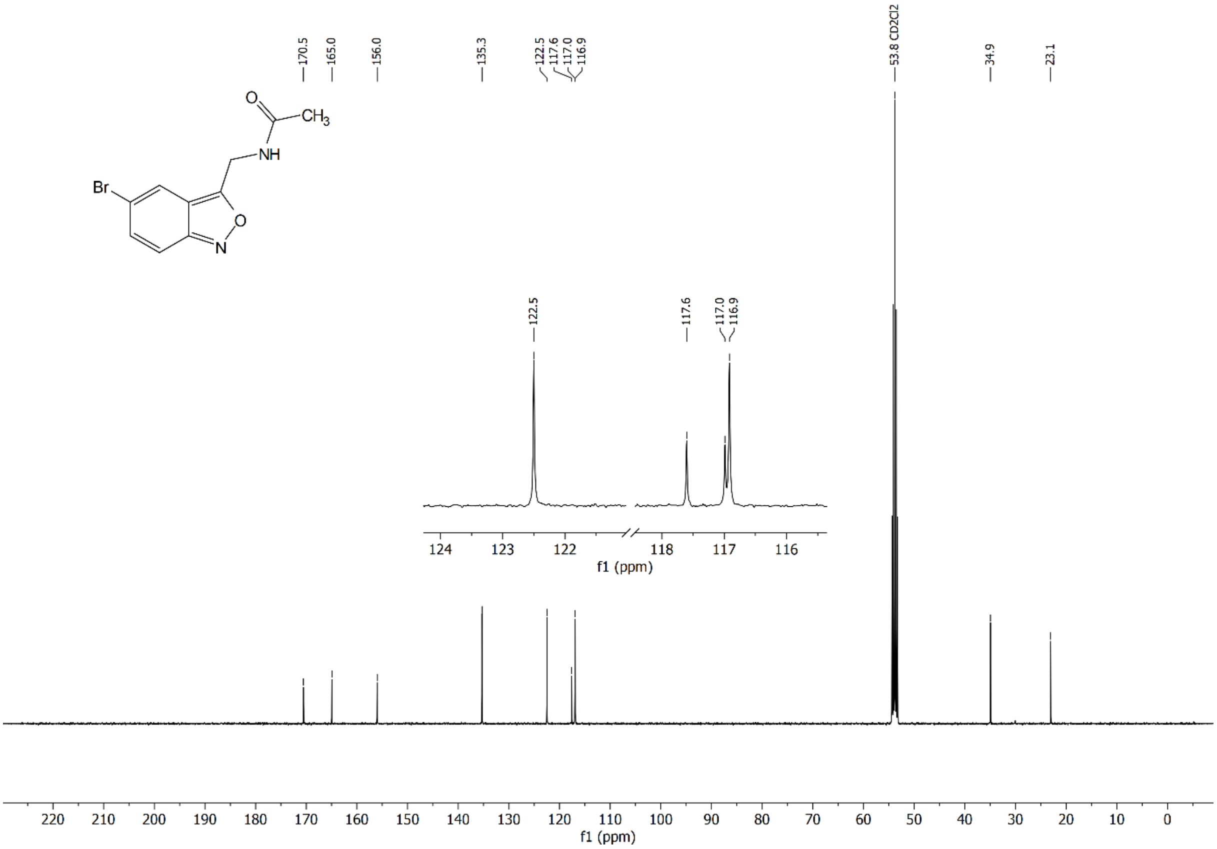

^{1}H NMR spectrum (400 MHz, CD_2Cl_2) of **5ad**.

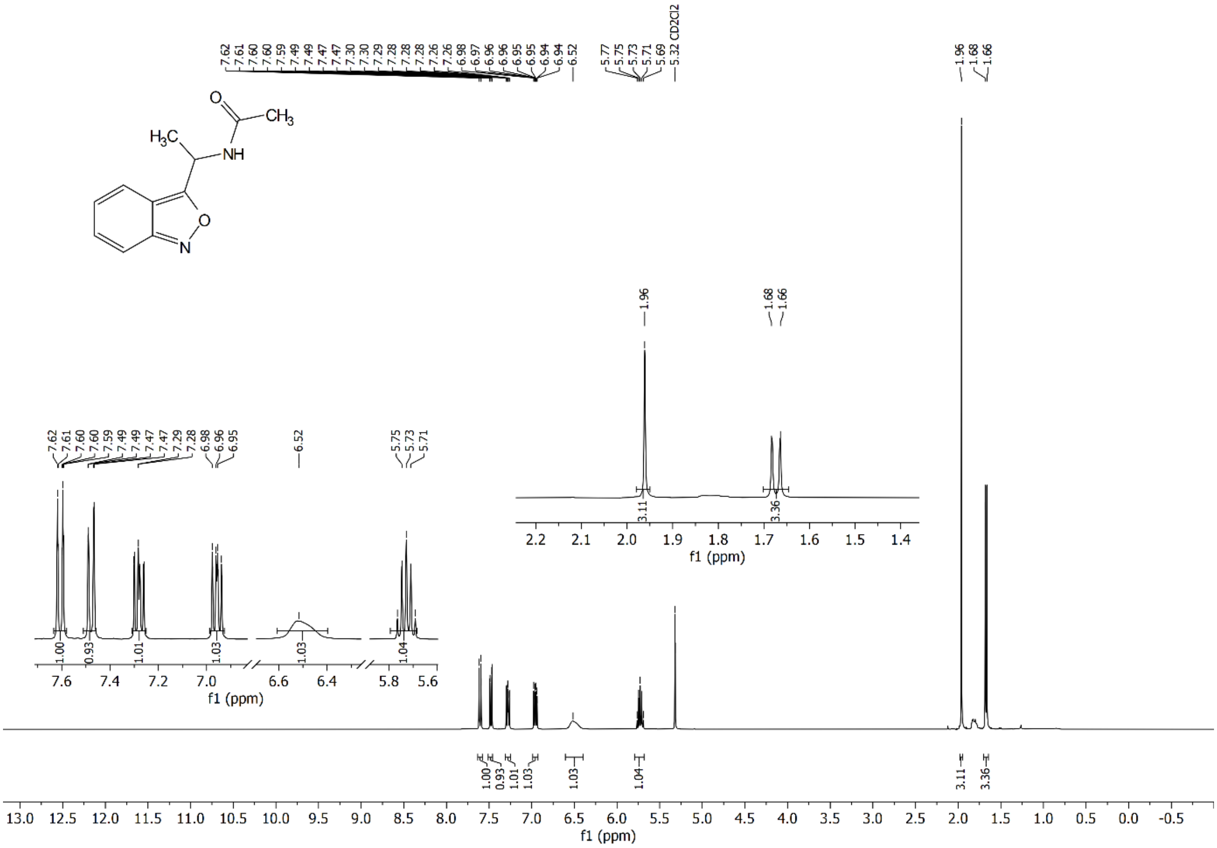

^{13}C NMR spectrum (101 MHz, CD_2Cl_2) of **5ad**.

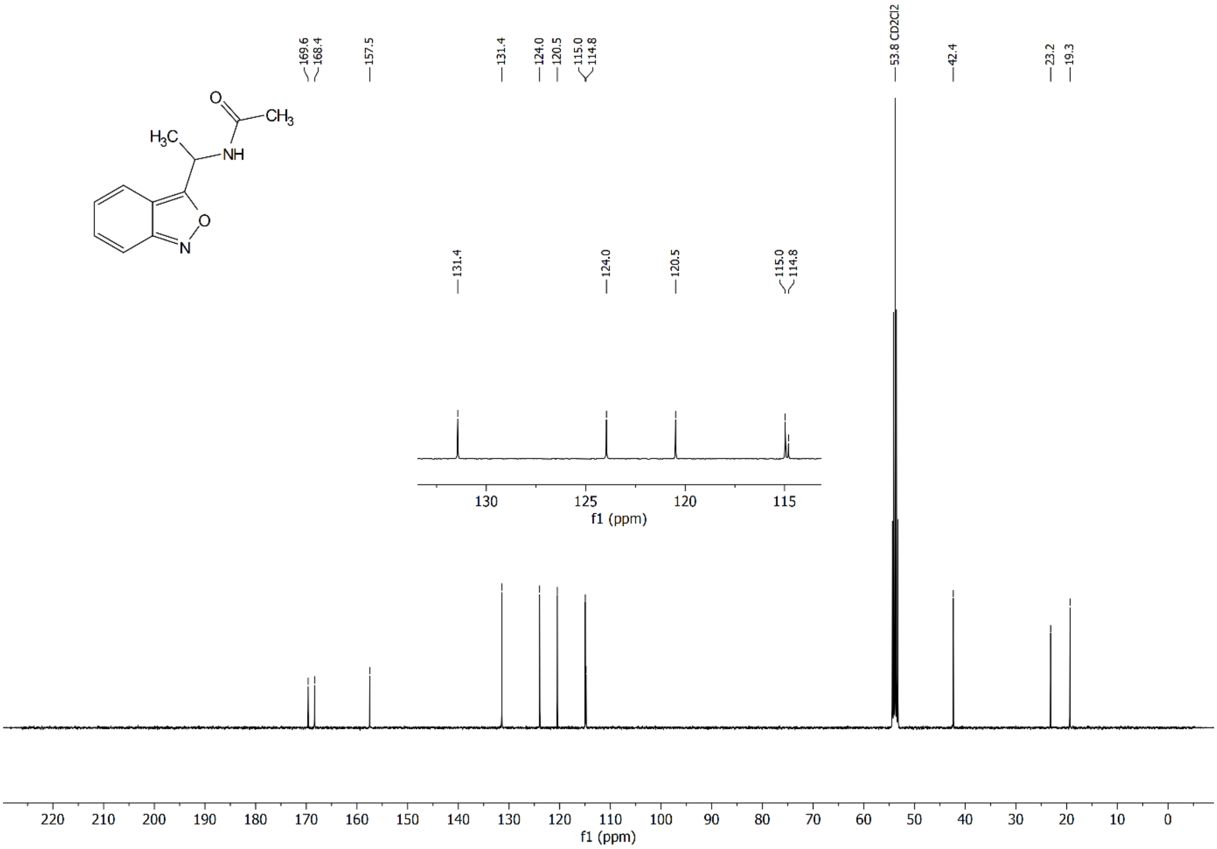

^{1}H NMR spectrum (400 MHz, CD_2Cl_2) of **5ae**.

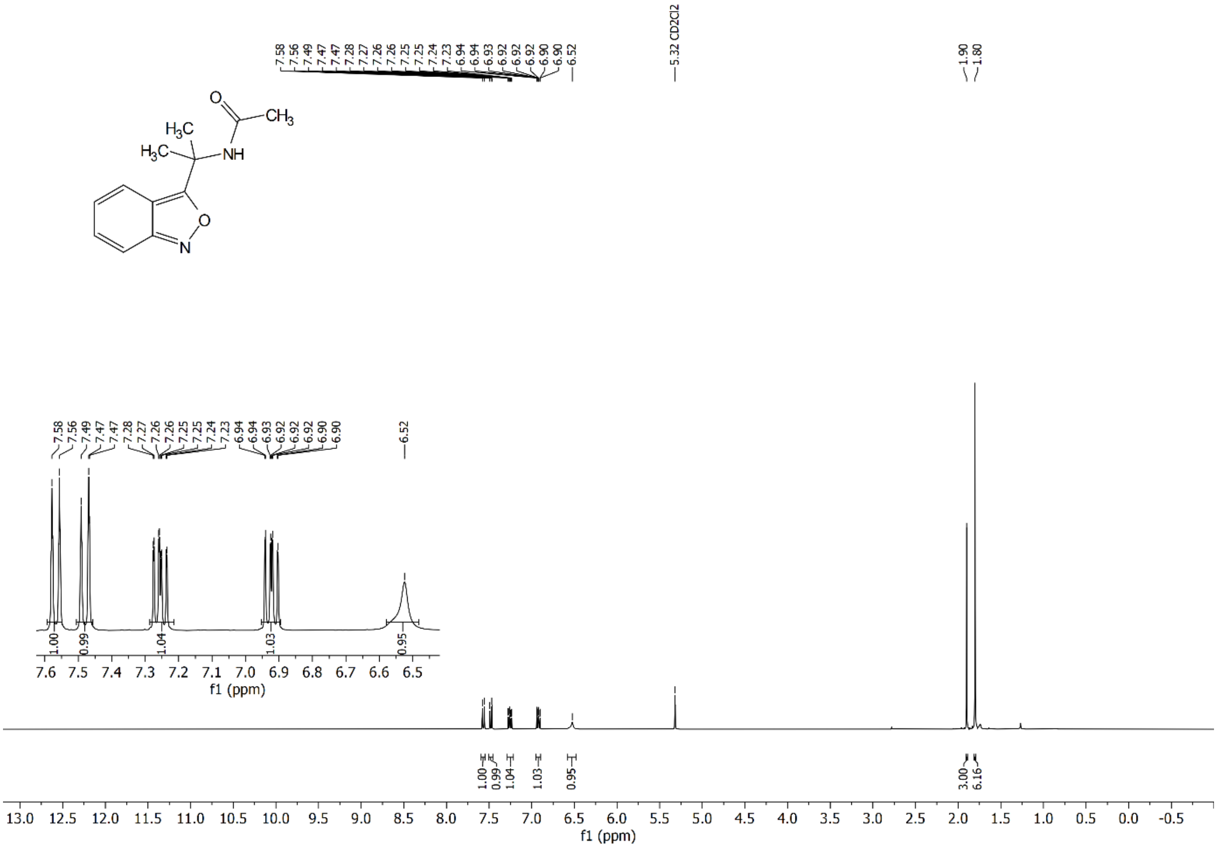

^{13}C NMR spectrum (101 MHz, CD_2Cl_2) of **5ae**.

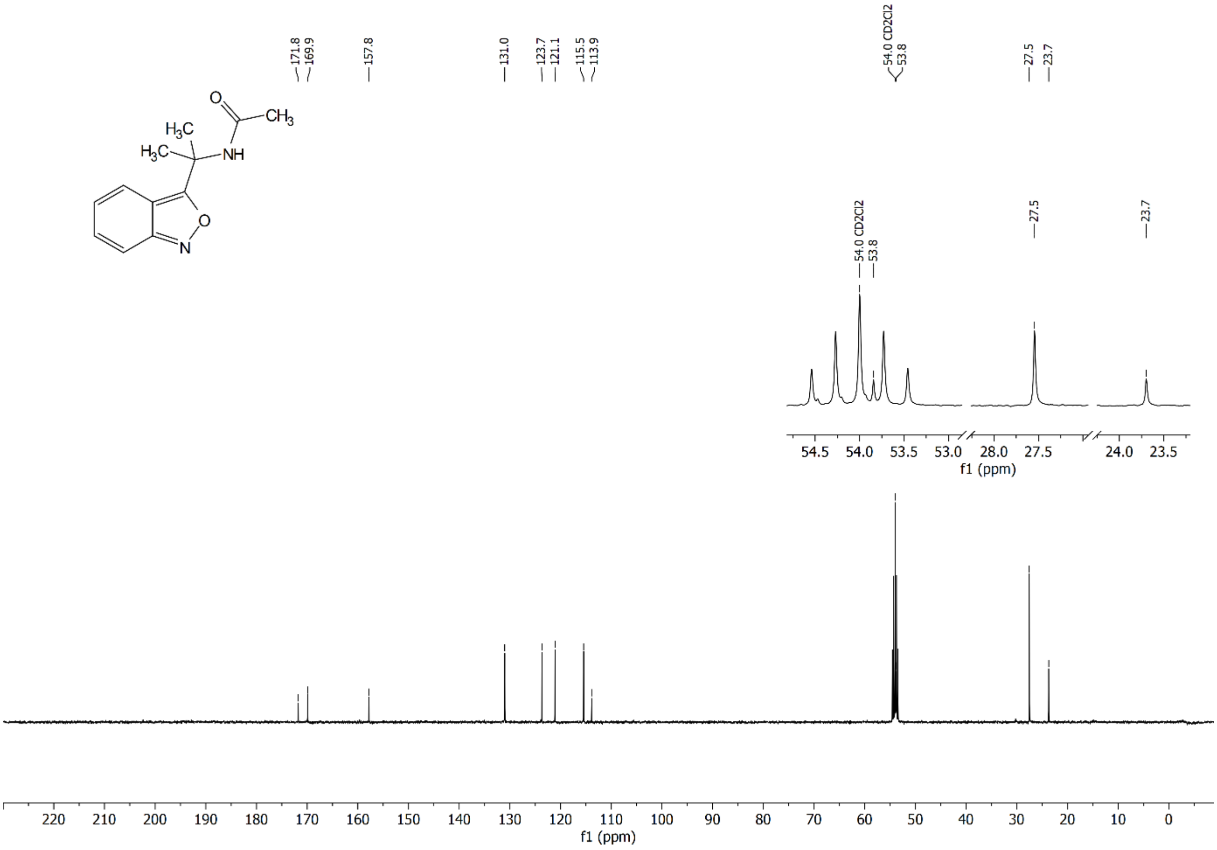

^{1}H NMR spectrum (400 MHz, CD_2Cl_2) of **5af**.

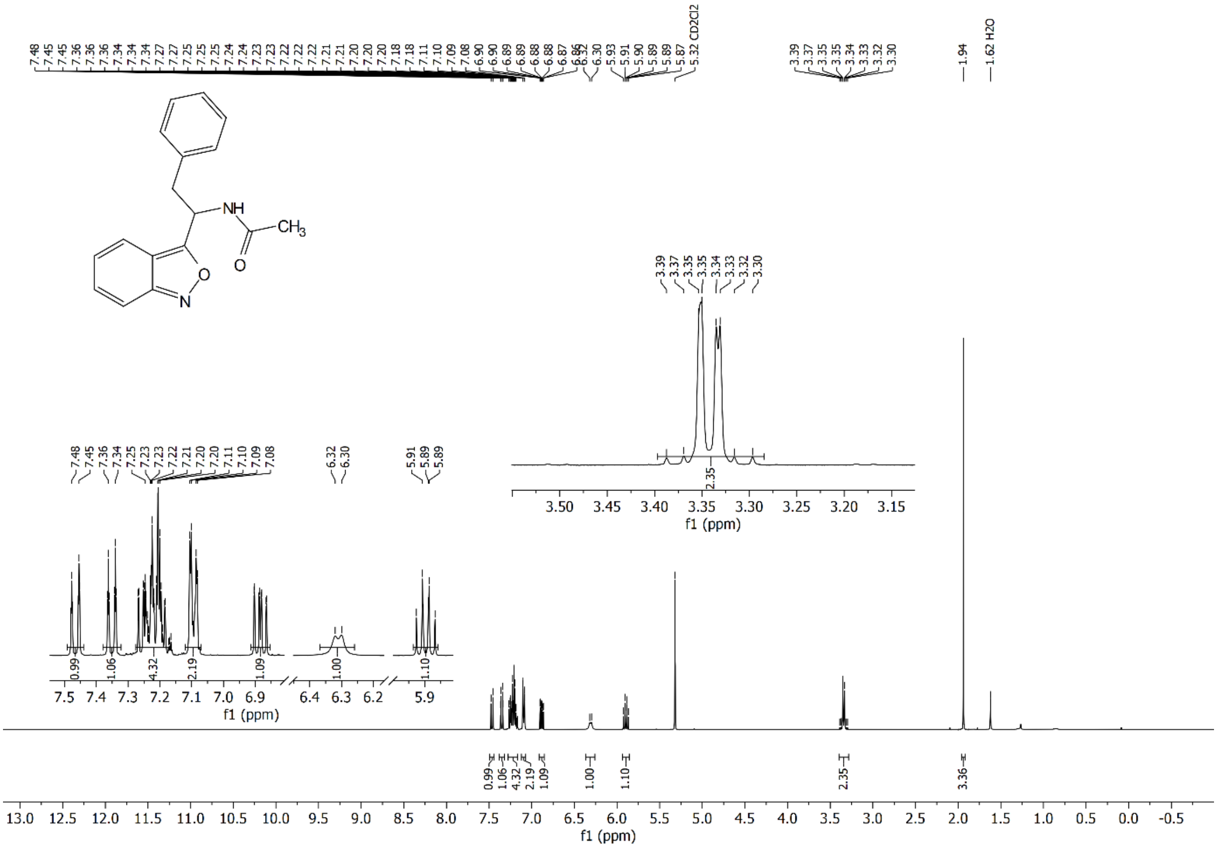

^{13}C NMR spectrum (101 MHz, CD_2Cl_2) of **5af**.

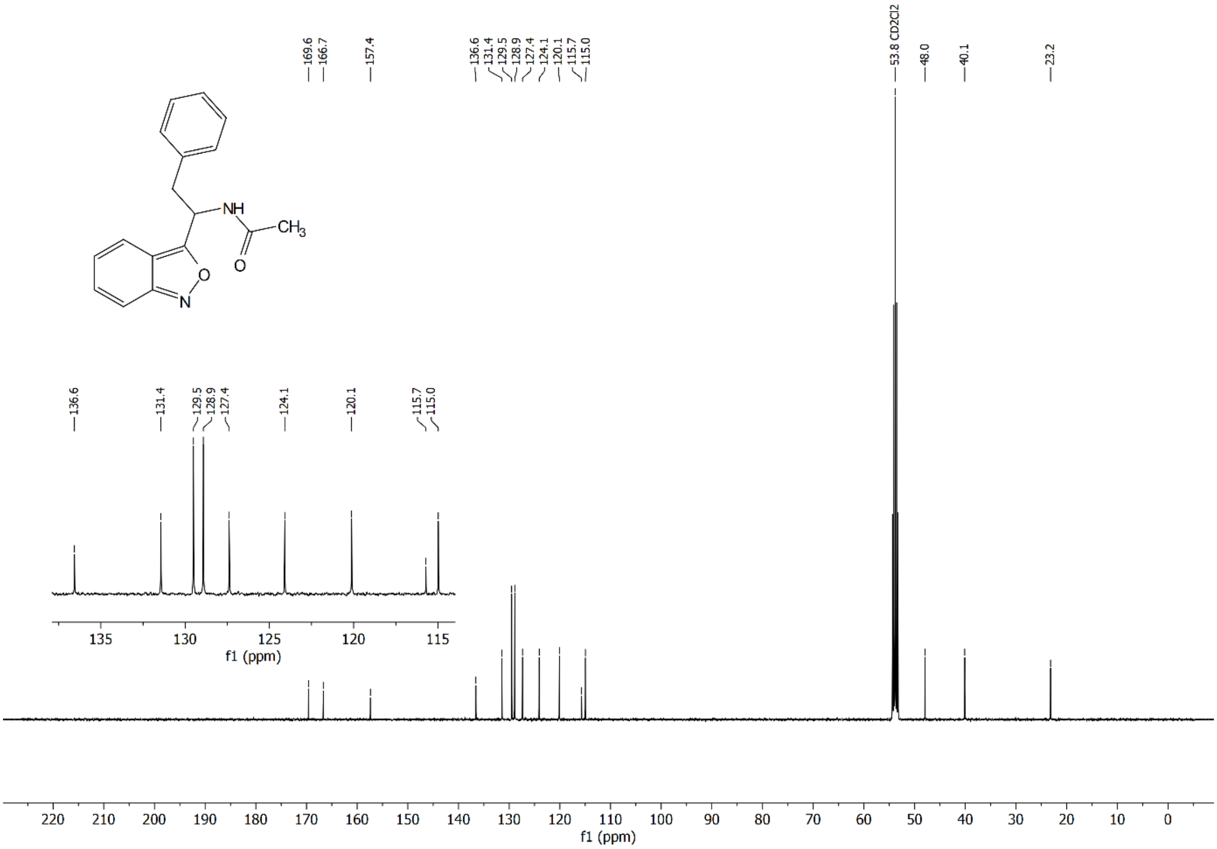

^{1}H NMR spectrum (400 MHz, CD_2Cl_2) of **5ag**.

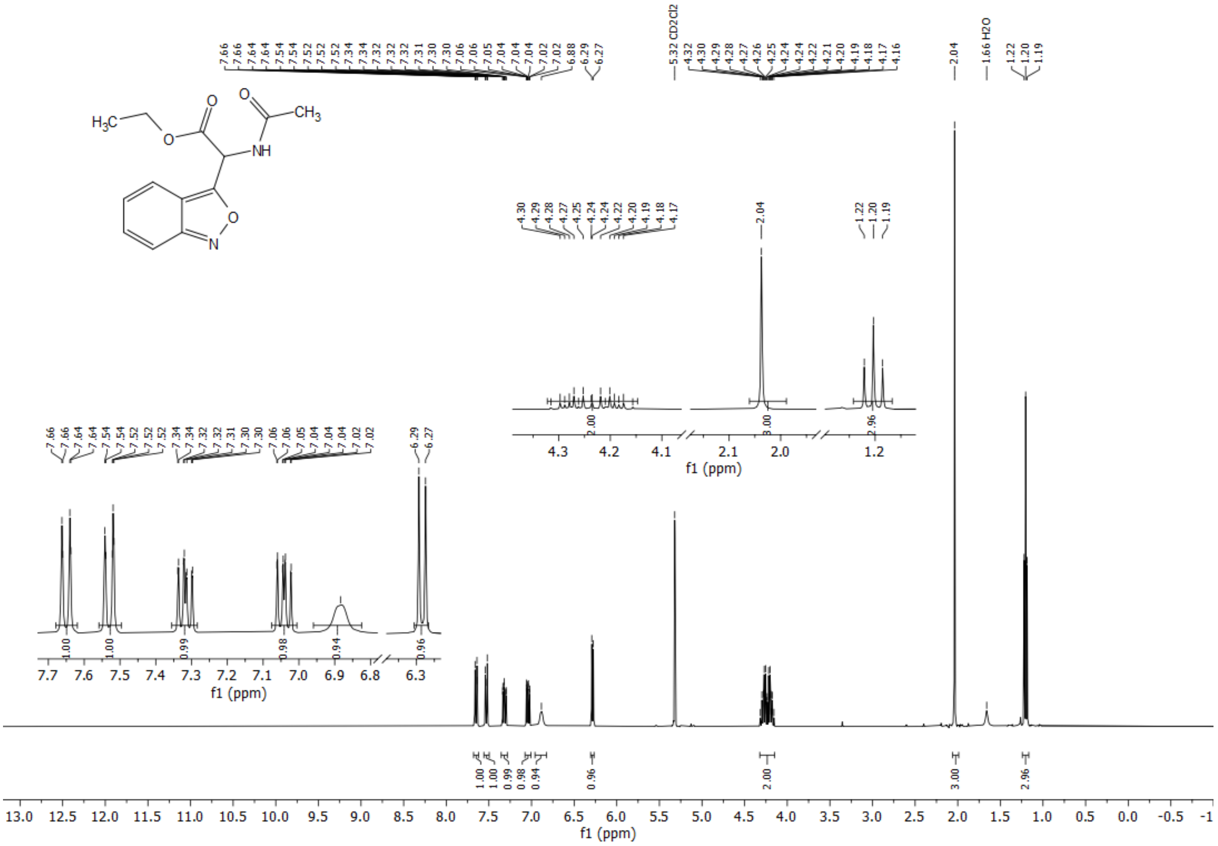

^{13}C NMR spectrum (101 MHz, CD_2Cl_2) of **5ag**.

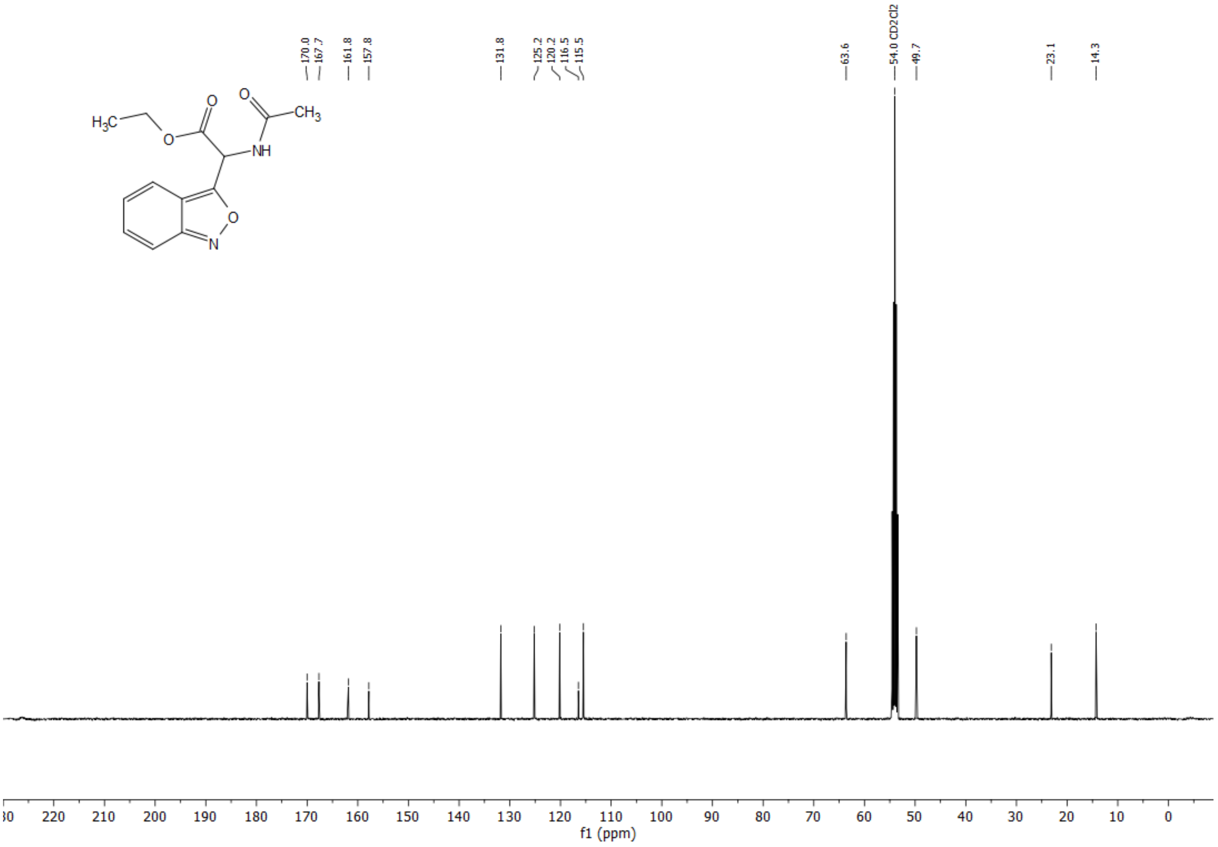

^{1}H NMR spectrum (400 MHz, CD_2Cl_2) of **5ah**.

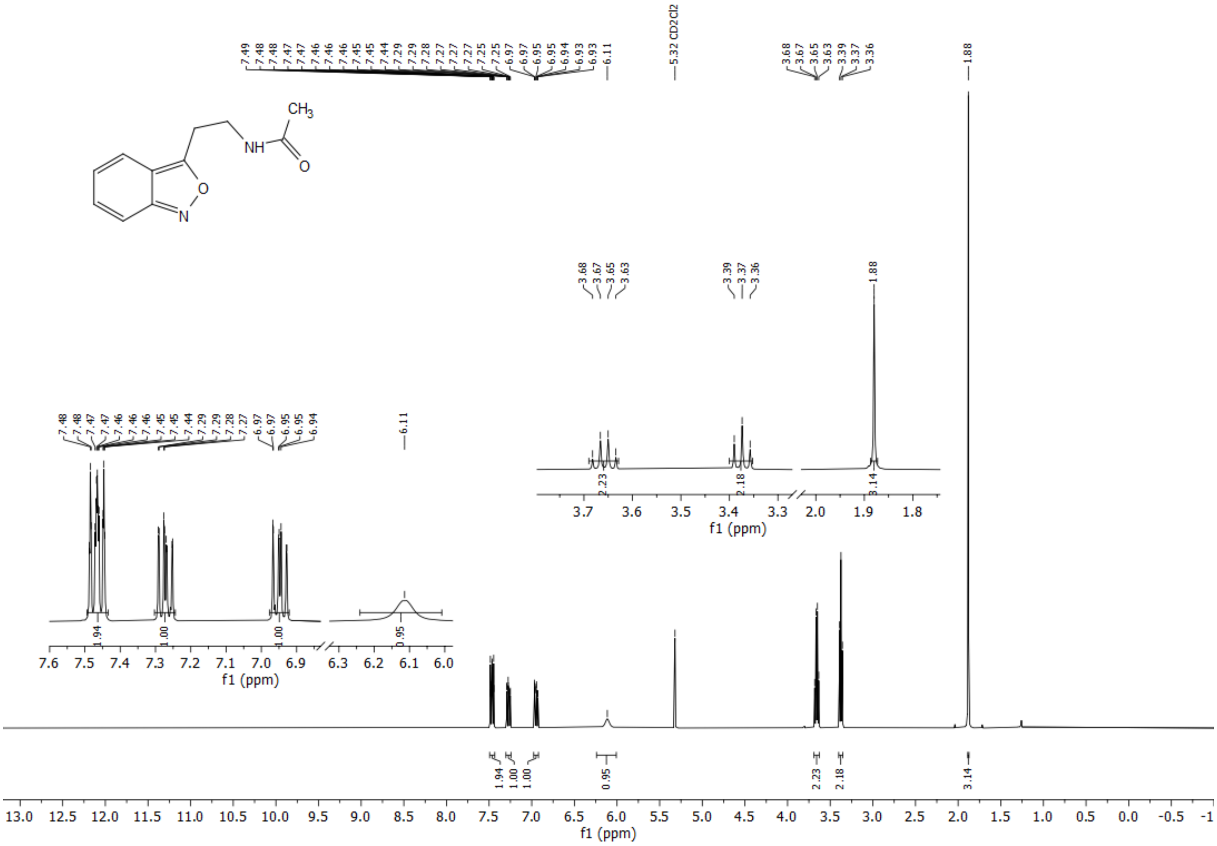

^{13}C NMR spectrum (101 MHz, CD_2Cl_2) of **5ah**.

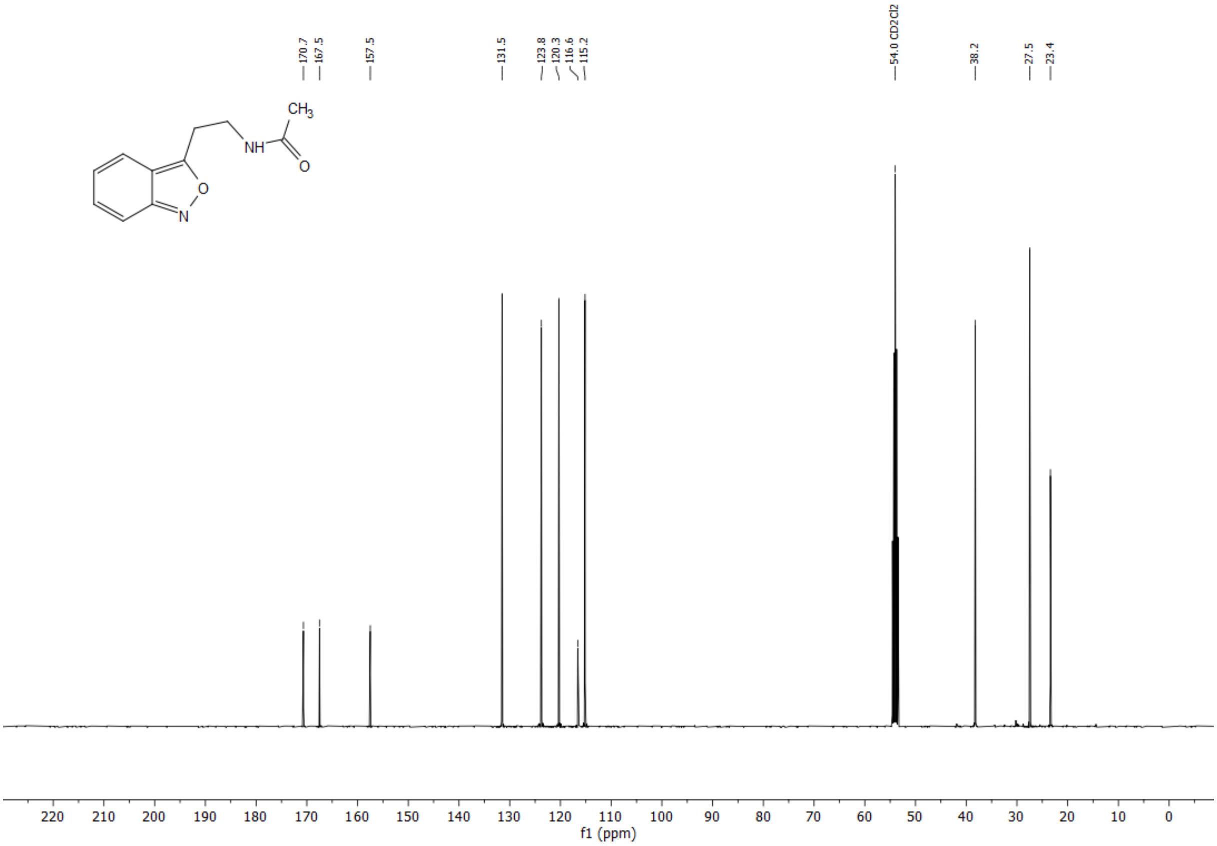

^{1}H NMR spectrum (400 MHz, CD_2Cl_2) of **5ai**.

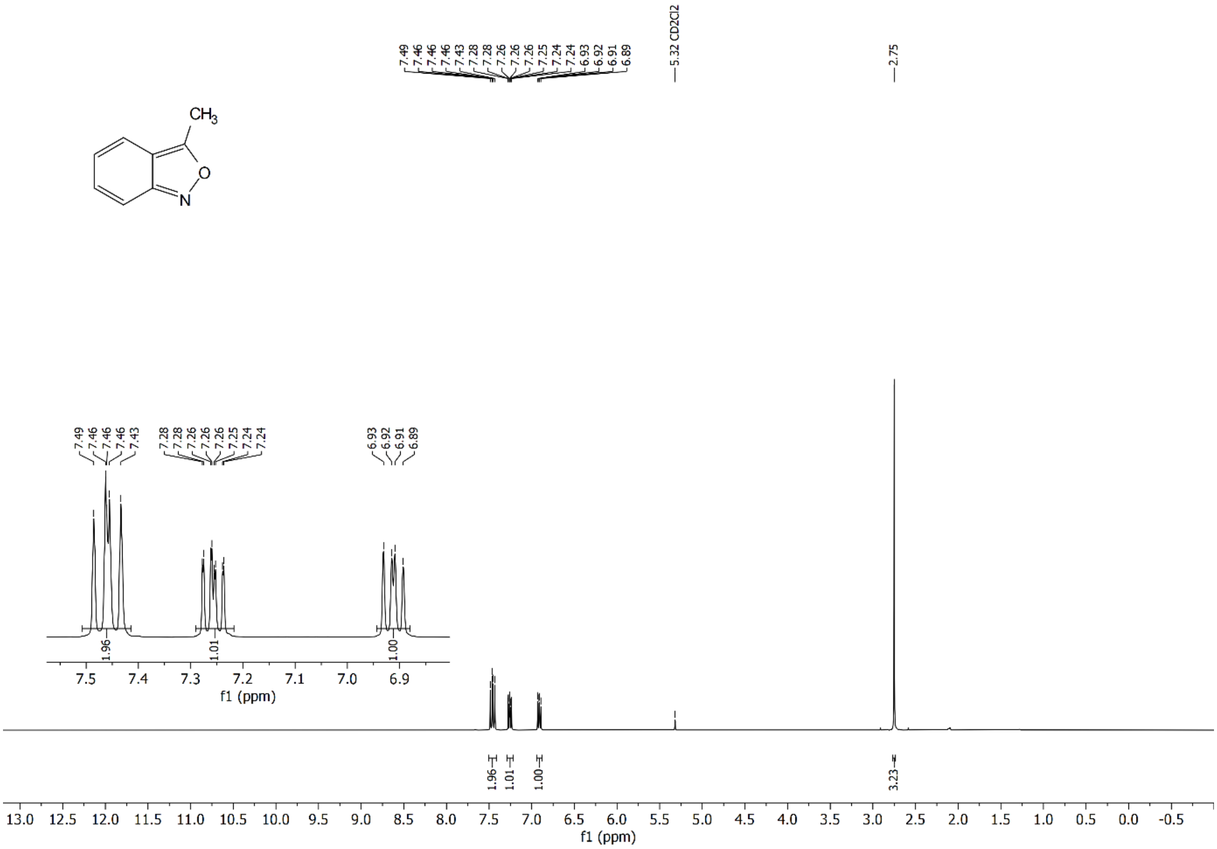

^{13}C NMR spectrum (101 MHz, CD_2Cl_2) of **5ai**.

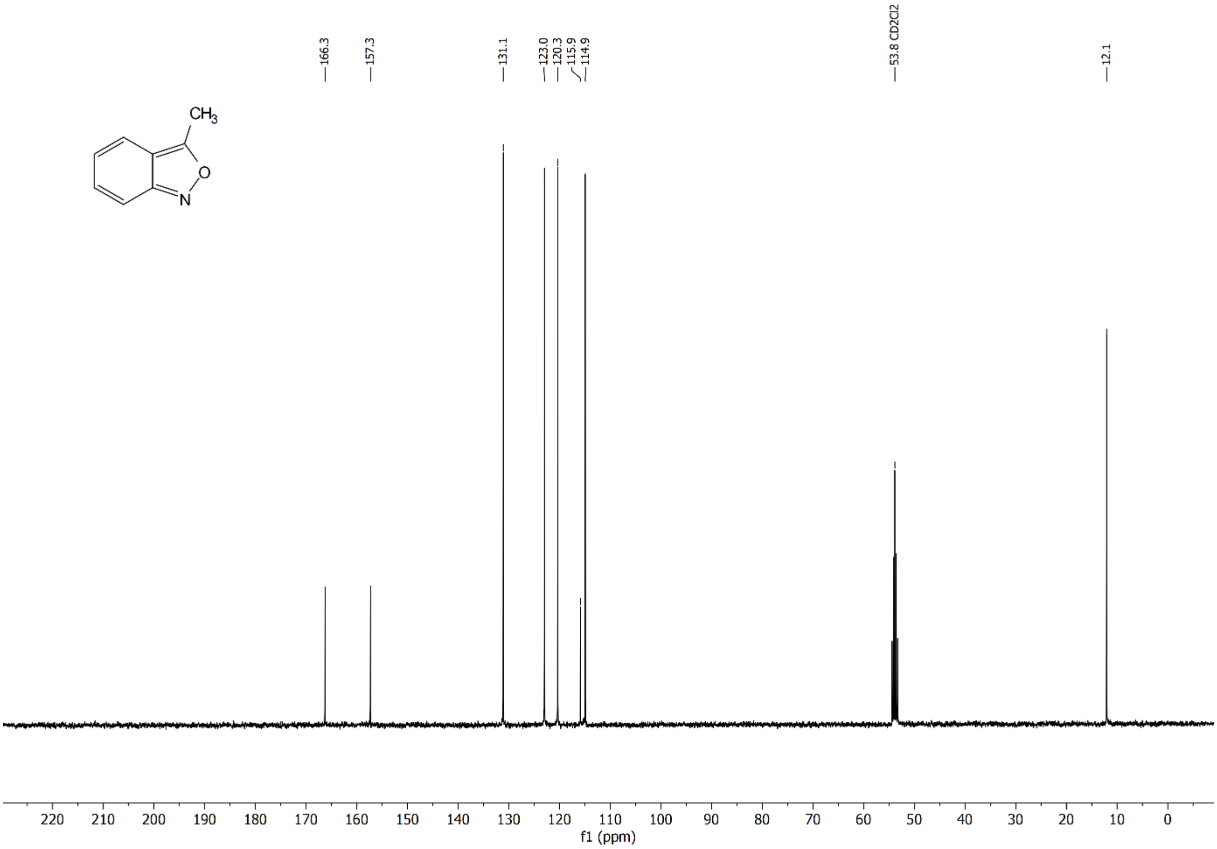

^1H NMR spectrum (400 MHz, CD_2Cl_2) of **5aj**.

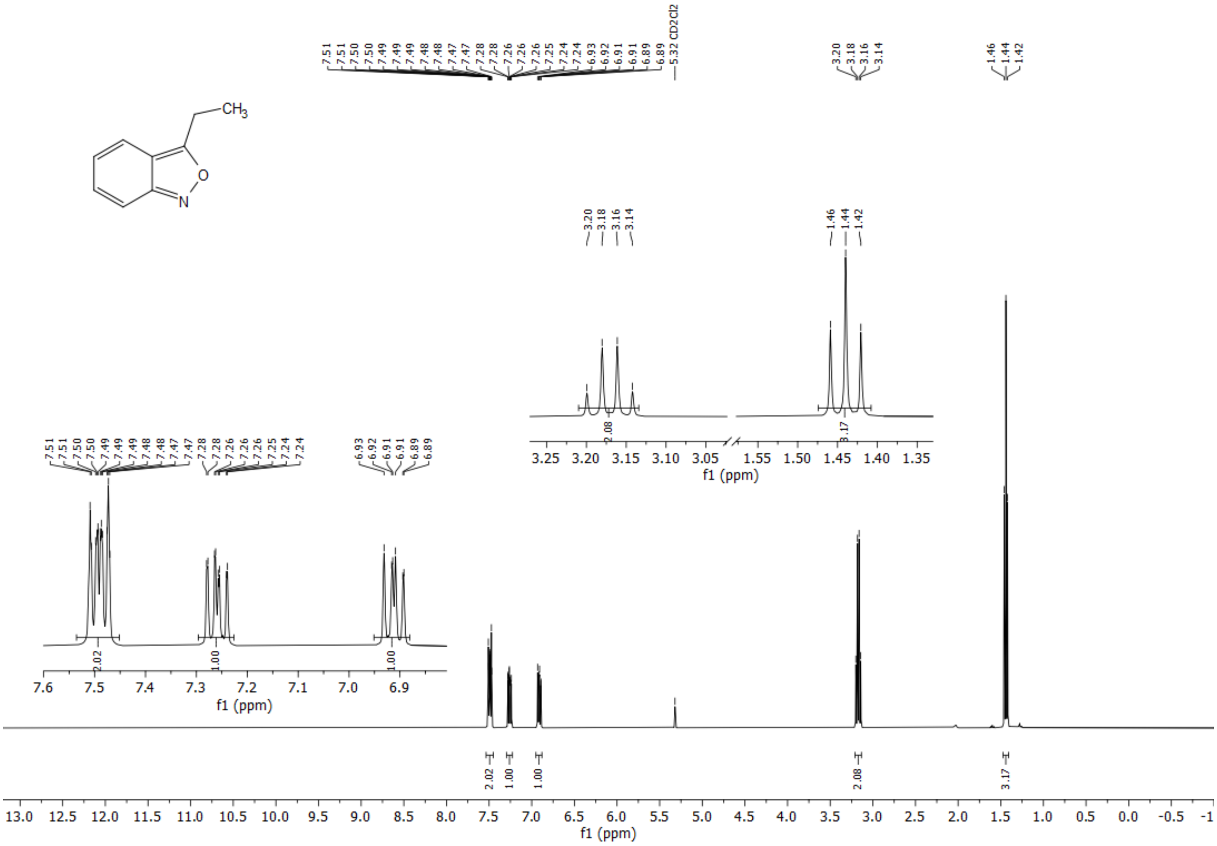

^{13}C NMR spectrum (101 MHz, CD_2Cl_2) of **5aj**.

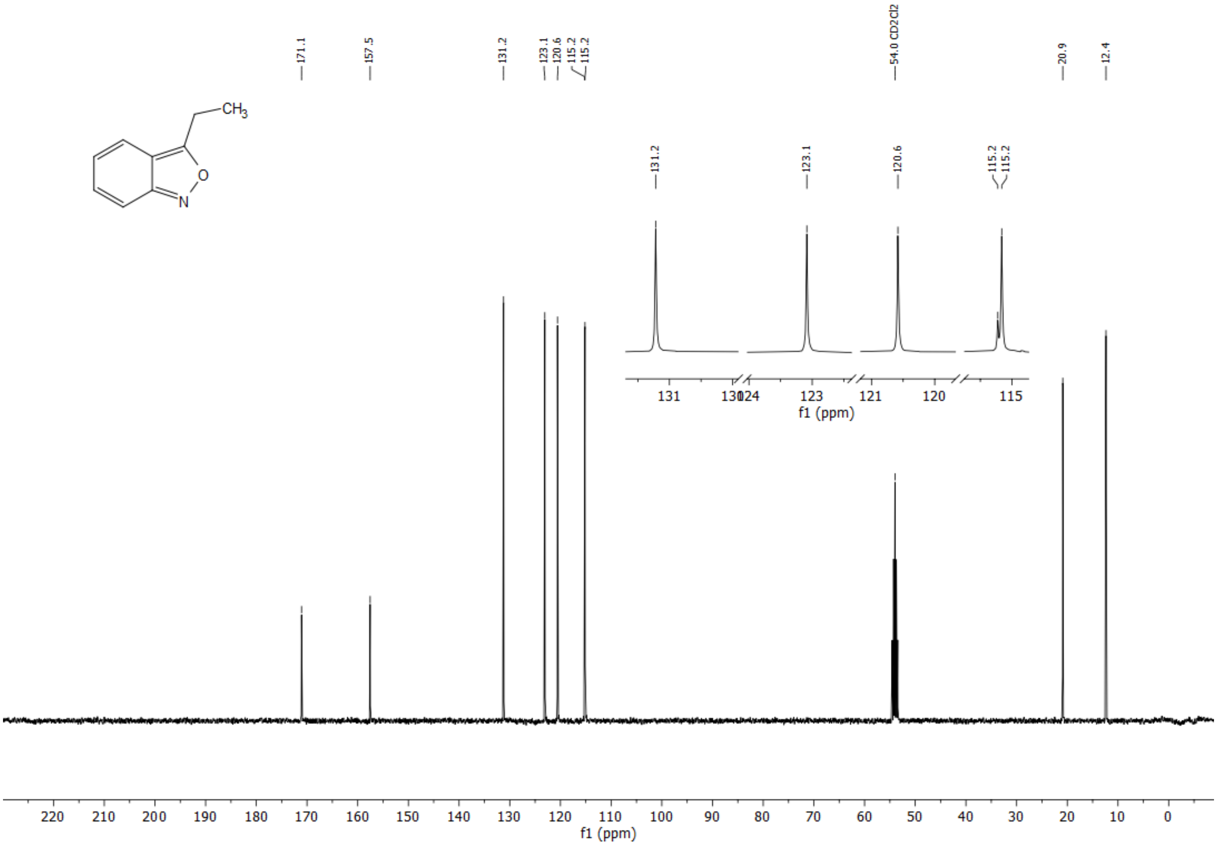

^{1}H NMR spectrum (400 MHz, CD_2Cl_2) of **5ak**.

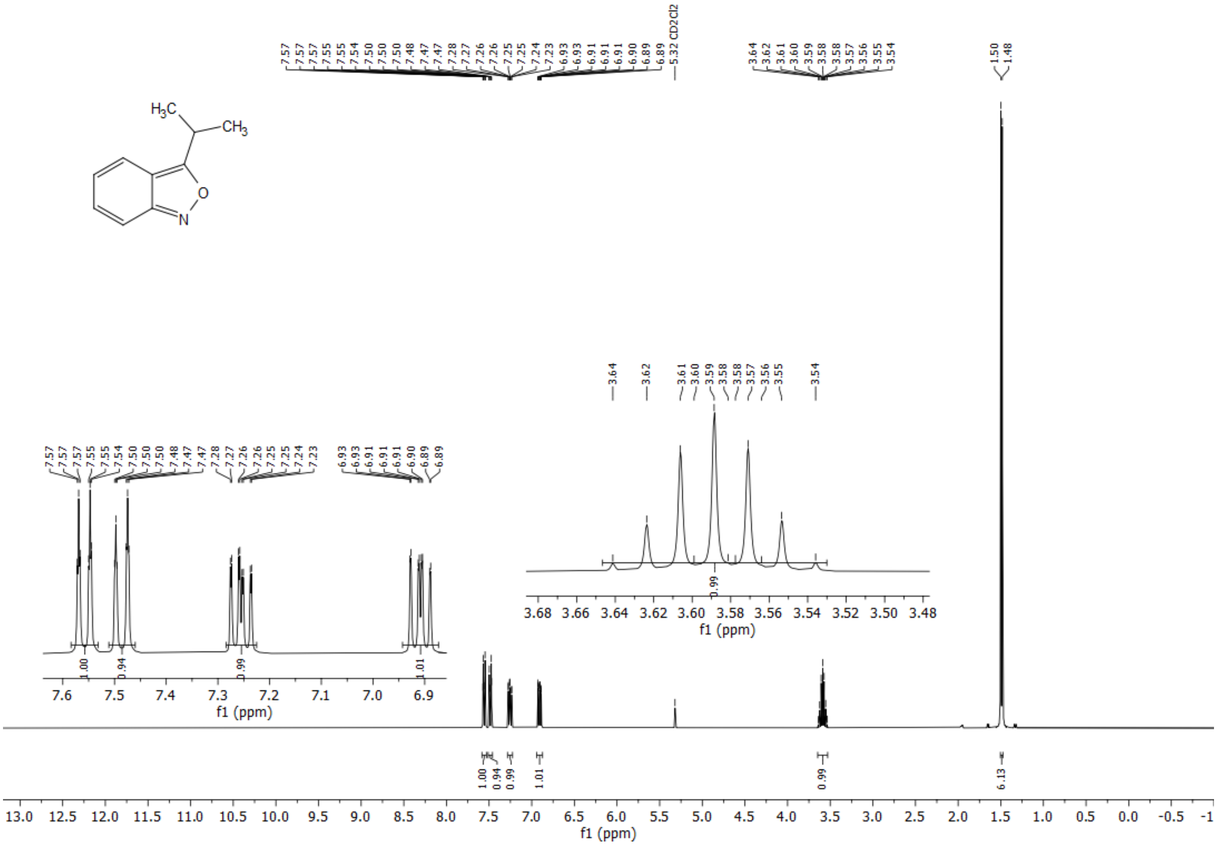

^{13}C NMR spectrum (101 MHz, CD_2Cl_2) of **5ak**.

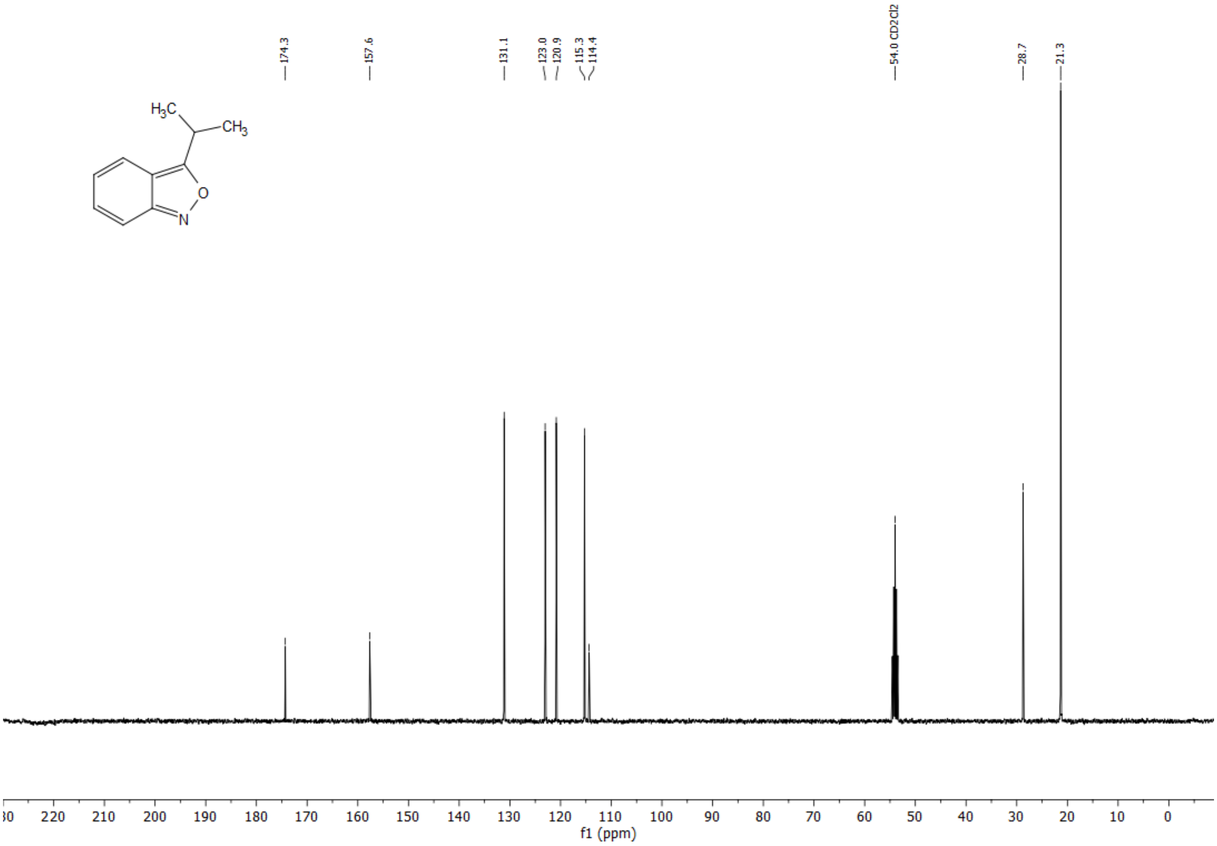

^{1}H NMR spectrum (400 MHz, CD_2Cl_2) of **5al**.

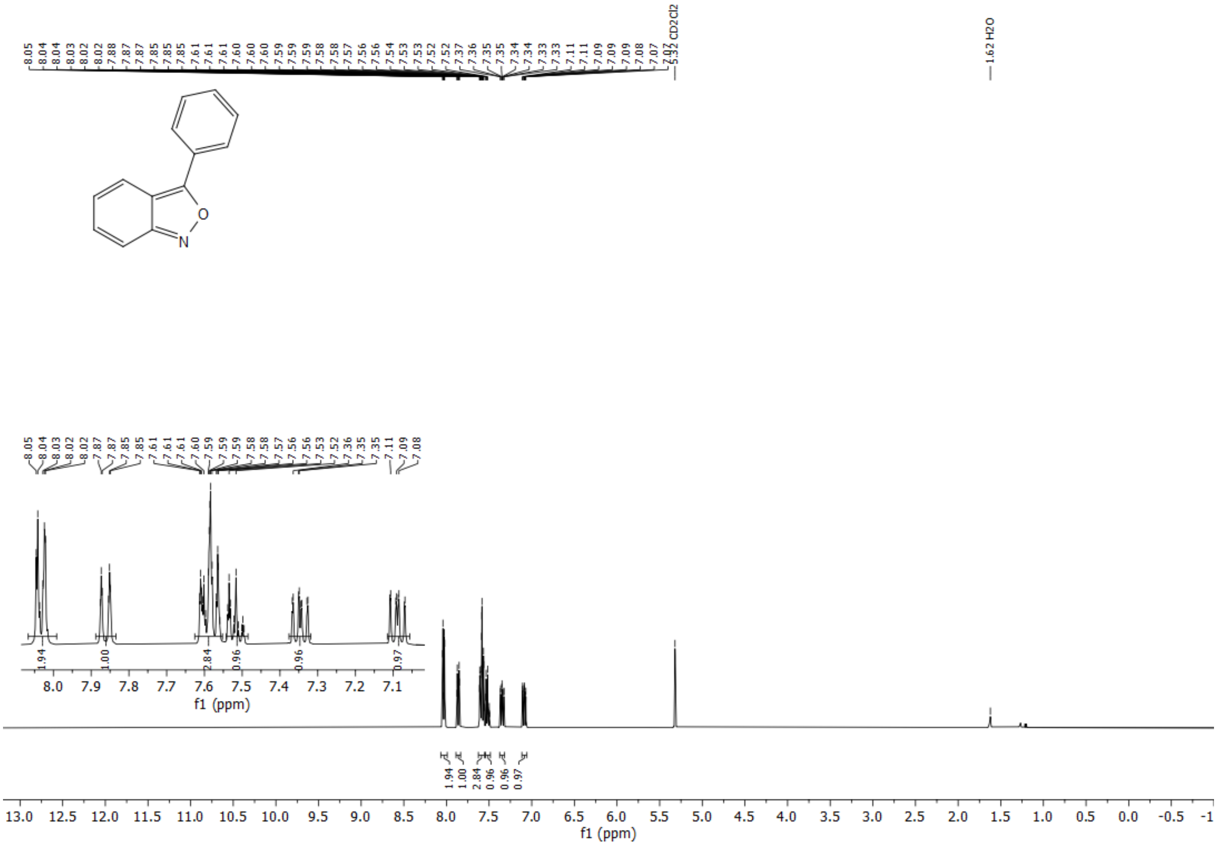

^{13}C NMR spectrum (101 MHz, CD_2Cl_2) of **5al**.

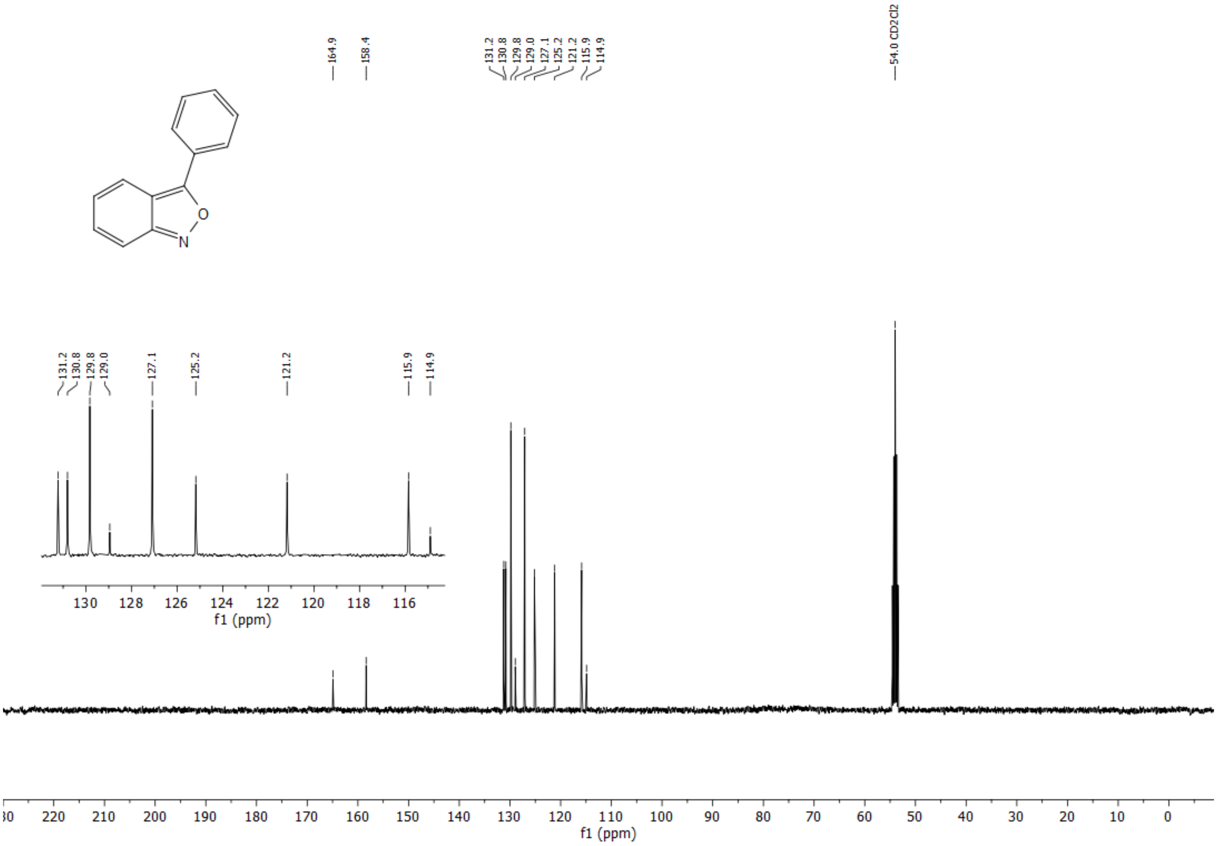

^1H NMR spectrum (400 MHz, CD_2Cl_2) of **5am**.

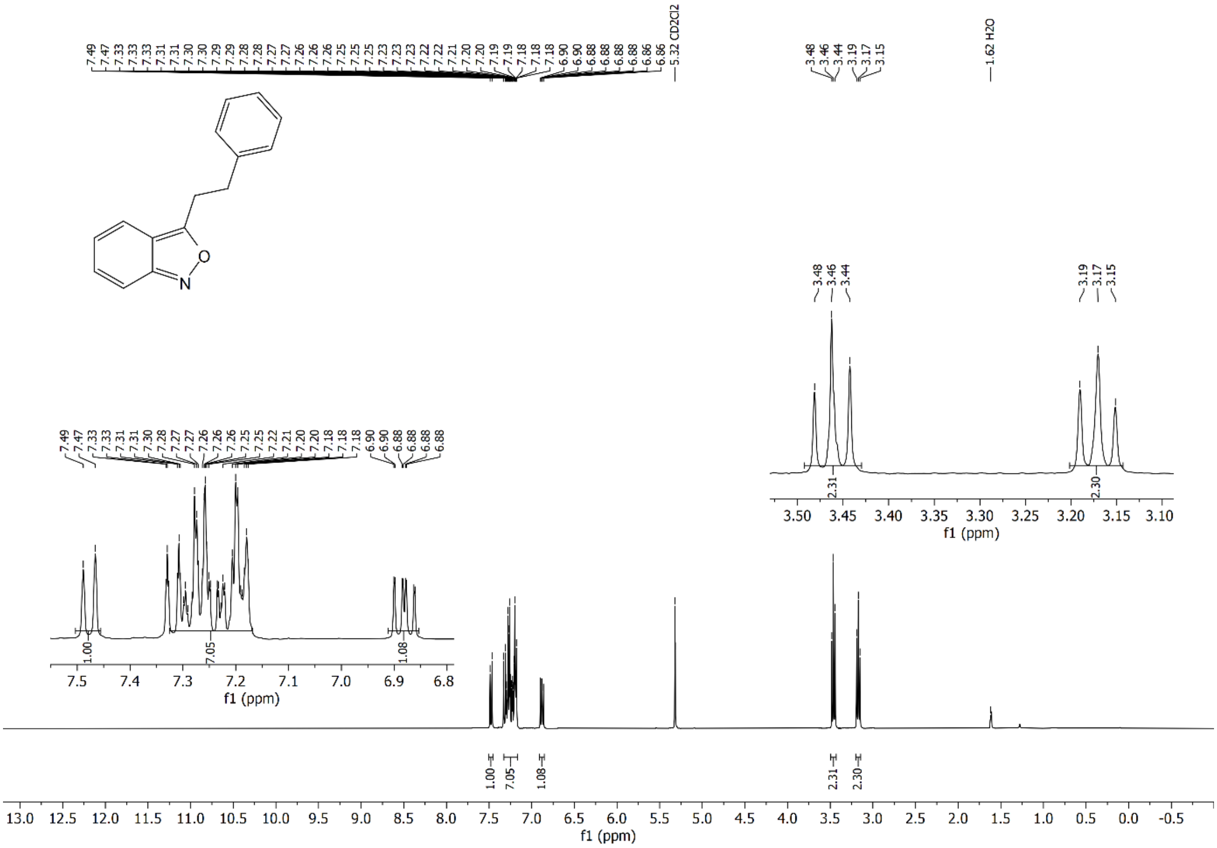

^{13}C NMR spectrum (101 MHz, CD_2Cl_2) of **5am**.

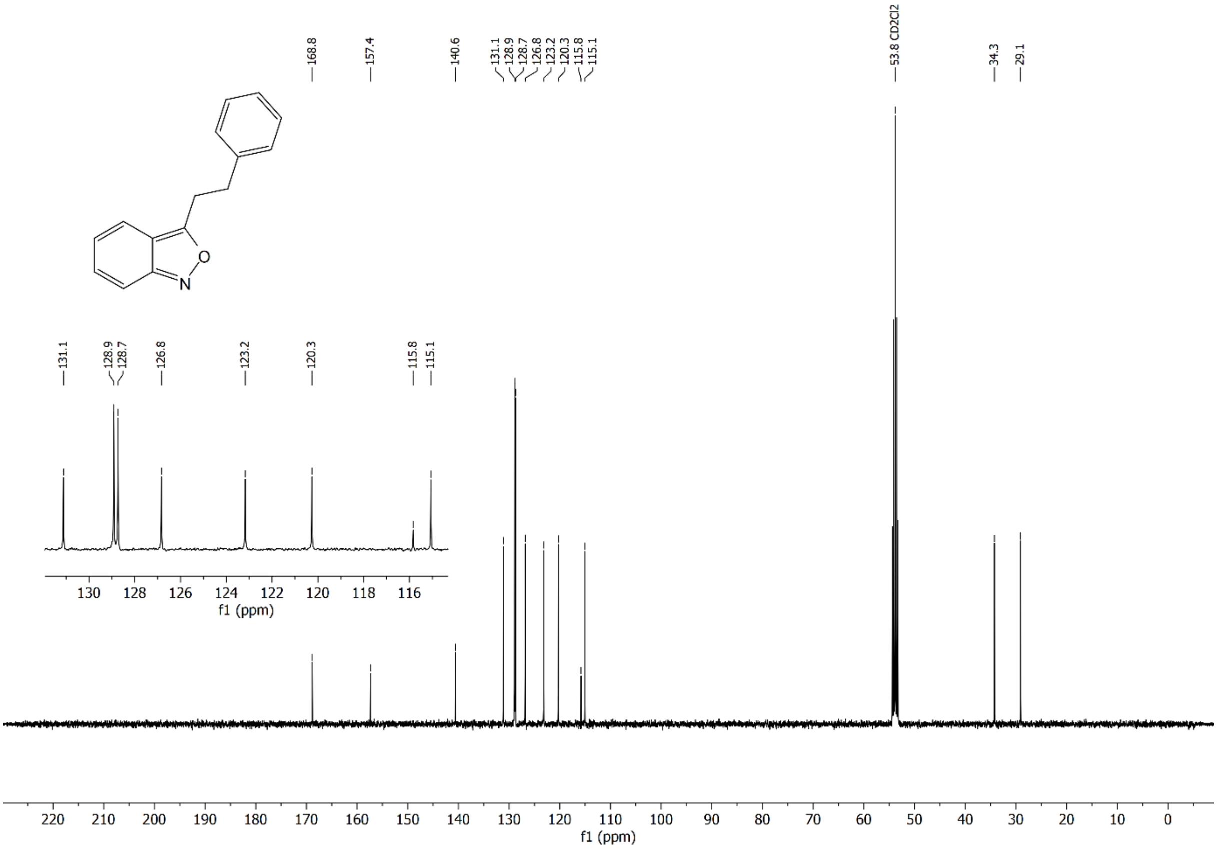

10. Supplemental References

1 C. Gütz, M. Bänziger, C. Bucher, T. R. Galvão and S. R. Waldvogel, *Org. Process Res. Dev.*, 2015, **19**, 1428.

2 E. K. Oehl, P. T. Jirsch, J. Hammes, A. Stenglein, M. Méndez, S. Ruf and S. R. Waldvogel, *J. Org. Chem.*, 2024.

3 Dennis Pollok, Barbara Gleede, Andreas Stenglein and Siegfried R. Waldvogel, *Aldrichimica Acta*, 2021, **54**, 3.

4 A. V. Vorogushin, X. Huang and S. L. Buchwald, *J. Am. Chem. Soc.*, 2005, **127**, 8146.

5 E. Benzing, *Angew. Chem.*, 1959, **71**, 521.
